# To Target or Not to Target *Schistosoma mansoni* Cyclic Nucleotide Phosphodiesterase 4A?

**DOI:** 10.3390/ijms24076817

**Published:** 2023-04-06

**Authors:** Yang Zheng, Susanne Schroeder, Georgi K. Kanev, Sanaa S. Botros, Samia William, Abdel-Nasser A. Sabra, Louis Maes, Guy Caljon, Carmen Gil, Ana Martinez, Irene G. Salado, Koen Augustyns, Ewald Edink, Maarten Sijm, Erik de Heuvel, Iwan J. P. de Esch, Tiffany van der Meer, Marco Siderius, Geert Jan Sterk, David Brown, Rob Leurs

**Affiliations:** 1 Division of Medicinal Chemistry, Amsterdam Institute of Molecular and Life Sciences, Vrije Universiteit Amsterdam, 1081 HZ Amsterdam, The Netherlands; y.zheng@vu.nl (Y.Z.); gkkanev@gmail.com (G.K.K.); ewald.edink@inholland.nl (E.E.); sijm.maarten@gmail.com (M.S.); erikdeheuvel8@gmail.com (E.d.H.); i.de.esch@vu.nl (I.J.P.d.E.); t.k.vander.meer@vu.nl (T.v.d.M.); m.siderius@vu.nl (M.S.); g.j.sterk@vu.nl (G.J.S.); 2 School of Biosciences, University of Kent, Canterbury CT2 7NJ, UK; s.schroeder.uk@gmail.com (S.S.); david.brown@servier.com (D.B.); 3 Pharmacology Department, Theodor Bilharz Research Institute, Warrak El-Hadar, Imbaba, P.O. Box 30, Giza 12411, Egypt; sanaabotros113@gmail.com (S.S.B.); abdelnassersabraa@yahoo.com (A.-N.A.S.); 4 Parasitology Department, Theodor Bilharz Research Institute, Warrak El-Hadar, Imbaba, P.O. Box 30, Giza 12411, Egypt; samiaw2006@yahoo.com; 5 Laboratory of Microbiology, Parasitology and Hygiene (LMPH), University of Antwerp, Universiteitsplein 1, 2610 Wilrijk, Belgium; louis.maes@uantwerpen.be (L.M.); guy.caljon@uantwerpen.be (G.C.); 6 Centro de Investigaciones Biologicas (CIB-CSIC), Ramiro de Maeztu 9, 28040 Madrid, Spain; carmen.gil@csic.es (C.G.); ana.martinez@csic.es (A.M.); 7 Medicinal Chemistry, Faculty of Pharmaceutical, Biomedical and Veterinary Sciences, University of Antwerp, Universiteitsplein 1, 2610 Antwerp, Belgium; irenegarciasalado@gmail.com (I.G.S.); koen.augustyns@uantwerpen.be (K.A.)

**Keywords:** *Schistosoma mansoni*, phosphodiesterase, drug target

## Abstract

Schistosomiasis is a neglected tropical disease with high morbidity. Recently, the *Schistosoma mansoni* phosphodiesterase SmPDE4A was suggested as a putative new drug target. To support SmPDE4A targeted drug discovery, we cloned, isolated, and biochemically characterized the full-length and catalytic domains of SmPDE4A. The enzymatically active catalytic domain was crystallized in the apo-form (PDB code: 6FG5) and in the cAMP- and AMP-bound states (PDB code: 6EZU). The SmPDE4A catalytic domain resembles human PDE4 more than parasite PDEs because it lacks the parasite PDE-specific P-pocket. Purified SmPDE4A proteins (full-length and catalytic domain) were used to profile an in-house library of PDE inhibitors (PDE4NPD toolbox). This screening identified tetrahydrophthalazinones and benzamides as potential hits. The PDE inhibitor **NPD-0001** was the most active tetrahydrophthalazinone, whereas the approved human PDE4 inhibitors roflumilast and piclamilast were the most potent benzamides. As a follow-up, 83 benzamide analogs were prepared, but the inhibitory potency of the initial hits was not improved. Finally, **NPD-0001** and roflumilast were evaluated in an in vitro anti-*S. mansoni* assay. Unfortunately, both SmPDE4A inhibitors were not effective in worm killing and only weakly affected the egg-laying at high micromolar concentrations. Consequently, the results with these SmPDE4A inhibitors strongly suggest that SmPDE4A is not a suitable target for anti-schistosomiasis therapy.

## 1. Introduction

Schistosomiasis is one of the most important neglected tropical diseases in the world [1,2]. More than 780 million people are at risk of infection, and about 261 million are infected in 78 countries, of which 85% reside in sub-Saharan Africa [3]. No vaccine is available yet, and almost all control relies on the single drug praziquantel (PZQ), which has been considered the drug of choice since the 1970s as it is highly effective against all *Schistosoma* species after a single oral dose [4,5,6]. However, its efficacy is dependent on the age of the infection, the sex of the worms and their paired or unpaired status [4,7]. Moreover, alarming indications about the development of resistance were reported [6]. For the above reasons, the search for new anti-schistosomal targets and effective anthelmintic drugs still remains a high priority.

Cyclic nucleotide phosphodiesterases (PDEs) are important enzymes that hydrolyze the second messenger molecules cyclic AMP (cAMP) and cyclic GMP (cGMP) to their non-cyclized analogs AMP and GMP [8]. So far, eleven families of PDEs have been found in humans, and some of them are key drug targets in a variety of human diseases, for example, PDE3, PDE4 and PDE5 [9,10,11,12]. The best-known example is PDE5, for which sildenafil (Viagra^®^, Pfizer, New York, NY, USA) was successfully developed for the treatment of erectile dysfunction [12]. Next to PDE5 inhibitors, PDE4 inhibitors like roflumilast (Daxas^®^, Nycomed, Zurich, Switzerland) have found therapeutic use as an oral drug to treat chronic obstructive pulmonary disease (COPD) [13].

Besides their important roles in human physiology, PDEs are also found in parasites and have been hypothesized as potential anti-parasitic drug targets [14]. Due to the ample experience and knowledge of human PDE research, the last decade has witnessed a growing interest in discovering novel treatments by targeting PDEs of different parasite species. In 2007, *Trypanosoma brucei* PDEB1 and B2 were reported as promising drug targets for the treatment of Human African Trypanosomiasis [15]. Following this breakthrough, researchers adopted different approaches in the search for novel anti-trypanosomal drugs [16,17,18,19,20]. With TbrPDEB1 and B2 being the most validated targets, the related *Leishmania* PDEs, *Plasmodium falciparum* PDEs and the single *Giardia lamblia* PDE were also proposed as putative targets for anti-parasitic drug discovery [21,22,23].

Previous screening of the Sigma-Aldrich Library of Pharmacologically Active Compounds (LOPAC) to identify inhibitors of in vitro *S. mansoni* miracidial transformation resulted in the identification of a number of compounds that regulate the cAMP levels in miracidia [24]. Raising cAMP levels with the adenylate cyclase activator forskolin or with the non-selective PDE inhibitor isobutylmethylxanthine (IBMX) inhibited larval transformation [24], indicating that the control of the parasitic cyclic nucleotide homeostasis is at least critical for one process in the life cycle of *S. mansoni.* Within the EU-funded consortium PDE4NPD aiming at investigating the potential of parasite PDEs as drug targets, we decided at its start in 2014 to embark on a PDE target discovery program for *S. mansoni* [25]. Munday et al. [26] identified 11 different PDE open reading frames in the *S. mansoni* genome and cloned 10 of these SmPDEs (ranging from 517 to 1081 amino acids) from cDNA. Moreover, using different expression systems (yeast, *T. brucei*), enzymatic activity was documented for six of the SmPDEs [26], making these parasite PDEs attractive new drug targets.

While progressing these SmPDEs for drug discovery efforts within PDE4NPD, Long et al. proposed in 2017 the cAMP-specific phosphodiesterase SmPDE4A as a potential drug target [27]. A phenotypic screen of *S. mansoni* somules with 1085 benzoxaborole compounds from Anacor Pharmaceuticals revealed parasite hypermotility and degeneration associated with a set of human PDE4 inhibitors [28,29]. Thereafter, PDE4-like SmPDE genes were identified in the genome of the parasite, with one of them (SmPDE4A) being expressed for biological evaluation. Based on these experiments, a relationship between enzyme inhibition and parasite hypermotility was established and confirmed in experiments with SmPDE4A-transgenic *Caenorhabditis elegans* [27]. Based on these results, Long et al., (2017) identified SmPDE4A as a potential drug target for schistosomiasis [27].

In this work, we describe the expression, purification and biochemical characterization of both full-length and a catalytic domain construct of SmPDE4A. Moreover, the catalytic domain of SmPDE4A was employed for structural studies, and the X-ray structures of both the apo- and cAMP-bound SmPDE4A were solved. Finally, a PDE-focused compound library was used to identify new SmPDE4A inhibitors in an effort to validate this enzyme as a potential anti-schistosomal target.

## 2. Results and Discussion

### 2.1. Protein Purification and Biochemical Characterization

**Figure 1:**
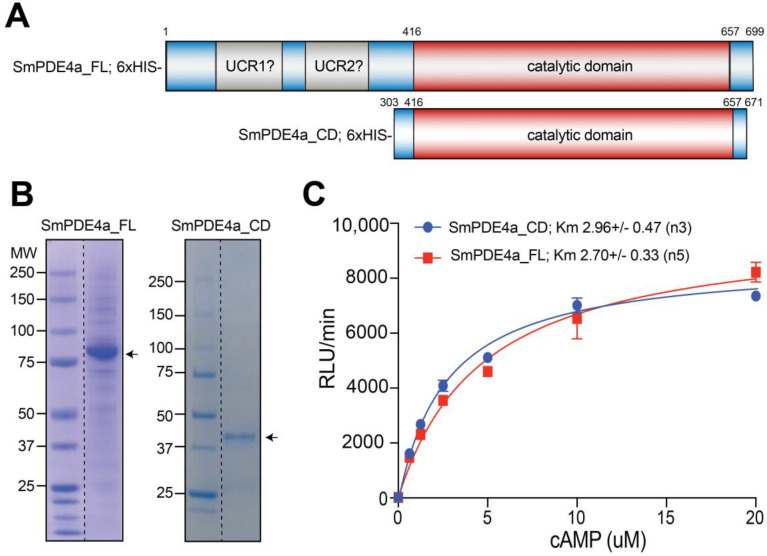
Expression and functional comparison of SmPDE4a_FL and SmPDE4a_CD. (**A**) Schematic view of the constructs for *SmPDE4a*_FL (amino acids 1–699) and *SmPDE4a*_CD (amino acids 303–671) with a 6×His tag on the *N*-terminus. The SmPDE4A_FL protein contains next to the catalytic domain (amino acids 416–657 in the red box) two potential regulatory UCR domains (gray boxes). (**B**) SDS-PAGE analysis of the purified SmPDE4A_FL and SmPDE4A_CD (indicated with an arrow) with expected molecular weights of 80.0 and 42.3 kDa, respectively. (**C**) Michaelis–Menten kinetics, as analyzed with GraphPad Prism, version 8, of both SmPDE4A enzyme constructs at 10 nM and 11 nM for, respectively, SmPDE4A_FL and SmPDE4A_CD.

After cloning the *SmPDE4a* gene [26], we set out to characterize this parasite PDE biochemically, pharmacologically, as well as structurally to enable a structure-based drug design program aiming to target SmPDE4A. To this end, two 6×His-tagged *SmPDE4a* constructs were generated, PCR amplified and cloned into pOPINF encoding a cleavable *N*-terminal 6×His tag. This enabled the expression of both a full-length (FL) protein (699 aa) and the SmPDE4A catalytic domain (CD) encompassing amino acids 303-671 (Figure 1A). The catalytic domain construct of *S. mansoni* PDE4A was based on secondary structure prediction and alignments with published human PDE4B catalytic domain constructs. The SmPDE4A proteins could be purified respectively from extracts of *Sf*21 insect cells (FL) and *E. coli* (CD), using affinity purification in combination with ion exchange and size exclusion chromatography. After removal of the 6×His tag, analysis of the purified protein fractions by SDS-PAGE indicated that the fractions mainly contained the SmPDE4A proteins with the expected molecular masses of 80.0 (SmPDE4A_FL) and 43.2 kDa (SmPDE4A_CD) (Figure 1B). Both constructs displayed cAMP hydrolyzing activity in our biochemical assay, and analysis of the Michaelis–Menten kinetics results in comparable micromolar *K*_m_ values for the substrate cAMP (Figure 1C; SmPDE4A_FL: *K*_m_ = 2.96 ± 0.47 µM, *n* = 3; SmPDE4A_CD: *K*_m_ = 2.70 ± 0.33 µM, *n* = 5).

### 2.2. Crystallization and X-Ray Structure of SmPDE4A Catalytic Domain

**Figure 2:**
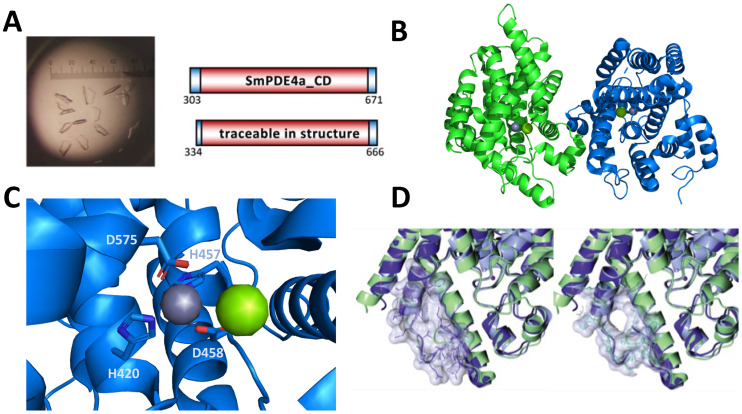
Crystal structure of SmPDE4A and comparison with other PDEs. (**A**) Crystallization of SmPDE4A_CD in 0.4 M ammonium sulfate 12–14% (*v/v*) PEG 3350 and structural features of the catalytic domain of SmPDE4A. (**B**) The protein crystallizes in a dimer form and contains two metal ions (the grey ball represents Mg^2+^, and the purple ball represents Zn^2+^). (**C**) These metal ions are coordinated by a number of histidine and aspartic acid residues in the active site. (**D**) the SmPDE4A structure (dark purple) is superimposed on the catalytic domain structures of LmjPDEB1 (PDB code: 2R8Q, green) and hPDE4D (PDB code: 3SL3, light blue). The surface is shown of residues within the P-pocket region in SmPDE4A (left) and LmjPDEB1 (right).

To enable structure-based drug discovery programs, we crystalized the catalytic domain of SmPDE4A (SmPDE4A_CD). The protein was recombinantly produced in *E. coli* and purified by metal affinity chromatography, ion exchange and size exclusion, as described in the experimental section. Crystals were obtained by hanging drop vapor diffusion at 4 °C in conditions containing 14–16% (*v*/*v*) PEG 3350, 0.4 M ammonium sulfate (Figure 2A). The amino acid residues 334–666 (Figure 2A) were traceable in the X-ray diffractions. The structure of SmPDE4A_CD in complex with cAMP was solved to 2.1 Å (PDB code: 6EZU [30]) by molecular replacement using the human PDE4D catalytic domain structure (PDB code: 3SL4) and further employed to solve the SmPDE4A_CD apo structure (PDB code: 6FG5) at 2.3 Å resolution (Figure 2B).

The apo structure (Figure 2B) of the SmPDE4A catalytic domain and its complex with cAMP (traceable residues 334–671) were determined by X-ray diffraction with two molecules (cAMP and AMP, Figure S2 [31]) in the crystallographic asymmetric unit. The loop (E545 to N556) of a symmetry-related SmPDE4A molecule is folding into the active site of SmPDE4A molecule B whereas the active site of SmPDE4A molecule A appears to be completely accessible to ligands. A monomer of the SmPDE4A catalytic domain is comprised of 16 helices arranged in a similar topology to those of the catalytic domains of human [32] and other parasite PDEs (Figure 2B) [21,33,34]. The two divalent metal ions bound in the active site have been designated Zn^2+^ and Mg^2+^ as the commonly reported ions in PDEs without further verification. The metal ions each form an octahedral geometry with Zn^2+^ coordinating H421 (H^MB.3^), H457 (H^MB.4^), D458 (D^MB.5^), D575 (D^MB.22^) and Mg^2+^coordinating D458 (D^MB.5^) (Figure 2C). The residue nomenclature within brackets follows the PDEStrIAn nomenclature introduced by Jansen et al. [35]. This nomenclature is particularly useful as it allows easy comparison of corresponding residues for all human and parasite PDEs for which structural information is available. The secondary structure elements of SmPDE4A are very similar to those of hPDE4D with an RMS of 0.79 (Figure 2D), whereas those of TcrPDEC, LmjPDEB1 and TbrPDEB1 have a slightly higher RMS with 1.45, 1.71 and 1.80, respectively. For all kinetoplastid PDEs, LmjPDEB1 [21], TbrPDEB1 [34] and TcrPDEC [33], the presence of a parasite-specific pocket (P-pocket) was observed in their X-ray structures and targeted as a selectivity-pocket to generate parasite-specific inhibitors. This pocket, first observed in PDEB1 from *L. major* and originally designated the L-pocket [21], is usually made up of residues of the M-loop and helix H149. In human PDEs, this pocket is either blocked by two large gating residues or disappears because of variations in sequence in H14, H15 and the M-loop. Comparison of the overall SmPDE4A_CD structure with that of the human PDE4D_CD and LmjPDEB1_CD structures (Figure 2D) revealed the absence of a ‘P-pocket’ in the catalytic site of the *S. mansoni* enzyme.

**Figure 3:**
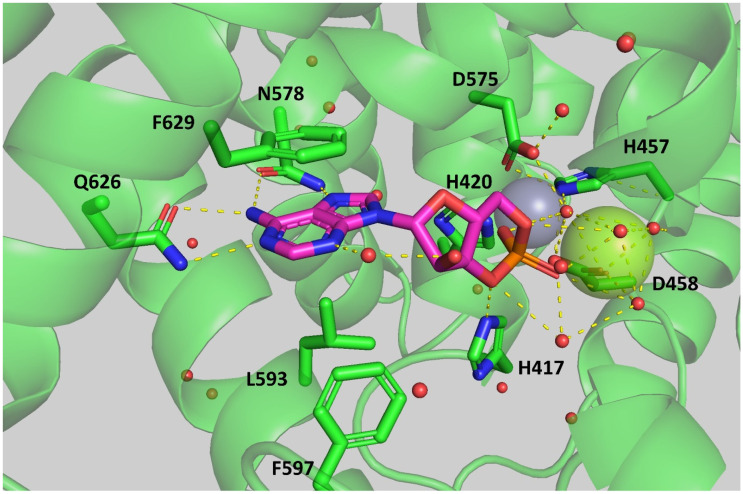
Interaction of the catalytic domain of SmPDE4A with cAMP (purple) as found in the chain A of 6FG5. The cyclic phosphate group interacts with the Mg^2+^ (green sphere) and Zn^2+^ (grey sphere) ions and interferes with the water (red dots) network on the left side of the substrate binding pocket.

As mentioned, we also succeeded in obtaining ligand-occupied crystals after soaking the crystals with 2 mM cAMP (Figure 3). In this dimeric structure (PDB code: 6EZU [30]), we observed the presence of cAMP in the catalytic site of chain A (Figure 3). Interestingly, its product AMP after enzymatic conversion was observed in chain B of the dimeric complex, indicating that the crystals still have catalytic activity. The substrate cAMP is bound by the catalytic domain of SmPDE4A via H-bonds of the adenine moiety to the invariant glutamine residue Q626 (Q^Q.50^) and hydrophobic interactions with the so-called hydrophobic clamped formed by F629 (F^HC.52^), L593 (L^HC.32^) and F597 (F^S.35^; Figure 3). The phosphate moiety points towards the metal ions in the substrate binding site and interacts with the extensive water network surrounding the bivalent ions. For its hydrolyzed product AMP, similar interactions are observed as for cAMP, now with the two hydroxyl groups on the tetrahydrofuran ring being exposed to the solvent (Figure S2B).

### 2.3. Screening of a PDE-Inhibitor Toolbox

**Figure 4:**
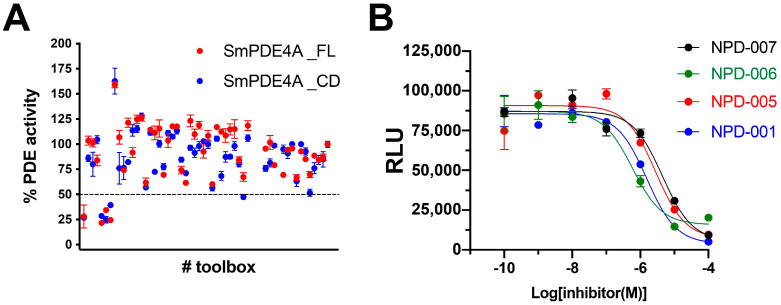
PDE4NPD Toolbox screen to identify inhibitors of SmPDE4A. (**A**) Inhibition of SmPDE4A_FL (red circles) and SmPDE4A_CD activity (blue circles) after incubation with PDE inhibitors at 10 µM. (**B**) Representative dose-response curves for inhibition of the enzymatic activity of SmPDE4A_CD with the toolbox hits **NPD-0001** (blue; pIC_50_ = 5.8 ± 0.06), **NPD-0005** (red; pIC_50_ = 5.5 ± 0.14), **NPD-0006** (green; pIC_50_ = 6.2 ± 0.10) and **NPD-0007** (black; pIC_50_ = 5.4 ± 0.11).

To identify potential new SmPDE4A inhibitors, we used the purified proteins to screen a set of known PDE inhibitors collected in a so-called PDE4NPD Toolbox (Table S1). In the EU-KP7-funded consortium PDE4NPD, we aimed to screen known PDE inhibitors as new pharmacological tools for parasite PDEs. We collected a series of known (from literature or patents) potent inhibitors for all 11 human PDEs, both available via commercial vendors or otherwise by in-house synthesis. The PDE4NPD toolbox contained 38 chemically diverse compounds with submicromolar IC_50_ values against human and some parasite PDEs, like the PDE4 inhibitor roflumilast, the PDE5 inhibitor sildenafil or the TbrPDEB1 inhibitor **NPD-0001** (Table S1 for all inhibitors). The PDE4NPD toolbox compounds were initially tested at 10 µM against both purified SmPDE4A proteins (full-length and catalytic domain). Like with the *K*_m_ value for cAMP, no significant difference was observed for the inhibition of SmPDE4A_FL and SmPDE4A_CD proteins with this set of compounds (Figure 4A and Figure S1), further strengthening the notion that the SmPDE4A_FL and SmPDE4A_CD proteins are biochemically behaving similarly.

From the toolbox screening (Figure 4A), four compounds gave at 10 µM >50% inhibition of the enzymatic activity of the purified SmPDE4A proteins: two tetrahydrophthalazinones, **NPD-0001** and **NPD-0007** and the two closely related benzamides **NPD-0005** and **NPD-0006** (piclamilast and roflumilast, respectively). These compounds were further tested in more detail, and full inhibition curves (Figure 4B) allowed the determination of p*K*_i_ values (Table 1). One of the hit compounds, **NPD-0006** or roflumilast (Daxas^®^, Nycomed, Zurich, Switzerland), is in clinical use for the treatment of severe COPD. This compound was also reported by Long et al. as a potent SmPDE4A inhibitor with an estimated p*K*_i_ value of 7.8 [27]. Evidently, this value is in reasonable agreement with the p*K*_i_ value of 7.1 observed with the purified SmPDE4A catalytic domain.

### 2.4. Docking of Roflumilast in the SmPDE4A Active Site

**Figure 5:**
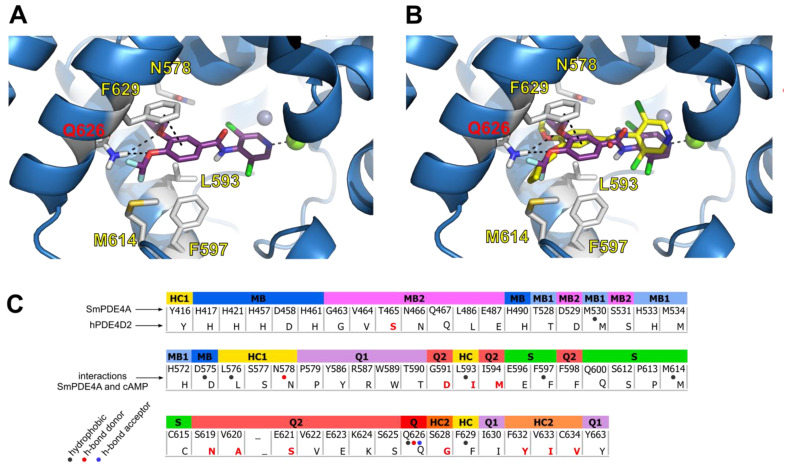
Docking results of roflumilast in SmPDE4A and comparison with the docking in hPDE4D2. (**A**) The docking pose of roflumilast in the catalytic site of SmPDE4A. The residue nomenclature follows the PDEStrIAn database [35]. (**B**) The docking pose of roflumilast in SmPDE4A (purple) and reference roflumilast (yellow) in hPDE4D (PDB:1XOQ). The docking pose makes hydrogen bonds with Q626 (Q^Q.50^) and face-to-face π-stacking interaction with F629 (F^HC.52^) as roflumilast in hPDE4D (PDB:1XOQ). (**C**) Alignment of the binding site residues of the SmPDE4A and hPDE4D2. The color bar above the residues indicates their location in the binding site. The different residues are highlighted in red. The interactions between SmPDE4A and cAMP are shown with dotted points with hydrophobic colored in grey, hydrogen bond donor colored in red and hydrogen bond acceptor colored in blue.

Following the screening of the PDE4NPD toolbox and the identification of **NPD-0006** (roflumilast) as the most potent SmPDE4A inhibitor so far, we tried to generate a SmPDE4A-roflumilast co-crystal. Despite our success in solving the crystal structures of the SmPDE4A catalytic domain in the apo- and cAMP/AMP binding form, we were unfortunately unsuccessful in obtaining a SmPDE4A_CD structure with roflumilast. Therefore, roflumilast was docked in the catalytic site of SmPDE4A_CD using AutoDock Vina (Figure 5A) [36]. Inside the binding pocket, the X-ray structure of SmPDE4A reveals hydrogen bonds of cAMP with the highly conserved glutamine Q626 (Q^Q.50^) and the partially conserved asparagine N578 (N^HC1.25^). While this glutamine is conserved in all PDEs, the specific asparagine is conserved in human PDE4s and PDE9s as well as PDEB1 and 2 in *Leishmania* and *T. brucei* [35]. This glutamine (Q^Q.50^) forms a “hydrophobic clamp” with the conserved phenylalanine (F^S.35^) and an aliphatic valine, leucine or isoleucine residue located at position HC.32 (PDEStrIAn [35] nomenclature, Figure 3). Differences in the residue level between the SmPDE4A and human PDE4 binding pocket are shown in Figure 5C. It can be seen that most residues are conserved, except for a Ser/Thr difference, indicating a huge similarity of the SmPDE4A and human PDE4 binding pockets. To dock roflumilast, the side chains of the residues M614 (M^S.40^) and N578 (N^HC1.25^) were kept flexible to allow the ligand to fully exploit the SmPDE4A binding site. The docking poses were compared to those in hPDE4D crystals (PDB code:1XOQ, Figure 5B) [37]. The best docking pose of roflumilast is shown in Figure 5A (AutoDock Vina score of −8.7 kcal/mol). Like in human PDE4, this docking pose of roflumilast makes hydrogen bonds with Q626 (Q^Q.50^) and face-to-face π-stacking interaction with F629 (F^HC.52^). The 3,5-dichlorpyridine moiety of roflumilast binds to the magnesium ion in the binding pocket of SmPDE4A.

### 2.5. Evaluation of Roflumilast Analogues as SmPDE4A Inhibitors

**Scheme 1:**
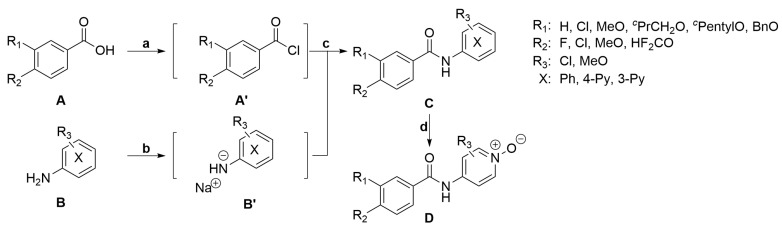
Synthetic route of the roflumilast analog library. Reagents and conditions: a: acid A, THF, (COCl)_2_, *cat*. DMF, 0 °C to RT, 30 min; b: amine/aniline B, NaH, DMF, 0 °C to RT, 30 min; c: A’, B’, 0 °C to RT, 30 min; d: C, *m-*CPBA, DCM, RT, 30 min. Further details of all synthesized compounds can be found in the Supporting Information.

Since roflumilast (**NPD-0006**) turned out to be the most potent inhibitor for SmPDE4A and is also a potent and approved human PDE4 inhibitor [13], we designed an analog library to study the SAR of this chemical class as SmPDE4A inhibitors. For this purpose, synthetic Scheme 1 was defined, which enabled us to introduce a variety of modifications on different positions of roflumilast.

The synthesis starts with the conversion of the properly substituted carboxylic acid to the acyl chloride (**A** to **A**’, step a) and the deprotonation of a substituted aniline group (**B** to **B**’, step b) using NaH in DMF. The addition of the acyl chloride **A’** dissolved in DCM to the deprotonated aniline **B**’ results in the formation of the amide (**C**, step c). If present in the molecule, the pyridine moiety in **C** could be oxidized to the *N*-oxide using 3-chloroperbenzoic acid (*m*-CPBA). Details of the synthesis and analytical characterization of all compounds are available in the Supplementary Information (Scheme S1, Figures S3–S257).

The next tables present an overview of the SAR of our roflumilast analog library at SmPDE4A. Only a small selection of the 83 analogs that were prepared are shown (details of the SmPDE4A inhibitory potencies of all compounds are available in Supplementary Information Table S2). Table 2 summarizes some of the variations in the R_1_ and R_2_ positions of roflumilast. Any investigated modification of the difluoromethoxy group resulted in a strong decrease in inhibitory potency at SmPDE4A. Only the replacement of the difluoromethoxy with the closely related methoxy group in **NPD-3094** leads to a relatively small decrease in activity against SmPDE4A (Table 2). Because of ease of chemistry and the relatively good potency of **NPD-3094** and piclamilast compared to roflumilast, the SAR at R_1_ was investigated with a 4-methoxy group instead of the difluoromethoxy of roflumilast (comparison with **NPD-3094**). Clearly, a replacement of the cyclopropylmethoxy group (**NPD-3094**) or the cyclopentyloxy (piclamilast) to a methoxy group is allowed to some extent (10-fold reduction in activity, Table 2). The unsubstituted, benzyloxy-, or chlorine-substituted R_1_ analogs proved to be inactive against SmPDE4A (Table 2).

Variation in the dichloropyridine part of roflumilast is also allowed to some extent (Table 3). Oxidation of the pyridine (**NPD-2980**) did not significantly affect the SmPDE4A inhibitory potency, whereas removal of one chloride atom (**NPD-1205**) resulted in only a three-fold reduction in potency. When both chloride atoms are removed (**NPD-3076**), a strong reduction in PDE inhibitory potency is observed. A subsequent shift of the nitrogen atom from the 4-position to the 3-position was also not allowed. The replacement of the nitrogen atom in **NPD-1204** by a carbon atom (**NPD-3080**) results in a 10-fold reduction in potency. Any other modification results in a complete loss of SmPDE4A inhibitory potency (Table 3).

### 2.6. Effect of Identified SmPDE4A Inhibitors on S. mansoni Survival and Ovipositioning

To validate the chemical classes of SmPDE4A inhibitors for their potential against schistosomiasis, we decided to test the most potent compounds, the tetrahydrophthalazinone NPD-0001 and the benzamide roflumilast for anti-*S.mansoni* activity (Table 4). The reference drug praziquantel was 100% effective on both worm killing and ovipositioning reduction at the lowest concentration tested (5 µM). Roflumilast did not induce worm killing but affected ovipositioning to some extent. The in vitro data on roflumilast have already been disclosed by Botros et al. [38]. Since *S. mansoni* eggs are responsible for the ultimate inflammation, this observed effect on ovipositioning, despite being incomplete, prompted us to test also the structurally unrelated SmPDE4A inhibitor tetrahydrophthalazinone NPD-0001. At the highest concentration (100 µM), NPD-0001 only affected the survival of the male parasite (63% worm killing), whereas female worms were unaffected (Table 4). At 100 micromolar, NPD-0001 also severely affected the egg-laying behavior, but at lower concentrations of NPD-0001, only marginal effects on oviposition were observed. Although the effects of NPD-0001 on both survival and oviposition are significant at 100 µM, these effects could well be explained by the cytotoxic effect of NPD-0001, as observed on human MRC-5_SV40_ lung fibroblasts.

## 3. Materials and Methods

### 3.1. PDE4NPD Toolbox Generation

To pharmacologically characterize SmPDE4A (GenBank accession number MH457509), we screened the so-called PDE4NPD toolbox. This PDE-focused compound library was generated as a resource of the EU-KP7-funded PDE4NPD project. Literature data on selective and non-selective human PDE inhibitors were evaluated, resulting in a series of 38 compounds with sub-micromolar potency against all 11 different human PDE isoforms. To this series, we added some compounds with proven potency (selective or not) against some non-mammalian class-I PDEs of *T. brucei* and *P. falciparum*. The PDE4NPD toolbox selection and information about the acquisition of the individual compounds are summarized in Table S1 [16,18,22,39,40,41,42,43,44,45,46,47,48,49,50,51,52,53,54,55,56,57,58,59,60,61].

### 3.2. Expression and Purification of SmPDE4A Full-Length (FL) and SmPDE4A Catalytic Domain (CD)

The SmPDE4a_FL gene encoding the full-length phosphodiesterase (amino acid 1-699, Figure 1A) protein with 2 potential regulatory Upstream Conserved Regions (UCR) and the catalytic domain (CD) was PCR amplified with a thrombin-cleavable *N*-terminal 6×His tag from a *Trypanosome* codon-optimized template synthesized by BaseClear (Leiden, the Netherlands). The construct was designed using the Integrated DNA Technologies tool [62] with manual checking to remove unwanted restriction sites from the sequences. The amplicon was generated with the appropriate primer extensions (including an upstream *BamH*I and downstream *Sph*I site) for ligation-independent cloning into a pOPINF [63] vector, and recombinant Bacmids were generated. Baculovirus particles were produced by transfecting *Sf*9 (*Spodoptera frugiperda*) cells with recombinant bacmids, which were then adopted to transfect *Sf*21 cells for protein production.

For expression of the catalytic domain SmPDE4a_CD, the gene sequence encoding amino acids 303-671 (Figure 1A) was PCR amplified and directly cloned into vector pOPINF providing the gene product with a cleavable *N*-terminal 6×His tag. This vector was used to transform *E. coli* Rosetta DE3 (pLysS) for protein production. The protein was purified from cleared lysates at 4 °C using a 5 mL HisTrap™ HP column (GE Healthcare, Chicago, IL, USA) charged with NiSO_4_. Unbound proteins were washed off with buffer A (500 mM NaCl, 20 mM imidazole, 20 mM Tris-HCl, pH 8.0, 1 mM dithiothreitol (DTT) and the protein eluted with buffer A containing 400 mM imidazole and immediately passed through a HiPrep 26/10 desalting column (GE Healthcare, Chicago, IL, USA) equilibrated in 20 mM Tris HCl, pH 8.0, 100 mM NaCl, and 2 mM DTT. Thereafter, the protein was subjected to HRV3C protease cleavage at 4 °C overnight. Cleaved material was separated from un-cleaved material by a second gravity flow IMAC and further purified by ion-exchange chromatography on QSepharose (HiTrap™ Q HP, GE Healthcare, Chicago, IL, USA) equilibrated in buffer C (50 mM Tris HCl, pH 8.0, 100 mM NaCl, 1 mM MgCl_2_ and 1 mM DTT). The protein was eluted with a linear gradient from 0–100% buffer D (50 mM Tris HCl, pH 8.0, 500 mM NaCl, 1 mM MgCl_2_ and 1 mM DTT) over 20 column volumes. Finally, aggregates were removed by gel filtration on a Superdex 200 Increase 10/300 GL (GE Healthcare, Chicago, IL, USA) equilibrated in 20 mM Tris HCl, pH 7.5, 100 mM NaCl, 1 mM MgCl_2_, and 1 mM DTT. A typical purification yielded about 3 mg SmPDE4A_CD from a 1-L cell culture.

Full-length SmPDE4A protein was performed using *S. frugiperda* Sf21 cells grown in EX-CELL^®^ 420 SF media (Sigma Aldrich, Burlington, MA, USA) and cells harvested 48 hrs post-infection. The cells were resuspended in buffer A and lysed by sonication. The protein was purified from cleared lysates at 4 °C using a 1 mL HisTrap™ HP column (GE Healthcare, Chicago, IL, USA) charged with NiSO_4_. Unbound proteins were washed off with buffer A, and the protein eluted with buffer A containing 400 mM imidazole and immediately passed through a HiPrep 26/10 desalting column (GE Healthcare, Chicago, IL, USA) equilibrated in 20 mM Tris HCl, pH 8.0, 150 mM NaCl, and 2 mM DTT. Thereafter, the protein was subjected to HRV3C protease cleavage at 4 °C overnight. Cleaved material was separated from un-cleaved material by a second gravity flow IMAC and further purified by gel filtration on a Superdex 200 Increase 10/300 GL (GE Healthcare, Chicago, IL, USA) equilibrated in 20 mM Tris HCl, pH 7.5, 150 mM NaCl, 1 mM MgCl_2_ and 1 mM DTT. A typical purification yielded about 5 mg SmPDE4A_FL from a 1-L cell culture.

### 3.3. Measurement of Phosphodiesterase Activity

Phosphodiesterase activity assays were performed using the PDELight™ HTS cAMP phosphodiesterase Kit (Lonza, Walkersville, MD, USA) at 25 °C in non-binding, low volume 384-well plates (Corning, Kennebunk, ME, USA). To biochemically characterize the purified proteins, the phosphodiesterase activity of SmPDE4A_FL (stock concentration 0.6 mg/mL, 7.4 µM) and SmPDE4A_CD (stock concentration 0.24 mg/mL, 5.6 µM) were determined at various concentrations of cAMP (1–20 µM). Since we noticed some differences in the stability of the purified proteins upon storage, the enzymatic activity was measured for each batch of protein before each experiment, resulting in the use of a concentration of the PDE displaying linear concentration dependency of enzyme activity and converting less than 20% of the substrate cAMP, following the recommendations of the supplier (Lonza, Walkersville, MD, USA). Enzyme velocity was plotted against substrate concentration, and *K*_m_ values were determined using GraphPad Prism software version 8, analyzing the Michaelis–Menten kinetics of the enzyme.

### 3.4. PDE Inhibitor Screening

PDE activity measurements (cAMP concentration at 2 × *K*_m_ (6 µM)) were made in ‘stimulation buffer’ (50 mM HEPES, 100 mM NaCl, 10 mM MgCl_2_, 0.5 mM EDTA, 0.05 mg/mL BSA, pH 7.5). Inhibitors were initially tested at 10 µM (triplicate measurements/assay, *n* = 3). In follow-up experiments, dose-response curves were made in the range 100 µM–10 pM (triplicate measurements/assay, *n* = 3). Test compounds were diluted in DMSO (final concentration in assay was 1%), and 2.5 µL aliquots were transferred to the 384-well plate, 2.5 µL of the PDE in stimulation buffer was added and mixed (leading to a final concentration of 10 nM and 11 nM for respectively SmPDE4A_FL and SmPDE4A_CD), 5 µL cAMP (at 2 × *K*_m_) was added, followed by incubation for 20 min at 300 rpm in 96-well plate shaker at room temperature. The reaction was terminated by the addition of 5 µL Lonza Stop Buffer supplemented with 10 µM pan-PDE4 inhibitor **NPD-0001**. Subsequently, 5 µL of Lonza Detection reagent (diluted to 80% with reaction buffer) was added, and the reaction was incubated for 10 min at 300 rpm. Luminescence was read with a Victor3 luminometer using a 0.1 s/well program. The determination of IC_50_ values for the PDE inhibitors was performed using GraphPad Prism statistics software version 8 under the assumption that the PDE inhibitors are competitively blocking the cAMP binding to the catalytic side. The Cheng-Prussoff equation, embedded in the GraphPad Prism software, was used to convert IC_50_ values to p*K*_i_ values.

### 3.5. SmPDE4A Protein Crystallography

Crystals of the purified SmPDE4A_CD were grown by hanging drop vapor diffusion (0.4 M ammonium sulfate 12–14% (*v/v*) PEG 3350) and protein-ligand complexes were generated either by soaking or co-crystallization. Diffraction data were collected at Diamond Light Source synchrotron beamlines I03 and I04-1 using a Pilatus3 6M and a Pilatus 2M detector, respectively, integrated and reduced with auto-processing pipeline available at the beamlines or by using IMOSFLM and AIMLESS programs as integrated into the Collaborative Computational Project Number 4 (CCP4) suite [64]. Structure solution was performed either by Molecular Replacement using PHASER (CCP4) or by Fourier synthesis. Adjustment of models was carried out using COOT [65] and refinement with REFMAC5 (CCP4). All details on the X-ray structures have been submitted at the PDB and can be accessed via entries 6FG5 (apo) and 6EZU (cAMP/AMP bound) [30].

### 3.6. Docking of Roflumilast in the SmPDE4A Crystal Structure

The ligand and the cAMP-liganded SmPDE4A_CD structure (PDB code: 6EZU [30]) were obtained from the PDEStrIAn database [35]. The protein, ligand and 3D grid were prepared with AutoDockTools v1.5.6. The coordinates of the center of the 3D grid were defined as: center_x = 17.609, center_y = 13.197, center_z = 47.972. The dimensions of the grid were: 26, 24 and 28 (for x, y and z, respectively). The docking was performed with AutoDock Vina v1.1.2 [36] using the default settings. The residues M614 (M^S.40^) and N578 (N^HC1.25^) were kept flexible.

### 3.7. Determination of Anti-Schistosomal Activity of SmPDE4A Inhibitors

Infected Syrian golden hamsters (Mesocrietus auratus) bred and maintained at the Schistosome Biology Supply Center (SBSC) of Theodor Bilharz Research Institute (TBRI), Giza, Egypt, were used. Animals were housed under controlled biological conditions. Animal experiments were conducted in accordance with the Guide for Care and Use of Laboratory Animals and were approved by the Institutional Review Board (IRB) of TBRI. Seven weeks post-infection, hamsters were anesthetized using pentobarbital in a dose of 200 mg/kg body weight and 2 hamsters were sacrificed to obtain the required number of worms. The animals were then perfused using a normal saline solution (0.9% NaCl), and worms were collected [66]. Six to 8 worms per well were placed in a 12-well tissue culture plate, taking care to guarantee 1 to 2 worm pairs/well and fresh RPMI-1640 medium (plus glutamine, 20% newborn calf serum and antibiotics [streptomycin, penicillin and gentamicin]), containing the indicated concentration of the test compound, was added [67,68]. Worms were incubated overnight in a CO_2_ incubator at 37 °C. On the 2nd day, worms were examined by microscopy, washed 3 times with normal saline, a fresh medium was added, and the incubation was continued. On the 3rd day, worm motility was observed, and on the 4th day, the medium was again changed. On day 5 (end of the observation period), worms were microscopically examined for their motility and appearance. Each concentration was tested in duplicate wells, and the final recording of percent worm mortality was determined as the number of dead worms [contracted and opaque] divided by the total number of worms ×100).

For these experiments, roflumilast and NPD-0001 were freshly prepared in different concentrations (100 µM, 50 µM, 25 µM 10 µM and 5 µM) in RPMI-1640 medium and tested in 12-well tissue culture plates for *S. mansoni* survival and ovipositing capacity. To assess the effects of roflumilast and NPD-0001 on egg production in vitro, we used 12-well tissue culture plates containing 6–8 worms, including at least 1 or 2 worm couples and fresh RPMI-1640 medium containing the indicated concentration of the test compound. Worms were incubated overnight in a CO_2_ incubator at 37 °C. On the 2nd day, worms were washed 3 times with normal saline, fresh medium was added, and the incubation was continued. Each concentration was tested in duplicate wells, and on the 4th day, eggs were counted and discarded, and the medium was changed. On day 5 (the end of the observation period), newly deposited eggs were counted. The final egg number is the total count of days 4 and 5 for each concentration tested. Statistical analysis was done with Student’s unpaired, 2-tailed *t*-test.

### 3.8. Synthesis

We prepared a library of 83 compounds to derive the structure-activity relationship (SAR) for roflumilast analogs in the context of SmPDE4A inhibition. All starting materials were obtained from commercial suppliers and used without purification. Anhydrous THF and DMF were obtained by passing through an activated alumina column prior to use. TLC analyses were performed using Merck F_254_ aluminum-backed silica plates and visualized with 254 nm UV light. Flash column chromatography was executed using Biotage Isolera equipment.

All compounds were obtained with the following general procedure: under an N_2_ atmosphere in a dry flask, oxalyl chloride (4.8 mmol, 1.2 eq.) was added dropwise to a mixture of carboxylic acid A (4.0 mmol, 1.0 eq.) and DMF (100 μL) in THF (20 mL) at 0 °C. The mixture was allowed to reach room temperature and stirred for an additional 30 min to obtain the corresponding acyl chloride. In another dry flask with an N_2_ insert, NaH (60% dispersion in mineral oil, 10.0 mmol, 2.5 eq.) was added in small portions to amine/aniline B (10.0 mmol, 2.5 eq.) in DMF (20 mL) at 0 °C. The suspension was allowed to reach room temperature and stirred for an additional 30 min. To this mixture, the crude acyl chloride solution was added dropwise at 0 °C. The reaction mixture was allowed to reach room temperature and stirred for an additional 30 min. After quenching with a saturated NH_4_Cl solution (150 mL), the aqueous suspension was extracted with EtOAc 3 times. The combined organic layers were washed with brine, dried over Na_2_SO_4_ and concentrated in vacuo. The crude product was purified using flash column chromatography. All compounds were analyzed using LCMS, ^1^H NMR, ^13^C NMR and HRMS and had purity >95% (area% on LC at 254 nm). Details of the synthesis and analytical characterization of all compounds are available in the Supplementary Information (Figures S3–S257). [69,70,71,72,73,74]

## 4. Conclusions

We isolated and characterized both the full-length and the catalytic domain (AAs 303-671) of the *S. mansoni* PDE4A, an enzyme recently suggested as a potential drug target. We tested our PDE toolbox containing 38 diverse human/parasite PDE inhibitors on this enzyme and identified four compounds that gave >50% inhibition at 10 µM belonging to two chemical classes, tetrahydrophthalazinones and benzamides, all of them described in the literature as human PDE4 inhibitors. As the benzamide roflumilast showed the highest potency and is an approved drug (Daxas^®^, Nycomed, Zurich, Switzerland), we decided to design a series of close analogs to study the SAR of this chemical class on SmPDE4A. In this study, we prepared and tested 83 compounds with variations in different parts of this molecule. All these compounds were significantly less active on this enzyme except for the roflumilast *N*-oxide **NPD-2980**, which showed equipotency with roflumilast. For validation, we tested the efficacy of key compounds (**NPD-0001** and roflumilast) on parasite survival and oviposition. Only **NPD-0001** showed significant effects at 100 µM that we believe are directly related to cytotoxicity at this high concentration. The disappointing efficacy of these compounds discourages further consideration of SmPDE4A as a suitable target of a novel anti-schistosomiasis therapy, although at this stage, it cannot be excluded that the tested SmPDE4A inhibitors might, e.g., not cross the tegument and thus not reach their target.

## Figures and Tables

**Table 1:** p*K*_i_ values of the PDE4NPD Toolbox hits for the inhibition of SmPDE4A_CD.

Comp No.	Structure	M.W.	cLogP ^a^	PSA ^a^	SmPDE4A_CD p*K*_i_ ^b^
**NPD-0001**	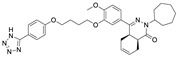	584.7	5.8	114.8	6.9 ± 0.2
**NPD-0007**	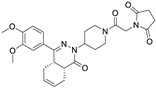	508.6	0.3	108.8	6.3 ± 0.1
**NPD-0006 (roflumilast)**	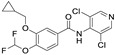	403.2	4.5	60.5	7.1 ± 0.1
**NPD-0005 (piclamilast)**	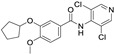	381.3	4.1	60.5	6.5 ± 0.2

^a^: cLogP and PSA generated from Collaborative Drug Discovery (CDD) vault; ^b^: Mean values ± standard error of the mean, *n* ≥ 3.

**Table 2:**
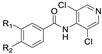
Variations of the R_1_ and R_2_ groups of roflumilast and the effect on physicochemical parameters and SmPDE4A inhibitory potency.

Comp No.	R_1_	R_2_	M.W.	cLogP ^a^	PSA ^a^	SmPDE4A_CD p*K*_i_^b^ or Inhibition at 10 µM (%)
**roflumilast**	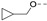	HF_2_CO	403.2	4.5	60.5	7.1 ± 0.1
**piclamilast**	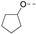	MeO	419.2	2.4	74.5	7.2 ± 0.0
**NPD-3094**	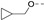	MeO	368.8	3.8	60.5	6.4 ± 0.1
**NPD-3093**	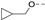	F	368.8	3.8	60.5	5.6 ± 0.0
**NPD-3096**	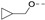	Cl	334.3	3.2	60.5	6.1 ± 0.1
**NPD-0546**	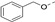	MeO	367.8	5.1	47.6	8%
**NPD-0446**	MeO	MeO	367.8	5.1	47.6	5.4 ± 0.0
**NPD-1180**	Cl	MeO	397.8	4.9	56.8	9%
**NPD-0548**	H	MeO	297.1	2.9	51.2	16%

^a^: cLogP and PSA generated from CDD vault; ^b^: Mean values ± standard error of the mean, *n* ≥ 3.

**Table 3:**
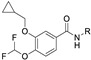
Variations on dichloropyridine part of roflumilast and the effect on physicochemical parameters and SmPDE4A inhibitory potency.

Comp No.	R	M.W.	cLogP ^a^	PSA ^a^	SmPDE4A_CD p*K*_i_^b^ or Inhibition at 10 µM (%)
**roflumilast**	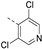	403.2	4.5	60.5	7.1 ± 0.1
**NPD-2980**	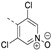	419.2	2.4	74.5	7.2 ± 0.0
**NPD-1204**	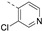	368.8	3.8	60.5	6.6 ± 0.1
**NPD-1205**	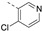	368.8	3.8	60.5	21%
**NPD-3076**	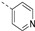	334.3	3.2	60.5	46%
**NPD-3080**	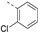	367.8	5.1	47.6	5.7 ± 0.1
**NPD-3075**	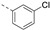	367.8	5.1	47.6	4%
**NPD-3000**	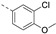	397.8	4.9	56.8	15%

^a^: cLogP and PSA generated from CDD vault; ^b^: Mean values ± standard error of the mean, *n* ≥ 3.

**Table 4:** Effect of SmPDE4A inhibitors on *S. mansoni* survival and oviposition.

Compound	SmPDE4A_CD pKi	Dose (µM)	%Worm Killing (Male)	%Worm Killing (Female)	% Oviposition Reduction	MRC-5 pIC50
**roflumilast ^a^**	7.1 ± 0.1	100	0	0	52 *	<4.2
		50	0	0	49 *	
		25	0	0	42 *	
		10	0	0	39 *	
		5	0	0	35 *	
**NPD-0001**	6.9 ± 0.2	100	63	0	100 *	5.1 ± 0.1
		50	0	0	23	
		25	0	0	13	
		10	0	0	0	
		5	0	0	0	

^a^ data from Botros et al. [38] and this study. * indicates *p* < 0.05, Student’s unpaired, two-tailed *t*-test.

## Data Availability

Not applicable.

## Supplementary Materials

The following supporting information can be downloaded at: https://www.mdpi.com/article/10.3390/ijms24076817/s1.

## References

[1] Kyu H.H., Abate D., Abate K.H., Abay S.M., Abbafati C., Abbasi N., Abbastabar H., Abd-Allah F., Abdela J., Abdelalim A. (2018). Global, Regional, and National Disability-Adjusted Life-Years (DALYs) for 359 Diseases and Injuries and Healthy Life Expectancy (HALE) for 195 Countries and Territories, 1990–2017: A Systematic Analysis for the Global Burden of Disease Study 2017. Lancet.

[2] Murray C.J.L., Vos T., Lozano R., Naghavi M., Flaxman A.D., Michaud C., Ezzati M., Shibuya K., Salomon J.A., Abdalla S. (2012). Disability-Adjusted Life Years (DALYs) for 291 Diseases and Injuries in 21 Regions, 1990–2010: A Systematic Analysis for the Global Burden of Disease Study 2010. Lancet.

[3] Lai Y.S., Biedermann P., Ekpo U.F., Garba A., Mathieu E., Midzi N., Mwinzi P., N’Goran E.K., Raso G., Assaré R.K. (2015). Spatial Distribution of Schistosomiasis and Treatment Needs in Sub-Saharan Africa: A Systematic Review and Geostatistical Analysis. Lancet Infect. Dis..

[4] Cioli D., Pica-Mattoccia L., Basso A., Guidi A. (2014). Schistosomiasis Control: Praziquantel Forever?. Mol. Biochem. Parasitol..

[5] Da Paixão Siqueira L., Fontes D.A.F., Aguilera C.S.B., Timóteo T.R.R., Ângelos M.A., Silva L.C.P.B.B., de Melo C.G., Rolim L.A., da Silva R.M.F., Neto P.J.R. (2017). Schistosomiasis: Drugs Used and Treatment Strategies. Acta Trop..

[6] Vale N., Gouveia M.J., Rinaldi G., Brindley P.J., Gärtner F., da Costa J.M.C. (2017). Praziquantel for Schistosomiasis: Single-Drug Metabolism Revisited, Mode of Action, and Resistance. Antimicrob. Agents Chemother..

[7] Botros S., Sayed H., Amer N., El-Ghannam M., Bennett J.L., Day T.A. (2005). Current Status of Sensitivity to Praziquantel in a Focus of Potential Drug Resistance in Egypt. Int. J. Parasitol..

[8] Daniel P.B., Walker W.H., Habener J.F. (1998). Cyclic amp signaling and gene regulation. Annu. Rev. Nutr..

[9] Pinner N.A., Hamilton L.A., Hughes A. (2012). Roflumilast: A Phosphodiesterase-4 Inhibitor for the Treatment of Severe Chronic Obstructive Pulmonary Disease. Clin. Ther..

[10] Calverley P.M., Rabe K.F., Goehring U.M., Kristiansen S., Fabbri L.M., Martinez F.J. (2009). Roflumilast in Symptomatic Chronic Obstructive Pulmonary Disease: Two Randomised Clinical Trials. Lancet.

[11] Packer M., Carver J.R., Rodeheffer R.J., Ivanhoe R.J., DiBianco R., Zeldis S.M., Hendrix G.H., Bommer W.J., Elkayam U., Kukin M.L. (1991). Effect of Oral Milrinone on Mortality in Severe Chronic Heart Failure. N. Engl. J. Med..

[12] Boolell M., Allen M.J., Ballard S.A., Gepi-Attee S., Muirhead G.J., Naylor A.M., Osterloh I.H., Gingell C. (1996). Sildenafil: An Orally Active Type 5 Cyclic GMP-Specific Phosphodiesterase Inhibitor for the Treatment of Penile Erectile Dysfunction. Int. J. Impot. Res..

[13] Giembycz M.A., Field S.K. (2010). Roflumilast: First Phosphodiesterase 4 Inhibitor Approved for Treatment of COPD. Drug Des. Dev. Ther..

[14] Seebeck T., Sterk G.J., Ke H. (2011). Phosphodiesterase Inhibitors as a New Generation of Antiprotozoan Drugs: Exploiting the Benefit of Enzymes That Are Highly Conserved between Host and Parasite. Future Med. Chem..

[15] Oberholzer M., Marti G., Baresic M., Kunz S., Hemphill A., Seebeck T. (2007). The Trypanosoma Brucei CAMP Phosphodiesterases TbrPDEBl and TbrPDEB2: Flagellar Enzymes That Are Essential for Parasite Virulence. FASEB J..

[16] Orrling K.M., Jansen C., Vu X.L., Balmer V., Bregy P., Shanmugham A., England P., Bailey D., Cos P., Maes L. (2012). Catechol Pyrazolinones as Trypanocidals: Fragment-Based Design, Synthesis, and Pharmacological Evaluation of Nanomolar Inhibitors of Trypanosomal Phosphodiesterase B1. J. Med. Chem..

[17] Wang C., Ashton T.D., Gustafson A., Bland N.D., Ochiana S.O., Campbell R.K., Pollastri M.P. (2012). Synthesis and Evaluation of Human Phosphodiesterases (PDE) 5 Inhibitor Analogs as Trypanosomal PDE Inhibitors. Part 1. Sildenafil Analogs. Bioorg. Med. Chem. Lett..

[18] Blaazer A.R., Singh A.K., De Heuvel E., Edink E., Orrling K.M., Veerman J.J.N., Van Den Bergh T., Jansen C., Balasubramaniam E., Mooij W.J. (2018). Targeting a Subpocket in Trypanosoma Brucei Phosphodiesterase B1 (TbrPDEB1) Enables the Structure-Based Discovery of Selective Inhibitors with Trypanocidal Activity. J. Med. Chem..

[19] De Heuvel E., Singh A.K., Boronat P., Kooistra A.J., van der Meer T., Sadek P., Blaazer A.R., Shaner N.C., Bindels D.S., Caljon G. (2019). Alkynamide Phthalazinones as a New Class of TbrPDEB1 Inhibitors (Part 2). Bioorg. Med. Chem..

[20] De Heuvel E., Singh A.K., Edink E., van der Meer T., van der Woude M., Sadek P., Krell-Jørgensen M.P., van den Bergh T., Veerman J., Caljon G. (2019). Alkynamide Phthalazinones as a New Class of TbrPDEB1 Inhibitors. Bioorg. Med. Chem..

[21] Wang H., Yan Z., Geng J., Kunz S., Seebeck T., Ke H. (2007). Crystal Structure of the Leishmania Major Phosphodiesterase LmjPDEB1 and Insight into the Design of the Parasite-Selective Inhibitors. Mol. Microbiol..

[22] Howard B.L., Harvey K.L., Stewart R.J., Azevedo M.F., Crabb B.S., Jennings I.G., Sanders P.R., Manallack D.T., Thompson P.E., Tonkin C.J. (2015). Identification of Potent Phosphodiesterase Inhibitors That Demonstrate Cyclic Nucleotide-Dependent Functions in Apicomplexan Parasites. ACS Chem. Biol..

[23] Kunz S., Balmer V., Sterk G.J., Pollastri M.P., Leurs R., Müller N., Hemphill A., Spycher C. (2017). The Single Cyclic Nucleotide-Specific Phosphodiesterase of the Intestinal Parasite Giardia Lamblia Represents a Potential Drug Target. PLoS Negl. Trop. Dis..

[24] Taft A.S., Norante F.A., Yoshino T.P. (2010). The Identification of Inhibitors of *Schistosoma mansoni* Miracidial Transformation by Incorporating a Medium-Throughput Small-Molecule Screen. Exp. Parasitol..

[25] Final Report Summary—PDE4NPD. https://cordis.europa.eu/project/id/602666/reporting.

[26] Munday J.C., Kunz S., Kalejaiye T.D., Siderius M., Schroeder S., Paape D., Alghamdi A.H., Abbasi Z., Huang S.X., Donachie A.-M. (2020). Cloning and Functional Complementation of Ten *Schistosoma mansoni* Phosphodiesterases Expressed in the Mammalian Host Stages. PLoS Negl. Trop. Dis..

[27] Long T., Rojo-Arreola L., Shi D., El-Sakkary N., Jarnagin K., Rock F., Meewan M., Rascón A.A., Lin L., Cunningham K.A. (2017). Phenotypic, Chemical and Functional Characterization of Cyclic Nucleotide Phosphodiesterase 4 (PDE4) as a Potential Anthelmintic Drug Target. PLoS Negl. Trop. Dis..

[28] Pagès L., Gavaldà A., Lehner M.D. (2009). PDE4 Inhibitors: A Review of Current Developments (2005–2009). Expert Opin. Ther. Pat..

[29] Zebda R., Paller A.S. (2018). Phosphodiesterase 4 Inhibitors. J. Am. Acad. Dermatol..

[30] Sebastián-Pérez V., Schroeder S., Munday J.C., van der Meer T., Zaldívar-Díez J., Siderius M., de Koning H.P., Brown D., Martínez A., Campillo N.E. (2019). Discovery of Novel *Schistosoma mansoni* PDE4A Inhibitors as Potential Agents against Schistosomiasis. Future Med. Chem..

[31] Rose A.S., Bradley A.R., Valasatava Y., Duarte J.M., Prlić A., Rose P.W. (2018). NGL Viewer: Web-Based Molecular Graphics for Large Complexes. Bioinformatics.

[32] Xu R.X., Hassell A.M., Vanderwall D., Lambert M.H., Holmes W.D., Luther M.A., Rocque W.J., Milburn M.V., Zhao Y., Ke H. (2000). Atomic Structure of PDE4: Insights into Phosphodiesterase Mechanism and Specificity. Science.

[33] Wang H., Kunz S., Chen G., Seebeck T., Wan Y., Robinson H., Martinelli S., Ke H. (2012). Biological and Structural Characterization of Trypanosoma Cruzi Phosphodiesterase C and Implications for Design of Parasite Selective Inhibitors. J. Biol. Chem..

[34] Jansen C., Wang H., Kooistra A.J., De Graaf C., Orrling K.M., Tenor H., Seebeck T., Bailey D., De Esch I.J.P., Ke H. (2013). Discovery of Novel Trypanosoma Brucei Phosphodiesterase B1 Inhibitors by Virtual Screening against the Unliganded TbrPDEB1 Crystal Structure. J. Med. Chem..

[35] Jansen C., Kooistra A.J., Kanev G.K., Leurs R., De Esch I.J.P., De Graaf C. (2016). PDEStrIAn: A Phosphodiesterase Structure and Ligand Interaction Annotated Database As a Tool for Structure-Based Drug Design. J. Med. Chem..

[36] Trott O., Olson A.J. (2010). AutoDock Vina: Improving the Speed and Accuracy of Docking with a New Scoring Function, Efficient Optimization, and Multithreading. J. Comput. Chem..

[37] Card G.L., England B.P., Suzuki Y., Fong D., Powell B., Lee B., Luu C., Tabrizizad M., Gillette S., Ibrahim P.N. (2004). Structural Basis for the Activity of Drugs That Inhibit Phosphodiesterases. Structure.

[38] Botros S.S., El-Lakkany N.M., Seif el-Din S.H., William S., Sabra A.N., Hammam O.A., de Koning H.P. (2020). The Phosphodiesterase-4 Inhibitor Roflumilast Impacts *Schistosoma mansoni* Ovipositing in Vitro but Displays Only Modest Antischistosomal Activity In Vivo. Exp. Parasitol..

[39] Freitag A., Wessier I., Racké K. (1998). Phosphodiesterase Inhibitors Suppress A2-Adrenoceptor-Mediated 5-Hydroxytryptamine Release from Tracheae of Newborn Rabbits. Eur. J. Pharmacol..

[40] Francis S.H., Sekhar K.R., Ke H., Corbin J.D., Fredholm B.B. (2011). Inhibition of Cyclic Nucleotide Phosphodiesterases by Methylxanthines and Related Compounds. Handbook of Experimental Pharmacology.

[41] Thompson W.J. (1991). Cyclic Nucleotide Phosphodiesterases: Pharmacology, Biochemistry and Function. Pharmacol. Ther..

[42] Menniti F.S., Faraci W.S., Schmidt C.J. (2006). Phosphodiesterases in the CNS: Targets for Drug Development. Nat. Rev. Drug Discov..

[43] Bell A.S., Terrett N.K. (1999). Pyrazolo[4,3-d]Pyrimidine Derivatives and Pharmaceutical Compositions Containing Them. Patent.

[44] Maurice D.H., Ke H., Ahmad F., Wang Y., Chung J., Manganiello V.C. (2014). Advances in Targeting Cyclic Nucleotide Phosphodiesterases. Nat. Rev. Drug Discov..

[45] Ruppert D., Weithmann K.U. (1982). HL 725, an Extremely Potent Inhibitor of Platelet Phosphodiesterase and Induced Platelet Aggregation in Vitro. Life Sci..

[46] Sircar I., Steffen R.P., Bobowski G., Burke S.E., Newton R.S., Weishaar R.E., Bristol J.A., Evans D.B. (1989). Cardiotonic Agents. 9. Synthesis and Biological Evaluation of a Series of (E)-4,5-Dihydro-6-[2-[4-(1H-Imidazol-1-Yl)Phenyl]Ethenyl]-3(2H)-Pyridazinones: A Novel Class of Compounds with Positive Inotropic, Antithrombotic, and Vasodilatory Activities for Th. J. Med. Chem..

[47] Sudo T., Tachibana K., Toga K., Tochizawa S., Inoue Y., Kimura Y., Hidaka H. (2000). Potent Effects of Novel Anti-Platelet Aggregatory Cilostamide Analogues on Recombinant Cyclic Nucleotide Phosphodiesterase Isozyme Activity. Biochem. Pharmacol..

[48] Mata M., Pallardo F., Morcillo E.J., Cortijo J. (2012). Piclamilast Inhibits the Pro-Apoptotic and Anti-Proliferative Responses of A549 Cells Exposed to H2O2 via Mechanisms Involving AP-1 Activation. Free Radic. Res..

[49] Sterk G.J., Hatzelmann A., Barsig J., Marx D., Kley H.-P., Christiaans J.A.M., Menge W.M.P.B. (2005). Pyrrolidinedione Substituted Piperidine-Phthalazones as Pde4. Inhibitors. Patent.

[50] Vellas B., Sol O., Snyder P.J., Ousset P.-J., Haddad R., Maurin M., Lemarie J.-C., Desire L., Pando M.P. (2011). EHT0202 in Alzheimers Disease: A 3-Month, Randomized, Placebo- Controlled, Double-Blind Study. Curr. Alzheimer Res..

[51] Sterk G.J., Hatzelmann A., Marx D., Steinhilber W. (2004). Phthalazinones Derivatives Useful as Pde4/7. Inhibitors. Patent.

[52] De Koning H.P., Gould M.K., Sterk G.J., Tenor H., Kunz S., Luginbuehl E., Seebeck T. (2012). Pharmacological Validation of Trypanosoma Brucei Phosphodiesterases as Novel Drug Targets. J. Infect. Dis..

[53] Weinbrenner S., Dunkern T., Marx D., Schmidt B., Stenget T., Flockerzi D., Kautz U., Hauser D., Diefenbach J., Christiaans J.A.M. (2008). 6-Benzyl-2,3,4,7-Tetrahydro-Indolo[2,3-c]Quinoline Compounds Useful as Pde5. Inhibitors. Patent.

[54] Haning H., Niewöhner U., Schenke T., Lampe T., Hillisch A., Bischoff E. (2005). Comparison of Different Heterocyclic Scaffolds as Substrate Analog PDE5 Inhibitors. Bioorg. Med. Chem. Lett..

[55] Choi S.-H., Choi D.-H., Song K.-S., Shin K.-H., Chun B.-G. (2002). Zaprinast, an Inhibitor of CGMP-Selective Phosphodiesterases, Enhances the Secretion of TNF-α and IL-1β and the Expression of INOS and MHC Class II Molecules in Rat Microglial Cells. J. Neurosci. Res..

[56] Smith S.J., Cieslinski L.B., Newton R., Donnelly L.E., Fenwick P.S., Nicholson A.G., Barnes P.J., Barnette M.S., Giembycz M.A. (2004). Discovery of BRL 50481 [3-(&lt;Em&gt;N,N&lt;/Em&gt;-Dimethylsulfonamido)-4-Methyl-Nitrobenzene], a Selective Inhibitor of Phosphodiesterase 7: In Vitro Studies in Human Monocytes, Lung Macrophages, and CD8&lt;Sup&gt;+&lt;/Sup&gt; T-Lymphocytes. Mol. Pharmacol..

[57] Redondo M., Brea J., Perez D.I., Soteras I., Val C., Perez C., Morales-García J.A., Alonso-Gil S., Paul-Fernandez N., Martin-Alvarez R. (2012). Effect of Phosphodiesterase 7 (PDE7) Inhibitors in Experimental Autoimmune Encephalomyelitis Mice. Discovery of a New Chemically Diverse Family of Compounds. J. Med. Chem..

[58] García A.M., Brea J., Morales-García J.A., Perez D.I., González A., Alonso-Gil S., Gracia-Rubio I., Ros-Simó C., Conde S., Cadavid M.I. (2014). Modulation of CAMP-Specific PDE without Emetogenic Activity: New Sulfide-Like PDE7 Inhibitors. J. Med. Chem..

[59] DeNinno M.P., Wright S.W., Visser M.S., Etienne J.B., Moore D.E., Olson T.V., Rocke B.N., Andrews M.P., Zarbo C., Millham M.L. (2011). 1,5-Substituted Nipecotic Amides: Selective PDE8 Inhibitors Displaying Diastereomer-Dependent Microsomal Stability. Bioorg. Med. Chem. Lett..

[60] Malamas M.S., Ni Y., Erdei J., Stange H., Schindler R., Lankau H.-J., Grunwald C., Fan K.Y., Parris K., Langen B. (2011). Highly Potent, Selective, and Orally Active Phosphodiesterase 10A Inhibitors. J. Med. Chem..

[61] Ceyhan O., Birsoy K., Hoffman C.S. (2012). Identification of Biologically Active PDE11-Selective Inhibitors Using a Yeast-Based High-Throughput Screen. Chem. Biol..

[62] Integrated DNA Technologies Tool. https://www.idtdna.com/CodonOpt.

[63] Berrow N.S., Alderton D., Sainsbury S., Nettleship J., Assenberg R., Rahman N., Stuart D.I., Owens R.J. (2007). A Versatile Ligation-Independent Cloning Method Suitable for High-Throughput Expression Screening Applications. Nucleic Acids. Res..

[64] Winn M.D., Ballard C.C., Cowtan K.D., Dodson E.J., Emsley P., Evans P.R., Keegan R.M., Krissinel E.B., Leslie A.G.W., McCoy A. (2011). Overview of the CCP4 Suite and Current Developments. Acta Crystallogr. Sect. D.

[65] Emsley P., Cowtan K. (2004). Coot: Model-Building Tools for Molecular Graphics. Acta Crystallogr. Sect. D.

[66] Duvall R.H., DsWitt W.B. (1967). An Improved Perfusion Technique for Recovering Adult Schistosomes from Laboratory Animals. Am. J. Trop. Med. Hyg..

[67] Pica-Mattoccia L., Cioli D. (2004). Sex- and Stage-Related Sensitivity of *Schistosoma mansoni* to In Vivo and In Vitro Praziquantel Treatment. Int. J. Parasitol..

[68] Botros S., Pica-Mattoccia L., William S., El-Lakkani N., Cioli D. (2005). Effect of Praziquantel on the Immature Stages of *Schistosoma haematobium*. Int. J. Parasitol..

[69] Ashton M.J., Cook D.C., Fenton G., Karlsson J.A., Palfreyman M.N., Raeburn D., Ratcliffe A.J., Souness J.E., Thurairatnam S., Vicker N. (1994). Selective Type IV Phosphodiesterase Inhibitors as Antiasthmatic Agents. The Syntheses and Biological Activities of 3-(Cyclopentyloxy)-4-Methoxybenzamides and Analogues. J. Med. Chem..

[70] Lin Y., Huang P., Liu S., Sima L., Chen L., Wang D. (2013). A Convenient Method for the Synthesis of Roflumilast. Res Chem. Intermed..

[71] Felding J., Sørensen M.D., Poulsen T.D., Larsen J., Andersson C., Refer P., Engell K., Ladefoged L.G., Thormann T., Vinggaard A.M. (2014). Discovery and Early Clinical Development of 2-{6-[2-(3,5-Dichloro-4- Pyridyl)Acetyl]-2,3-Dimethoxyphenoxy}- N -Propylacetamide (LEO 29102), a Soft-Drug Inhibitor of Phosphodiesterase 4 for Topical Treatment of Atopic Dermatitis. J. Med. Chem..

[72] Bland N.D., Wang C., Tallman C., Gustafson A.E., Wang Z., Ashton T.D., Ochiana S.O., McAllister G., Cotter K., Fang A.P. (2011). Pharmacological Validation of Trypanosoma Brucei Phosphodiesterases B1 and B2 as Druggable Targets for African Sleeping Sickness. J. Med. Chem..

[73] Yao L., Xiao Z. (2016). Preparation Method of Roflumilast N-Oxide. C.N. Patent.

[74] Zhou Z.Z., Ge B.C., Zhong Q.P., Huang C., Cheng Y.F., Yang X.M., Wang H.T., Xu J.P. (2016). Development of Highly Potent Phosphodiesterase 4 Inhibitors with Anti-Neuroinflammation Potential: Design, Synthesis, and Structure-Activity Relationship Study of Catecholamides Bearing Aromatic Rings. Eur. J. Med. Chem..

