# Supplementary material for: To Target or Not to Target Schistosoma mansoni Cyclic Nucleotide Phosphodiesterase 4A?
Source: Int J Mol Sci. 2023 Apr 6;24(7):6817. doi: 10.3390/ijms24076817 (PMC10095301; doi:10.3390/ijms24076817)
Supplement: Supplementary file 1 [file ijms-24-06817-s001.zip › ijms-2236490-supplementary.pdf]

Supplementary data

To target or not to target *Schistosoma mansoni* cyclic nucleotide phosphodiesterase 4A?

Yang Zheng[1], Susanne Schroeder[2], Georgi K. Kanev[1], Sanaa S. Botros[3], Samia William[4], Abdel-Nasser A. Sabra[3], Louis Maes[5], Guy Caljon[5], Carmen Gil[6], Ana Martinez[6], Irene G. Salado[7], Koen Augustyns[7], Ewald Edink[1], Maarten Sijm[1], Erik de Heuvel[1], Iwan J. P. de Esch[1], Tiffany van der Meer[1], Marco Siderius[1], Geert Jan Sterk[1], David Brown[2] and Rob Leurs[1]*

[1] Division of Medicinal Chemistry, Amsterdam Institute of Molecular and Life Sciences, Vrije Universiteit Amsterdam, 1081 HZ Amsterdam, the Netherlands.

[2] School of Biosciences, University of Kent, Canterbury CT2 7NJ, UK.

[3] Pharmacology Department, Theodor Bilharz Research Institute, Warrak El-Hadar, Imbaba, P.O. Box 30, Giza, 12411, Egypt.

[4] Parasitology Department, Theodor Bilharz Research Institute, Warrak El-Hadar, Imbaba, P.O. Box 30, Giza, 12411, Egypt.

[5] Laboratory of Microbiology, Parasitology and Hygiene (LMPH), University of Antwerp, Universiteitsplein 1, 2610 Wilrijk, Belgium.

[6] Centro de Investigaciones Biologicas (CIB-CSIC), Ramiro de Maeztu 9, 28040 Madrid, Spain.

[7] Medicinal Chemistry, Faculty of Pharmaceutical, Biomedical and Veterinary Sciences, University of Antwerp, Universiteitsplein 1, 2610 Antwerp, Belgium.

* Corresponding author: r.leurs@vu.nl (RL)

Table of contents

**Experimental section**	**P3-29**
**Table S1-2**	**P30-32**
**Figure S1-2**	**P33-34**
**Crystallographic data**	**P35**
**Figure S3-S257**	**P36-206**
**References**	**P207-209**

Table S1. Overview of the PDE4NPD toolbox and inhibitor selectivity for human (h) or parasite PDEs.

Table S2. SmPDE4A inhibition of roflumilast analogues.

Fig S1. Correlation plot of the inhibition of the PDE Toolbox compounds of the enzymatic activities of purified SmPDE4A_CD (catalytic domain) and SmPDE4A_FL (full length).

Fig S2. Binding of the substrate cAMP (A) and its hydrolyzed product AMP (B) in the binding pockets of SmPDE4A_CD, as observed in the chain A and chain B of the X-ray structure 6FG5.

Fig S3-S257. Chemical characterization of final compounds.

Experimental section

All starting materials were obtained from commercial suppliers and used without purification. Anhydrous THF and DMF were obtained by passing through an activated alumina column prior to use. TLC analyses were performed using Merck F_{254} aluminum-backed silica plates and visualized with 254 nm UV light. Flash column chromatography was executed using Biotage Isolera equipment. All HRMS spectra were recorded on a Bruker microTOF mass spectrometer using ESI in positive-ion mode. All NMR spectra were recorded on either a Bruker Avance 250, Bruker Avance 300, Bruker Avance 500, or Bruker Avance 600 spectrometer. The peak multiplicities are defined as follows: s, singlet; d, doublet; t, triplet; q, quartet; p, pentet; dd, doublet of doublets; dt, doublet of triplets; td, triplet of doublets; br, broad; m, multiplet; app, apparent. The spectra were referenced to the internal solvent peak as follows: $CDCl_3$ (δ = 7.26 ppm in ^{1}H NMR, δ = 77.16 ppm in ^{13}C NMR), DMSO-d_6 (δ = 2.50 ppm in ^{1}H NMR, δ = 39.52 ppm in ^{13}C NMR). IUPAC names were adapted from ChemBioDraw Ultra 19.0. Purities were measured with the aid of analytical LC-MS using a Shimadzu LC-20AD liquid chromatography pump system with a Shimadzu SPDM20A diode array detector with the MS detection performed with a Shimadzu LCMS-2010EV mass spectrometer operating in positive ionization mode. The column used was an Xbridge (C18) 5 µm column (100 mm × 4.6 mm). The following solutions are used for the eluents. Solvent A: H_2O/HCOOH 999:1, and solvent B: MeCN/HCOOH 999:1. The eluent program used is as follows: flow rate: 1.0 mL/min, start with 95% A in a linear gradient to 10% A over 4.5 min, hold 1.5 min at 10% A, in 0.5 min in a linear gradient to 95% A, hold 1.5 min at 95% A, total run time: 8.0 min. Compound purities were calculated as the percentage peak area of the analyzed compound by UV detection at 254 nm. Given that many compounds were made by undergraduate students and re-purification was usually necessary, yields are not representative and are therefore omitted from the procedures.

General procedure for all compounds: under an N_2 atmosphere in a dry flask, oxalyl chloride (4.8 mmol, 1.2 eq.) was added dropwise to a mixture of carboxylic acid A (4.0 mmol, 1.0 eq.) and DMF (100 µL) in THF (20 mL) at 0°C. The mixture was allowed to reach room temperature and stirred for an additional 30 min to obtain the corresponding acyl chloride. In another dry flask with N_2 insert, NaH (60% dispersion in mineral oil, 10.0 mmol, 2.5 eq.) was added in small portions to amine/aniline B (10.0 mmol, 2.5 eq.) in DMF (20 mL) at 0°C. The suspension was allowed to reach room temperature and stirred for an additional 30 min. To this mixture, the crude acyl chloride solution was added dropwise at 0°C. The reaction mixture was allowed to reach room temperature and stirred for an additional 30 min. After quenching with a saturated NH_4Cl solution (150 mL), the aqueous suspension was extracted with EtOAc for three times. The combined organic layers were washed with brine, dried over Na_2SO_4 and concentrated *in vacuo*. The crude product was purified using flash column chromatography. All compounds were analyzed using LCMS, ^{1}H NMR, ^{13}C NMR and HRMS and had purity >95% (area% on LC at 254 nm).

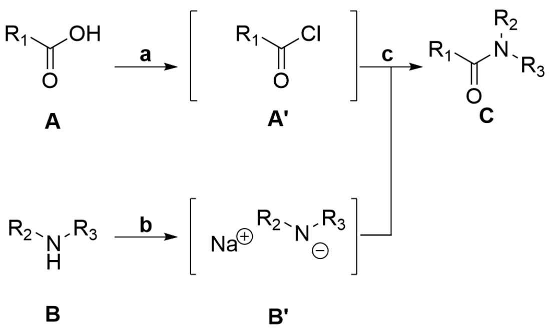

Scheme 1. General synthetic route of the roflumilast analogue library. Reagents and conditions: a: acid A, THF, $(COCl)_2$, *cat*. DMF, 0°C to RT, 30 min; b: amine/aniline B, NaH, DMF, 0°C to RT, 30 min; c: A', B', 0°C to RT, 30 min; d: C, *m*-CPBA, DCM, RT, 30 min.

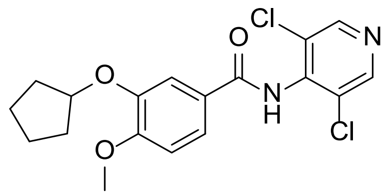

3-(Cyclopentyloxy)-*N*-(3,5-dichloropyridin-4-yl)-4-methoxybenzamide (**NPD-0005**).

^{1}H NMR (500 MHz, $CDCl_3$) δ 8.56 (s, 2H), 7.69 (s, 1H), 7.52 - 7.48 (m, 2H), 6.94 (d, *J* = 8.3 Hz, 1H), 4.87 (tt, *J* = 6.3, 3.2 Hz, 1H), 3.93 (s, 3H), 2.04 - 1.80 (m, 6H), 1.67 - 1.59 (m, 2H). ^{13}C NMR (126 MHz, $CDCl_3$) δ 164.3, 154.2, 148.2, 148.0, 140.7, 128.8, 125.0, 120.7, 114.3, 111.0, 80.9, 56.3, 32.9, 24.2. LC-MS: t_R= 4.52 min, purity: >99%, M/z $[M+H]^+$: 381; HRMS calcd. for $C_{18}H_{18}Cl_2N_2O_3$ $[M+H]^+$ = 381.0767, found 381.0784. Spectral data are in agreement with a previous report. [1]

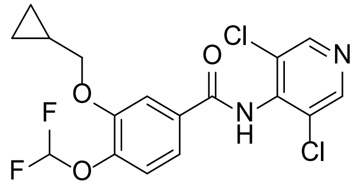

3-(Cyclopropylmethoxy)-*N*-(3,5-dichloropyridin-4-yl)-4-(difluoromethoxy)benzamide (**NPD-0006**)

^{1}H NMR (600 MHz, $CDCl_3$) δ 8.58 (s, 2H), 7.70 (s, 1H), 7.59 (s, 1H), 7.47 (d, *J* = 8.2 Hz, 1H), 7.29 (d, *J* = 8.2 Hz, 1H), 6.75 (t, *J* = 74.8 Hz, 1H), 3.97 (d, *J* = 6.9 Hz, 2H), 1.36 - 1.28 (m, 1H), 0.70 - 0.66 (m, 2H), 0.40 - 0.36 (m, 2H). ^{13}C NMR (151 MHz, $CDCl_3$) δ 163.8, 151.1, 148.4, 144.0, 139.9, 130.9, 129.0, 122.5, 120.0, 117.5, 115.8 (t, *J* = 256.7 Hz), 74.4, 10.2, 3.5. LC-MS: t_R= 4.65 min, purity: 99%, M/z $[M+H]^+$: 403; HRMS calcd. for $C_{17}H_{14}Cl_2F_2N_2O_3$ $[M+H]^+$ = 403.0422, found 403.0417. Spectral data are in agreement with a previous report. [2]

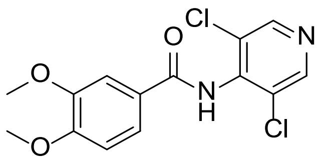

***N*-(3,5-Dichloropyridin-4-yl)-3,4-dimethoxybenzamide** (**NPD-0446**)

^{1}H NMR (500 MHz, DMSO-d_6) δ 10.48 (s, 1H), 8.77 (s, 2H), 7.70 (dd, *J* = 8.4, 2.1 Hz, 1H), 7.59 (d, *J* = 2.1 Hz, 1H), 7.14 (d, *J* = 8.5 Hz, 1H), 3.87 (s, 3H), 3.85 (s, 3H). ^{13}C NMR (126 MHz, DMSO-d_6) δ 164.3, 152.3, 148.5, 148.3, 141.6, 130.8, 124.7, 121.6, 111.1, 111.0, 55.8, 55.6. LC-MS: t_R= 3.51 min, purity: >99%, M/z [M+H]$^+$: 327; HRMS calcd. for $C_{14}H_{12}Cl_2N_2O_3$ [M+H]$^+$ = 327.0298, found 327.0309. Spectral data are in agreement with a previous report. [3]

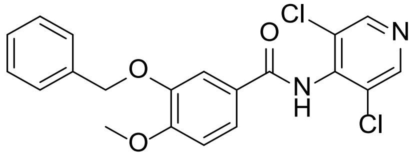

3-(Benzyloxy)-*N*-(3,5-dichloropyridin-4-yl)-4-methoxybenzamide (**NPD-0546**)

^{1}H NMR (500 MHz, DMSO-d_6 + 1 drop of $CDCl_3$) δ 10.35 (s, 1H), 8.61 (s, 2H), 7.71 - 7.66 (m, 2H), 7.47 - 7.43 (m, 2H), 7.38 - 7.33 (m, 2H), 7.32 - 7.27 (m, 1H), 7.07 - 7.02 (m, 1H), 5.13 (s, 2H), 3.86 (s, 3H). ^{13}C NMR (126 MHz, DMSO-d_6 + 1 drop of $CDCl_3$) δ 164.2, 152.5, 147.9, 147.3, 141.4, 136.5, 130.7, 128.2, 127.75, 127.68, 124.7, 122.0, 112.9, 110.8, 70.1, 55.6. LC-MS: t_R= 4.52 min, purity: >99%, M/z [M+H]$^+$: 403; HRMS calcd. for $C_{20}H_{16}Cl_2N_2O_3$ [M+H]$^+$ = 403.0611, found 403.0609. Spectral data are in agreement with a previous report. [4]

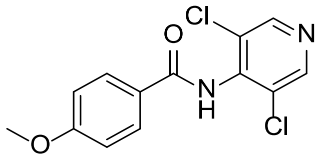

***N*-(3,5-Dichloropyridin-4-yl)-4-methoxybenzamide** (**NPD-0548**)

^{1}H NMR (500 MHz, DMSO-d_6) δ 10.49 (s, 1H), 8.76 (s, 2H), 8.02 (d, *J* = 8.9 Hz, 2H), 7.12 (d, *J* = 8.9 Hz, 2H), 3.87 (s, 3H). ^{13}C NMR (126 MHz, DMSO-d_6) δ 164.3, 162.5, 148.3, 141.6, 130.9, 130.0, 124.8, 113.9, 55.6. LC-MS: t_R= 3.73 min, purity: >99%, M/z [M+H]$^+$: 297; HRMS calcd. for $C_{13}H_{10}Cl_2N_2O_2$ [M+H]$^+$ = 297.0192, found 297.0201.

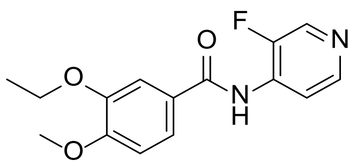

3-Ethoxy-*N*-(3-fluoropyridin-4-yl)-4-methoxybenzamide (**NPD-1177**)

^{1}H NMR (250 MHz, DMSO-d_6) δ 10.28 (s, 1H), 8.58 (d, *J* = 2.8 Hz, 1H), 8.38 (d, *J* = 5.3 Hz, 1H), 7.87 (dd, *J* = 6.7, 5.3 Hz, 1H), 7.63 (dd, *J* = 8.4, 2.1 Hz, 1H), 7.54 (d, *J* = 2.1 Hz, 1H), 7.10 (d, *J* = 8.5 Hz, 1H), 4.09 (q, *J* = 7.0 Hz, 2H), 3.85 (s, 3H), 1.36 (t, *J* = 7.0 Hz, 3H). ^{13}C NMR (126 MHz, DMSO-d_6) δ 165.2, 152.4, 151.4 (d, *J* = 255.1 Hz), 147.6, 146.3 (d, *J* = 5.0 Hz), 138.2 (d, *J* = 21.8 Hz), 133.7 (d, *J* = 9.5 Hz), 125.4, 121.8, 118.7 (app s), 112.1, 111.0, 63.9, 55.7, 14.7. LC-MS: t_R= 3.26 min, purity: >99%, M/z [M+H]$^+$: 291; HRMS calcd. for $C_{15}H_{15}FN_2O_3$ [M+H]$^+$ = 291.1139, found 291.1150.

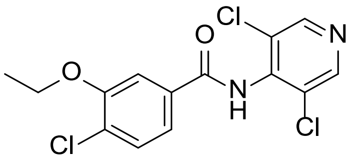

4-Chloro-*N*-(3,5-dichloropyridin-4-yl)-3-ethoxybenzamide (**NPD-1178**)

^{1}H NMR (250 MHz, DMSO-d_6) δ 10.73 (s, 1H), 8.77 (s, 2H), 7.71 - 7.65 (m, 1H), 7.66 - 7.61 (m, 2H), 4.22 (q, *J* = 7.0 Hz, 2H), 1.40 (t, *J* = 7.0 Hz, 3H). ^{13}C NMR (126 MHz, DMSO-d_6) δ 164.3, 154.4, 148.8, 141.6, 133.0, 131.1, 130.6, 126.1, 121.3, 113.1, 65.1, 15.0. LC-MS: t_R= 4.60 min, purity: 99%, M/z [M+H]$^+$: 345; HRMS calcd. For $C_{14}H_{11}Cl_3N_2O_2$ [M+H]$^+$ = 344.9959, found 344.9958.

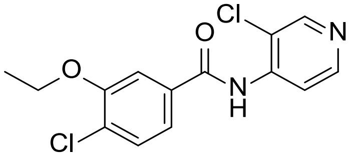

4-Chloro-*N*-(3-chloropyridin-4-yl)-3-ethoxybenzamide (**NPD-1179)**

^{1}H NMR (250 MHz, DMSO-d_6) δ 10.25 (s, 1H), 8.70 (s, 1H), 8.52 (d, *J* = 5.3 Hz, 1H), 7.83 (d, *J* = 5.3 Hz, 1H), 7.67 (d, *J* = 1.7 Hz, 1H), 7.63 (d, *J* = 8.2 Hz, 1H), 7.57 (dd, *J* = 8.2, 1.8 Hz, 1H), 4.22 (q, *J* = 7.0 Hz, 2H), 1.40 (t, *J* = 7.0 Hz, 3H). ^{13}C NMR (126 MHz, DMSO-d_6) δ 165.1, 154.3, 150.2, 149.1, 142.8, 133.9, 130.5, 126.0, 125.2, 121.4, 120.7, 113.2, 65.1, 15.0. LC-MS: t_R= 4.64 min, purity: >99%, M/z [M+H]$^+$: 311; HRMS calcd. For $C_{14}H_{12}Cl_2N_2O_2$ [M+H]$^+$ = 311.0349, found 311.0351.

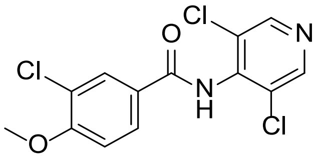

3-Chloro-*N*-(3,5-dichloropyridin-4-yl)-4-methoxybenzamide (**NPD-1180**)

^{1}H NMR (250 MHz, DMSO-d_6) δ 10.63 (s, 1H), 8.75 (s, 2H), 8.11 (s, 1H), 8.01 (d, *J* = 8.7 Hz, 1H), 7.34 (d, *J* = 8.8 Hz, 1H), 3.96 (s, 3H). ^{13}C NMR (126 MHz, DMSO-d_6) δ 163.2, 157.8, 148.4, 141.2, 130.7, 129.5, 128.9, 125.5, 121.2, 112.7, 56.7. LC-MS: t_R= 4.18 min, purity: 98%, M/z [M+H]$^+$: 331; HRMS calcd. For $C_{13}H_9Cl_3N_2O_2$ [M+H]$^+$ = 330.9802, found 330.9810.

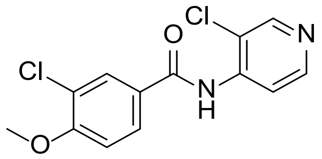

3-Chloro-*N*-(3-chloropyridin-4-yl)-4-methoxybenzamide (**NPD-1181**)

^{1}H NMR (250 MHz, DMSO-d_6) δ 10.17 (s, 1H), 8.68 (s, 1H), 8.50 (d, *J* = 5.4 Hz, 1H), 8.09 (d, *J* = 2.2 Hz, 1H), 8.00 (dd, *J* = 8.6, 2.3 Hz, 1H), 7.84 (dd, *J* = 5.6, 0.5 Hz, 1H), 7.33 (d, *J* = 8.7 Hz, 1H), 3.97 (s, 3H). ^{13}C NMR (126 MHz, DMSO-d_6) δ 164.3, 158.1, 150.2, 149.0, 142.9, 130.1, 129.4, 126.7, 125.1, 121.5, 120.5, 113.0, 57.1. LC-MS: t_R= 4.13 min, purity: >99%, M/z [M+H]$^+$: 297; HRMS calcd. For $C_{13}H_{10}Cl_2N_2O_2$ [M+H]$^+$= 297.0192, found 297.0197.

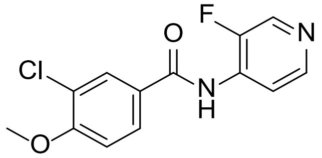

3-Chloro-*N*-(3-fluoropyridin-4-yl)-4-methoxybenzamide (**NPD-1182**)

^{1}H NMR (500 MHz, $CDCl_3$) δ 10.45 (s, 1H), 8.59 (d, *J* = 2.9 Hz, 1H), 8.38 (d, *J* = 5.3 Hz, 1H), 8.09 (d, *J* = 2.2 Hz, 1H), 7.98 (dd, *J* = 8.7, 2.3 Hz, 1H), 7.88 (dd, *J* = 6.7, 5.3 Hz, 1H), 7.31 (d, *J* = 8.8 Hz, 1H), 3.96 (s, 3H). ^{13}C NMR (126 MHz, DMSO-d_6) δ 164.2, 157.6, 151.3 (d, *J* = 255.7 Hz), 146.3 (d, *J* = 5.1 Hz), 138.2 (d, *J* = 21.8 Hz), 133.4 (d, *J* = 9.2 Hz), 129.8, 129.1, 126.3, 120.9, 118.6 (app s), 112.5, 56.6. LC-MS: t_R= 3.53 min, purity: >99%, M/z [M+H]$^+$: 281; HRMS calcd. For $C_{13}H_{10}ClFN_2O_2$ [M+H]$^+$= 281.0488, found 281.0490.

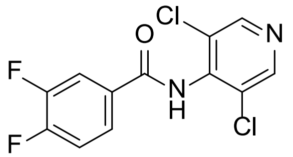

***N*-(3,5-Dichloropyridin-4-yl)-3,4-difluorobenzamide** (**NPD-1183**)

^{1}H NMR (250 MHz, DMSO-d_6) δ 10.82 (s, 1H), 8.79 (s, 2H), 8.11-8.00 (m, 1H), 7.96-7.89 (m, 1H), 7.75-7.62 (m, 1H). ^{13}C NMR (126 MHz, DMSO-d_6) δ 162.8 (d, *J* = 2.0 Hz), 152.2 (dd, *J* = 252.3, 12.6 Hz), 149.7 (dd, *J* = 249.8, 12.7 Hz), 148.4, 140.9, 130.7, 130.0 (dd, *J* = 4.7, 3.4 Hz), 125.7 (dd, *J* = 7.7, 3.5 Hz), 118.2 (d, *J* = 17.7 Hz), 117.4 (d, *J* = 18.6 Hz). LC-MS: t_R= 4.15 min, purity: >99%, M/z [M+H]$^+$: 303; HRMS calcd. For $C_{12}H_6Cl_2F_2N_2O$ [M+H]$^+$= 302.9898, found 302.9908.

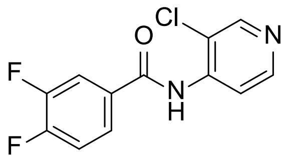

***N*-(3-Chloropyridin-4-yl)-3,4-difluorobenzamide** (**NPD-1184**)

^{1}H NMR (250 MHz, DMSO-d_6) δ 10.33 (s, 1H), 8.70 (d, *J* = 0.5 Hz, 1H), 8.52 (d, *J* = 5.3 Hz, 1H), 8.11-8.00 (m, 1H), 7.93-7.85 (m, 1H), 7.83 (dd, *J* = 5.4, 0.5 Hz, 1H), 7.75-7.58 (m, 1H). ^{13}C NMR (126 MHz, DMSO-d_6) δ 163.6 (d, *J* = 2.0 Hz), 152.0 (dd, *J* = 252.0, 12.7 Hz), 149.8, 149.2 (dd, *J* = 247.2, 13.1 Hz), 148.7, 142.3, 130.9 (dd, *J* = 5.3, 3.4 Hz), 125.8 (dd, *J* = 7.7, 3.5 Hz), 124.8, 120.2, 118.0 (d, *J* = 17.8 Hz), 117.6 (d, *J* = 18.6 Hz). LC-MS: t_R= 4.01 min, purity: >99%, M/z $[M+H]^+$: 269; HRMS calcd. For $C_{12}H_7ClF_2N_2O$ $[M+H]^+$= 269.0288, found 269.0299.

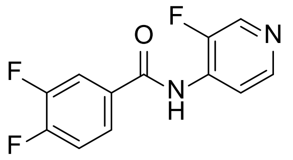

3,4-Difluoro-*N*-(3-fluoropyridin-4-yl)benzamide (**NPD-1185**)

^{1}H NMR (250 MHz, $CDCl_3$) δ 8.49 (d, *J* = 2.3 Hz, 1H), 8.45 (d, *J* = 6.2 Hz, 1H), 8.41 (d, *J* = 5.4 Hz, 1H), 8.16 (s, 1H), 7.84 - 7.71 (m, 1H), 7.71 - 7.59 (m, 1H), 7.40 - 7.27 (m, 1H). ^{13}C NMR (126 MHz, DMSO-d_6) δ 163.9 (d, *J* = 2.1 Hz), 151.2 (d, *J* = 255.6 Hz), 152.0 (dd, *J* = 251.9, 12.6 Hz), 149.1 (dd, *J* = 246.9, 13.0 Hz), 146.4 (d, *J* = 5.3 Hz), 138.3 (d, *J* = 21.6 Hz), 133.1 (d, *J* = 9.4 Hz), 130.9 (dd, *J* = 5.3, 3.4 Hz), 125.9 (dd, *J* = 7.6, 3.4 Hz), 118.5 (app s), 117.8 (d, *J* = 17.7 Hz), 117.8 (d, *J* = 18.6 Hz). LC-MS: t_R= 3.50 min, purity: >99%, M/z $[M+H]^+$: 253; HRMS calcd. For $C_{12}H_7F_3N_2O$ $[M+H]^+$= 253.0583, found 253.0593.

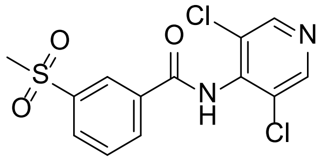

***N*-(3,5-Dichloropyridin-4-yl)-3-(methylsulfonyl)benzamide** (**NPD-1186**)

^{1}H NMR (500 MHz, DMSO-d_6 + 1 drop of $CDCl_3$) δ 10.96 (s, 1H), 8.69 (s, 2H), 8.57 (d, *J* = 8.7 Hz, 1H), 8.33 (d, *J* = 7.8 Hz, 1H), 8.18 (d, *J* = 7.9 Hz, 1H), 7.83 (t, *J* = 7.8 Hz, 1H), 3.26 (s, 3H). ^{13}C NMR (126 MHz, DMSO-d_6 + 1 drop of $CDCl_3$) δ 163.8, 148.6, 148.2, 141.7, 134.2, 133.4, 131.1, 131.0, 130.4, 126.9, 44.0. LC-MS: t_R= 3.81 min, purity: >99%, M/z $[M+H]^+$: 345; HRMS calcd. For $C_{13}H_{10}Cl_2N_2O_3S$ $[M+H]^+$= 344.9862, found 344.9852.

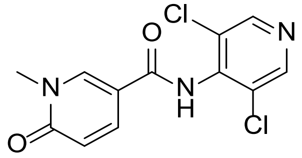

***N*-(3,5-Dichloropyridin-4-yl)-1-methyl-6-oxo-1,6-dihydropyridine-3-carboxamide** (**NPD-1187**)

^{1}H NMR (250 MHz, DMSO-d_6) δ 10.41 (s, 1H), 8.74 (s, 2H), 8.57 (d, *J* = 2.6 Hz, 1H), 7.98 (dd, *J* = 9.5, 2.7 Hz, 1H), 6.50 (d, *J* = 9.5 Hz, 1H), 3.53 (s, 3H). ^{13}C NMR (126 MHz, DMSO-d_6) δ 162.6, 162.3, 148.8, 144.3, 141.6, 138.2, 130.9, 118.6,

111.1, 38.0. LC-MS: t_R= 2.69 min, purity: >99%, M/z $[M+H]^+$: 298; HRMS calcd. For $C_{12}H_9Cl_2N_3O_2$ $[M+H]^+$= 298.0145, found 298.0152.

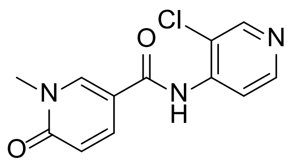

***N*-(3-Chloropyridin-4-yl)-1-methyl-6-oxo-1,6-dihydropyridine-3-carboxamide acetate** (**NPD-1188**)

^{1}H NMR (250 MHz, DMSO-d_6) δ 8.66 (s, 1H), 8.60 (d, *J* = 2.6 Hz, 1H), 8.48 (d, *J* = 5.4 Hz, 1H), 7.94 (dd, *J* = 9.5, 2.7 Hz, 1H), 7.82 (d, *J* = 5.4 Hz, 1H), 6.48 (d, *J* = 9.6 Hz, 1H), 1.89 (s, 3H). ^{13}C NMR (126 MHz, DMSO-d_6) δ 172.3, 163.0, 161.8, 149.7, 148.6, 143.9, 142.5, 138.1, 123.9, 119.4, 118.0, 111.4, 37.5. LC-MS: t_R= 2.29 min, purity: 95%, M/z $[M+H]^+$: 264; HRMS calcd. For $C_{12}H_{10}ClN_3O_2$ $[M+H]^+$= 264.0534, found 264.0545.

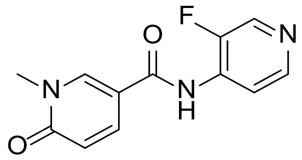

***N*-(3-Fluoropyridin-4-yl)-1-methyl-6-oxo-1,6-dihydropyridine-3-carboxamide** (**NPD-1189**)

^{1}H NMR (250 MHz, DMSO-d_6) δ 10.20 (s, 1H), 8.61- 8.55 (m, 2H), 8.36 (d, *J* = 5.3 Hz, 1H), 7.94- 7.87 (m, 2H), 6.46 (d, *J* = 9.5 Hz, 1H), 3.51 (s, 3H). ^{13}C NMR (126 MHz, DMSO-d_6) δ 163.3, 161.8, 150.8 (d, *J* = 255.1 Hz), 146.4 (d, *J* = 5.1 Hz), 143.9, 138.3, 138.1 (d, *J* = 21.7 Hz), 133.4 (d, *J* = 9.0 Hz), 118.0 (app s), 117.8, 111.3, 37.5. LC-MS: t_R= 1.94 min, purity: >99%, M/z $[M+H]^+$: 248; HRMS calcd. For $C_{12}H_{10}FN_3O_2$ $[M+H]^+$= 248.0830, found 248.0840.

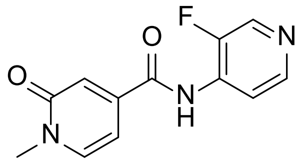

***N*-(3-Fluoropyridin-4-yl)-1-methyl-2-oxo-1,2-dihydropyridine-4-carboxamide** (**NPD-1190**)

^{1}H NMR (250 MHz, DMSO-d_6) δ 8.55 (d, *J* = 2.9 Hz, 1H), 8.35 (d, *J* = 5.3 Hz, 1H), 7.93 - 7.78 (m, 2H), 6.94 (d, *J* = 1.6 Hz, 1H), 6.60 (dd, *J* = 7.0, 2.0 Hz, 1H), 3.47 (s, 3H). ^{13}C NMR (126 MHz, DMSO-d_6) δ 164.5, 161.7, 151.5 (d, *J* = 255.8 Hz), 146.2 (d, *J* = 5.1 Hz), 144.9, 140.2, 138.1 (d, *J* = 21.9 Hz), 134.4 (app s), 118.8, 118.4 (app s), 103.2, 36.9. LC-MS: t_R= 2.00 min, purity: >99%, M/z $[M+H]^+$: 248; HRMS calcd. For $C_{12}H_{10}FN_3O_2$ $[M+H]^+$= 248.0830, found 248.0829.

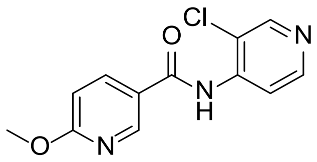

***N*-(3-Chloropyridin-4-yl)-6-methoxynicotinamide** (**NPD-1191**)

^{1}H NMR (250 MHz, DMSO-d_6) δ 10.20 (s, 1H), 8.81 (d, *J* = 2.1 Hz, 1H), 8.68 (s, 1H), 8.51 (d, *J* = 5.4 Hz, 1H), 8.24 (dd, *J* = 8.7, 2.5 Hz, 1H), 7.89 (d, *J* = 5.4 Hz, 1H), 6.98 (d, *J* = 8.7 Hz, 1H), 3.95 (s, 3H). ^{13}C NMR (126 MHz, DMSO-d_6) δ 166.3, 164.5, 150.2, 149.1, 148.5, 142.7, 139.4, 124.7, 123.4, 120.1, 110.9, 54.3. LC-MS: t_R= 3.37 min, purity: >99%, M/z [M+H]$^+$: 264; HRMS calcd. For $C_{12}H_{10}ClN_3O_2$ [M+H]$^+$= 264.0534, found 264.0535.

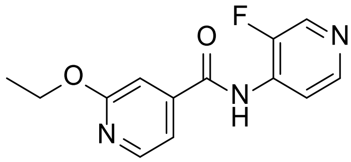

2-Ethoxy-*N*-(3-fluoropyridin-4-yl)isonicotinamide (**NPD-1192**)

^{1}H NMR (250 MHz, DMSO-d_6) δ 10.69 (s, 1H), 8.61 (d, *J* = 2.8 Hz, 1H), 8.40 (d, *J* = 5.3 Hz, 1H), 8.34 (dd, *J* = 5.3, 0.8 Hz, 1H), 7.91 (dd, *J* = 6.7, 5.3 Hz, 1H), 7.40 (dd, *J* = 5.3, 1.5 Hz, 1H), 7.27 (dd, *J* = 1.5, 0.8 Hz, 1H), 4.36 (q, *J* = 7.0 Hz, 2H), 1.33 (t, *J* = 7.0 Hz, 3H). ^{13}C NMR (126 MHz, DMSO-d_6) δ 164.4, 163.7, 151.2 (d, *J* = 256.2 Hz), 147.9, 146.4 (d, *J* = 5.3 Hz), 143.9, 138.3 (d, *J* = 21.7 Hz), 132.9 (d, *J* = 9.3 Hz), 118.6 (app s), 115.0, 109.4, 61.8, 14.5. LC-MS: t_R= 3.39 min, purity: >99%, M/z [M+H]$^+$: 262; HRMS calcd. For $C_{13}H_{12}FN_3O_2$ [M+H]$^+$= 262.0986, found 262.0989.

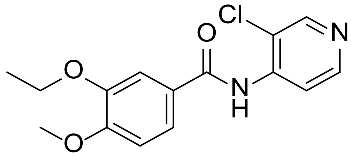

***N*-(3-Chloropyridin-4-yl)-3-ethoxy-4-methoxybenzamide** (**NPD-1193**)

^{1}H NMR (250 MHz, DMSO-d_6) δ 9.94 (s, 1H), 8.67 (s, 1H), 8.49 (d, *J* = 5.4 Hz, 1H), 7.86 (d, *J* = 5.4 Hz, 1H), 7.64 (dd, *J* = 8.4, 2.1 Hz, 1H), 7.54 (d, *J* = 2.1 Hz, 1H), 7.11 (d, *J* = 8.5 Hz, 1H), 4.09 (q, *J* = 7.0 Hz, 2H), 3.85 (s, 3H), 1.36 (t, *J* = 6.9 Hz, 3H). ^{13}C NMR (126 MHz, DMSO-d_6) δ 165.4, 152.8, 150.1, 149.0, 148.1, 143.1, 125.8, 124.8, 122.0, 120.2, 112.4, 111.6, 64.3, 56.2, 15.2. LC-MS: t_R= 3.83 min, purity: >99%, M/z [M+H]$^+$: 307; HRMS calcd. For $C_{15}H_{15}ClN_2O_3$ [M+H]$^+$= 307.0844, found 307.0852.

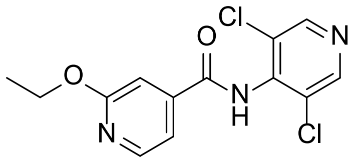

***N*-(3,5-Dichloropyridin-4-yl)-2-ethoxyisonicotinamide** (**NPD-1194**)

^{1}H NMR (250 MHz, DMSO-d_6) δ 10.95 (s, 1H), 8.79 (s, 2H), 8.39 (dd, *J* = 5.3, 0.6 Hz, 2H), 7.45 (d, *J* = 5.3, 1.5 Hz, 1H), 7.35 - 7.28 (m, 1H), 4.38 (q, *J* = 7.0 Hz, 2H), 1.35 (t, *J* = 7.0 Hz, 3H). ^{13}C NMR (126 MHz, DMSO-d_6) δ 164.3, 163.7,

148.9, 148.7, 143.3, 141.0, 131.1, 115.2, 109.5, 62.3, 14.9. LC-MS: t_R= 4.01 min, purity: >99%, M/z $[M+H]^+$: 312; HRMS calcd. For $C_{13}H_{11}Cl_2N_3O_2$ $[M+H]^+$= 312.0301, found 312.0296.

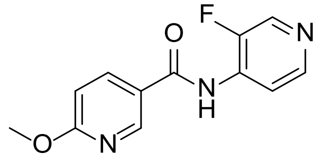

***N*-(3-Fluoropyridin-4-yl)-6-methoxynicotinamide** (**NPD-1195**)

^{1}H NMR (250 MHz, DMSO-d_6) δ 10.51 (s, 1H), 8.78 (dd, *J* = 2.5, 0.8 Hz, 1H), 8.59 (d, *J* = 2.8 Hz, 1H), 8.38 (d, *J* = 5.3 Hz, 1H), 8.22 (dd, *J* = 8.7, 2.5 Hz, 1H), 7.93 (dd, *J* = 6.7, 5.3 Hz, 1H), 6.96 (dd, *J* = 8.7, 0.7 Hz, 1H), 3.93 (s, 3H). ^{13}C NMR (126 MHz, DMSO-d_6) δ 166.2, 164.8, 151.5 (d, *J* = 255.5 Hz), 148.7, 146.8 (d, *J* = 5.2 Hz), 139.5, 138.6 (d, *J* = 21.5 Hz), 133.8 (d, *J* = 9.3 Hz), 123.5, 118.7 (app s), 110.8, 54.3. LC-MS: t_R= 2.83 min, purity: >99%, M/z $[M+H]^+$: 248; HRMS calcd. For $C_{12}H_{10}FN_3O_2$ $[M+H]^+$= 248.0830, found 248.0830.

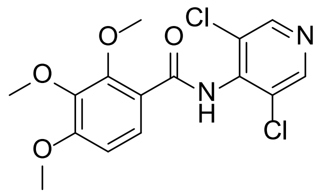

***N*-(3,5-Dichloropyridin-4-yl)-2,3,4-trimethoxybenzamide** (**NPD-1196**)

^{1}H NMR (500 MHz, $CDCl_3$) δ 10.01 (s, 1H), 8.56 (s, 2H), 7.99 (d, *J* = 9.0 Hz, 1H), 6.84 (d, *J* = 9.0 Hz, 1H), 4.16 (s, 3H), 3.95 (s, 3H), 3.92 (s, 3H). ^{13}C NMR (126 MHz, $CDCl_3$) δ 162.0, 157.8, 152.7, 148.0, 141.8, 140.9, 129.5, 127.7, 117.2, 108.0, 62.5, 61.2, 56.4. LC-MS: t_R= 4.27 min, purity: >99%, M/z $[M+H]^+$: 357; HRMS calcd. For $C_{15}H_{14}Cl_2N_2O_4$ $[M+H]^+$= 357.0403, found 357.0403. Spectral data are in agreement with a previous report. [3]

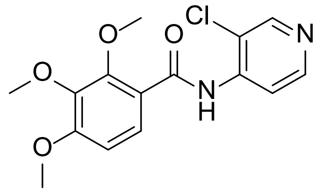

***N*-(3-Chloropyridin-4-yl)-2,3,4-trimethoxybenzamide** (**NPD-1197**)

^{1}H NMR (500 MHz, $CDCl_3$) δ 11.08 (s, 1H), 8.74 (d, *J* = 5.8 Hz, 1H), 8.58 (s, 1H), 8.44 (d, *J* = 5.8 Hz, 1H), 8.00 (d, *J* = 9.0 Hz, 1H), 6.85 (d, *J* = 9.1 Hz, 1H), 4.15 (s, 3H), 3.95 (s, 3H), 3.91 (s, 3H). ^{13}C NMR (126 MHz, $CDCl_3$) δ 163.7, 158.1, 152.7, 147.7, 147.6, 144.0, 141.7, 127.7, 120.1, 117.4, 115.1, 108.0, 62.5, 61.2, 56.3. LC-MS: t_R= 4.06 min, purity: >99%, M/z $[M+H]^+$: 323; HRMS calcd. For $C_{15}H_{15}ClN_2O_4$ $[M+H]^+$= 323.0793, found 323.0795.

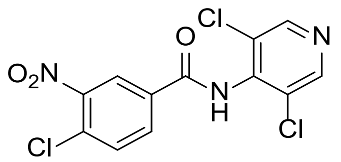

4-Chloro-*N*-(3,5-dichloropyridin-4-yl)-3-nitrobenzamide (**NPD-1198**)

^{1}H NMR (500 MHz, $CDCl_3$ + 1 drop of DMSO-d_6) δ 10.44 (s, 1H), 8.70 (s, 1H), 8.51 (s, 2H), 8.21 (d, *J* = 9.5 Hz, 1H), 7.63 (d, *J* = 9.5 Hz, 1H). ^{13}C NMR (126 MHz, $CDCl_3$ + 1 drop of DMSO-d_6) δ 162.4, 148.1, 147.6, 140.7, 133.3, 132.6, 132.1, 130.77, 130.75, 125.5. LC-MS: t_R= 4.31 min, purity: >99%, M/z [M+H]$^+$: 346; HRMS calcd. For $C_{12}H_6Cl_3N_3O_3$ [M+H]$^+$= 345.9548, found 345.9547.

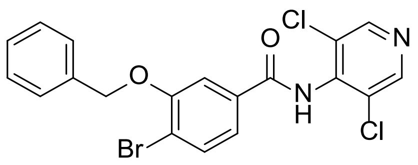

3-(Benzyloxy)-4-bromo-*N*-(3,5-dichloropyridin-4-yl)benzamide (**NPD-1199**)

^{1}H NMR (500 MHz, $CDCl_3$) δ 9.82 (s, 1H), 8.45 (s, 2H), 7.62 - 7.53 (m, 2H), 7.49 -7.43 (m, 1H), 7.42 - 7.35 (m, 2H), 7.32 - 7.19 (m, 3H), 5.12 (s, 2H). ^{13}C NMR (126 MHz, $CDCl_3$) δ 164.5, 154.9, 148.0, 140.7, 135.8, 133.1, 133.0, 130.5, 128.4, 127.9, 127.0, 121.4, 116.9, 113.2, 70.6. LC-MS: t_R= 5.07 min, purity: >99%, M/z [M+H]$^+$: 453; HRMS calcd. For $C_{19}H_{13}BrCl_2N_2O_2$ [M+H]$^+$= 450.9610, found 450.9621.

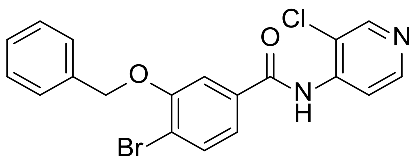

3-(Benzyloxy)-4-bromo-*N*-(3-chloropyridin-4-yl)benzamide (**NPD-1200**)

^{1}H NMR (500 MHz, $CDCl_3$) δ 8.65 - 8.56 (m, 2H), 8.56 - 8.46 (m, 2H), 7.73 (d, *J* = 8.2 Hz, 1H), 7.53 - 7.47 (m, 3H), 7.44 - 7.39 (m, 2H), 7.37 - 7.33 (m, 1H), 7.30 (dd, *J* = 8.2, 2.0 Hz, 1H), 5.28 (s, 2H). ^{13}C NMR (126 MHz, $CDCl_3$) δ 164.7, 155.9, 148.3, 148.0, 142.3, 135.7, 134.1, 133.8, 128.9, 128.4, 127.1, 120.1, 119.7, 118.5, 114.4, 112.7, 71.1. LC-MS: t_R= 5.17 min, purity: >99%, M/z [M+H]$^+$: 417; HRMS calcd. For $C_{19}H_{14}BrClN_2O_2$ [M+H]$^+$= 417.0000, found 417.0006.

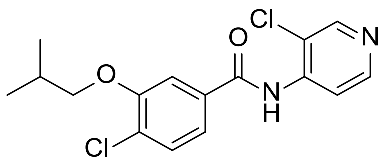

4-Chloro-*N*-(3-chloropyridin-4-yl)-3-isobutoxybenzamide (**NPD-1201**)

^{1}H NMR (500 MHz, $CDCl_3$) δ 8.62 - 8.56 (m, 3H), 8.50 (d, *J* = 5.7 Hz, 1H), 7.53 - 7.47 (m, 2H), 7.31 (dd, *J* = 8.2, 2.0 Hz, 1H), 3.88 (d, *J* = 6.5 Hz, 2H), 2.19 (app hept, *J* = 6.6 Hz, 1H), 1.08 (d, *J* = 6.7 Hz, 6H). ^{13}C NMR (126 MHz, $CDCl_3$) δ 164.8,

155.6, 148.4, 148.1, 142.3, 133.0, 130.7, 128.6, 120.1, 118.5, 114.4, 112.5, 75.7, 28.4, 19.3. LC-MS: t_R= 5.34 min, purity: 98%, M/z [M+H]$^+$: 339; HRMS calcd. For $C_{16}H_{16}Cl_2N_2O_2$ [M+H]$^+$= 339.0662, found 339.0670.

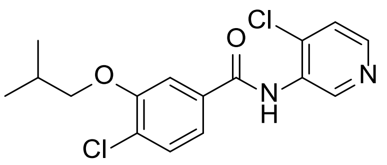

4-Chloro-*N*-(4-chloropyridin-3-yl)-3-isobutoxybenzamide (**NPD-1202**)

^{1}H NMR (500 MHz, DMSO-d_6) δ 10.44 (s, 1H), 8.71 (s, 1H), 8.47 (d, *J* = 5.2 Hz, 1H), 7.71 (s, 2H), 7.68 - 7.56 (m, 2H), 3.96 (d, *J* = 6.3 Hz, 2H), 2.12 (app hept, *J* = 6.5 Hz, 1H), 1.05 (d, *J* = 6.6 Hz, 6H). ^{13}C NMR (126 MHz, DMSO-d_6) δ 164.7, 154.0, 149.6, 148.2, 139.7, 133.4, 132.2, 130.0, 125.4, 124.7, 120.8, 112.7, 74.8, 27.8, 19.0. LC-MS: t_R= 4.95 min, purity: >99%, M/z [M+H]$^+$: 339; HRMS calcd. For $C_{16}H_{16}Cl_2N_2O_2$ [M+H]$^+$= 339.0662, found 339.0675.

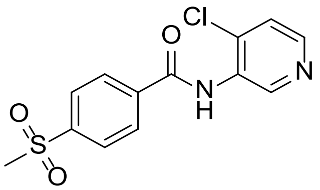

***N*-(4-Chloropyridin-3-yl)-4-(methylsulfonyl)benzamide** (**NPD-1203**)

^{1}H NMR (500 MHz, DMSO-d_6) δ 10.71 (s, 1H), 8.72 (s, 1H), 8.48 (d, *J* = 5.3 Hz, 1H), 8.23 (d, *J* = 8.5 Hz, 2H), 8.12 (d, *J* = 8.5 Hz, 2H), 7.72 (d, *J* = 5.3 Hz, 1H), 3.31 (s, 3H). ^{13}C NMR (126 MHz, DMSO-d_6) δ 164.7, 149.5, 148.5, 143.7, 139.8, 137.8, 131.9, 128.9, 127.4, 124.9, 43.3. LC-MS: t_R= 2.90 min, purity: 96%, M/z [M+H]$^+$: 311; HRMS calcd. For $C_{13}H_{11}ClN_2O_3S$ [M+H]$^+$= 311.0252, found 311.0257.

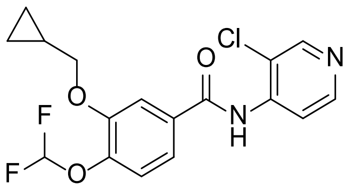

***N*-(3-Chloropyridin-4-yl)-3-(cyclopropylmethoxy)-4-(difluoromethoxy)benzamide** (**NPD-1204**)

^{1}H NMR (500 MHz, $CDCl_3$) δ 8.58 (s, 1H), 8.52 (d, *J* = 5.5 Hz, 2H), 8.48 (d, *J* = 5.6 Hz, 1H), 7.57 (d, *J* = 2.1 Hz, 1H), 7.38 (dd, *J* = 8.3, 2.1 Hz, 1H), 7.30 (d, *J* = 8.3 Hz, 1H), 6.74 (t, *J* = 74.7 Hz, 1H), 3.97 (d, *J* = 7.0 Hz, 2H), 1.37 - 1.28 (m, 1H), 0.71 - 0.66 (m, 2H), 0.41 - 0.37 (m, 2H). ^{13}C NMR (126 MHz, $CDCl_3$) δ 164.7, 151.2, 149.4, 149.1, 143.9, 141.4, 131.9, 122.5, 120.0, 119.0, 115.7 (t, *J* = 262.0 Hz), 114.3, 114.0, 74.3, 10.1, 3.5. LC-MS: t_R= 4.68 min, purity: >99%, M/z [M+H]$^+$: 369; HRMS calcd. For $C_{17}H_{15}ClF_2N_2O_3$ [M+H]$^+$= 369.0812, found 369.0806.

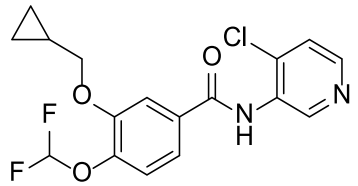

***N*-(4-Chloropyridin-3-yl)-3-(cyclopropylmethoxy)-4-(difluoromethoxy)benzamide** (**NPD-1205**)

^{1}H NMR (500 MHz, DMSO-d_6) δ 10.37 (s, 1H), 8.68 (s, 1H), 8.46 (d, *J* = 5.3 Hz, 1H), 7.74 - 7.68 (m, 2H), 7.64 (dd, *J* = 8.4, 2.0 Hz, 1H), 7.40 - 7.08 (m, 2H), 3.99 (d, *J* = 7.0 Hz, 2H), 1.28 (m, 1H), 0.62 - 0.57 (m, 2H), 0.40 - 0.35 (m, 2H). ^{13}C NMR (126 MHz, DMSO-d_6) δ 164.7, 149.6, 149,6 (confirmed by HMBC), 148.2, 142.7 (t, *J* = 3.1 Hz), 140.0, 132.2, 131.2, 124.8, 120.7, 120.3, 116.5 (t, *J* = 258.3 Hz), 113.6, 73.3, 10.0, 3.1. LC-MS: t_R= 4.38 min, purity: >99%, M/z [M+H]$^+$: 369; HRMS calcd. For $C_{17}H_{15}ClF_2N_2O_3$ [M+H]$^+$= 369.0812, found 369.0805.

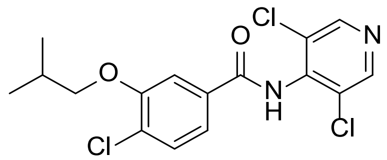

4-Chloro-*N*-(3,5-dichloropyridin-4-yl)-3-isobutoxybenzamide (**NPD-1206**)

^{1}H NMR (500 MHz, CDCl$_3$) δ 8.59 – 8.50 (m, 3H), 8.46 (dd, *J* = 5.7, 3.0 Hz, 1H), 7.51 - 7.45 (m, 2H), 7.32 – 7.26 (m, 1H), 3.89 – 3.83 (m, 2H), 2.25 – 2.10 (m, 1H), 1.09 – 1.04 (m, 6H). ^{13}C NMR (126 MHz, DMSO-d_6) δ 166.2, 157.7, 150.7, 142.0, 134.4, 132.7, 131.1, 130.7, 121.7, 115.0, 77.9, 30.6, 21.5. LC-MS: t_R= 5.22 min, purity: 99%, M/z [M+H]$^+$: 373; HRMS calcd. For $C_{16}H_{15}Cl_3N_2O_2$ [M+Na]$^+$= 395.0091, found 395.0073.

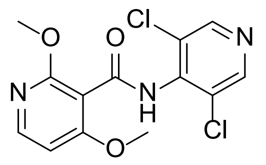

***N*-(3,5-Dichloropyridin-4-yl)-2,4-dimethoxynicotinamide** (**NPD-1472**)

^{1}H NMR (300 MHz, DMSO-d_6) δ 9.97 (s, 1H), 8.73 (s, 2H), 8.21 (d, *J* = 8.3 Hz, 1H), 6.56 (d, *J* = 8.3 Hz, 1H), 4.08 (s, 3H), 3.97 (s, 3H). ^{13}C NMR (126 MHz, DMSO-d_6) δ 164.8, 161.5, 160.4, 148.1, 143.9, 141.5, 130.7, 106.6, 102.7, 54.2, 54.1. LC-MS: t_R= 4.58 min, purity: >99%, M/z [M+H]$^+$: 328; HRMS calcd. For $C_{13}H_{11}Cl_2N_3O_3$ [M+H]$^+$= 328.0250, found 328.0254.

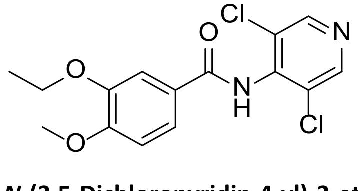

***N*-(3,5-Dichloropyridin-4-yl)-3-ethoxy-4-methoxybenzamide** (**NPD-2976**)

^{1}H NMR (600 MHz, DMSO-d_6) δ 10.42 (s, 1H), 8.74 (s, 2H), 7.67 (dd, *J* = 8.5, 2.1 Hz, 1H), 7.56 (d, *J* = 2.1 Hz, 1H), 7.12 (d, *J* = 8.4 Hz, 1H), 4.09 (q, *J* = 7.0 Hz, 2H), 3.85 (s, 3H), 1.36 (t, *J* = 6.9 Hz, 3H). ^{13}C NMR (151 MHz, DMSO-d_6) δ 164.2, 152.4, 148.2, 147.7, 141.7 (confirmed by HMBC), 130.7, 124.8, 121.6, 112.0, 111.2, 63.9, 55.7, 14.7. LC-MS: t_R= 3.81 min, purity: >99%, M/z [M+H]$^+$: 341; HRMS calcd. For $C_{15}H_{14}Cl_2N_2O_3$ [M+H]$^+$= 341.0454, found 341.0457. Spectral data are in agreement with a previous report. [4]

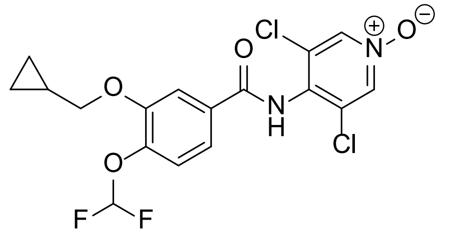

3,5-Dichloro-4-(3-(cyclopropylmethoxy)-4-(difluoromethoxy)benzamido)pyridine 1-oxide (**NPD-2980**)

To a stirred, ice-cooled (0 ˚C) solution of **NPD-0006** (0.15 g, 0.37 mmol) in DCM (15 mL) was added portion-wise *m*-CPBA (0.11 g, 0.48 mmol). The reaction mixture was warmed to room temperature and stirred overnight. Saturated aq. $NaHCO_3$ (10 mL) was slowly added and the mixture was extracted with DCM (3 x 15 mL). The combined organic layers were washed with brine (50 mL), dried over Na_2SO_4, filtered and concentrated *in vacuo*. The crude product was purified with flash column chromatography using a gradient elution of EtOAc in cyclohexane (30% to 80%) to 5:1 as eluents to yield the title compound as a white solid. ^{1}H NMR (600 MHz, $CDCl_3$) δ 8.25 (s, 2H), 7.82 (s, 1H), 7.58 (s, 1H), 7.46 (d, *J* = 8.3 Hz, 1H), 7.28 (d, *J* = 8.2 Hz, 1H), 6.74 (t, *J* = 74.8 Hz, 1H), 3.96 (d, *J* = 6.9 Hz, 2H), 1.36 - 1.27 (m, 1H), 0.70 - 0.65 (m, 2H), 0.40 - 0.36 (m, 2H). ^{13}C NMR (151 MHz, $CDCl_3$) δ 164.4, 151.1, 144.1, 138.5, 131.8, 131.4, 130.6, 122.5, 119.9, 115.8 (t, *J* = 264.3 Hz), 114.3, 74.4, 10.1, 3.4. LC-MS: t_R= 4.01 min, purity: 98%, M/z [M+H]$^+$: 419; HRMS calcd. For $C_{17}H_{14}Cl_2F_2N_2O_4$ [M+H]$^+$= 419.0371, found 419.0364. Spectral data are in agreement with a previous report. [5]

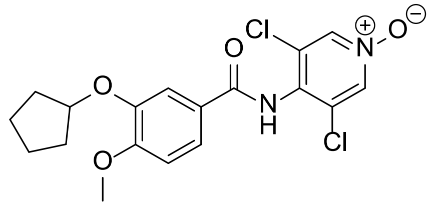

3,5-Dichloro-4-(3-(cyclopentyloxy)-4-methoxybenzamido)pyridine 1-oxide (**NPD-2981**)

Prepared from **NPD-0005** (0.15 g, 0.39 mmol) with *m*-CPBA (0.12 g, 0.51 mmol) as described for **NPD-2980** to give the title compound as a white solid. ^{1}H NMR (500 MHz, $CDCl_3$) δ 8.28 (s, 2H), 7.74 (s, 1H), 7.59 - 7.43 (m, 2H), 6.93 (d, *J* = 7.8 Hz, 1H), 4.87 (s, 1H), 3.93 (s, 3H), 2.07 - 1.78 (m, 6H), 1.63 (app s, 2H). ^{13}C NMR (126 MHz, $CDCl_3$) δ 164.6, 154.4, 148.2, 138.5, 134.4, 131.0, 124.5, 120.8, 114.3, 111.0, 80.9, 56.3, 32.9, 24.2. LC-MS: t_R= 3.74 min, purity: 97%,

M/z [M+H]$^+$: 397; HRMS calcd. For $C_{18}H_{18}Cl_2N_2O_4$ [M+H]$^+$= 397.0716, found 397.0705. Spectral data are in agreement with a previous report. [1]

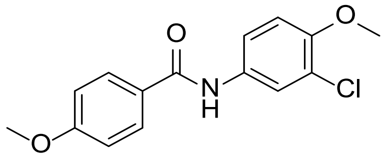

***N*-(3-Chloro-4-methoxyphenyl)-4-methoxybenzamide** (**NPD-2991**)

^{1}H NMR (500 MHz, $CDCl_3$) δ 7.82 (d, *J* = 8.5 Hz, 2H), 7.71 - 7.65 (m, 2H), 7.50 (dd, *J* = 8.8, 2.1 Hz, 1H), 6.96 (d, *J* = 8.6 Hz, 2H), 6.91 (d, *J* = 8.8 Hz, 1H), 3.90 (s, 3H), 3.87 (s, 3H). ^{13}C NMR (126 MHz, $CDCl_3$) δ 165.3, 162.7, 152.1, 131.7, 129.0, 126.8, 122.9, 122.6, 120.1, 114.1, 112.4, 56.5, 55.6. LC-MS: t_R= 4.32 min, purity: >99%, M/z [M+H]$^+$: 292; HRMS calcd. For $C_{15}H_{14}ClNO_3$ [M+H]$^+$= 292.0735, found 292.0728.

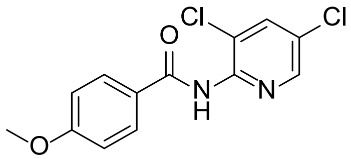

***N*-(3,5-Dichloropyridin-2-yl)-4-methoxybenzamide** (**NPD-2992**)

^{1}H NMR (500 MHz, $CDCl_3$) δ 8.38 (d, *J* = 1.7 Hz, 1H), 8.30 (s, 1H), 7.90 (d, *J* = 8.7 Hz, 2H), 7.78 (d, *J* = 2.1 Hz, 1H), 6.99 (d, *J* = 8.7 Hz, 2H), 3.88 (s, 3H). ^{13}C NMR (126 MHz, $CDCl_3$) δ 164.4, 163.2, 146.6, 145.6, 137.7, 129.7, 127.9, 126.0, 123.0, 114.2, 55.7. LC-MS: t_R= 4.00 min, purity: >99%, M/z [M+H]$^+$: 297; HRMS calcd. For $C_{13}H_{10}Cl_2N_2O_2$ [M+H]$^+$= 297.0192, found 297.0186.

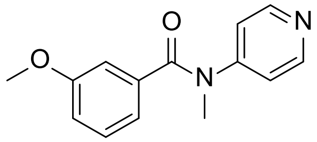

3-Methoxy-*N*-methyl-*N*-(pyridin-4-yl)benzamide (**NPD-2993**)

^{1}H NMR (500 MHz, $CDCl_3$) δ 8.44 (d, *J* = 4.6 Hz, 2H), 7.13 (t, *J* = 7.9 Hz, 1H), 6.96 (d, *J* = 6.0 Hz, 2H), 6.92 (s, 1H), 6.88 (dd, *J* = 8.2, 2.4 Hz, 1H), 6.83 (d, *J* = 7.6 Hz, 1H), 3.71 (s, 3H), 3.51 (s, 3H). ^{13}C NMR (126 MHz, $CDCl_3$) δ 170.7, 159.5, 152.2, 150.6, 136.3, 129.4, 121.1, 120.2, 116.8, 113.9, 55.4, 37.5. LC-MS: t_R= 2.48 min, purity: >99%, M/z [M+H]$^+$: 243; HRMS calcd. For $C_{14}H_{14}N_2O_2$ [M+H]$^+$= 243.1128, found 243.1130.

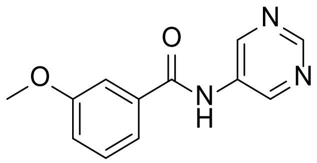

3-Methoxy-*N*-(pyrimidin-5-yl)benzamide (**NPD-2994**)

^{1}H NMR (600 MHz, $CDCl_3$) δ 9.20 (s, 2H), 9.01 (s, 1H), 8.17 (s, 1H), 7.48 - 7.39 (m, 3H), 7.13 (ddd, *J* = 7.9, 2.5, 1.1 Hz, 1H), 3.88 (s, 3H). ^{13}C NMR (151 MHz, $CDCl_3$) δ 166.0, 160.3, 153.8, 148.2, 134.8, 133.9, 130.2, 119.1, 119.0, 112.9, 55.7. LC-MS: t_R= 3.06 min, purity: >99%, M/z $[M+H]^+$: 230; HRMS calcd. For $C_{12}H_{11}N_3O_2$ $[M+H]^+$= 230.0924, found 230.0916.

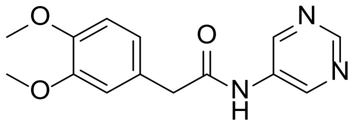

2-(3,4-Dimethoxyphenyl)-*N*-(pyrimidin-5-yl)acetamide (**NPD-2995**)

^{1}H NMR (500 MHz, $CDCl_3$) δ 8.97 - 8.88 (m, 3H), 7.41 (s, 1H), 6.92 - 6.85 (m, 2H), 6.82 (s, 1H), 3.91 - 3.87 (m, 6H), 3.73 (s, 2H). ^{13}C NMR (126 MHz, $CDCl_3$) δ 170.2, 154.3, 149.7, 149.0, 147.8, 133.3, 125.9, 121.9, 112.4, 111.9, 56.11, 56.07, 44.1. LC-MS: t_R= 2.78 min, purity: >99%, M/z $[M+H]^+$: 274; HRMS calcd. For $C_{14}H_{15}N_3O_3$ $[M+H]^+$= 274.1186, found 274.1190.

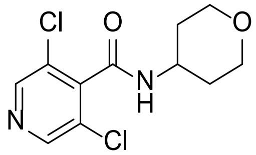

3,5-Dichloro-*N*-(tetrahydro-2*H*-pyran-4-yl)isonicotinamide (**NPD-2996**)

^{1}H NMR (500 MHz, $CDCl_3$) δ 8.52 (s, 2H), 5.80 (d, *J* = 7.3 Hz, 1H), 4.31 - 4.22 (m, 1H), 3.98 (s, 2H), 3.58 - 3.49 (m, 2H), 2.08 - 2.02 (m, 2H), 1.66 - 1.53 (m, 2H). ^{13}C NMR (126 MHz, $CDCl_3$) δ 161.4, 147.9, 142.2, 129.1, 66.7, 46.7, 32.9. LC-MS: t_R= 2.78 min, purity: >99%, M/z $[M+H]^+$: 275; HRMS calcd. For $C_{11}H_{12}Cl_2N_2O_2$ $[M+H]^+$= 275.0349, found 275.0338.

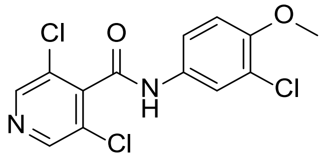

3,5-Dichloro-*N*-(3-chloro-4-methoxyphenyl)isonicotinamide (**NPD-2997**)

^{1}H NMR (500 MHz, $CDCl_3$) δ 8.56 (s, 2H), 7.68 - 7.64 (m, 1H), 7.59 (s, 1H), 7.52 - 7.46 (m, 1H), 6.94 (d, *J* = 8.8 Hz, 1H), 3.92 (s, 3H). ^{13}C NMR (126 MHz, $CDCl_3$) δ 160.1, 153.1, 148.0, 141.8, 129.9, 129.2, 123.3, 123.0, 120.5, 112.4, 56.6. LC-MS: t_R= 4.26 min, purity: >99%, M/z $[M+H]^+$: 331; HRMS calcd. For $C_{13}H_9Cl_3N_2O_2$ $[M+H]^+$= 330.9802, found 330.9799.

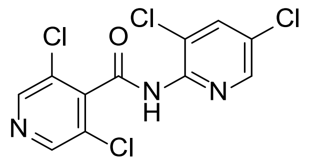

3,5-Dichloro-*N*-(3,5-dichloropyridin-2-yl)isonicotinamide (**NPD-2998**)

^{1}H NMR (500 MHz, DMSO-*d*$_6$) δ 8.52 (s, 2H), 8.42 (s, 1H), 7.75 (s, 2H). ^{13}C NMR (126 MHz, DMSO-*d*$_6$) δ 149.0, 147.5, 145.1, 144.2, 137.9, 128.5. Note: not all Carbon signals visible in spectrum. LC-MS: t_R= 4.07 min, purity: >99%, M/z [M+H]$^+$: 338; HRMS calcd. For $C_{11}H_5Cl_4N_3O$ [M+H]$^+$= 335.9259, found 335.9260.

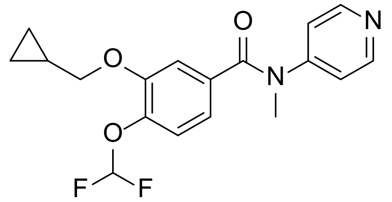

3-(Cyclopropylmethoxy)-4-(difluoromethoxy)-*N*-methyl-*N*-(pyridin-4-yl)benzamide (**NPD-2999**)

^{1}H NMR (500 MHz, CDCl$_3$) δ 8.47 (d, *J* = 5.3 Hz, 2H), 7.04 - 6.98 (m, 4H), 6.85 (dd, *J* = 8.3, 2.0 Hz, 1H), 6.62 (t, *J* = 74.9 Hz, 1H), 3.74 (d, *J* = 7.0 Hz, 2H), 3.52 (s, 3H), 1.22 - 1.10 (m, 1H), 0.66 - 0.57 (m, 2H), 0.34 - 0.28 (m, 2H). ^{13}C NMR (126 MHz, CDCl$_3$) δ 169.9, 152.7, 150.3, 149.8, 142.3, 132.8, 122.0, 121.9, 119.9, 115.8 (t, *J* = 260.5 Hz), 115.0, 74.1, 37.7, 10.0, 3.4. LC-MS: t_R= 3.60 min, purity: >99%, M/z [M+H]$^+$: 349; HRMS calcd. For $C_{18}H_{18}F_2N_2O_3$ [M+H]$^+$= 349.1358, found 349.1363.

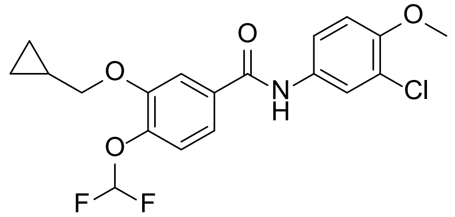

***N*-(3-Chloro-4-methoxyphenyl)-3-(cyclopropylmethoxy)-4-(difluoromethoxy)benzamide** (**NPD-3000**)

^{1}H NMR (500 MHz, CDCl$_3$) δ 7.73 - 7.66 (m, 2H), 7.53 (s, 1H), 7.49 (d, *J* = 8.6 Hz, 1H), 7.31 (d, *J* = 8.0 Hz, 1H), 7.23 (d, *J* = 8.2 Hz, 1H), 6.93 (d, *J* = 8.8 Hz, 1H), 6.72 (t, *J* = 75.0 Hz, 1H), 3.94 (d, *J* = 6.9 Hz, 2H), 3.91 (s, 3H), 1.31 (m, 1H), 0.67 (app d, *J* = 7.6 Hz, 2H), 0.37 (app d, *J* = 4.5 Hz, 2H). ^{13}C NMR (126 MHz, CDCl$_3$) δ 164.7, 152.4, 151.0, 143.1, 133.0, 131.3, 123.0, 122.8, 122.3, 120.2, 118.8, 115.9 (t, *J* = 260.4 Hz), 113.9, 112.4, 74.3, 56.5, 10.2, 3.4. LC-MS: t_R= 5.00 min, purity: >99%, M/z [M+H]$^+$: 398; HRMS calcd. For $C_{19}H_{18}ClF_2NO_4$ [M+H]$^+$= 398.0965, found 398.0955.

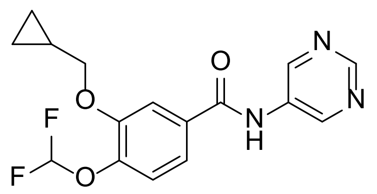

3-(Cyclopropylmethoxy)-4-(difluoromethoxy)-*N*-(pyrimidin-5-yl)benzamide (**NPD-3001**)

^{1}H NMR (500 MHz, $CDCl_3$) δ 9.16 (s, 2H), 9.02 (s, 1H), 8.18 (s, 1H), 7.56 (s, 1H), 7.40 (d, *J* = 8.1 Hz, 1H), 7.24 (d, *J* = 8.1 Hz, 1H), 6.74 (t, *J* = 74.8 Hz, 1H), 3.95 (d, *J* = 6.9 Hz, 2H), 1.31 (tt, *J* = 12.7, 7.8 Hz, 1H), 0.67 (app q, *J* = 7.6 Hz, 2H), 0.37 (app q, *J* = 4.7 Hz, 2H). ^{13}C NMR (126 MHz, $CDCl_3$) δ 165.2, 154.6, 151.1, 148.4, 143.7 (t, *J* = 3.2 Hz), 133.5, 131.6, 122.3, 119.2, 115.8 (t, *J* = 264.2 Hz), 114.0, 74.4, 10.1, 3.4. LC-MS: t_R= 4.09 min, purity: >99%, M/z $[M+H]^+$: 336; HRMS calcd. For $C_{16}H_{15}F_2N_3O_3$ $[M+H]^+$= 336.1154, found 336.1145.

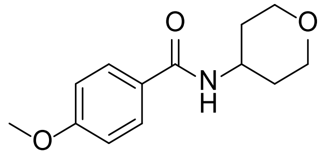

4-Methoxy-*N*-(tetrahydro-2*H*-pyran-4-yl)benzamide (**NPD-3002**)

^{1}H NMR (500 MHz, $CDCl_3$) δ 7.66 (d, *J* = 8.0 Hz, 2H), 6.85 (d, *J* = 8.0 Hz, 2H), 5.90 (d, *J* = 6.2 Hz, 1H), 4.17 - 4.07 (m, 1H), 3.93 (app d, *J* = 10.2 Hz, 2H), 3.78 (s, 3H), 3.46 (app t, *J* = 11.7 Hz, 2H), 1.93 (app d, *J* = 12.5 Hz, 2H), 1.49 (app q, *J* = 11.8 Hz, 2H). ^{13}C NMR (126 MHz, $CDCl_3$) δ 166.5, 162.4, 128.8, 126.9, 113.9, 67.1, 55.6, 46.3, 33.5. LC-MS: t_R= 3.01 min, purity: >99%, M/z $[M+H]^+$: 236; HRMS calcd. For $C_{13}H_{17}NO_3$ $[M+H]^+$= 236.1281, found 236.1272.

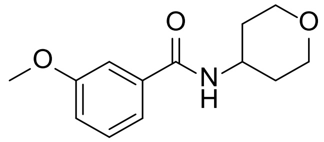

3-Methoxy-*N*-(tetrahydro-2*H*-pyran-4-yl)benzamide (**NPD-3003**)

^{1}H NMR (500 MHz, $CDCl_3$) δ 7.30 - 7.25 (m, 2H), 7.24 - 7.18 (m, 1H), 6.98 (d, *J* = 8.1 Hz, 1H), 6.04 - 5.93 (m, 1H), 4.21 - 4.08 (m, 1H), 3.94 (app d, *J* = 10.6 Hz, 2H), 3.79 (s, 3H), 3.48 (app t, *J* = 11.7 Hz, 2H), 1.95 (app d, *J* = 12.6 Hz, 2H), 1.51 (app q, *J* = 11.9 Hz, 2H).^{13}C NMR (151 MHz, $CDCl_3$) δ 166.8, 160.0, 136.2, 129.7, 118.7, 117.7, 112.5, 67.0, 55.6, 46.4, 33.3. LC-MS: t_R= 3.10 min, purity: >99%, M/z $[M+H]^+$: 236; HRMS calcd. For $C_{13}H_{17}NO_3$ $[M+H]^+$= 236.1281, found 236.1271.

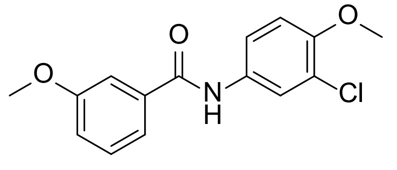

***N*-(3-Chloro-4-methoxyphenyl)-3-methoxybenzamide** (**NPD-3004**)

^{1}H NMR (500 MHz, $CDCl_3$) δ 7.86 (s, 1H), 7.68 (s, 1H), 7.50 (d, *J* = 8.8 Hz, 1H), 7.41 (s, 1H), 7.39 - 7.32 (m, 2H), 7.07 (s, 1H), 6.90 (d, *J* = 8.8 Hz, 1H), 3.89 (s, 3H), 3.85 (s, 3H). ^{13}C NMR (126 MHz, $CDCl_3$) δ 165.5, 160.0, 152.1, 136.1, 131.4, 129.8, 122.8, 122.5, 120.0, 118.6, 118.1, 112.4, 112.2, 56.4, 55.5. LC-MS: t_R= 4.39 min, purity: >99%, M/z $[M+H]^+$: 292; HRMS calcd. For $C_{15}H_{14}ClNO_3$ $[M+H]^+$= 292.0735, found 292.0721.

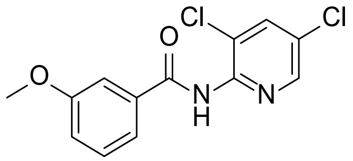

***N*-(3,5-Dichloropyridin-2-yl)-3-methoxybenzamide** (**NPD-3005**)

^{1}H NMR (500 MHz, $CDCl_3$) δ 8.38 (s, 2H), 7.79 (s, 1H), 7.49 (s, 1H), 7.45 (d, *J* = 7.5 Hz, 1H), 7.41 (t, *J* = 7.7 Hz, 1H), 7.13 (d, *J* = 8.0 Hz, 1H), 3.88 (s, 3H). ^{13}C NMR (126 MHz, $CDCl_3$) δ 164.8, 160.2, 146.3, 145.7, 137.7, 135.3, 130.1, 128.2, 123.1, 119.3, 118.9, 113.0, 55.7. LC-MS: t_R= 4.06 min, purity: >99%, M/z $[M+H]^+$: 297; HRMS calcd. For $C_{13}H_{10}Cl_2N_2O_2$ $[M+H]^+$= 297.0192, found 297.0182.

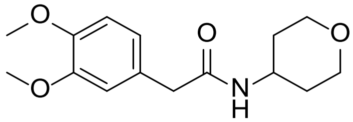

2-(3,4-Dimethoxyphenyl)-*N*-(tetrahydro-2H-pyran-4-yl)acetamide (**NPD-3006**)

^{1}H NMR (500 MHz, $CDCl_3$) δ 6.85 (d, *J* = 7.9 Hz, 1H), 6.80 - 6.75 (m, 2H), 5.29 (d, *J* = 7.4 Hz, 1H), 4.03 - 3.93 (m, 1H), 3.91 - 3.86 (m, 8H), 3.50 (s, 2H), 3.44 (app t, *J* = 11.6 Hz, 2H), 1.82 (app d, *J* = 12.7 Hz, 2H), 1.33 (app q, *J* = 11.7 Hz, 2H). ^{13}C NMR (151 MHz, $CDCl_3$) δ 170.7, 149.4, 148.5, 127.3, 121.7, 112.5, 111.7, 66.8, 56.1, 56.1, 45.8, 43.6, 33.1. LC-MS: t_R= 2.78 min, purity: >99%, M/z $[M+H]^+$: 280; HRMS calcd. For $C_{15}H_{21}NO_4$ $[M+H]^+$= 280.1543, found 280.1531.

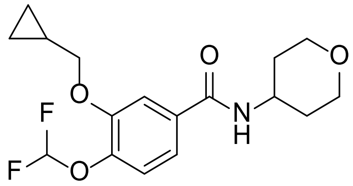

3-(Cyclopropylmethoxy)-4-(difluoromethoxy)-*N*-(tetrahydro-2H-pyran-4-yl)benzamide (**NPD-3007**)

^{1}H NMR (500 MHz, $CDCl_3$) δ 7.46 (s, 1H), 7.23 – 7.14 (m, 2H), 6.69 (t, *J* = 75.1 Hz, 1H), 5.95 (d, *J* = 6.7 Hz, 1H), 4.24 – 4.12 (m, 1H), 4.00 (app d, *J* = 10.8 Hz, 2H), 3.93 (app d, *J* = 6.8 Hz, 2H), 3.53 (app t, *J* = 11.7 Hz, 2H), 2.00 (app d, *J* = 12.0 Hz, 2H), 1.65 - 1.54 (m, 2H), 1.36 - 1.22 (m, 1H), 0.66 (app d, *J* = 7.0 Hz, 2H), 0.36 (app s, 2H). ^{13}C NMR (151 MHz, $CDCl_3$) δ 165.9, 150.8, 142.9 (t, *J* = 3.1 Hz), 122.3, 118.7, 115.9 (t, *J* = 261.1 Hz), 113.9, 74.3, 66.9, 46.6, 33.3, 10.2,

3.4. LC-MS: t_R= 4.15 min, purity: >99%, M/z $[M+H]^+$: 342; HRMS calcd. For $C_{17}H_{21}F_2NO_4$ $[M+H]^+$= 342.1511, found 342.1509.

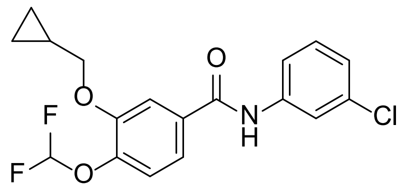

N-(3-Chlorophenyl)-3-(cyclopropylmethoxy)-4-(difluoromethoxy)benzamide (**NPD-3075**)

^{1}H NMR (500 MHz, $CDCl_3$) δ 7.80 (s, 1H), 7.76 (app t, *J* = 2.0 Hz, 1H), 7.53 (d, *J* = 2.0 Hz, 1H), 7.49 - 7.45 (m, 1H), 7.34 - 7.27 (m, 2H), 7.24 (d, *J* = 8.2 Hz, 1H), 7.14 (ddd, *J* = 8.0, 1.9, 0.9 Hz, 1H), 6.72 (t, *J* = 74.9 Hz, 1H), 3.94 (d, *J* = 7.0 Hz, 2H), 1.35 - 1.27 (m, 1H), 0.69 - 0.65 (m, 2H), 0.39 - 0.35 (m, 2H). ^{13}C NMR (126 MHz, $CDCl_3$) δ 164.8, 151.0, 143.3, 138.9, 135.0, 132.9, 130.3, 124.9, 122.4, 120.4, 118.8, 118.2, 115.9 (t, *J* = 263.6 Hz), 113.9, 74.3, 10.1, 3.4. LC-MS: t_R= 5.18 min, purity: >99%, M/z $[M+H]^+$: 368; HRMS calcd. For $C_{18}H_{16}ClF_2NO_3$ $[M+H]^+$= 368.0860, found 368.0854.

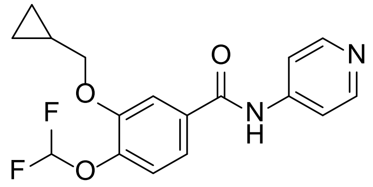

3-(Cyclopropylmethoxy)-4-(difluoromethoxy)-N-(pyridin-4-yl)benzamide (**NPD-3076**)

^{1}H NMR (500 MHz, $CDCl_3$) δ 8.55 (d, *J* = 5.1 Hz, 2H), 8.14 (s, 1H), 7.61 (d, *J* = 5.1 Hz, 2H), 7.53 (s, 1H), 7.35 (d, *J* = 8.2 Hz, 1H), 7.24 (d, *J* = 8.2 Hz, 1H), 6.73 (t, *J* = 74.8 Hz, 1H), 3.94 (d, *J* = 6.9 Hz, 2H), 1.31 (m, 1H), 0.67 (app d, *J* = 7.3 Hz, 2H), 0.37 (app d, *J* = 4.4 Hz, 2H). ^{13}C NMR (151 MHz, DMSO-d_6) δ 165.3, 151.1, 150.9, 145.1, 143.1 (t, *J* = 3.1 Hz), 132.4, 122.3, 119.1, 115.8 (t, *J* = 264.7 Hz), 114.0, 114.0, 74.3, 10.1, 3.4. LC-MS: t_R= 3.54 min, purity: >99%, M/z $[M+H]^+$: 335; HRMS calcd. For $C_{17}H_{16}F_2N_2O_3$ $[M+H]^+$= 335.1202, found 335.1209.

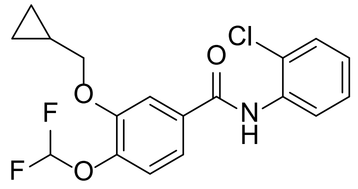

N-(2-Chlorophenyl)-3-(cyclopropylmethoxy)-4-(difluoromethoxy)benzamide (**NPD-3080**)

^{1}H NMR (500 MHz, $CDCl_3$) δ 8.52 (dd, *J* = 8.3, 1.5 Hz, 1H), 8.40 (s, 1H), 7.59 (d, *J* = 2.0 Hz, 1H), 7.44 - 7.38 (m, 2H), 7.36 - 7.32 (m, 1H), 7.27 (d, *J* = 8.2 Hz, 1H), 7.10 (app td, *J* = 7.8, 1.5 Hz, 1H), 6.73 (app t, *J* = 74.9 Hz, 1H), 3.97 (d, *J* = 7.0 Hz, 2H), 1.37 - 1.29 (m, 1H), 0.71 - 0.65 (m, 2H), 0.42 - 0.36 (m, 2H). ^{13}C NMR (126 MHz, $CDCl_3$) δ 164.4, 151.0, 143.4, 134.6, 133.1, 129.2, 128.1, 125.1, 123.2, 122.5, 121.6, 118.9, 115.9 (t, *J* = 261.6 Hz), 114.0, 74.2, 10.2, 3.4. LC-MS: t_R=

5.08 min, purity: >99%, M/z $[M+H]^+$: 368; HRMS calcd. For $C_{18}H_{16}ClF_2NO3$ $[M+H]^+$= 368.0860, found 368.0851. Spectral data are in agreement with a previous report. [6]

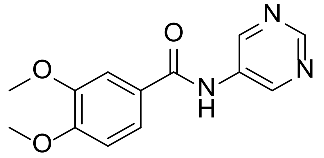

3,4-Dimethoxy-*N*-(pyrimidin-5-yl)benzamide (**NPD-3081**)

^{1}H NMR (500 MHz, $CDCl_3$) δ 9.11 (s, 2H), 9.00 (s, 1H), 8.01 (s, 1H), 7.50 (s, 1H), 7.44 (d, *J* = 8.3 Hz, 1H), 6.92 (d, *J* = 8.2 Hz, 1H), 3.95 (app s, 6H). ^{13}C NMR (126 MHz, $CDCl_3$) δ 165.7, 154.3, 152.9, 149.5, 148.2, 133.8, 125.9, 120.0, 110.9, 110.5, 56.3, 56.3. LC-MS: t_R= 2.82 min, purity: 97%, M/z $[M+H]^+$: 260; HRMS calcd. For $C_{13}H_{13}N_3O_3$ $[M+H]^+$= 260.1030, found 260.1024.

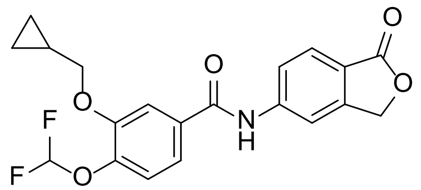

3-(Cyclopropylmethoxy)-4-(difluoromethoxy)-*N*-(1-oxo-1,3-dihydroisobenzofuran-5-yl)benzamide (**NPD-3083**)

^{1}H NMR (500 MHz, $CDCl_3$) δ 8.23 (s, 2H), 7.87 (d, *J* = 8.2 Hz, 1H), 7.55 (s, 1H), 7.47 (d, *J* = 8.2 Hz, 1H), 7.39 (d, *J* = 8.1 Hz, 1H), 7.29 - 7.24 (m, 1H), 6.73 (t, *J* = 74.8 Hz, 1H), 5.32 (s, 2H), 3.96 (d, *J* = 6.9 Hz, 2H), 1.32 (m, 1H), 0.68 (app q, *J* = 5.4 Hz, 2H), 0.38 (app q, *J* = 4.8 Hz, 2H). ^{13}C NMR (126 MHz, $CDCl_3$) δ 170.7, 165.0, 150.9, 148.6, 143.4 (t, *J* = 3.1 Hz), 143.2, 132.4, 126.7, 122.3, 121.3, 120.6, 119.0, 115.7 (t, *J* = 261.1 Hz), 113.8, 112.7, 74.2, 69.6, 10.0, 3.3. LC-MS: t_R= 4.55 min, purity: 98%, M/z $[M+H]^+$: 390; HRMS calcd. For $C_{20}H_{17}F_2NO_5$ $[M+H]^+$= 390.1148, found 390.1140.

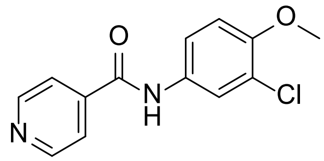

***N*-(3-chloro-4-methoxyphenyl)isonicotinamide** (**NPD-3085**)

^{1}H NMR (500 MHz, $CDCl_3$) δ 8.80 (d, *J* = 3.5 Hz, 2H), 7.83 (s, 1H), 7.70 (app s, 3H), 7.52 (d, *J* = 8.6 Hz, 1H), 6.94 (d, *J* = 8.7 Hz, 1H), 3.92 (s, 3H). ^{13}C NMR (126 MHz, $CDCl_3$) δ 163.7, 152.7, 151.0, 141.8, 130.7, 123.1, 122.9, 120.9, 120.3, 112.3, 56.5. LC-MS: t_R= 3.33 min, purity: >99%, M/z $[M+H]^+$: 263; HRMS calcd. For $C_{13}H_{11}ClN_2O_2$ $[M+H]^+$= 263.0582, found 263.0586.

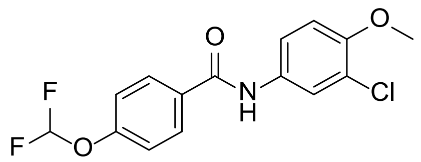

_N_-(3-Chloro-4-methoxyphenyl)-4-(difluoromethoxy)benzamide (**NPD-3086**)

^{1}H NMR (500 MHz, $CDCl_3$) δ 7.87 (d, *J* = 7.7 Hz, 2H), 7.76 (s, 1H), 7.67 (s, 1H), 7.49 (d, *J* = 8.8 Hz, 1H), 7.20 (d, *J* = 7.8 Hz, 2H), 6.91 (d, *J* = 8.8 Hz, 1H), 6.59 (t, *J* = 73.1 Hz, 1H), 3.90 (s, 3H). ^{13}C NMR (126 MHz, $CDCl_3$) δ 164.6, 153.8 (t, *J* = 2.7 Hz), 152.2, 131.5, 131.2, 129.0, 123.0, 122.6, 120.2, 119.3, 115.4 (t, *J* = 261.5 Hz), 112.2, 56.4. LC-MS: t_R= 4.57 min, purity: >99%, M/z [M+H]$^+$: 328; HRMS calcd. For $C_{15}H_{12}ClF_2NO_3$ [M+H]$^+$= 328.0547, found 328.0540.

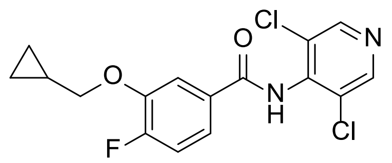

3-(Cyclopropylmethoxy)-_N_-(3,5-dichloropyridin-4-yl)-4-fluorobenzamide (**NPD-3093**)

^{1}H NMR (500 MHz, $CDCl_3$) δ 8.57 (s, 2H), 7.68 (s, 1H), 7.59 (dd, *J* = 8.0, 2.1 Hz, 1H), 7.46 (ddd, *J* = 8.3, 4.0, 2.2 Hz, 1H), 7.20 (dd, *J* = 10.5, 8.4 Hz, 1H), 3.96 (d, *J* = 7.0 Hz, 2H), 1.38 - 1.29 (m, 1H), 0.71 - 0.65 (m, 2H), 0.38 (app q, *J* = 4.8 Hz, 2H). ^{13}C NMR (126 MHz, $CDCl_3$) δ 163.8, 155.8 (d, *J* = 254.3 Hz), 148.5, 147.9 (d, *J* = 10.9 Hz), 139.9, 129.2 (d, *J* = 3.2 Hz), 128.9, 120.3 (d, *J* = 8.2 Hz), 116.5 (d, *J* = 19.5 Hz), 115.2 (d, *J* = 3.2 Hz), 74.6, 10.2, 3.5. LC-MS: t_R= 4.52 min, purity: 96%, M/z [M+H]$^+$: 355; HRMS calcd. For $C_{16}H_{13}Cl_2FN_2O_2$ [M+H]$^+$= 355.0411, found 355.0398.

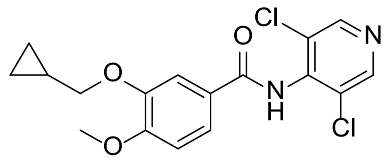

3-(Cyclopropylmethoxy)-_N_-(3,5-dichloropyridin-4-yl)-4-methoxybenzamide (**NPD-3094**)

^{1}H NMR (500 MHz, $CDCl_3$) δ 8.57 (s, 2H), 7.73 - 7.48 (m, 3H), 6.97 (d, *J* = 7.9 Hz, 1H), 4.05 - 3.85 (m, 5H), 1.45 - 1.33 (m, 1H), 0.69 (app d, *J* = 7.2 Hz, 2H), 0.47 - 0.31 (m, 2H). ^{13}C NMR (126 MHz, $CDCl_3$) δ 164.3, 153.6, 148.9, 148.4, 140.3, 128.7, 125.1, 121.0, 112.9, 110.8, 74.3, 56.3, 10.3, 3.6. LC-MS: t_R= 4.17 min, purity: 98%, M/z [M+H]$^+$: 367; HRMS calcd. For $C_{17}H_{16}Cl_2N_2O_3$ [M+H]$^+$= 367.0611, found 367.0593.

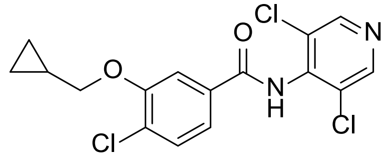

4-Chloro-3-(cyclopropylmethoxy)-_N_-(3,5-dichloropyridin-4-yl)benzamide (**NPD-3096**)

^{1}H NMR (500 MHz, $CDCl_3$) δ 8.58 (s, 2H), 7.69 (s, 1H), 7.56 - 7.48 (m, 2H), 7.40 (dd, *J* = 8.1, 1.6 Hz, 1H), 3.98 (d, *J* = 6.8 Hz, 2H), 1.39 - 1.30 (m, 1H), 0.68 (app q, *J* = 5.5 Hz, 2H), 0.41 (app q, *J* = 5.1 Hz, 2H). ^{13}C NMR (126 MHz, $CDCl_3$) δ 163.9, 155.3, 148.5, 139.8, 132.3, 130.7, 129.0, 128.6, 119.7,113.3, 74.3, 10.1, 3.5. LC-MS: t_R= 4.81 min, purity: >99%, M/z $[M+H]^+$: 371; HRMS calcd. For $C_{16}H_{13}Cl_3N_2O_2$ $[M+H]^+$= 371.0115, found 371.0101.

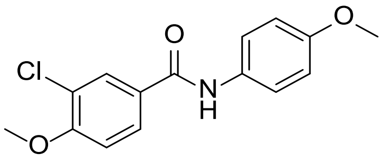

3-Chloro-4-methoxy-*N*-(4-methoxyphenyl)benzamide (**NPD-3286**)

^{1}H NMR (600 MHz, $CDCl_3$) δ 7.89 (d, *J* = 2.1 Hz, 1H), 7.78 (dd, *J* = 8.6, 2.2 Hz, 1H), 7.63 (s, 1H), 7.51 (d, *J* = 8.7 Hz, 2H), 6.99 (d, *J* = 8.5 Hz, 1H), 6.91 (d, *J* = 8.9 Hz, 2H), 3.97 (s, 3H), 3.82 (s, 3H). ^{13}C NMR (151 MHz, $CDCl_3$) δ 164.0, 157.7, 156.7, 130.8, 129.1, 128.0, 127.2, 122.8, 122.2, 114.3, 111.6, 56.4, 55.5. LC-MS: t_R= 4.25 min, purity: >99%, M/z $[M+H]^+$: 292; HRMS calcd. For $C_{15}H_{14}ClNO_3$ $[M+H]^+$= 292.0735, found 292.0729.

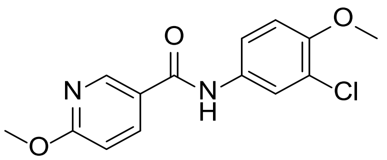

***N*-(3-Chloro-4-methoxyphenyl)-6-methoxynicotinamide** (**NPD-3287**)

^{1}H NMR (600 MHz, $CDCl_3$) δ 8.70 (s, 1H), 8.09 (d, *J* = 8.3 Hz, 1H), 7.70 (s, 1H), 7.64 (s, 1H), 7.51 (d, *J* = 7.9 Hz, 1H), 6.95 (d, *J* = 8.7 Hz, 1H), 6.86 (d, *J* = 8.5 Hz, 1H), 4.03 (s, 3H), 3.93 (s, 3H). ^{13}C NMR (151 MHz, $CDCl_3$) δ 166.4, 163.9, 152.4, 146.5, 138.0, 131.3, 123.9, 123.2, 122.8, 120.3, 112.4, 111.3, 56.6, 54.3. LC-MS: t_R= 4.06 min, purity: 99%, M/z $[M+H]^+$: 293; HRMS calcd. For $C_{14}H_{13}ClN_2O_3$ $[M+H]^+$= 293.0687, found 293.0676.

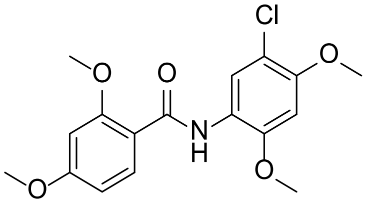

***N*-(5-Chloro-2,4-dimethoxyphenyl)-2,4-dimethoxybenzamide** (**NPD-3288**)

^{1}H NMR (300 MHz, $CDCl_3$) δ 9.87 (s, 1H), 7.98 (d, *J* = 9.0 Hz, 1H), 7.72 (d, *J* = 2.5 Hz, 1H), 7.58 (dd, *J* = 8.9, 2.5 Hz, 1H), 6.92 (d, *J* = 8.9 Hz, 1H), 6.83 (d, *J* = 9.0 Hz, 1H), 4.07 (s, 3H), 3.93 (s, 3H), 3.92 - 3.88 (m, 6H). ^{13}C NMR (151 MHz, $CDCl_3$) δ 162.8, 157.0, 152.3, 151.8, 141.9, 132.4, 127.1, 122.7, 122.6, 119.8, 118.8, 112.5, 108.0, 62.1, 61.2, 56.6, 56.3. LC-MS: t_R= 4.61 min, purity: >99%, M/z $[M+H]^+$: 352; HRMS calcd. For $C_{17}H_{18}ClNO_5$ $[M+H]^+$= 352.0946, found 352.0945.

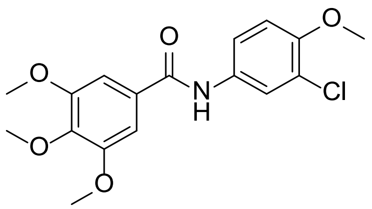

***N*-(3-Chloro-4-methoxyphenyl)-3,4,5-trimethoxybenzamide** (**NPD-3289**)

^{1}H NMR (600 MHz, $CDCl_3$) δ 7.68 (s, 1H), 7.66 (d, *J* = 2.5 Hz, 1H), 7.53 (dd, *J* = 8.8, 2.5 Hz, 1H), 7.06 (s, 2H), 6.93 (d, *J* = 8.9 Hz, 1H), 3.92 (app s, 6H), 3.91 (s, 3H), 3.90 (s, 3H). ^{13}C NMR (151 MHz, $CDCl_3$) δ 165.5, 153.5, 152.3, 141.5, 131.5, 130.2, 123.0, 122.8, 120.2, 112.4, 104.6, 61.1, 56.6. Note: Carbon signals of the two non-equivalent methoxy groups overlap. LC-MS: t_R= 4.23 min, purity: >99%, M/z $[M+H]^+$: 352; HRMS calcd. For $C_{17}H_{18}ClNO_5$ $[M+H]^+$= 352.0946, found 352.0940.

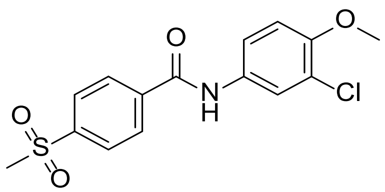

***N*-(3-Chloro-4-methoxyphenyl)-4-(methylsulfonyl)benzamide** (**NPD-3290**)

^{1}H NMR (300 MHz, $CDCl_3$ + DMSO-d_6) δ 10.16 (s, 1H), 8.14 (d, *J* = 8.4 Hz, 2H), 7.96 (d, *J* = 8.4 Hz, 2H), 7.83 (d, *J* = 2.4 Hz, 1H), 7.62 - 7.57 (m, 1H), 6.89 (d, *J* = 8.9 Hz, 1H), 3.82 (s, 3H), 3.07 (s, 3H). ^{13}C NMR (151 MHz, $CDCl_3$ + DMSO-d_6) δ 163.2, 150.6, 142.0, 138.8, 131.4, 127.9, 126.3, 122.0, 120.7, 119.5, 111.2, 55.3, 43.3. LC-MS: t_R= 3.83 min, purity: 97%, M/z $[M+H]^+$: 340; HRMS calcd. For $C_{15}H_{14}ClNO_4S$ $[M+H]^+$= 340.0405, found 340.0400.

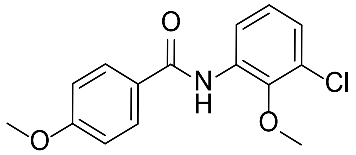

***N*-(3-Chloro-2-methoxyphenyl)-4-methoxybenzamide** (**NPD-3291**)

^{1}H NMR (300 MHz, $CDCl_3$) δ 8.48 (s, 1H), 8.43 (dd, *J* = 5.8, 4.0 Hz, 1H), 7.86 (d, *J* = 8.8 Hz, 2H), 7.11 (s, 1H), 7.10 (d, *J* = 1.9 Hz, 1H), 7.01 (d, *J* = 8.8 Hz, 2H), 3.95 (s, 3H), 3.89 (s, 3H). ^{13}C NMR (151 MHz, $CDCl_3$) δ 165.0, 162.9, 145.2, 133.5, 129.1, 127.0, 126.9, 125.5, 125.0, 119.0, 114.3, 61.1, 55.7. LC-MS: t_R= 4.65 min, purity: >99%, M/z $[M+H]^+$: 292; HRMS calcd. For $C_{15}H_{14}ClNO_3$ $[M+H]^+$= 292.0735, found 292.0726.

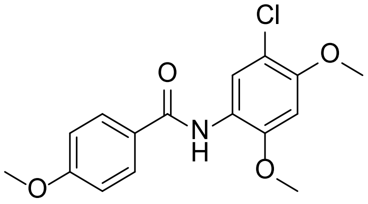

***N*-(5-Chloro-2,4-dimethoxyphenyl)-4-methoxybenzamide** (**NPD-3292**)

^{1}H NMR (300 MHz, $CDCl_3$) δ 8.60 (s, 1H), 8.25 (s, 1H), 7.86 (d, *J* = 8.8 Hz, 2H), 7.01 (d, *J* = 8.8 Hz, 2H), 6.58 (s, 1H), 3.97 (s, 3H), 3.93 (s, 3H), 3.90 (s, 3H). ^{13}C NMR (151 MHz, $CDCl_3$) δ 164.6, 162.6, 151.4, 147.9, 129.0, 127.4, 122.0, 121.7, 114.14, 114.11, 96.8, 56.9, 56.4, 55.6. LC-MS: t_R= 4.46 min, purity: >99%, M/z [M+H]$^+$: 322; HRMS calcd. For $C_{16}H_{16}ClNO_4$ [M+H]$^+$= 322.0841, found 322.0840.

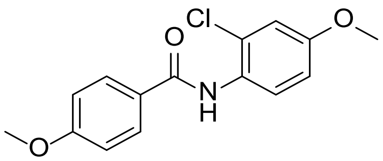

***N*-(2-Chloro-4-methoxyphenyl)-4-methoxybenzamide** (**NPD-3293**)

^{1}H NMR (300 MHz, $CDCl_3$) δ 8.38 (d, *J* = 9.1 Hz, 1H), 8.14 (s, 1H), 7.88 (d, *J* = 8.8 Hz, 2H), 7.04 - 6.94 (m, 3H), 6.88 (dd, *J* = 9.1, 2.8 Hz, 1H), 3.88 (s, 3H), 3.81 (s, 3H). ^{13}C NMR (151 MHz, $CDCl_3$) δ 164.8, 162.8, 156.3, 129.1, 128.4, 127.1, 124.3, 123.0, 114.7, 114.2, 113.4, 55.9, 55.6. LC-MS: t_R= 4.26 min, purity: >99%, M/z [M+H]$^+$: 292; HRMS calcd. For $C_{15}H_{14}ClNO_3$ [M+H]$^+$= 292.0735, found 292.0728.

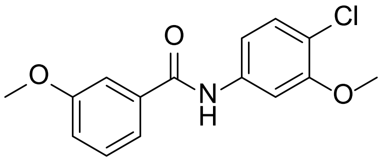

***N*-(4-Chloro-3-methoxyphenyl)-3-methoxybenzamide** (**NPD-3294**)

^{1}H NMR (300 MHz, $CDCl_3$) δ 7.81 (s, 1H), 7.72 (d, *J* = 2.2 Hz, 1H), 7.43 (s, 1H), 7.39 (app d, *J* = 7.2 Hz, 2H), 7.32 (d, *J* = 8.5 Hz, 1H), 7.10 (m, 1H), 6.90 (dd, *J* = 8.5, 2.3 Hz, 1H), 3.95 (s, 3H), 3.88 (s, 3H). ^{13}C NMR (151 MHz, $CDCl_3$) δ 165.7, 160.2, 155.5, 137.9, 136.2, 130.2, 130.0, 118.7, 118.4, 117.9, 112.6, 112.3, 104.7, 56.4, 55.7. LC-MS: t_R= 4.46 min, purity: >99%, M/z [M+H]$^+$: 292; HRMS calcd. For $C_{15}H_{14}ClNO_3$ [M+H]$^+$= 292.0735, found 292.0725.

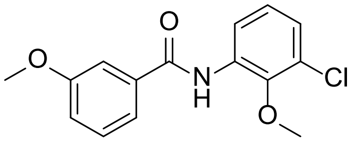

***N*-(3-Chloro-2-methoxyphenyl)-3-methoxybenzamide** (**NPD-3295**)

^{1}H NMR (300 MHz, $CDCl_3$) δ 8.38 (d, *J* = 9.1 Hz, 1H), 8.21 (s, 1H), 7.51 - 7.45 (m, 1H), 7.42 (app d, *J* = 6.6 Hz, 2H), 7.10 (m, 1H), 6.98 (d, *J* = 2.8 Hz, 1H), 6.89 (dd, *J* = 9.1, 2.8 Hz, 1H), 3.89 (s, 3H), 3.81 (s, 3H). ^{13}C NMR (151 MHz, $CDCl_3$) δ 165.2, 160.2, 156.5, 136.4, 130.0, 128.2, 124.4, 123.1, 118.8, 118.3, 114.7, 113.5, 112.7, 55.9, 55.6. LC-MS: t_R= 4.33 min, purity: >99%, M/z $[M+H]^+$: 292; HRMS calcd. For $C_{15}H_{14}ClNO_3$ $[M+H]^+$= 292.0735, found 292.0732.

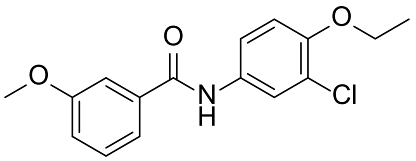

***N*-(3-Chloro-4-ethoxyphenyl)-3-methoxybenzamide** (**NPD-3296**)

^{1}H NMR (300 MHz, $CDCl_3$) δ 7.71 (s, 1H), 7.68 (d, *J* = 2.6 Hz, 1H), 7.49 (dd, *J* = 8.9, 2.6 Hz, 1H), 7.44 - 7.40 (m, 1H), 7.40 - 7.33 (m, 2H), 7.08 (m, 1H), 6.92 (d, *J* = 8.9 Hz, 1H), 4.11 (q, *J* = 7.0 Hz, 2H), 3.87 (s, 3H), 1.47 (t, *J* = 7.0 Hz, 3H). ^{13}C NMR (151 MHz, $CDCl_3$) δ 165.6, 160.1, 151.7, 136.3, 131.5, 129.9, 123.3, 122.9, 120.1, 118.7, 118.2, 113.9, 112.6, 65.3, 55.6, 14.9. LC-MS: t_R= 4.61 min, purity: >99%, M/z $[M+H]^+$: 306; HRMS calcd. For $C_{16}H_{16}ClNO_3$ $[M+H]^+$= 306.0891, found 306.0881.

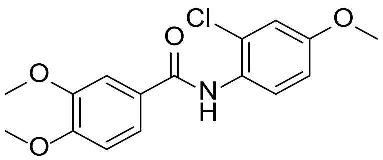

***N*-(2-Chloro-4-methoxyphenyl)-3,4-dimethoxybenzamide** (**NPD-3297**)

^{1}H NMR (300 MHz, $CDCl_3$) δ 8.59 (s, 1H), 8.29 (s, 1H), 7.45 (s, 1H), 7.42 - 7.34 (m, 2H), 7.15 - 7.02 (m, 1H), 6.56 (s, 1H), 3.94 (s, 3H), 3.91 (s, 3H), 3.88 (s, 3H). ^{13}C NMR (151 MHz, $CDCl_3$) δ 164.9, 160.1, 151.6, 148.0, 136.6, 129.9, 121.8, 121.7, 118.8, 118.0, 114.1, 112.7, 96.7, 56.9, 56.4, 55.6. LC-MS: t_R= 4.53 min, purity: >99%, M/z $[M+H]^+$: 322; HRMS calcd. For $C_{16}H_{16}ClNO_4$ $[M+H]^+$= 322.0841, found 322.0835.

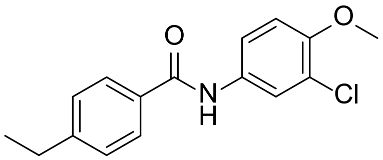

***N*-(3-Chloro-4-methoxyphenyl)-4-ethylbenzamide** (**NPD-3298**)

^{1}H NMR (300 MHz, $CDCl_3$) δ 7.77 (d, *J* = 8.1 Hz, 2H), 7.69 (d, *J* = 2.4 Hz, 2H), 7.52 (dd, *J* = 8.9, 2.5 Hz, 1H), 7.31 (d, *J* = 8.1 Hz, 2H), 6.92 (d, *J* = 8.9 Hz, 1H), 3.90 (s, 3H), 2.72 (q, *J* = 7.6 Hz, 2H), 1.27 (t, *J* = 7.6 Hz, 3H). ^{13}C NMR (151 MHz, $CDCl_3$) δ 165.7, 152.2, 148.9, 132.1, 131.7, 128.4, 127.2, 122.9, 122.7, 120.1, 112.4, 56.6, 29.0, 15.4. LC-MS: t_R= 4.74 min, purity: >99%, M/z $[M+H]^+$: 290; HRMS calcd. For $C_{16}H_{16}ClNO_2$ $[M+H]^+$= 290.0942, found 290.0933.

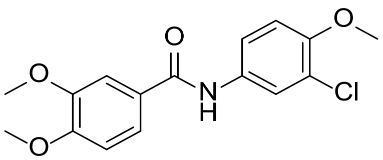

***N*-(3-Chloro-4-methoxyphenyl)-3,4-dimethoxybenzamide** (**NPD-3299**)

^{1}H NMR (600 MHz, $CDCl_3$) δ 7.70 (s, 1H), 7.67 (d, *J* = 2.5 Hz, 1H), 7.52 (dd, *J* = 8.8, 2.5 Hz, 1H), 7.48 (d, *J* = 1.8 Hz, 1H), 7.37 (dd, *J* = 8.3, 1.8 Hz, 1H), 6.94 - 6.89 (m, 2H), 3.95 (s, 6H), 3.90 (s, 3H). ^{13}C NMR (151 MHz, $CDCl_3$) δ 165.3, 152.3, 152.1, 149.4, 131.7, 127.3, 123.0, 122.7, 120.1, 119.5, 112.4, 110.8, 110.5, 56.6, 56.2. Note: Two methoxy group carbon signals overlap. LC-MS: t_R= 4.07 min, purity: >99%, M/z $[M+H]^+$: 322; HRMS calcd. For $C_{16}H_{16}ClNO_4$ $[M+H]^+$= 322.0841, found 322.0841.

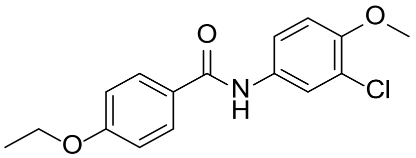

***N*-(3-Chloro-4-methoxyphenyl)-4-ethoxybenzamide** (**NPD-3300**)

^{1}H NMR (300 MHz, $CDCl_3$) δ 7.81 (d, *J* = 8.8 Hz, 2H), 7.70 - 7.63 (m, 2H), 7.50 (dd, *J* = 8.9, 2.5 Hz, 1H), 6.98 - 6.89 (m, 3H), 4.10 (q, *J* = 7.0 Hz, 2H), 3.90 (s, 3H), 1.45 (t, *J* = 7.0 Hz, 3H). ^{13}C NMR (151 MHz, $CDCl_3$) δ 165.3, 162.1, 152.1, 131.8, 129.0, 126.7, 122.9, 122.7, 120.1, 114.6, 112.4, 63.9, 56.6, 14.8. LC-MS: t_R= 4.52 min, purity: >99%, M/z $[M+H]^+$: 306; HRMS calcd. For $C_{16}H_{16}ClNO_3$ $[M+H]^+$= 306.0891, found 306.0884.

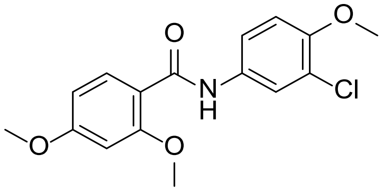

***N*-(3-Chloro-4-methoxyphenyl)-2,4-dimethoxybenzamide** (**NPD-3301**)

^{1}H NMR (300 MHz, $CDCl_3$) δ 9.59 (s, 1H), 8.24 (d, *J* = 8.8 Hz, 1H), 7.67 (d, *J* = 2.5 Hz, 1H), 7.60 (d, *J* = 2.4 Hz, 1H), 6.91 (d, *J* = 2.4 Hz 1H), 6.65 (dd, *J* = 8.8, 2.3 Hz, 1H), 6.53 (d, *J* = 2.1 Hz, 1H), 4.03 (s, 3H), 3.90 (s, 3H), 3.88 (s, 3H). ^{13}C NMR (151 MHz, $CDCl_3$) δ 164.0, 163.2, 158.7, 151.7, 134.4, 132.4, 122.9, 122.5, 120.1, 114.6, 112.4, 105.9, 98.9, 56.6, 56.4, 55.8. LC-MS: t_R= 4.59 min, purity: >99%, M/z $[M+H]^+$: 322; HRMS calcd. For $C_{16}H_{16}ClNO_4$ $[M+H]^+$= 322.0841, found 322.0838.

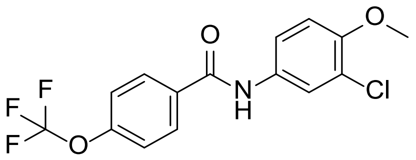

***N*-(3-Chloro-4-methoxyphenyl)-4-(trifluoromethoxy)benzamide** (**NPD-3302**)

^{1}H NMR (300 MHz, $CDCl_3$) δ 7.89 (d, *J* = 8.7 Hz, 2H), 7.74 (s, 1H), 7.67 (d, *J* = 2.4 Hz, 1H), 7.49 (dd, *J* = 8.8, 2.4 Hz, 1H), 7.31 (d, *J* = 8.4 Hz, 2H), 6.92 (d, *J* = 8.9 Hz, 1H), 3.90 (s, 3H). ^{13}C NMR (151 MHz, $CDCl_3$) δ 164.3, 152.4, 151.8, 133.1, 131.1, 128.9, 123.0, 122.7, 120.9, 120.1, 119.5 (q, *J* = 260.4 Hz), 112.3, 56.4. LC-MS: t_R= 4.86 min, purity: >99%, M/z [M+H]$^+$: 346; HRMS calcd. For $C_{15}H_{11}ClF_3NO_3$ [M+H]$^+$= 346.0452, found 346.0456.

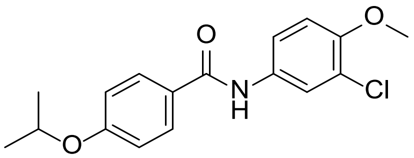

***N*-(3-Chloro-4-methoxyphenyl)-4-isopropoxybenzamide** (**NPD-3303**)

^{1}H NMR (300 MHz, $CDCl_3$) δ 7.80 (d, *J* = 8.8 Hz, 2H), 7.68 (d, *J* = 2.5 Hz, 1H), 7.63 (s, 1H), 7.50 (dd, *J* = 8.8, 2.5 Hz, 1H), 6.98 - 6.89 (m, 3H), 4.64 (hept, *J* = 6.1 Hz, 1H), 3.90 (s, 3H), 1.37 (d, *J* = 6.1 Hz, 6H). ^{13}C NMR (151 MHz, $CDCl_3$) δ 165.3, 161.2, 152.1, 131.8, 129.0, 126.4, 122.9, 122.7, 120.1, 115.6, 112.4, 70.3, 56.6, 22.1. LC-MS: t_R= 4.72 min, purity: >99%, M/z [M+H]$^+$: 320; HRMS calcd. For $C_{17}H_{18}ClNO_3$ [M+H]$^+$= 320.1048, found 320.1036.

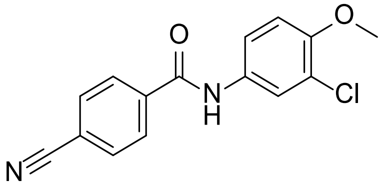

***N*-(3-Chloro-4-methoxyphenyl)-4-cyanobenzamide** (**NPD-3304**)

^{1}H NMR (300 MHz, $CDCl_3$) δ 7.96 (d, *J* = 8.3 Hz, 2H), 7.79 (d, *J* = 8.4 Hz, 2H), 7.75 (s, 1H), 7.68 (d, *J* = 2.3 Hz, 1H), 7.50 (dd, *J* = 8.8, 2.2 Hz, 1H), 6.94 (d, *J* = 8.9 Hz, 1H), 3.91 (s, 3H). ^{13}C NMR (126 MHz, DMSO-d_6) δ 163.9, 151.1, 138.7, 132.5, 132.4, 128.5, 122.0, 120.5, 120.3, 118.4, 113.9, 112.8, 56.2. LC-MS: t_R= 4.20 min, purity: >99%, M/z [M+H]$^+$: 287; HRMS calcd. For $C_{15}H_{11}ClN_2O_2$ [M+H]$^+$= 287.0582, found 287.0570.

Table S1. Overview of the PDE4NPD toolbox and inhibitor selectivity for human (h) or parasite PDEs

Tool	Compound ID	Name	PDE selectivity	Literature
**NPD-0013**	VUF14752	IBMX	non-selective	[7]
**NPD-0014**	VUF14753	Caffeine	non-selective	[8]
**NPD-0015**	VUF14754	Dipyradamole	non-selective	[9]
**NPD-0009**	VUF14748	Vinpocetine	hPDE1	[10]
**NPD-0031**	VUF14680		hPDE1	[11]
**NPD-0017**	VUF14759	BAY 60-7550	hPDE2	[12]
**NPD-0010**	VUF14749	Trequinsin	hPDE3	[13]
**NPD-0011**	VUF14750	Amrinone	hPDE3	[14]
**NPD-0018**	VUF14760	Milrinone	hPDE3	[12]
**NPD-0027**	VUF14810	Cilostamide	hPDE3	[15]
**NPD-0005**	VUF14664	piclamilast	hPDE4	[16]
**NPD-0006**	VUF14665	roflumilast	hPDE4	[12]
**NPD-0007**	VUF14672		hPDE4	[17]
**NPD-0034**	VUF14910	etazolate	hPDE4	[18]
**NPD-0072**	VUF14761	Rolipram	hPDE4	[12]
**NPD-0029**	VUF14834		hPDE4/7	[19]
**NPD-0001**	VUF14656	PPS54019	hPDE4/TbrPDEB1	[20]
**NPD-0340**	VUF13524		hPDE4/TbrPDEB1	[21]
**NPD-0339**	VUF11842		hPDE4/TbrPDEB1	[12]
**NPD-0002**	VUF14661		hPDE5	[22]
**NPD-0003**	VUF14662	Sildenafil	hPDE5	[12]
**NPD-0004**	VUF14789		hPDE5	[23]
**NPD-0022**	VUF14786	Tadalafil	hPDE5	[12]
**NPD-0030**	VUF14837	Vardenafil	hPDE5	[12]
**NPD-0025**	VUF14808	Zaprinast	hPDE5/PDE6	[24]
**NPD-0020**	VUF14782	BRL-50481	hPDE7	[25]
**NPD-0078**	MR 1.51		hPDE7	[26]
**NPD-0082**	AGF2.20		hPDE7	[27]
**NPD-1091**	WP5.11b		hPDE8	[28]
**NPD-1092**	WP5.19R		hPDE8	[28]
**NPD-1100**	VSP2.34		hPDE8	[28]
**NPD-0012**	VUF14751	BAY73-6691	hPDE9	[12]
**NPD-0019**	VUF14763	BIPPO	PfPDEalpha	[29]
**NPD-0024**	VUF14792		PfPDEalpha	[29]
**NPD-0008**	VUF14680	PPS58083	TbrPDEB1	[30]
**NPD-0016**	VUF14755	Papaverine	hPDE10	[12]
**NPD-0026**	VUF14809	TC-E 5005	hPDE10	[31]

**NPD-0028**	VUF14811	PF-2545920	hPDE11	[32]

Table S2. SmPDE4A inhibition of roflumilast analogues

Comp	SmPDE4A pK_i[a] or inhibition at 10 μM	Comp	SmPDE4A pK_i[a] or inhibition at 10 μM	Comp	SmPDE4A pK_i[a] or inhibition at 10 μM
**NPD-1177**	44%	**NPD-1201**	1%	**NPD-3083**	3%
**NPD-1178**	48%	**NPD-1202**	0%	**NPD-3085**	1%
**NPD-1179**	50%	**NPD-1203**	10%	**NPD-3086**	41%
**NPD-1181**	22%	**NPD-1206**	14%	**NPD-3093**	5.6 ± 0.0[a]
**NPD-1182**	22%	**NPD-1472**	5%	**NPD-3286**	7%
**NPD-1183**	15%	**NPD-2976**	46%	**NPD-3287**	20%
**NPD-1184**	22%	**NPD-2981**	6.4 ± 0.1	**NPD-3288**	0%
**NPD-1185**	18%	**NPD-2991**	5.2 ± 0.2[a]	**NPD-3289**	0%
**NPD-1186**	19%	**NPD-2992**	4%	**NPD-3290**	0%
**NPD-1187**	20%	**NPD-2993**	24%	**NPD-3291**	0%
**NPD-1188**	13%	**NPD-2994**	7%	**NPD-3292**	0%
**NPD-1189**	8%	**NPD-2995**	3%	**NPD-3293**	2%
**NPD-1190**	9%	**NPD-2996**	21%	**NPD-3294**	0%
**NPD-1191**	8%	**NPD-2997**	27%	**NPD-3295**	8%
**NPD-1192**	6%	**NPD-2998**	11%	**NPD-3296**	6%
**NPD-1193**	7%	**NPD-2999**	26%	**NPD-3297**	0%
**NPD-1194**	0%	**NPD-3001**	15%	**NPD-3298**	6.1 ± 0.0[a]
**NPD-1195**	0%	**NPD-3002**	21%	**NPD-3299**	0%
**NPD-1196**	1%	**NPD-3003**	4%	**NPD-3300**	5.8 ± 0.0[a]
**NPD-1197**	10%	**NPD-3004**	5.0 ± 0.0[a]	**NPD-3301**	16%
**NPD-1198**	6%	**NPD-3005**	18%	**NPD-3302**	42%
**NPD-1199**	28%	**NPD-3006**	16%	**NPD-3303**	27%
**NPD-1200**	0%	**NPD-3007**	34%	**NPD-3304**	18%

[a] Partial curve

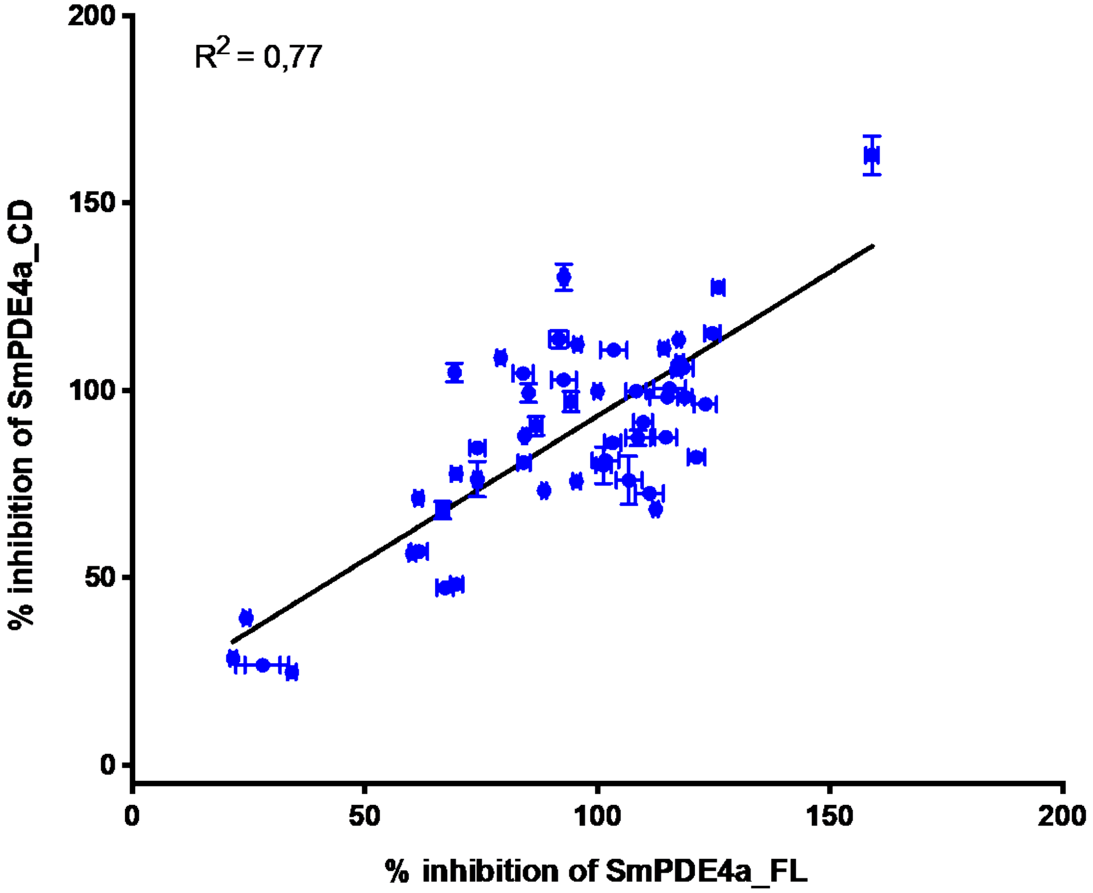

Fig S1. Correlation plot of the inhibition of the PDE Toolbox compounds of the enzymatic activities of purified SmPDE4A_CD (catalytic domain) and SmPDE4A_FL (full length).

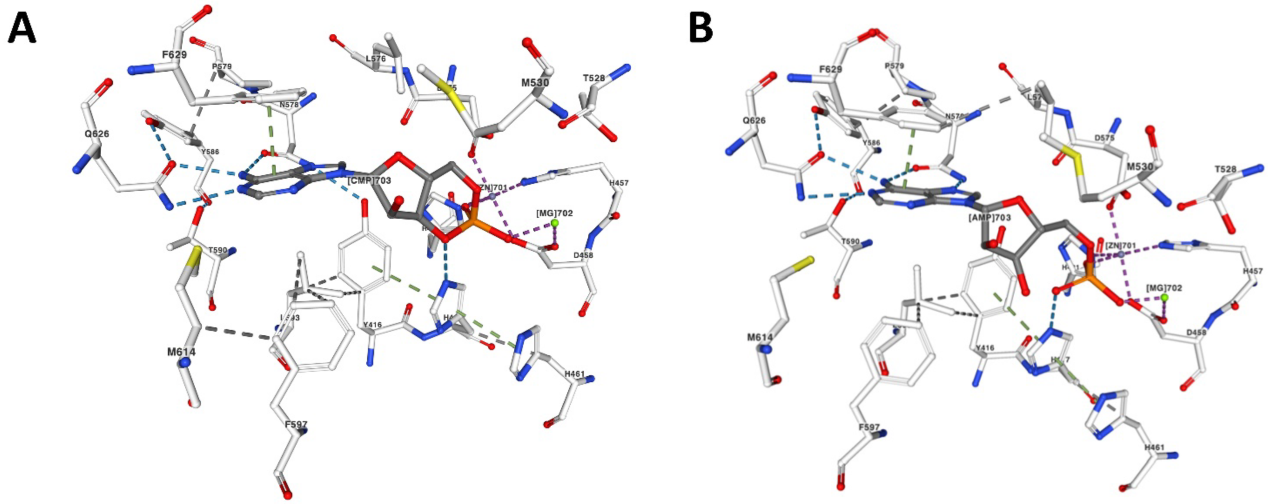

Fig S2. Binding of the substrate cAMP (A) and its hydrolyzed product AMP (B) in the binding pockets of SmPDE4A_CD, as observed in the chain A and chain B of the X-ray structure 6FG5. Binding pocket residues are visualized with NGL Viewer [33] **implemented at the RCSB PDB website.**

Crystallographic data

The coordinates of the 2 obtained x-ray structures of SmPDE4A_CD can be obtained from the Protein Data Bank with the IDs 6FG5 (apo structure) and 6EZU (co-crystal with cAMP and AMP). Full validation reports for both structures can be found at https://files.rcsb.org/pub/pdb/validation_reports/fg/6fg5/6fg5_full_validation.pdf for 6FG5 and at https://files.rcsb.org/pub/pdb/validation_reports/ez/6ezu/6ezu_full_validation.pdf for 6EZU. For both structures the data and refinement statistics are reported in the tables below.

6FG5:

Property	Value	Source
Space group	P 31 2 1	Depositor
Cell constants<br>a, b, c, α, β, γ	81.80Å 81.80Å 256.13Å<br>90.00° 90.00° 120.00°	Depositor
Resolution (Å)	47.50 – 2.35<br>47.50 – 2.35	Depositor<br>EDS
% Data completeness<br>(in resolution range)	100.0 (47.50-2.35)<br>96.1 (47.50-2.35)	Depositor<br>EDS
R_{merge}	0.07	Depositor
R_{sym}	(Not available)	Depositor
$< I/\sigma(I) >$ [1]	1.67 (at 2.34Å)	Xtriage
Refinement program	REFMAC 5.8.0189	Depositor
R, R_{free}	0.203 , 0.245<br>0.205 , (Not available)	Depositor<br>DCC
R_{free} test set	No test flags present.	wwPDB-VP
Wilson B-factor ($Å^2$)	50.1	Xtriage
Anisotropy	0.236	Xtriage
Bulk solvent $k_{sol}(e/Å^3)$, $B_{sol}(Å^2)$	0.31 , 26.2	EDS
L-test for twinning[2]	$< \|L\| > = 0.49$, $< L^2 > = 0.33$	Xtriage
Estimated twinning fraction	0.026 for -h,-k,l	Xtriage
F_o,F_c correlation	0.95	EDS
Total number of atoms	5411	wwPDB-VP
Average B, all atoms ($Å^2$)	57.0	wwPDB-VP

6EZU:

Property	Value	Source
Space group	P 31 2 1	Depositor
Cell constants<br>a, b, c, α, β, γ	81.55Å 81.55Å 255.87Å<br>90.00° 90.00° 120.00°	Depositor
Resolution (Å)	85.44 – 2.04<br>47.41 – 2.04	Depositor<br>EDS
% Data completeness<br>(in resolution range)	99.9 (85.44-2.04)<br>100.0 (47.41-2.04)	Depositor<br>EDS
R_{merge}	0.07	Depositor
R_{sym}	(Not available)	Depositor
$< I/\sigma(I) >$ [1]	1.84 (at 2.05Å)	Xtriage
Refinement program	REFMAC 5.8.0189	Depositor
R, R_{free}	0.199 , 0.233<br>0.205 , 0.236	Depositor<br>DCC
R_{free} test set	3042 reflections (4.76%)	wwPDB-VP
Wilson B-factor (Å^2)	49.4	Xtriage
Anisotropy	0.254	Xtriage
Bulk solvent k_{sol}(e/Å^3), B_{sol}(Å^2)	0.32 , 44.8	EDS
L-test for twinning[2]	$< \|L\| > = 0.50$, $< L^2 > = 0.34$	Xtriage
Estimated twinning fraction	0.022 for -h,-k,l	Xtriage
F_o,F_c correlation	0.96	EDS
Total number of atoms	5340	wwPDB-VP
Average B, all atoms (Å^2)	61.0	wwPDB-VP

Medicinal Chemistry
Vrije Universiteit Amsterdam

Acquired by : Admin
Date Acquired : 25/4/2019 9:19:48 AM
Sample Name : NPD-0005
Sample ID :
Tray# : 1
Vial# : 3
Injection Volume : 2
Data File : C:\LabSolutions\Data\2019\2019-wk17\NPD-0005.lcd
Background File : blanco 25042019.lcd
Method File : Method SCAN ACID standard.lcm
Report Format : DefaultLCMS.lcr
Tuning File : C:\LabSolutions\Tuning\Tuning-ESI-pos-neg01072015a.lct
Processed by : Admin
Modified Date : 25/4/2019 10:14:22 AM

PDA Graph

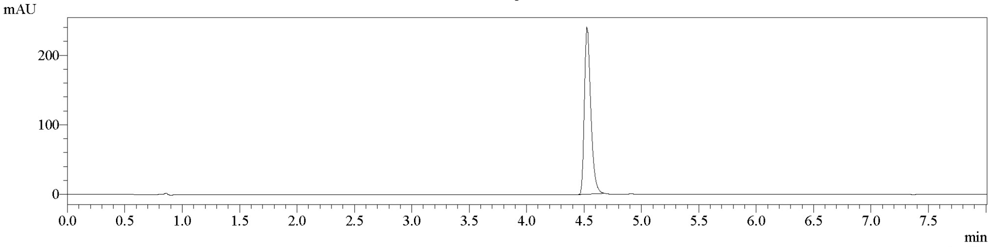

PeakTable

PDA Ch1 254nm 4nm

Peak#	Name	Ret. Time	Area	Area %
1		4.521	924888	100.000

MS Chromatogram
NPD-0005 C:\LabSolutions\Data\2019\2019-wk17\NPD-0005.lcd

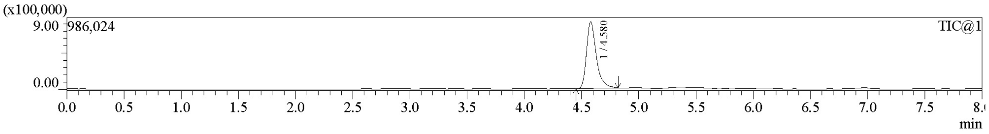

MS Spectrum Graph

#:1 Ret.Time:Averaged 4.570-4.590(Scan#:458-460)
BG Mode:Calc 4.450<->4.820(446<->483)
Mass Peaks:14 Base Peak:381.05(373123) Polarity:Pos Segment1 - Event1

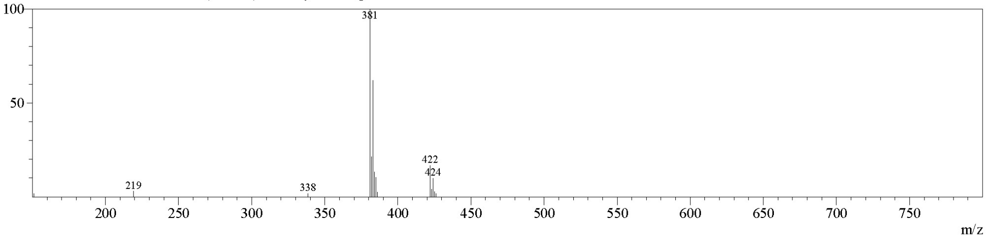

MS Spectrum Table

#:1 Ret.Time:
BG Mode:Calc 4.450<->4.820(446<->483)
Mass Peaks:14 Base Peak:381.05(373123) Polarity:Pos Segment1 - Event1

#	m/z	Abs.Inten.	Rel.Inten.	Charge	Polarity	Monoisotopic
1	150.90	6333	1.70			
2	219.10	10740	2.88			
3	338.30	6050	1.62			
4	381.05	373123	100.00			
5	382.00	79416	21.28			
6	383.05	231440	62.03			
7	384.05	49370	13.23			

#	m/z	Abs.Inten.	Rel.Inten.	Charge	Polarity	Monoisotopic
8	385.05	38470	10.31			
9	386.15	8921	2.39			
10	422.10	61998	16.62			
11	423.05	14621	3.92			
12	424.10	36514	9.79			
13	425.15	9774	2.62			
14	426.05	6295	1.69			

Figure S3 LCMS spectrum of compound NPD-0005

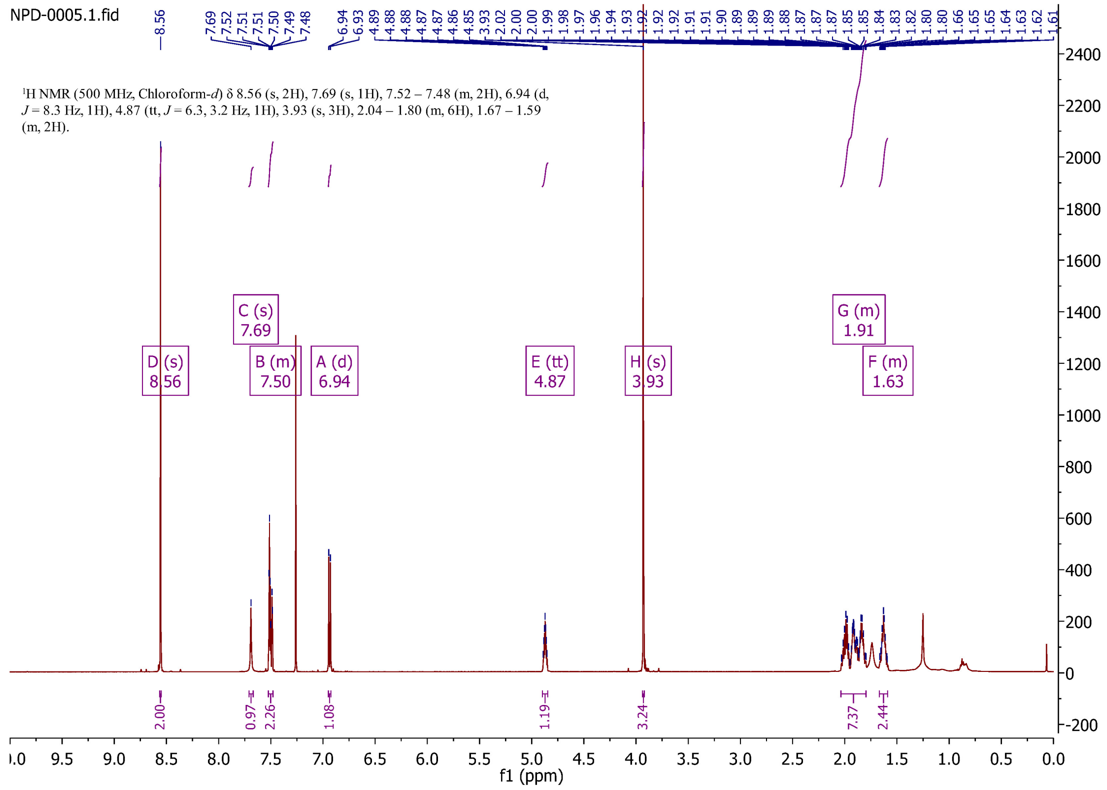

Figure S4 ^{1}H NMR spectrum of compound NPD-0005

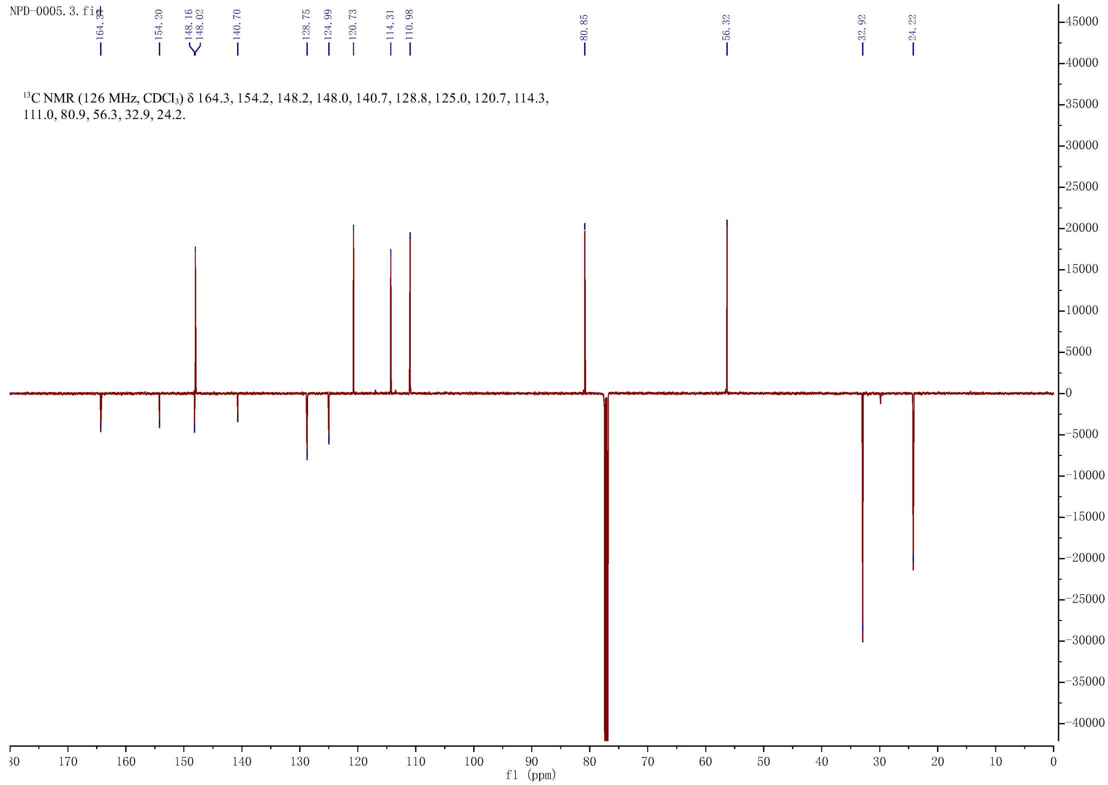

Figure S5 ^{13}C NMR spectrum of compound NPD-0005

Acquired by	: Admin
Date Acquired	: 1/24/2017 1:30:09 PM
Sample Name	: YAZH01-022
Sample ID	:
Tray#	: 1
Vial#	: 17
Injection Volume	: 1
Data File	: C:\LabSolutions\Data\2017\2017-wk04\YAZH01-022.lcd
Background File	: Blanco24012017.lcd
Method File	: Method SCAN ACID standard.lcm
Report Format	: DefaultLCMS.lcr
Tuning File	: C:\LabSolutions\Tuning\Tuning-ESI-pos-neg01072015a.lct
Processed by	: Admin
Modified Date	: 1/24/2017 2:07:19 PM

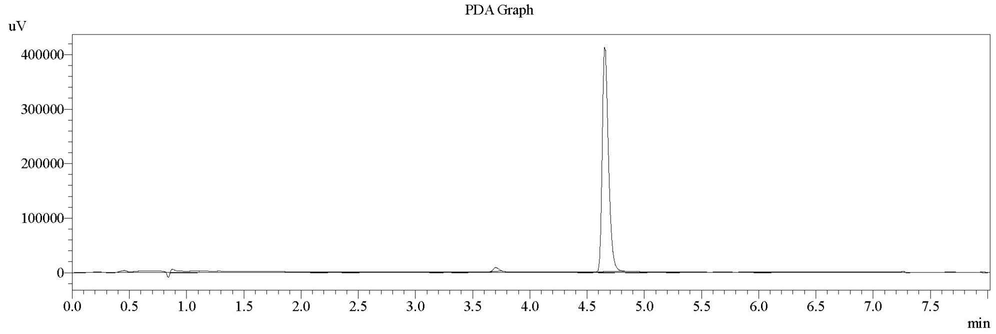

PDA Ch1 254nm 4nm

Peak#	Name	Ret. Time	Area	Area %
1		3.697	22427	1.427
2		4.650	1549438	98.573

MS Spectrum Graph

#:1 Ret.Time:Averaged 4.690-4.710(Scan#:470-472)
Mass Peaks:22 Base Peak:403.05(245047) Polarity:Pos Segment1 - Event1

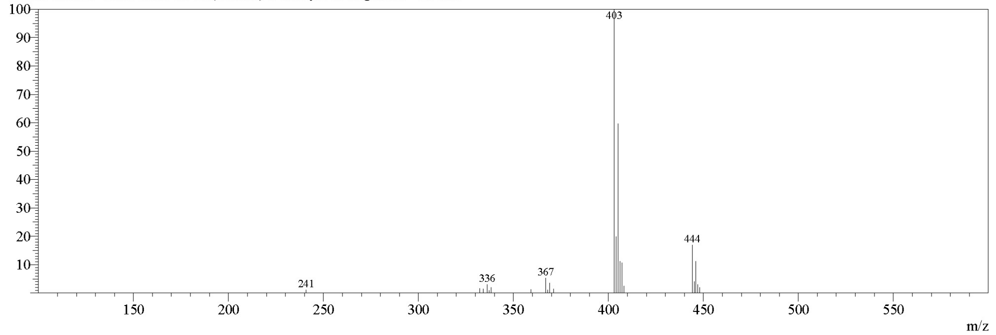

MS Spectrum Table

#:1 Ret.Time:
BG Mode:Calc 4.580<->4.910(459<->492)
Mass Peaks:22 Base Peak:403.05(245047) Polarity:Pos Segment1 - Event1

#	m/z	Abs.Inten.	Rel.Inten.	Charge	Polarity	Monoisotopic	#	m/z	Abs.Inten.	Rel.Inten.	Charge	Polarity	Monoisotopic
1	240.90	2748	1.12				12	403.05	245047	100.00			
2	332.25	4102	1.67				13	404.10	48852	19.94			
3	334.15	3658	1.49				14	405.05	146051	59.60			
4	336.15	7441	3.04				15	406.00	27547	11.24			
5	337.15	2468	1.01				16	407.05	26258	10.72			
6	338.20	4864	1.98				17	408.10	6298	2.57			
7	359.35	3002	1.23				18	444.10	41602	16.98			
8	367.05	12833	5.24				19	445.25	10121	4.13			
9	368.15	2868	1.17				20	446.15	27620	11.27			
10	369.10	8749	3.57				21	447.15	7595	3.10			
11	371.15	3473	1.42				22	448.10	4990	2.04			

Figure S6 LCMS spectrum of compound NPD-0006

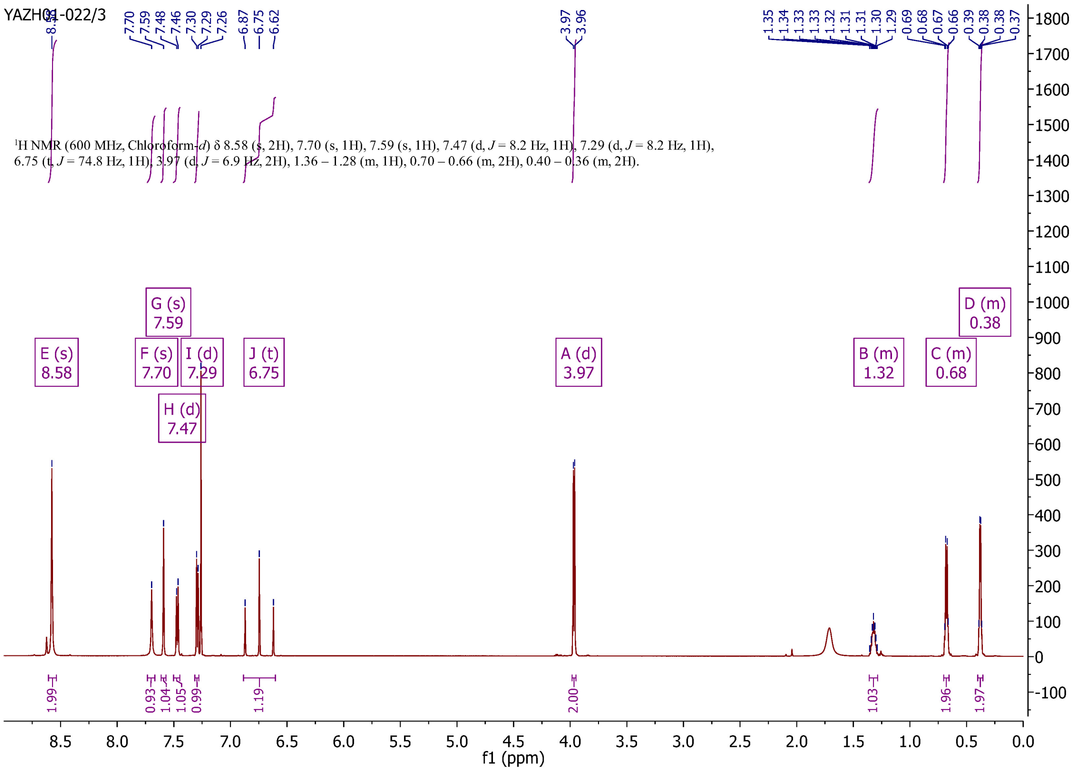

Figure S7 ^{1}H NMR spectrum of compound NPD-0006

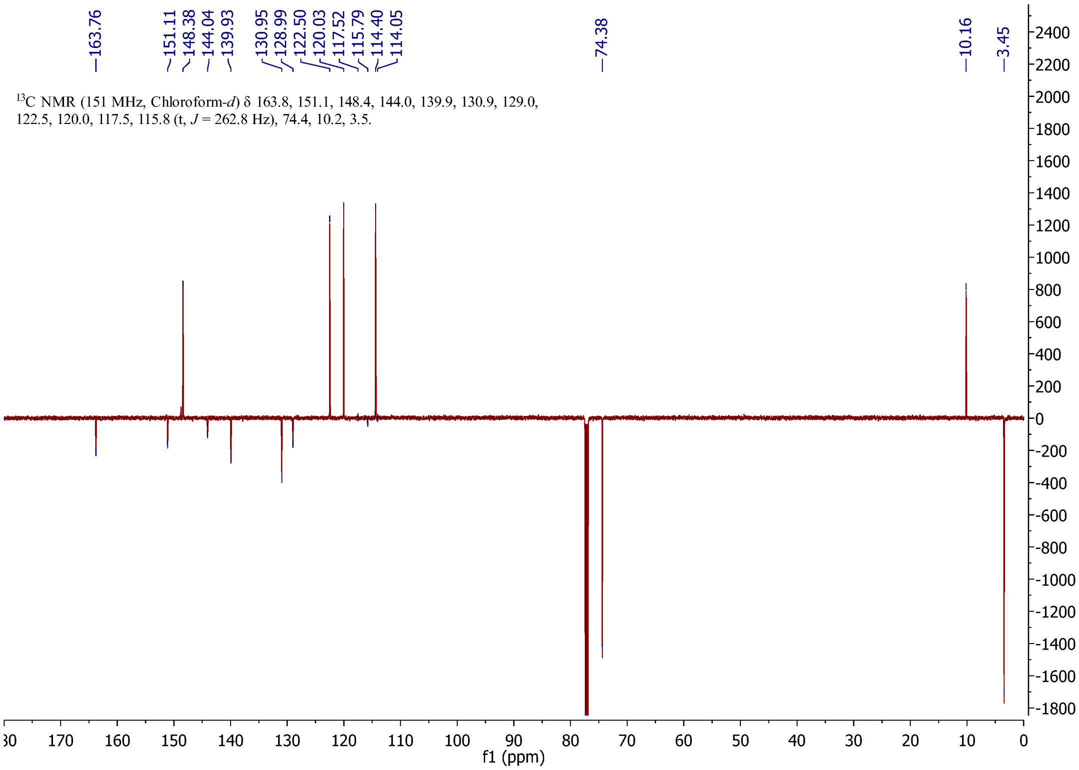

Figure S8 ^{13}C NMR spectrum of compound NPD-0006

Acquired by	: Admin
Date Acquired	: 6/5/2019 10:03:02 AM
Sample Name	: VUF14667
Sample ID	:
Tray#	: 1
Vial#	: 3
Injection Volume	: 3
Data File	: C:\LabSolutions\Data\2019\2019-wk19\VUF14667.lcd
Background File	: blanco 06052019.lcd
Method File	: Method SCAN ACID standard.lcm
Report Format	: DefaultLCMS.lcr
Tuning File	: C:\LabSolutions\Tuning\Tuning-ESI-pos-neg01072015a.lct
Processed by	: Admin
Modified Date	: 6/5/2019 10:28:29 AM

PDA Graph

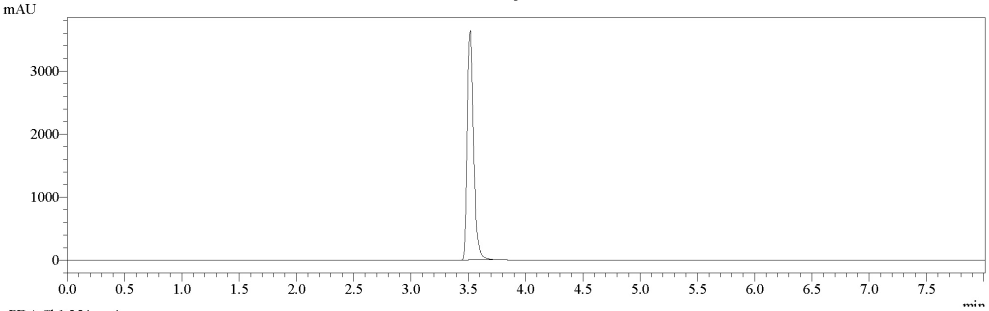

PDA Ch1 254nm 4nm

Peak#	Name	Ret. Time	Area	Area %
1		3.511	13845926	100.000

MS Spectrum Graph

#:1 Ret.Time:Averaged 3.550-3.570(Scan#:356-358)
Mass Peaks:12 Base Peak:326.95(850318) Polarity:Pos Segment1 - Event1

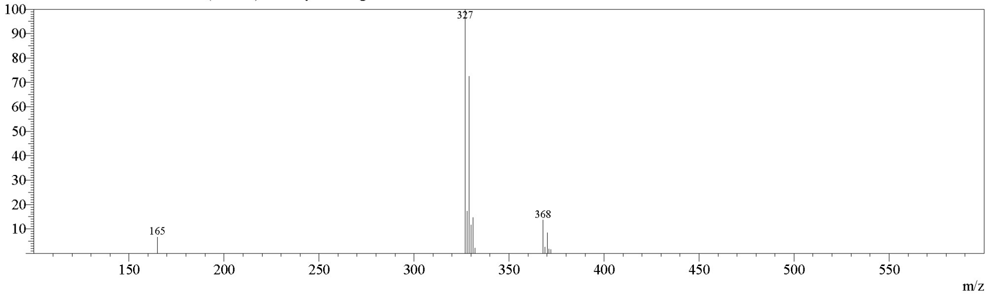

MS Spectrum Table

#:1 Ret.Time:
BG Mode:Calc 3.450<->3.950(346<->396)
Mass Peaks:12 Base Peak:326.95(850318) Polarity:Pos Segment1 - Event1

#	m/z	Abs.Inten.	Rel.Inten.	Charge	Polarity	Monoisotopic
1	165.00	56320	6.62			
2	326.95	850318	100.00			
3	328.00	147851	17.39			
4	329.00	617151	72.58			
5	329.95	99378	11.69			
6	331.00	124143	14.60			
7	332.00	18582	2.19			
8	368.00	115503	13.58			
9	369.05	22429	2.64			
10	370.05	71128	8.36			
11	371.05	16009	1.88			
12	372.05	12762	1.50			

Figure S9 LCMS spectrum of compound NPD-0446

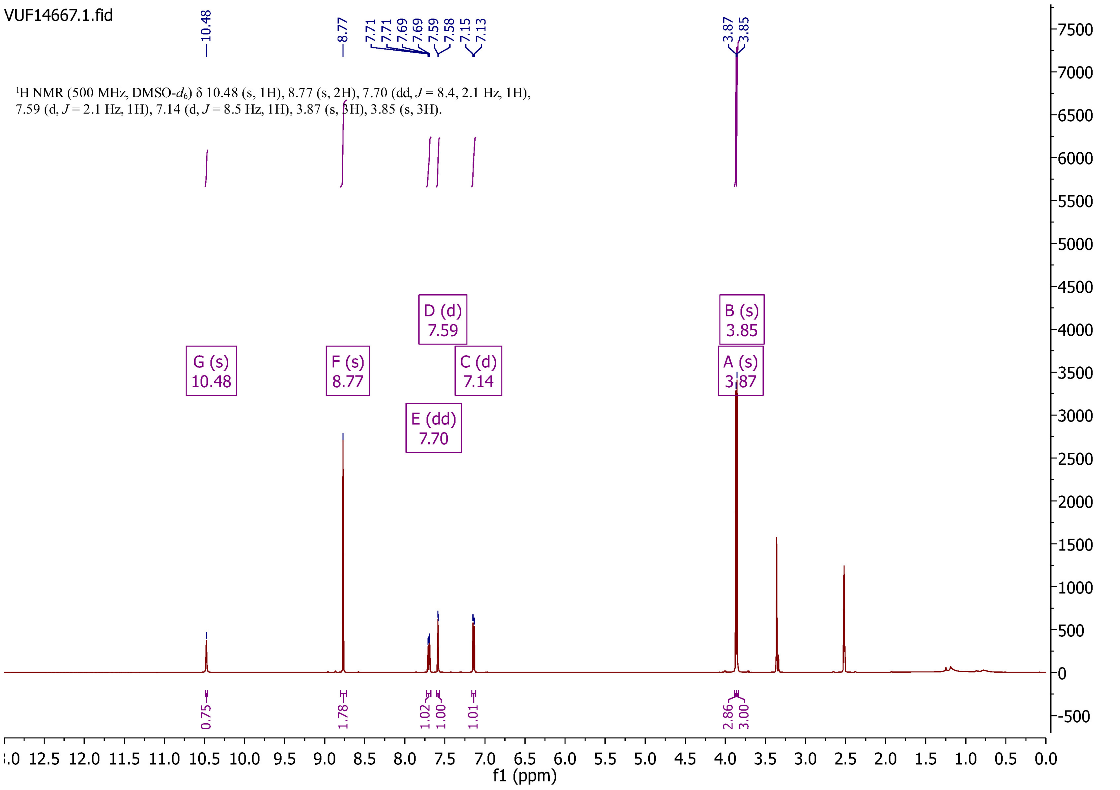

Figure S10 ^{1}H NMR spectrum of compound NPD-0446

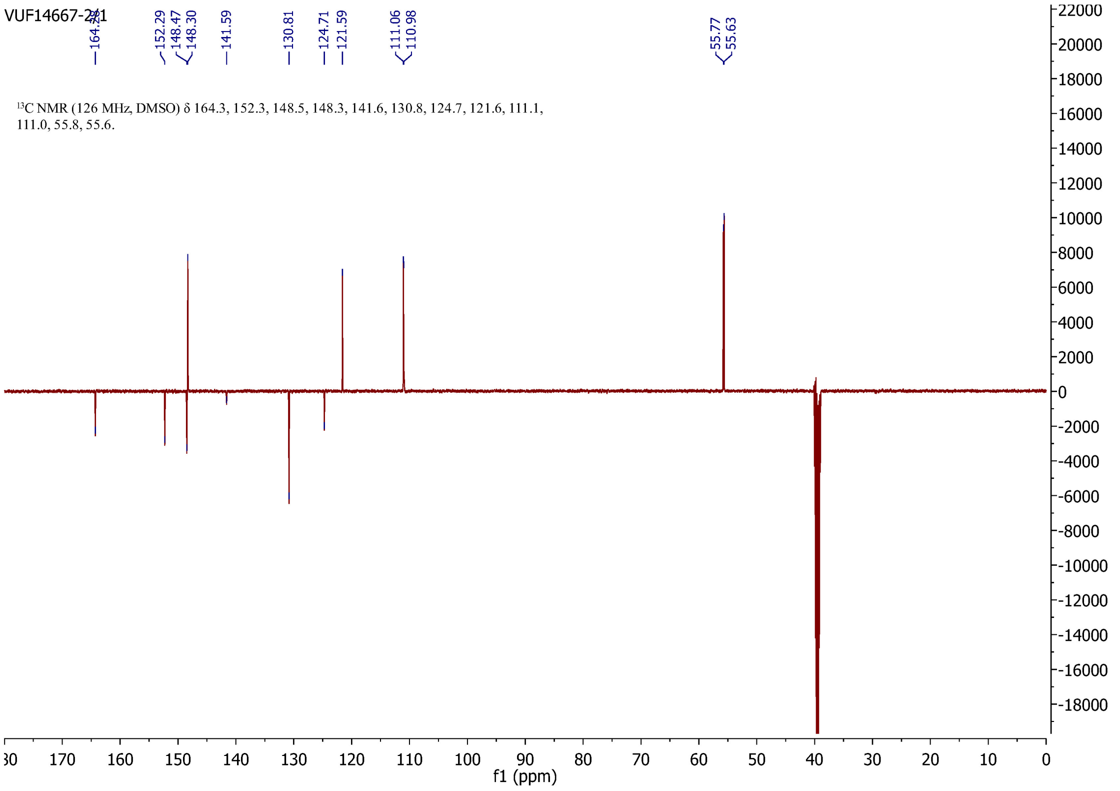

Figure S11 ^{13}C NMR spectrum of compound NPD-0446

Acquired by : Admin
Date Acquired : 1/26/2016 5:05:32 PM
Sample Name : Fatih compound 62
Sample ID :
Tray# : 1
Vial# : 5
Injection Volume : 1
Data File : C:\LabSolutions\Methods\FAR Pract Fatih compound 62.lcd
Background File : Blanco-2 26012016.lcd
Method File : Method SCAN ACID standard.lcm
Report Format : DefaultLCMS.lcr
Tuning File : C:\LabSolutions\Tuning File\Tuning-ESI-pos-neg01072015.lct
Processed by : Admin
Modified Date : 1/27/2016 9:06:35 AM

PDA Graph

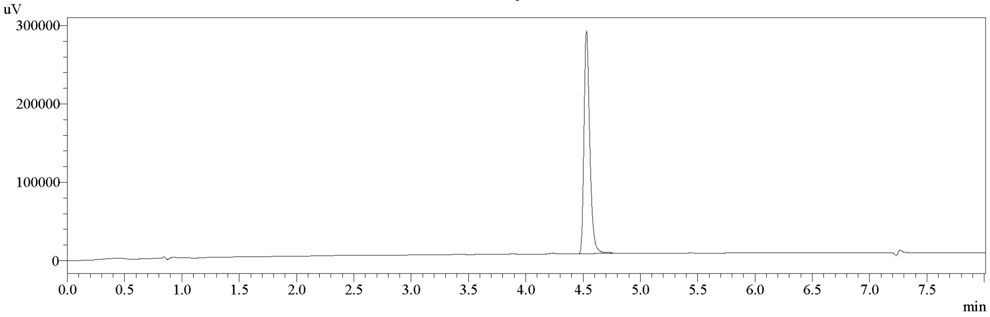

PDA Ch1 254nm 4nm

Peak#	Name	Ret. Time	Area	Area %
1		4.524	989667	100.000

MS Spectrum Graph

#:1 Ret.Time:Averaged 4.650-4.680(Scan#:465-468)
Mass Peaks:6 Base Peak:403.02(1149896) Polarity:Pos Segment1 - Event1

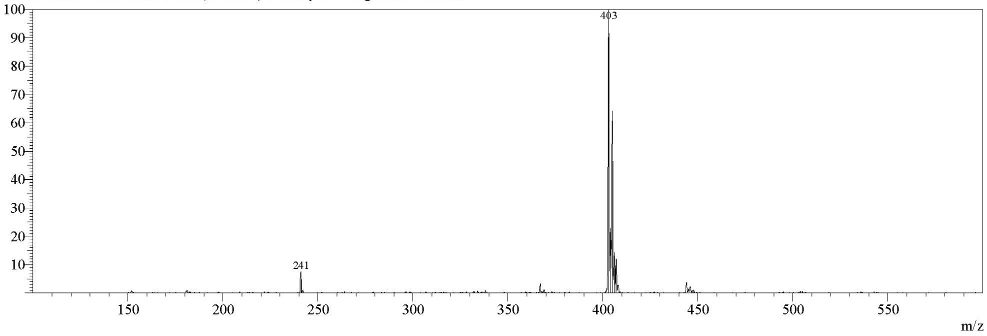

MS Spectrum Table

#:1 Ret.Time:
BG Mode:None
Mass Peaks:6 Base Peak:403.02(1067212) Polarity:Pos Segment1 - Event

#	m/z	Abs.Inten.	Rel.Inten.	Charge	Polarity	Monoisotopic	#	m/z	Abs.Inten.	Rel.Inten.	Charge	Polarity	Monoisotopic
1	241.07	73936	6.93				4	405.01	707412	66.29			
2	403.02	1067212	100.00				5	406.08	142461	13.35			
3	404.08	229993	21.55				6	407.01	121379	11.37			

Figure S12 LCMS spectrum of compound NPD-0546

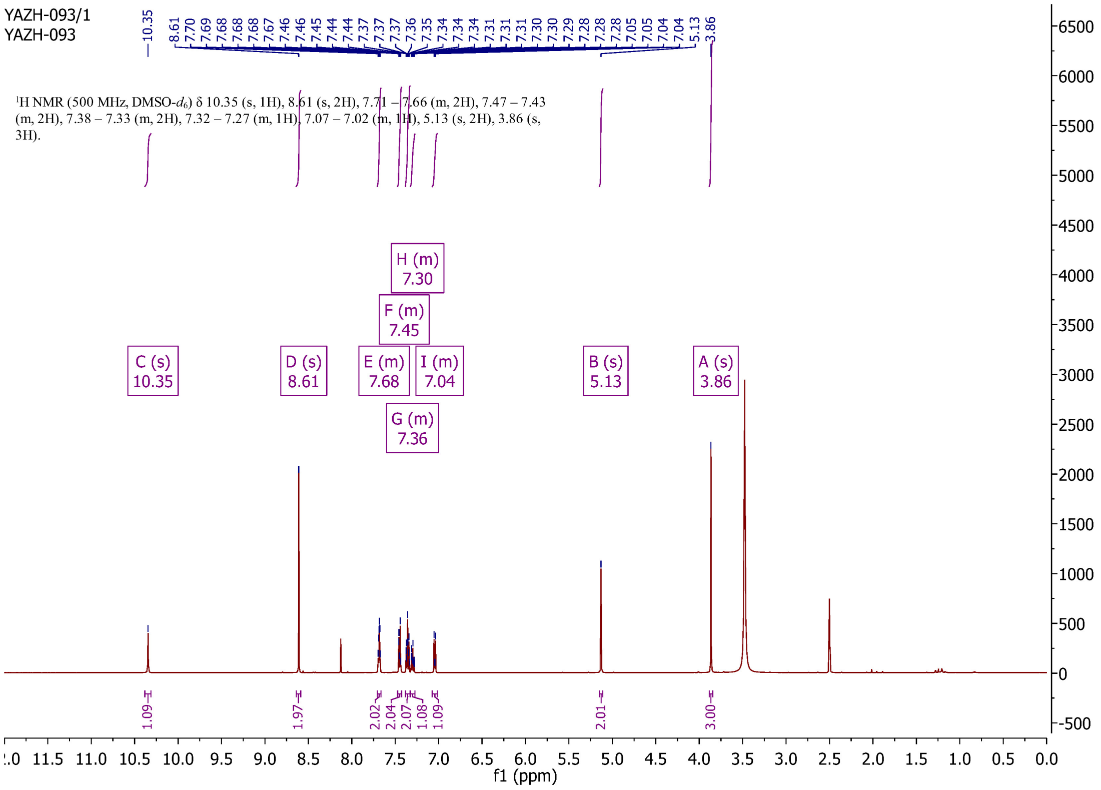

Figure S13 ¹H NMR spectrum of compound NPD-0546

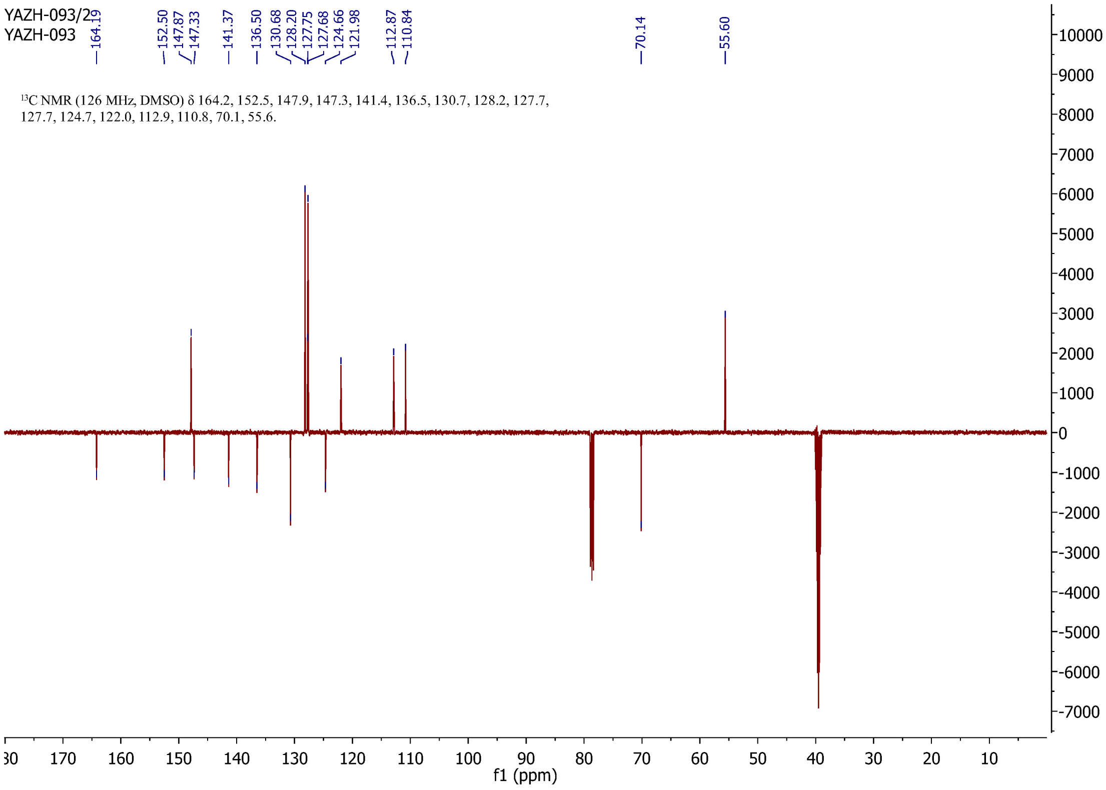

Figure S14 ¹³C NMR spectrum of compound NPD-0546

Acquired by : Admin
Date Acquired : 6/5/2019 9:54:24 AM
Sample Name : VUF14666
Sample ID :
Tray# : 1
Vial# : 2
Injection Volume : 3
Data File : C:\LabSolutions\Data\2019\2019-wk19\VUF14666.lcd
Background File : blanco 06052019.lcd
Method File : Method SCAN ACID standard.lcm
Report Format : DefaultLCMS.lcr
Tuning File : C:\LabSolutions\Tuning\Tuning-ESI-pos-neg01072015a.lct
Processed by : Admin
Modified Date : 6/5/2019 10:25:29 AM

PDA Graph

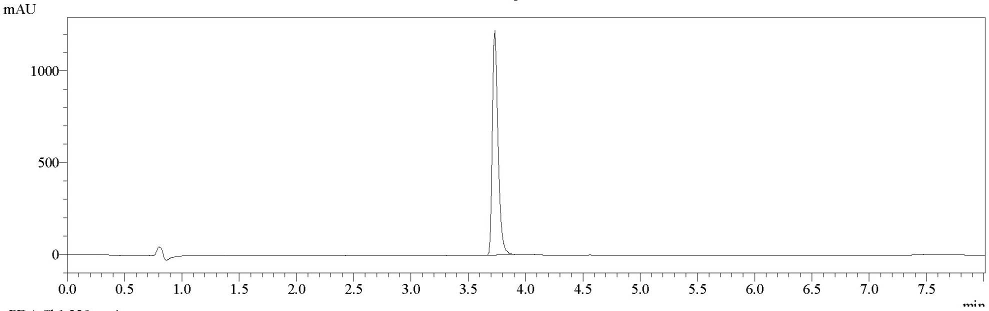

PDA Ch1 230nm 4nm

Peak#	Name	Ret. Time	Area	Area %
1		3.726	4222080	100.000

MS Spectrum Graph

#:1 Ret.Time:Averaged 3.780-3.800(Scan#:379-381)
Mass Peaks:11 Base Peak:297.00(756618) Polarity:Pos Segment1 - Event1

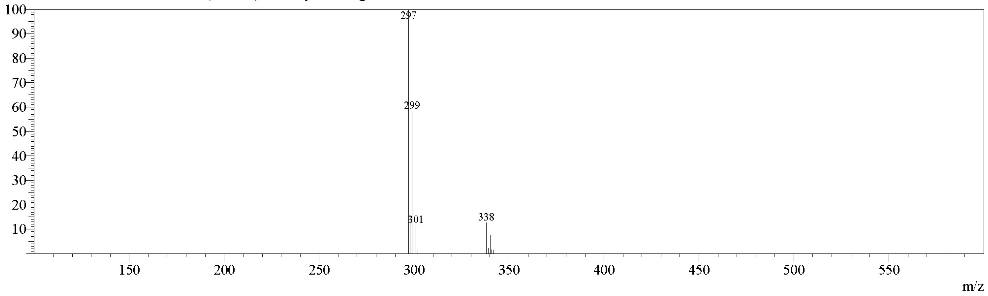

MS Spectrum Table

#:1 Ret.Time:
BG Mode:Calc 3.680<->4.090(369<->410)
Mass Peaks:11 Base Peak:297.00(756618) Polarity:Pos Segment1 - Event1

#	m/z	Abs.Inten.	Rel.Inten.	Charge	Polarity	Monoisotopic	#	m/z	Abs.Inten.	Rel.Inten.	Charge	Polarity	Monoisotopic
1	297.00	756618	100.00				7	338.00	95139	12.57			
2	298.00	113869	15.05				8	339.05	16956	2.24			
3	299.00	440277	58.19				9	340.05	55883	7.39			
4	300.00	70642	9.34				10	341.05	11650	1.54			
5	300.95	87257	11.53				11	341.95	9945	1.31			
6	302.00	11893	1.57										

Figure S15 LCMS spectrum of compound NPD-0548

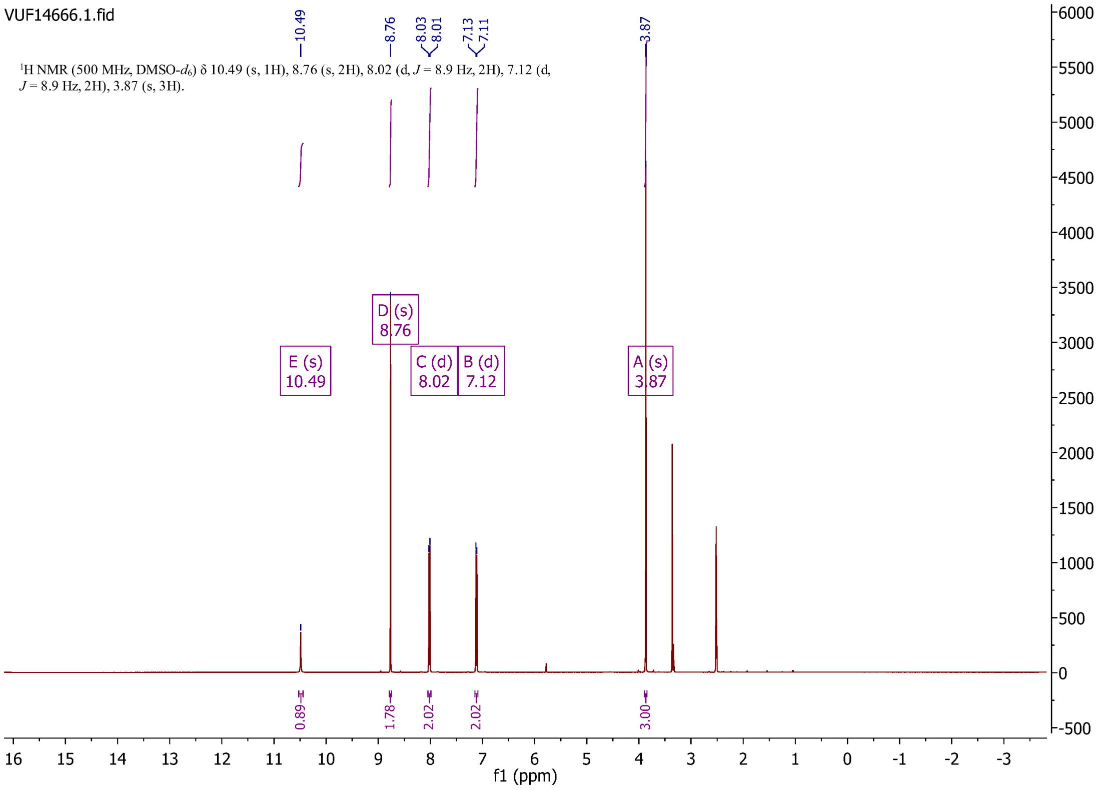

Figure S16 ^{1}H NMR spectrum of compound NPD-0548

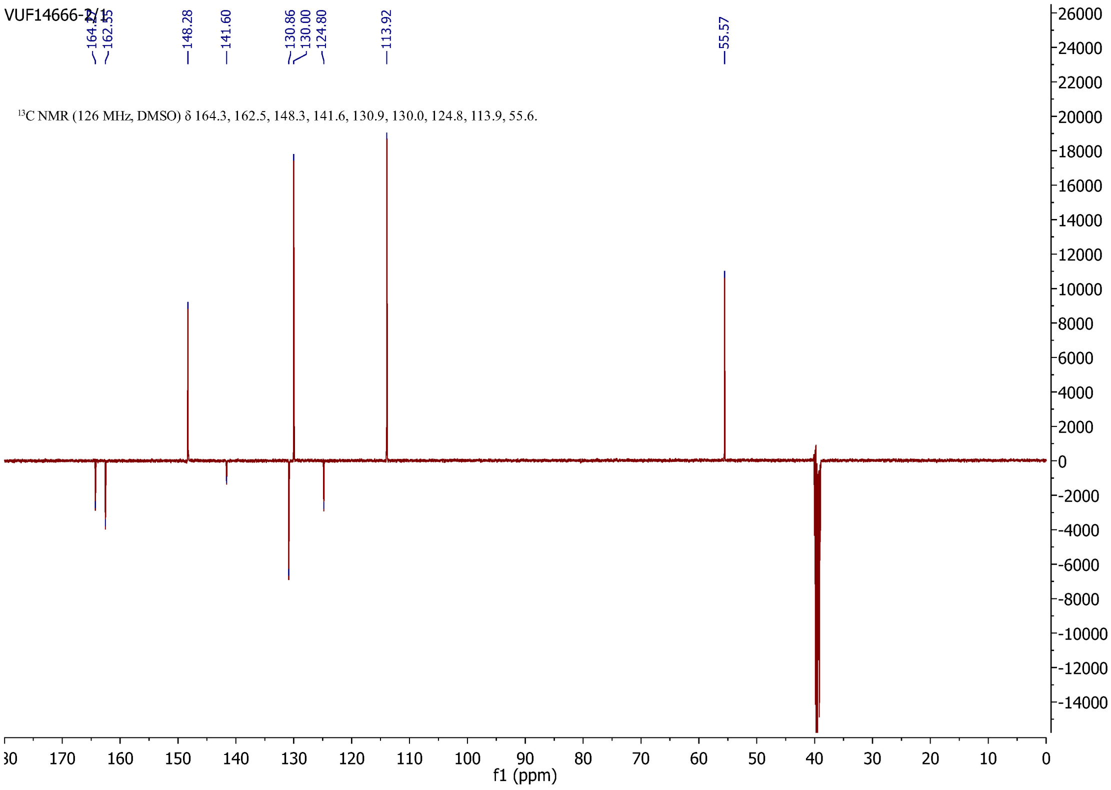

Figure S17 ^{13}C NMR spectrum of compound NPD-0548

Acquired by : Admin
Date Acquired : 10/15/2015 4:57:07 PM
Sample Name : YAZH-005
Sample ID :
Tray# : 1
Vial# : 3
Injection Volume : 1
Data File : C:\LabSolutions\Data\2015\2015-wk42\YAZH-005.lcd
Background File : blanco 16102016.lcd
Method File : Method SCAN ACID standard.lcm
Report Format : DefaultLCMS.lcr
Tuning File : C:\LabSolutions\Tuning File\Tuning-ESI-pos-neg01072015.lct
Processed by : Admin
Modified Date : 10/16/2015 4:42:28 PM

PDA Graph

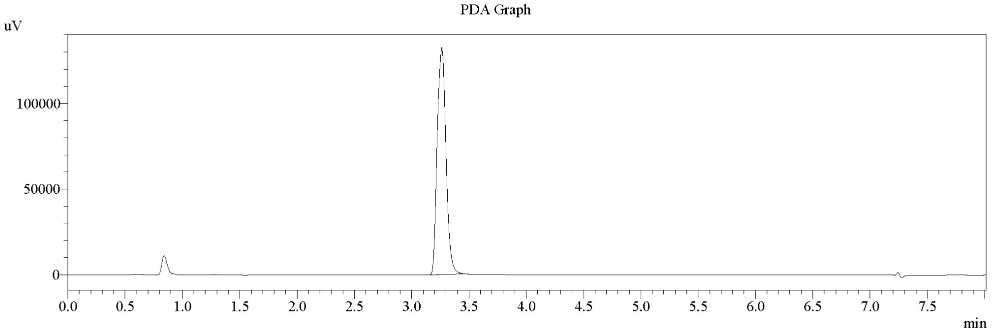

PDA Ch1 254nm 4nm

Peak#	Name	Ret. Time	Area	Area %
1		3.258	710577	100.000

MS Spectrum Graph
#:1 Ret.Time:Averaged 3.320-3.340(Scan#:333-335
Mass Peaks:6 Base Peak:291.00(1921412) Polarity:Pos Segment1 - Event1

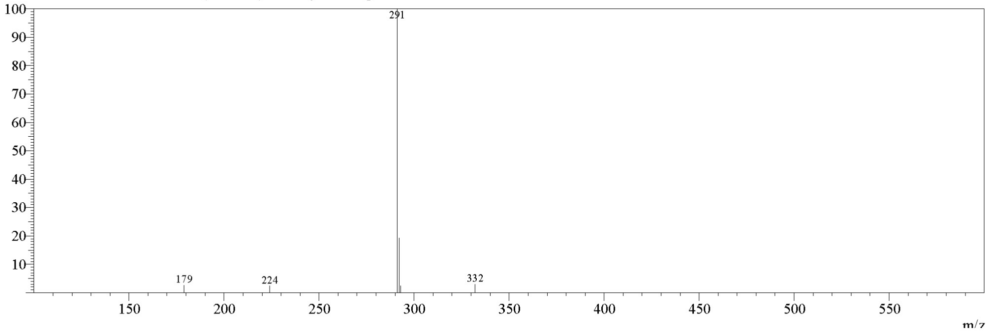

MS Spectrum Table
#:1 Ret.Time:
BG Mode:Calc 3.190<->3.560(320<->357)
Mass Peaks:6 Base Peak:291.00(1921412) Polarity:Pos Segment1 - Event1

#	m/z	Abs.Inten.	Rel.Inten.	Charge	Polarity	Monoisotopic
1	179.00	50991	2.65			
2	224.10	45000	2.34			
3	291.00	1921412	100.00			
4	292.05	369332	19.22			
5	293.05	44757	2.33			
6	332.10	56581	2.94			

Figure S18 LCMS spectrum of compound NPD-1177

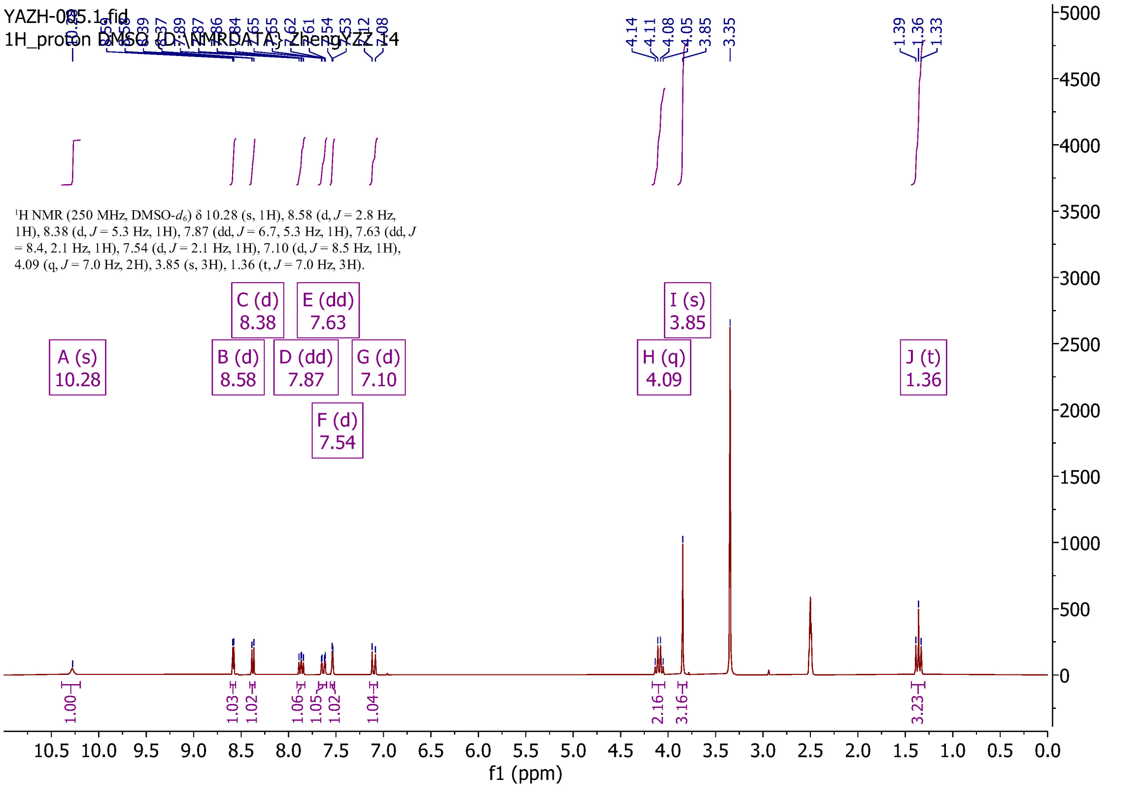

Figure S19 ^{1}H NMR spectrum of compound NPD-1177

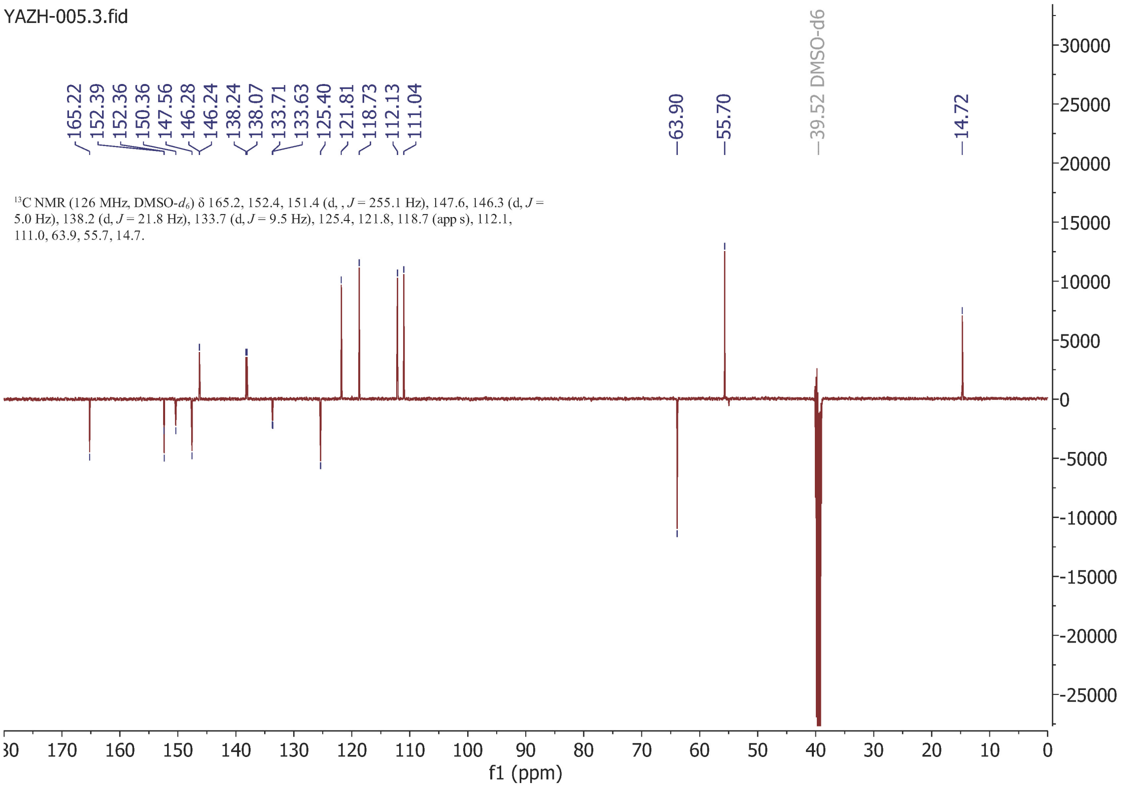

Figure S20 ^{13}C NMR spectrum of compound NPD-1177

Acquired by	: Admin
Date Acquired	: 10/15/2015 5:05:45 PM
Sample Name	: YAZH-006
Sample ID	:
Tray#	: 1
Vial#	: 4
Injection Volume	: 1
Data File	: C:\LabSolutions\Data\2015\2015-wk42\YAZH-006.lcd
Background File	: blanco 16102016.lcd
Method File	: Method SCAN ACID standard.lcm
Report Format	: DefaultLCMS.lcr
Tuning File	: C:\LabSolutions\Tuning File\Tuning-ESI-pos-neg01072015.lct
Processed by	: Admin
Modified Date	: 10/16/2015 9:12:10 AM

PDA Graph

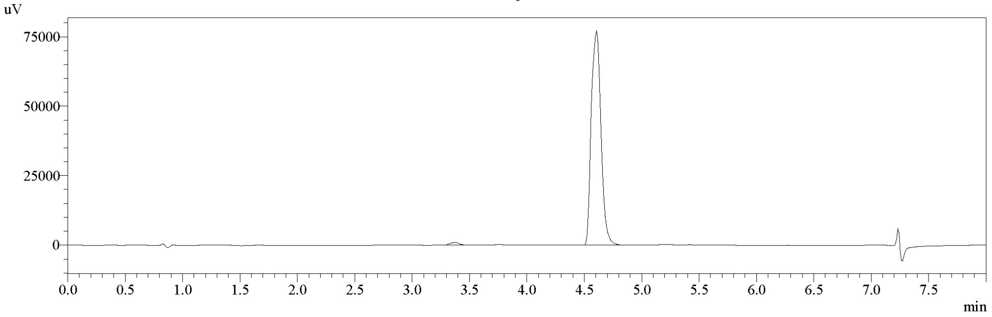

PDA Ch1 254nm 4nm

Peak#	Name	Ret. Time	Area	Area %
1		3.368	5235	1.147
2		4.601	451033	98.853

MS Spectrum Graph

#:1 Ret.Time:Averaged 4.670-4.690(Scan#:468-470
Mass Peaks:20 Base Peak:345.00(212441) Polarity:Pos Segment1 - Event1

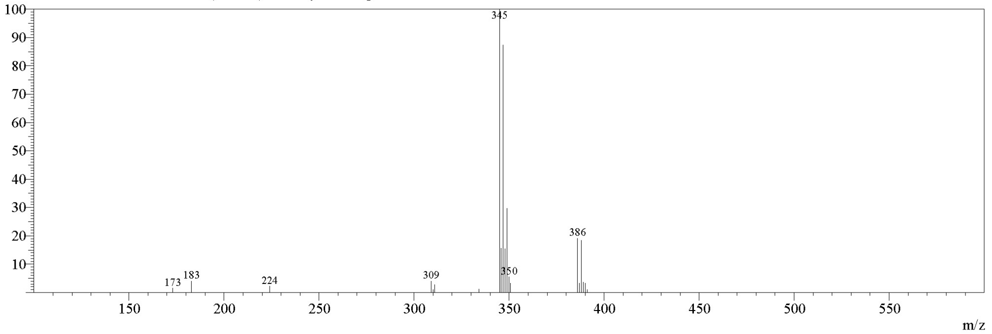

MS Spectrum Table

#:1 Ret.Time:
BG Mode:Calc 4.550<->4.900(456<->491)
Mass Peaks:20 Base Peak:345.00(212441) Polarity:Pos Segment1 - Event1

#	m/z	Abs.Inten.	Rel.Inten.	Charge	Polarity	Monoisotopic
1	173.00	3126	1.47			
2	182.90	8434	3.97			
3	223.95	4605	2.17			
4	308.95	8497	4.00			
5	310.10	2352	1.11			
6	310.80	5736	2.70			
7	334.20	2461	1.16			
8	345.00	212441	100.00			
9	345.90	33033	15.55			
10	346.95	185495	87.32			
11	348.00	32728	15.41			
12	348.95	63089	29.70			
13	350.00	11665	5.49			
14	350.80	6922	3.26			
15	386.05	40483	19.06			
16	386.95	6898	3.25			
17	387.90	39119	18.41			
18	389.15	7650	3.60			
19	390.05	6968	3.28			
20	391.05	2142	1.01			

Figure S21 LCMS spectrum of compound NPD-1178

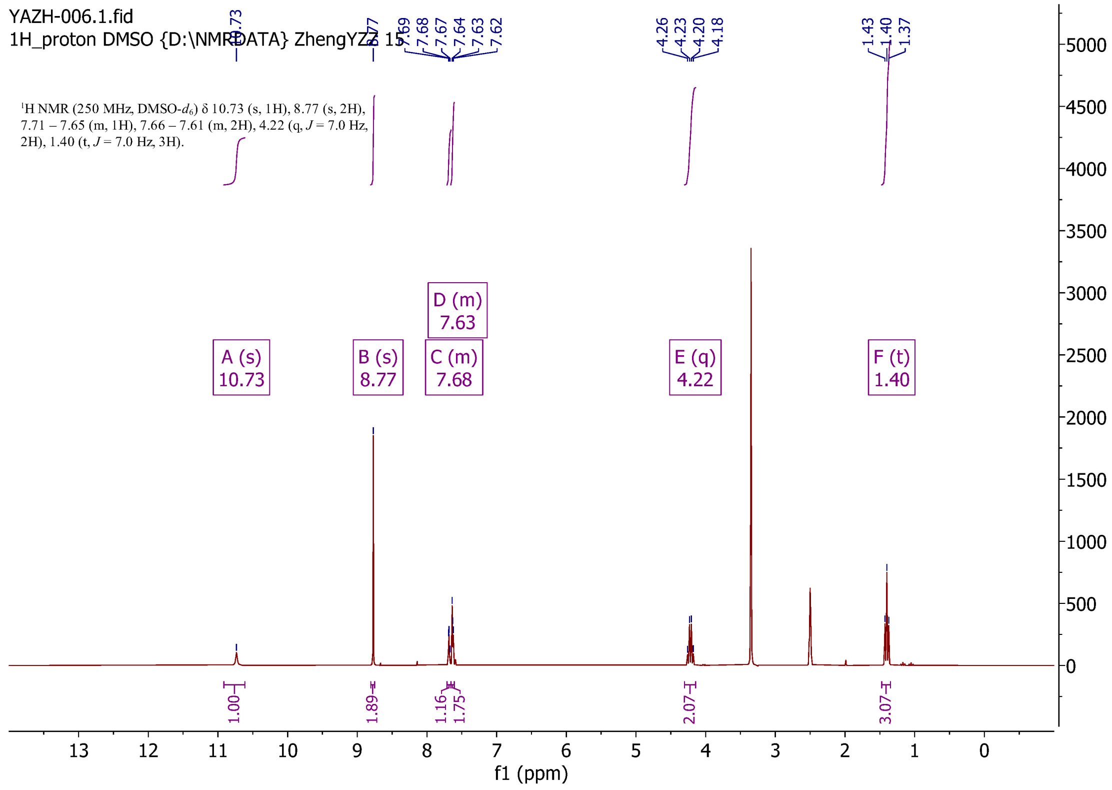

Figure S22 ^{1}H NMR spectrum of compound NPD-1178

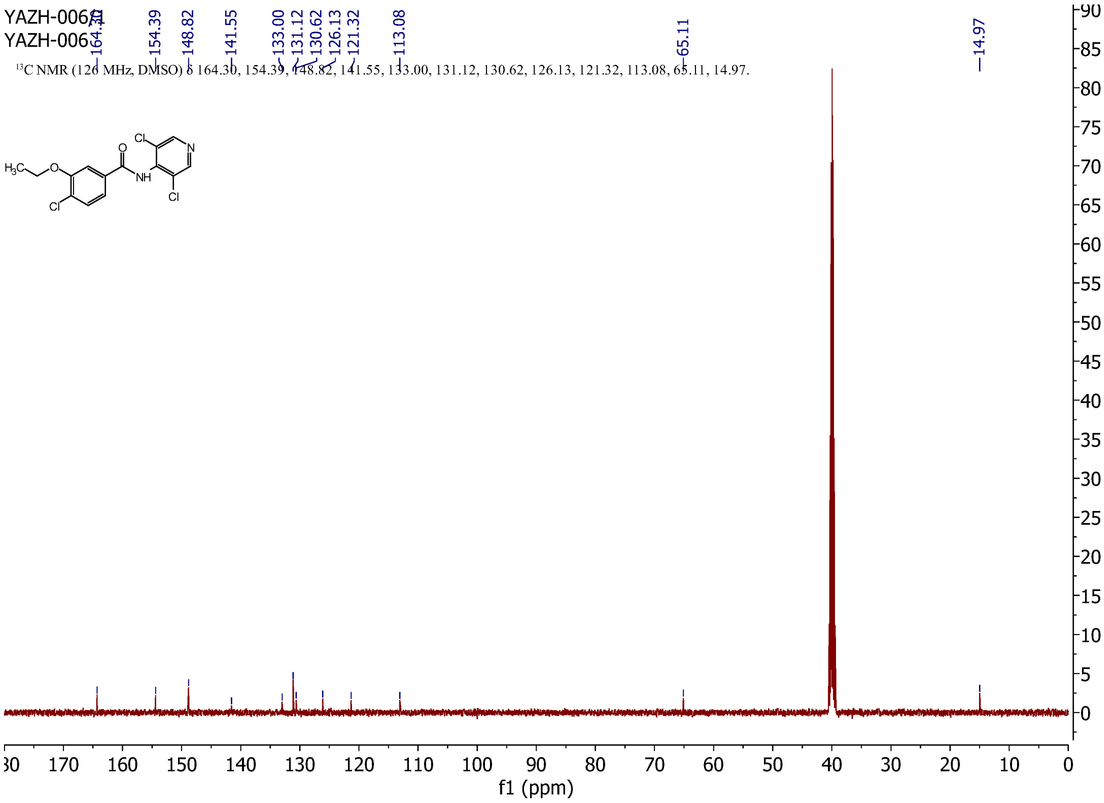

Figure S23 ^{13}C NMR spectrum of compound NPD-1178

Acquired by : Admin
Date Acquired : 10/15/2015 5:14:23 PM
Sample Name : YAZH-007
Sample ID :
Tray# : 1
Vial# : 5
Injection Volume : 1
Data File : C:\LabSolutions\Data\2015\2015-wk42\YAZH-007.lcd
Background File : blanco 16102016.lcd
Method File : Method SCAN ACID standard.lcm
Report Format : DefaultLCMS.lcr
Tuning File : C:\LabSolutions\Tuning File\Tuning-ESI-pos-neg01072015.lct
Processed by : Admin
Modified Date : 10/16/2015 9:14:19 AM

PDA Graph

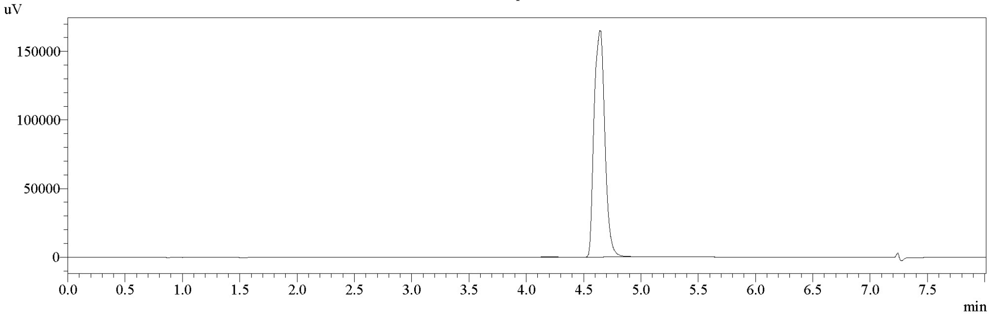

PDA Ch1 254nm 4nm

Peak#	Name	Ret. Time	Area	Area %
1		4.210	3214	0.297
2		4.639	1076423	99.535
3		5.231	1810	0.167

MS Spectrum Graph

#:1 Ret.Time:Averaged 4.700-4.720(Scan#:471-473)
Mass Peaks:9 Base Peak:310.90(1130445) Polarity:Pos Segment1 - Event1

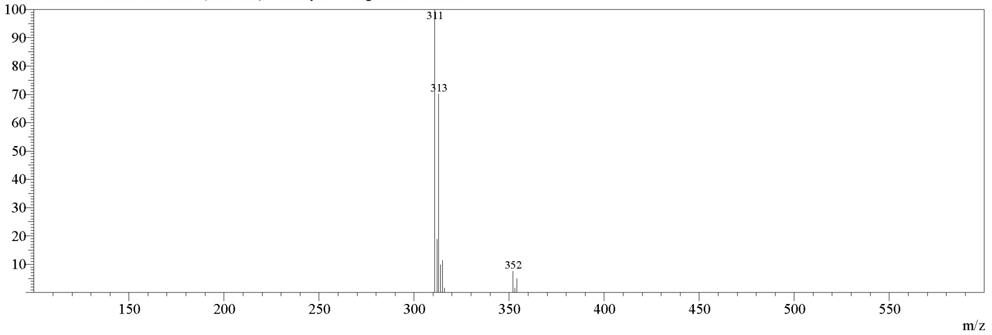

MS Spectrum Table

#:1 Ret.Time:
BG Mode:Calc 4.560<->4.960(457<->497)
Mass Peaks:9 Base Peak:310.90(1130445) Polarity:Pos Segment1 - Event1

#	m/z	Abs.Inten.	Rel.Inten.	Charge	Polarity	Monoisotopic
1	310.90	1130445	100.00			
2	312.00	212987	18.84			
3	312.95	792980	70.15			
4	314.00	111042	9.82			
5	315.00	129351	11.44			
6	316.10	17698	1.57			
7	352.00	84880	7.51			
8	353.00	18323	1.62			
9	354.00	55399	4.90			

Figure S24 LCMS spectrum of compound NPD-1179

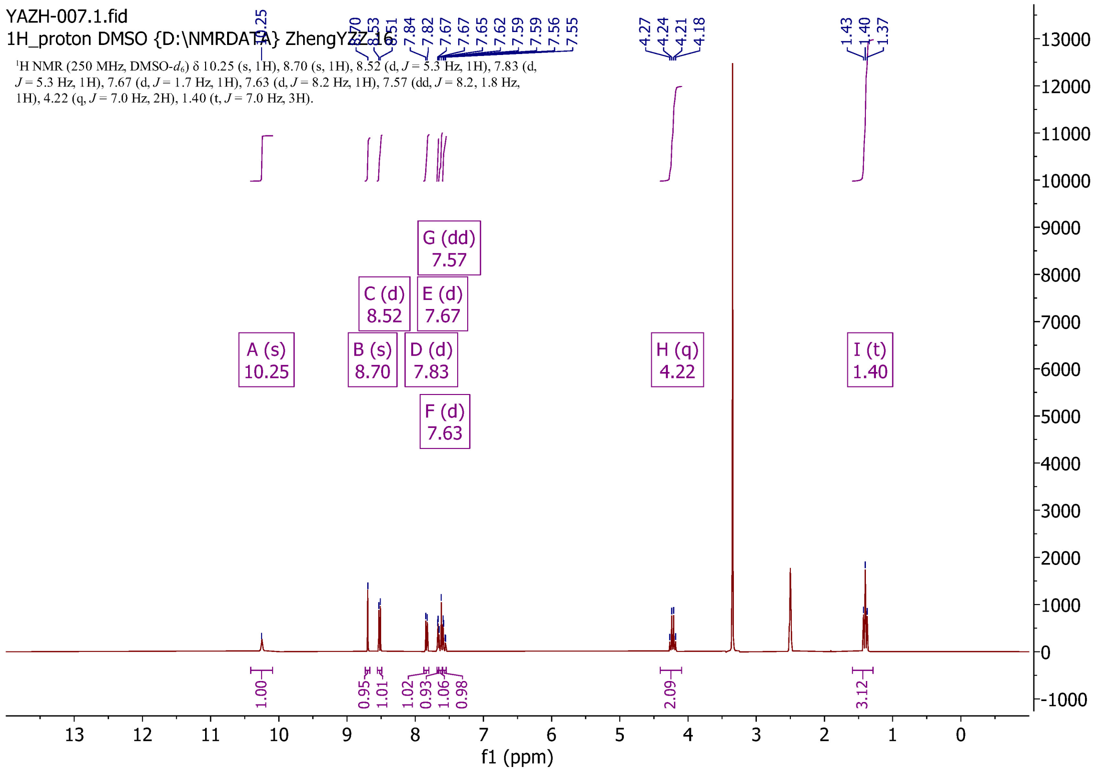

Figure S25 ^{1}H NMR spectrum of compound NPD-1179

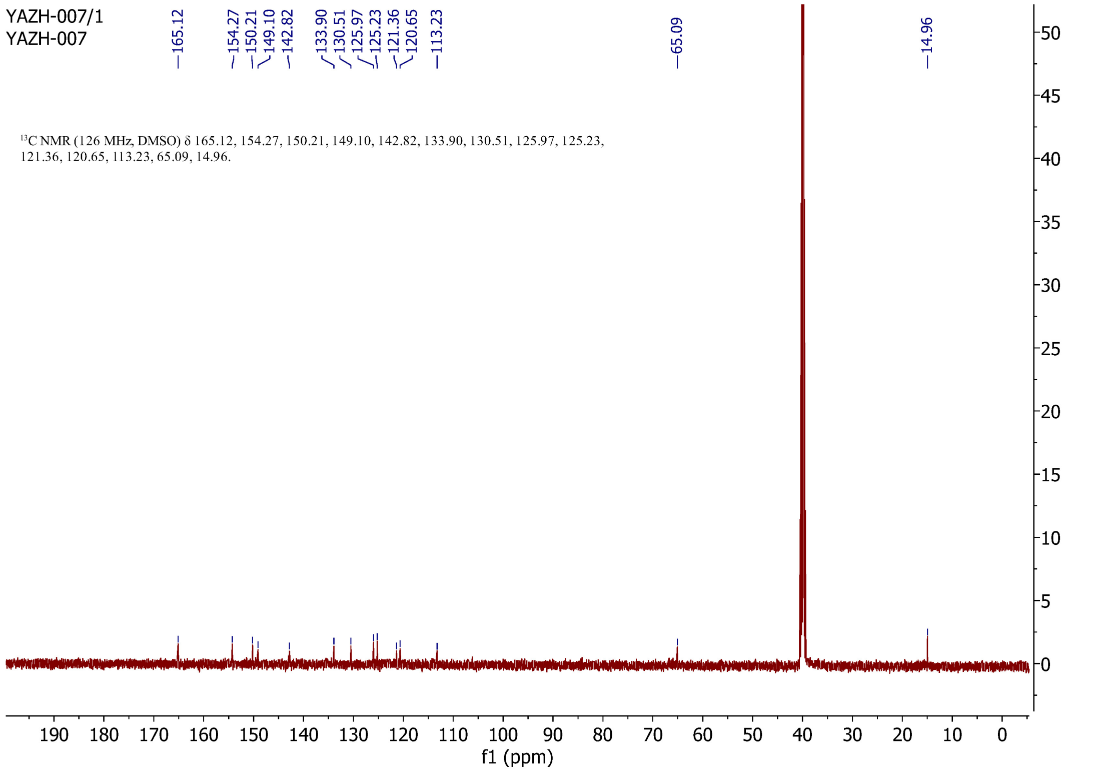

Figure S26 ^{13}C NMR spectrum of compound NPD-1179

Acquired by	: Admin
Date Acquired	: 10/15/2015 5:57:41 PM
Sample Name	: YAZH-012
Sample ID	:
Tray#	: 1
Vial#	: 10
Injection Volume	: 1
Data File	: C:\LabSolutions\Data\2015\2015-wk42\YAZH-012.lcd
Background File	: blanco 16102016.lcd
Method File	: Method SCAN ACID standard.lcm
Report Format	: DefaultLCMS.lcr
Tuning File	: C:\LabSolutions\Tuning File\Tuning-ESI-pos-neg01072015.lct
Processed by	: Admin
Modified Date	: 10/16/2015 9:36:21 AM

PDA Graph

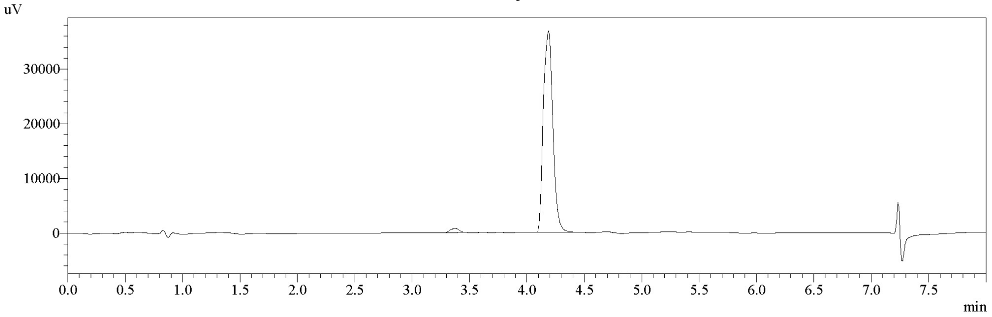

PDA Ch1 254nm 4nm

Peak#	Name	Ret. Time	Area	Area %
1		3.368	3970	1.836
2		4.183	212236	98.164

MS Spectrum Graph

#:1 Ret.Time:Averaged 4.260-4.280(Scan#:427-429)
Mass Peaks:29 Base Peak:332.95(98731) Polarity:Pos Segment1 - Event1

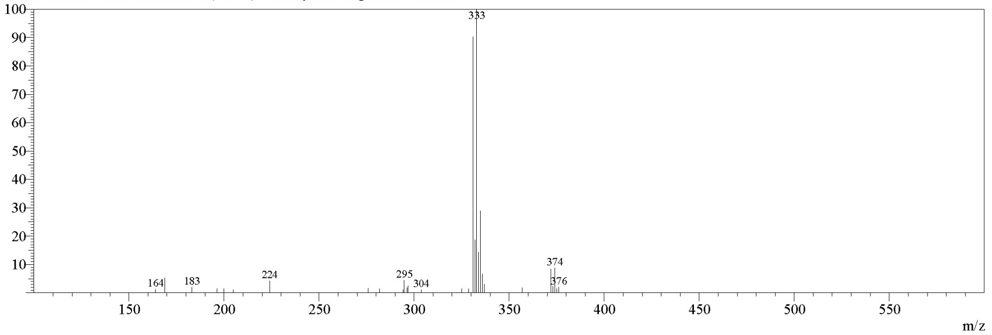

MS Spectrum Table

#:1 Ret.Time:
BG Mode:Calc 4.140<->4.430(415<->444
Mass Peaks:29 Base Peak:332.95(98731) Polarity:Pos Segment1 - Event

#	m/z	Abs.Inten.	Rel.Inten.	Charge	Polarity	Monoisotopic
1	164.00	1285	1.30			
2	168.85	5260	5.33			
3	183.00	2007	2.03			
4	196.15	1366	1.38			
5	199.95	1394	1.41			
6	204.75	1083	1.10			
7	223.85	4249	4.30			
8	275.85	1686	1.71			
9	281.85	1477	1.50			
10	294.20	1034	1.05			
11	294.80	4317	4.37			
12	296.20	1779	1.80			
13	296.80	2477	2.51			
14	303.80	1057	1.07			

#	m/z	Abs.Inten.	Rel.Inten.	Charge	Polarity	Monoisotopic
15	325.15	1409	1.43			
16	328.75	1447	1.47			
17	330.90	89102	90.25			
18	332.00	18499	18.74			
19	332.95	98731	100.00			
20	333.85	14089	14.27			
21	334.95	28552	28.92			
22	335.90	6508	6.59			
23	337.00	2904	2.94			
24	357.00	1712	1.73			
25	371.95	8292	8.40			
26	373.00	2227	2.26			
27	374.00	8706	8.82			
28	374.95	1519	1.54			

Figure S27 LCMS spectrum of compound NPD-1180

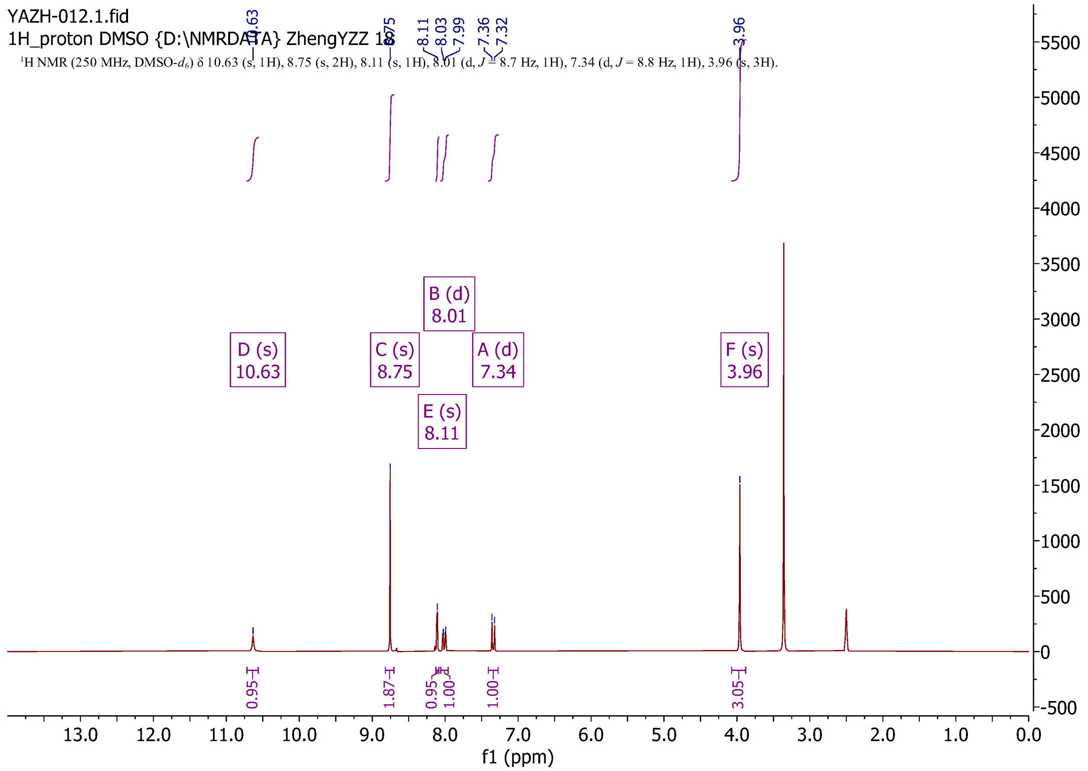

Figure S28 ^{1}H NMR spectrum of compound NPD-1180

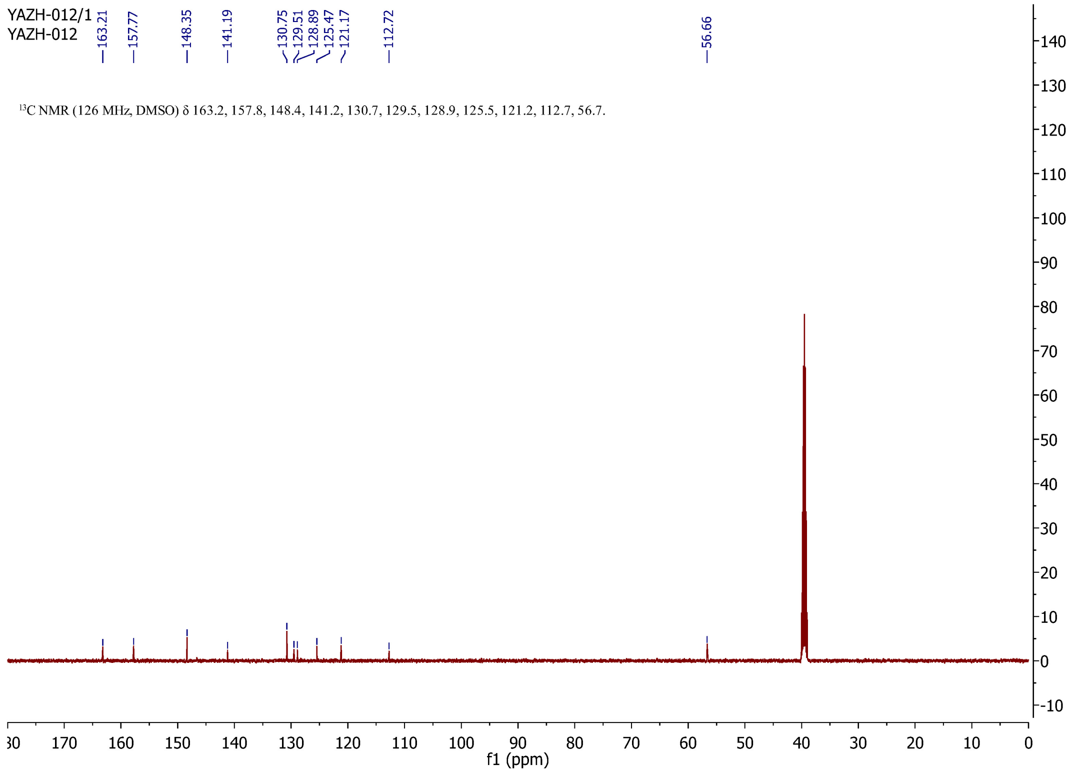

Figure S29 ^{13}C NMR spectrum of compound NPD-1180

Acquired by	: Admin
Date Acquired	: 10/15/2015 6:06:19 PM
Sample Name	: YAZH-013
Sample ID	:
Tray#	: 1
Vial#	: 11
Injection Volume	: 1
Data File	: C:\LabSolutions\Data\2015\2015-wk42\YAZH-013.lcd
Background File	: blanco 16102016.lcd
Method File	: Method SCAN ACID standard.lcm
Report Format	: DefaultLCMS.lcr
Tuning File	: C:\LabSolutions\Tuning File\Tuning-ESI-pos-neg01072015.lct
Processed by	: Admin
Modified Date	: 10/16/2016 9:38:45 AM

PDA Graph

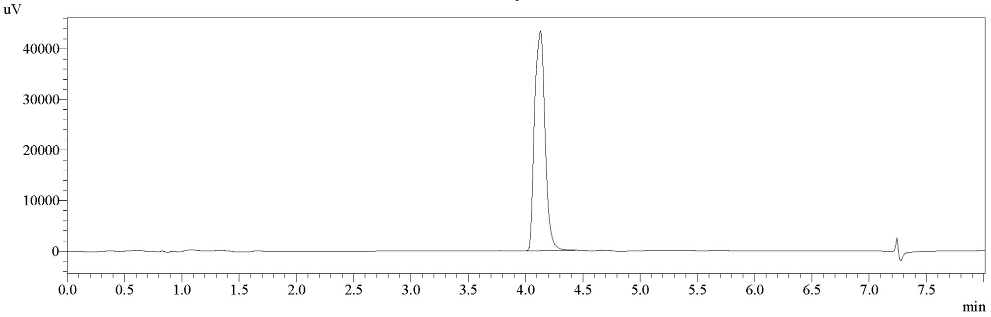

PDA Ch1 254nm 4nm

Peak#	Name	Ret. Time	Area	Area %
1		4.125	275772	100.000

MS Spectrum Graph

#:1 Ret.Time:Averaged 4.180-4.200(Scan#:419-421)
Mass Peaks:9 Base Peak:296.95(379046) Polarity:Pos Segment1 - Event1

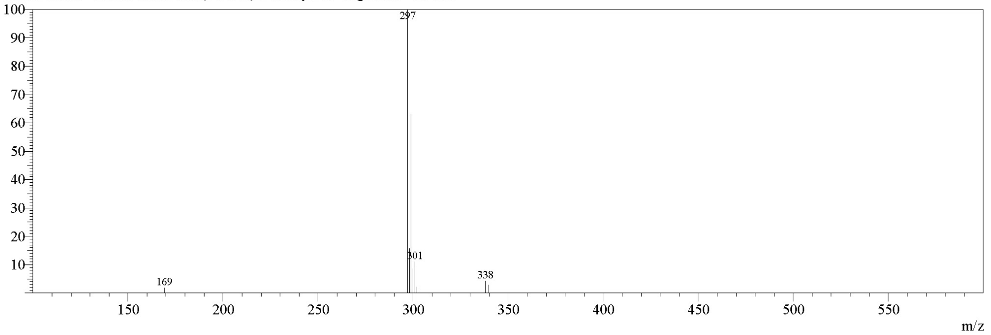

MS Spectrum Table

#:1 Ret.Time:
BG Mode:Calc 4.050<->4.410(406<->442)
Mass Peaks:9 Base Peak:296.95(379046) Polarity:Pos Segment1 - Event1

#	m/z	Abs.Inten.	Rel.Inten.	Charge	Polarity	Monoisotopic
1	169.00	6886	1.82			
2	296.95	379046	100.00			
3	298.00	59840	15.79			
4	299.00	239611	63.21			
5	300.00	32198	8.49			
6	300.95	41810	11.03			
7	301.95	7970	2.10			
8	337.90	15932	4.20			
9	339.95	10560	2.79			

Figure S30 LCMS spectrum of compound NPD-1181

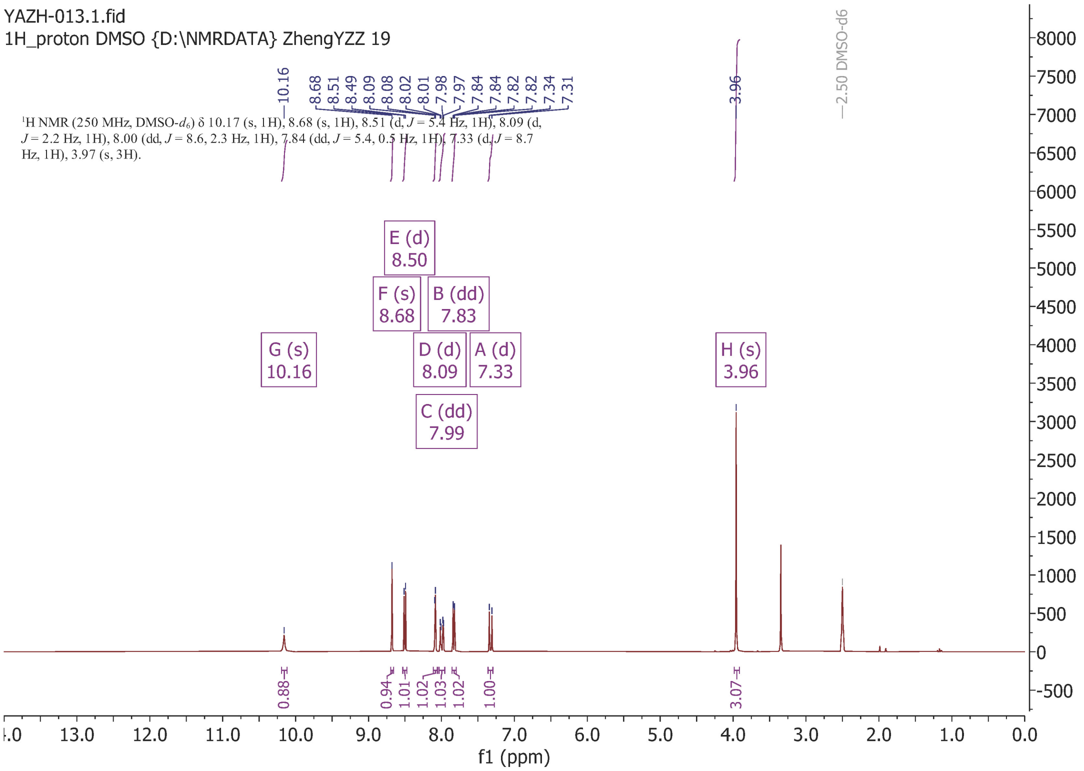

Figure S31 ^{1}H NMR spectrum of compound NPD-1181

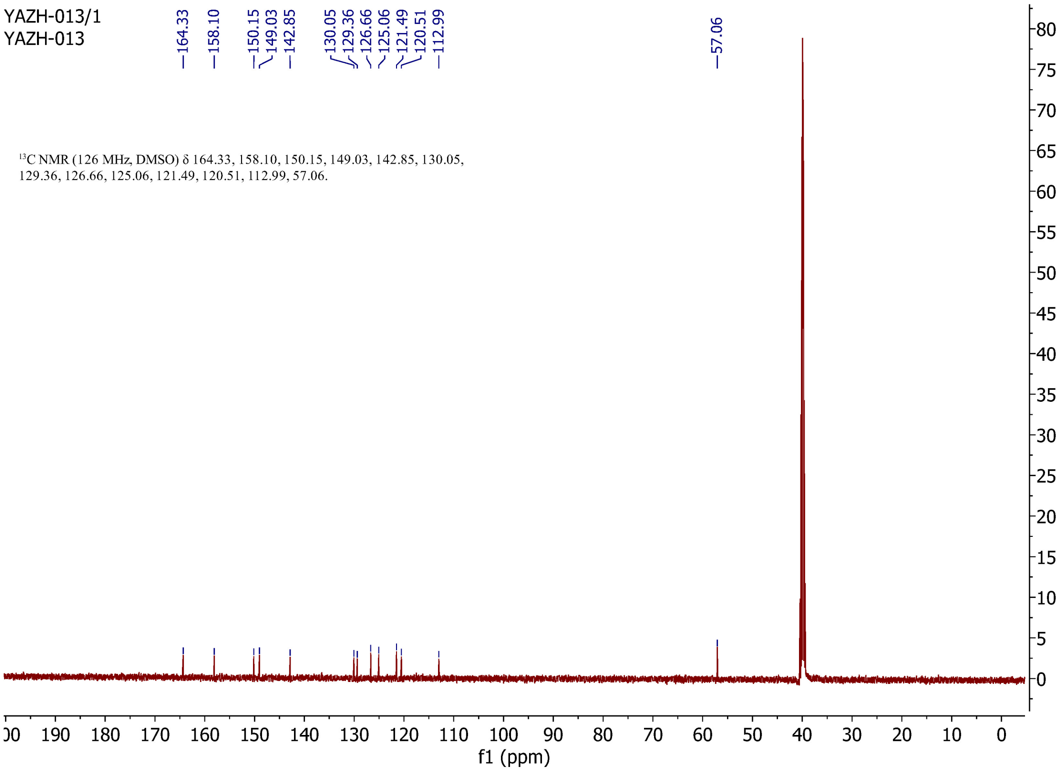

Figure S32 ^{13}C NMR spectrum of compound NPD-1181

Medicinal Chemistry
Vrije Universiteit Amsterdam

Acquired by	: Admin
Date Acquired	: 10/5/2019 3:42:14 PM
Sample Name	: YAZH-014
Sample ID	:
Tray#	: 1
Vial#	: 36
Injection Volume	: 3
Data File	: C:\LabSolutions\Data\2019\2019-wk19\YAZH-014.lcd
Background File	: blanco 10052019.lcd
Method File	: Method SCAN ACID standard.lcm
Report Format	: DefaultLCMS.lcr
Tuning File	: C:\LabSolutions\Tuning\Tuning-ESI-pos-neg01072015a.lct
Processed by	: Admin
Modified Date	: 10/5/2019 4:20:45 PM

PDA Graph

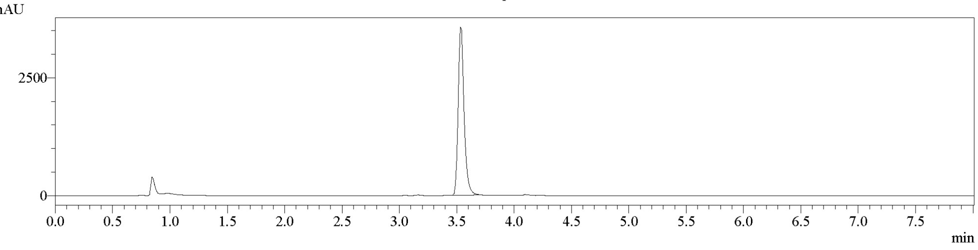

PeakTable

PDA Ch1 254nm 4nm

Peak#	Name	Ret. Time	Area	Area %
1		3.530	12764741	100.000

MS Chromatogram
YAZH-014 C:\LabSolutions\Data\2019\2019-wk19\YAZH-014.lcd

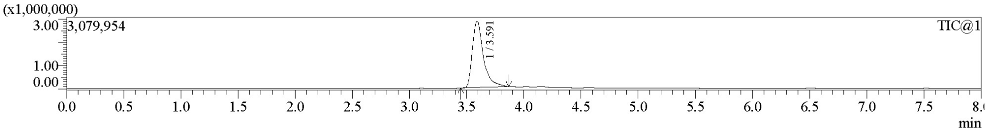

MS Spectrum Graph

#:1 Ret.Time:Averaged 3.580-3.600(Scan#:359-361)
BG Mode:Calc 3.450<->3.870(346<->388)
Mass Peaks:8 Base Peak:281.00(1768940) Polarity:Pos Segment1 - Event1

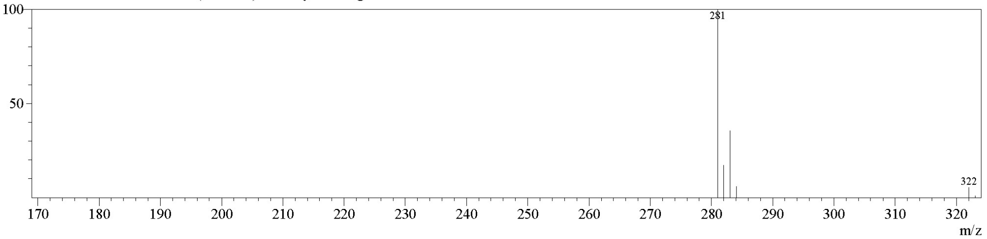

MS Spectrum Table

#:1 Ret.Time:
BG Mode:Calc 3.450<->3.870(346<->388)
Mass Peaks:8 Base Peak:281.00(1768940) Polarity:Pos Segment1 - Event1

#	m/z	Abs.Inten.	Rel.Inten.	Charge	Polarity	Monoisotopic
1	169.00	18379	1.04			
2	281.00	1768940	100.00			
3	282.00	304788	17.23			
4	283.00	629665	35.60			
5	284.05	104172	5.89			
6	322.05	94099	5.32			
7	323.05	18549	1.05			
8	324.05	33808	1.91			

Figure S33 LCMS spectrum of compound NPD-1182

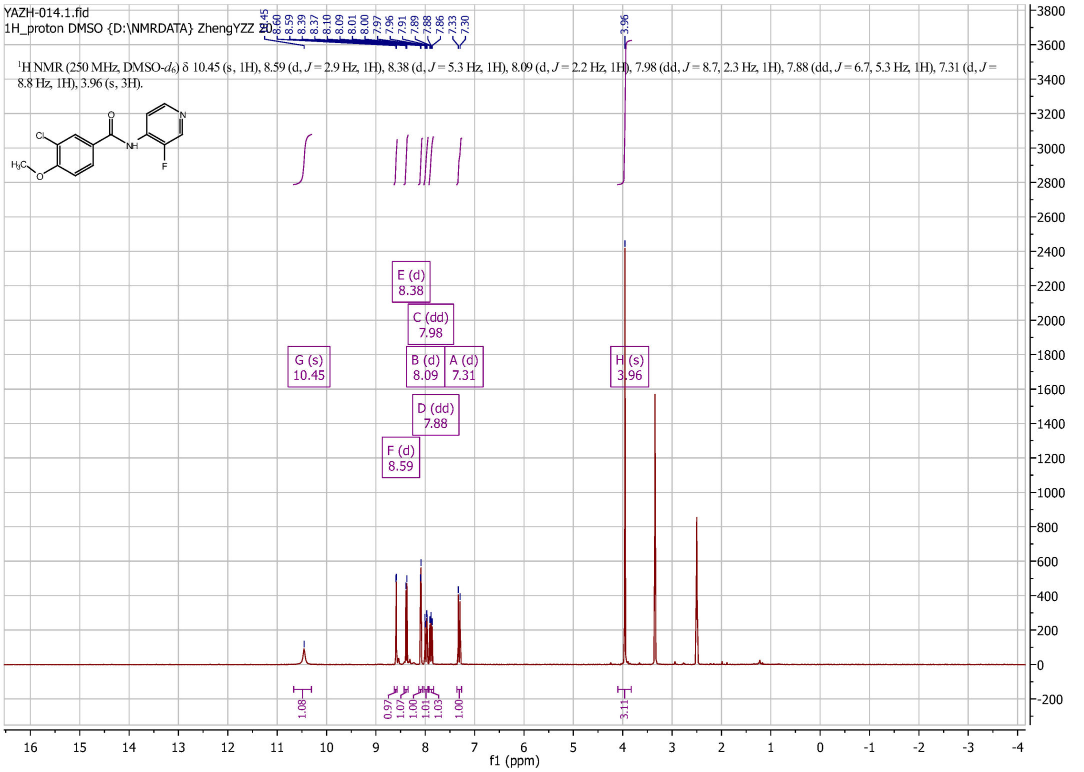

Figure S34 ^{1}H NMR spectrum of compound NPD-1182

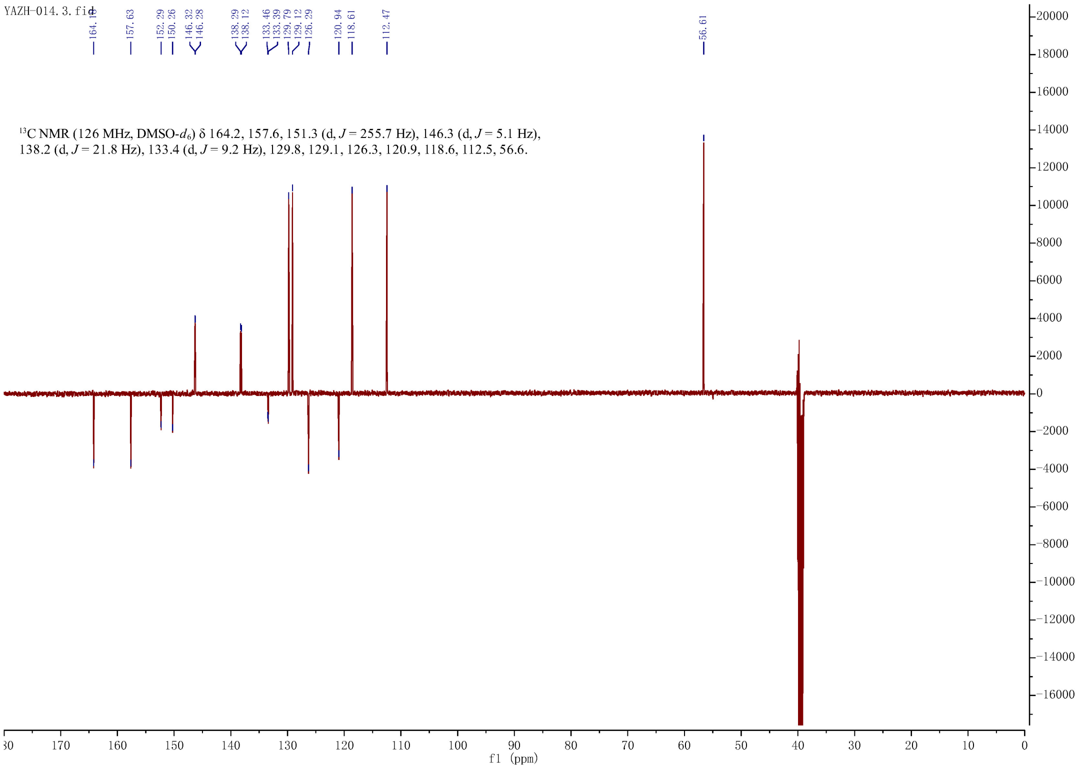

Figure S35 ^{13}C NMR spectrum of compound NPD-1182

Acquired by	: Admin
Date Acquired	: 10/15/2015 6:23:36 PM
Sample Name	: YAZH-015
Sample ID	:
Tray#	: 1
Vial#	: 13
Injection Volume	: 1
Data File	: C:\LabSolutions\Data\2015\2015-wk42\YAZH-015.lcd
Background File	: blanco 16102016.lcd
Method File	: Method SCAN ACID standard.lcm
Report Format	: DefaultLCMS.lcr
Tuning File	: C:\LabSolutions\Tuning File\Tuning-ESI-pos-neg01072015.lct
Processed by	: Admin
Modified Date	: 10/16/2015 9:42:53 AM

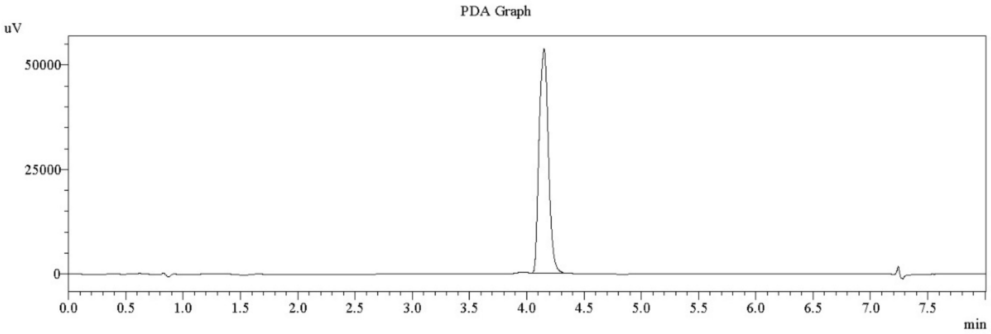

PDA Ch1 254nm 4nm

Peak#	Name	Ret. Time	Area	Area %
1		4.146	301413	100.000

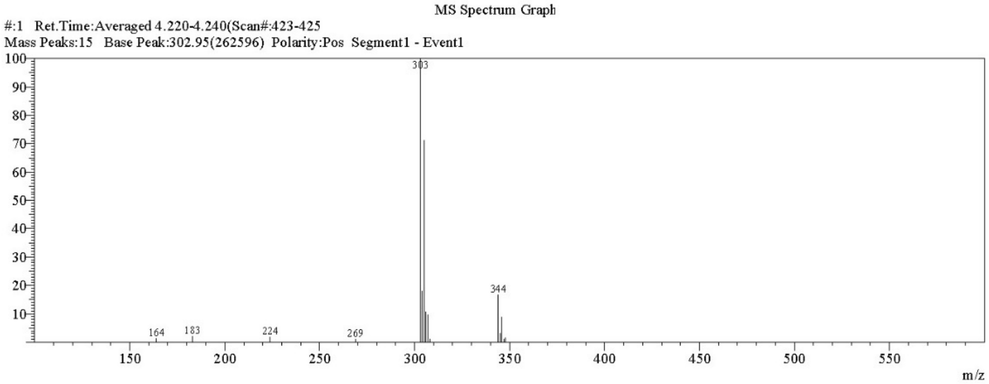

#:1 Ret.Time:
BG Mode:Calc 4.100<->4.450(411<->446)
Mass Peaks:15 Base Peak:302.95(262596) Polarity:Pos Segment1 - Event1

#	m/z	Abs.Inten.	Rel.Inten.	Charge	Polarity	Monoisotopic
1	164.00	3498	1.33			
2	182.95	5134	1.96			
3	223.90	4892	1.86			
4	268.85	2932	1.12			
5	302.95	262596	100.00			
6	303.95	46888	17.86			
7	304.95	186816	71.14			
8	305.85	27745	10.57			
9	306.95	25124	9.57			
10	308.05	2683	1.02			
11	343.95	43986	16.75			
12	345.15	8229	3.13			
13	345.90	23230	8.85			
14	347.00	2967	1.13			
15	348.00	3792	1.44			

Figure S36 LCMS spectrum of compound NPD-1183

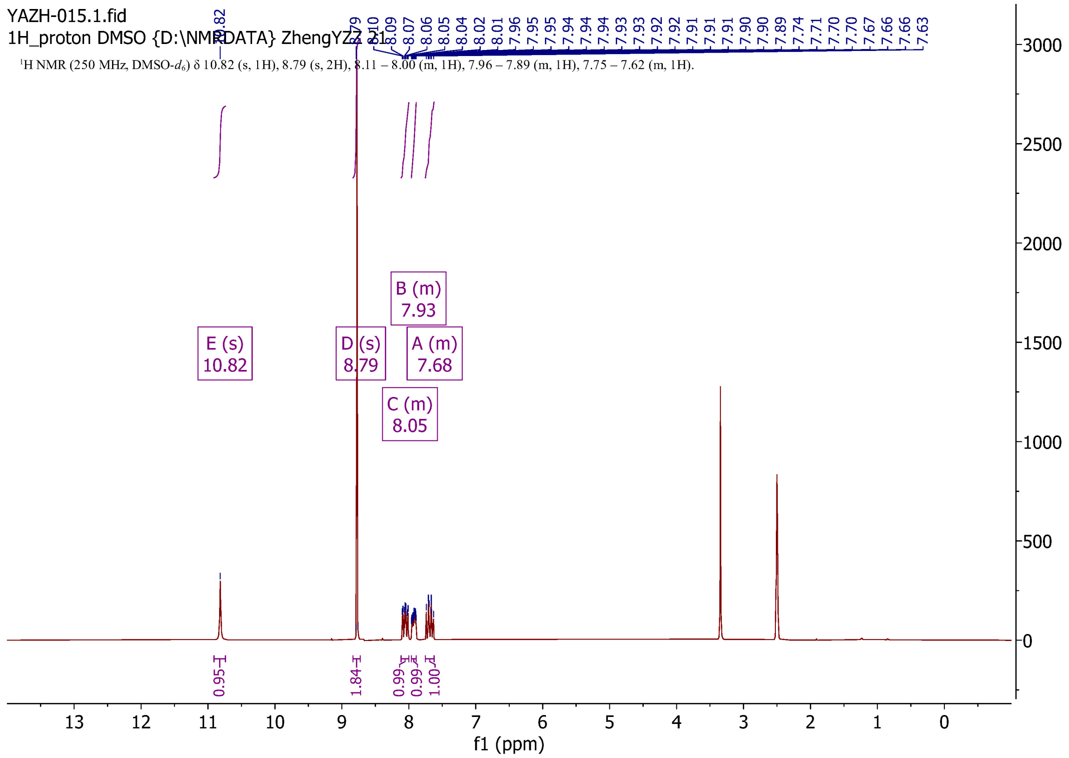

Figure S37 ^{1}H NMR spectrum of compound NPD-1183

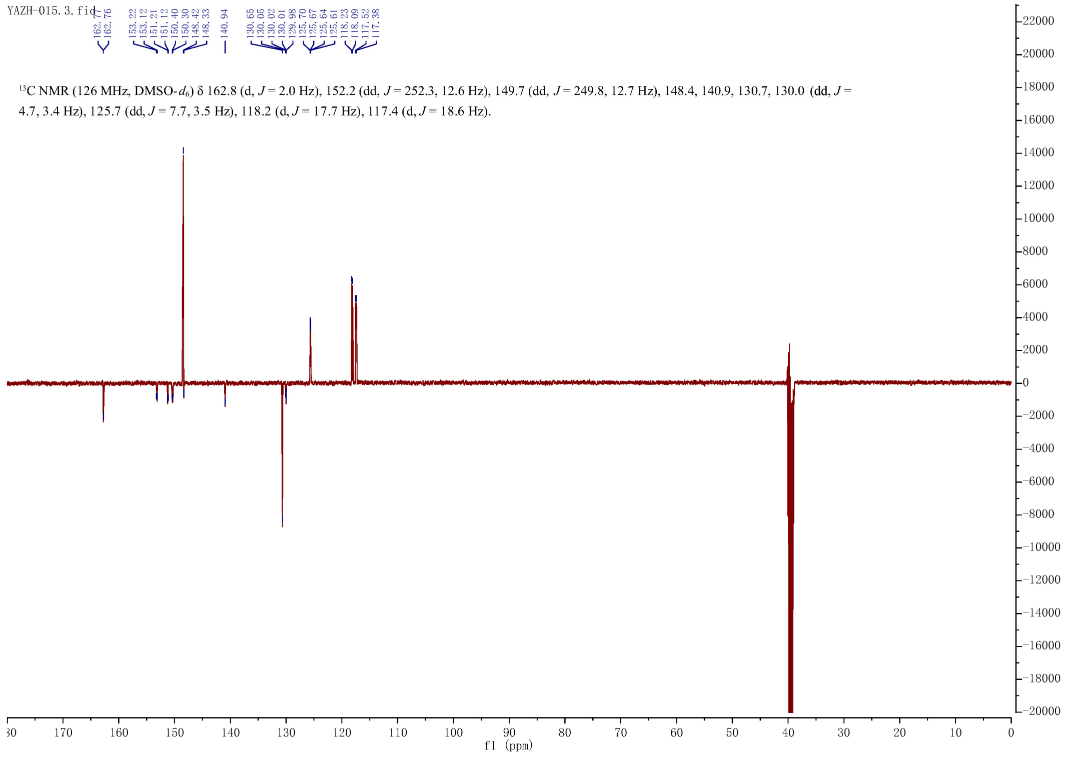

Figure S38 ^{13}C NMR spectrum of compound NPD-1183

Acquired by	: Admin
Date Acquired	: 10/15/2015 6:32:16 PM
Sample Name	: YAZH-016
Sample ID	:
Tray#	: 1
Vial#	: 14
Injection Volume	: 1
Data File	: C:\LabSolutions\Data\2015\2015-wk42\YAZH-016.lcd
Background File	: blanco 16102016.lcd
Method File	: Method SCAN ACID standard.lcm
Report Format	: DefaultLCMS.lcr
Tuning File	: C:\LabSolutions\Tuning File\Tuning-ESI-pos-neg01072015.lct
Processed by	: Admin
Modified Date	: 10/16/2015 9:44:13 AM

PDA Graph

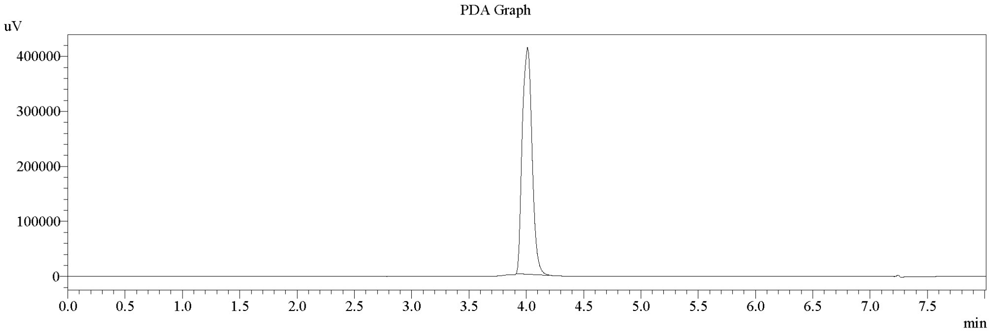

PDA Ch1 254nm 4nm

Peak#	Name	Ret. Time	Area	Area %
1		4.006	2450571	100.000

MS Spectrum Graph

#:1 Ret.Time:Averaged 4.080-4.100(Scan#:409-411)
Mass Peaks:7 Base Peak:268.95(2264436) Polarity:Pos Segment1 - Event1

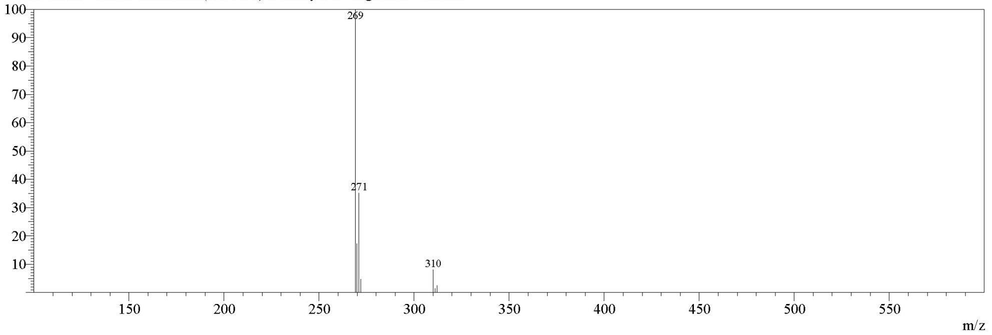

MS Spectrum Table

#:1 Ret.Time:
BG Mode:Calc 3.920<->4.510(393<->452
Mass Peaks:7 Base Peak:268.95(2264436) Polarity:Pos Segment1 - Event

#	m/z	Abs.Inten.	Rel.Inten.	Charge	Polarity	Monoisotopic
1	268.95	2264436	100.00			
2	269.95	390369	17.24			
3	270.95	795370	35.12			
4	272.00	109158	4.82			
5	310.00	182863	8.08			
6	311.05	33790	1.49			
7	312.00	55214	2.44			

Figure S39 LCMS spectrum of compound NPD-1184

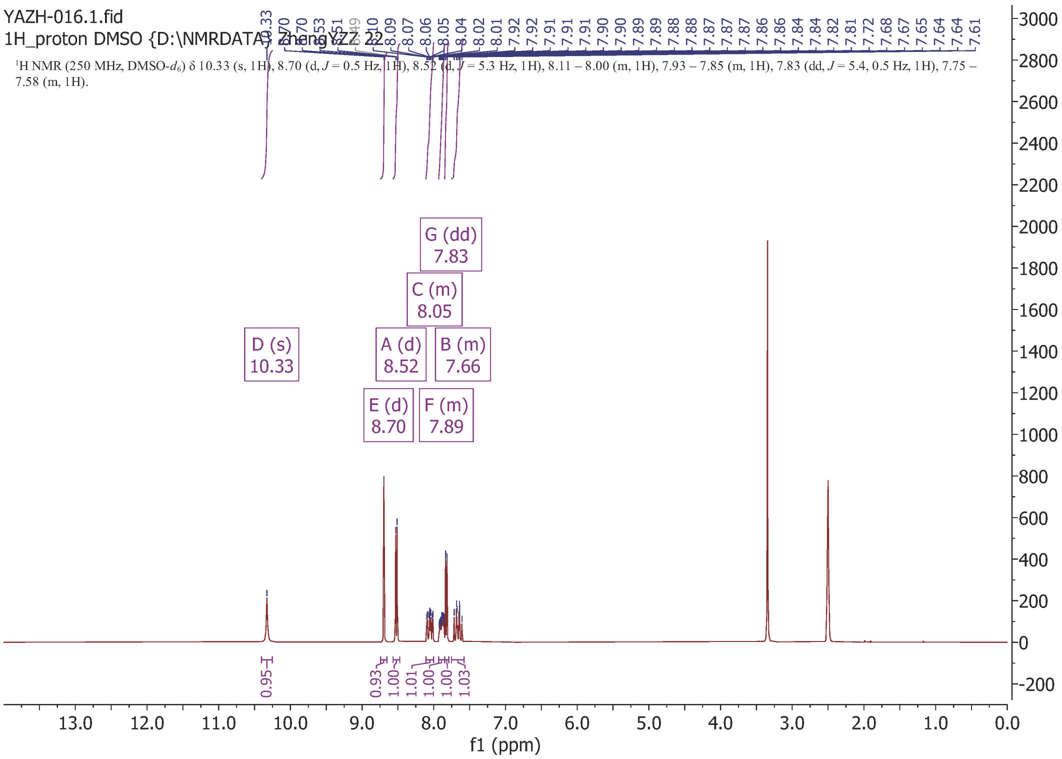

Figure S40 ¹H NMR spectrum of compound NPD-1184

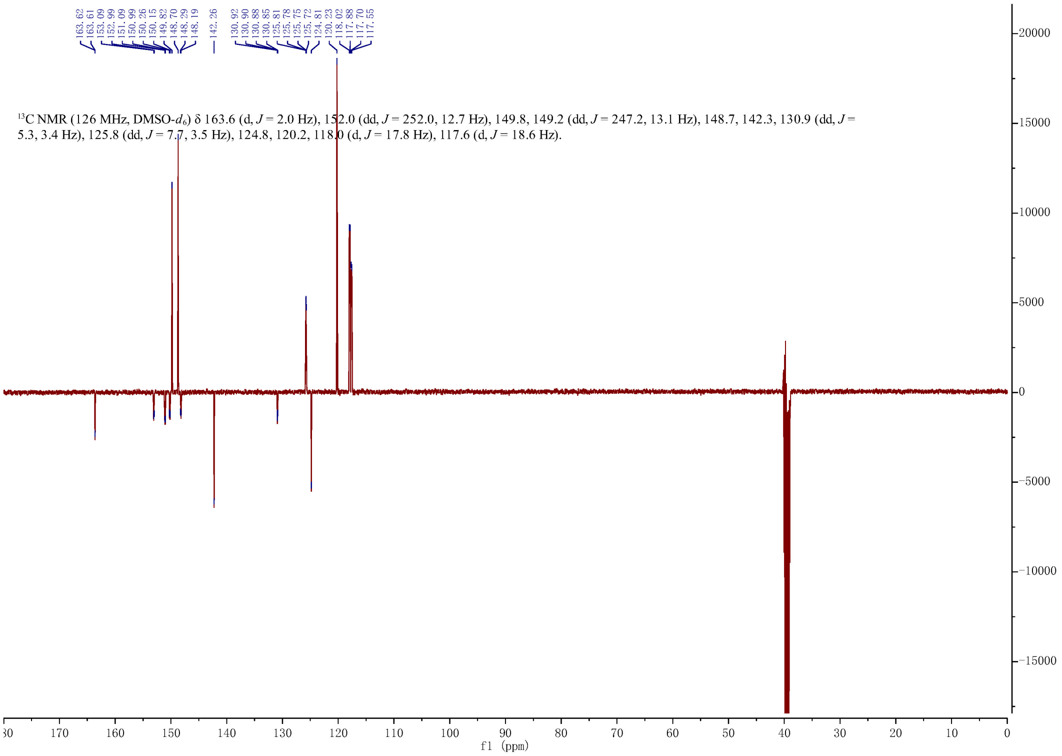

Figure S41 ¹³C NMR spectrum of compound NPD-1184

Acquired by : Admin
Date Acquired : 10/15/2015 6:40:56 PM
Sample Name : YAZH-017
Sample ID :
Tray# : 1
Vial# : 15
Injection Volume : 1
Data File : C:\LabSolutions\Data\2015\2015-wk42\YAZH-017.lcd
Background File : blanco 16102016.lcd
Method File : Method SCAN ACID standard.lcm
Report Format : DefaultLCMS.lcr
Tuning File : C:\LabSolutions\Tuning File\Tuning-ESI-pos-neg01072015.lct
Processed by : Admin
Modified Date : 10/16/2015 9:45:29 AM

PDA Graph

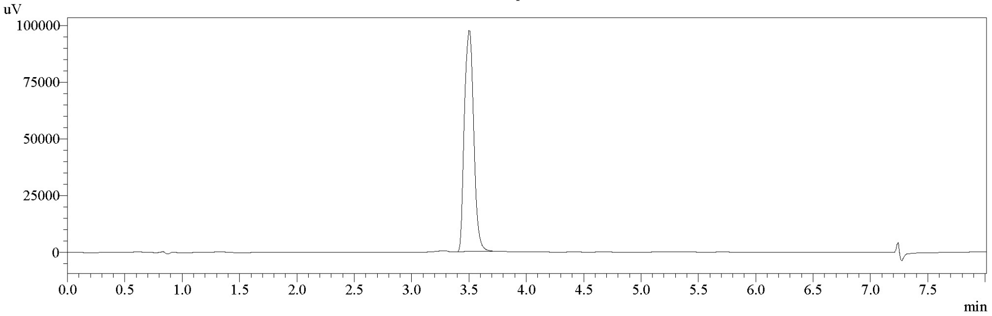

PDA Ch1 254nm 4nm

Peak#	Name	Ret. Time	Area	Area %
1		3.498	548298	100.000

MS Spectrum Graph

#:1 Ret.Time:Averaged 3.570-3.590(Scan#:358-360)
Mass Peaks:5 Base Peak:252.95(874331) Polarity:Pos Segment1 - Event1

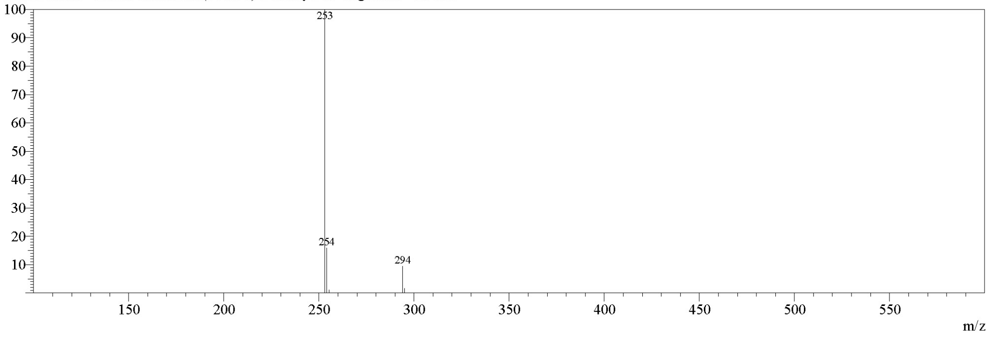

MS Spectrum Table

#:1 Ret.Time:
BG Mode:Calc 3.450<->3.780(346<->379
Mass Peaks:5 Base Peak:252.95(874331) Polarity:Pos Segment1 - Event

#	m/z	Abs.Inten.	Rel.Inten.	Charge	Polarity	Monoisotopic
1	252.95	874331	100.00			
2	254.00	138589	15.85			
3	255.25	9793	1.12			
4	294.00	82311	9.41			
5	294.90	14439	1.65			

Figure S42 LCMS spectrum of compound NPD-1185

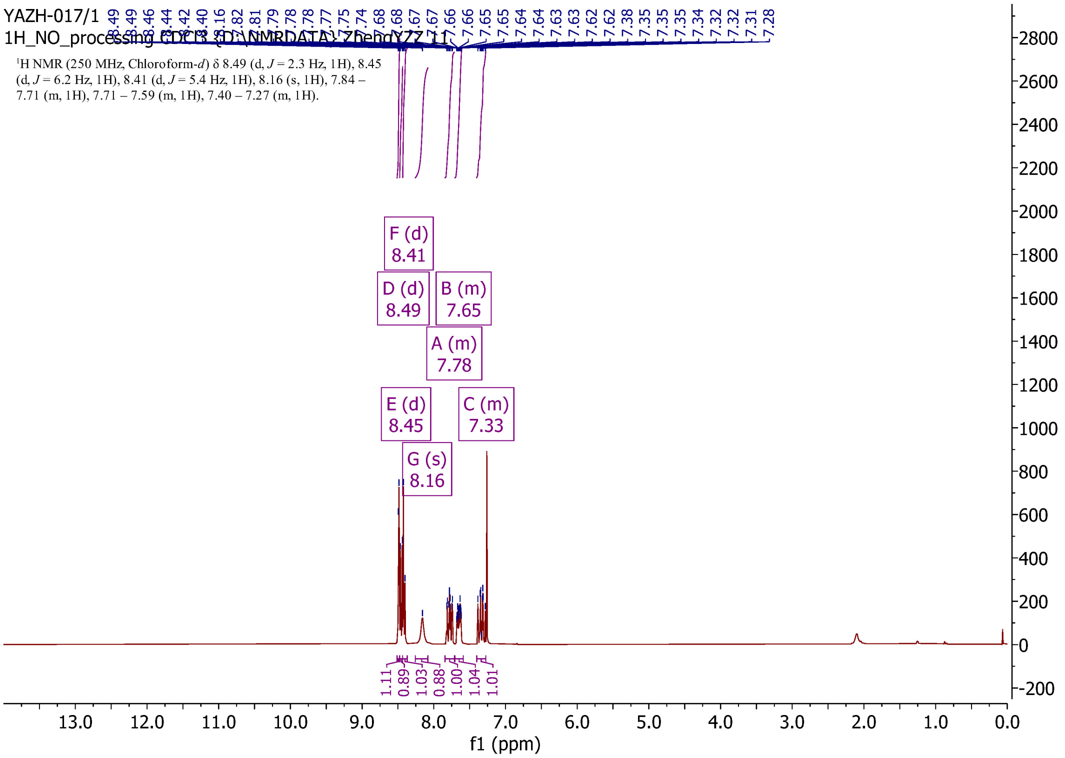

Figure S43 ^{1}H NMR spectrum of compound NPD-1185

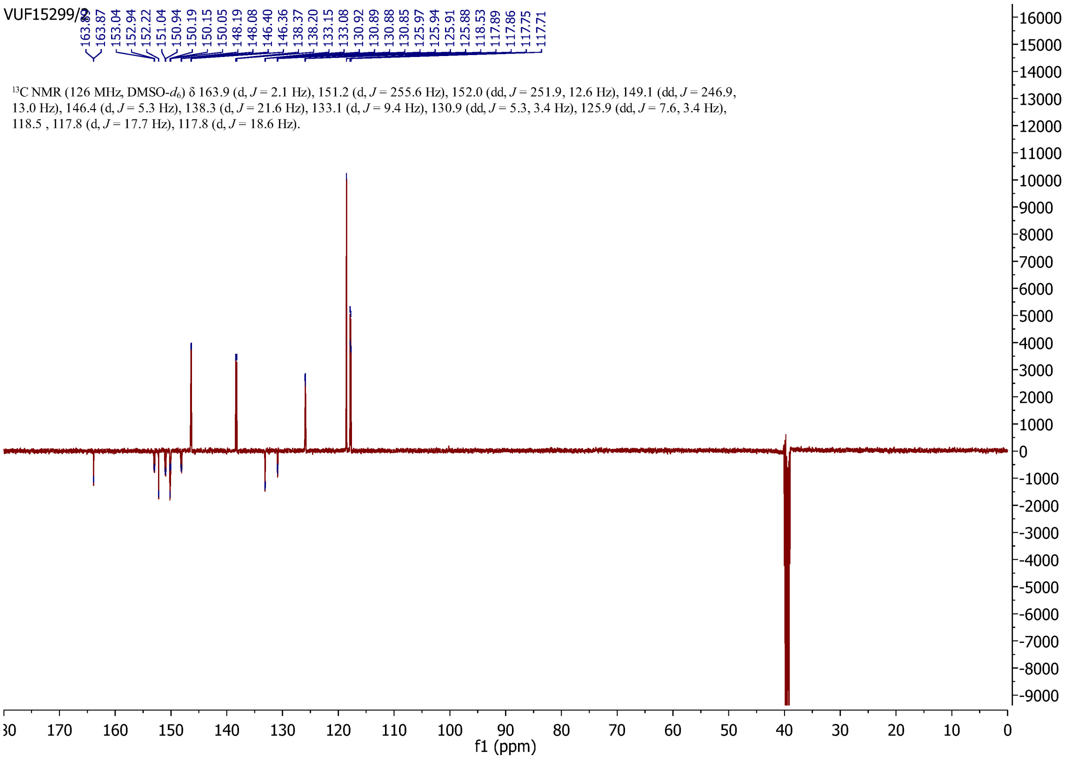

Figure S44 ^{13}C NMR spectrum of compound NPD-1185

Medicinal Chemistry
Vrije Universiteit Amsterdam

Acquired by	: Admin
Date Acquired	: 10/5/2019 3:59:27 PM
Sample Name	: YAZH-018
Sample ID	:
Tray#	: 1
Vial#	: 37
Injection Volume	: 3
Data File	: C:\LabSolutions\Data\2019\2019-wk19\YAZH-018.lcd
Background File	: blanco 10052019.lcd
Method File	: Method SCAN ACID standard.lcm
Report Format	: DefaultLCMS.lcr
Tuning File	: C:\LabSolutions\Tuning\Tuning-ESI-pos-neg01072015a.lct
Processed by	: Admin
Modified Date	: 10/5/2019 4:21:59 PM

PDA Graph

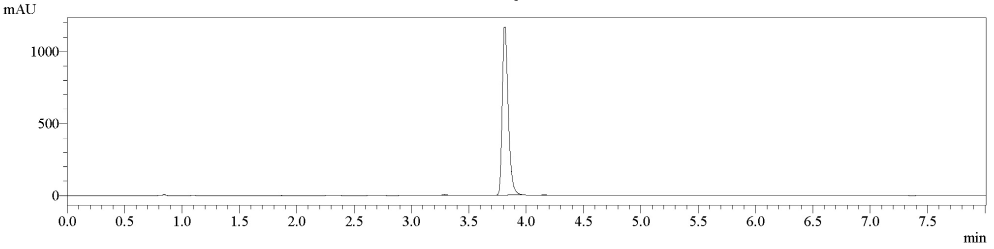

PeakTable

PDA Ch1 254nm 4nm

Peak#	Name	Ret. Time	Area	Area %
1		3.281	21243	0.502
2		3.808	4200066	99.252
3		4.153	10397	0.246

MS Chromatogram
YAZH-018 C:\LabSolutions\Data\2019\2019-wk19\YAZH-018.lcd

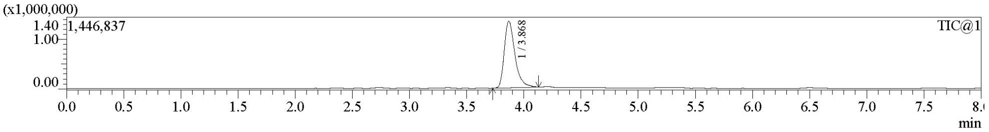

MS Spectrum Graph

#:1 Ret.Time:Averaged 3.860-3.880(Scan#:387-389)
BG Mode:Calc 3.730<->4.130(374<->414)
Mass Peaks:9 Base Peak:341.00(627181) Polarity:Pos Segment1 - Event1

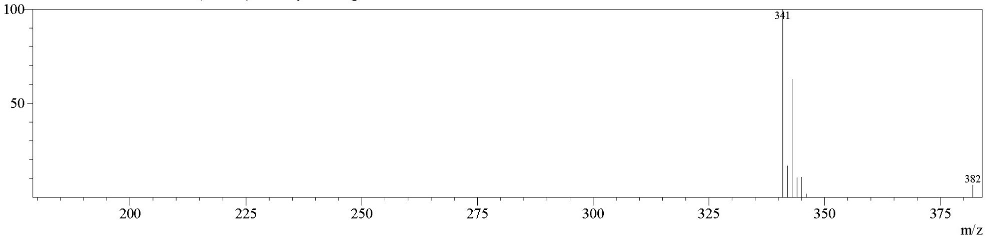

MS Spectrum Table

#:1 Ret.Time:
BG Mode:Calc 3.730<->4.130(374<->414)
Mass Peaks:9 Base Peak:341.00(627181) Polarity:Pos Segment1 - Event1

#	m/z	Abs.Inten.	Rel.Inten.	Charge	Polarity	Monoisotopic
1	179.00	23933	3.82			
2	341.00	627181	100.00			
3	342.05	105030	16.75			
4	343.00	394560	62.91			
5	344.05	65299	10.41			
6	345.05	66619	10.62			
7	346.05	11028	1.76			
8	382.00	40100	6.39			
9	384.05	27033	4.31			

Figure S45 LCMS spectrum of compound NPD-1186

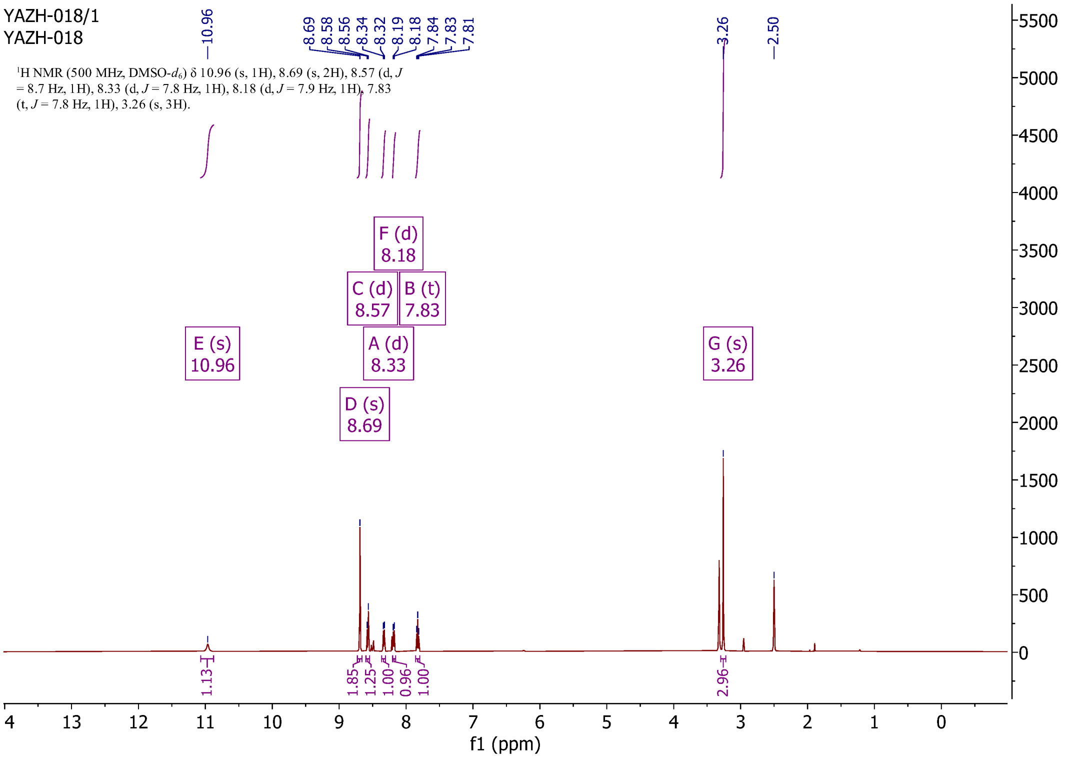

Figure S46 ^{1}H NMR spectrum of compound NPD-1186

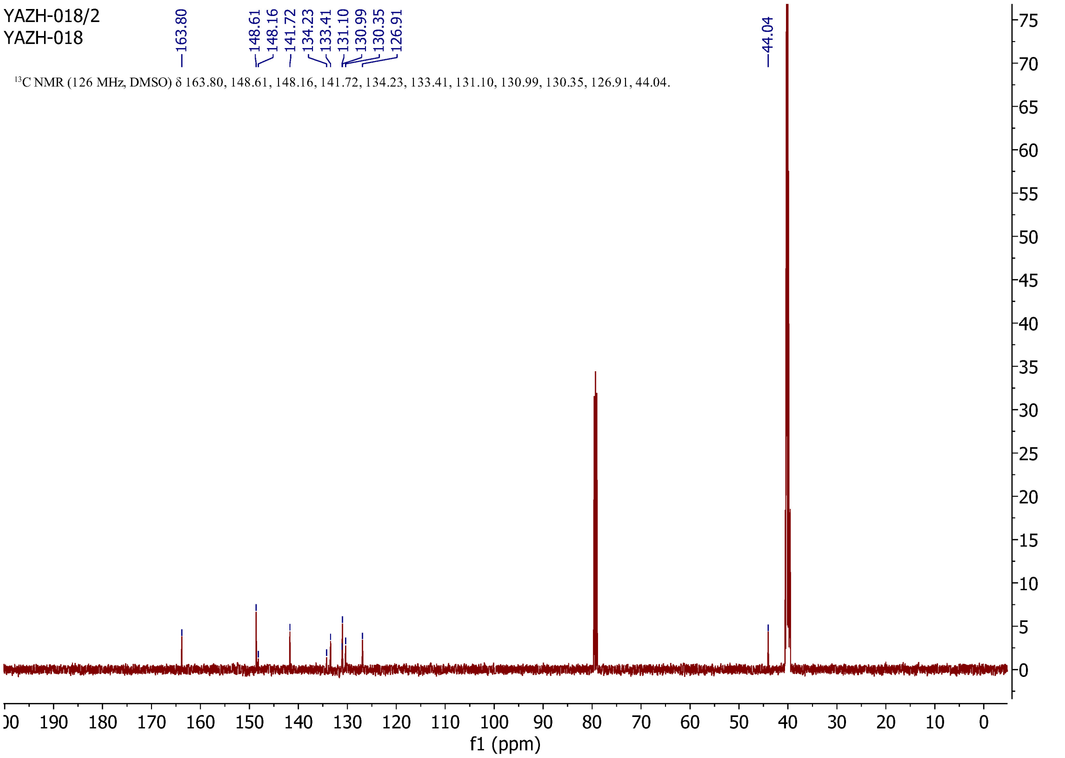

Figure S47 ^{13}C NMR spectrum of compound NPD-1186

Acquired by	: Admin
Date Acquired	: 10/15/2015 7:24:14 PM
Sample Name	: YAZH-022
Sample ID	:
Tray#	: 1
Vial#	: 20
Injection Volume	: 1
Data File	: C:\LabSolutions\Data\2015\2015-wk42\YAZH-022.lcd
Background File	: blanco 16102016.lcd
Method File	: Method SCAN ACID standard.lcm
Report Format	: DefaultLCMS.lcr
Tuning File	: C:\LabSolutions\Tuning File\Tuning-ESI-pos-neg01072015.lct
Processed by	: Admin
Modified Date	: 10/16/2016 9:58:56 AM

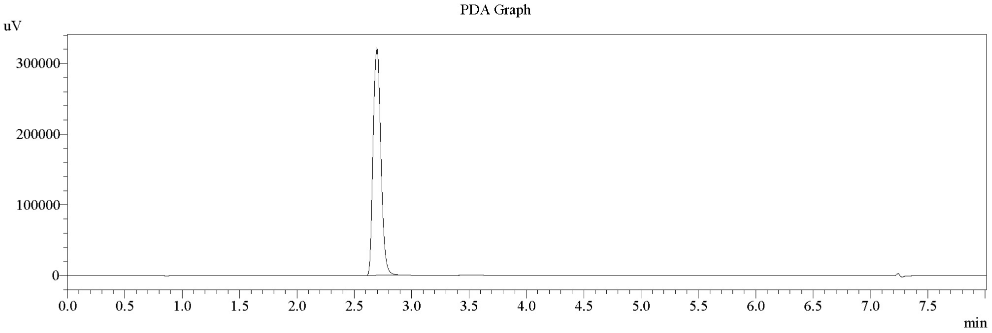

PDA Ch1 254nm 4nm

Peak#	Name	Ret. Time	Area	Area %
1		2.693	1518425	100.000

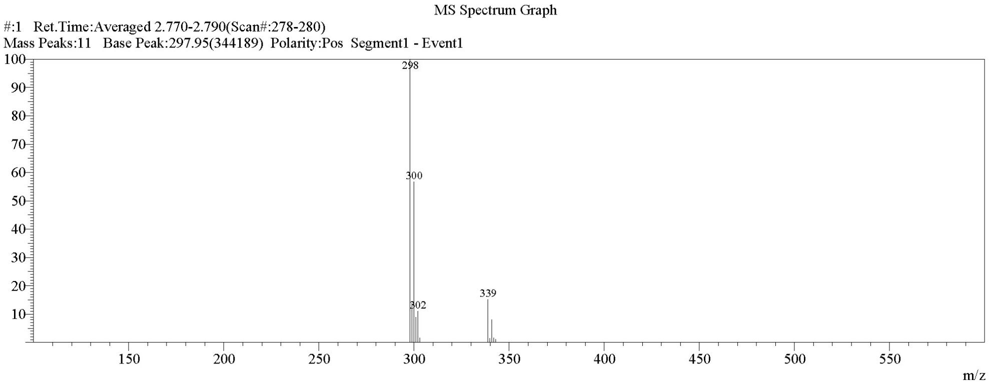

MS Spectrum Table

#:1 Ret.Time:
BG Mode:Calc 2.640<->3.010(265<->302
Mass Peaks:11 Base Peak:297.95(344189) Polarity:Pos Segment1 - Event

#	m/z	Abs.Inten.	Rel.Inten.	Charge	Polarity	Monoisotopic
1	297.95	344189	100.00			
2	299.00	47397	13.77			
3	299.95	195121	56.69			
4	300.95	30774	8.94			
5	301.90	37966	11.03			
6	303.05	5665	1.65			
7	338.90	52191	15.16			
8	339.95	4878	1.42			
9	340.95	27680	8.04			
10	341.95	5631	1.64			
11	342.90	3658	1.06			

Figure S48 LCMS spectrum of compound NPD-1187

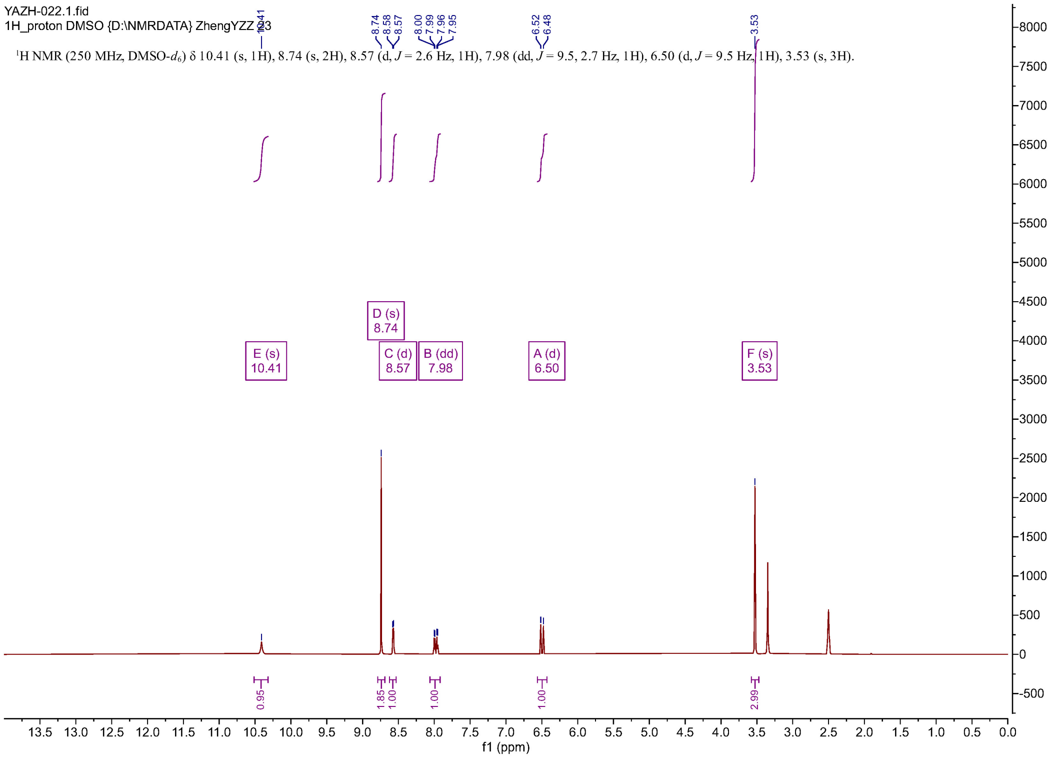

Figure S49 ^{1}H NMR spectrum of compound NPD-1187

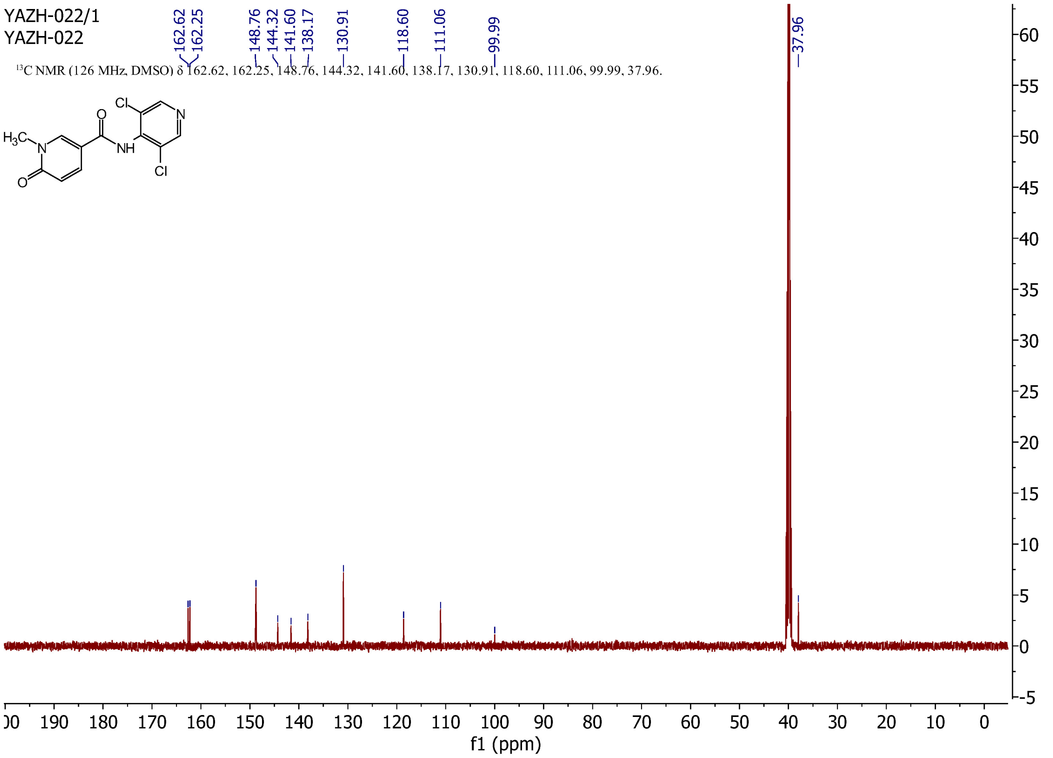

Figure S50 ^{13}C NMR spectrum of compound NPD-1187

Acquired by	: Admin
Date Acquired	: 10/15/2015 7:32:54 PM
Sample Name	: YAZH-023
Sample ID	:
Tray#	: 1
Vial#	: 21
Injection Volume	: 1
Data File	: C:\LabSolutions\Data\2015\2015-wk42\YAZH-023.lcd
Background File	: blanco 16102016.lcd
Method File	: Method SCAN ACID standard.lcm
Report Format	: DefaultLCMS.lcr
Tuning File	: C:\LabSolutions\Tuning File\Tuning-ESI-pos-neg01072015.lct
Processed by	: Admin
Modified Date	: 10/16/2016 10:00:42 AM

PDA Graph

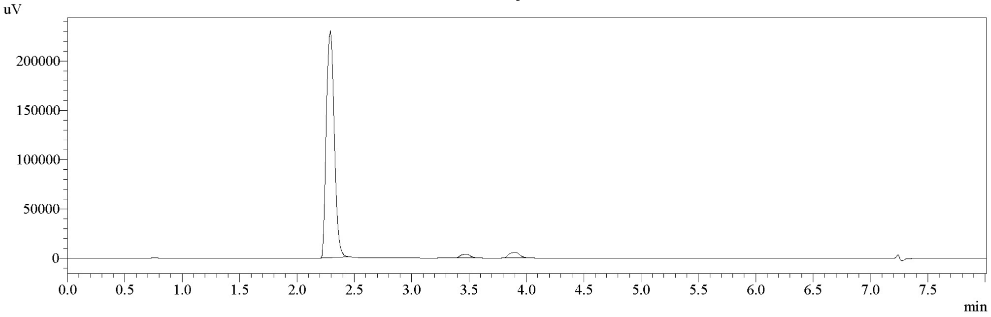

PDA Ch1 254nm 4nm

Peak#	Name	Ret. Time	Area	Area %
1		2.286	1098596	95.233
2		3.469	19853	1.721
3		3.897	35139	3.046

MS Spectrum Graph

#:1 Ret.Time:Averaged 2.350-2.370(Scan#:236-238)
Mass Peaks:5 Base Peak:264.00(981666) Polarity:Pos Segment1 - Event1

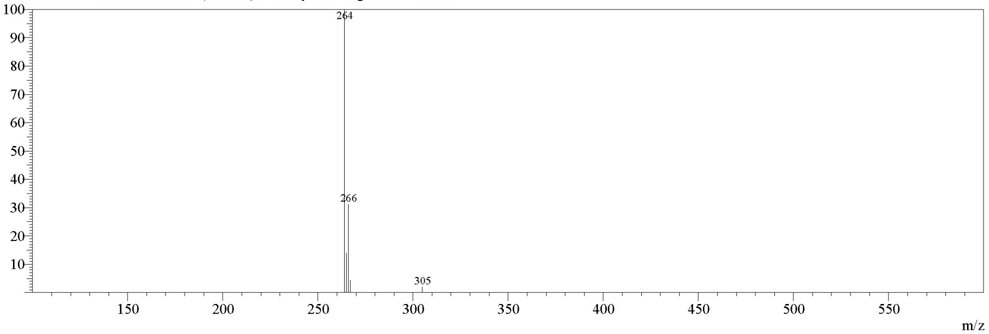

MS Spectrum Table

#:1 Ret.Time:
BG Mode:Calc 2.230<->2.610(224<->262
Mass Peaks:5 Base Peak:264.00(981666) Polarity:Pos Segment1 - Event

#	m/z	Abs.Inten.	Rel.Inten.	Charge	Polarity	Monoisotopic
1	264.00	981666	100.00			
2	265.00	137234	13.98			
3	266.00	305677	31.14			
4	266.95	42805	4.36			
5	304.95	19192	1.96			

Figure S51 LCMS spectrum of compound NPD-1188

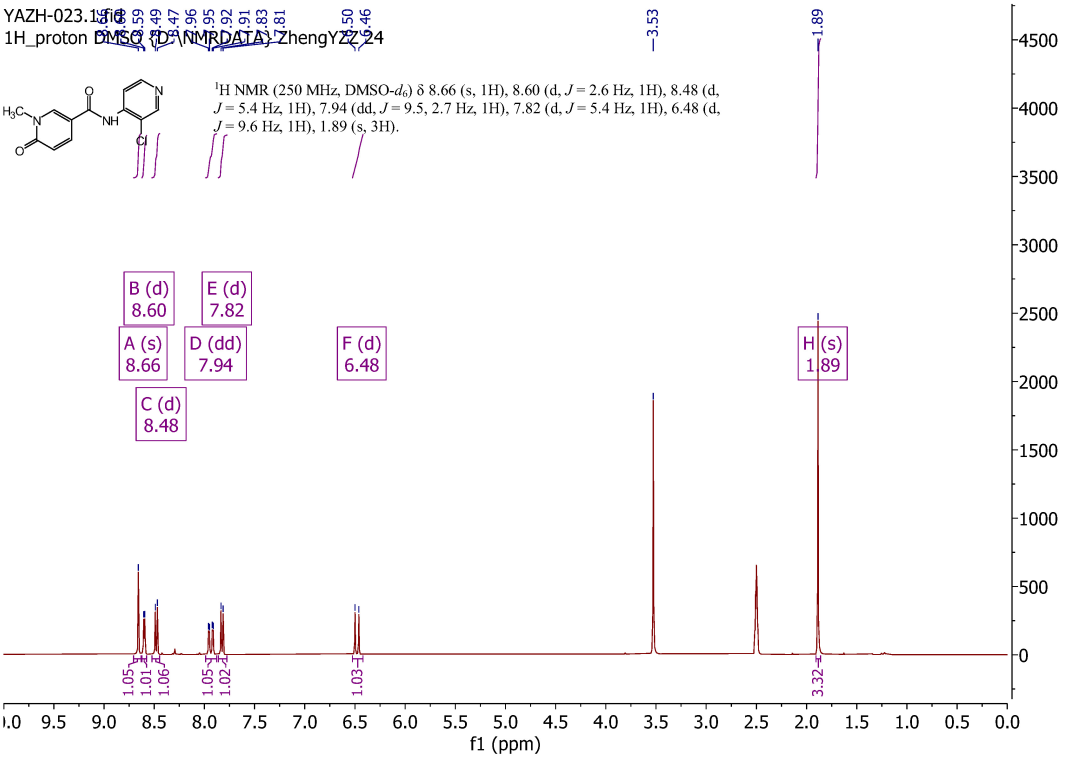

Figure S52 ^{1}H NMR spectrum of compound NPD-1188

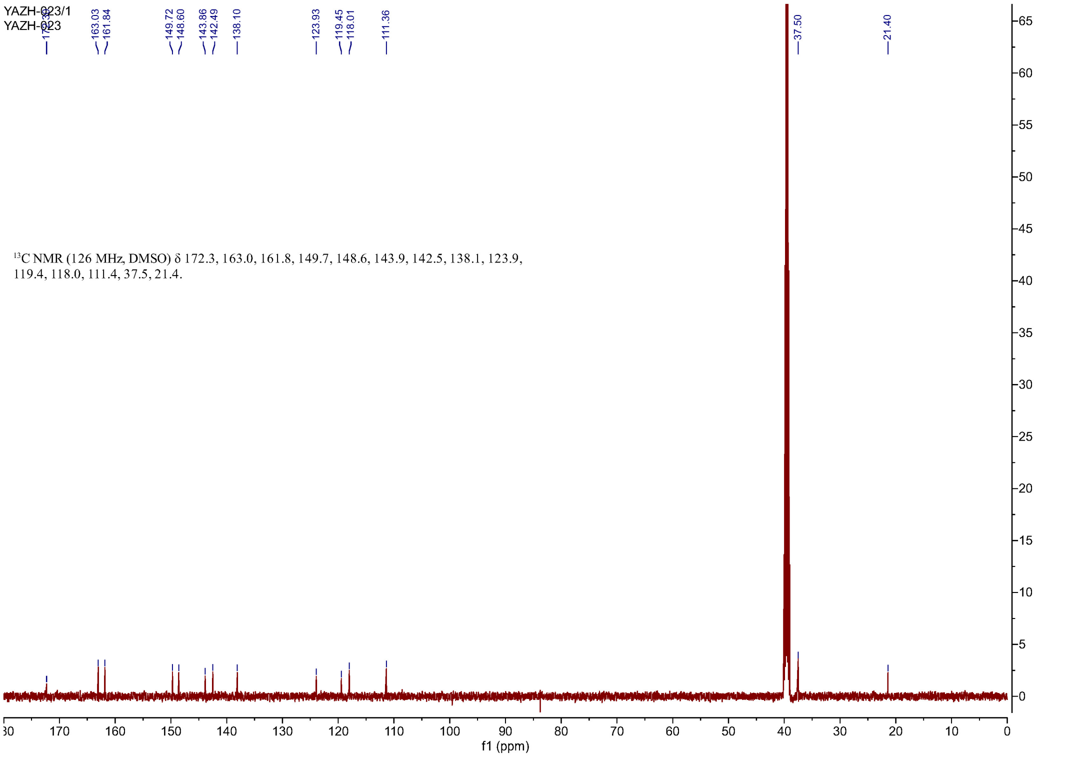

Figure S53 ^{13}C NMR spectrum of compound NPD-1188

Acquired by	: Admin
Date Acquired	: 10/5/2019 3:43:01 PM
Sample Name	: YAZH-024
Sample ID	:
Tray#	: 1
Vial#	: 28
Injection Volume	: 3
Data File	: C:\LabSolutions\Data\2019\2019-wk19\YAZH-024.lcd
Background File	: blanco 10052019.lcd
Method File	: Method SCAN ACID standard.lcm
Report Format	: DefaultLCMS.lcr
Tuning File	: C:\LabSolutions\Tuning\Tuning-ESI-pos-neg01072015a.lct
Processed by	: Admin
Modified Date	: 10/5/2019 3:53:03 PM

Medicinal Chemistry
Vrije Universiteit Amsterdam

PDA Graph

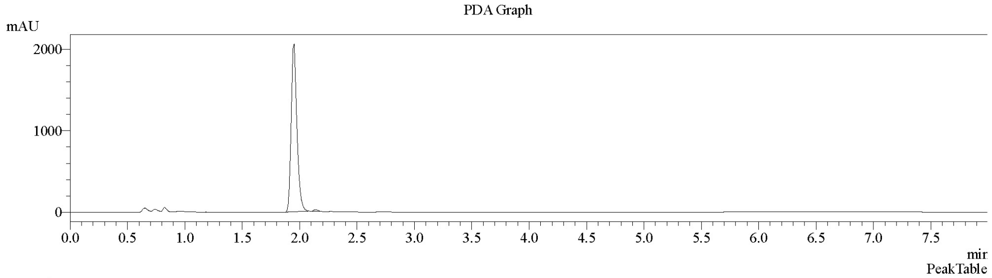

PeakTable

PDA Ch1 254nm 4nm

Peak#	Name	Ret. Time	Area	Area %
1		1.943	7186373	99.251
2		2.132	54222	0.749

MS Chromatogram
YAZH-024 C:\LabSolutions\Data\2019\2019-wk11-20\2019-wk19\YAZH-024.lcd

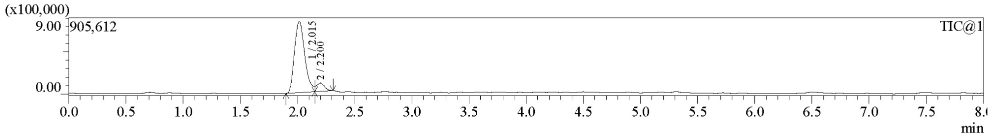

MS Spectrum Graph

#:1 Ret.Time:Averaged 2.000-2.020(Scan#:201-203)
BG Mode:Calc 1.900<->2.150(191<->216)
Mass Peaks:4 Base Peak:248.05(726813) Polarity:Pos Segment1 - Event1

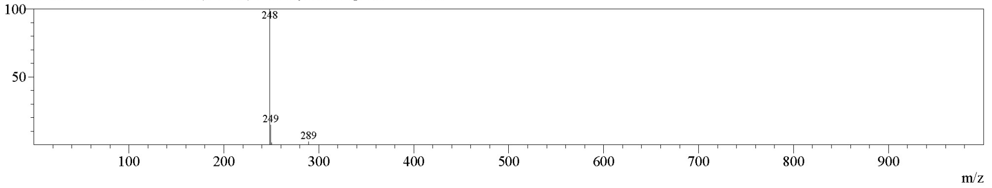

MS Spectrum Table

#:1 Ret.Time:
BG Mode:Calc 1.900<->2.150(191<->216)
Mass Peaks:4 Base Peak:248.05(726813) Polarity:Pos Segment1 - Event1

#	m/z	Abs.Inten.	Rel.Inten.	Charge	Polarity	Monoisotopic	#	m/z	Abs.Inten.	Rel.Inten.	Charge	Polarity	Monoisotopic
1	248.05	726813	100.00				3	250.05	11223	1.54			
2	249.00	107663	14.81				4	289.05	17644	2.43			

Figure S54 LCMS spectrum of compound NPD-1189

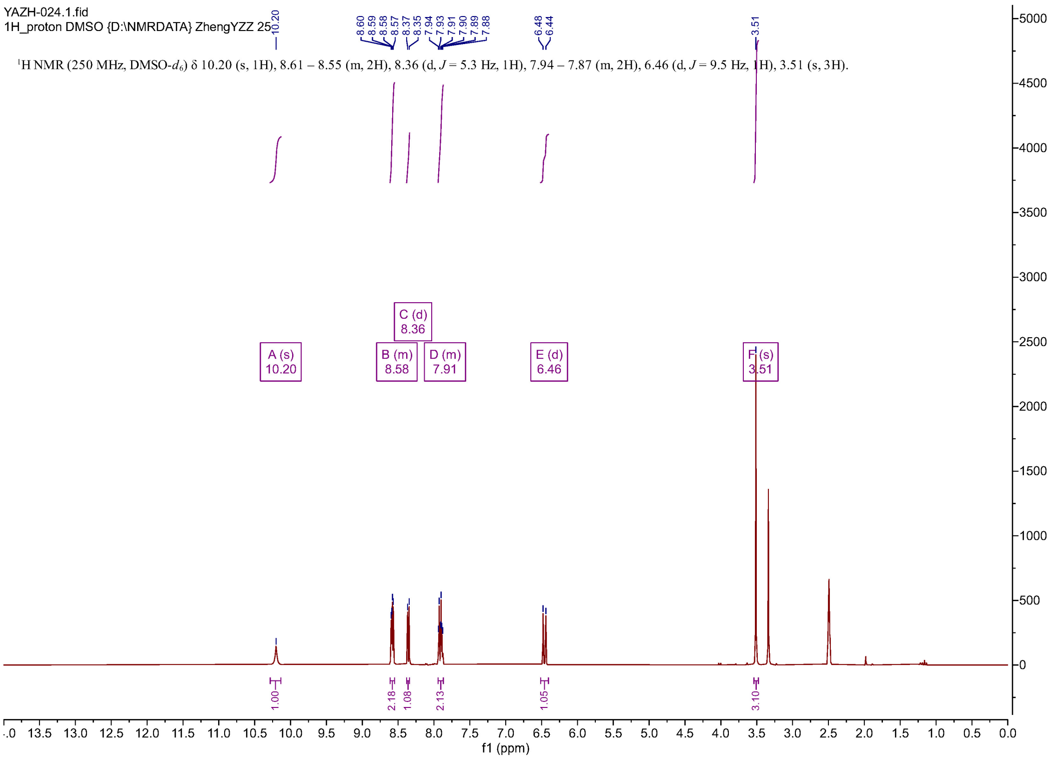

Figure S55 ^{1}H NMR spectrum of compound NPD-1189

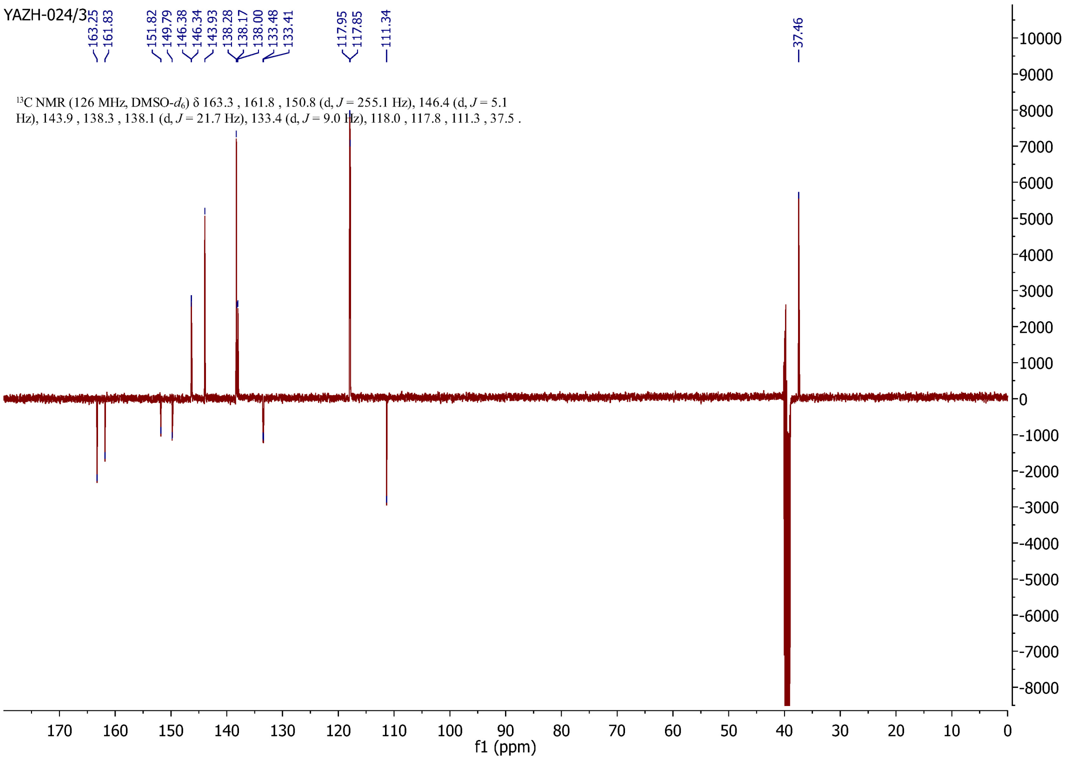

Figure S56 ^{13}C NMR spectrum of compound NPD-1189

Medicinal Chemistry
Vrije Universiteit Amsterdam

Acquired by	: Admin
Date Acquired	: 10/5/2019 4:51:39 PM
Sample Name	: YAZH-029
Sample ID	:
Tray#	: 1
Vial#	: 29
Injection Volume	: 3
Data File	: C:\LabSolutions\Data\2019\2019-wk19\YAZH-029.lcd
Background File	: blanco 10052019.lcd
Method File	: Method SCAN ACID standard.lcm
Report Format	: DefaultLCMS.lcr
Tuning File	: C:\LabSolutions\Tuning\Tuning-ESI-pos-neg01072015a.lct
Processed by	: Admin
Modified Date	: 10/5/2019 5:00:29 PM

PDA Graph

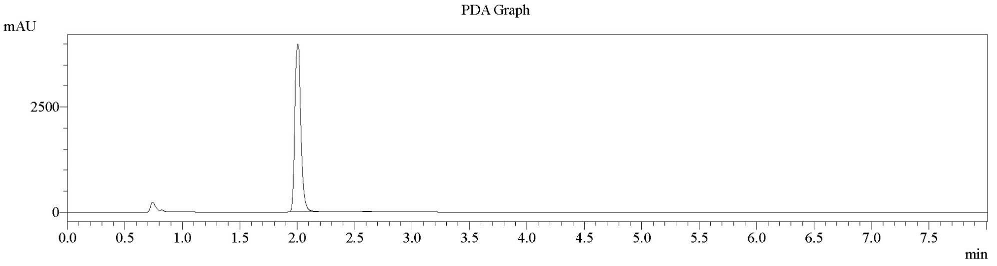

PeakTable

PDA Ch1 254nm 4nm

Peak#	Name	Ret. Time	Area	Area %
1		2.001	14532492	99.441
2		2.598	81620	0.559

MS Chromatogram
YAZH-029 C:\LabSolutions\Data\2019\2019-wk19\YAZH-029.lcd

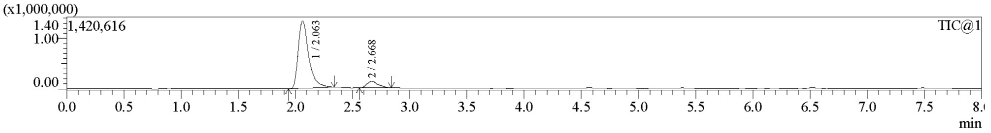

MS Spectrum Graph

#:1 Ret.Time:Averaged 2.050-2.070(Scan#:206-208)
BG Mode:Calc 1.940<->2.340(195<->235)
Mass Peaks:4 Base Peak:248.00(1149468) Polarity:Pos Segment1 - Event1

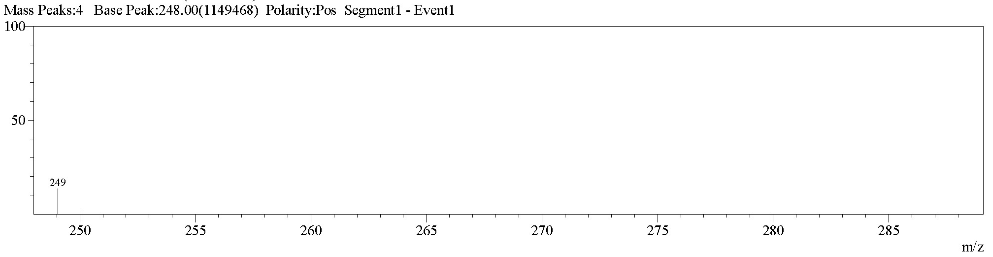

MS Spectrum Table

#:1 Ret.Time:
BG Mode:Calc 1.940<->2.340(195<->235)
Mass Peaks:4 Base Peak:248.00(1149468) Polarity:Pos Segment1 - Event1

#	m/z	Abs.Inten.	Rel.Inten.	Charge	Polarity	Monoisotopic	#	m/z	Abs.Inten.	Rel.Inten.	Charge	Polarity	Monoisotopic
1	248.00	1149468	100.00				3	250.05	16702	1.45			
2	249.05	154747	13.46				4	289.10	32360	2.82			

Figure S57 LCMS spectrum of compound NPD-1190

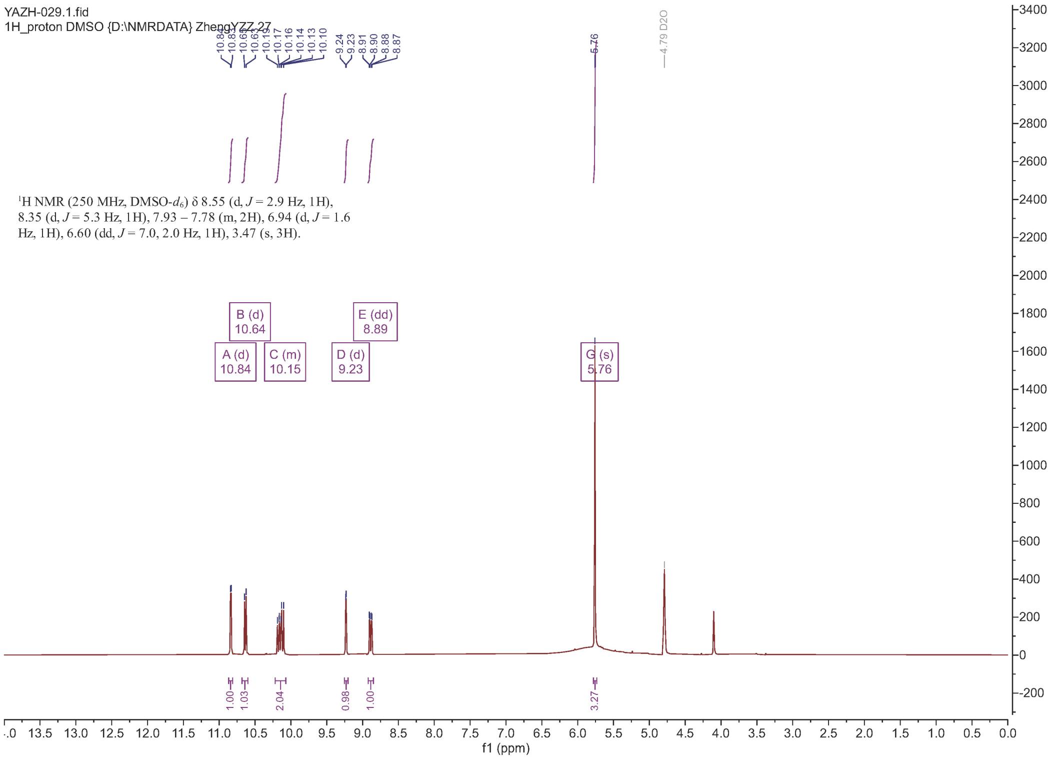

Figure S58 ^{1}H NMR spectrum of compound NPD-1190

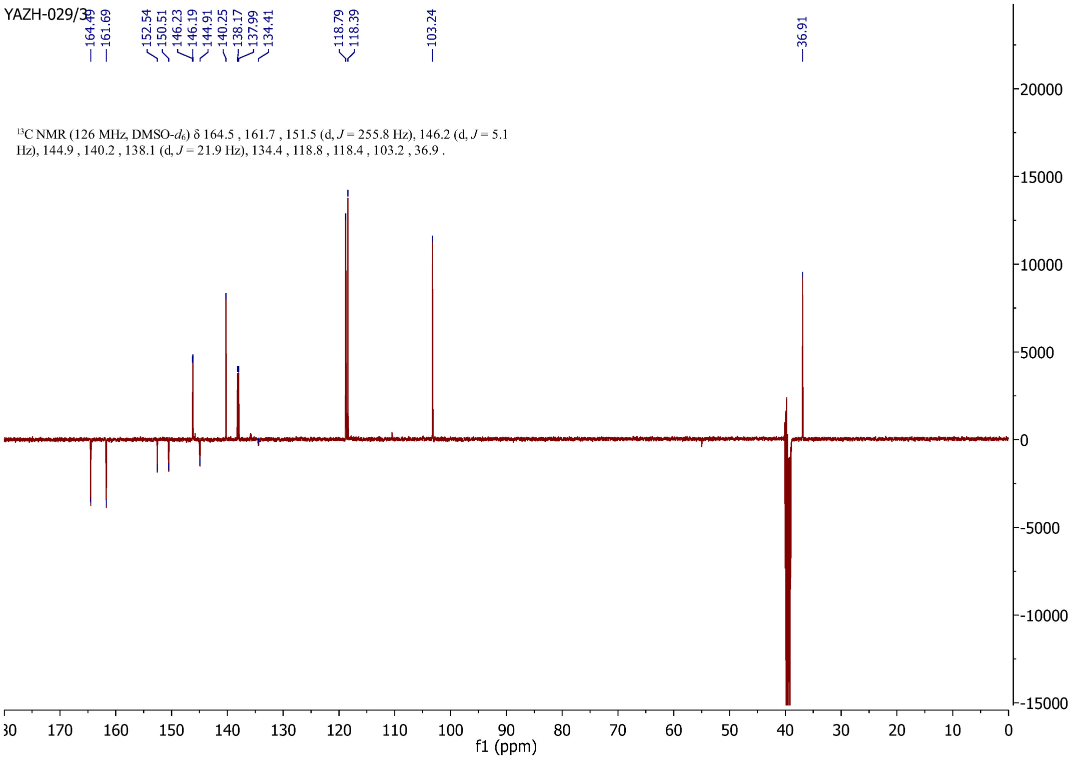

Figure S59 ^{13}C NMR spectrum of compound NPD-1190

Acquired by	: Admin
Date Acquired	: 10/15/2015 8:42:00 PM
Sample Name	: YAZH-031
Sample ID	:
Tray#	: 1
Vial#	: 29
Injection Volume	: 1
Data File	: C:\LabSolutions\Data\2015\2015-wk42\YAZH-031.lcd
Background File	: blanco 16102016.lcd
Method File	: Method SCAN ACID standard.lcm
Report Format	: DefaultLCMS.lcr
Tuning File	: C:\LabSolutions\Tuning File\Tuning-ESI-pos-neg01072015.lct
Processed by	: Admin
Modified Date	: 10/16/2015 10:14:59 AM

PDA Graph

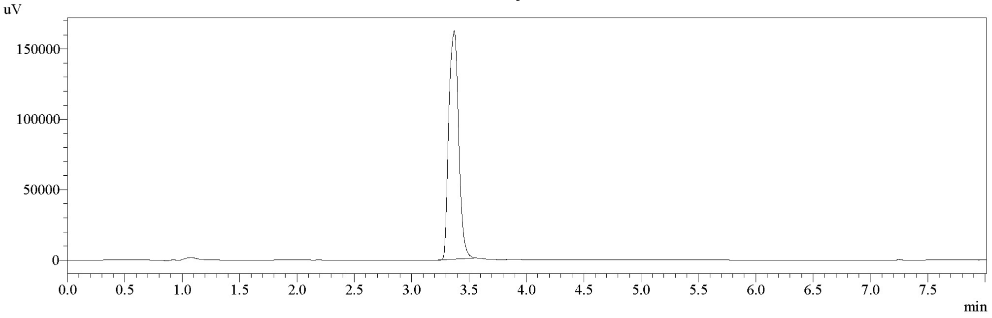

PDA Ch1 254nm 4nm

Peak#	Name	Ret. Time	Area	Area %
1		3.366	967925	100.000

MS Spectrum Graph

#:1 Ret.Time:Averaged 3.430-3.450(Scan#:344-346)
Mass Peaks:6 Base Peak:263.95(1119848) Polarity:Pos Segment1 - Event1

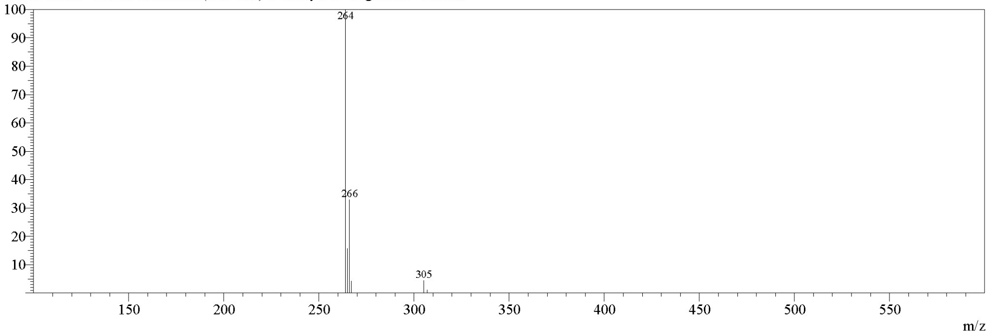

MS Spectrum Table

#:1 Ret.Time:
BG Mode:Calc 3.290<->3.650(330<->366
Mass Peaks:6 Base Peak:263.95(1119848) Polarity:Pos Segment1 - Event

#	m/z	Abs.Inten.	Rel.Inten.	Charge	Polarity	Monoisotopic
1	263.95	1119848	100.00			
2	265.00	175976	15.71			
3	266.00	368548	32.91			
4	267.05	47817	4.27			
5	305.05	50223	4.48			
6	306.95	11456	1.02			

Figure S60 LCMS spectrum of compound NPD-1191

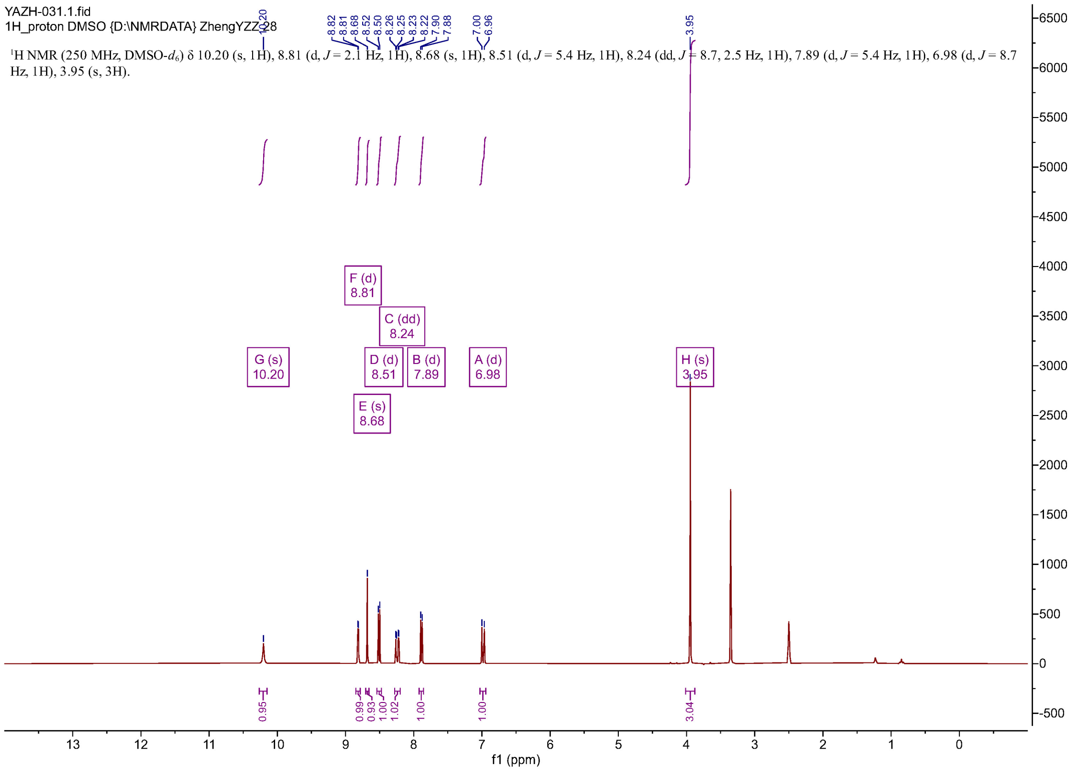

Figure S61 ¹H NMR spectrum of compound NPD-1191

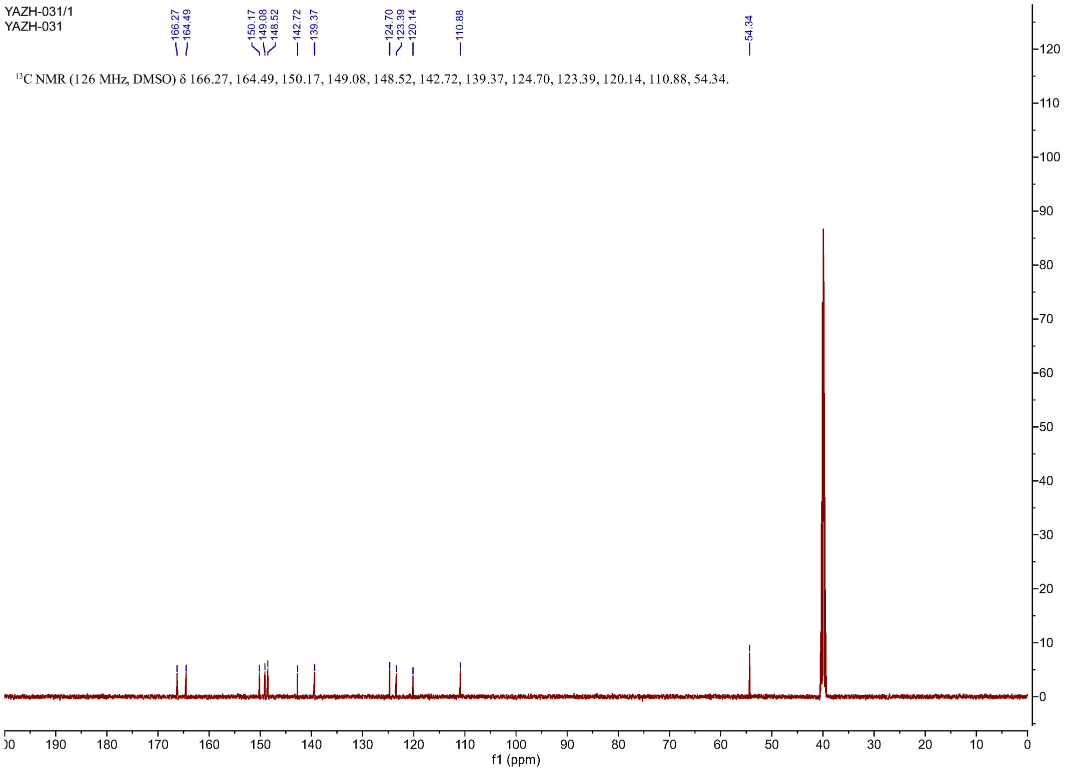

Figure S62 ¹³C NMR spectrum of compound NPD-1191

Acquired by	: Admin
Date Acquired	: 10/15/2015 9:16:40 PM
Sample Name	: YAZH-035
Sample ID	:
Tray#	: 1
Vial#	: 33
Injection Volume	: 1
Data File	: C:\LabSolutions\Data\2015\2015-wk42\YAZH-035.lcd
Background File	: blanco 16102016.lcd
Method File	: Method SCAN ACID standard.lcm
Report Format	: DefaultLCMS.lcr
Tuning File	: C:\LabSolutions\Tuning File\Tuning-ESI-pos-neg01072015.lct
Processed by	: Admin
Modified Date	: 10/16/2015 10:26:32 AM

PDA Graph

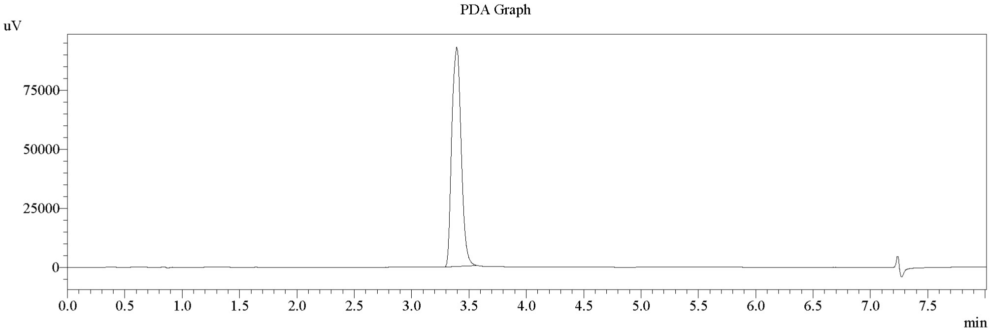

PDA Ch1 254nm 4nm

Peak#	Name	Ret. Time	Area	Area %
1		3.388	529345	100.000

MS Spectrum Graph

#:1 Ret.Time:Averaged 3.460-3.480(Scan#:347-349)
Mass Peaks:5 Base Peak:262.05(1018880) Polarity:Pos Segment1 - Event1

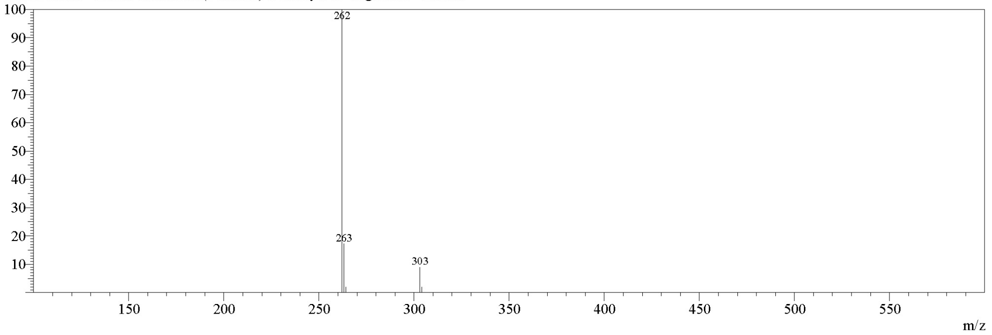

MS Spectrum Table

#:1 Ret.Time:
BG Mode:Calc 3.330<->3.700(334<->371
Mass Peaks:5 Base Peak:262.05(1018880) Polarity:Pos Segment1 - Event

#	m/z	Abs.Inten.	Rel.Inten.	Charge	Polarity	Monoisotopic
1	262.05	1018880	100.00			
2	263.05	175193	17.19			
3	264.05	20439	2.01			
4	303.05	91380	8.97			
5	304.05	20228	1.99			

Figure S63 LCMS spectrum of compound NPD-1192

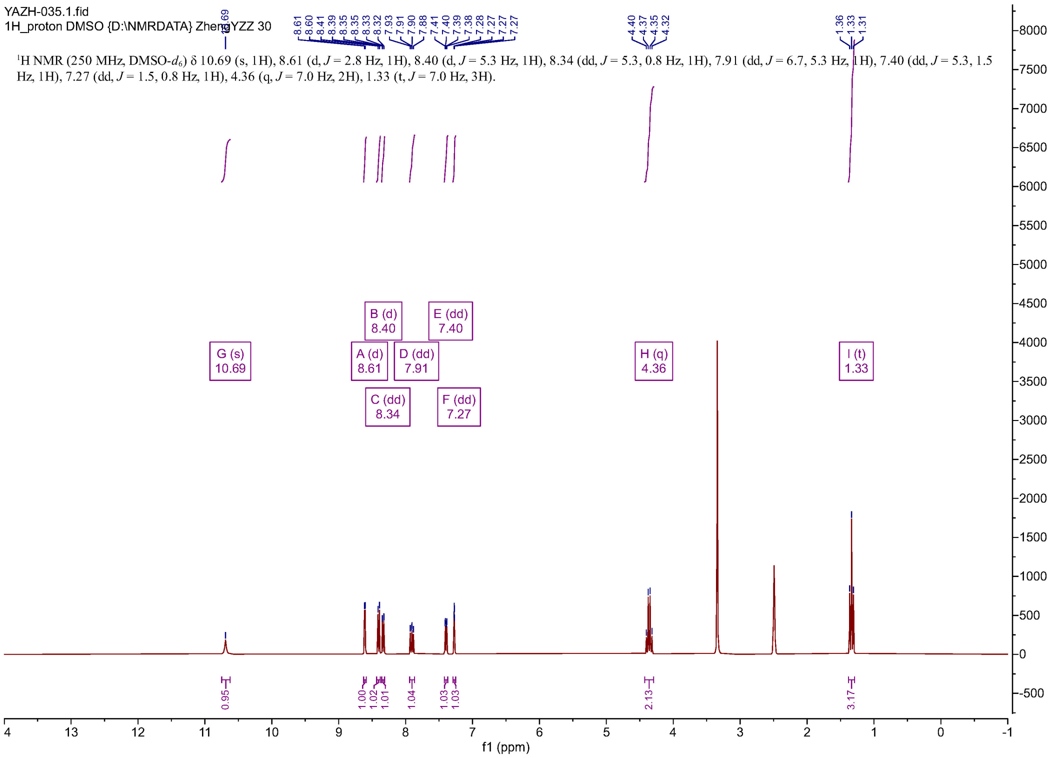

Figure S64 ¹H NMR spectrum of compound NPD-1192

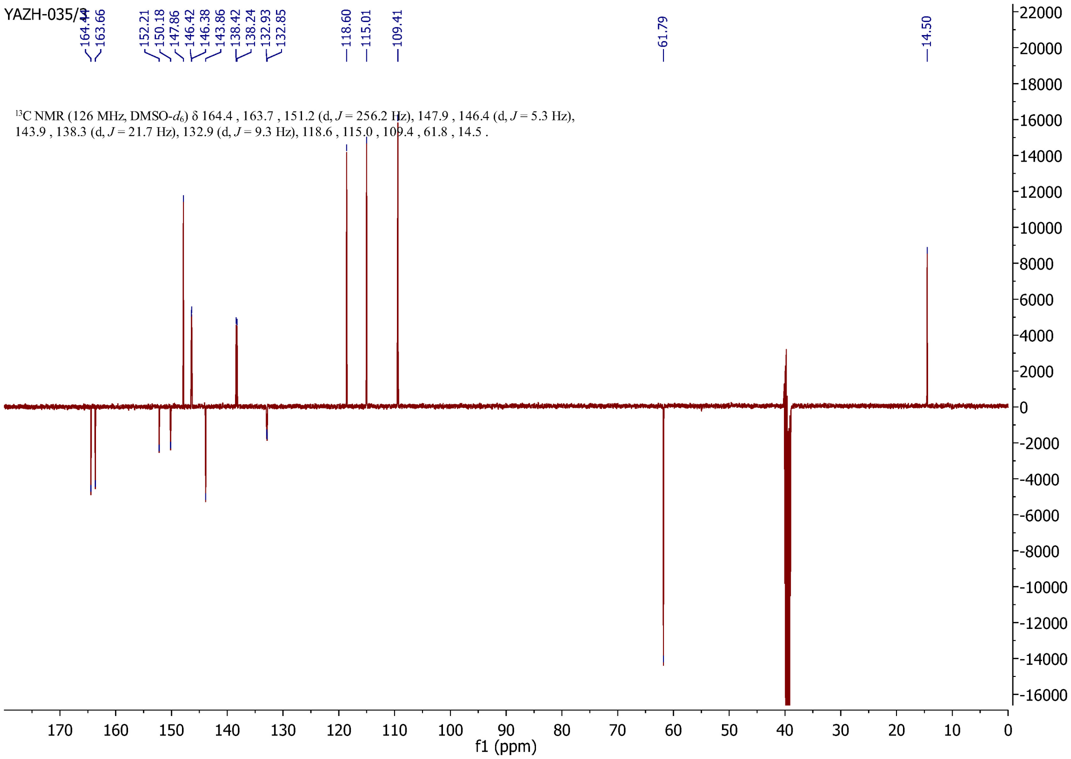

Figure S65 ¹³C NMR spectrum of compound NPD-1192

Acquired by	: Admin
Date Acquired	: 10/15/2015 9:25:18 PM
Sample Name	: YAZH-036
Sample ID	:
Tray#	: 1
Vial#	: 34
Injection Volume	: 1
Data File	: C:\LabSolutions\Data\2015\2015-wk42\YAZH-036.lcd
Background File	: blanco 16102016.lcd
Method File	: Method SCAN ACID standard.lcm
Report Format	: DefaultLCMS.lcr
Tuning File	: C:\LabSolutions\Tuning File\Tuning-ESI-pos-neg01072015.lct
Processed by	: Admin
Modified Date	: 10/19/2015 9:03:28 AM

PDA Graph

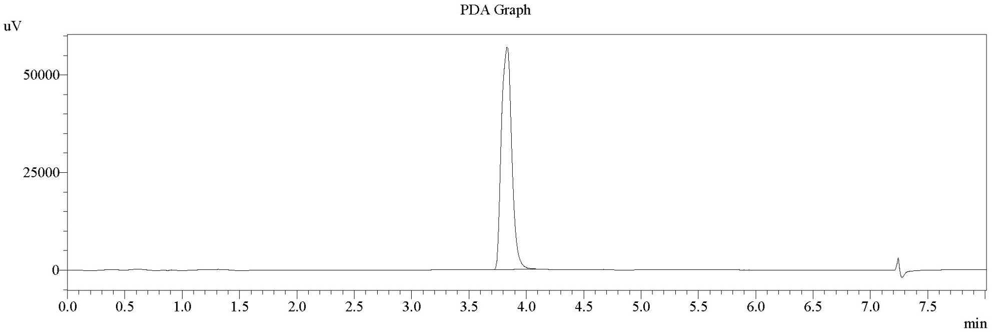

PDA Ch1 254nm 4nm

Peak#	Name	Ret. Time	Area	Area %
1		3.828	358888	100.000

MS Spectrum Graph

#:1 Ret.Time:Averaged 3.890-3.910(Scan#:390-392
Mass Peaks:6 Base Peak:307.05(1032243) Polarity:Pos Segment1 - Event1

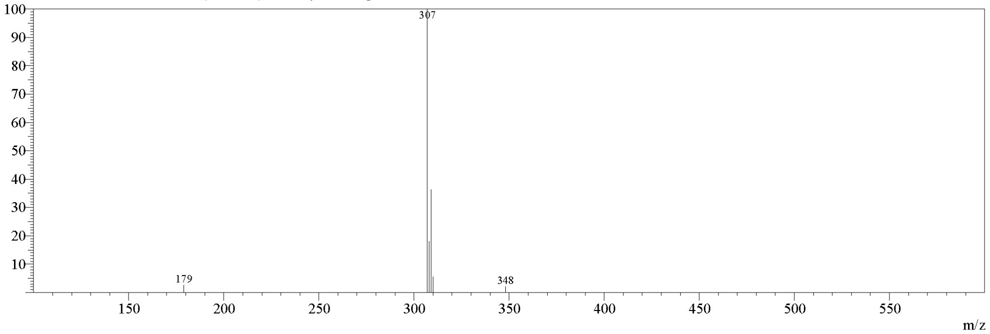

MS Spectrum Table

#:1 Ret.Time:
BG Mode:Calc 3.750<->4.170(376<->418
Mass Peaks:6 Base Peak:307.05(1032243) Polarity:Pos Segment1 - Event

#	m/z	Abs.Inten.	Rel.Inten.	Charge	Polarity	Monoisotopic
1	179.00	27516	2.67			
2	307.05	1032243	100.00			
3	308.05	187211	18.14			
4	309.00	375372	36.36			
5	310.05	56840	5.51			
6	348.10	21337	2.07			

Figure S66 LCMS spectrum of compound NPD-1193

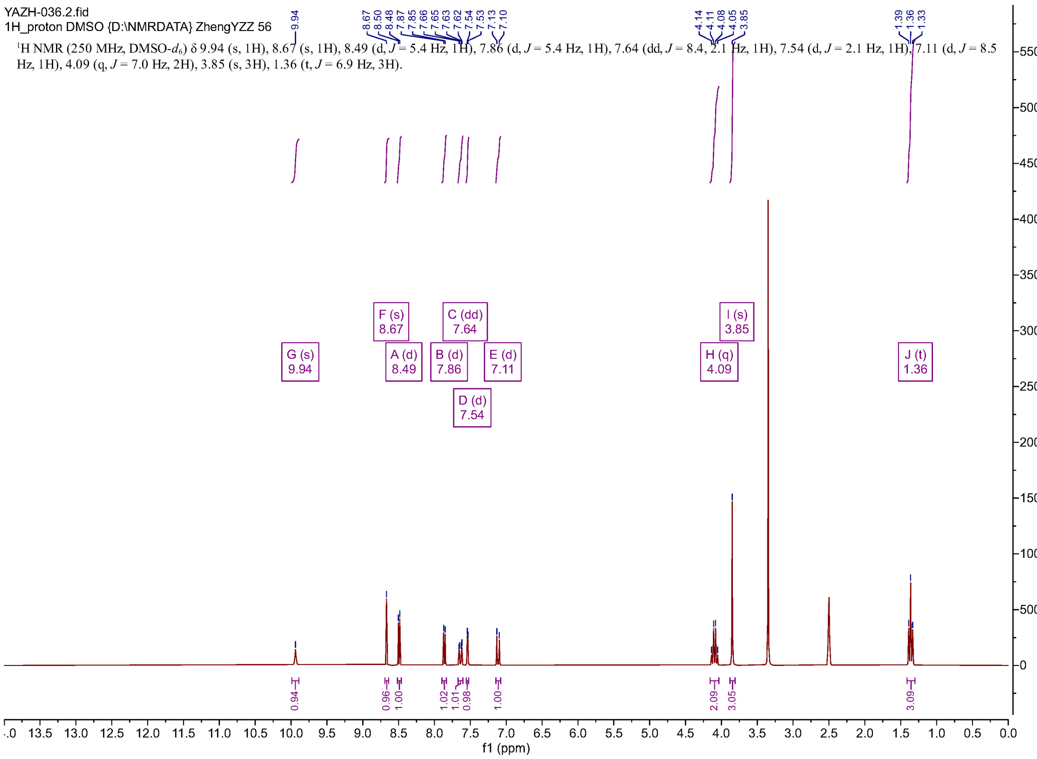

Figure S67 ^{1}H NMR spectrum of compound NPD-1193

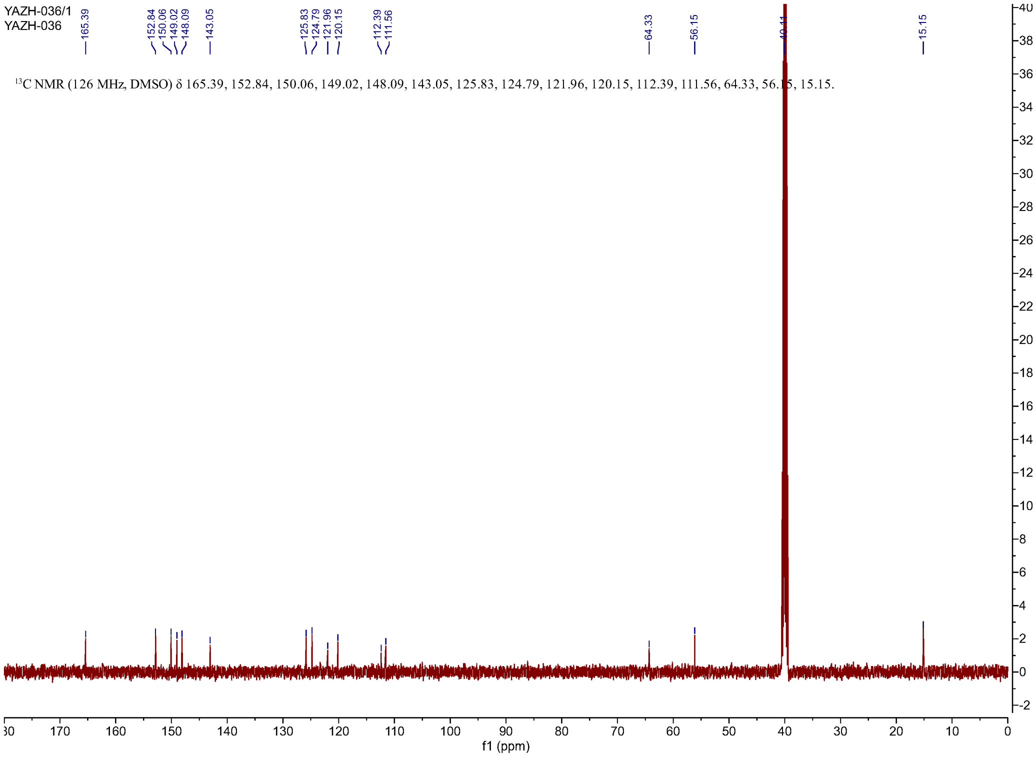

Figure S68 ^{13}C NMR spectrum of compound NPD-1193

Acquired by	: Admin
Date Acquired	: 10/16/2015 12:26:39 AM
Sample Name	: YAZH-057
Sample ID	:
Tray#	: 1
Vial#	: 55
Injection Volume	: 1
Data File	: C:\LabSolutions\Data\2015\2015-wk42\YAZH-057.lcd
Background File	: blanco 16102016.lcd
Method File	: Method SCAN ACID standard.lcm
Report Format	: DefaultLCMS.lcr
Tuning File	: C:\LabSolutions\Tuning File\Tuning-ESI-pos-neg01072015.lct
Processed by	: Admin
Modified Date	: 10/16/2015 11:57:34 AM

PDA Graph

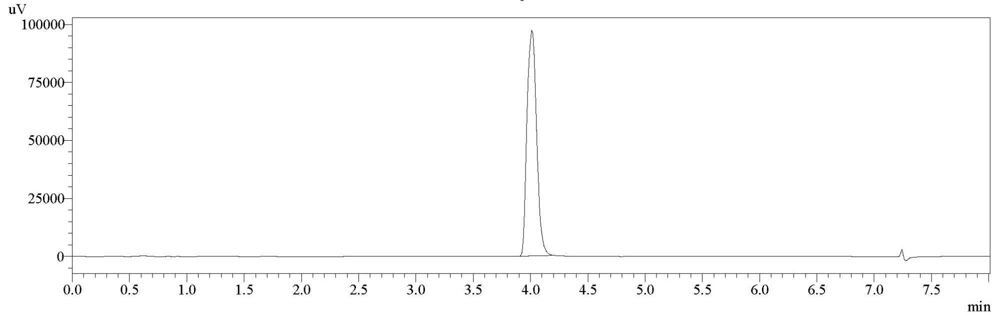

PDA Ch1 254nm 4nm

Peak#	Name	Ret. Time	Area	Area %
1		4.008	570367	100.000

MS Spectrum Graph

#:1 Ret.Time:Averaged 4.080-4.100(Scan#:409-411
Mass Peaks:10 Base Peak:311.95(1102667) Polarity:Pos Segment1 - Event1

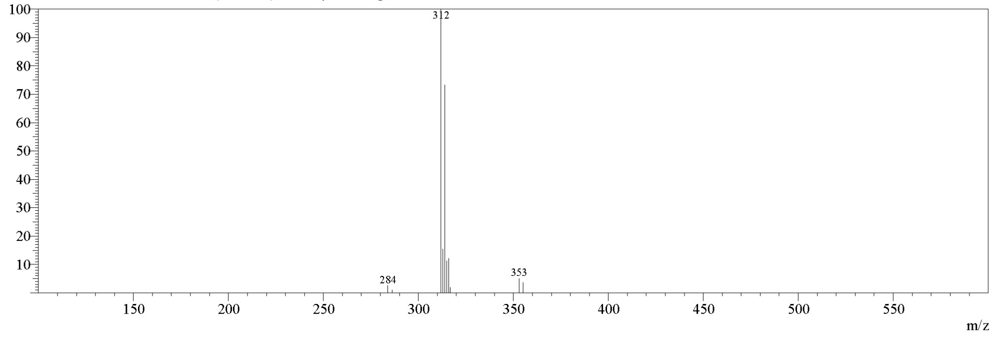

MS Spectrum Table

#:1 Ret.Time:
BG Mode:Calc 3.940<->4.290(395<->430
Mass Peaks:10 Base Peak:311.95(1102667) Polarity:Pos Segment1 - Event

#	m/z	Abs.Inten.	Rel.Inten.	Charge	Polarity	Monoisotopic
1	284.00	27661	2.51			
2	286.10	11507	1.04			
3	311.95	1102667	100.00			
4	313.00	169450	15.37			
5	314.00	808420	73.31			

#	m/z	Abs.Inten.	Rel.Inten.	Charge	Polarity	Monoisotopic
6	315.00	124002	11.25			
7	316.00	133888	12.14			
8	316.90	20365	1.85			
9	353.00	55551	5.04			
10	355.05	39760	3.61			

Figure S69 LCMS spectrum of compound NPD-1194

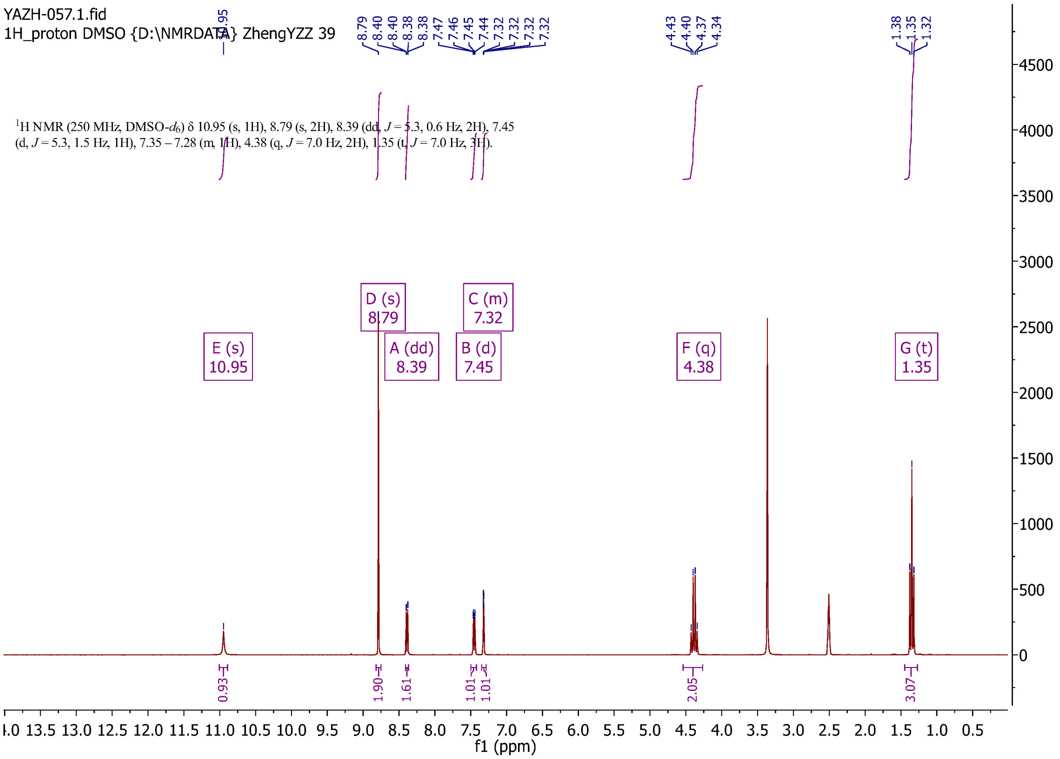

Figure S70 ^{1}H NMR spectrum of compound NPD-1194

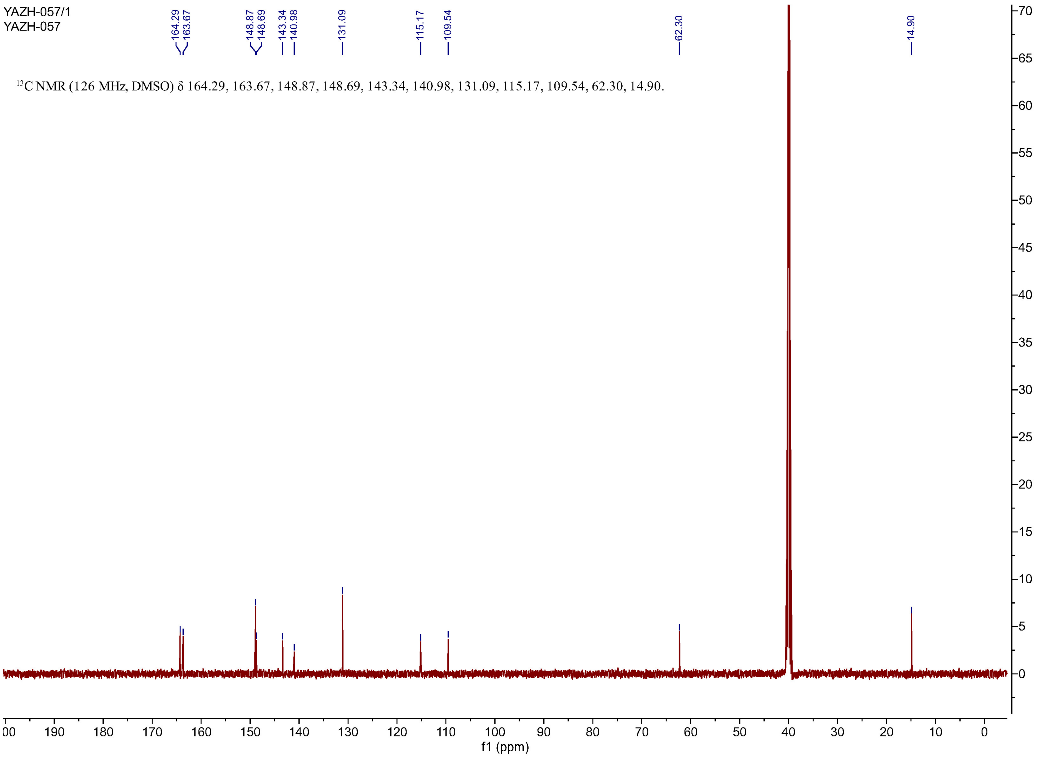

Figure S71 ^{13}C NMR spectrum of compound NPD-1194

Acquired by	: Admin
Date Acquired	: 10/16/2015 1:53:00 AM
Sample Name	: YAZH-067
Sample ID	:
Tray#	: 1
Vial#	: 65
Injection Volume	: 1
Data File	: C:\LabSolutions\Data\2015\2015-wk42\YAZH-067.lcd
Background File	: blanco 16102016.lcd
Method File	: Method SCAN ACID standard.lcm
Report Format	: DefaultLCMS.lcr
Tuning File	: C:\LabSolutions\Tuning File\Tuning-ESI-pos-neg01072015.lct
Processed by	: Admin
Modified Date	: 10/16/2015 12:10:12 PM

PDA Graph

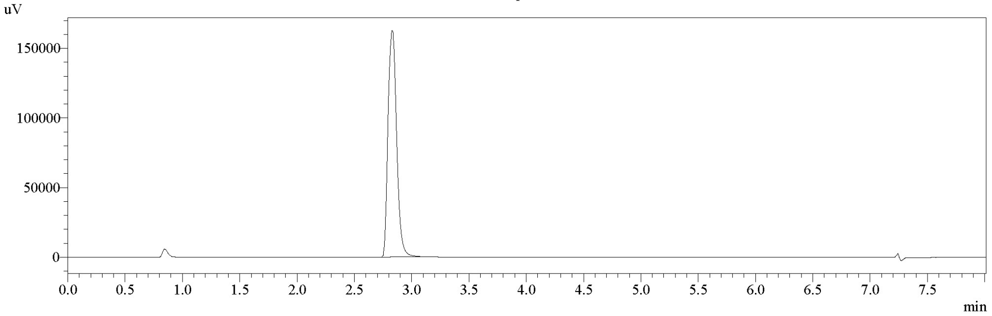

PDA Ch1 254nm 4nm

Peak#	Name	Ret. Time	Area	Area %
1		2.826	851963	100.000

MS Spectrum Graph

#:1 Ret.Time:Averaged 2.900-2.920(Scan#:291-293)
Mass Peaks:4 Base Peak:247.95(1162640) Polarity:Pos Segment1 - Event1

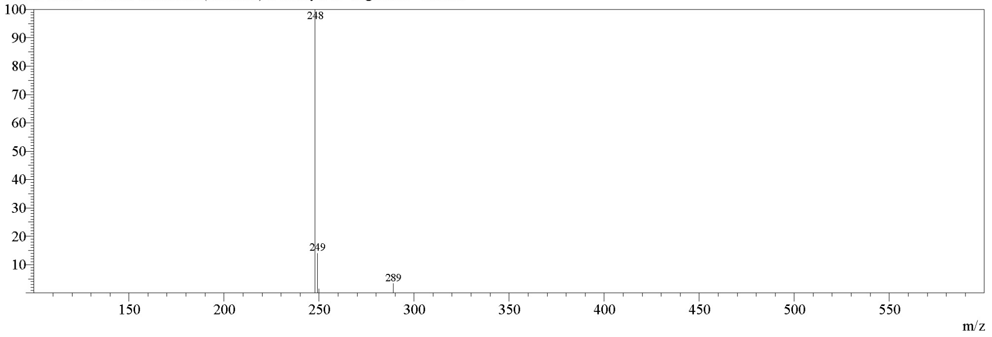

MS Spectrum Table

#:1 Ret.Time:
BG Mode:Calc 2.770<->3.100(278<->311
Mass Peaks:4 Base Peak:247.95(1162640) Polarity:Pos Segment1 - Event

#	m/z	Abs.Inten.	Rel.Inten.	Charge	Polarity	Monoisotopic	#	m/z	Abs.Inten.	Rel.Inten.	Charge	Polarity	Monoisotopic
1	247.95	1162640	100.00				3	249.95	17045	1.47			
2	249.05	162346	13.96				4	288.95	38528	3.31			

Figure S72 LCMS spectrum of compound NPD-1195

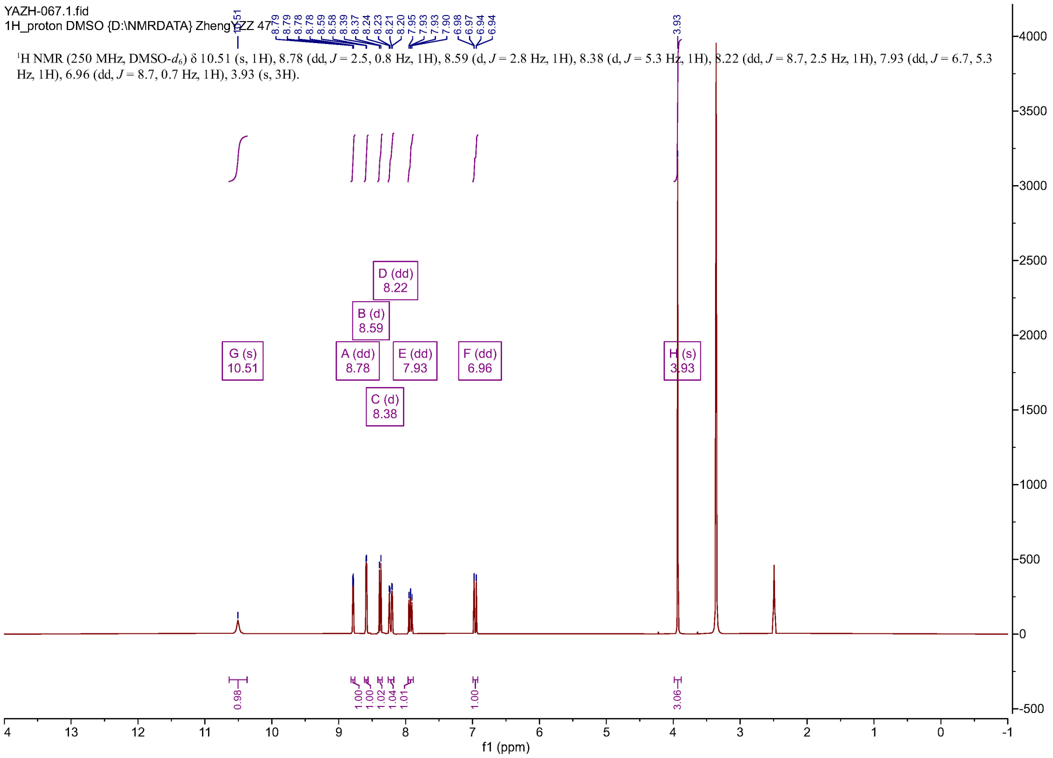

Figure S73 ^{1}H NMR spectrum of compound NPD-1195

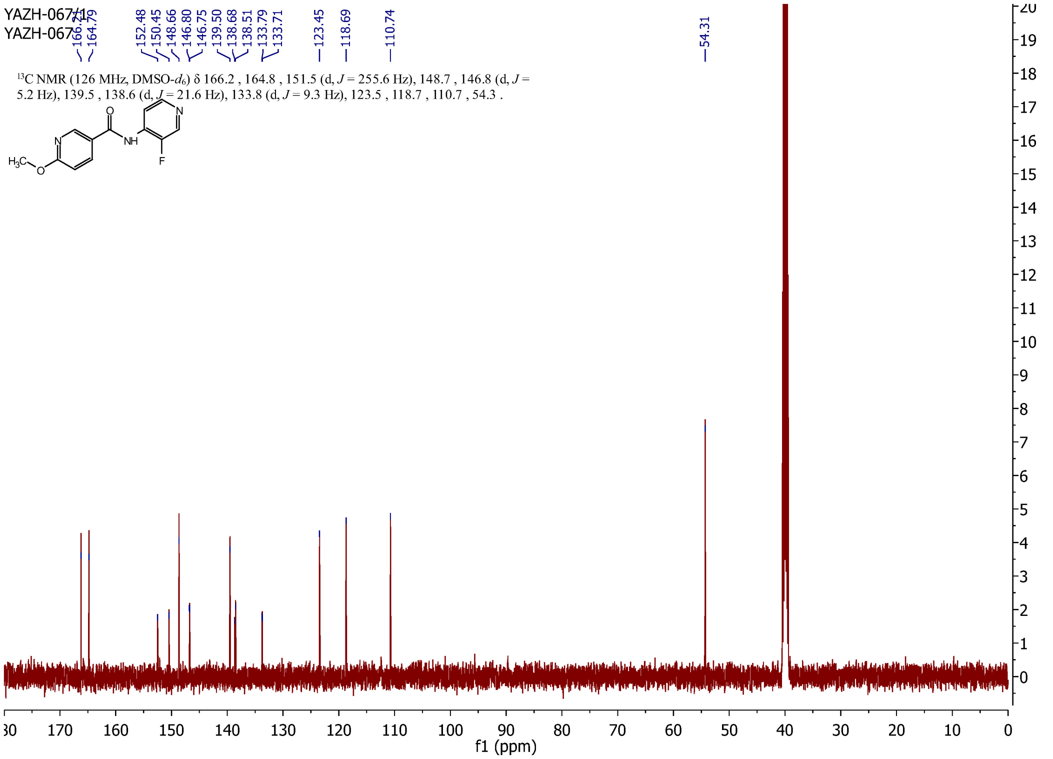

Figure S74 ^{13}C NMR spectrum of compound NPD-1195

Acquired by : Admin
Date Acquired : 1/26/2016 6:32:01 PM
Sample Name : Arien compound 68
Sample ID :
Tray# : 1
Vial# : 15
Injection Volume : 1
Data File : C:\LabSolutions\Methods\FAR Pract Arien compound 68.lcd
Background File : Blanco-2 26012016.lcd
Method File : Method SCAN ACID standard.lcm
Report Format : DefaultLCMS.lcr
Tuning File : C:\LabSolutions\Tuning File\Tuning-ESI-pos-neg01072015.lct
Processed by : Admin
Modified Date : 1/27/2016 9:25:55 AM

PDA Graph

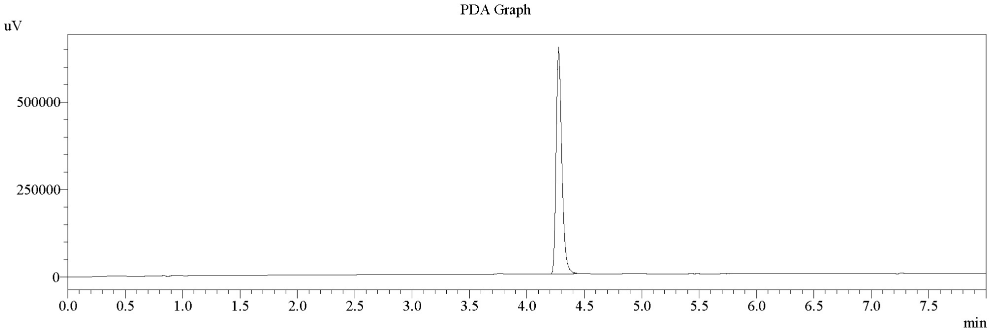

PDA Ch1 254nm 4nm

Peak#	Name	Ret. Time	Area	Area %
1		4.271	2209356	100.000

MS Spectrum Graph

#:1 Ret.Time:Averaged 4.360-4.420(Scan#:436-442)
Mass Peaks:6 Base Peak:357.02(2049230) Polarity:Pos Segment1 - Event1

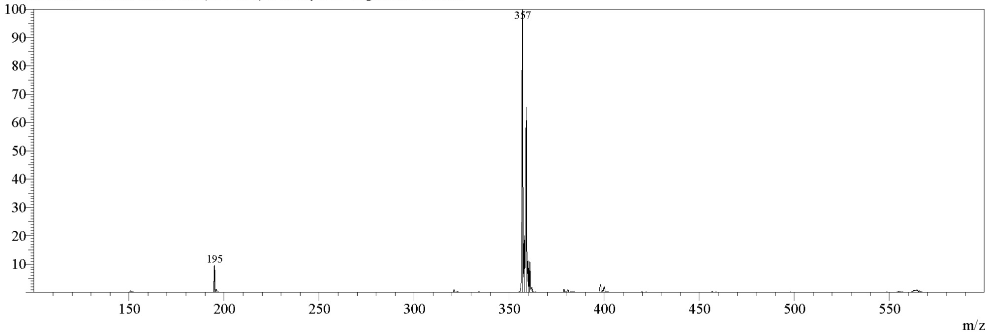

MS Spectrum Table

#:1 Ret.Time:
BG Mode:None
Mass Peaks:6 Base Peak:357.02(2015727) Polarity:Pos Segment1 - Event

#	m/z	Abs.Inten.	Rel.Inten.	Charge	Polarity	Monoisotopic	#	m/z	Abs.Inten.	Rel.Inten.	Charge	Polarity	Monoisotopic
1	194.94	182373	9.05				4	359.01	1331645	66.06			
2	357.02	2015727	100.00				5	359.94	226471	11.24			
3	358.01	398416	19.77				6	360.94	214688	10.65			

Figure S75 LCMS spectrum of compound NPD-1196

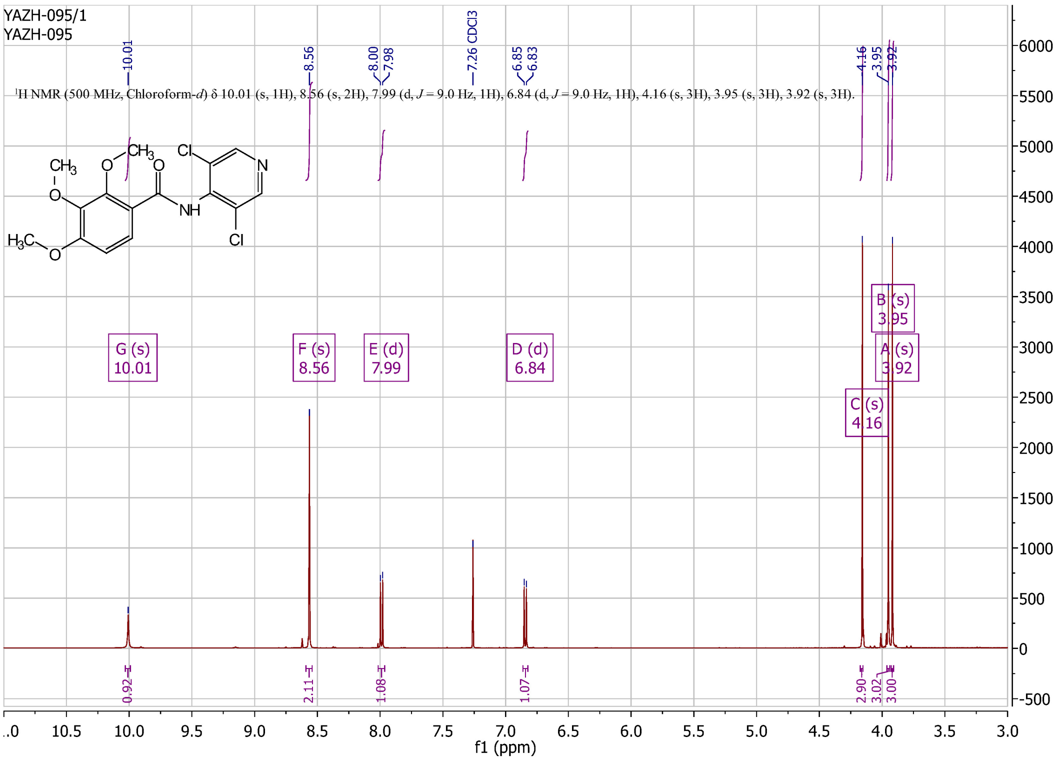

Figure S76 ^{1}H NMR spectrum of compound NPD-1196

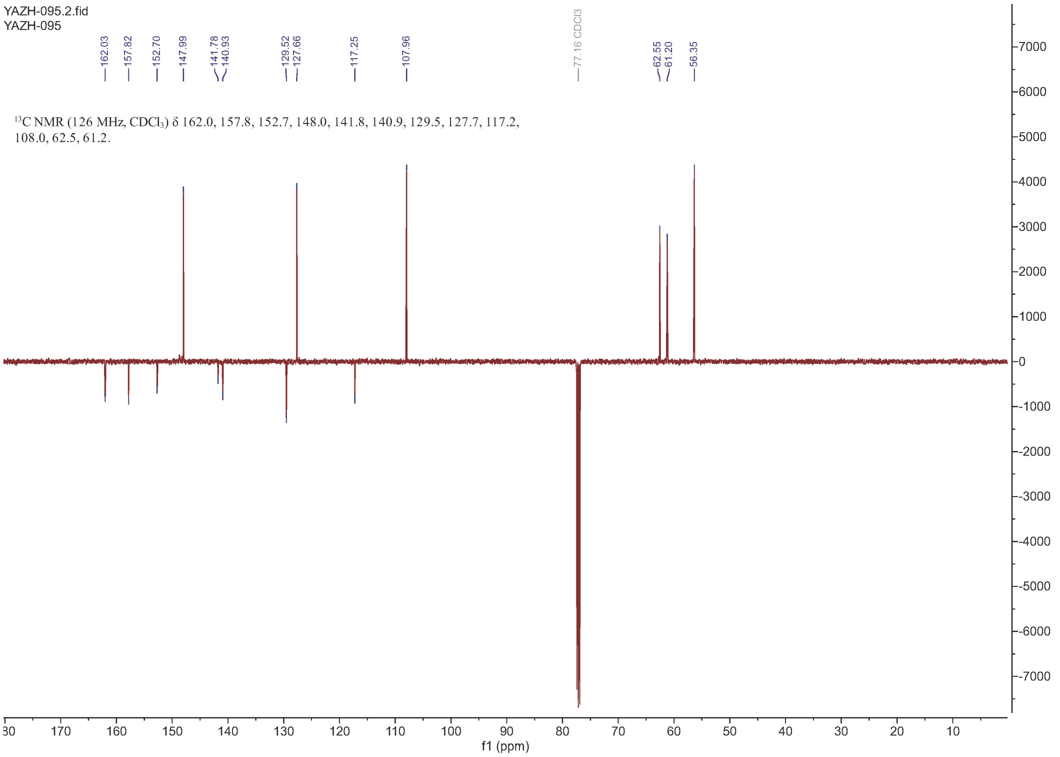

Figure S77 ^{13}C NMR spectrum of compound NPD-1196

Medicinal Chemistry
Vrije Universiteit Amsterdam

Acquired by	: Admin
Date Acquired	: 13/5/2019 9:34:56 AM
Sample Name	: VUF15577
Sample ID	:
Tray#	: 1
Vial#	: 3
Injection Volume	: 3
Data File	: C:\LabSolutions\Data\2019\2019-wk20\VUF15577.lcd
Background File	: blanco 13052019.lcd
Method File	: Method SCAN ACID standard.lcm
Report Format	: DefaultLCMS.lcr
Tuning File	: C:\LabSolutions\Tuning\Tuning-ESI-pos-neg01072015a.lct
Processed by	: Admin
Modified Date	: 13/5/2019 10:57:10 AM

PDA Graph

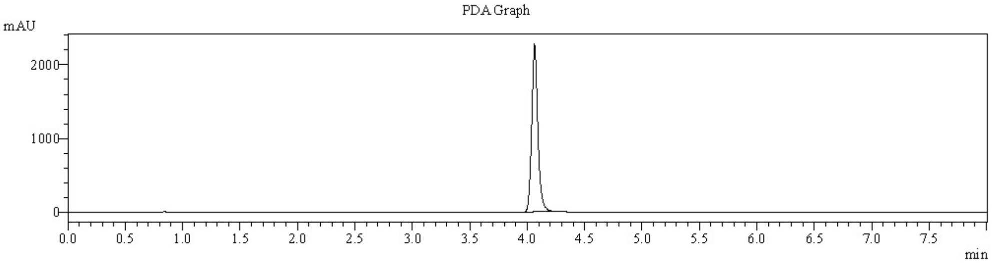

PeakTable

PDA Ch1 254nm 4nm

Peak#	Name	Ret. Time	Area	Area %
1		4.059	8301793	100.000

MS Chromatogram
VUF15577 C:\LabSolutions\Data\2019\2019-wk20\VUF15577.lcd

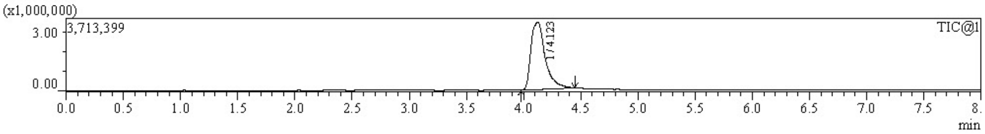

MS Spectrum Graph

#:1 Ret.Time:Averaged 4.110-4.130(Scan#:412-414)
BG Mode:Calc 3.980<->4.450(399<->446)
Mass Peaks:6 Base Peak:323.05(2105968) Polarity:Pos Segment1 - Event1

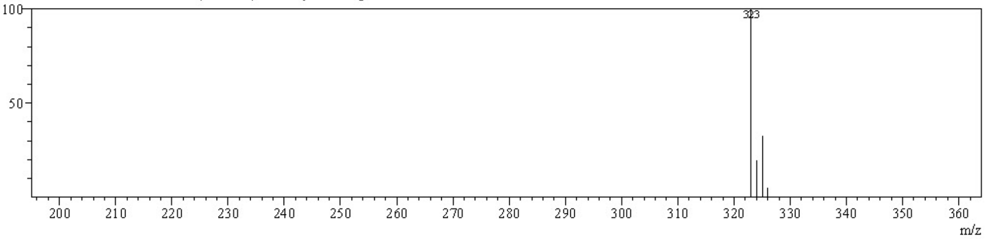

MS Spectrum Table

#:1 Ret.Time:
BG Mode:Calc 3.980<->4.450(399<->446)
Mass Peaks:6 Base Peak:323.05(2105968) Polarity:Pos Segment1 - Event1

#	m/z	Abs.Inten.	Rel.Inten.	Charge	Polarity	Monoisotopic
1	195.00	68515	3.25			
2	323.05	2105968	100.00			
3	324.05	412686	19.60			
4	325.05	682617	32.41			
5	326.05	104359	4.96			
6	364.05	58990	2.80			

Figure S78 LCMS spectrum of compound NPD-1197

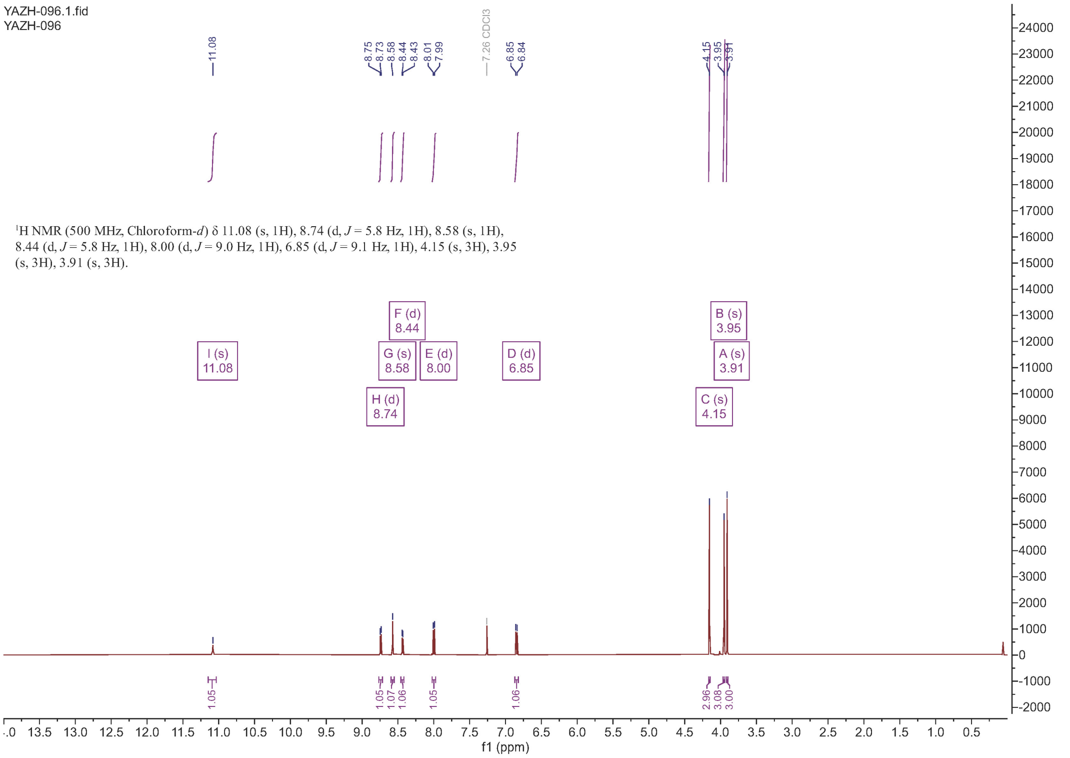

Figure S79 ^{1}H NMR spectrum of compound NPD-1197

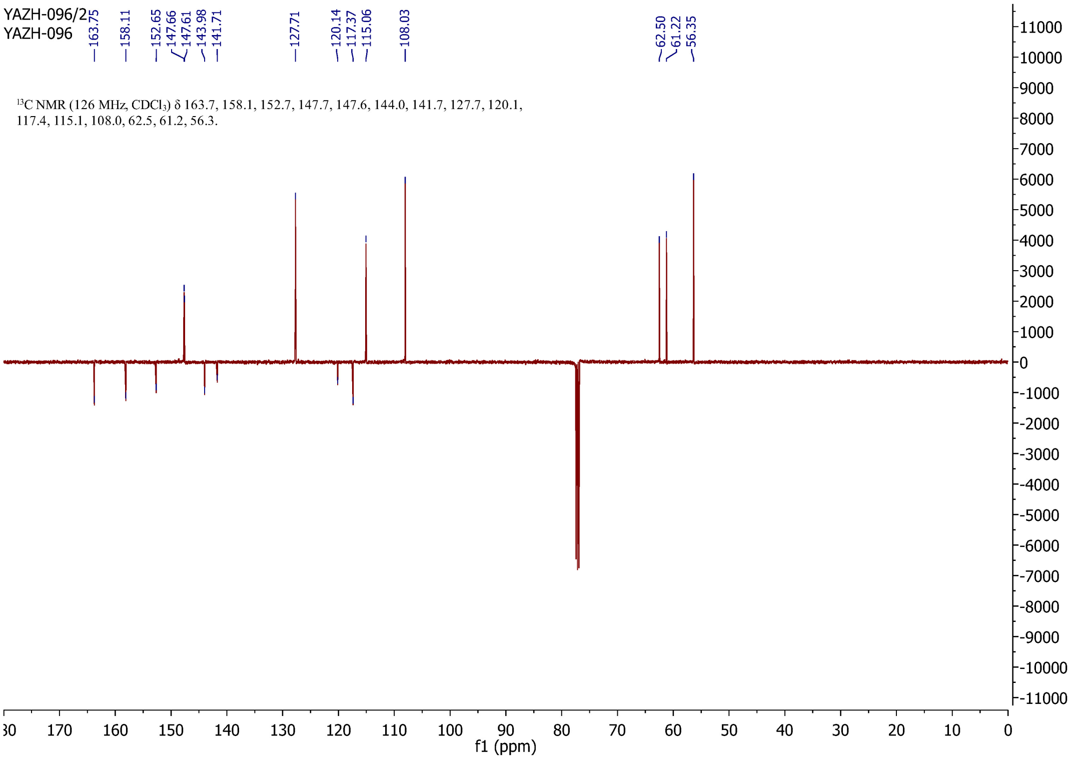

Figure S80 ^{13}C NMR spectrum of compound NPD-1197

Acquired by	: Admin
Date Acquired	: 1/26/2016 10:42:51 PM
Sample Name	: Floor compound 71
Sample ID	:
Tray#	: 1
Vial#	: 44
Injection Volume	: 1
Data File	: C:\LabSolutions\Methods\FAR Pract Floor compound 71.lcd
Background File	: Blanco-2 26012016.lcd
Method File	: Method SCAN ACID standard.lcm
Report Format	: DefaultLCMS.lcr
Tuning File	: C:\LabSolutions\Tuning File\Tuning-ESI-pos-neg01072015.lct
Processed by	: Admin
Modified Date	: 1/27/2016 11:34:03 AM

PDA Graph

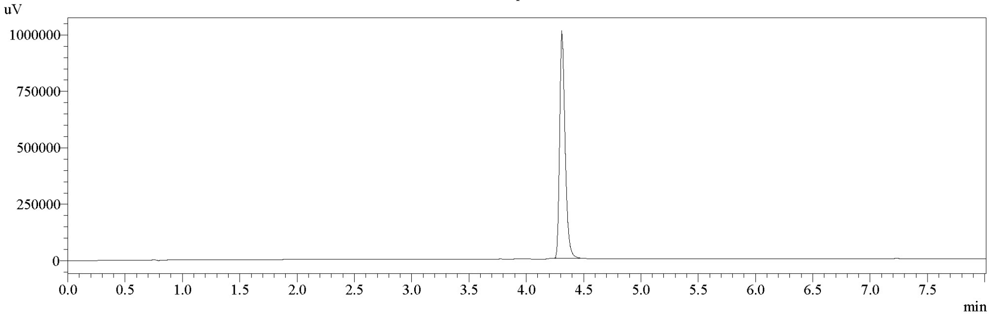

PDA Ch1 254nm 4nm

Peak#	Name	Ret. Time	Area	Area %
1		4.306	3356031	100.000

MS Spectrum Graph

#:1 Ret.Time:Averaged 4.410-4.450(Scan#:441-445)
Mass Peaks:7 Base Peak:345.83(887348) Polarity:Pos Segment1 - Event1

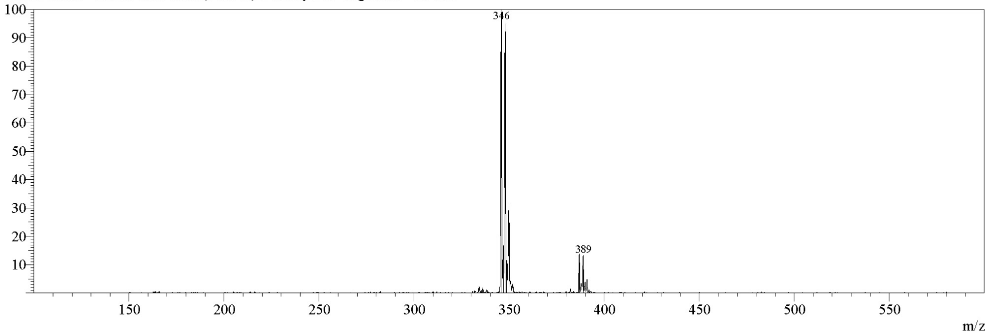

MS Spectrum Table

#:1 Ret.Time:
BG Mode:None
Mass Peaks:7 Base Peak:345.83(804222) Polarity:Pos Segment1 - Event

#	m/z	Abs.Inten.	Rel.Inten.	Charge	Polarity	Monoisotopic
1	345.83	804222	100.00			
2	346.96	125677	15.63			
3	347.89	786867	97.84			
4	348.76	99929	12.43			
5	349.89	231288	28.76			
6	386.91	105571	13.13			
7	388.97	109803	13.65			

Figure S81 LCMS spectrum of compound NPD-1198

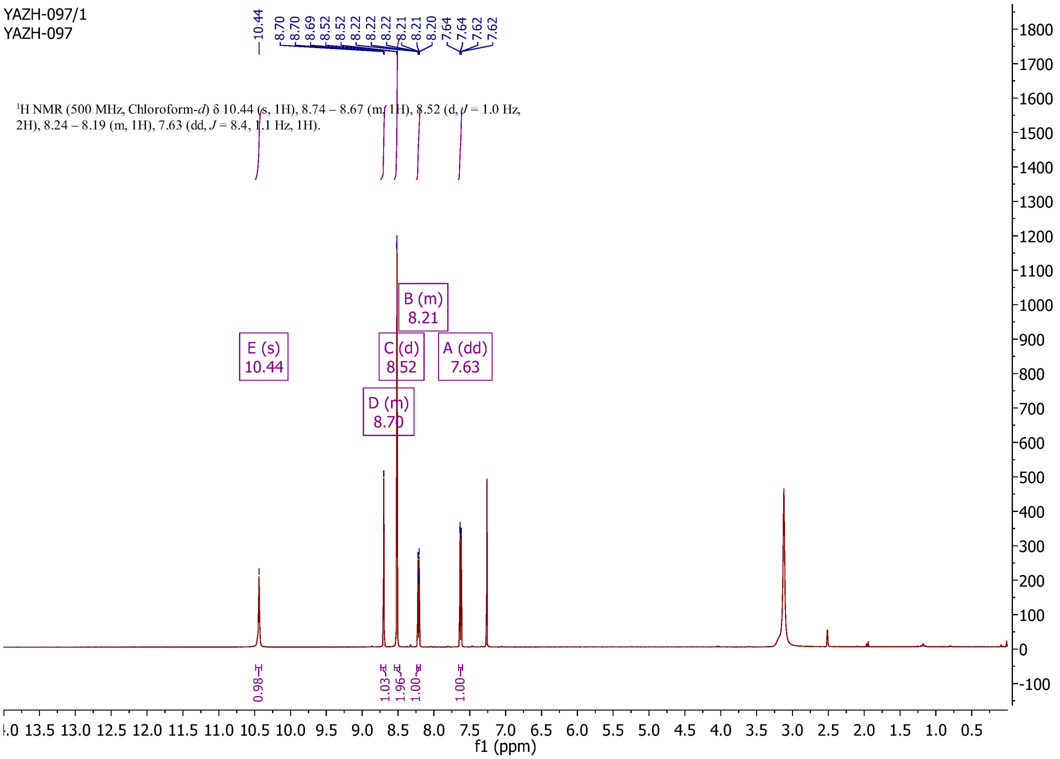

Figure S82 ^{1}H NMR spectrum of compound NPD-1198

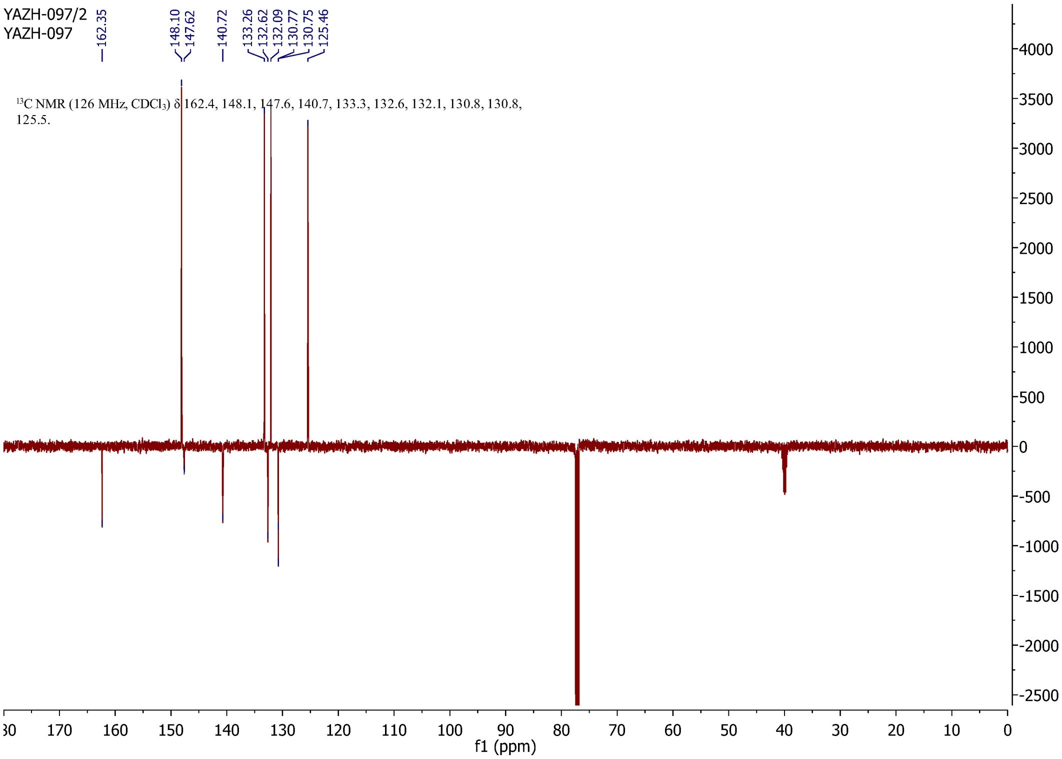

Figure S83 ^{13}C NMR spectrum of compound NPD-1198

Acquired by : Admin
Date Acquired : 1/26/2016 7:23:57 PM
Sample Name : Jay compound 74
Sample ID :
Tray# : 1
Vial# : 21
Injection Volume : 1
Data File : C:\LabSolutions\Methods\FAR Pract Jay compound 74.lcd
Background File : Blanco-2 26012016.lcd
Method File : Method SCAN ACID standard.lcm
Report Format : DefaultLCMS.lcr
Tuning File : C:\LabSolutions\Tuning File\Tuning-ESI-pos-neg01072015.lct
Processed by : Admin
Modified Date : 1/27/2016 9:50:12 AM

PDA Graph

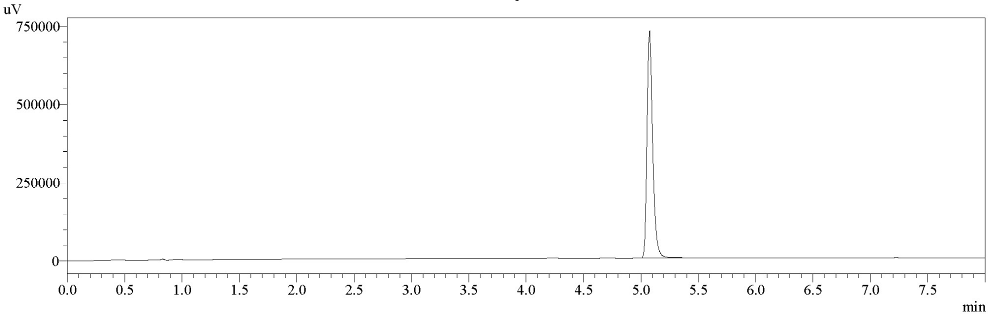

PDA Ch1 254nm 4nm

Peak#	Name	Ret. Time	Area	Area %
1		5.070	2494903	100.000

MS Spectrum Graph

#:1 Ret.Time:Averaged 5.170-5.240(Scan#:517-524)
Mass Peaks:8 Base Peak:452.95(634766) Polarity:Pos Segment1 - Event1

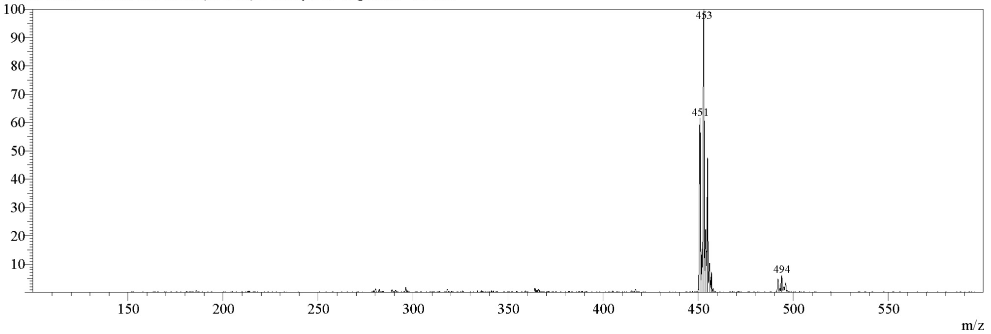

MS Spectrum Table

#:1 Ret.Time:
BG Mode:None
Mass Peaks:8 Base Peak:452.95(593061) Polarity:Pos Segment1 - Event

#	m/z	Abs.Inten.	Rel.Inten.	Charge	Polarity	Monoisotopic
1	450.95	374414	63.13			
2	452.01	85256	14.38			
3	452.95	593061	100.00			
4	453.94	131388	22.15			
5	454.94	261926	44.17			
6	456.01	55285	9.32			
7	457.01	38313	6.46			
8	493.95	37433	6.31			

Figure S84 LCMS spectrum of compound NPD-1199

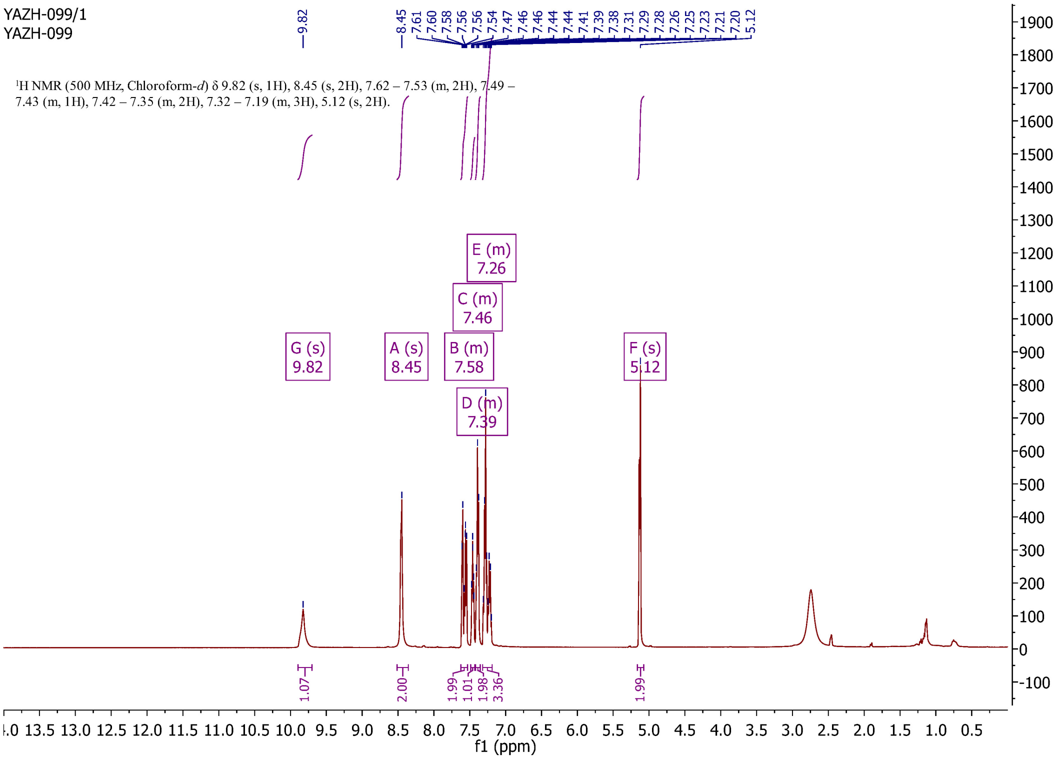

Figure S85 ^{1}H NMR spectrum of compound NPD-1199

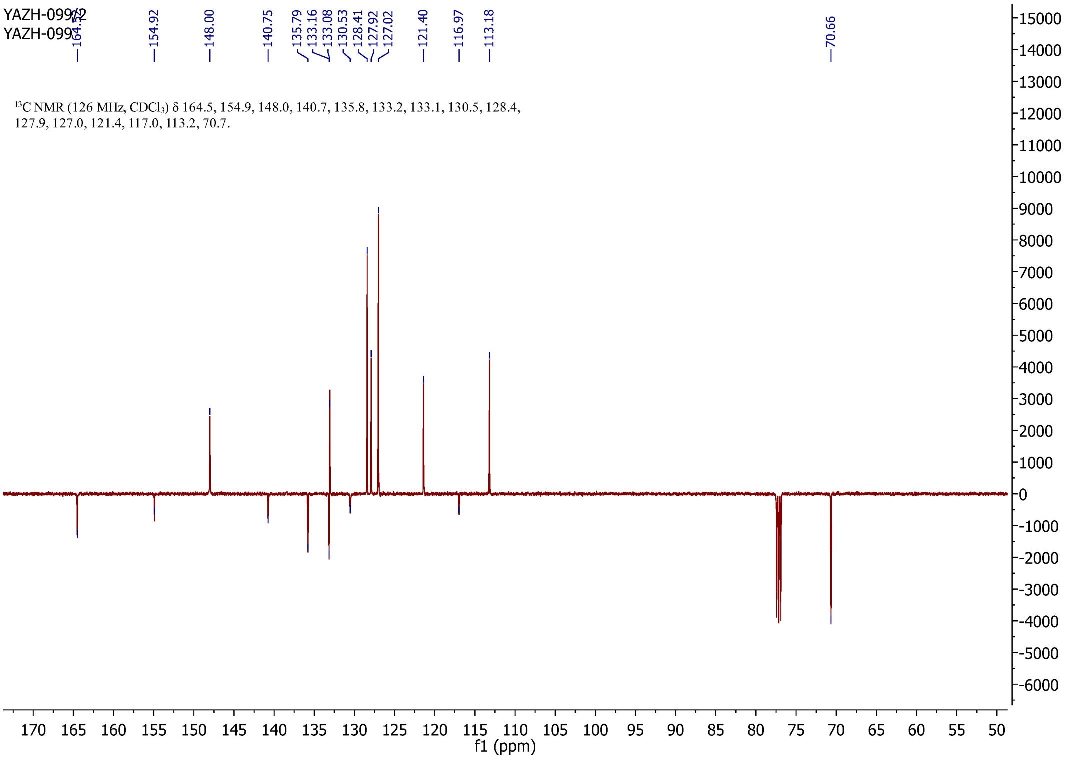

Figure S86 ^{13}C NMR spectrum of compound NPD-1199

Acquired by : Admin
Date Acquired : 1/26/2016 7:32:35 PM
Sample Name : Fatema compound 75A
Sample ID :
Tray# : 1
Vial# : 22
Injection Volume : 1
Data File : C:\LabSolutions\Methods\FAR Pract Fatema compound 75A.lcd
Background File : Blanco-2 26012016.lcd
Method File : Method SCAN ACID standard.lcm
Report Format : DefaultLCMS.lcr
Tuning File : C:\LabSolutions\Tuning File\Tuning-ESI-pos-neg01072015.lct
Processed by : Admin
Modified Date : 1/27/2016 9:55:29 AM

PDA Graph

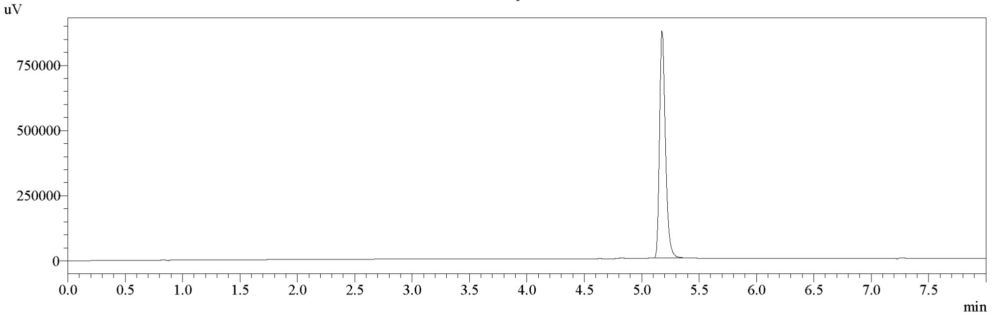

PDA Ch1 254nm 4nm

Peak#	Name	Ret. Time	Area	Area %
1		5.173	3089682	100.000

MS Spectrum Graph

#:1 Ret.Time:Averaged 5.270-5.320(Scan#:527-532)
Mass Peaks:5 Base Peak:418.99(3440008) Polarity:Pos Segment1 - Event1

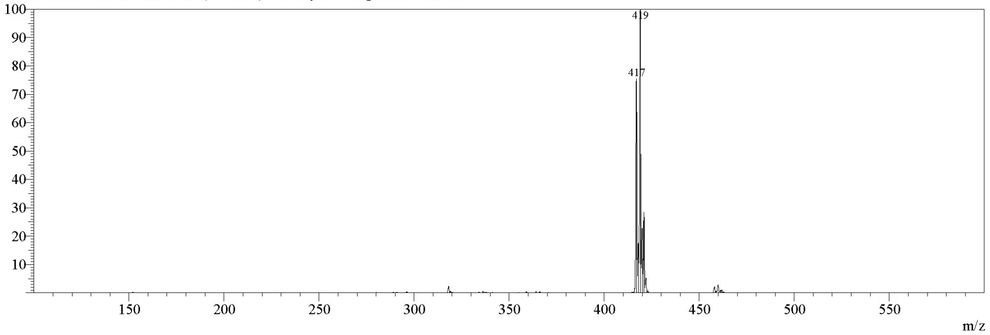

MS Spectrum Table

#:1 Ret.Time:
BG Mode:None
Mass Peaks:5 Base Peak:418.99(3179486) Polarity:Pos Segment1 - Event

#	m/z	Abs.Inten.	Rel.Inten.	Charge	Polarity	Monoisotopic
1	417.00	2499700	78.62			
2	417.93	561811	17.67			
3	418.99	3179486	100.00			
4	419.99	745467	23.45			
5	420.92	888480	27.94			

Figure S87 LCMS spectrum of compound NPD-1200

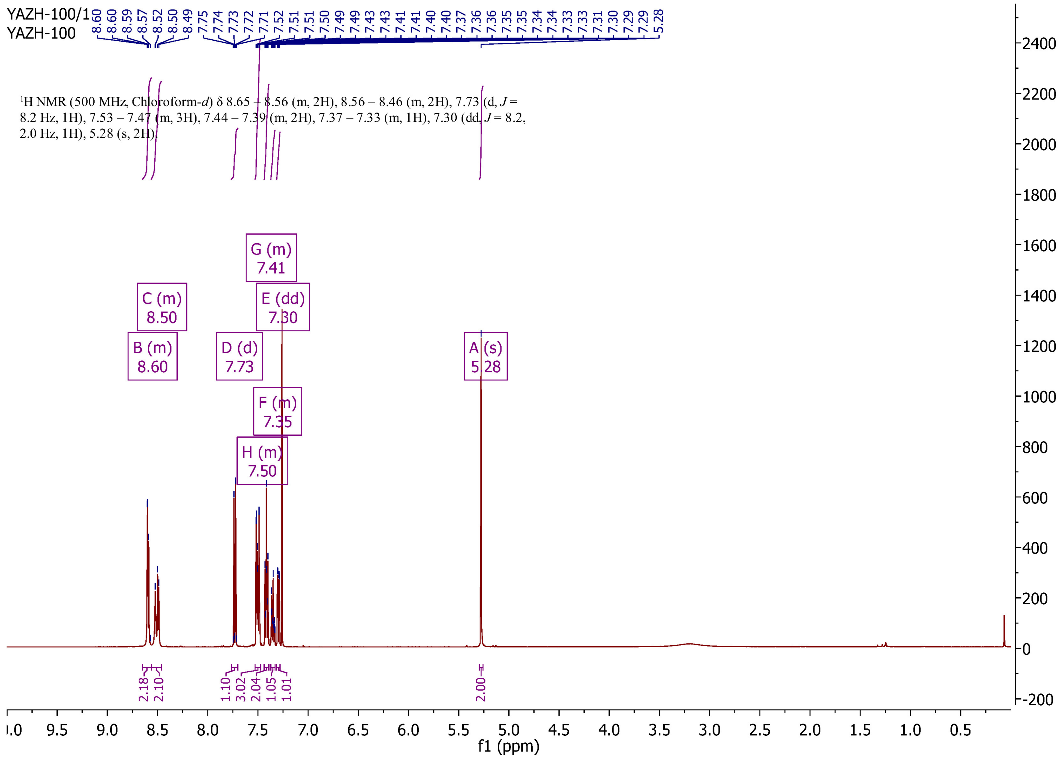

Figure S88 ^{1}H NMR spectrum of compound NPD-1200

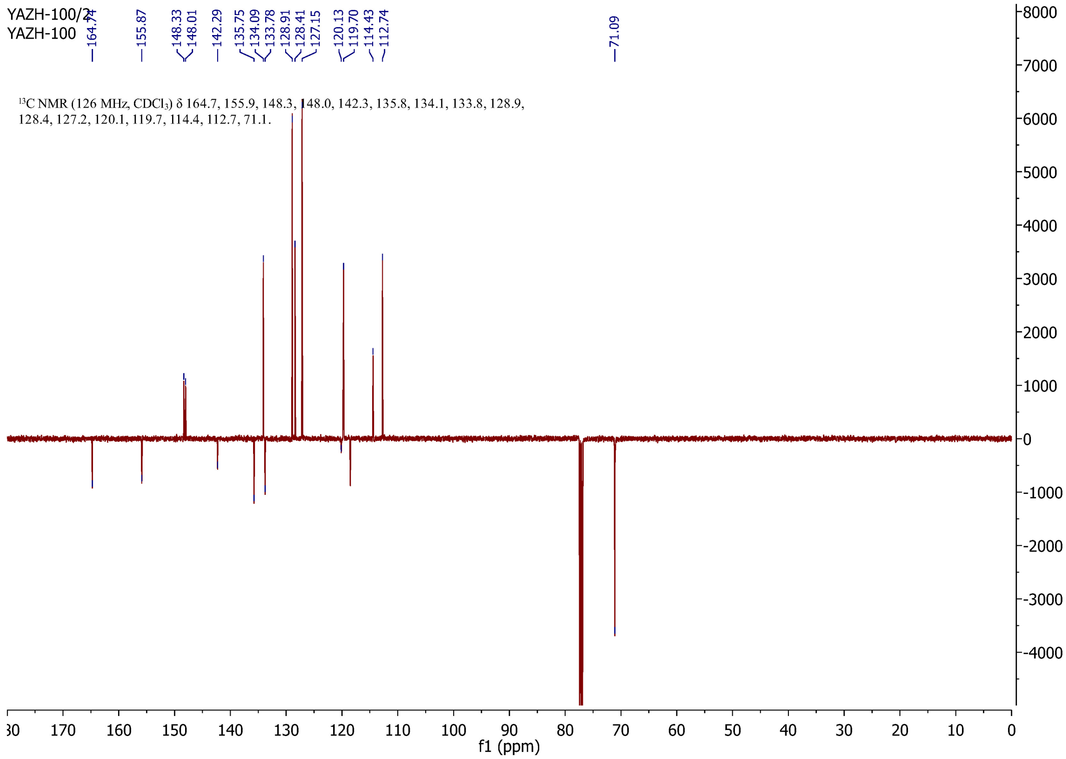

Figure S89 ^{13}C NMR spectrum of compound NPD-1200

Acquired by	: Admin
Date Acquired	: 1/26/2016 8:24:28 PM
Sample Name	: Rosan K compound 78
Sample ID	:
Tray#	: 1
Vial#	: 28
Injection Volume	: 1
Data File	: C:\LabSolutions\Methods\FAR Pract Rosan K compound 78.lcd
Background File	: Blanco-2 26012016.lcd
Method File	: Method SCAN ACID standard.lcm
Report Format	: DefaultLCMS.lcr
Tuning File	: C:\LabSolutions\Tuning File\Tuning-ESI-pos-neg01072015.lct
Processed by	: Admin
Modified Date	: 1/27/2016 10:08:26 AM

PDA Graph

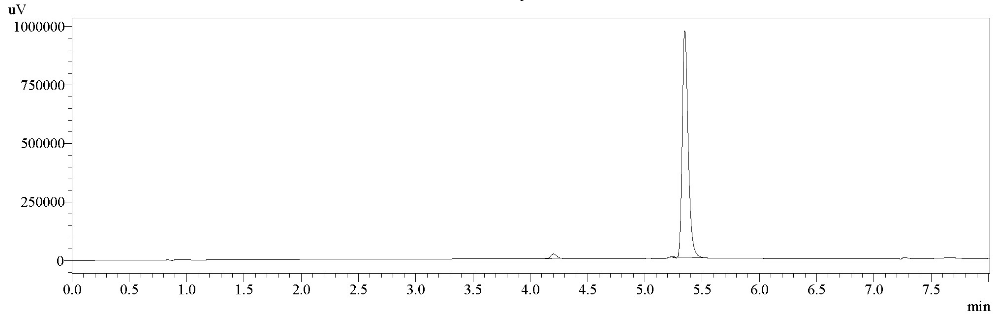

PDA Ch1 254nm 4nm

Peak#	Name	Ret. Time	Area	Area %
1		4.199	65625	1.850
2		5.344	3481795	98.150

MS Spectrum Graph

#:1 Ret.Time:Averaged 5.450-5.480(Scan#:545-548)
Mass Peaks:5 Base Peak:338.98(5223922) Polarity:Pos Segment1 - Event1

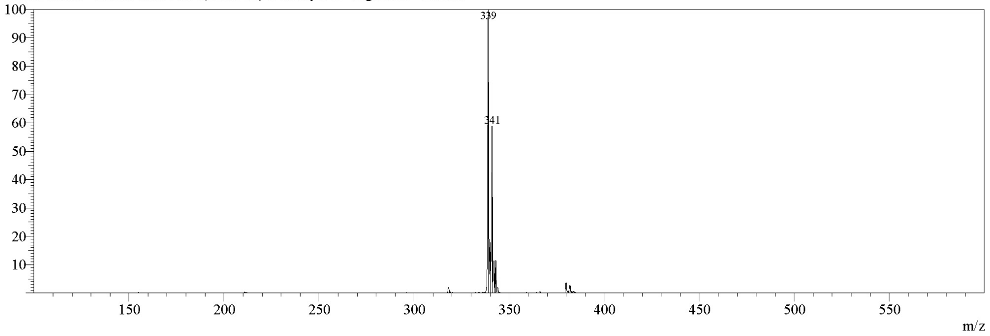

MS Spectrum Table

#:1 Ret.Time:
BG Mode:None
Mass Peaks:5 Base Peak:338.98(4826833) Polarity:Pos Segment1 - Event

#	m/z	Abs.Inten.	Rel.Inten.	Charge	Polarity	Monoisotopic
1	338.98	4826833	100.00			
2	340.04	867402	17.97			
3	341.04	2801323	58.04			
4	342.04	543457	11.26			
5	343.04	515410	10.68			

Figure S90 LCMS spectrum of compound NPD-1201

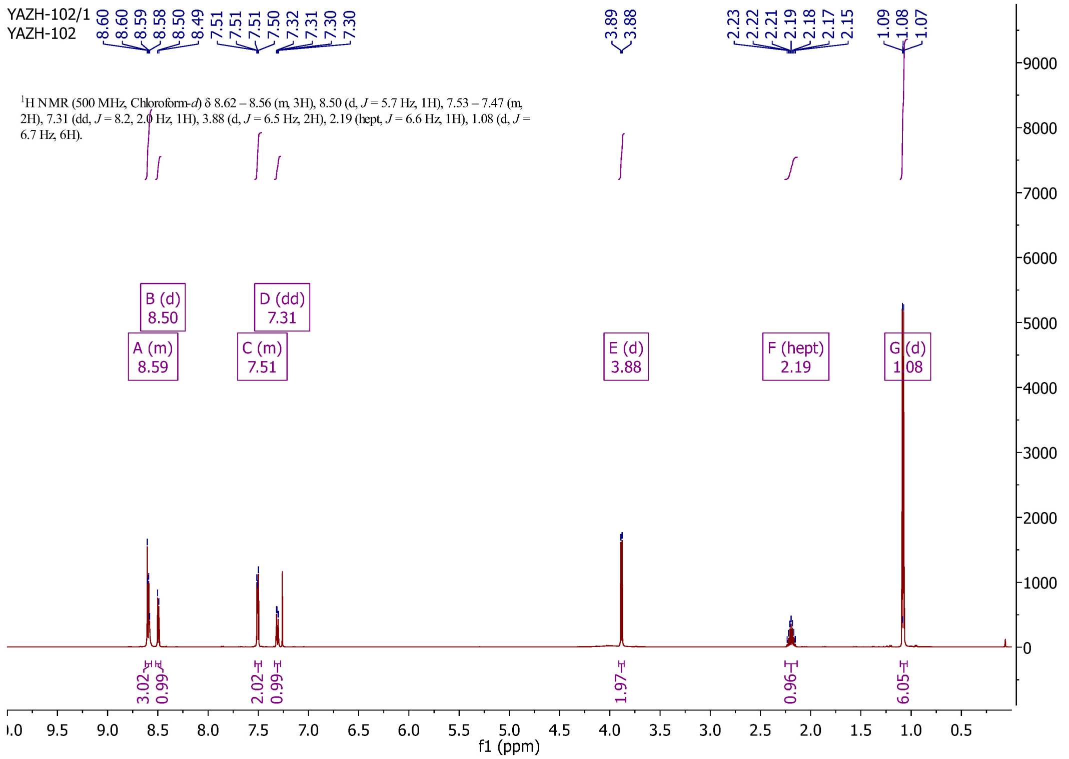

Figure S91 ^{1}H NMR spectrum of compound NPD-1201

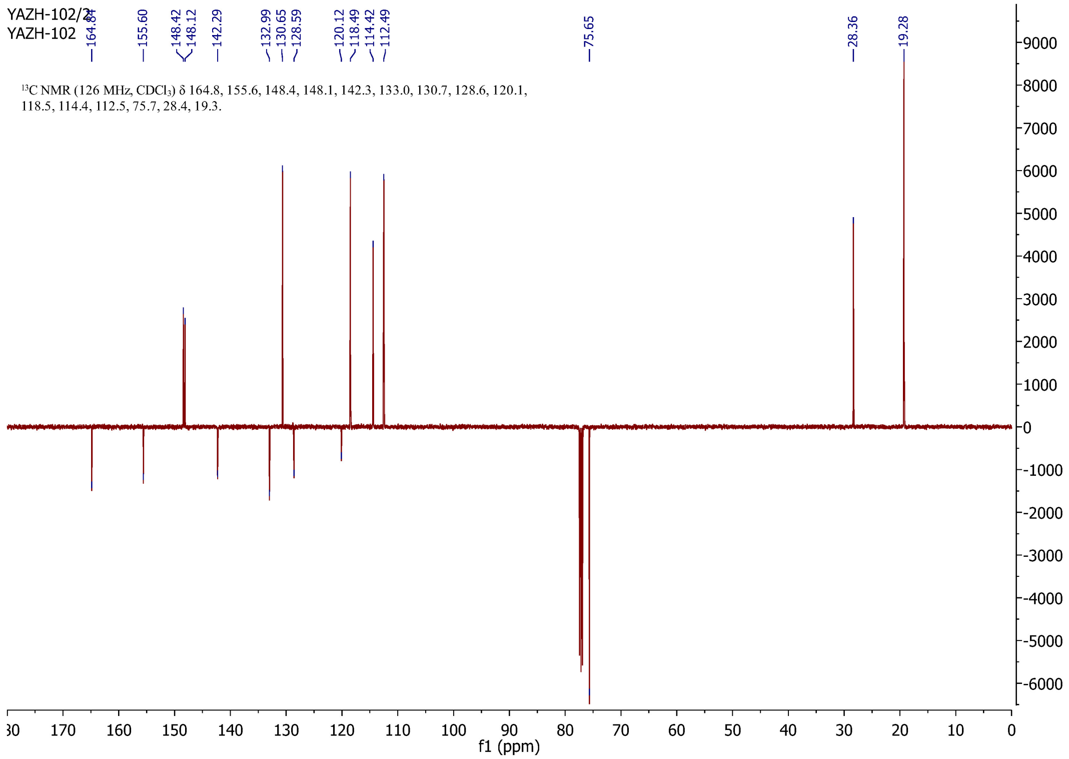

Figure S92 ^{13}C NMR spectrum of compound NPD-1201

Acquired by	: Admin
Date Acquired	: 3/5/2019 3:12:41 PM
Sample Name	: YAZH-103
Sample ID	:
Tray#	: 1
Vial#	: 8
Injection Volume	: 3
Data File	: C:\LabSolutions\Data\2019\2019-wk18\YAZH-103.lcd
Background File	: blanco 03052019.lcd
Method File	: Method SCAN ACID standard.lcm
Report Format	: DefaultLCMS.lcr
Tuning File	: C:\LabSolutions\Tuning\Tuning-ESI-pos-neg01072015a.lct
Processed by	: Admin
Modified Date	: 3/5/2019 3:47:13 PM

Medicinal Chemistry
Vrije Universiteit Amsterdam

Chromatogram
YAZH-103 C:\LabSolutions\Data\2019\2019-wk18\YAZH-103.lcd

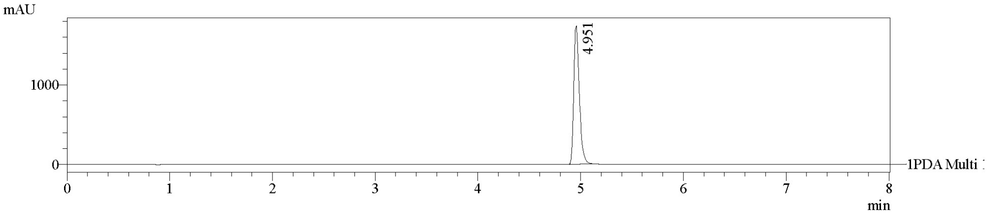

1 PDA Multi 1 / 254nm 4nm
PDA Ch1 254nm 4nm

Peak#	Name	Ret. Time	Area	Area %
1		4.951	6511021	100.000

MS Chromatogram
YAZH-103 C:\LabSolutions\Data\2019\2019-wk18\YAZH-103.lcd

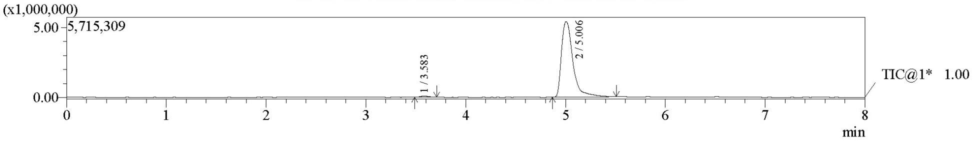

MS Spectrum Graph
#:1 Ret.Time:Averaged 5.000-5.020(Scan#:501-503)
Mass Peaks:13 Base Peak:339.05(2246230) Polarity:Pos Segment1 - Event1

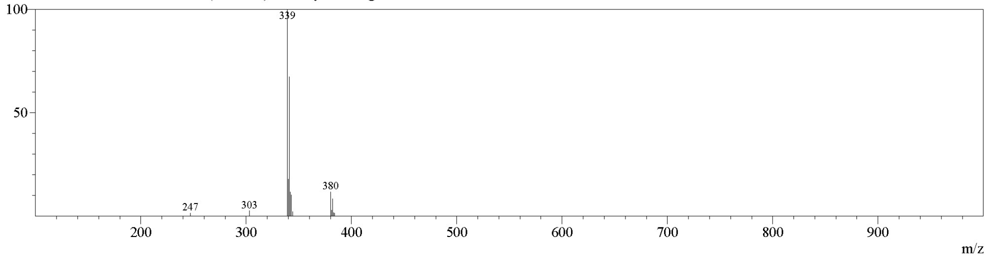

MS Spectrum Table
#:1 Ret.Time:
BG Mode:Calc 4.870<->5.510(488<->552)
Mass Peaks:13 Base Peak:339.05(2246230) Polarity:Pos Segment1 - Event1

#	m/z	Abs.Inten.	Rel.Inten.	Charge	Polarity	Monoisotopic
1	246.95	28892	1.29			
2	303.05	55544	2.47			
3	339.05	2246230	100.00			
4	340.05	398781	17.75			
5	341.00	1515279	67.46			
6	342.00	262388	11.68			
7	343.00	226478	10.08			
8	344.05	44618	1.99			
9	380.10	262473	11.69			
10	381.10	60800	2.71			
11	382.10	187956	8.37			
12	383.05	33808	1.51			
13	384.05	28437	1.27			

Figure S93 LCMS spectrum of compound NPD-1202

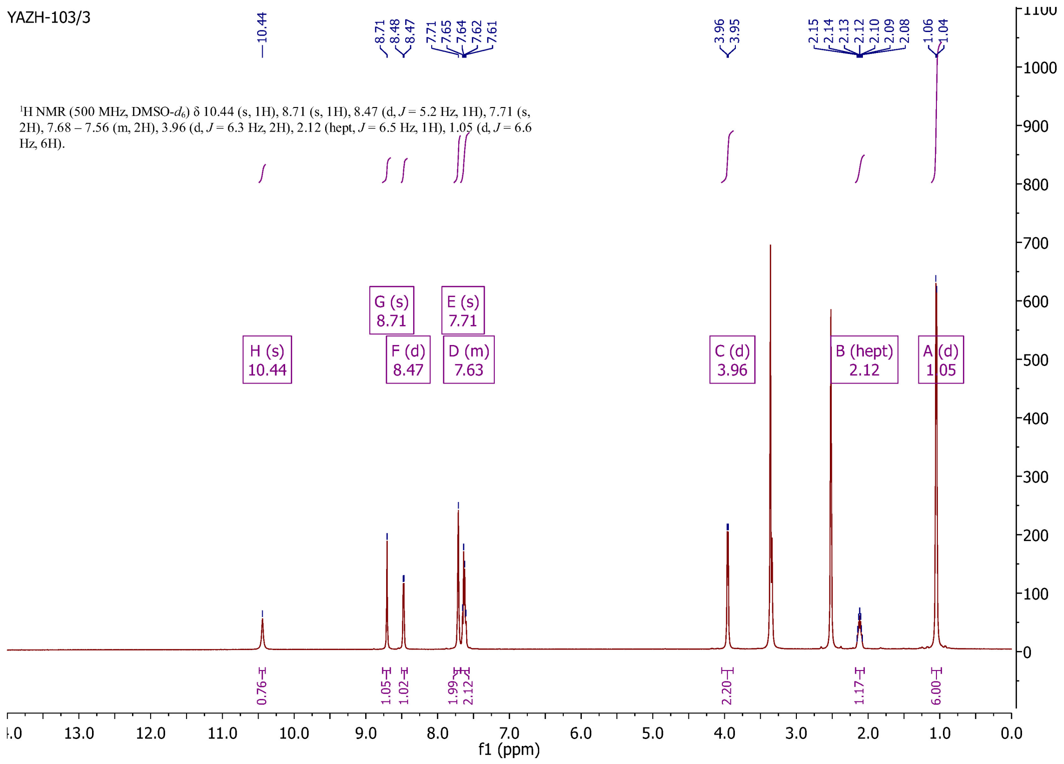

Figure S94 ^{1}H NMR spectrum of compound NPD-1202

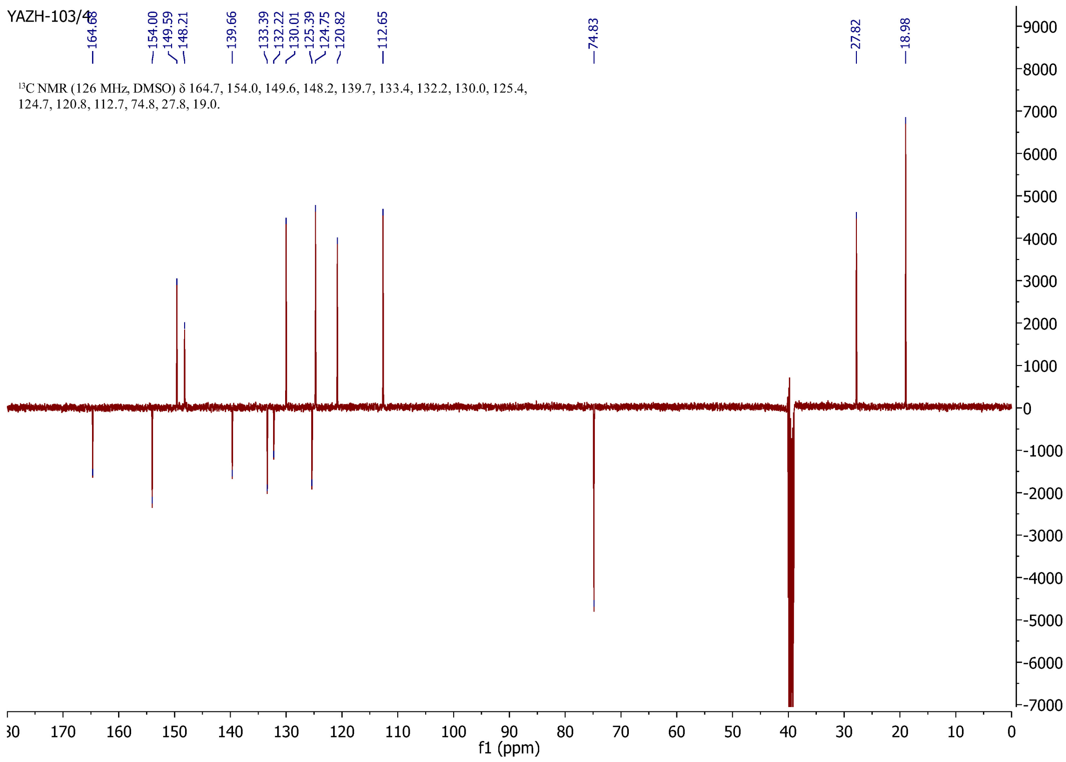

Figure S95 ^{13}C NMR spectrum of compound NPD-1202

Acquired by : Admin
Date Acquired : 1/26/2016 8:59:07 PM
Sample Name : Tamara compound 82
Sample ID :
Tray# : 1
Vial# : 32
Injection Volume : 1
Data File : C:\LabSolutions\Methods\FAR Pract Tamara compound 82.lcd
Background File : Blanco-2 26012016.lcd
Method File : Method SCAN ACID standard.lcm
Report Format : DefaultLCMS.lcr
Tuning File : C:\LabSolutions\Tuning File\Tuning-ESI-pos-neg01072015.lct
Processed by : Admin
Modified Date : 1/27/2016 10:15:46 AM

PDA Graph

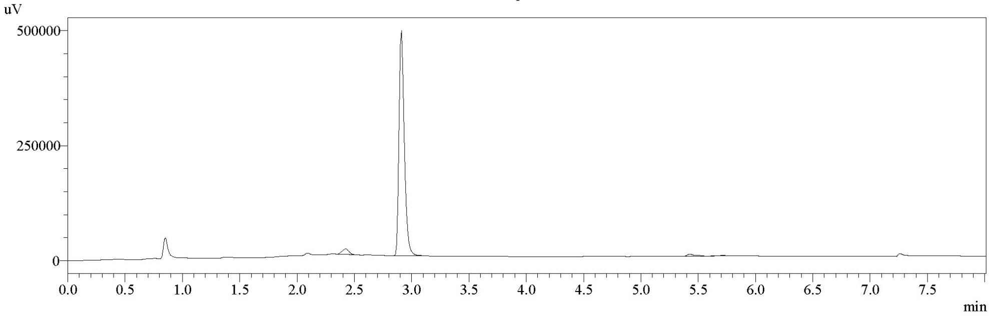

PDA Ch1 254nm 4nm

Peak#	Name	Ret. Time	Area	Area %
1		2.419	45731	2.788
2		2.904	1567771	95.589
3		5.422	20848	1.271
4		5.708	5769	0.352

MS Spectrum Graph

#:1 Ret.Time:Averaged 3.020-3.060(Scan#:302-306)
Mass Peaks:5 Base Peak:310.96(858231) Polarity:Pos Segment1 - Event1

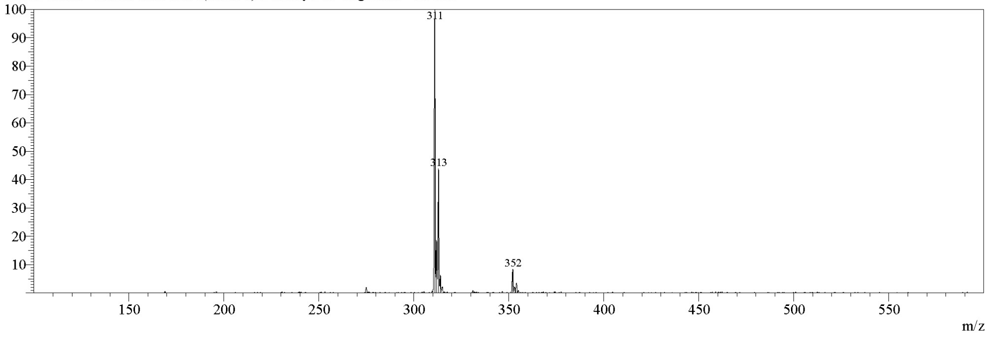

MS Spectrum Table

#:1 Ret.Time:
BG Mode:None
Mass Peaks:5 Base Peak:310.96(779830) Polarity:Pos Segment1 - Event

#	m/z	Abs.Inten.	Rel.Inten.	Charge	Polarity	Monoisotopic
1	310.96	779830	100.00			
2	311.89	138952	17.82			
3	312.95	320056	41.04			
4	313.95	43430	5.57			
5	352.02	70113	8.99			

Figure S96 LCMS spectrum of compound NPD-1203

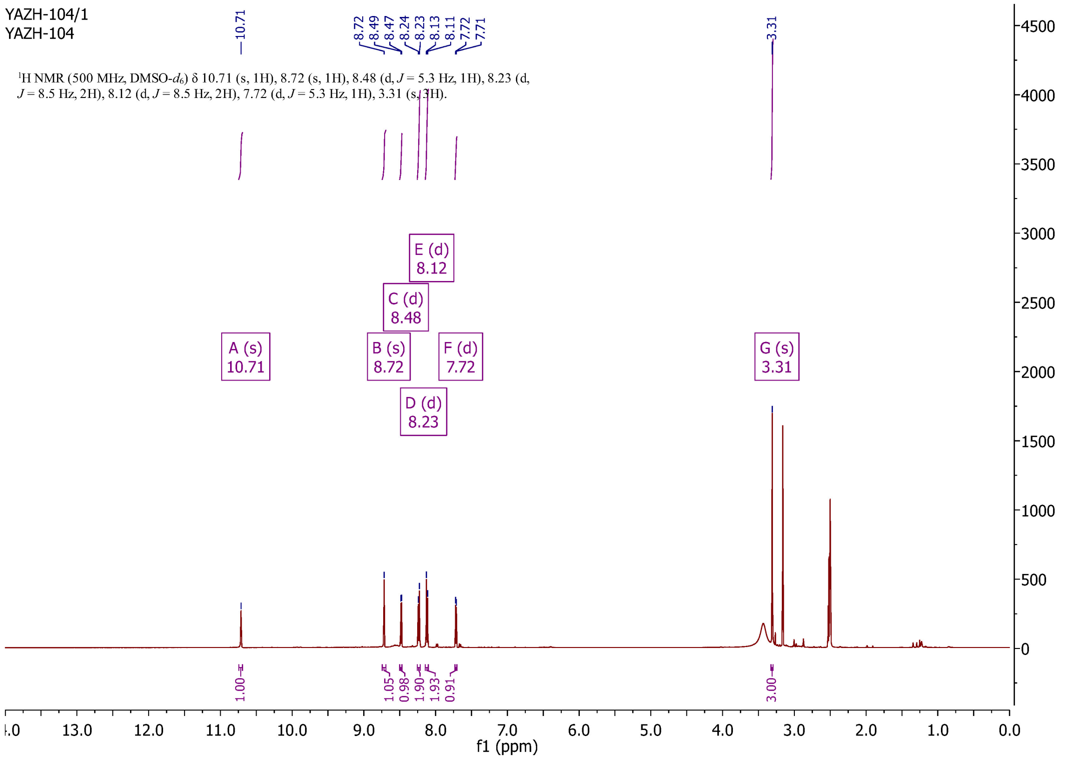

Figure S97 ^{1}H NMR spectrum of compound NPD-1203

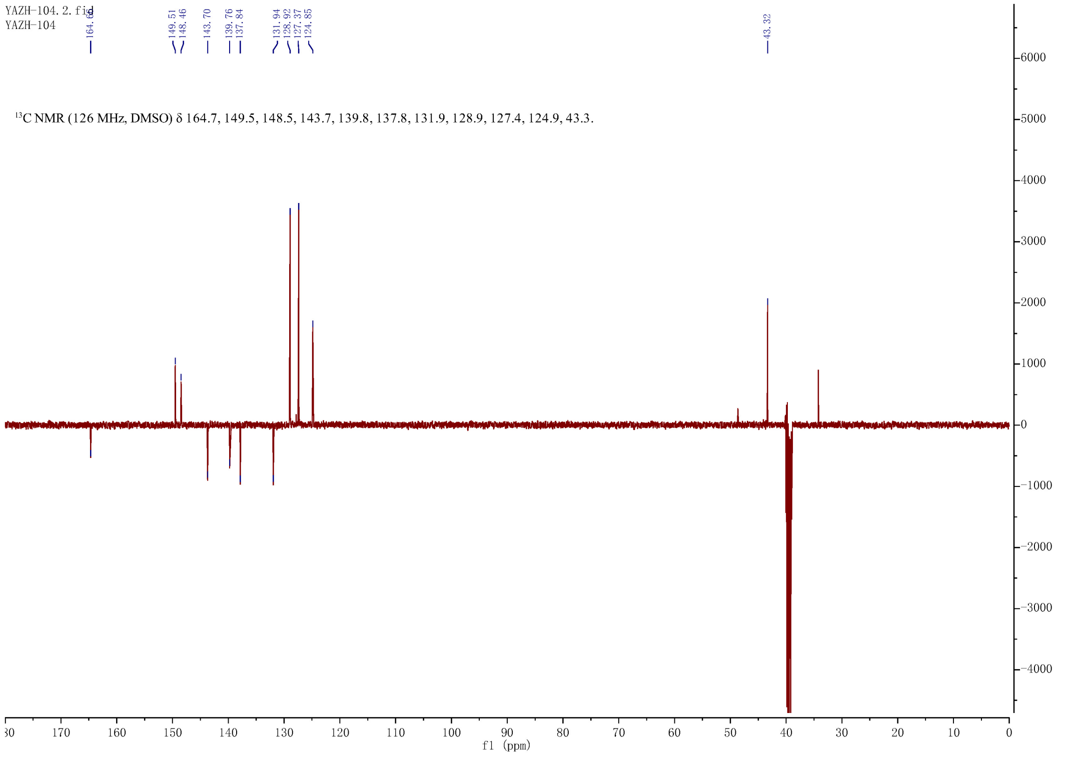

Figure S98 ^{13}C NMR spectrum of compound NPD-1203

Acquired by : Admin
Date Acquired : 1/26/2016 9:51:03 PM
Sample Name : Aron compound 85
Sample ID :
Tray# : 1
Vial# : 38
Injection Volume : 1
Data File : C:\LabSolutions\Methods\FAR Pract Aron compound 85.lcd
Background File : Blanco-2 26012016.lcd
Method File : Method SCAN ACID standard.lcm
Report Format : DefaultLCMS.lcr
Tuning File : C:\LabSolutions\Tuning File\Tuning-ESI-pos-neg01072015.lct
Processed by : Admin
Modified Date : 1/27/2016 10:50:49 AM

PDA Graph

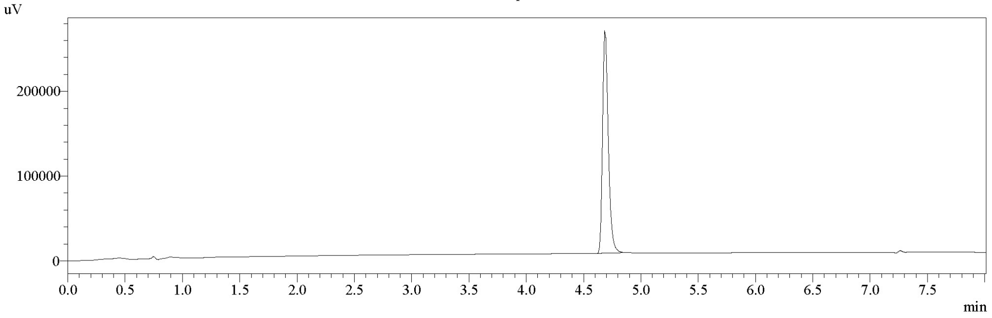

PDA Ch1 254nm 4nm

Peak#	Name	Ret. Time	Area	Area %
1		4.681	907011	100.000

MS Spectrum Graph

#:1 Ret.Time:Averaged 4.790-4.850(Scan#:479-485)
Mass Peaks:4 Base Peak:369.00(2431561) Polarity:Pos Segment1 - Event1

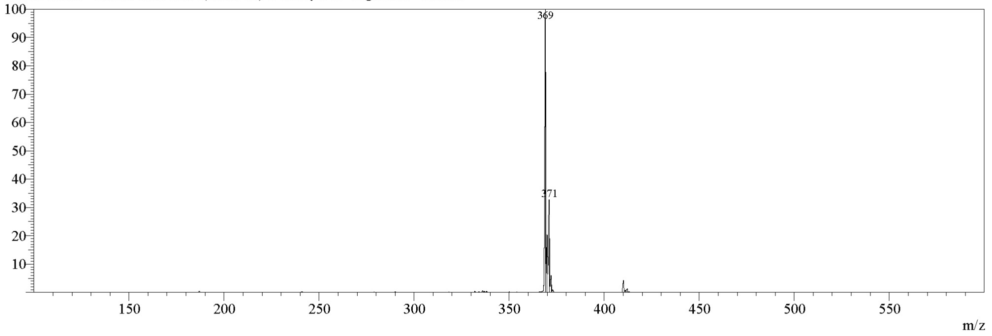

MS Spectrum Table

#:1 Ret.Time:
BG Mode:None
Mass Peaks:4 Base Peak:369.00(2276897) Polarity:Pos Segment1 - Event

#	m/z	Abs.Inten.	Rel.Inten.	Charge	Polarity	Monoisotopic
1	369.00	2276897	100.00			
2	369.93	458155	20.12			
3	371.06	734239	32.25			
4	372.06	124487	5.47			

Figure S99 LCMS spectrum of compound NPD-1204

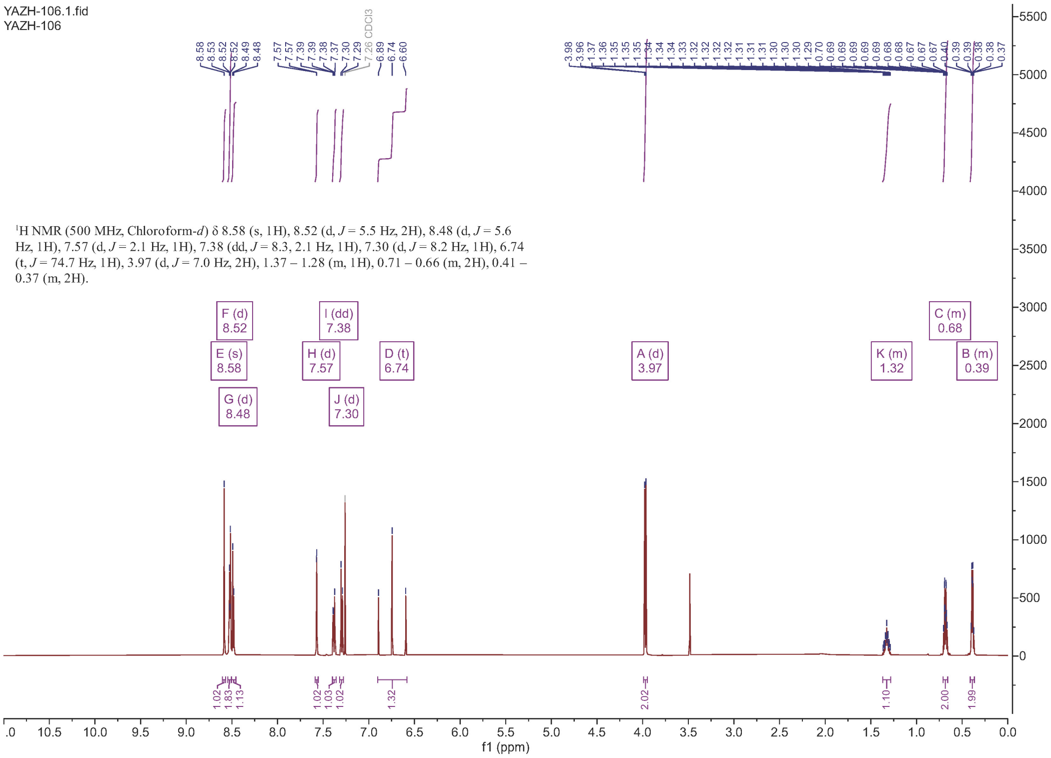

Figure S100 ^{1}H NMR spectrum of compound NPD-1204

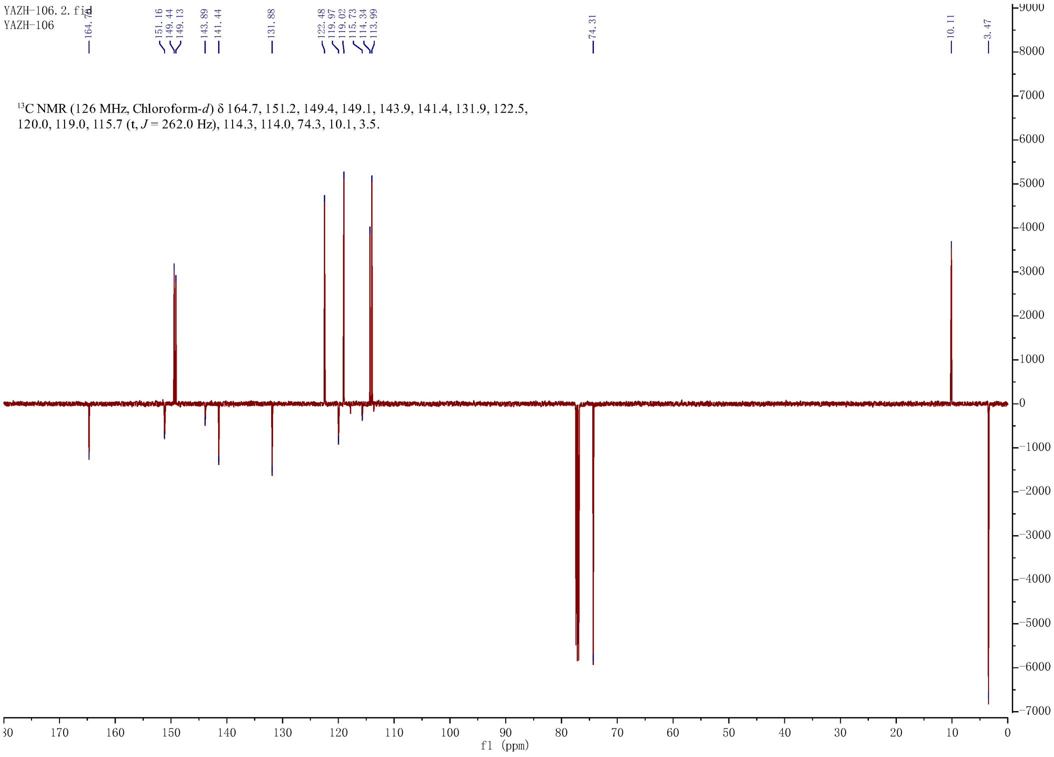

Figure S101 ^{13}C NMR spectrum of compound NPD-1204

Acquired by	: Admin
Date Acquired	: 3/5/2019 3:21:20 PM
Sample Name	: YAZH-107
Sample ID	:
Tray#	: 1
Vial#	: 9
Injection Volume	: 3
Data File	: C:\LabSolutions\Data\2019\2019-wk18\YAZH-107.lcd
Background File	: blanco 03052019.lcd
Method File	: Method SCAN ACID standard.lcm
Report Format	: DefaultLCMS.lcr
Tuning File	: C:\LabSolutions\Tuning\Tuning-ESI-pos-neg01072015a.lct
Processed by	: Admin
Modified Date	: 3/5/2019 3:48:58 PM

Medicinal Chemistry
Vrije Universiteit Amsterdam

Chromatogram
YAZH-107 C:\LabSolutions\Data\2019\2019-wk18\YAZH-107.lcd

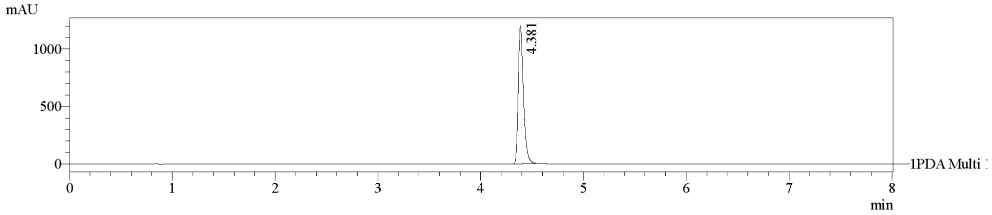

1 PDA Multi 1 / 254nm 4nm
PDA Ch1 254nm 4nm

Peak#	Name	Ret. Time	Area	Area %
1		4.381	4206090	100.000

MS Chromatogram
YAZH-107 C:\LabSolutions\Data\2019\2019-wk18\YAZH-107.lcd

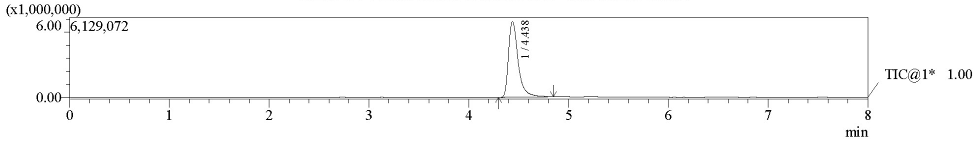

MS Spectrum Graph
#:1 Ret.Time:Averaged 4.430-4.450(Scan#:444-446)
Mass Peaks:8 Base Peak:369.05(3233991) Polarity:Pos Segment1 - Event1

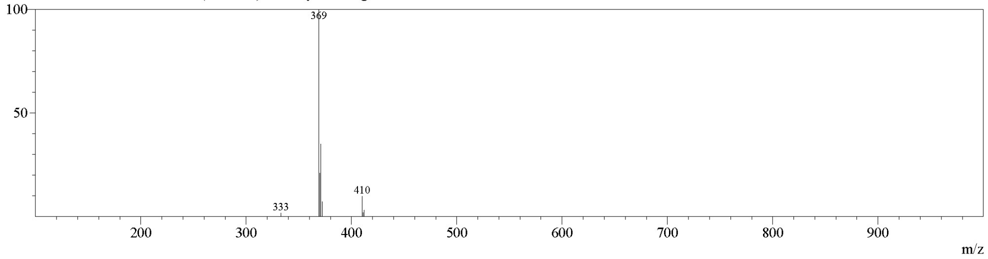

MS Spectrum Table
#:1 Ret.Time:
BG Mode:Calc 4.300<->4.850(431<->486)
Mass Peaks:8 Base Peak:369.05(3233991) Polarity:Pos Segment1 - Event1

#	m/z	Abs.Inten.	Rel.Inten.	Charge	Polarity	Monoisotopic	#	m/z	Abs.Inten.	Rel.Inten.	Charge	Polarity	Monoisotopic
1	333.00	54730	1.69				5	372.05	229595	7.10			
2	369.05	3233991	100.00				6	410.05	313094	9.68			
3	370.05	673836	20.84				7	411.10	58022	1.79			
4	371.05	1129791	34.93				8	412.05	100478	3.11			

Figure S102 LCMS spectrum of compound NPD-1205

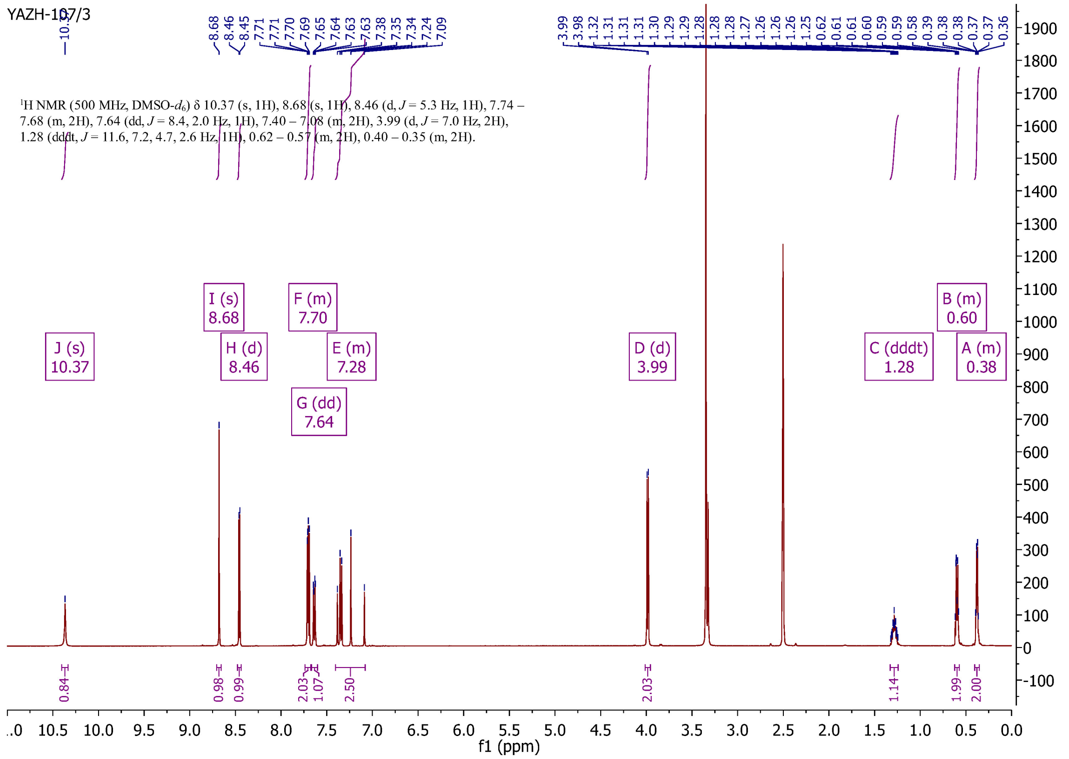

Figure S103 ^{1}H NMR spectrum of compound NPD-1205

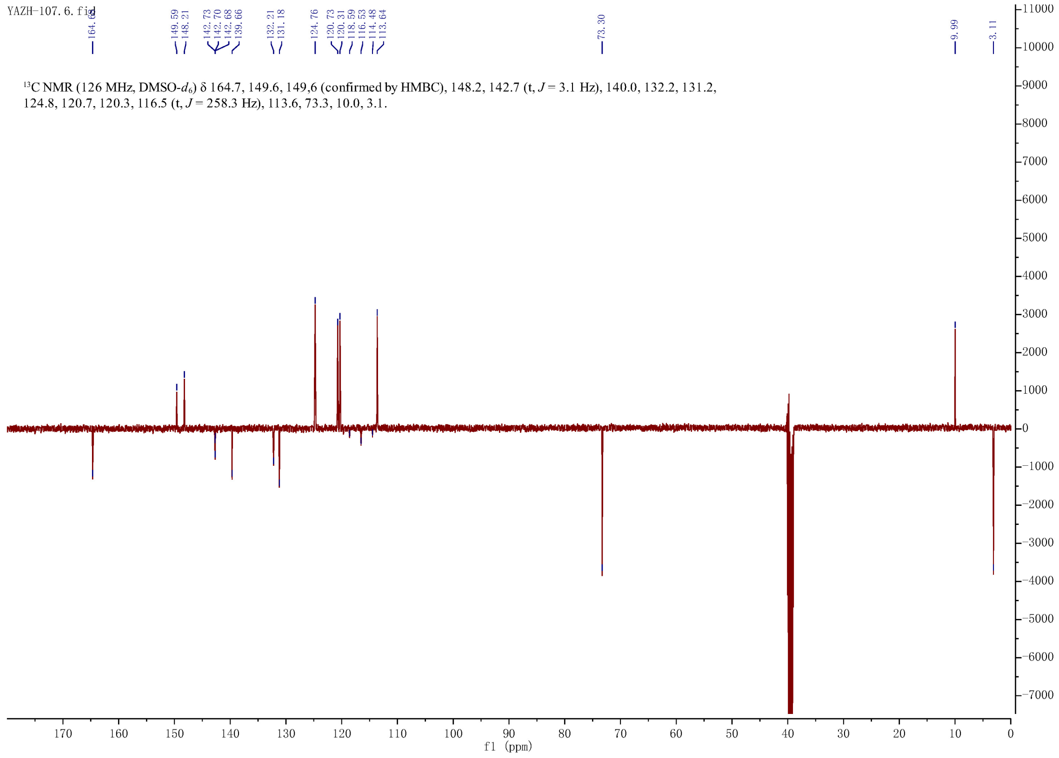

Figure S104 ^{13}C NMR spectrum of compound NPD-1205

Acquired by	: Admin
Date Acquired	: 1/26/2016 11:00:06 PM
Sample Name	: Robbert compound 77
Sample ID	:
Tray#	: 1
Vial#	: 46
Injection Volume	: 1
Data File	: C:\LabSolutions\Methods\FAR Pract Robbert compound 77.lcd
Background File	: Blanco-2 26012016.lcd
Method File	: Method SCAN ACID standard.lcm
Report Format	: DefaultLCMS.lcr
Tuning File	: C:\LabSolutions\Tuning File\Tuning-ESI-pos-neg01072015.lct
Processed by	: Admin
Modified Date	: 1/27/2016 11:35:49 AM

PDA Graph

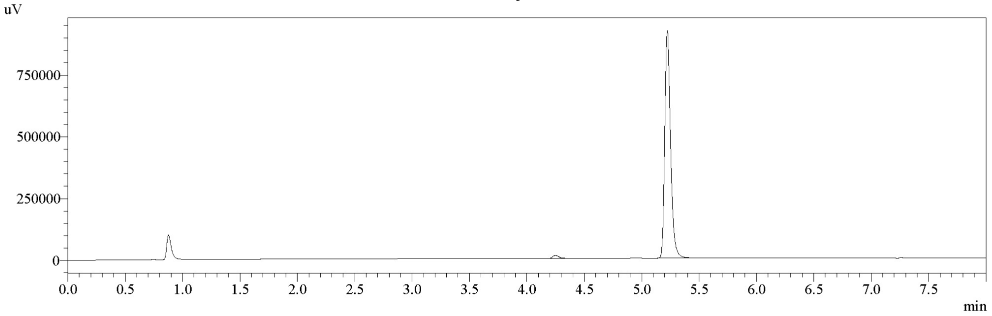

PDA Ch1 254nm 4nm

Peak#	Name	Ret. Time	Area	Area %
1		4.243	38819	1.185
2		5.218	3237832	98.815

MS Spectrum Graph

#:1 Ret.Time:Averaged 5.320-5.370(Scan#:532-537)
Mass Peaks:11 Base Peak:372.99(1220376) Polarity:Pos Segment1 - Event1

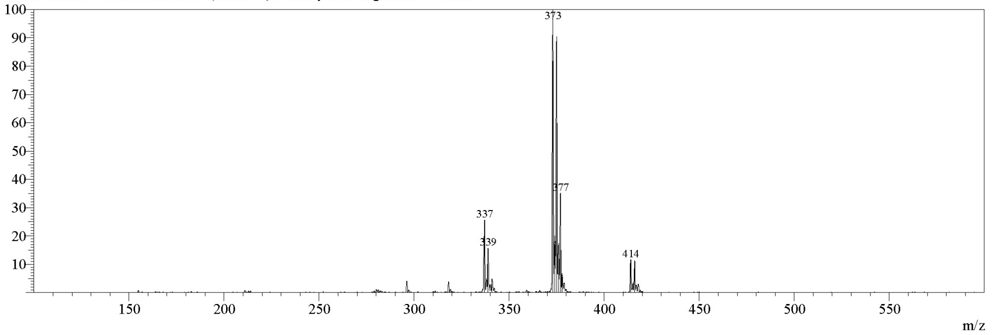

MS Spectrum Table

#:1 Ret.Time:
BG Mode:None
Mass Peaks:11 Base Peak:372.99(996605) Polarity:Pos Segment1 - Event

#	m/z	Abs.Inten.	Rel.Inten.	Charge	Polarity	Monoisotopic
1	337.05	259467	26.04			
2	338.91	157288	15.78			
3	340.97	58254	5.85			
4	372.99	996605	100.00			
5	374.06	205801	20.65			
6	374.99	921759	92.49			

#	m/z	Abs.Inten.	Rel.Inten.	Charge	Polarity	Monoisotopic
7	375.99	178375	17.90			
8	376.99	322428	32.35			
9	377.92	67191	6.74			
10	414.00	129525	13.00			
11	416.06	110035	11.04			

Figure S105 LCMS spectrum of compound NPD-1206

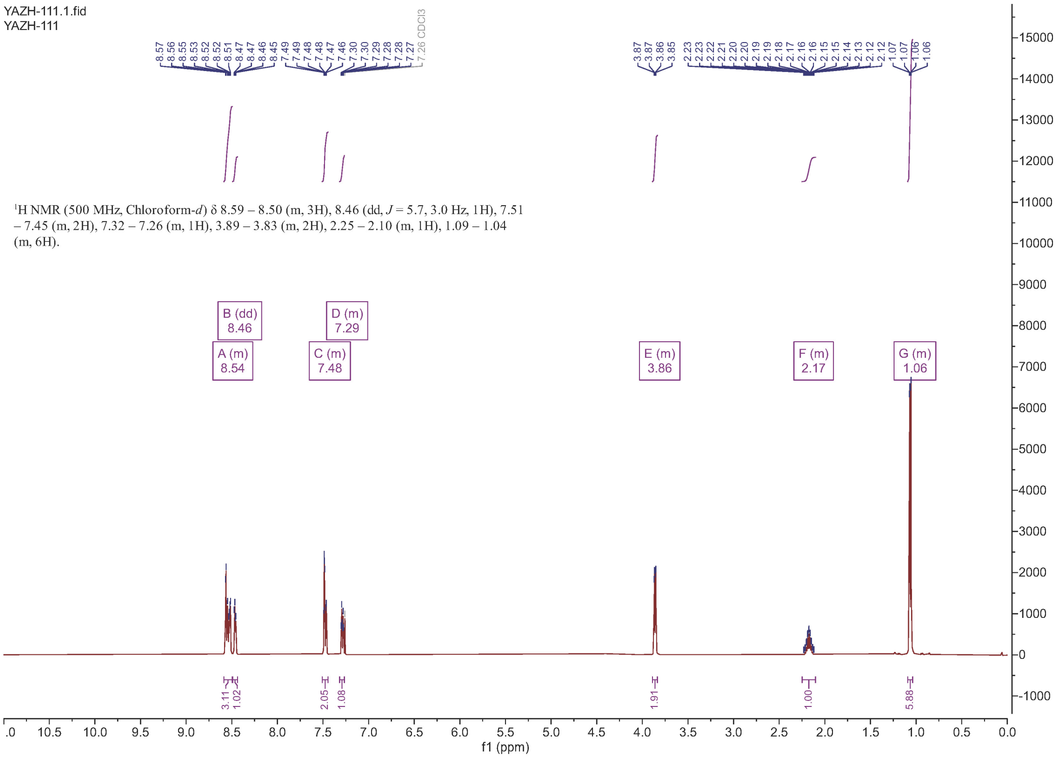

Figure S106 ^{1}H NMR spectrum of compound NPD-1206

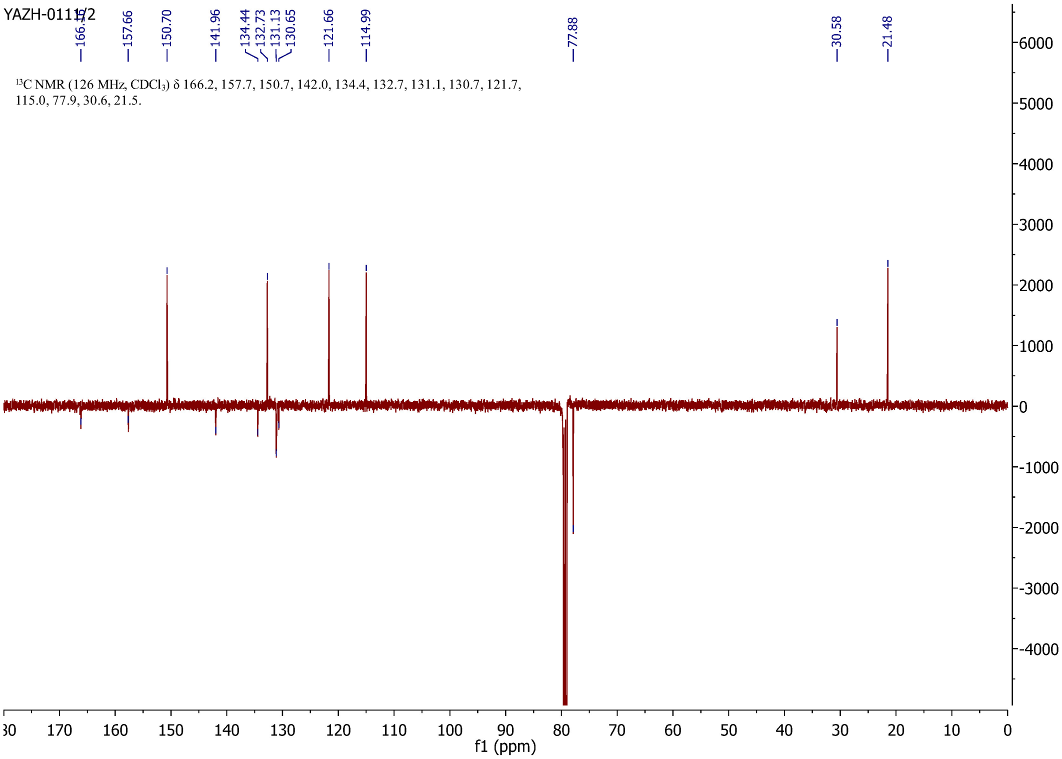

Figure S107 ^{13}C NMR spectrum of compound NPD-1206

Acquired by	: Admin
Date Acquired	: 6/9/2016 2:12:41 PM
Sample Name	: YAZH-145-2
Sample ID	:
Tray#	: 1
Vial#	: 43
Injection Volume	: 1
Data File	: C:\LabSolutions\Data\2016-wk23\YAZH-145-2.lcd
Background File	: BLANCO 09062016.lcd
Method File	: Method SCAN ACID standard.lcm
Report Format	: DefaultLCMS.lcr
Tuning File	: C:\LabSolutions\Tuning\Tuning-ESI-pos-neg01072015a.lct
Processed by	: Admin
Modified Date	: 6/9/2016 3:07:45 PM

PDA Graph

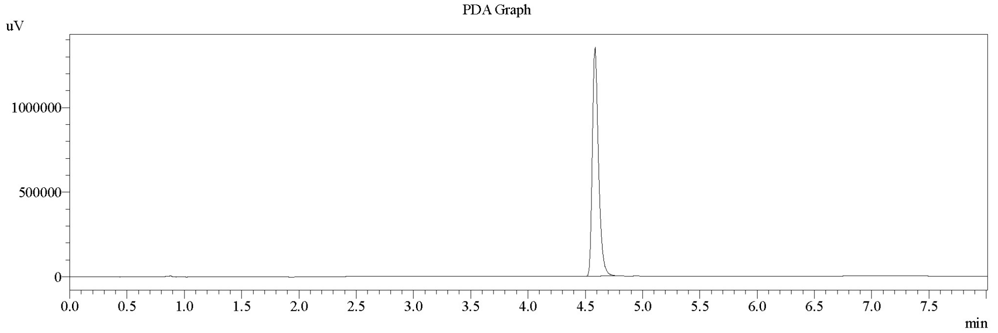

PDA Ch1 254nm 4nm

Peak#	Name	Ret. Time	Area	Area %
1		4.579	5081780	100.000

MS Spectrum Graph

#:1 Ret.Time:Averaged 4.650-4.670(Scan#:466-468)
Mass Peaks:10 Base Peak:327.95(881302) Polarity:Pos Segment1 - Event1

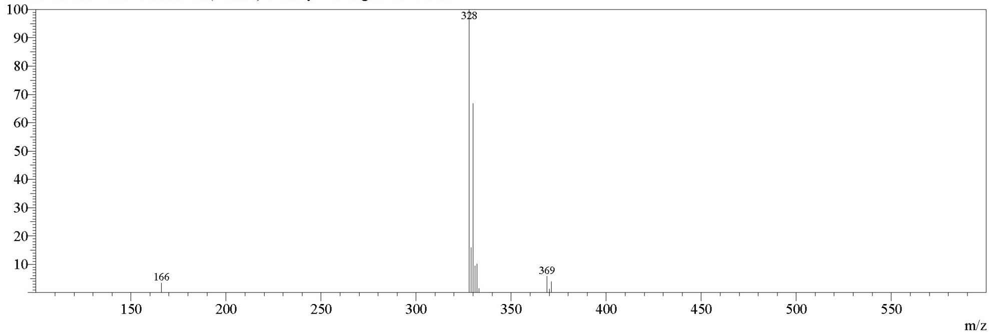

MS Spectrum Table

#:1 Ret.Time:
BG Mode:Calc 4.540<->4.990(455<->500)
Mass Peaks:10 Base Peak:327.95(881302) Polarity:Pos Segment1 - Event1

#	m/z	Abs.Inten.	Rel.Inten.	Charge	Polarity	Monoisotopic	#	m/z	Abs.Inten.	Rel.Inten.	Charge	Polarity	Monoisotopic
1	165.95	29510	3.35				6	332.00	89644	10.17			
2	327.95	881302	100.00				7	333.00	13321	1.51			
3	329.00	139315	15.81				8	368.95	50560	5.74			
4	329.95	589330	66.87				9	370.00	10710	1.22			
5	331.00	82638	9.38				10	371.00	33942	3.85			

Figure S108 LCMS spectrum of compound NPD-1472

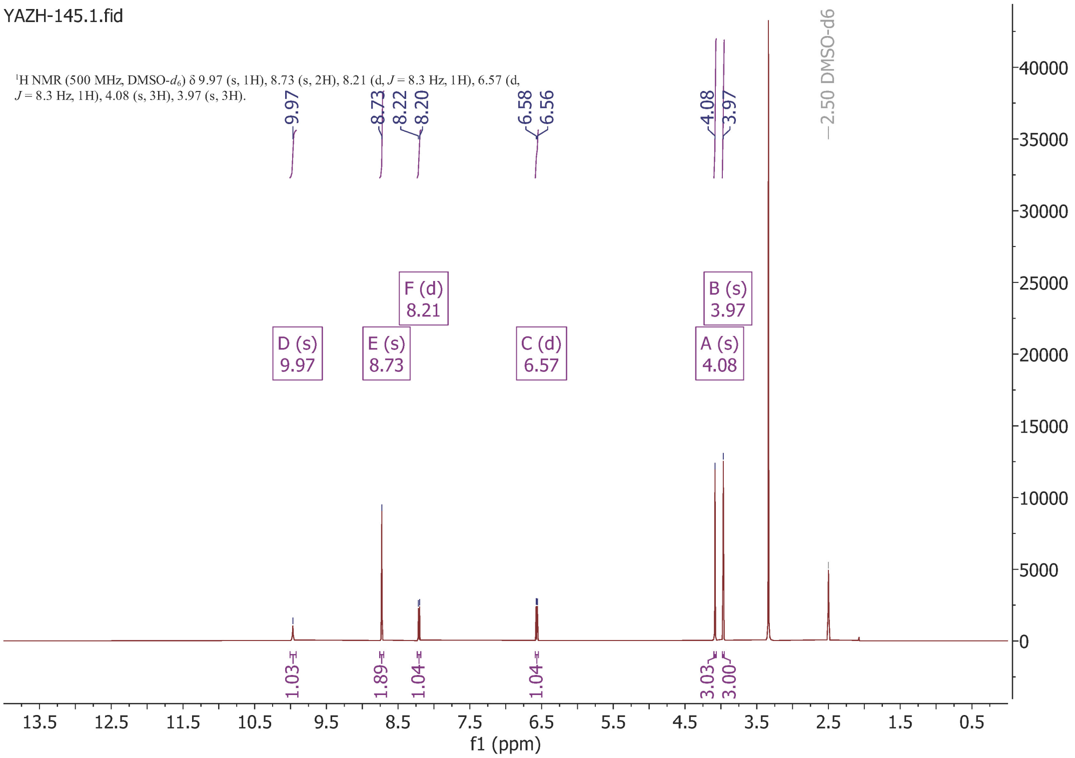

Figure S109 ^{1}H NMR spectrum of compound NPD-1472

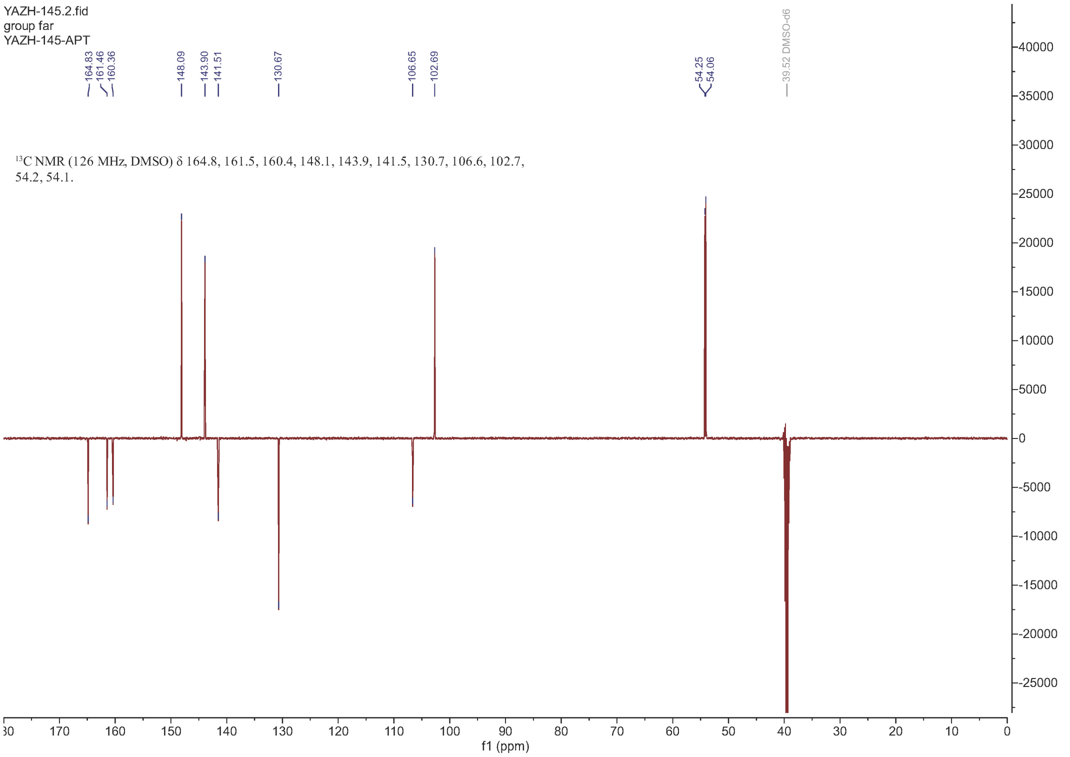

Figure S110 ^{13}C NMR spectrum of compound NPD-1472

Medicinal Chemistry
Vrije Universiteit Amsterdam

Acquired by	: Admin
Date Acquired	: 10/5/2019 3:59:27 PM
Sample Name	: YAZH-018
Sample ID	:
Tray#	: 1
Vial#	: 37
Injection Volume	: 3
Data File	: C:\LabSolutions\Data\2019\2019-wk19\YAZH-018.lcd
Background File	: blanco 10052019.lcd
Method File	: Method SCAN ACID standard.lcm
Report Format	: DefaultLCMS.lcr
Tuning File	: C:\LabSolutions\Tuning\Tuning-ESI-pos-neg01072015a.lct
Processed by	: Admin
Modified Date	: 10/5/2019 4:21:59 PM

PDA Graph

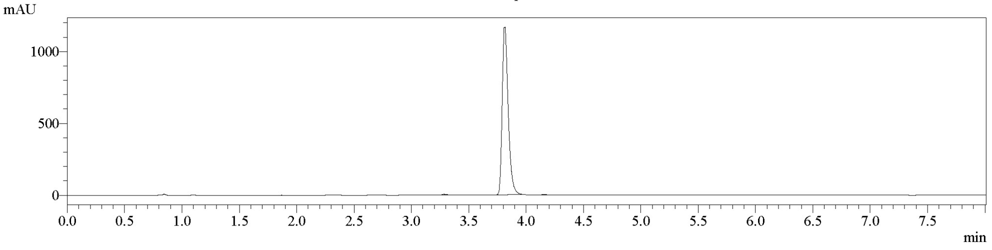

PeakTable

PDA Ch1 254nm 4nm

Peak#	Name	Ret. Time	Area	Area %
1		3.281	21243	0.502
2		3.808	4200066	99.252
3		4.153	10397	0.246

MS Chromatogram
YAZH-018 C:\LabSolutions\Data\2019\2019-wk19\YAZH-018.lcd

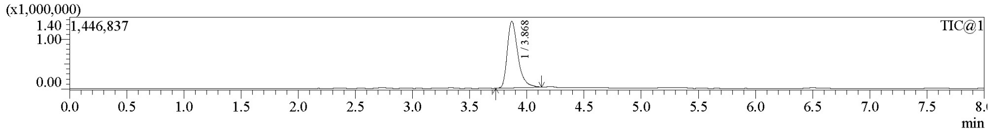

MS Spectrum Graph

#:1 Ret.Time:Averaged 3.860-3.880(Scan#:387-389)
BG Mode:Calc 3.730<->4.130(374<->414)
Mass Peaks:9 Base Peak:341.00(627181) Polarity:Pos Segment1 - Event1

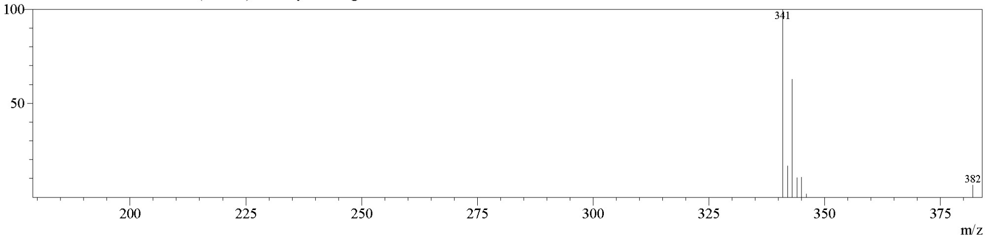

MS Spectrum Table

#:1 Ret.Time:
BG Mode:Calc 3.730<->4.130(374<->414)
Mass Peaks:9 Base Peak:341.00(627181) Polarity:Pos Segment1 - Event1

#	m/z	Abs.Inten.	Rel.Inten.	Charge	Polarity	Monoisotopic
1	179.00	23933	3.82			
2	341.00	627181	100.00			
3	342.05	105030	16.75			
4	343.00	394560	62.91			
5	344.05	65299	10.41			
6	345.05	66619	10.62			
7	346.05	11028	1.76			
8	382.00	40100	6.39			
9	384.05	27033	4.31			

Figure S111 LCMS spectrum of compound NPD-2976

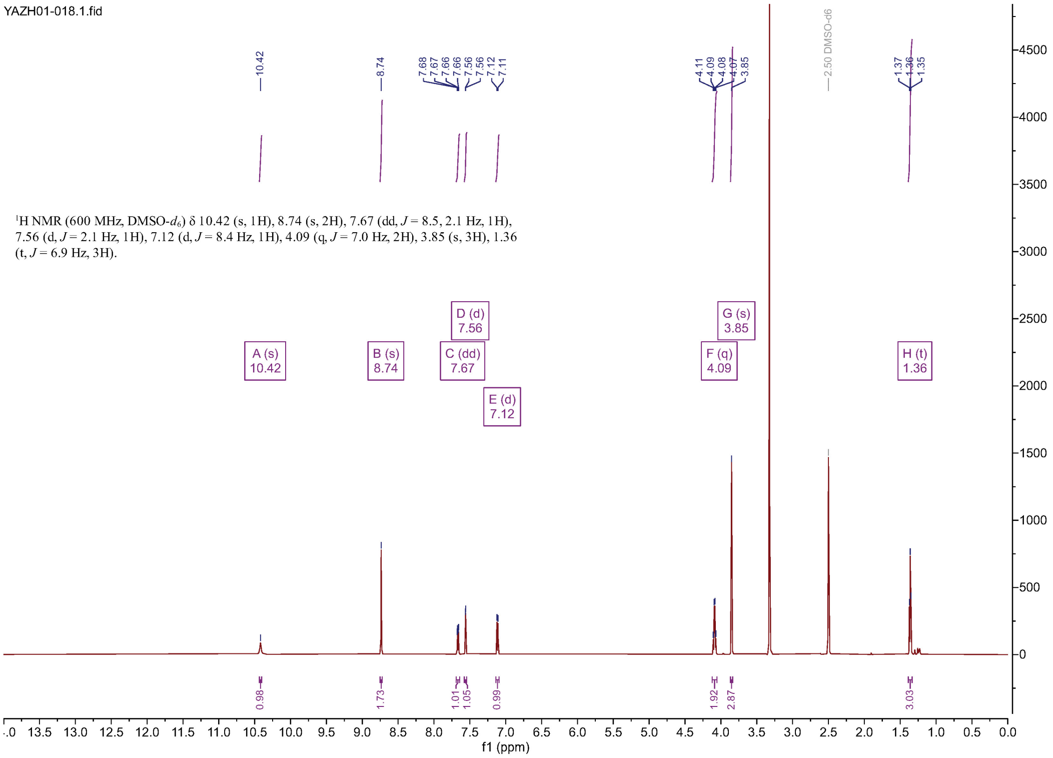

^{1}H NMR (600 MHz, DMSO-d_6) δ 10.42 (s, 1H), 8.74 (s, 2H), 7.67 (dd, J = 8.5, 2.1 Hz, 1H), 7.56 (d, J = 2.1 Hz, 1H), 7.12 (d, J = 8.4 Hz, 1H), 4.09 (q, J = 7.0 Hz, 2H), 3.85 (s, 3H), 1.36 (t, J = 6.9 Hz, 3H).

Figure S112 ^{1}H NMR spectrum of compound NPD-2976

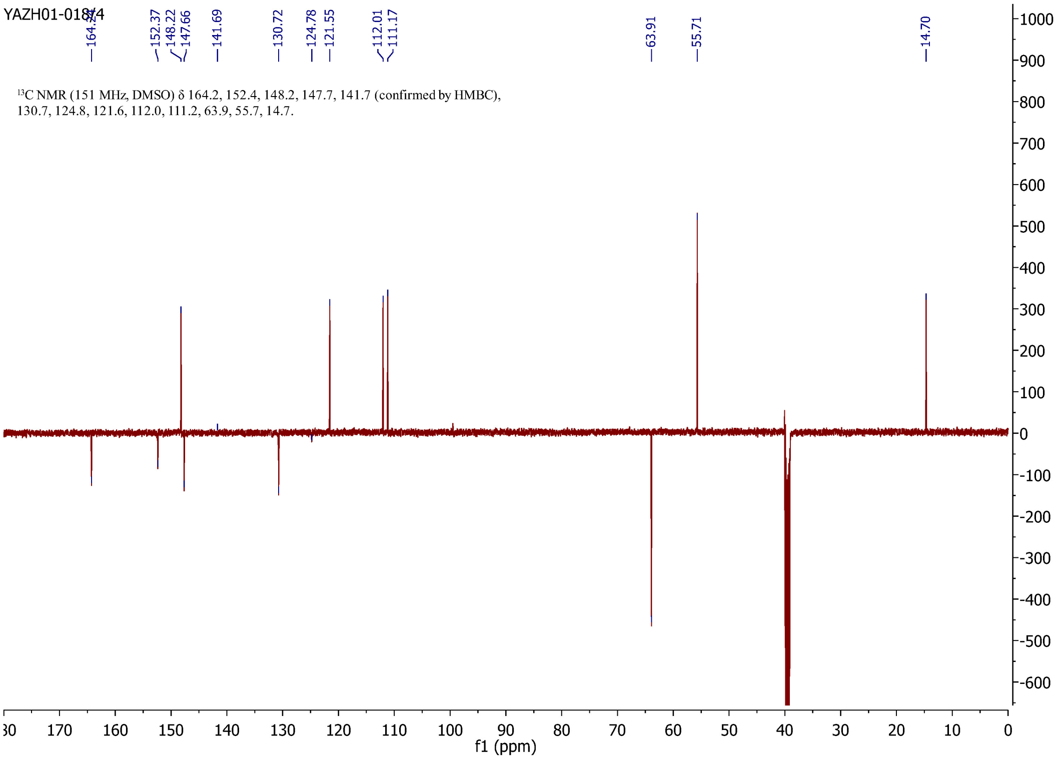

^{13}C NMR (151 MHz, DMSO) δ 164.2, 152.4, 148.2, 147.7, 141.7 (confirmed by HMBC), 130.7, 124.8, 121.6, 112.0, 111.2, 63.9, 55.7, 14.7.

Figure S113 ^{13}C NMR spectrum of compound NPD-2976

Acquired by	: Admin
Date Acquired	: 1/23/2017 10:39:54 AM
Sample Name	: YAZH01-025
Sample ID	:
Tray#	: 1
Vial#	: 14
Injection Volume	: 1
Data File	: C:\LabSolutions\Data\2017\2017-wk04\YAZH01-025.lcd
Background File	: Blanco23012017.lcd
Method File	: Method SCAN ACID standard.lcm
Report Format	: DefaultLCMS.lcr
Tuning File	: C:\LabSolutions\Tuning\Tuning-ESI-pos-neg01072015a.lct
Processed by	: Admin
Modified Date	: 1/23/2017 11:27:17 AM

PDA Graph

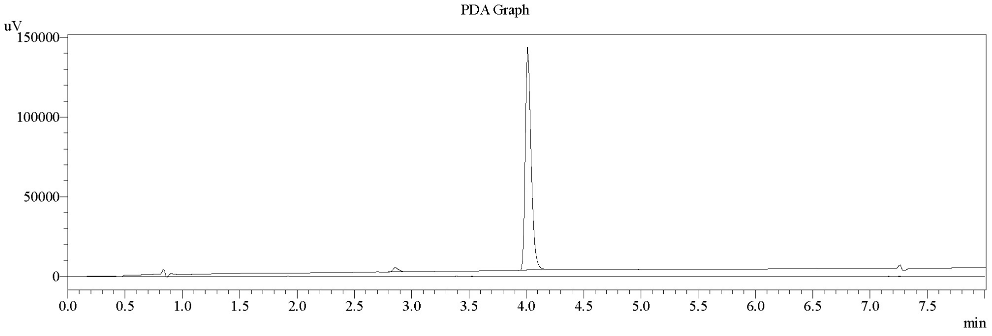

PDA Ch1 254nm 4nm

Peak#	Name	Ret. Time	Area	Area %
1		2.853	9126	1.816
2		4.007	493479	98.184

MS Spectrum Graph

#:1 Ret.Time:Averaged 4.060-4.080(Scan#:407-409)
Mass Peaks:12 Base Peak:419.05(158506) Polarity:Pos Segment1 - Event1

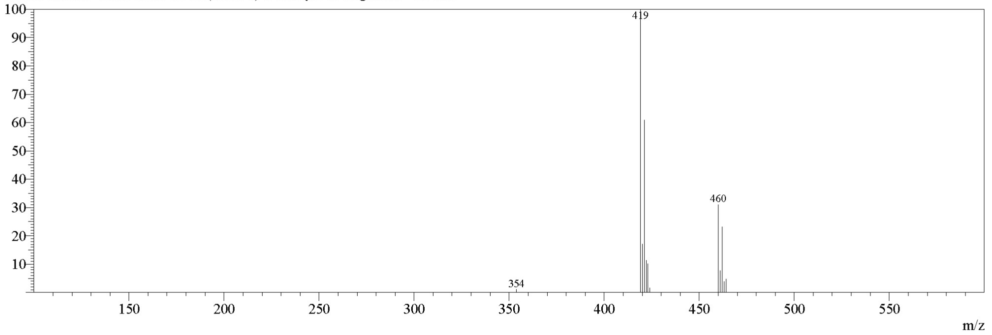

MS Spectrum Table

#:1 Ret.Time:
BG Mode:Calc 3.960<->4.240(397<->425)
Mass Peaks:12 Base Peak:419.05(158506) Polarity:Pos Segment1 - Event1

#	m/z	Abs.Inten.	Rel.Inten.	Charge	Polarity	Monoisotopic
1	353.70	1674	1.06			
2	419.05	158506	100.00			
3	420.15	27037	17.06			
4	421.10	96492	60.88			
5	422.10	17895	11.29			
6	423.00	16159	10.19			
7	424.10	2631	1.66			
8	460.10	49139	31.00			
9	461.15	12270	7.74			
10	462.15	36868	23.26			
11	463.10	6115	3.86			
12	464.15	7568	4.77			

Figure S114 LCMS spectrum of compound NPD-2980

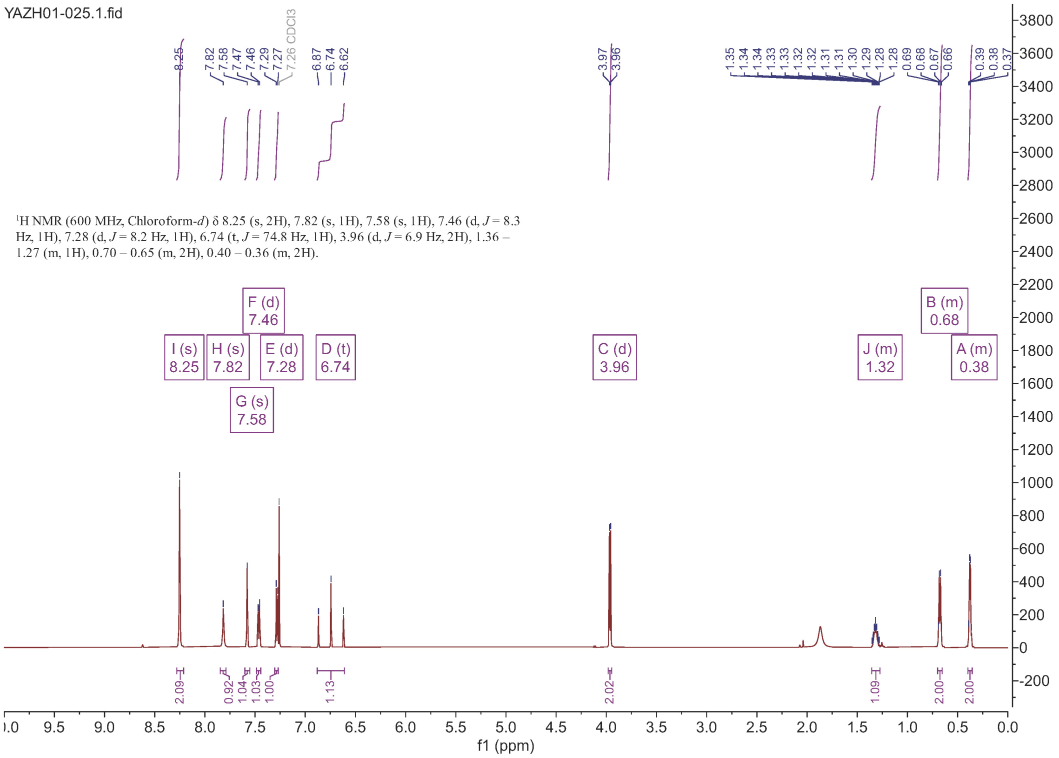

Figure S115 ^{1}H NMR spectrum of compound NPD-2980

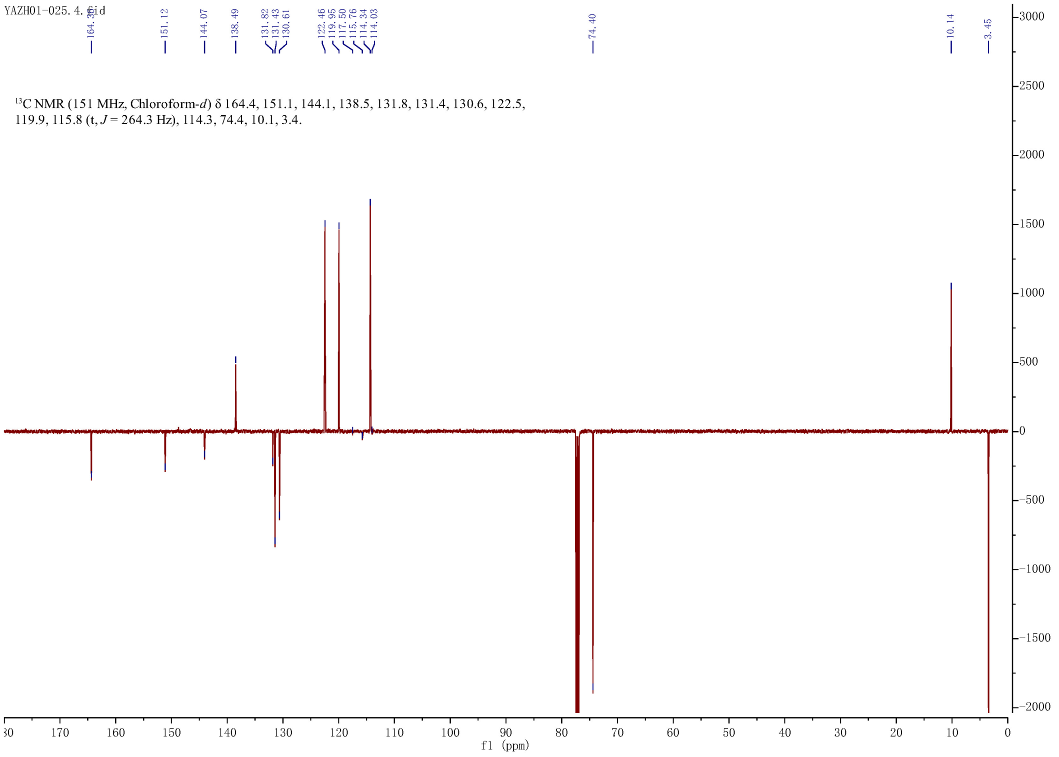

Figure S116 ^{13}C NMR spectrum of compound NPD-2980

Medicinal Chemistry
Vrije Universiteit Amsterdam

Acquired by	: Admin
Date Acquired	: 13/5/2019 9:26:18 AM
Sample Name	: YAZH01-027
Sample ID	:
Tray#	: 1
Vial#	: 2
Injection Volume	: 3
Data File	: C:\LabSolutions\Data\2019\2019-wk20\YAZH01-027.lcd
Background File	: blanco 13052019.lcd
Method File	: Method SCAN ACID standard.lcm
Report Format	: DefaultLCMS.lcr
Tuning File	: C:\LabSolutions\Tuning\Tuning-ESI-pos-neg01072015a.lct
Processed by	: Admin
Modified Date	: 13/5/2019 10:56:18 AM

PDA Graph

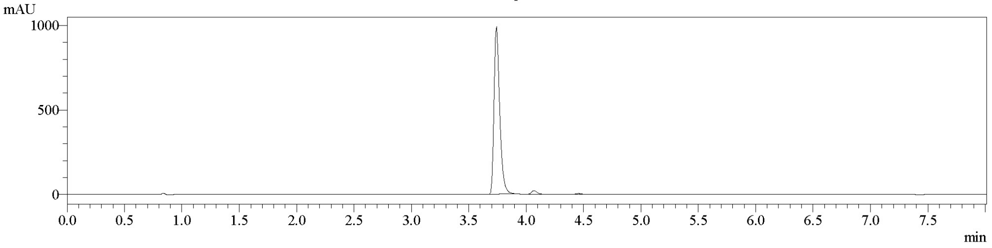

PeakTable

PDA Ch1 254nm 4nm

Peak#	Name	Ret. Time	Area	Area %
1		3.232	7898	0.224
2		3.735	3426277	97.259
3		4.061	64224	1.823
4		4.449	17567	0.499
5		4.648	2364	0.067
6		4.853	4499	0.128

MS Chromatogram
YAZH01-027 C:\LabSolutions\Data\2019\2019-wk20\YAZH01-027.lcd

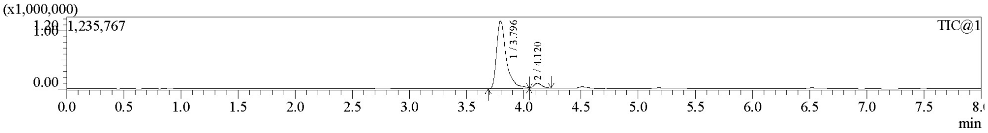

MS Spectrum Graph

#:1 Ret.Time:Averaged 3.790-3.810(Scan#:380-382)
BG Mode:Calc 3.690<->4.050(370<->406)
Mass Peaks:20 Base Peak:397.05(366130) Polarity:Pos Segment1 - Event1

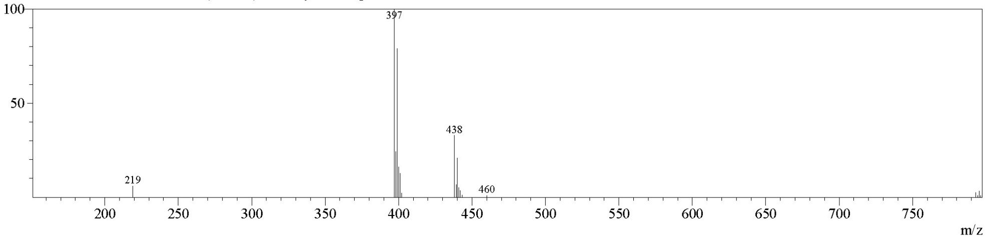

MS Spectrum Table

#:1 Ret.Time:
BG Mode:Calc 3.690<->4.050(370<->406)
Mass Peaks:20 Base Peak:397.05(366130) Polarity:Pos Segment1 - Event1

#	m/z	Abs.Inten.	Rel.Inten.	Charge	Polarity	Monoisotopic
1	150.95	16336	4.46			
2	219.10	21867	5.97			
3	397.05	366130	100.00			
4	398.10	88607	24.20			
5	399.10	288995	78.93			
6	400.10	58695	16.03			
7	401.10	46866	12.80			
8	402.10	8046	2.20			
9	438.10	120100	32.80			
10	439.15	24040	6.57			
11	440.15	75756	20.69			
12	441.15	18221	4.98			
13	442.15	12605	3.44			
14	443.20	4145	1.13			
15	460.15	3774	1.03			
16	793.10	8735	2.39			
17	794.30	3937	1.08			
18	795.30	12281	3.35			

Figure S117 LCMS spectrum of compound NPD-2981

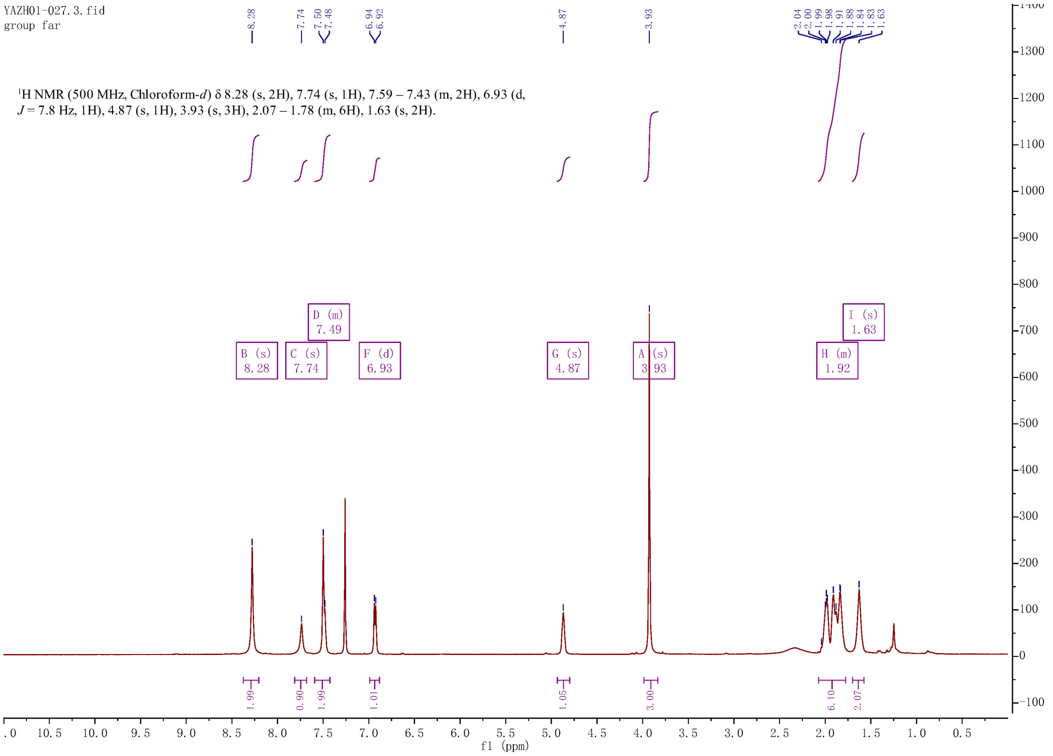

Figure S118 ^{1}H NMR spectrum of compound NPD-2981

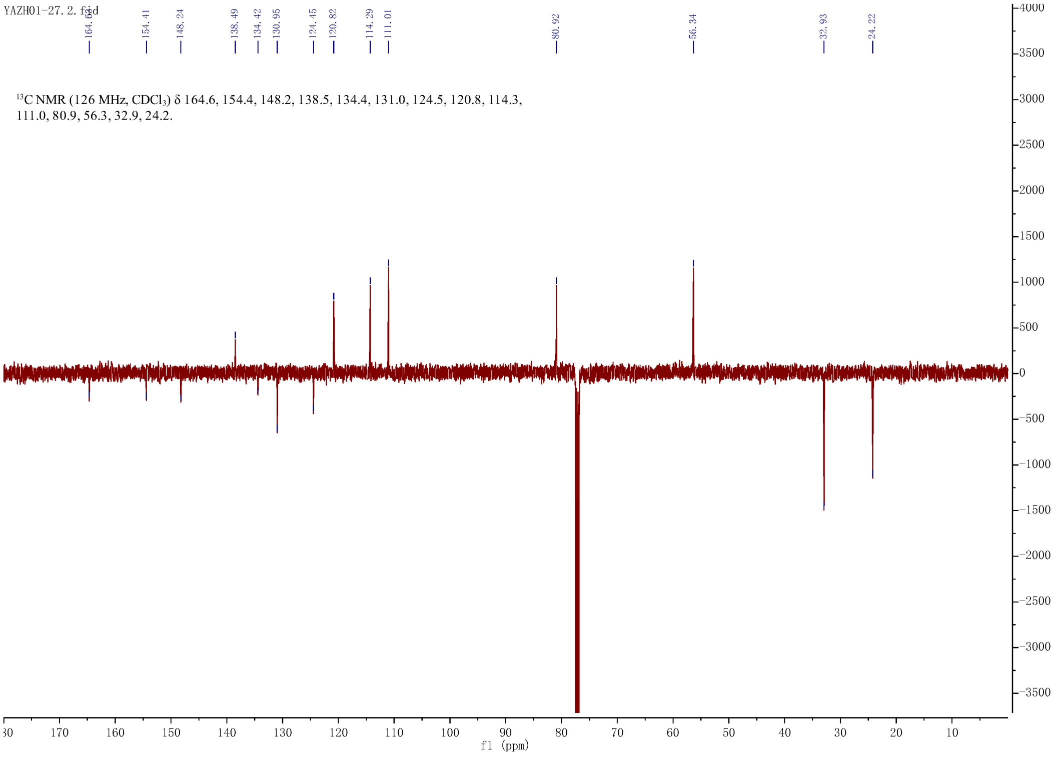

Figure S119 ^{13}C NMR spectrum of compound NPD-2981

Acquired by	: Admin
Date Acquired	: 2/22/2017 9:26:11 AM
Sample Name	: Practical Course-116
Sample ID	:
Tray#	: 1
Vial#	: 17
Injection Volume	: 1
Data File	: C:\LabSolutions\Data\2017\2017-wk08\Practical Course-116.lcd
Background File	: blanco 22022017.lcd
Method File	: Method SCAN ACID standard.lcm
Report Format	: DefaultLCMS.lcr
Tuning File	: C:\LabSolutions\Tuning\Tuning-ESI-pos-neg01072015a.lct
Processed by	: Admin
Modified Date	: 2/22/2017 11:38:57 AM

PDA Graph

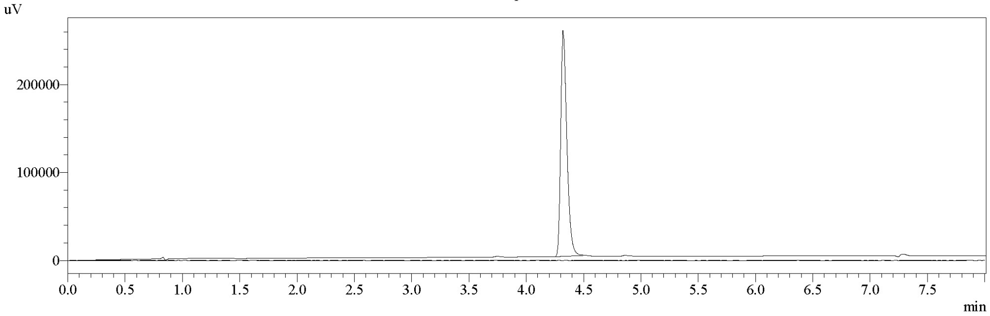

PDA Ch1 254nm 4nm

Peak#	Name	Ret. Time	Area	Area %
1		4.318	962680	100.000

MS Spectrum Graph

#:1 Ret.Time:Averaged 4.360-4.380(Scan#:437-439)
Mass Peaks:6 Base Peak:292.05(217591) Polarity:Pos Segment1 - Event1

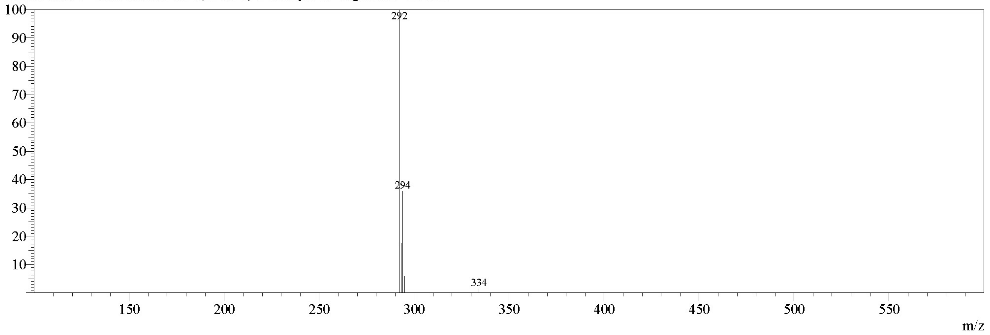

MS Spectrum Table

#:1 Ret.Time:
BG Mode:Calc 4.270<->4.520(428<->453)
Mass Peaks:6 Base Peak:292.05(217591) Polarity:Pos Segment1 - Event1

#	m/z	Abs.Inten.	Rel.Inten.	Charge	Polarity	Monoisotopic
1	292.05	217591	100.00			
2	293.05	37967	17.45			
3	294.05	78124	35.90			
4	295.10	12584	5.78			
5	333.15	2847	1.31			
6	334.20	3111	1.43			

Figure S120 LCMS spectrum of compound NPD-2991

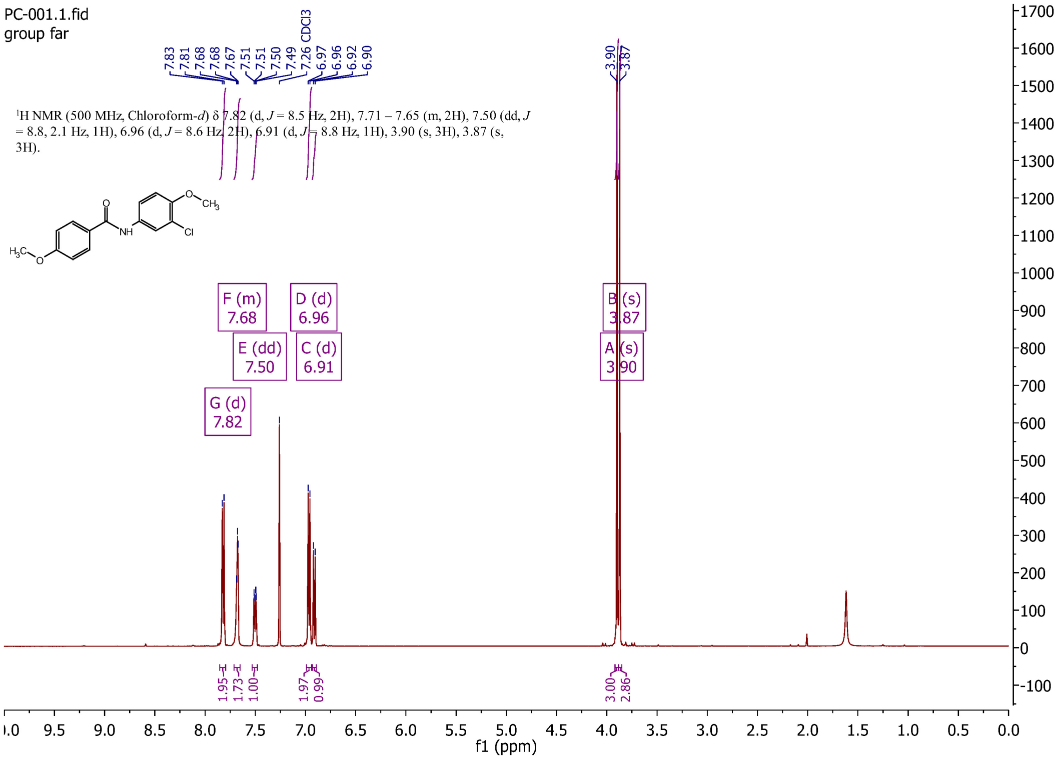

Figure S121 ^{1}H NMR spectrum of compound NPD-2991

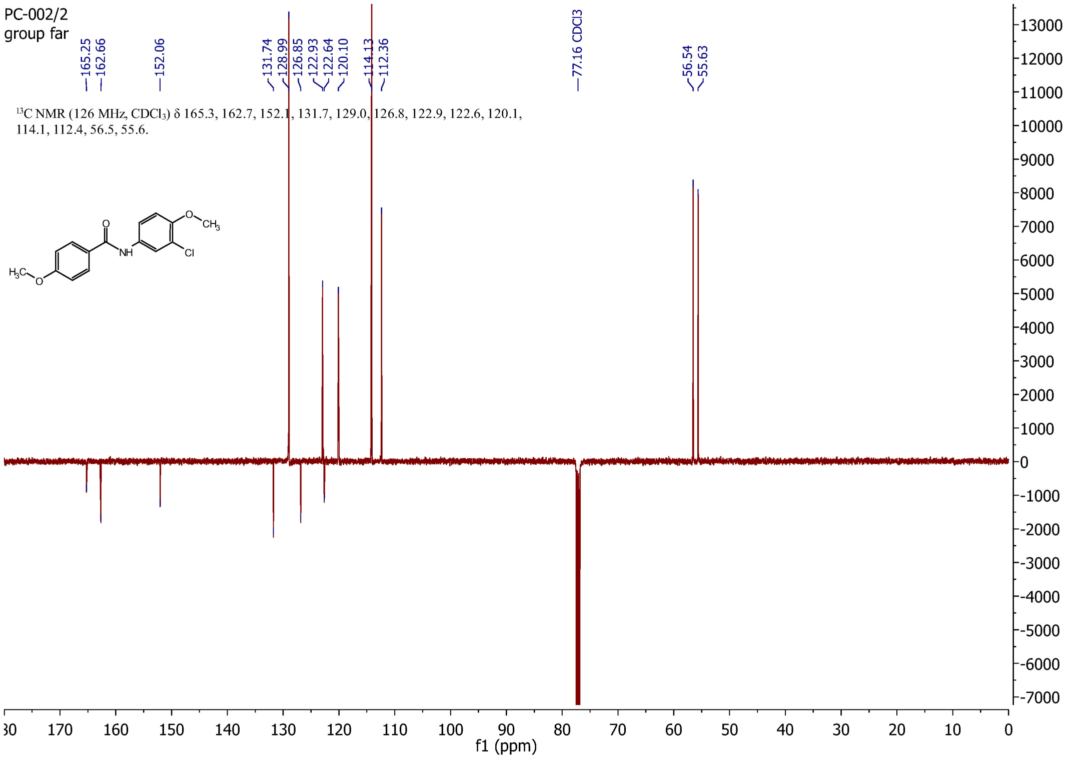

Figure S122 ^{13}C NMR spectrum of compound NPD-2991

Acquired by	: Admin
Date Acquired	: 2/22/2017 9:34:48 AM
Sample Name	: Practical Course-118
Sample ID	:
Tray#	: 1
Vial#	: 18
Injection Volume	: 1
Data File	: C:\LabSolutions\Data\2017\2017-wk08\Practical Course-118.lcd
Background File	: blanco 22022017.lcd
Method File	: Method SCAN ACID standard.lcm
Report Format	: DefaultLCMS.lcr
Tuning File	: C:\LabSolutions\Tuning\Tuning-ESI-pos-neg01072015a.lct
Processed by	: Admin
Modified Date	: 2/22/2017 11:39:43 AM

PDA Graph

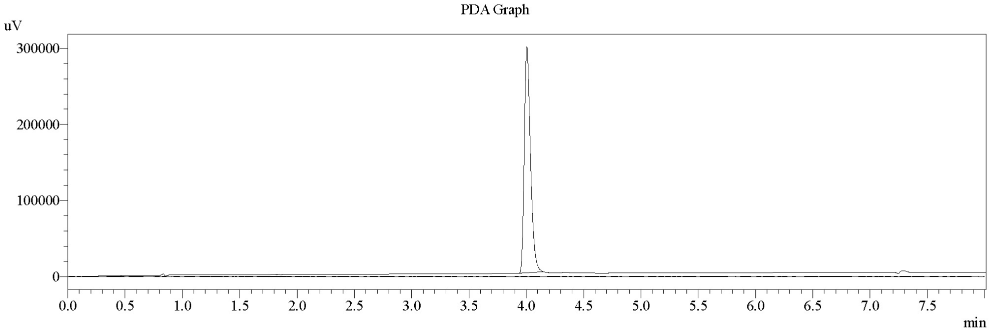

PDA Ch1 254nm 4nm

Peak#	Name	Ret. Time	Area	Area %
1		4.000	1123956	100.000

MS Spectrum Graph

#:1 Ret.Time:Averaged 4.050-4.070(Scan#:406-408)
Mass Peaks:19 Base Peak:297.00(300798) Polarity:Pos Segment1 - Event1

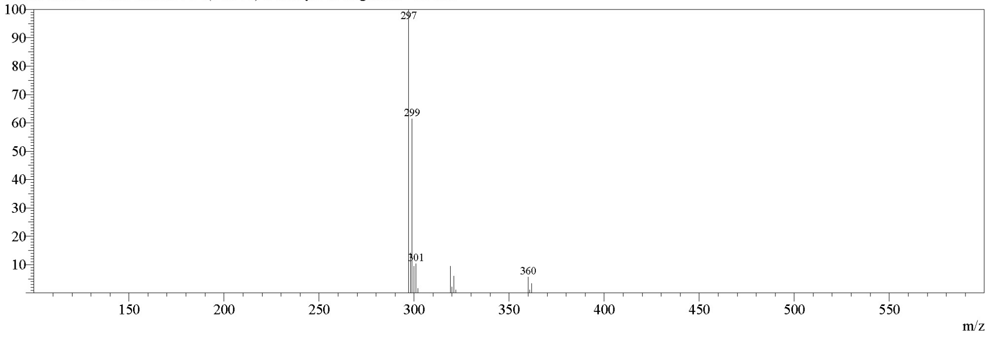

MS Spectrum Table

#:1 Ret.Time:
BG Mode:Calc 3.930<->4.310(394<->432)
Mass Peaks:19 Base Peak:297.00(300798) Polarity:Pos Segment1 - Event1

#	m/z	Abs.Inten.	Rel.Inten.	Charge	Polarity	Monoisotopic
1	297.00	300798	100.00			
2	298.00	42559	14.15			
3	299.00	184817	61.44			
4	299.95	28497	9.47			
5	301.05	30936	10.28			
6	301.95	4667	1.55			
7	319.00	28681	9.53			
8	320.00	6262	2.08			
9	320.95	17935	5.96			

#	m/z	Abs.Inten.	Rel.Inten.	Charge	Polarity	Monoisotopic
10	322.00	3453	1.15			
11	360.00	17128	5.69			
12	360.90	3156	1.05			
13	361.95	10308	3.43			
14	615.05	9214	3.06			
15	616.05	4246	1.41			
16	617.00	16108	5.36			
17	618.00	4738	1.58			
18	619.05	6994	2.33			

Figure S123 LCMS spectrum of compound NPD-2992

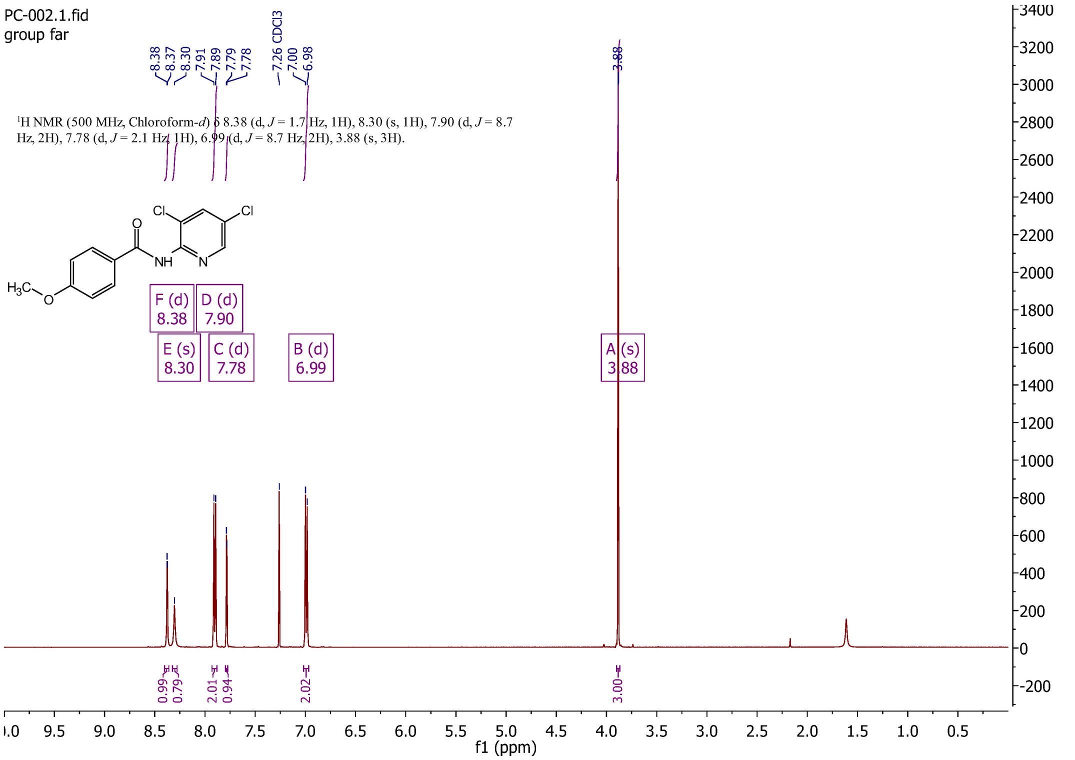

Figure S124 ^{1}H NMR spectrum of compound NPD-2992

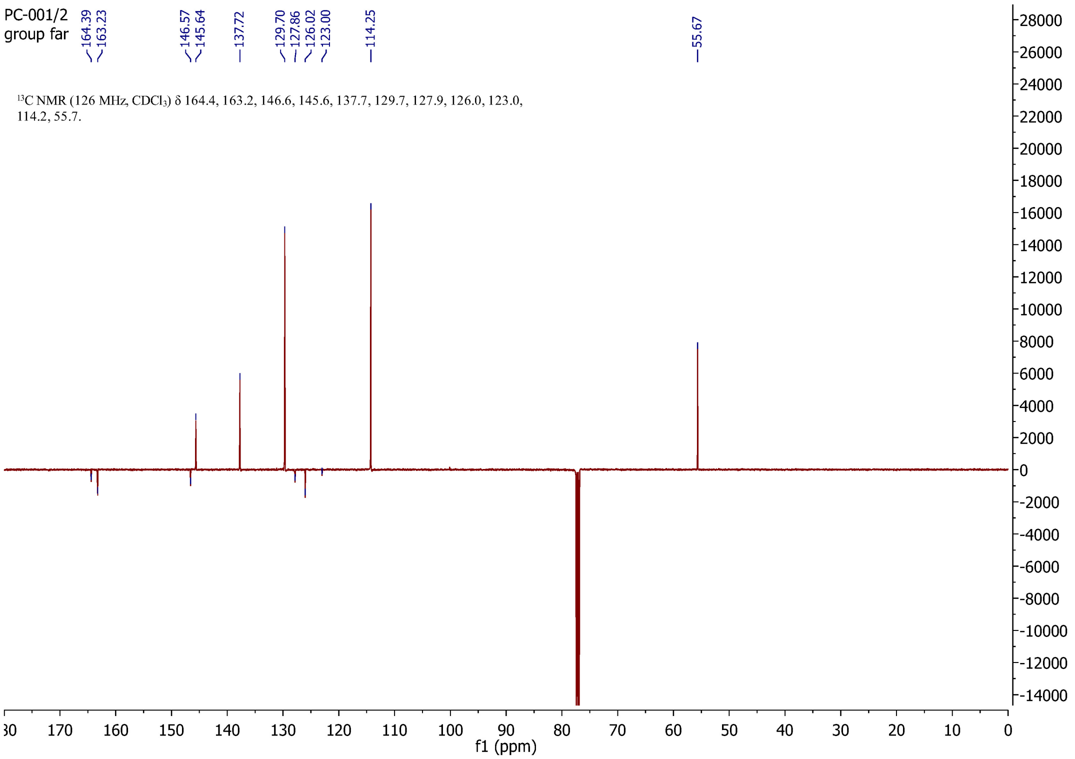

Figure S125 ^{13}C NMR spectrum of compound NPD-2992

Acquired by : Admin
Date Acquired : 2/22/2017 12:37:37 PM
Sample Name : Practical Course-121-1
Sample ID :
Tray# : 1
Vial# : 37
Injection Volume : 1
Data File : C:\LabSolutions\Data\2017\2017-wk08\Practical Course-121-1.lcd
Background File : blanco 22022017.lcd
Method File : Method SCAN ACID standard.lcm
Report Format : DefaultLCMS.lcr
Tuning File : C:\LabSolutions\Tuning\Tuning-ESI-pos-neg01072015a.lct
Processed by : Admin
Modified Date : 2/22/2017 1:42:04 PM

PDA Graph

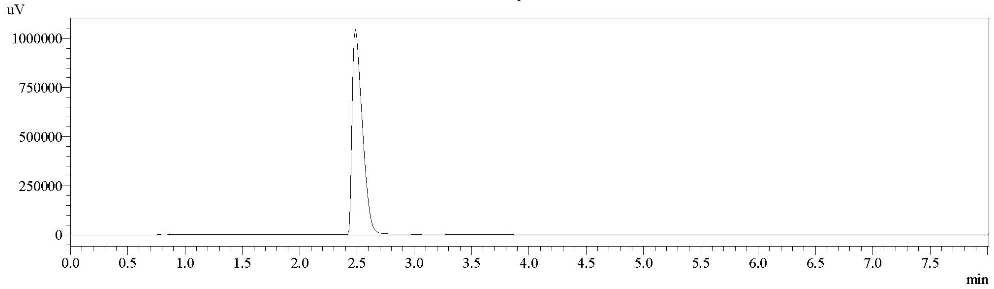

PeakTable

PDA Ch1 254nm 4nm

Peak#	Name	Ret. Time	Area	Area %
1		2.481	6369576	100.000

MS Chromatogram
Practical Course-121-1 C:\LabSolutions\Data\2017\2017-wk08\Practical Course-121-1.lcd

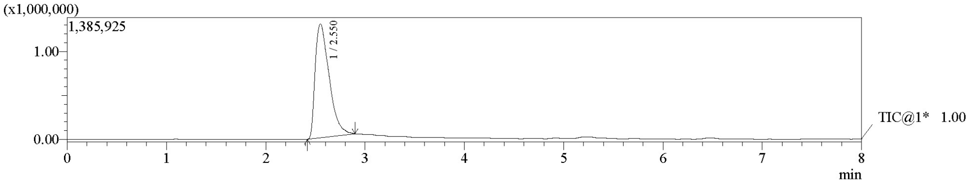

MS Spectrum Graph

#:1 Ret.Time:Averaged 2.540-2.560(Scan#:255-257)
Mass Peaks:3 Base Peak:243.10(1122590) Polarity:Pos Segment1 - Event1

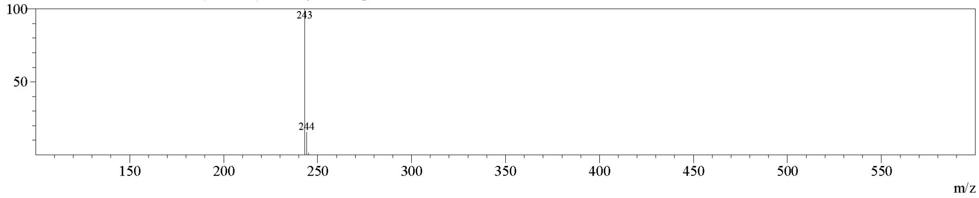

MS Spectrum Table

#:1 Ret.Time:
BG Mode:Calc 2.420<->2.900(243<->291)
Mass Peaks:3 Base Peak:243.10(1122590) Polarity:Pos Segment1 - Event1

#	m/z	Abs.Inten.	Rel.Inten.	Charge	Polarity	Monoisotopic	#	m/z	Abs.Inten.	Rel.Inten.	Charge	Polarity	Monoisotopic
1	243.10	1122590	100.00				3	245.10	14910	1.33			
2	244.10	172408	15.36										

Figure S126 LCMS spectrum of compound NPD-2993

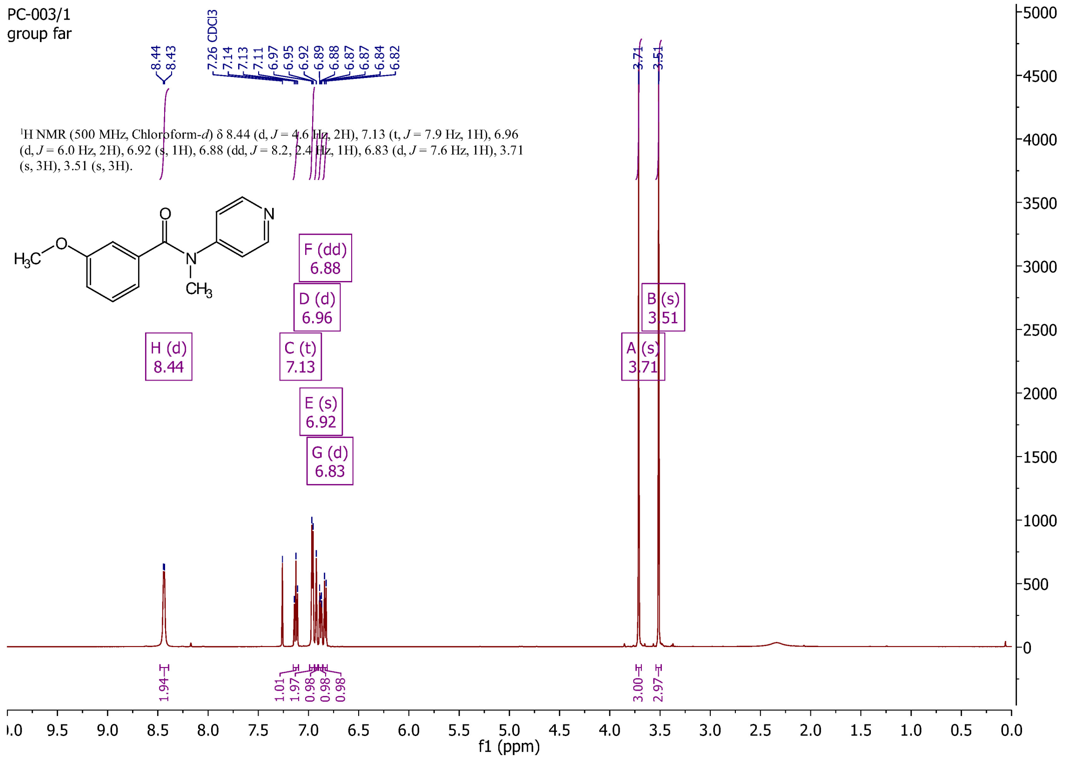

Figure S127 ^{1}H NMR spectrum of compound NPD-2993

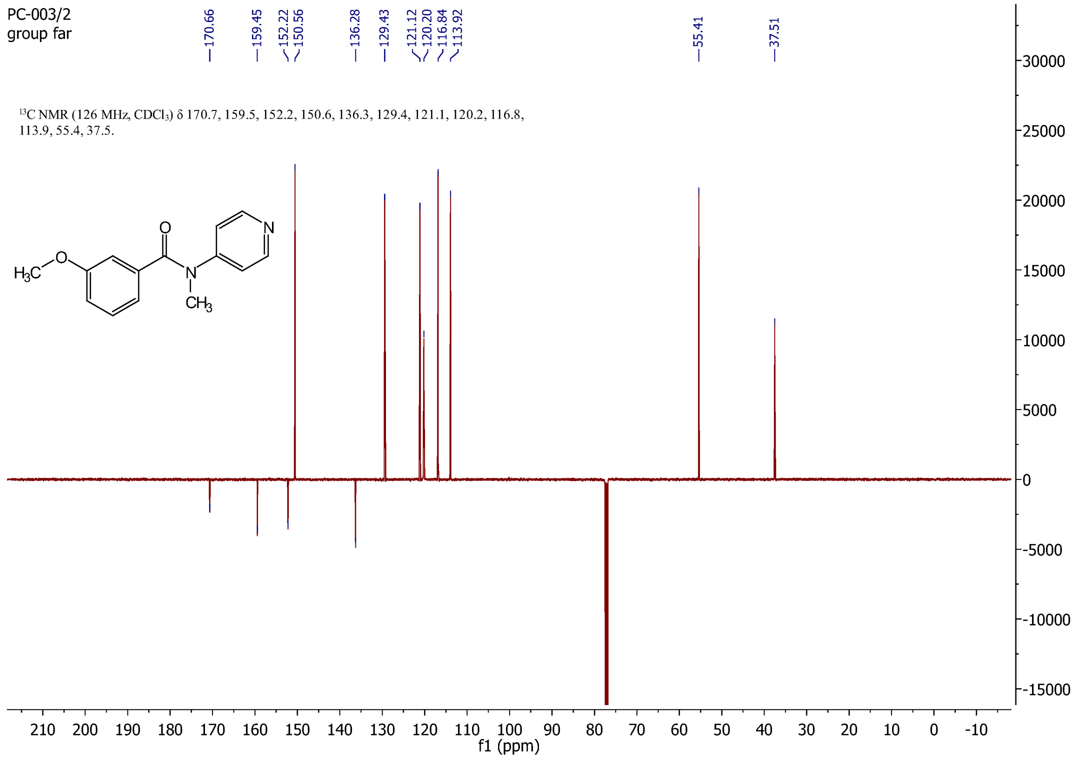

Figure S128 ^{13}C NMR spectrum of compound NPD-2993

Acquired by	: Admin
Date Acquired	: 2/22/2017 10:43:59 AM
Sample Name	: Practical Course-123
Sample ID	:
Tray#	: 1
Vial#	: 27
Injection Volume	: 1
Data File	: C:\LabSolutions\Data\2017\2017-wk08\Practical Course-123.lcd
Background File	: blanco 22022017.lcd
Method File	: Method SCAN ACID standard.lcm
Report Format	: DefaultLCMS.lcr
Tuning File	: C:\LabSolutions\Tuning\Tuning-ESI-pos-neg01072015a.lct
Processed by	: Admin
Modified Date	: 2/22/2017 11:41:14 AM

PDA Graph

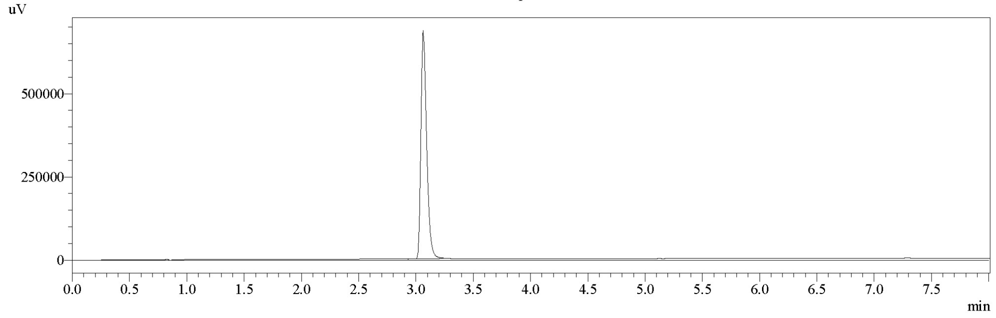

PDA Ch1 254nm 4nm

Peak#	Name	Ret. Time	Area	Area %
1		3.059	2366839	100.000

MS Spectrum Graph

#:1 Ret.Time:Averaged 3.110-3.130(Scan#:312-314)
Mass Peaks:6 Base Peak:230.05(380325) Polarity:Pos Segment1 - Event1

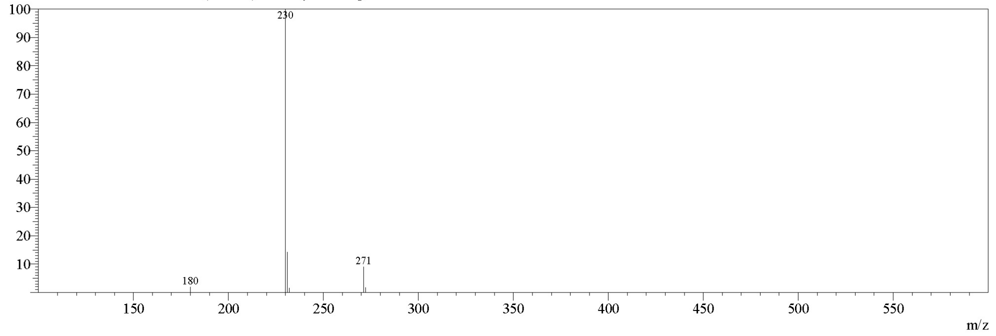

MS Spectrum Table

#:1 Ret.Time:
BG Mode:Calc 3.010<->3.190(302<->320)
Mass Peaks:6 Base Peak:230.05(380325) Polarity:Pos Segment1 - Event1

#	m/z	Abs.Inten.	Rel.Inten.	Charge	Polarity	Monoisotopic	#	m/z	Abs.Inten.	Rel.Inten.	Charge	Polarity	Monoisotopic
1	180.00	7033	1.85				4	232.00	5927	1.56			
2	230.05	380325	100.00				5	271.10	34608	9.10			
3	231.05	53980	14.19				6	272.10	6395	1.68			

Figure S129 LCMS spectrum of compound NPD-2994

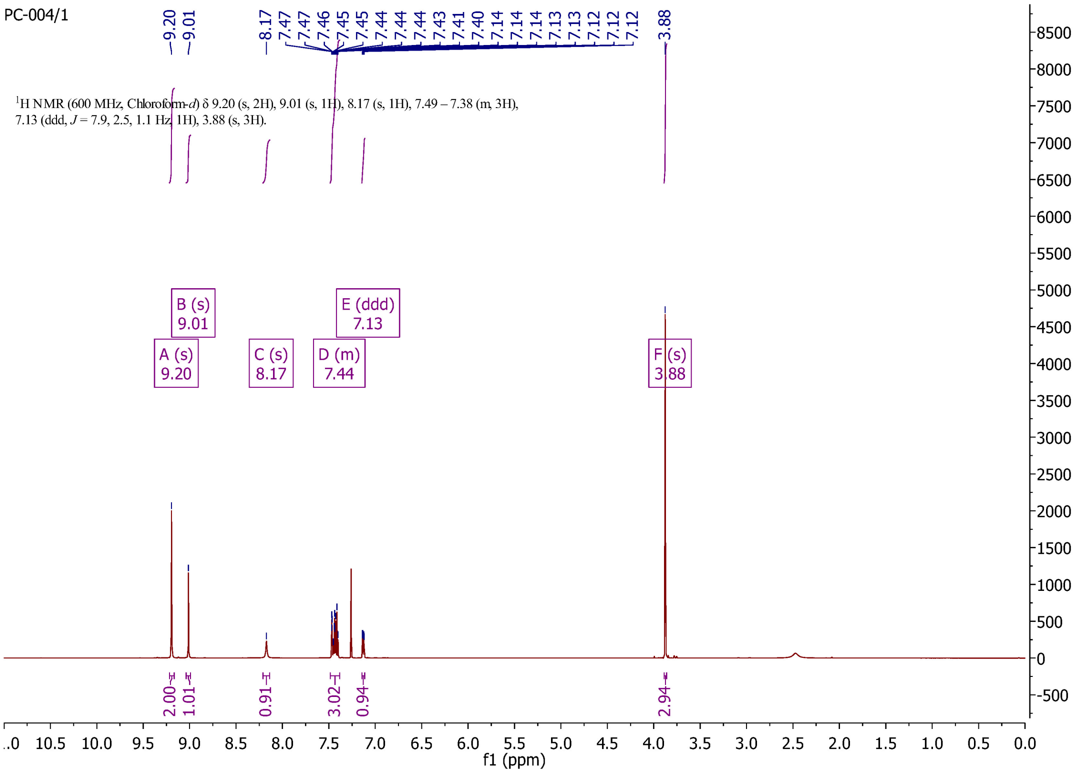

Figure S130 ^{1}H NMR spectrum of compound NPD-2994

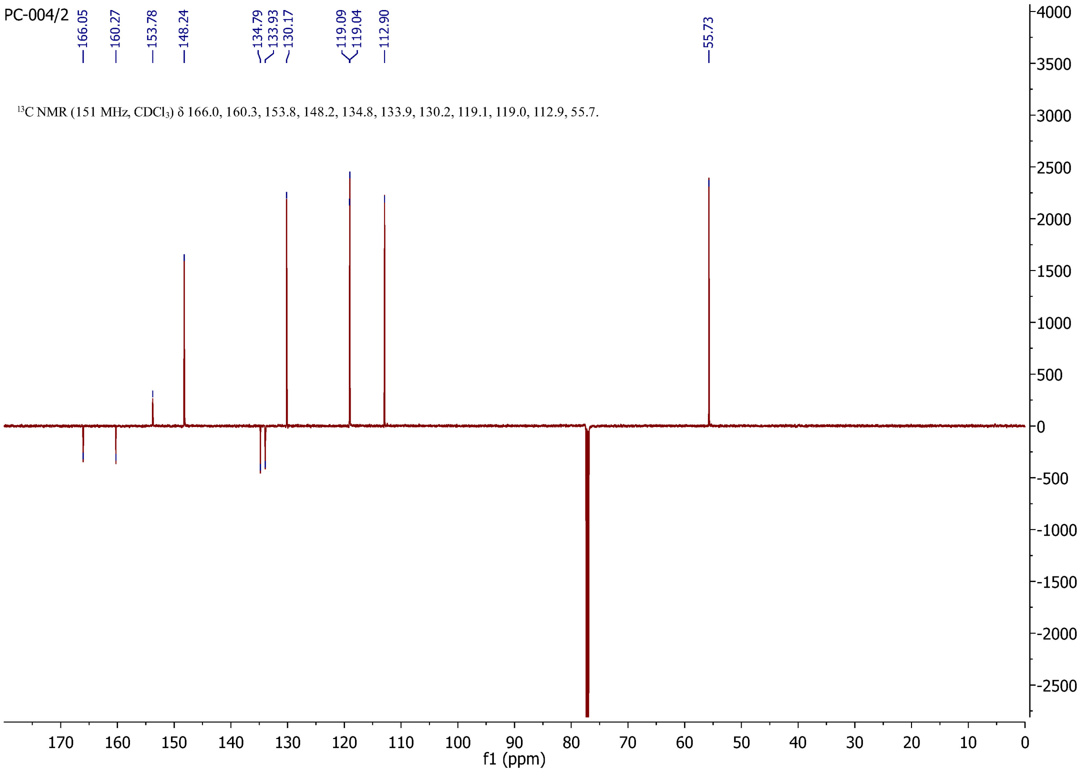

Figure S131 ^{13}C NMR spectrum of compound NPD-2994

Acquired by	: Admin
Date Acquired	: 2/22/2017 11:01:15 AM
Sample Name	: PC-1289
Sample ID	:
Tray#	: 1
Vial#	: 28
Injection Volume	: 1
Data File	: C:\LabSolutions\Data\2017\2017-wk08\PC-129.lcd
Background File	: blanco 22022017.lcd
Method File	: Method SCAN ACID standard.lcm
Report Format	: DefaultLCMS.lcr
Tuning File	: C:\LabSolutions\Tuning\Tuning-ESI-pos-neg01072015a.lct
Processed by	: Admin
Modified Date	: 2/22/2017 11:34:19 AM

PDA Graph

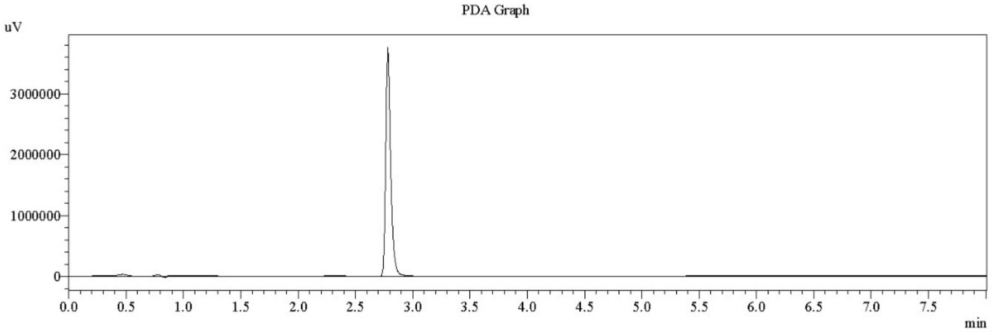

PDA Ch1 230nm 4nm

Peak#	Name	Ret. Time	Area	Area%
1		2.778	11122396	100.000

MS Spectrum Graph

#:1 Ret.Time:Averaged 2.830-2.850(Scan#:284-286)
Mass Peaks:5 Base Peak:274.10(981528) Polarity:Pos Segment1 - Event1

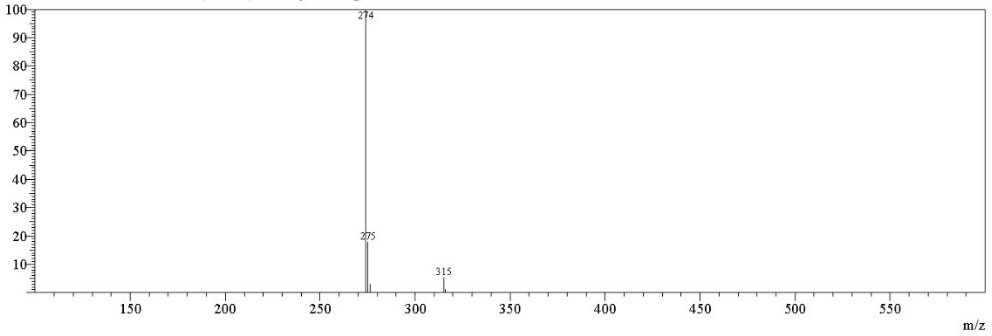

MS Spectrum Table

#:1 Ret.Time:
BG Mode:Calc 2.730<->3.100(274<->311)
Mass Peaks:5 Base Peak:274.10(981528) Polarity:Pos Segment1 - Event1

#	m/z	Abs.Inten.	Rel.Inten.	Charge	Polarity	Monoisotopic
1	274.10	981528	100.00			
2	275.15	173963	17.72			
3	276.10	28897	2.94			
4	315.10	50688	5.16			
5	316.10	11287	1.15			

Figure S132 LCMS spectrum of compound NPD-2995

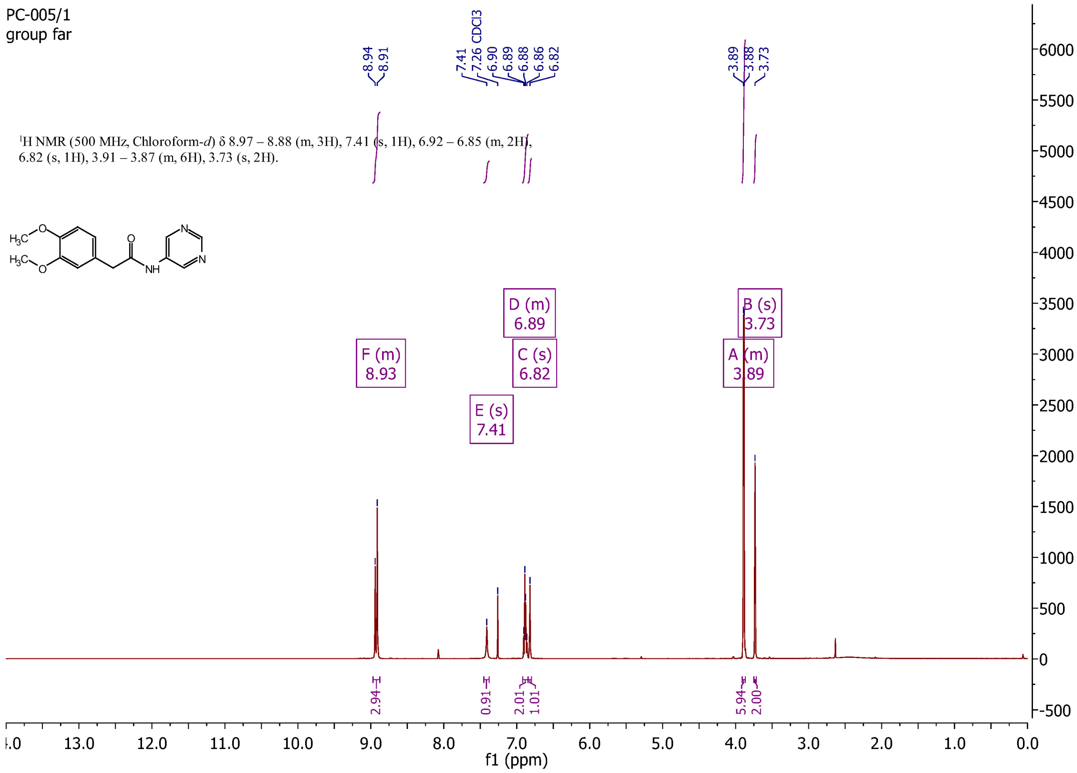

Figure S133 ^{1}H NMR spectrum of compound NPD-2995

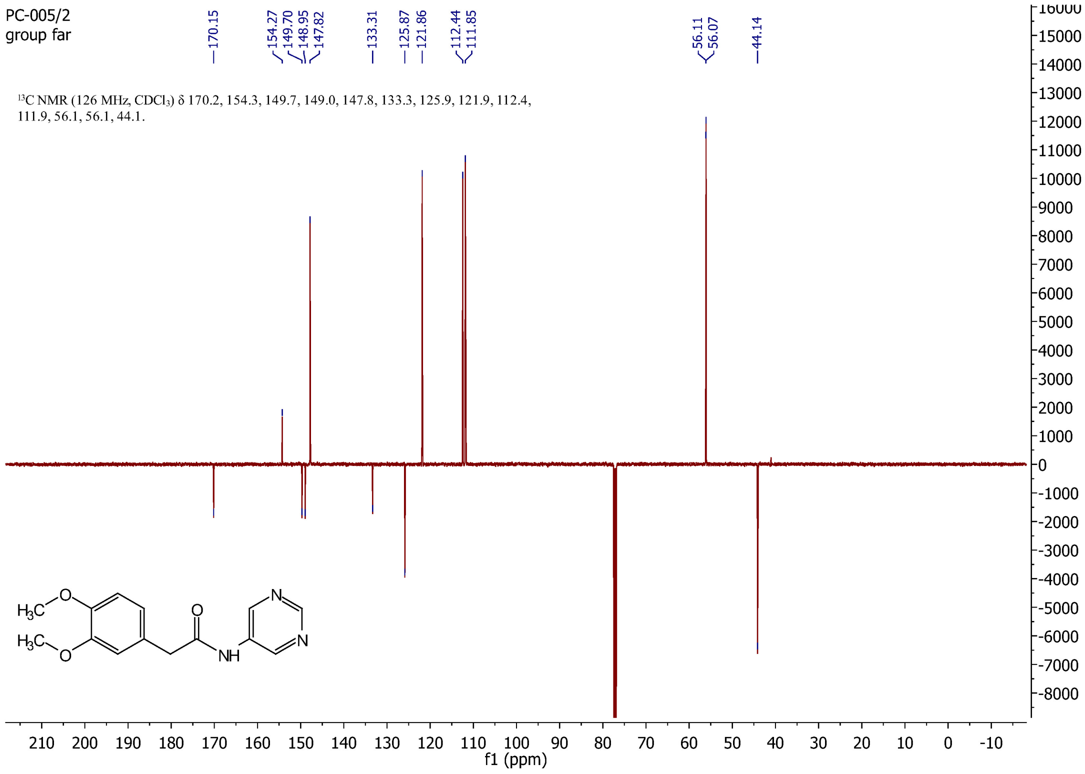

Figure S134 ^{13}C NMR spectrum of compound NPD-2995

Acquired by	: Admin
Date Acquired	: 2/22/2017 11:09:52 AM
Sample Name	: PC-131
Sample ID	:
Tray#	: 1
Vial#	: 29
Injection Volume	: 1
Data File	: C:\LabSolutions\Data\2017\2017-wk08\PC-131.lcd
Background File	: blanco 22022017.lcd
Method File	: Method SCAN ACID standard.lcm
Report Format	: DefaultLCMS.lcr
Tuning File	: C:\LabSolutions\Tuning\Tuning-ESI-pos-neg01072015a.lct
Processed by	: Admin
Modified Date	: 2/22/2017 11:38:30 AM

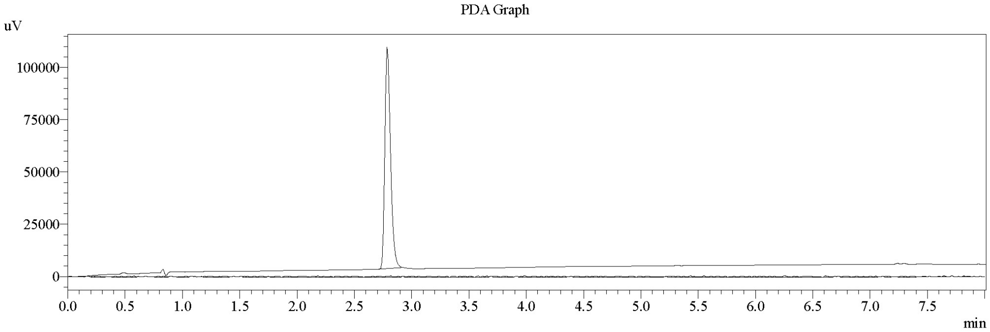

PDA Ch1 254nm 4nm

Peak#	Name	Ret. Time	Area	Area %
1		2.782	364495	100.000

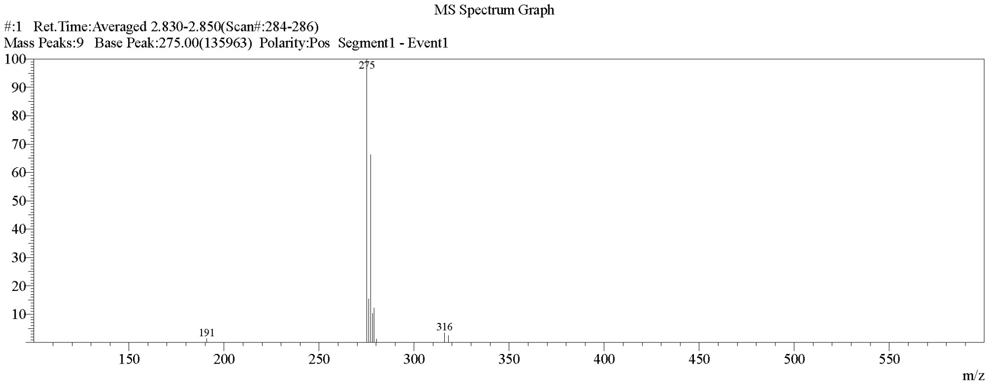

MS Spectrum Table

#:1 Ret.Time:
BG Mode:Calc 2.730<->3.000(274<->301)
Mass Peaks:9 Base Peak:275.00(135963) Polarity:Pos Segment1 - Event1

#	m/z	Abs.Inten.	Rel.Inten.	Charge	Polarity	Monoisotopic
1	190.95	1918	1.41			
2	275.00	135963	100.00			
3	276.00	20916	15.38			
4	277.00	90163	66.31			
5	278.05	13972	10.28			
6	279.00	16514	12.15			
7	280.15	1778	1.31			
8	316.10	4588	3.37			
9	318.10	3465	2.55			

Figure S135 LCMS spectrum of compound NPD-2996

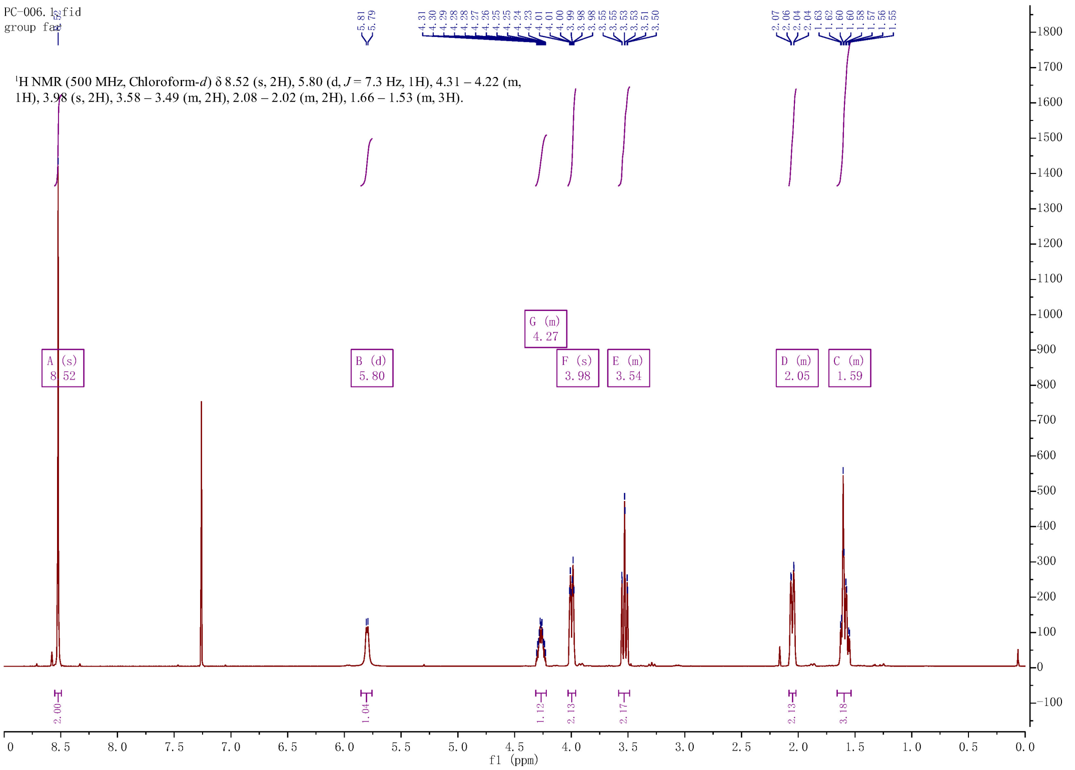

Figure S136 ^{1}H NMR spectrum of compound NPD-2996

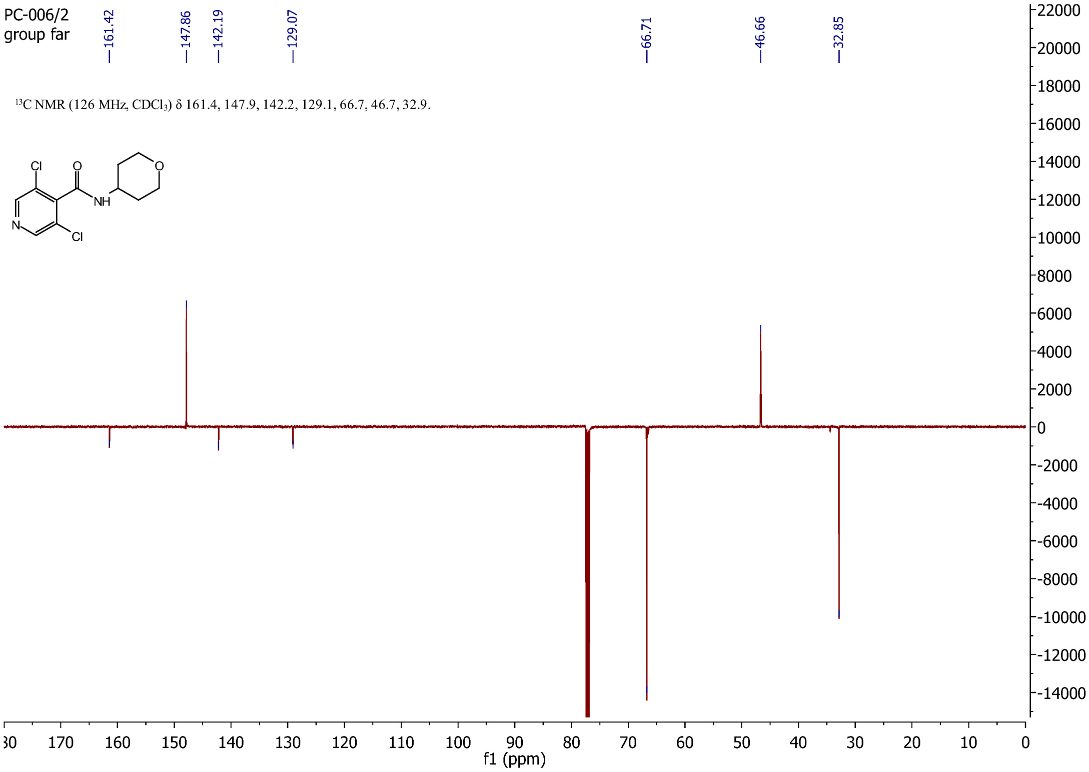

Figure S137 ^{13}C NMR spectrum of compound NPD-2996

Acquired by	: Admin
Date Acquired	: 2/22/2017 12:11:00 PM
Sample Name	: PC-134
Sample ID	:
Tray#	: 1
Vial#	: 35
Injection Volume	: 1
Data File	: C:\LabSolutions\Data\2017\2017-wk08\PC-134.lcd
Background File	: blanco 22022017.lcd
Method File	: Method SCAN ACID standard.lcm
Report Format	: DefaultLCMS.lcr
Tuning File	: C:\LabSolutions\Tuning\Tuning-ESI-pos-neg01072015a.lct
Processed by	: Admin
Modified Date	: 2/22/2017 1:42:58 PM

PDA Graph

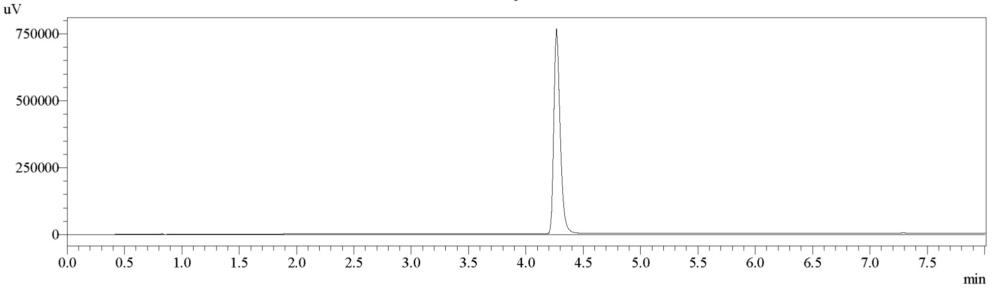

PeakTable

PDA Ch1 254nm 4nm

Peak#	Name	Ret. Time	Area	Area %
1		4.262	2880432	100.000

MS Chromatogram
PC-134 C:\LabSolutions\Data\2017\2017-wk08\PC-134.lcd

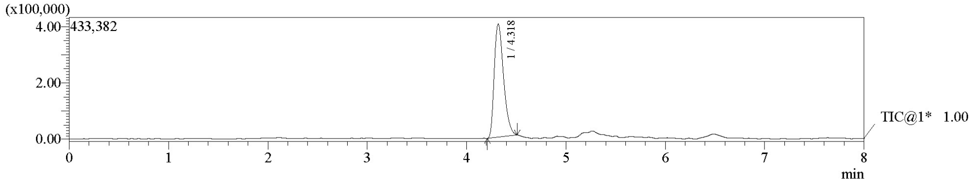

MS Spectrum Graph

#:1 Ret.Time:Averaged 4.310-4.330(Scan#:432-434)
Mass Peaks:15 Base Peak:330.95(137025) Polarity:Pos Segment1 - Event1

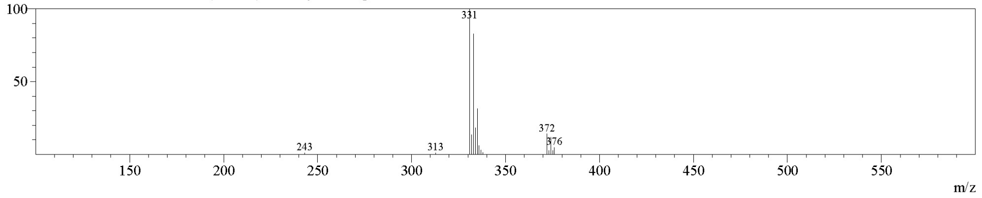

MS Spectrum Table

#:1 Ret.Time:
BG Mode:Calc 4.210<->4.510(422<->452)
Mass Peaks:15 Base Peak:330.95(137025) Polarity:Pos Segment1 - Event1

#	m/z	Abs.Inten.	Rel.Inten.	Charge	Polarity	Monoisotopic
1	243.10	1560	1.14			
2	312.80	1561	1.14			
3	330.95	137025	100.00			
4	332.00	18575	13.56			
5	332.95	113425	82.78			
6	334.00	25041	18.27			
7	335.00	42988	31.37			
8	335.90	8190	5.98			
9	336.90	4317	3.15			
10	337.90	1887	1.38			
11	372.00	19257	14.05			
12	373.00	3712	2.71			
13	374.00	16322	11.91			
14	375.00	3773	2.75			
15	376.00	6662	4.86			

Figure S138 LCMS spectrum of compound NPD-2997

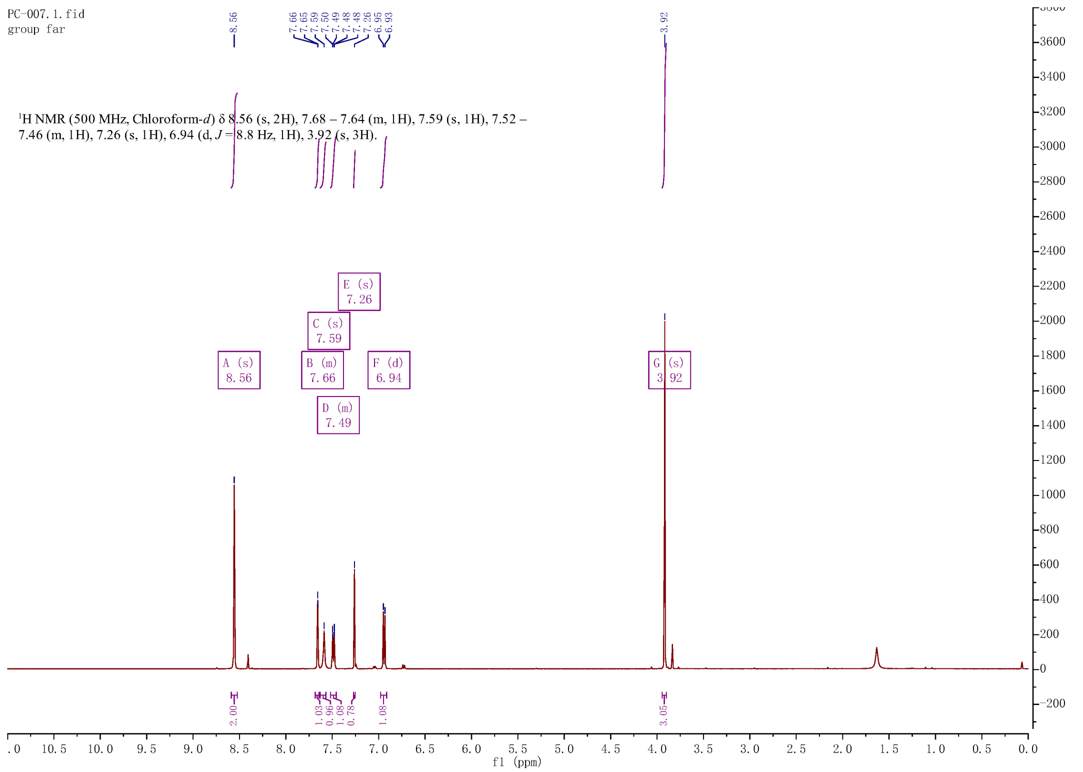

Figure S139 ^{1}H NMR spectrum of compound NPD-2997

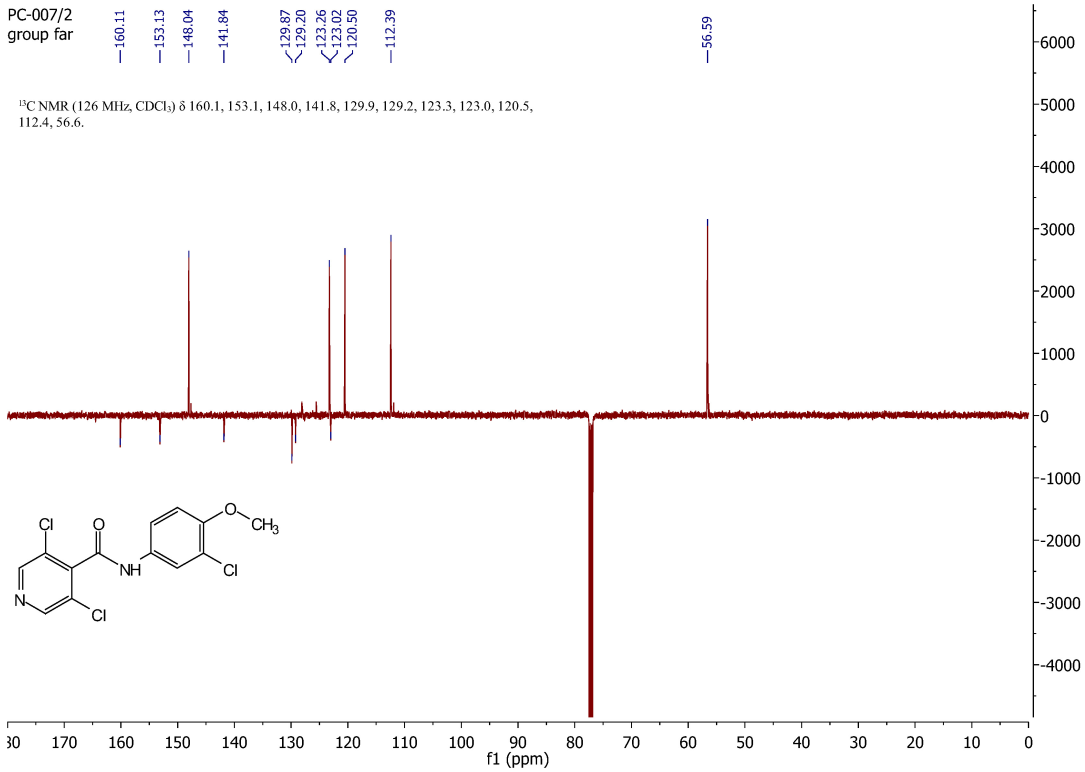

Figure S140 ^{13}C NMR spectrum of compound NPD-2997

Acquired by	: Admin
Date Acquired	: 2/23/2017 10:02:01 AM
Sample Name	: PC-136
Sample ID	:
Tray#	: 1
Vial#	: 20
Injection Volume	: 1
Data File	: C:\LabSolutions\Data\2017\2017-wk08\PC-136.lcd
Background File	: blanco 23022017.lcd
Method File	: Method SCAN ACID standard.lcm
Report Format	: DefaultLCMS.lcr
Tuning File	: C:\LabSolutions\Tuning\Tuning-ESI-pos-neg01072015a.lct
Processed by	: Admin
Modified Date	: 2/23/2017 10:43:37 AM

PDA Graph

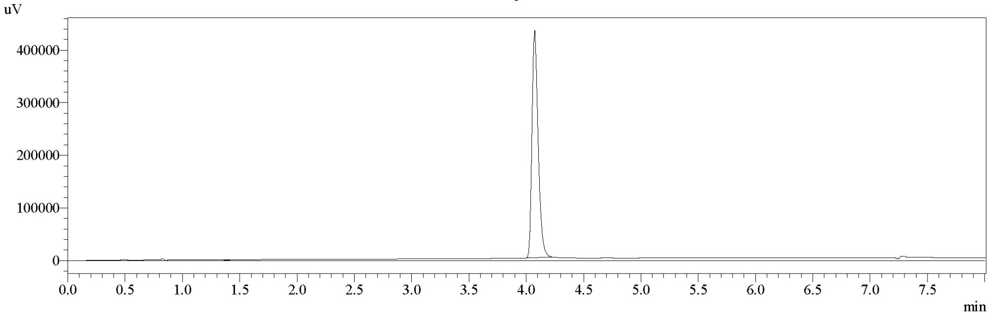

PDA Ch1 254nm 4nm

Peak#	Name	Ret. Time	Area	Area %
1		4.067	1602139	100.000

MS Spectrum Graph

#:1 Ret.Time:Averaged 4.120-4.140(Scan#:413-415)
Mass Peaks:14 Base Peak:337.90(199602) Polarity:Pos Segment1 - Event1

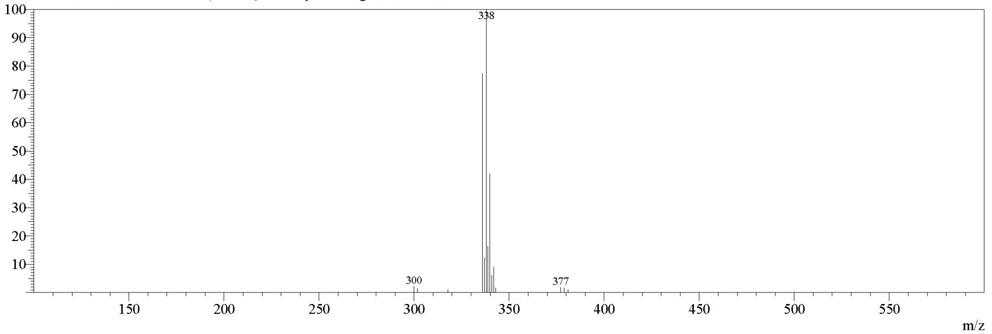

MS Spectrum Table

#:1 Ret.Time:
BG Mode:Calc 4.020<->4.320(403<->433)
Mass Peaks:14 Base Peak:337.90(199602) Polarity:Pos Segment1 - Event1

#	m/z	Abs.Inten.	Rel.Inten.	Charge	Polarity	Monoisotopic
1	299.85	4378	2.19			
2	301.80	3054	1.53			
3	317.85	2056	1.03			
4	335.90	154624	77.47			
5	336.90	24398	12.22			
6	337.90	199602	100.00			
7	338.90	32867	16.47			
8	339.85	83797	41.98			
9	340.85	12125	6.07			
10	341.90	17832	8.93			
11	342.95	3371	1.69			
12	377.00	3659	1.83			
13	378.80	3293	1.65			
14	380.95	2116	1.06			

Figure S141 LCMS spectrum of compound NPD-2998

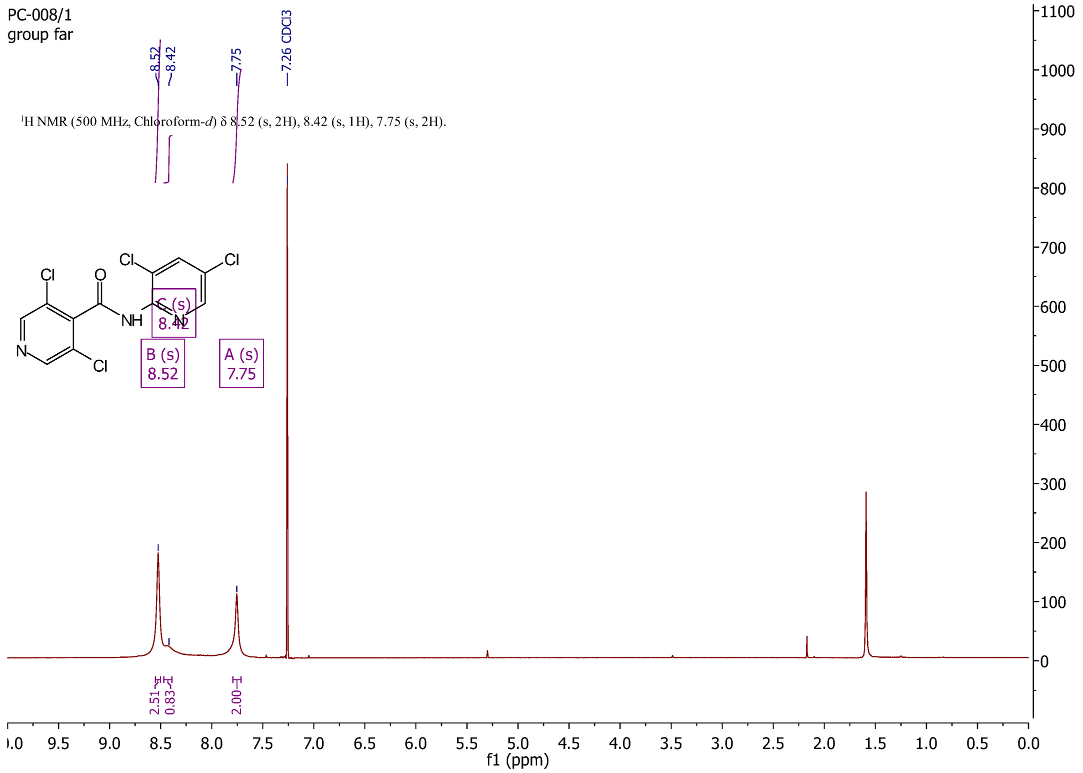

Figure S142 ^{1}H NMR spectrum of compound NPD-2998

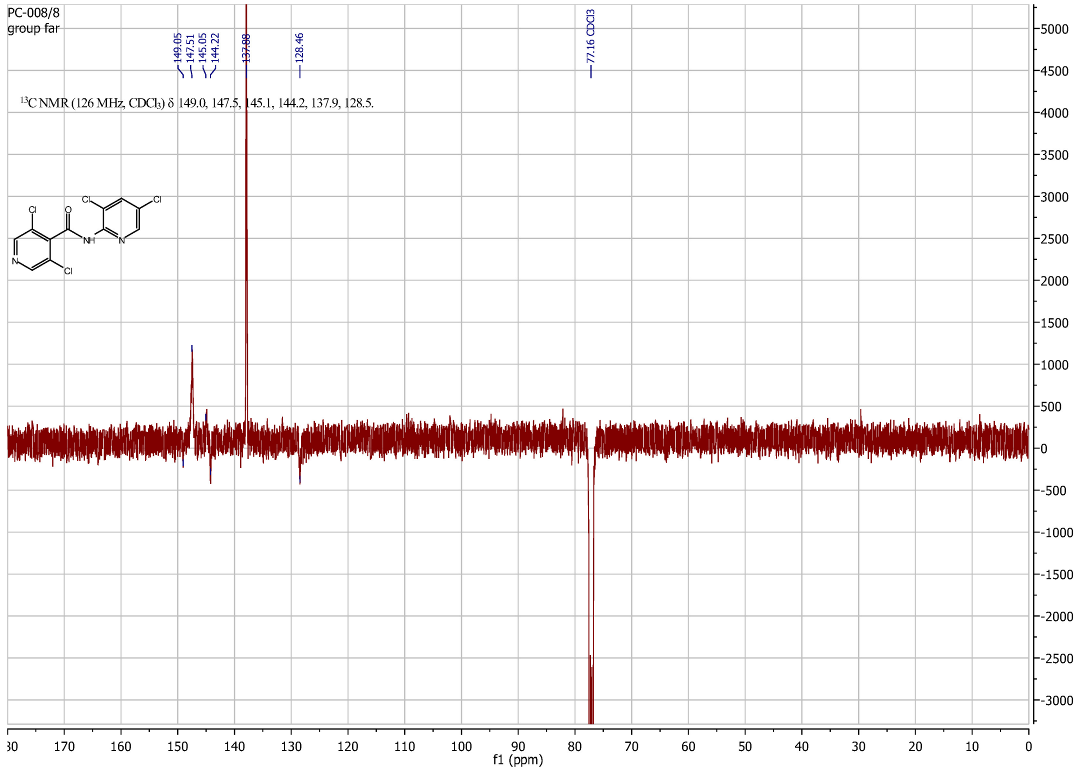

Figure S143 ^{13}C NMR spectrum of compound NPD-2998

Acquired by : Admin
Date Acquired : 2/23/2017 10:10:37 AM
Sample Name : PC-145
Sample ID :
Tray# : 1
Vial# : 21
Injection Volume : 1
Data File : C:\LabSolutions\Data\2017\2017-wk08\PC-145.lcd
Background File : blanco 23022017.lcd
Method File : Method SCAN ACID standard.lcm
Report Format : DefaultLCMS.lcr
Tuning File : C:\LabSolutions\Tuning\Tuning-ESI-pos-neg01072015a.lct
Processed by : Admin
Modified Date : 2/23/2017 11:59:46 AM

PDA Graph

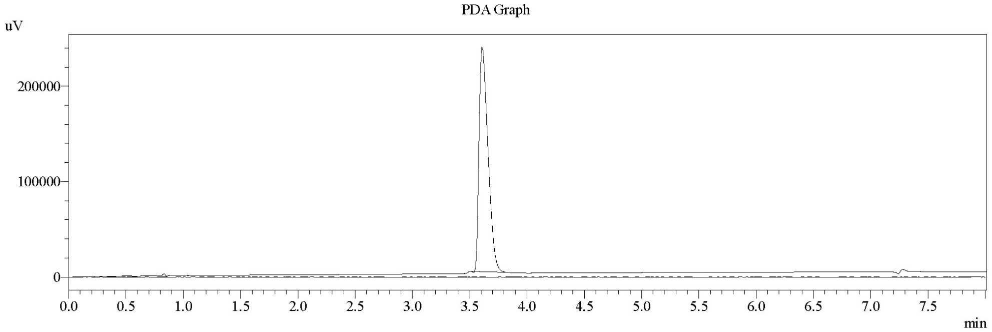

PDA Ch1 254nm 4nm

Peak#	Name	Ret. Time	Area	Area %
1		3.604	1268254	100.000

MS Spectrum Graph

#:1 Ret.Time:Averaged 3.650-3.670(Scan#:366-368)
Mass Peaks:4 Base Peak:349.10(2022664) Polarity:Pos Segment1 - Event1

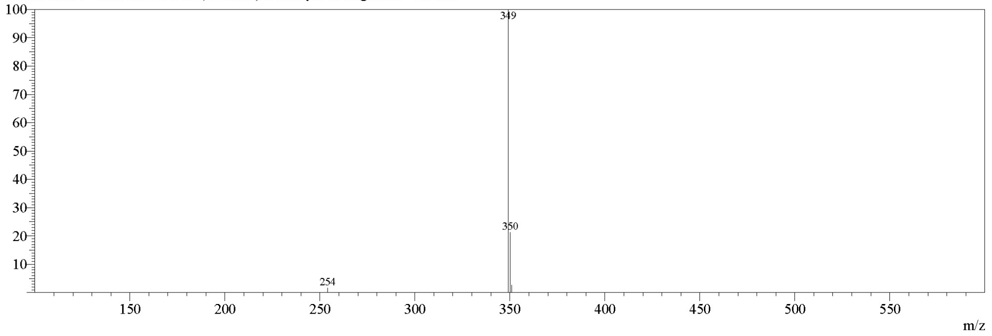

MS Spectrum Table

#:1 Ret.Time:
BG Mode:Calc 3.520<->3.980(353<->399)
Mass Peaks:4 Base Peak:349.10(2022664) Polarity:Pos Segment1 - Event1

#	m/z	Abs.Inten.	Rel.Inten.	Charge	Polarity	Monoisotopic
1	254.05	34110	1.69			
2	349.10	2022664	100.00			
3	350.10	431483	21.33			
4	351.10	55256	2.73			

Figure S144 LCMS spectrum of compound NPD-2999

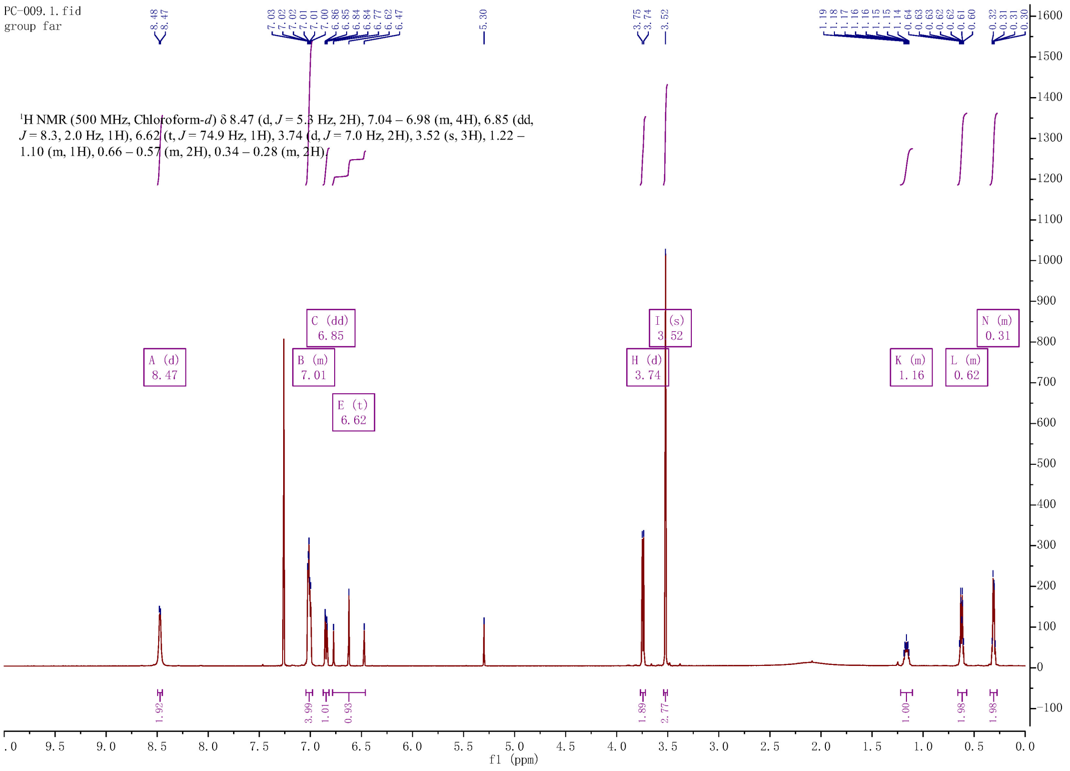

Figure S145 ^{1}H NMR spectrum of compound NPD-2999

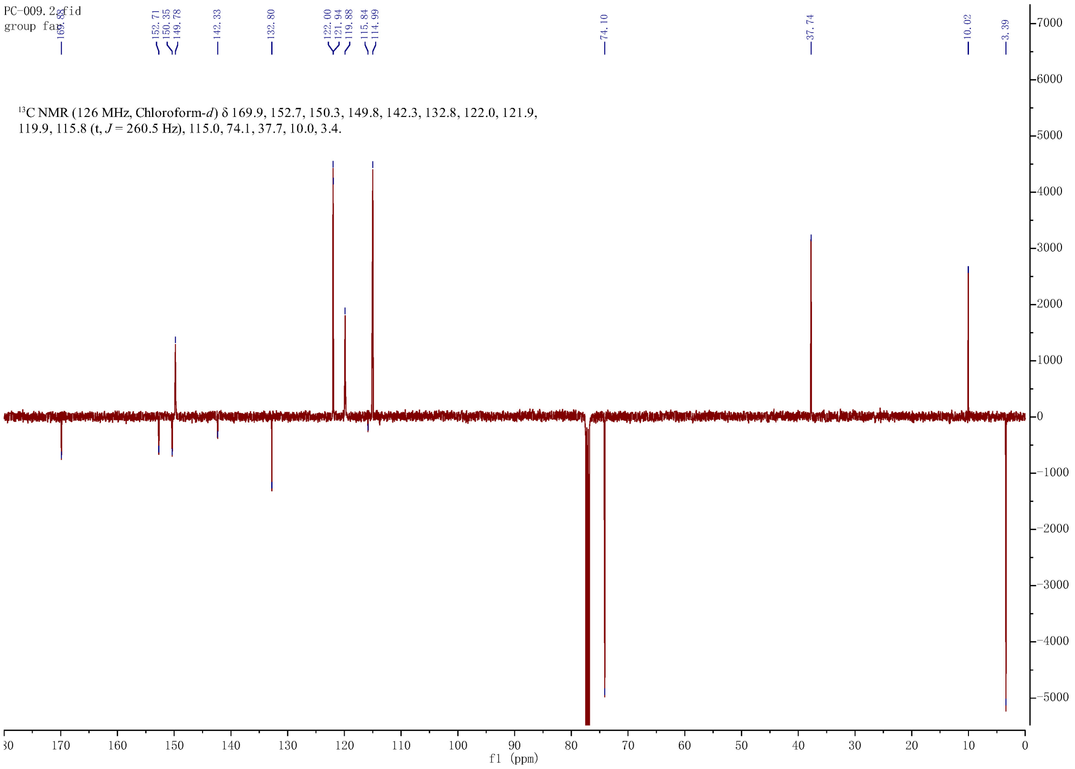

Figure S146 ^{13}C NMR spectrum of compound NPD-2999

Acquired by	: Admin
Date Acquired	: 2/23/2017 10:19:13 AM
Sample Name	: PC-146
Sample ID	:
Tray#	: 1
Vial#	: 22
Injection Volume	: 1
Data File	: C:\LabSolutions\Data\2017\2017-wk08\PC-146.lcd
Background File	: blanco 23022017.lcd
Method File	: Method SCAN ACID standard.lcm
Report Format	: DefaultLCMS.lcr
Tuning File	: C:\LabSolutions\Tuning\Tuning-ESI-pos-neg01072015a.lct
Processed by	: Admin
Modified Date	: 2/23/2017 12:00:31 PM

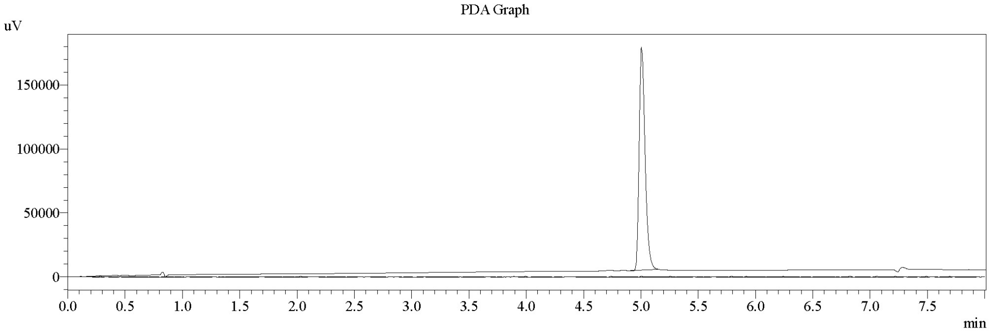

PDA Ch1 254nm 4nm

Peak#	Name	Ret. Time	Area	Area %
1		5.001	628557	100.000

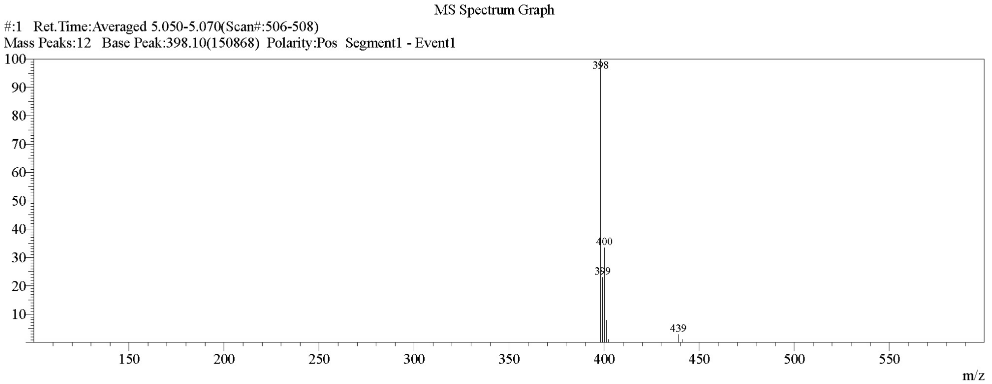

MS Spectrum Table

#:1 Ret.Time:
BG Mode:Calc 4.950<->5.250(496<->526)
Mass Peaks:12 Base Peak:398.10(150868) Polarity:Pos Segment1 - Event1

#	m/z	Abs.Inten.	Rel.Inten.	Charge	Polarity	Monoisotopic
1	398.10	150868	100.00			
2	399.10	34813	23.08			
3	400.10	50380	33.39			
4	401.10	11841	7.85			
5	402.10	1575	1.04			
6	439.15	4307	2.85			
7	441.05	1771	1.17			
8	623.60	3408	2.26			
9	624.70	4133	2.74			
10	625.75	2207	1.46			
11	631.70	1871	1.24			
12	823.40	1534	1.02			

Figure S147 LCMS spectrum of compound NPD-3000

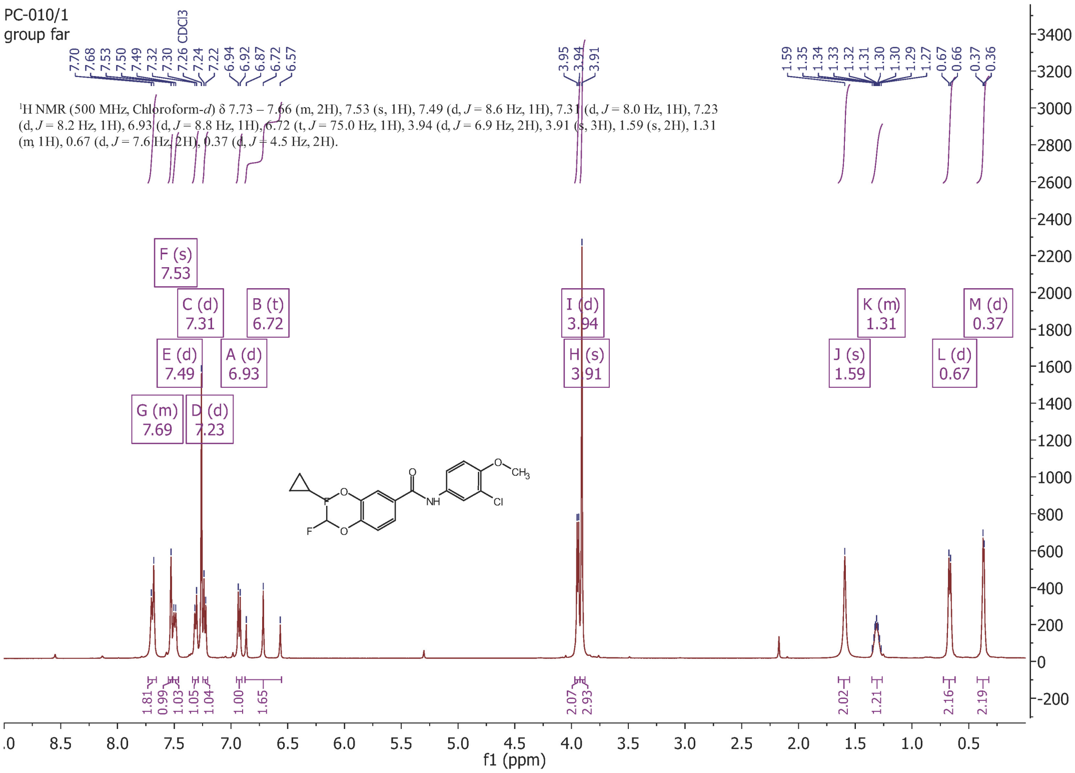

Figure S148 ^{1}H NMR spectrum of compound NPD-3000

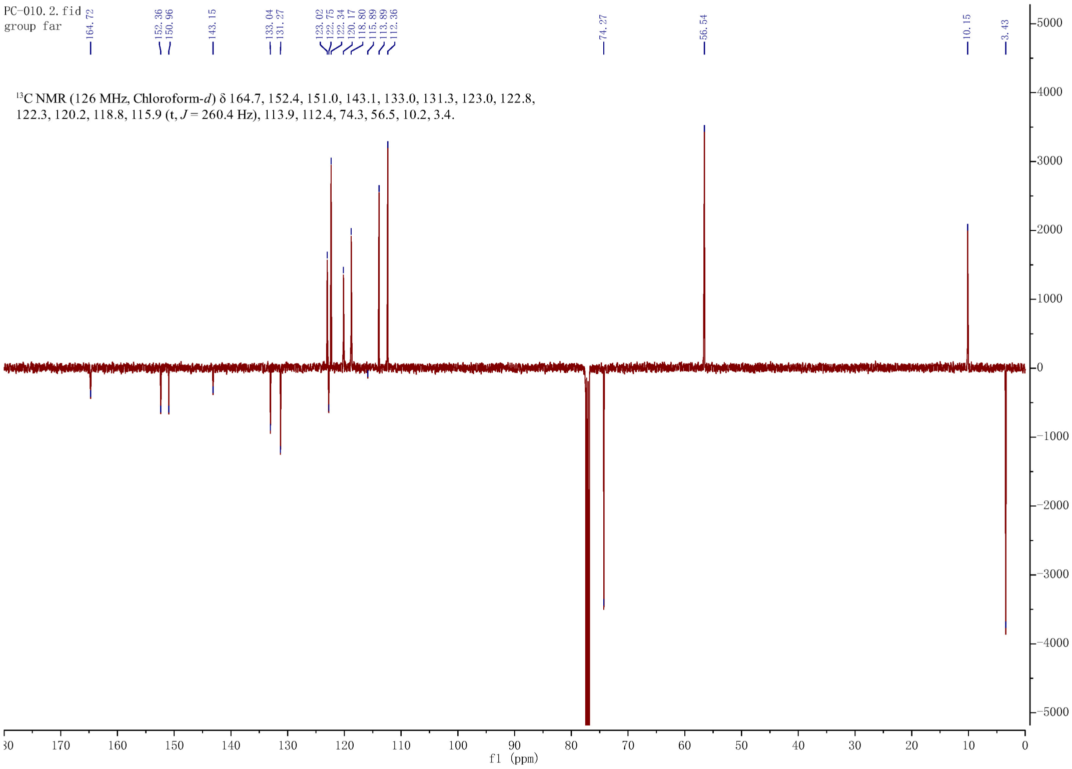

Figure S149 ^{13}C NMR spectrum of compound NPD-3000

Acquired by	: Admin
Date Acquired	: 2/23/2017 10:27:50 AM
Sample Name	: PC-147
Sample ID	:
Tray#	: 1
Vial#	: 23
Injection Volume	: 1
Data File	: C:\LabSolutions\Data\2017\2017-wk08\PC-147.lcd
Background File	: blanco 23022017.lcd
Method File	: Method SCAN ACID standard.lcm
Report Format	: DefaultLCMS.lcr
Tuning File	: C:\LabSolutions\Tuning\Tuning-ESI-pos-neg01072015a.lct
Processed by	: Admin
Modified Date	: 2/23/2017 12:01:22 PM

PDA Graph

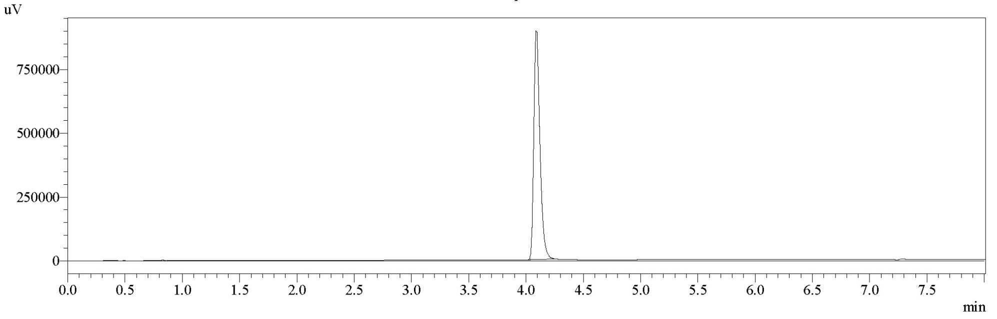

PDA Ch1 254nm 4nm

Peak#	Name	Ret. Time	Area	Area %
1		4.085	3291341	100.000

MS Spectrum Graph

#:1 Ret.Time:Averaged 4.130-4.150(Scan#:414-416)
Mass Peaks:5 Base Peak:336.10(1142073) Polarity:Pos Segment1 - Event1

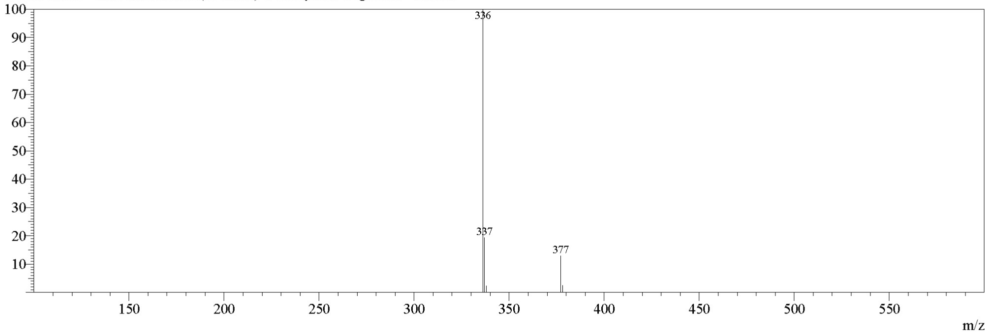

MS Spectrum Table

#:1 Ret.Time:
BG Mode:Calc 4.020<->4.420(403<->443)
Mass Peaks:5 Base Peak:336.10(1142073) Polarity:Pos Segment1 - Event1

#	m/z	Abs.Inten.	Rel.Inten.	Charge	Polarity	Monoisotopic
1	336.10	1142073	100.00			
2	337.10	221193	19.37			
3	338.10	26083	2.28			
4	377.10	147807	12.94			
5	378.10	27991	2.45			

Figure S150 LCMS spectrum of compound NPD-3001

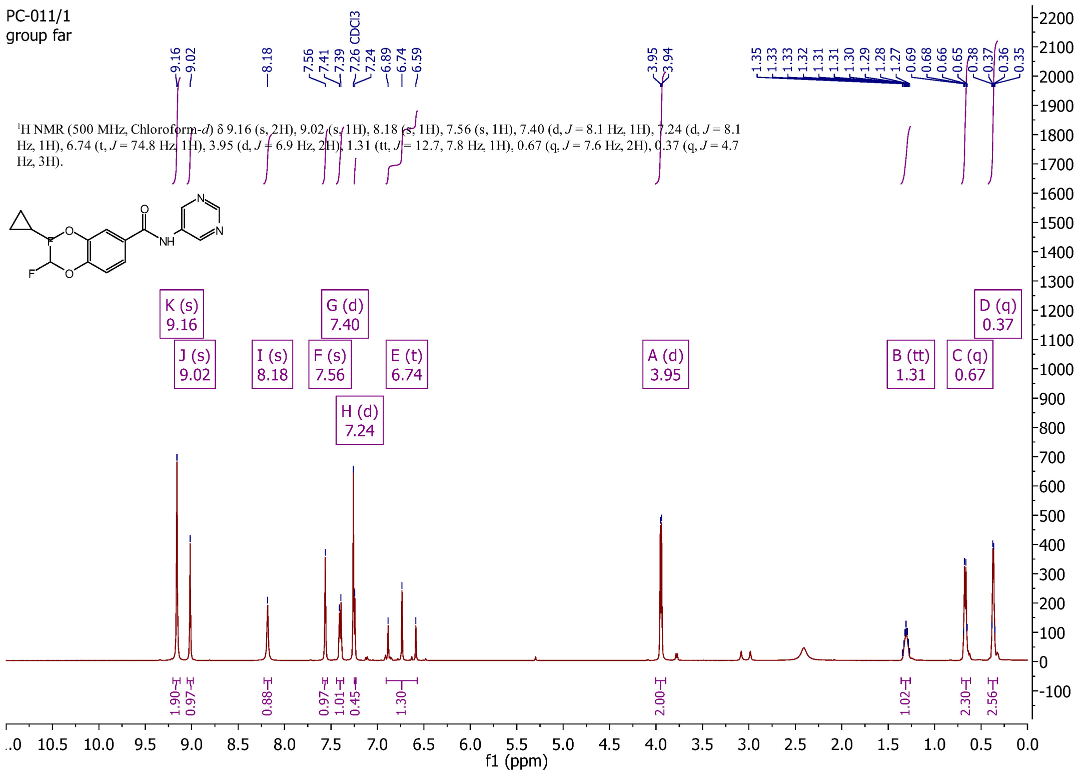

Figure S151 ^{1}H NMR spectrum of compound NPD-3001

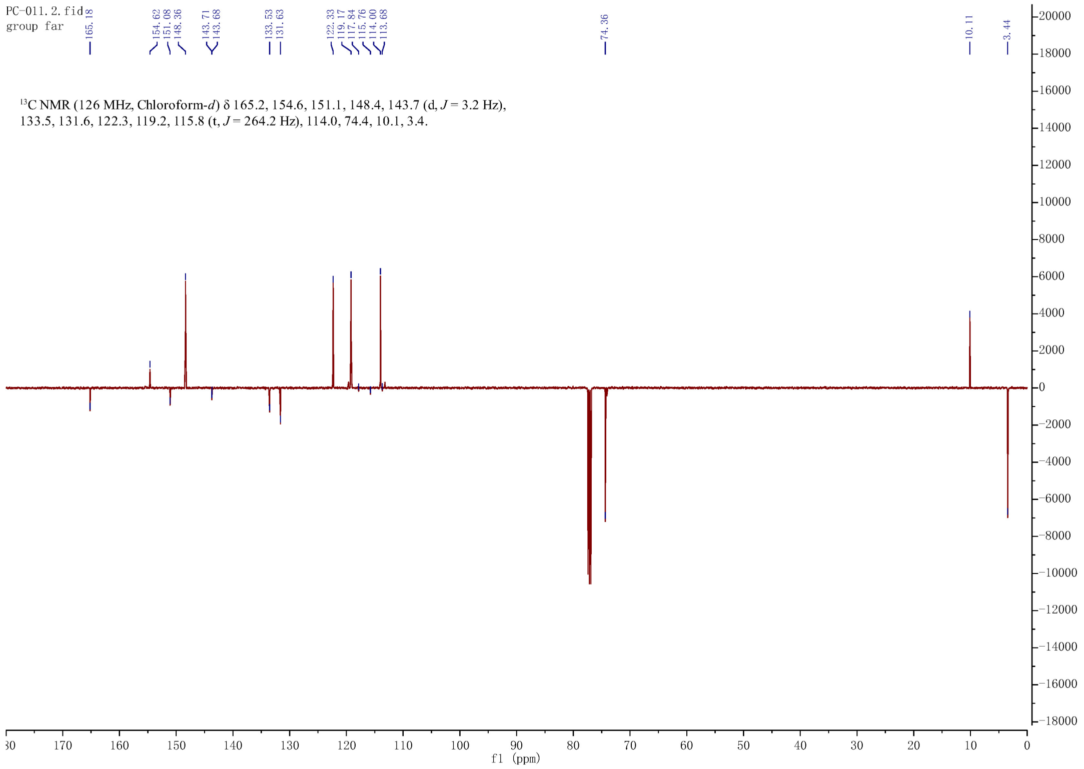

Figure S152 ^{13}C NMR spectrum of compound NPD-3001

Acquired by	: Admin
Date Acquired	: 2/1/2017 5:01:59 PM
Sample Name	: cpd 113 Dominique
Sample ID	:
Tray#	: 1
Vial#	: 11
Injection Volume	: 1
Data File	: C:\LabSolutions\Data\2017\2017-WK05\cpd 113 Dominique.lcd
Background File	: Blanco01022017.lcd
Method File	: Method SCAN ACID standard MW100.lcm
Report Format	: DefaultLCMS.lcr
Tuning File	: C:\LabSolutions\Tuning\Tuning-ESI-pos-neg01072015a.lct
Processed by	: Admin
Modified Date	: 2/20/2017 4:06:21 PM

PDA Graph

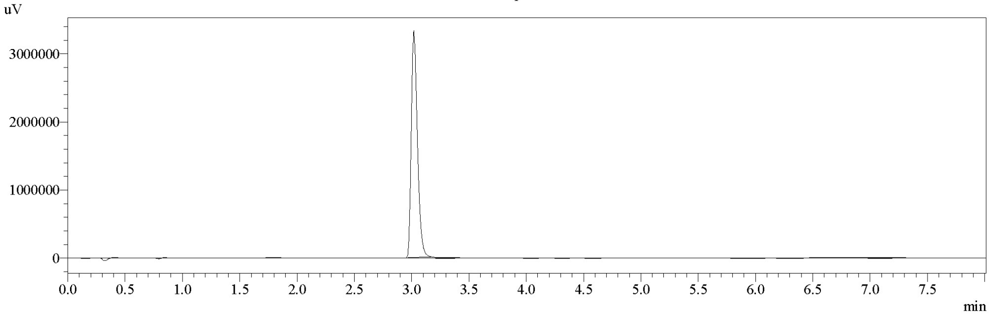

PDA Ch1 254nm 4nm

Peak#	Name	Ret. Time	Area	Area %
1		3.014	12268611	100.000

MS Spectrum Graph

#:1 Ret.Time:Averaged 3.070-3.090(Scan#:308-310)
Mass Peaks:7 Base Peak:236.05(353179) Polarity:Pos Segment1 - Event1

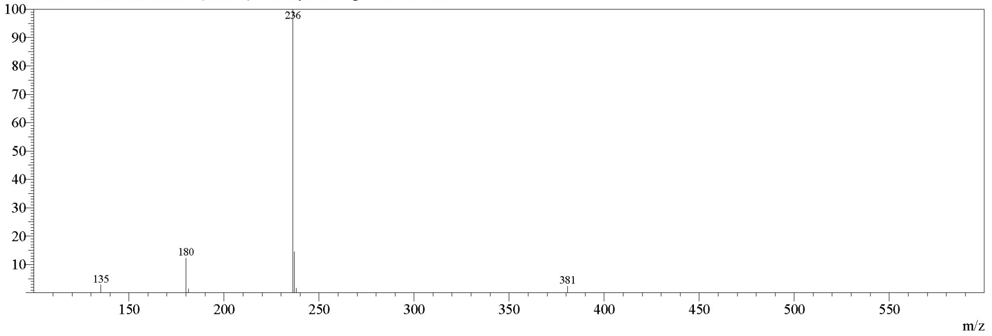

MS Spectrum Table

#:1 Ret.Time:
BG Mode:Calc 2.980<->3.290(299<->330)
Mass Peaks:7 Base Peak:236.05(353179) Polarity:Pos Segment1 - Event1

#	m/z	Abs.Inten.	Rel.Inten.	Charge	Polarity	Monoisotopic	#	m/z	Abs.Inten.	Rel.Inten.	Charge	Polarity	Monoisotopic
1	135.05	9777	2.77				5	237.05	50876	14.41			
2	180.05	43065	12.19				6	238.10	5796	1.64			
3	181.30	5006	1.42				7	380.70	8174	2.31			
4	236.05	353179	100.00										

Figure S153 LCMS spectrum of compound NPD-3002

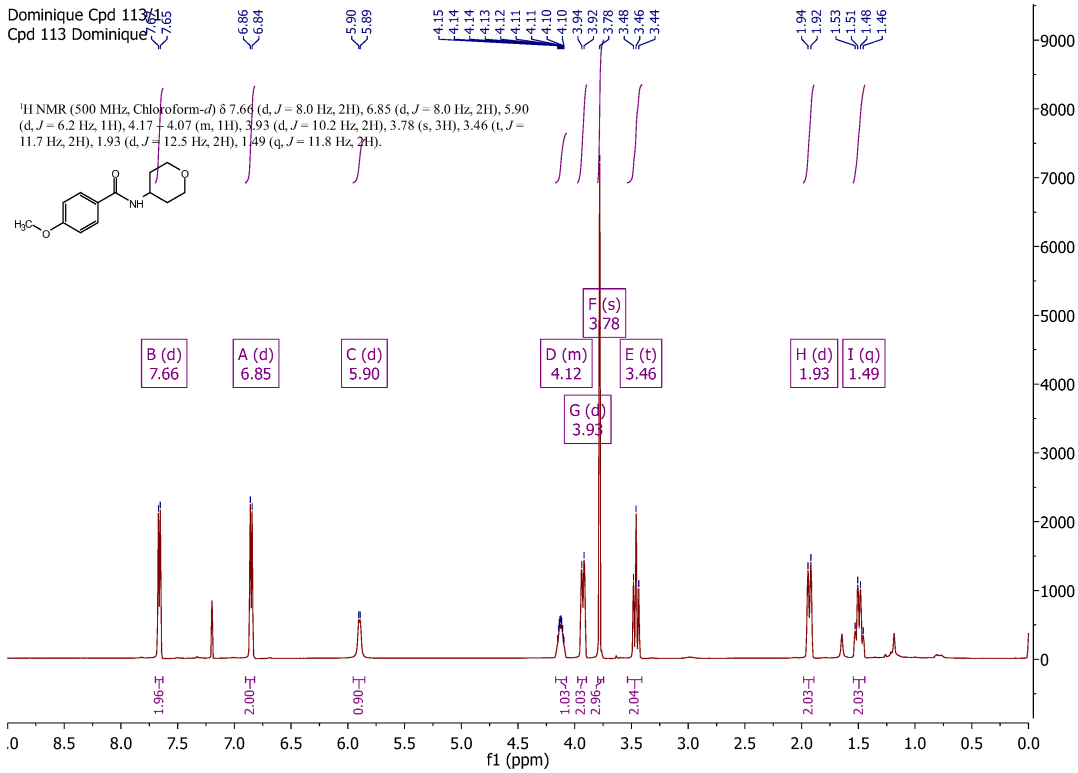

Figure S154 ^{1}H NMR spectrum of compound NPD-3002

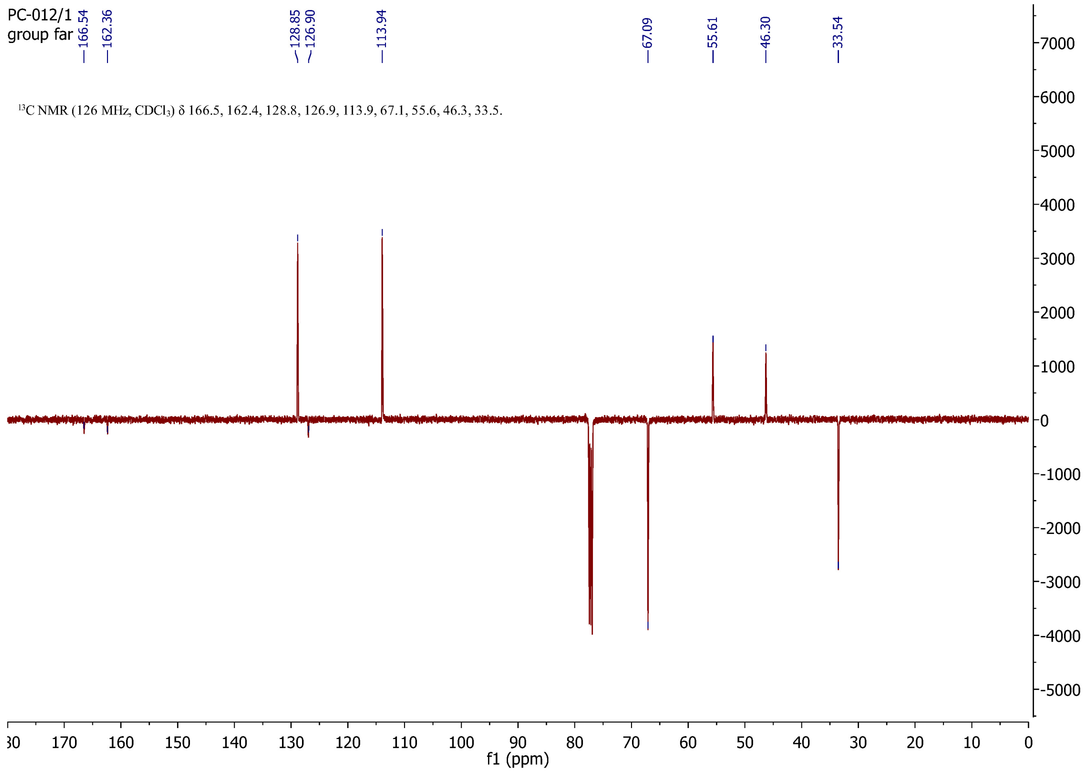

Figure S155 ^{13}C NMR spectrum of compound NPD-3002

Acquired by : Admin
Date Acquired : 2/1/2017 7:03:50 PM
Sample Name : cpd 119 Mohamad
Sample ID :
Tray# : 1
Vial# : 19
Injection Volume : 1
Data File : C:\LabSolutions\Data\2017\2017-WK05\cpd 119 Mohamad.lcd
Background File : Blanco01022017.lcd
Method File : Method SCAN ACID standard MW100.lcm
Report Format : DefaultLCMS.lcr
Tuning File : C:\LabSolutions\Tuning\Tuning-ESI-pos-neg01072015a.lct
Processed by : Admin
Modified Date : 2/23/2017 4:21:11 PM

PDA Graph

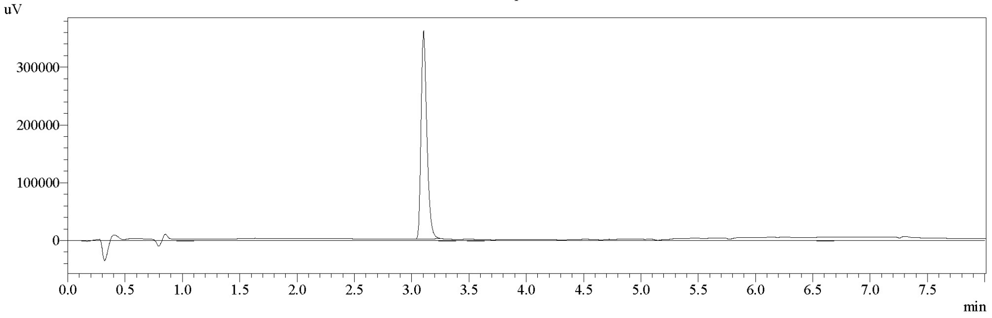

PDA Ch1 254nm 4nm

Peak#	Name	Ret. Time	Area	Area %
1		3.098	1270468	100.000

MS Spectrum Graph

#:1 Ret.Time:Averaged 3.140-3.160(Scan#:315-317)
Mass Peaks:9 Base Peak:236.10(247954) Polarity:Pos Segment1 - Event1

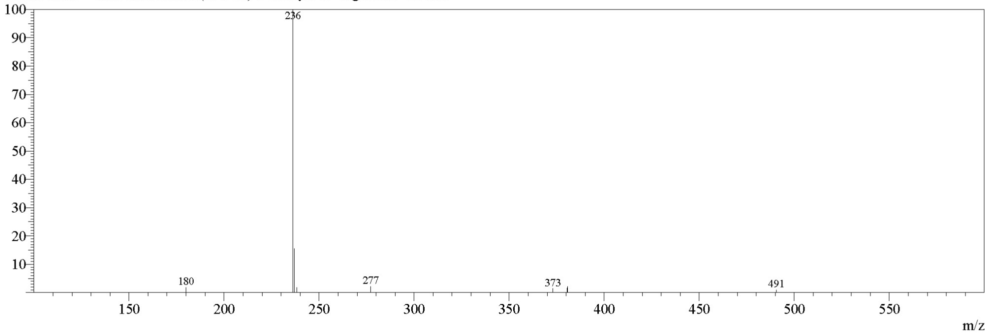

MS Spectrum Table

#:1 Ret.Time:
BG Mode:Calc 3.040<->3.350(305<->336)
Mass Peaks:9 Base Peak:236.10(247954) Polarity:Pos Segment1 - Event1

#	m/z	Abs.Inten.	Rel.Inten.	Charge	Polarity	Monoisotopic
1	179.90	4384	1.77			
2	236.10	247954	100.00			
3	237.05	38579	15.56			
4	238.15	4569	1.84			
5	277.15	5512	2.22			

#	m/z	Abs.Inten.	Rel.Inten.	Charge	Polarity	Monoisotopic
6	373.00	3481	1.40			
7	380.50	3832	1.55			
8	380.80	5476	2.21			
9	490.60	2505	1.01			

Figure S156 LCMS spectrum of compound NPD-3003

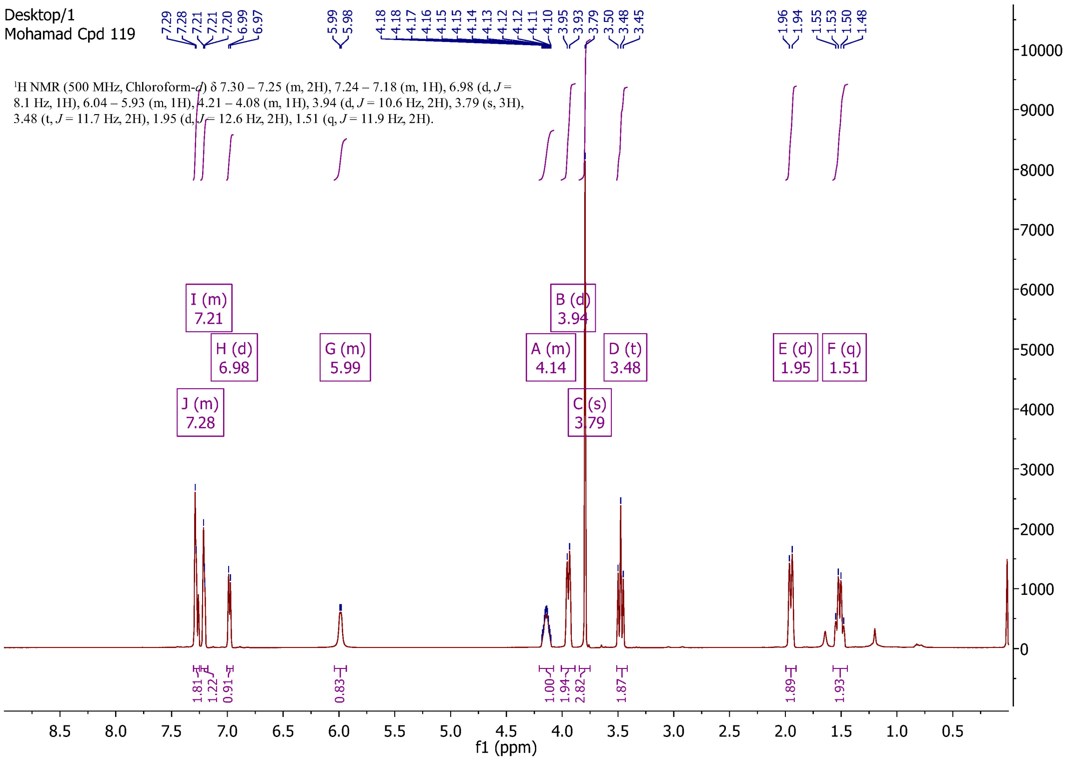

Figure S157 ^{1}H NMR spectrum of compound NPD-3003

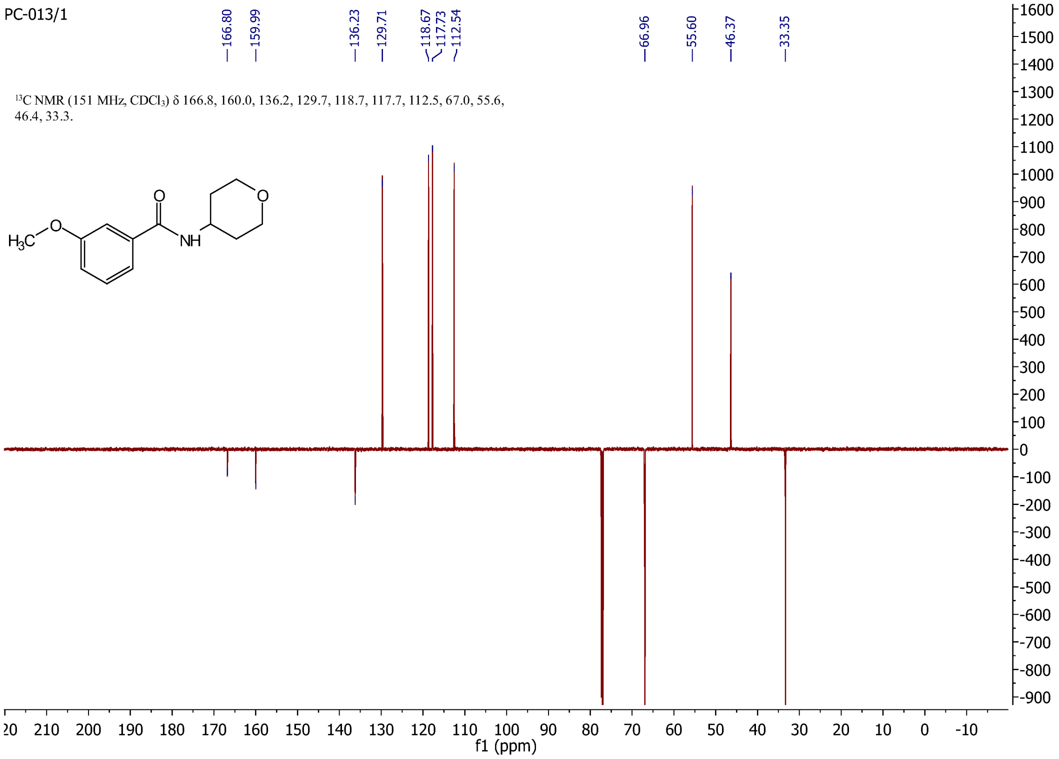

Figure S158 ^{13}C NMR spectrum of compound NPD-3003

Acquired by	: Admin
Date Acquired	: 2/1/2017 7:29:38 PM
Sample Name	: cpd 122-I Maurice
Sample ID	:
Tray#	: 1
Vial#	: 22
Injection Volume	: 1
Data File	: C:\LabSolutions\Data\2017\2017-WK05\cpd 122-I Maurice.lcd
Background File	: Blanco01022017.lcd
Method File	: Method SCAN ACID standard MW100.lcm
Report Format	: DefaultLCMS.lcr
Tuning File	: C:\LabSolutions\Tuning\Tuning-ESI-pos-neg01072015a.lct
Processed by	: Admin
Modified Date	: 2/20/2017 4:07:55 PM

PDA Graph

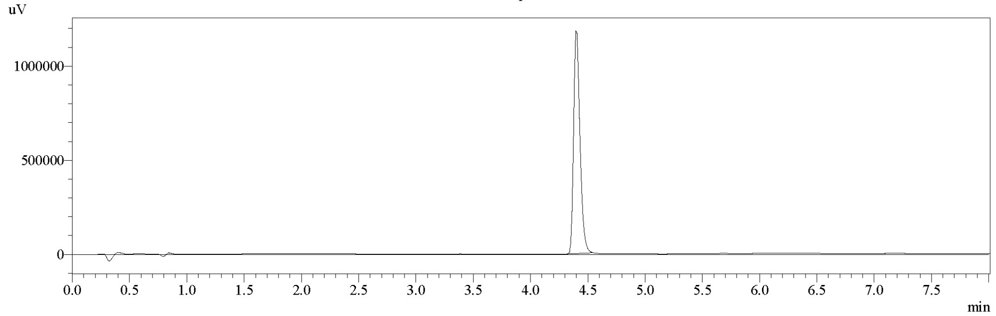

PDA Ch1 254nm 4nm

Peak#	Name	Ret. Time	Area	Area %
1		4.394	4518440	100.000

MS Spectrum Graph

#:1 Ret.Time:Averaged 4.440-4.460(Scan#:445-447)
Mass Peaks:11 Base Peak:292.05(359384) Polarity:Pos Segment1 - Event1

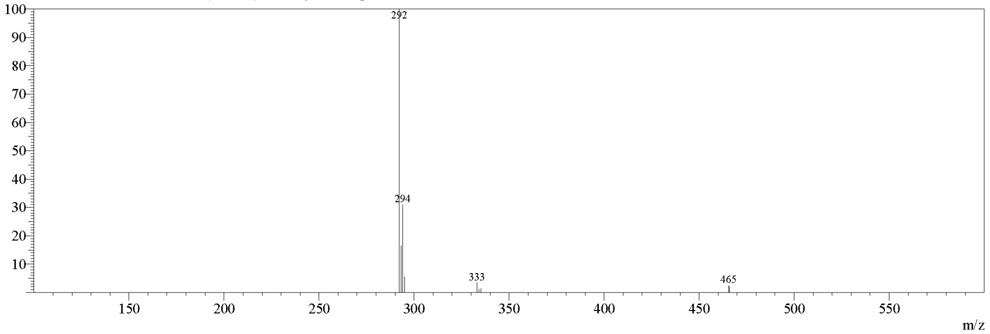

MS Spectrum Table

#:1 Ret.Time:
BG Mode:Calc 4.330<->4.690(434<->470)
Mass Peaks:11 Base Peak:292.05(359384) Polarity:Pos Segment1 - Event1

#	m/z	Abs.Inten.	Rel.Inten.	Charge	Polarity	Monoisotopic
1	292.05	359384	100.00			
2	293.10	59297	16.50			
3	294.00	111312	30.97			
4	295.10	19875	5.53			
5	333.10	12236	3.40			
6	334.10	4080	1.14			
7	335.15	4939	1.37			
8	465.55	8571	2.38			
9	465.95	7184	2.00			
10	610.75	3990	1.11			
11	612.05	4036	1.12			

Figure S159 LCMS spectrum of compound NPD-3004

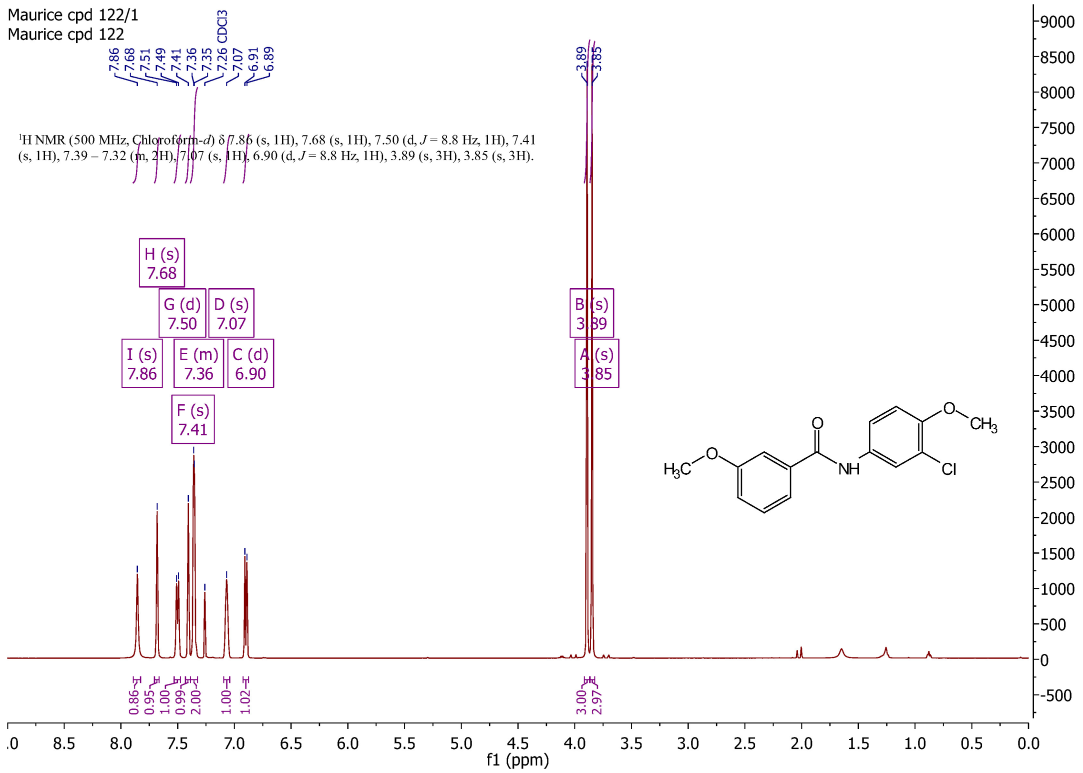

Figure S160 ^{1}H NMR spectrum of compound NPD-3004

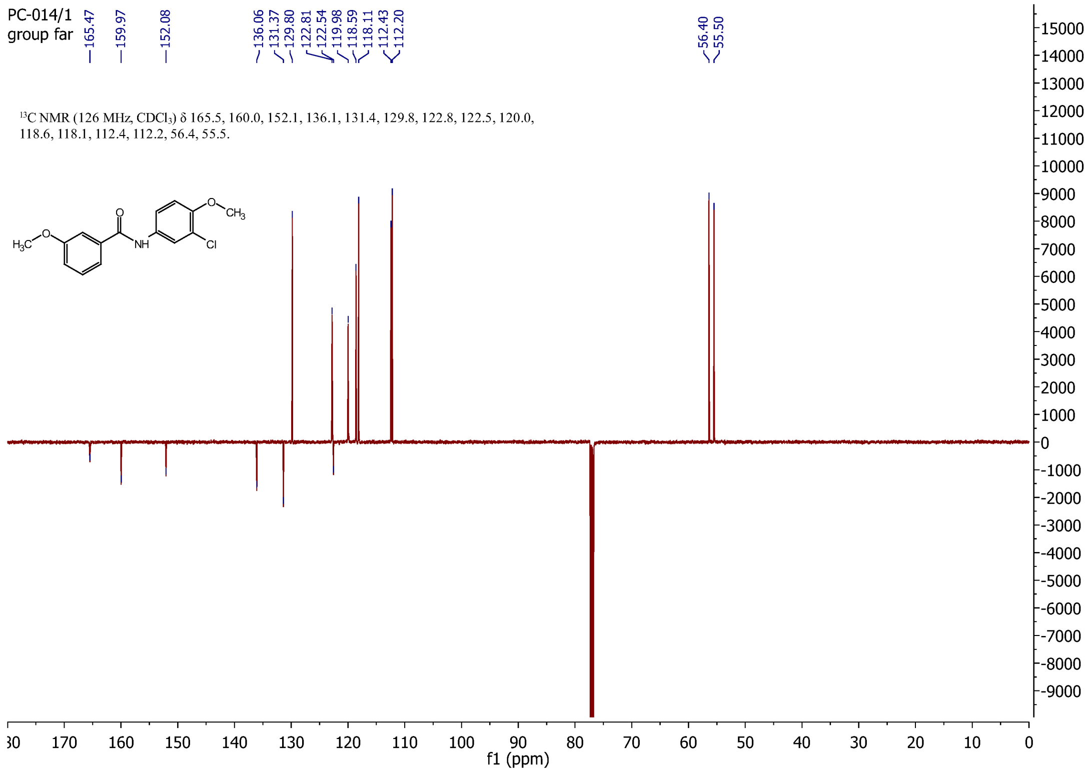

Figure S161 ^{13}C NMR spectrum of compound NPD-3004

Medicinal Chemistry
Vrije Universiteit Amsterdam

Acquired by	: Admin
Date Acquired	: 15/5/2019 2:06:17 PM
Sample Name	: PC-015-1
Sample ID	:
Tray#	: 1
Vial#	: 17
Injection Volume	: 2
Data File	: C:\LabSolutions\Data\2019\2019-wk20\PC-015-1.lcd
Background File	: blanco 15052019.lcd
Method File	: Method SCAN ACID standard.lcm
Report Format	: DefaultLCMS.lcr
Tuning File	: C:\LabSolutions\Tuning\Tuning-ESI-pos-neg01072015a.lct
Processed by	: Admin
Modified Date	: 15/5/2019 3:39:17 PM

PDA Graph

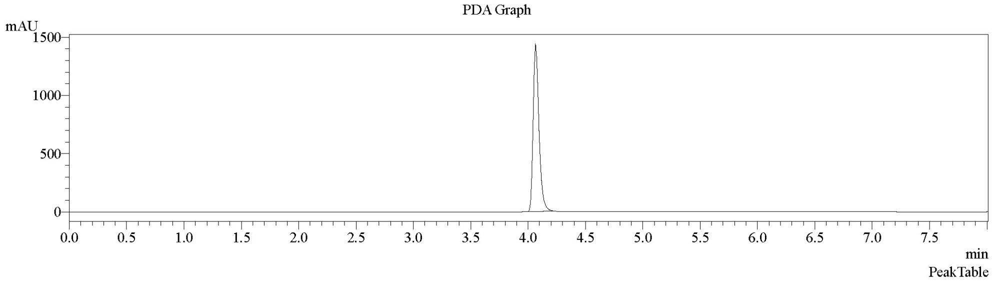

PeakTable

PDA Ch1 254nm 4nm

Peak#	Name	Ret. Time	Area	Area %
1		4.060	5063651	99.841
2		4.708	8066	0.159

MS Chromatogram
PC-015-1 C:\LabSolutions\Data\2019\2019-wk20\PC-015-1.lcd

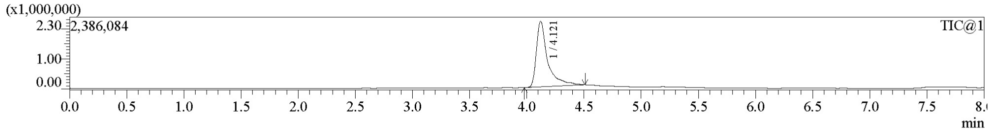

MS Spectrum Graph

#:1 Ret.Time:Averaged 4.110-4.130(Scan#:412-414)
BG Mode:Calc 3.980<->4.510(399<->452)
Mass Peaks:7 Base Peak:296.95(1033989) Polarity:Pos Segment1 - Event1

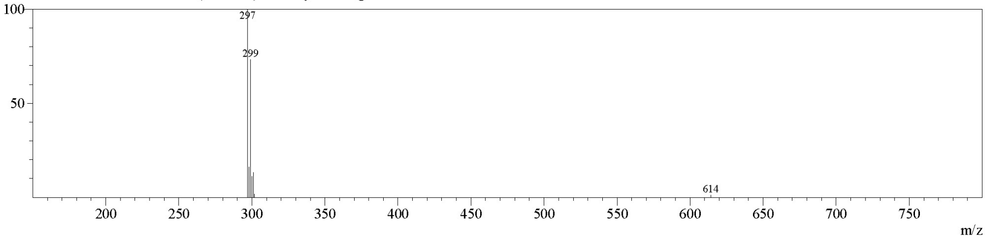

MS Spectrum Table

#:1 Ret.Time:
BG Mode:Calc 3.980<->4.510(399<->452)
Mass Peaks:7 Base Peak:296.95(1033989) Polarity:Pos Segment1 - Event1

#	m/z	Abs.Inten.	Rel.Inten.	Charge	Polarity	Monoisotopic
1	296.95	1033989	100.00			
2	298.00	166920	16.14			
3	299.00	759043	73.41			
4	299.95	113931	11.02			
5	300.95	137316	13.28			
6	301.85	17748	1.72			
7	614.15	11671	1.13			

Figure S162 LCMS spectrum of compound NPD-3005

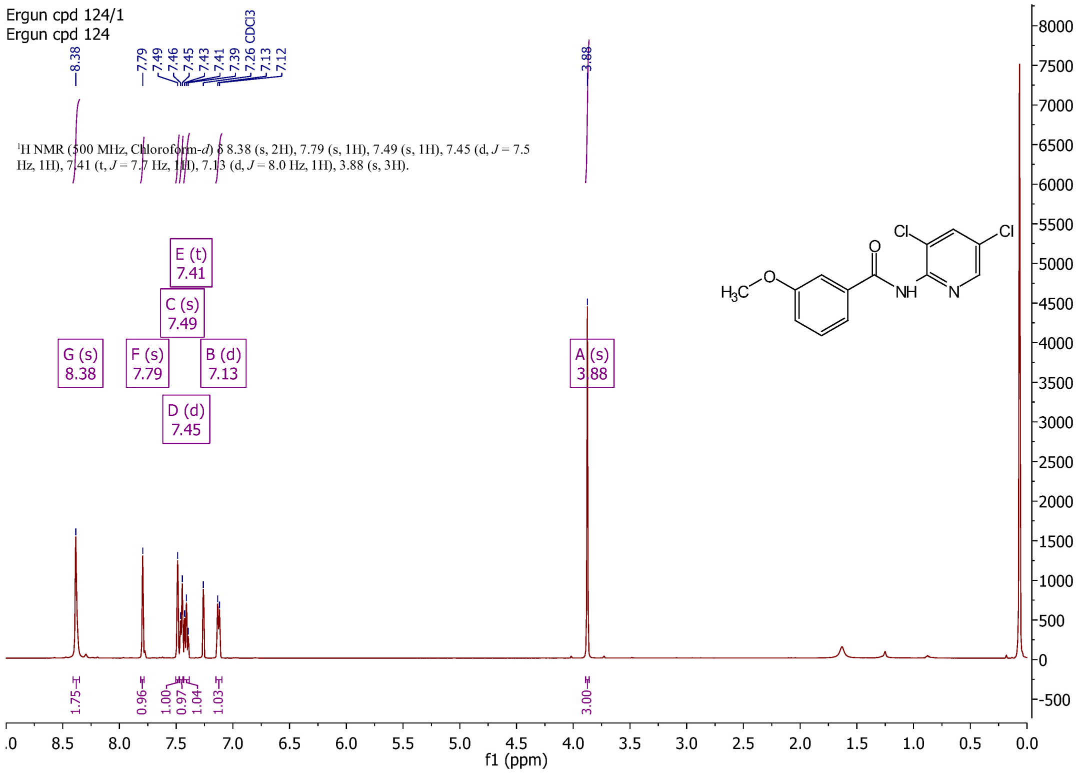

Figure S163 ^{1}H NMR spectrum of compound NPD-3005

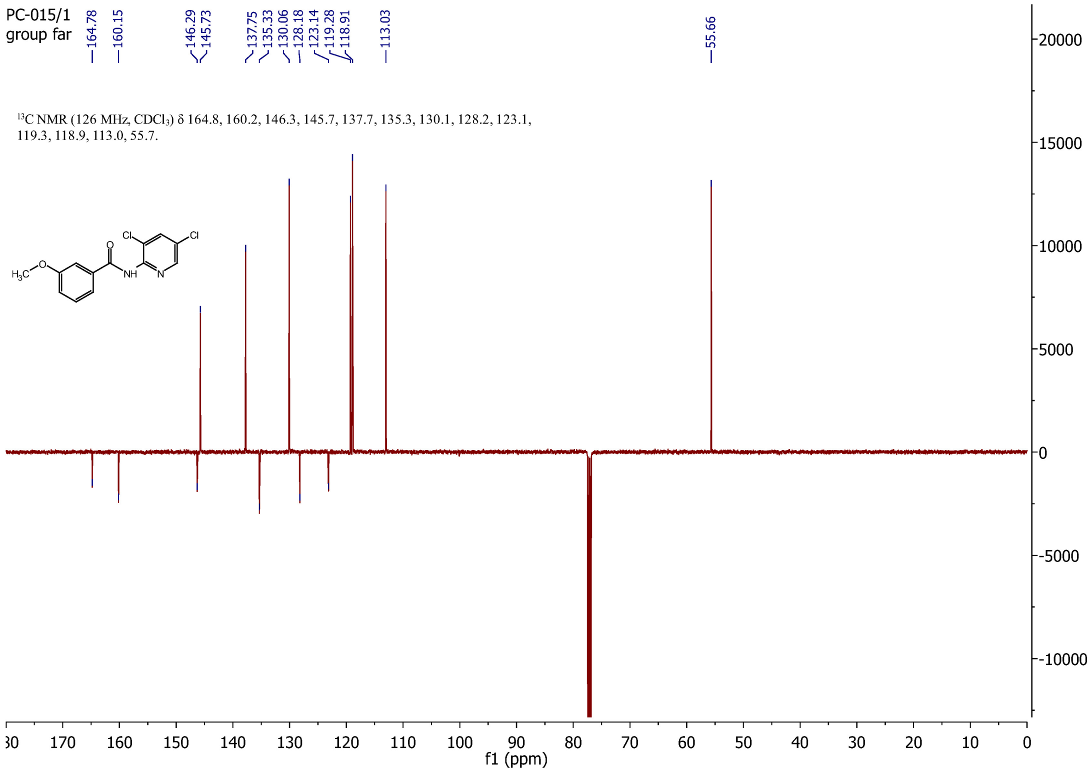

Figure S164 ^{13}C NMR spectrum of compound NPD-3005

Medicinal Chemistry
Vrije Universiteit Amsterdam

Acquired by	: Admin
Date Acquired	: 22/5/2019 12:27:18 PM
Sample Name	: VUF16127
Sample ID	:
Tray#	: 1
Vial#	: 7
Injection Volume	: 2
Data File	: C:\LabSolutions\Data\2019\2019-wk21\VUF16127.lcd
Background File	: blanco 22052019.lcd
Method File	: Method SCAN ACID standard.lcm
Report Format	: DefaultLCMS.lcr
Tuning File	: C:\LabSolutions\Tuning\Tuning-ESI-pos-neg01072015a.lct
Processed by	: Admin
Modified Date	: 22/5/2019 12:59:26 PM

PDA Graph

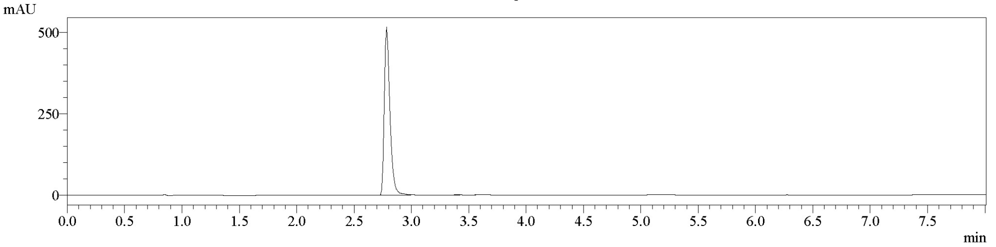

PeakTable

PDA Ch1 254nm 4nm

Peak#	Name	Ret. Time	Area	Area %
1		2.393	2190	0.125
2		2.778	1738088	99.543
3		3.387	1798	0.103
4		3.593	4001	0.229

MS Chromatogram
VUF16127 C:\LabSolutions\Data\2019\2019-wk21\VUF16127.lcd

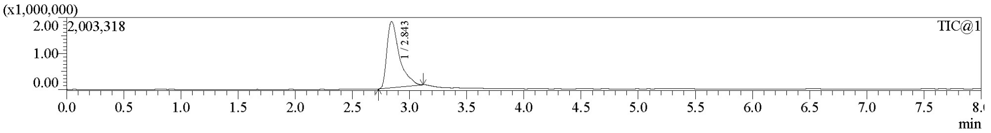

MS Spectrum Graph

#:1 Ret.Time:Averaged 2.830-2.850(Scan#:284-286)
BG Mode:Calc 2.730<->3.120(274<->313)
Mass Peaks:8 Base Peak:280.10(1332668) Polarity:Pos Segment1 - Event1

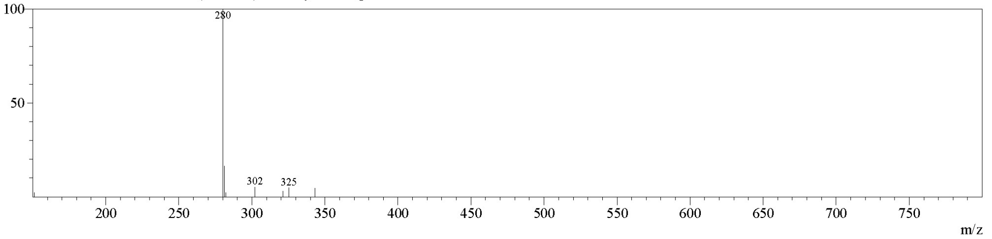

MS Spectrum Table

#:1 Ret.Time:
BG Mode:Calc 2.730<->3.120(274<->313)
Mass Peaks:8 Base Peak:280.10(1332668) Polarity:Pos Segment1 - Event1

#	m/z	Abs.Inten.	Rel.Inten.	Charge	Polarity	Monoisotopic	#	m/z	Abs.Inten.	Rel.Inten.	Charge	Polarity	Monoisotopic
1	151.00	30849	2.31				5	302.05	66981	5.03			
2	280.10	1332668	100.00				6	321.10	39725	2.98			
3	281.10	219538	16.47				7	325.20	62900	4.72			
4	282.05	28743	2.16				8	343.15	61585	4.62			

Figure S165 LCMS spectrum of compound NPD-3006

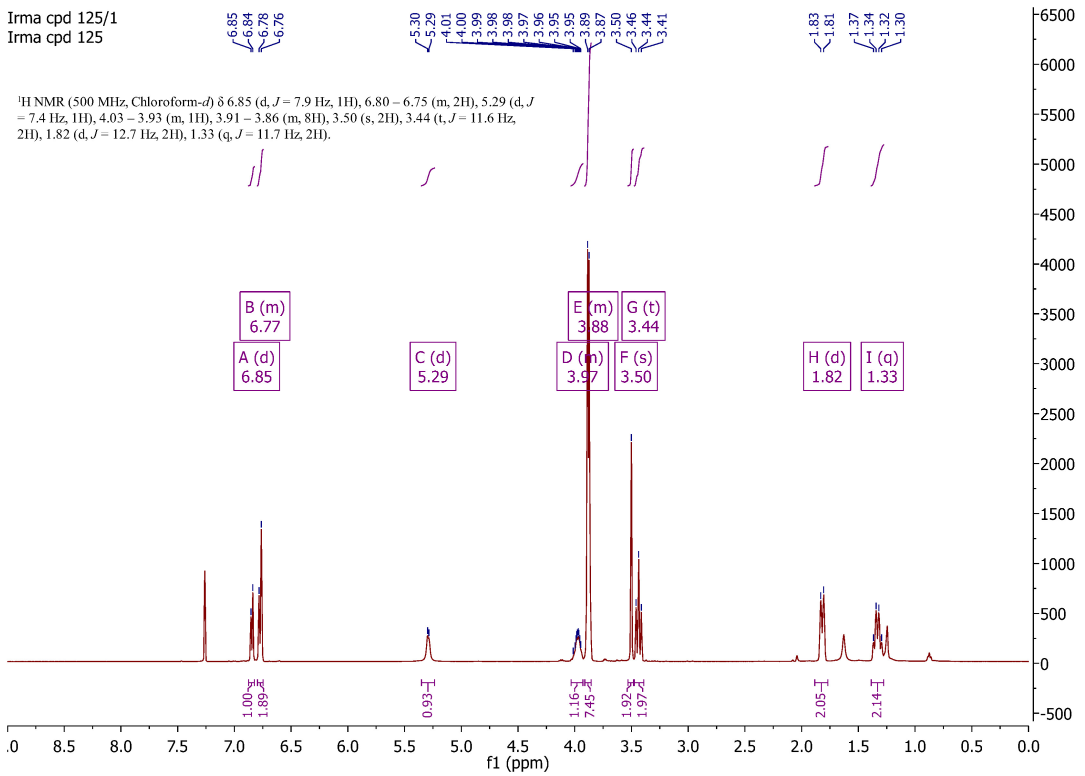

Figure S166 ^{1}H NMR spectrum of compound NPD-3006

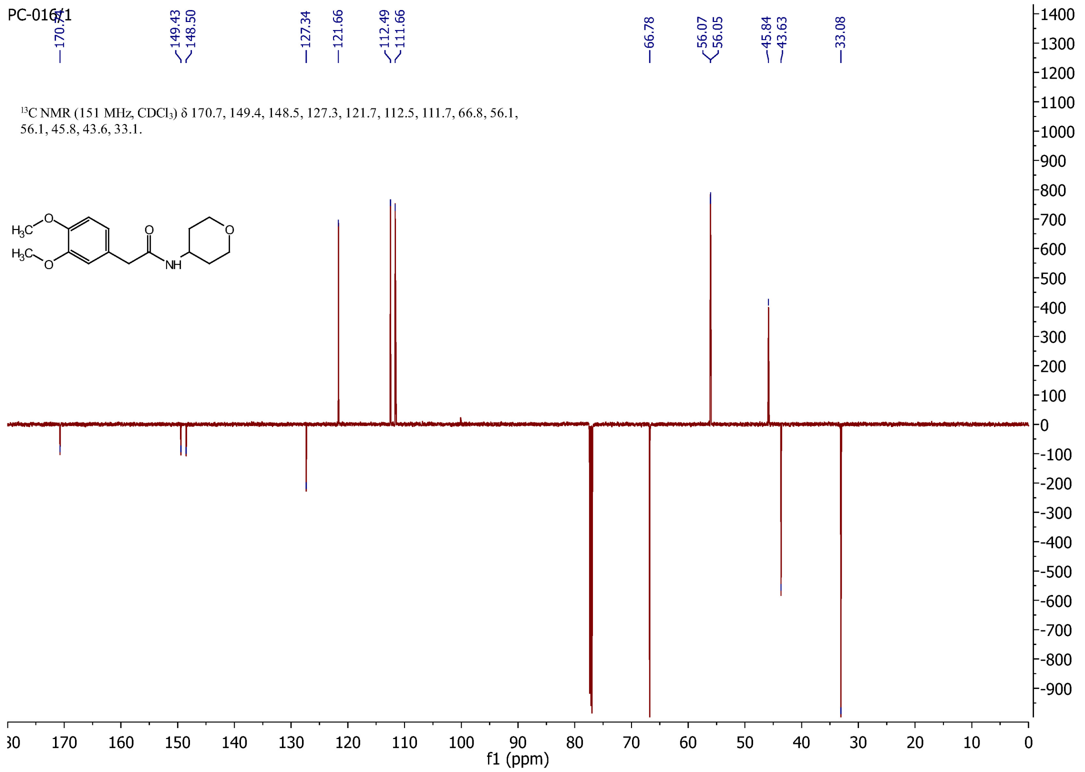

Figure S167 ^{13}C NMR spectrum of compound NPD-3006

Acquired by	: Admin
Date Acquired	: 2/1/2017 9:56:02 PM
Sample Name	: cpd 143 Narjisse
Sample ID	:
Tray#	: 1
Vial#	: 39
Injection Volume	: 1
Data File	: C:\LabSolutions\Data\2017\2017-WK05\cpd 143 Narjisse.lcd
Background File	: Blanco01022017.lcd
Method File	: Method SCAN ACID standard MW100.lcm
Report Format	: DefaultLCMS.lcr
Tuning File	: C:\LabSolutions\Tuning\Tuning-ESI-pos-neg01072015a.lct
Processed by	: Admin
Modified Date	: 2/20/2017 4:10:08 PM

PDA Graph

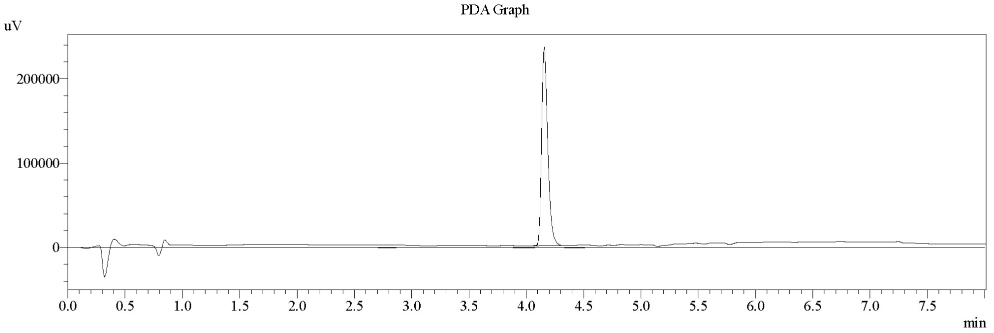

PDA Ch1 254nm 4nm

Peak#	Name	Ret. Time	Area	Area %
1		4.151	886700	100.000

MS Spectrum Graph

#:1 Ret.Time:Averaged 4.200-4.220(Scan#:421-423)
Mass Peaks:8 Base Peak:342.10(393978) Polarity:Pos Segment1 - Event1

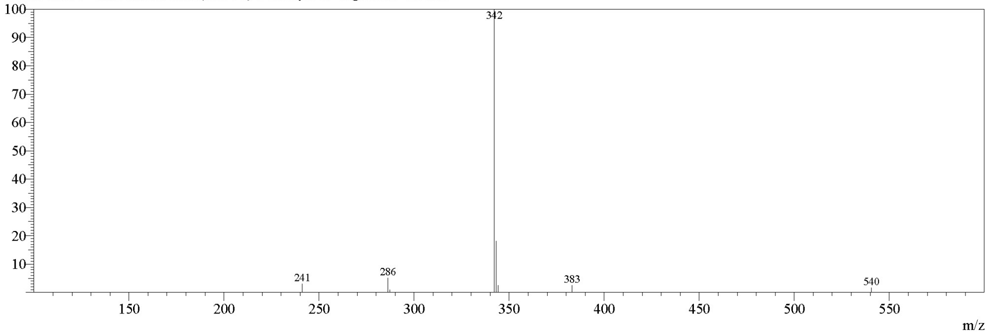

MS Spectrum Table

#:1 Ret.Time:
BG Mode:Calc 4.090<->4.390(410<->440)
Mass Peaks:8 Base Peak:342.10(393978) Polarity:Pos Segment1 - Event1

#	m/z	Abs.Inten.	Rel.Inten.	Charge	Polarity	Monoisotopic
1	241.00	12221	3.10			
2	286.10	20021	5.08			
3	287.10	3949	1.00			
4	342.10	393978	100.00			
5	343.15	71695	18.20			
6	344.15	10110	2.57			
7	383.05	9814	2.49			
8	540.50	6261	1.59			

Figure S168 LCMS spectrum of compound NPD-3007

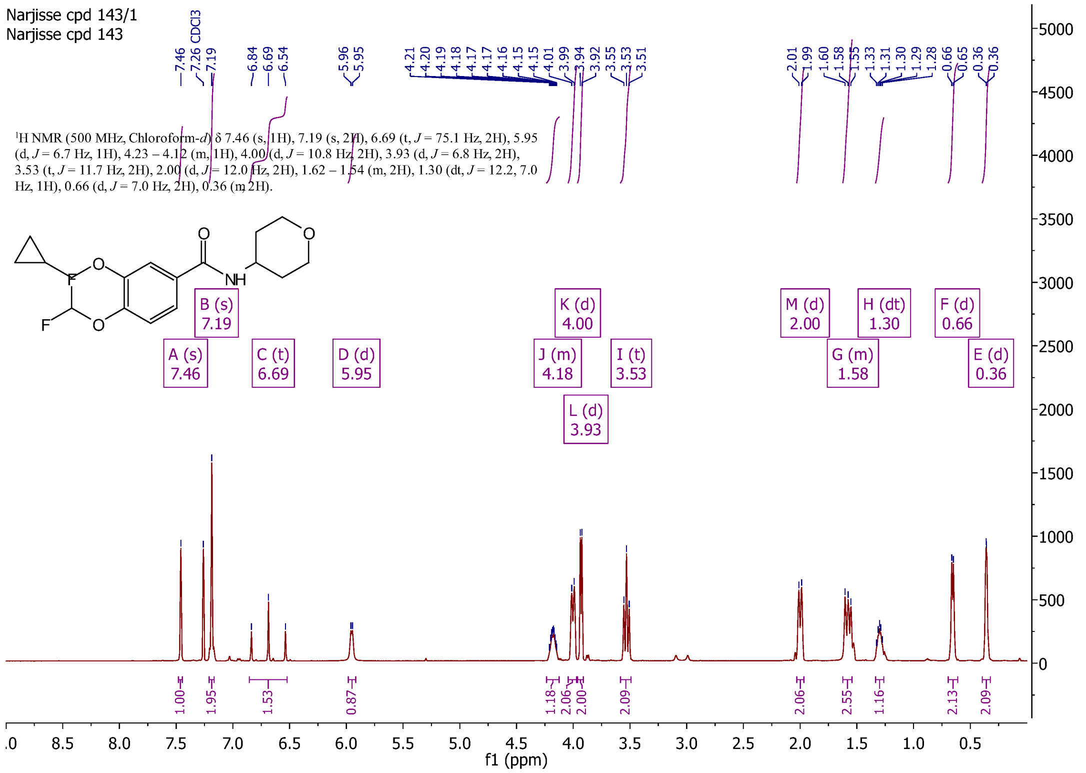

Figure S169 ^{1}H NMR spectrum of compound NPD-3007

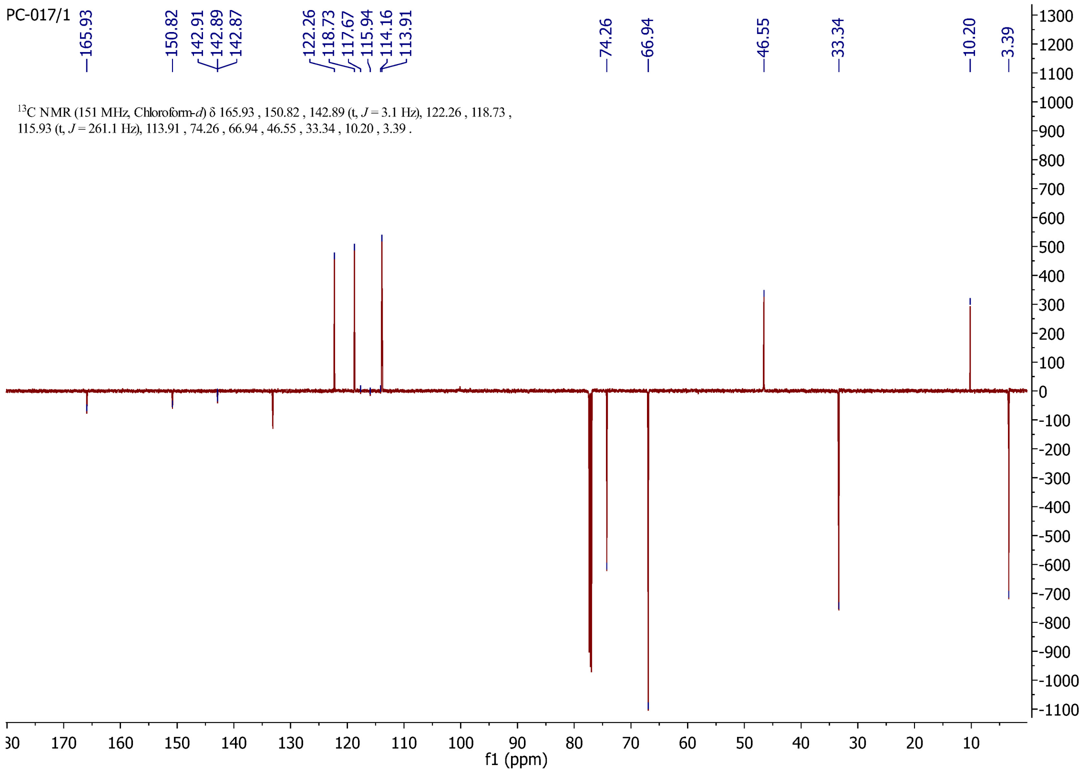

Figure S170 ^{13}C NMR spectrum of compound NPD-3007

Acquired by	: Admin
Date Acquired	: 4/19/2017 10:40:22 AM
Sample Name	: YAZH01-079-3
Sample ID	:
Tray#	: 1
Vial#	: 18
Injection Volume	: 1
Data File	: C:\LabSolutions\Data\2017\2017-wk16\YAZH01-079-3.lcd
Background File	: blanco 19042017.lcd
Method File	: Method SCAN ACID standard.lcm
Report Format	: DefaultLCMS.lcr
Tuning File	: C:\LabSolutions\Tuning\Tuning-ESI-pos-neg01072015a.lct
Processed by	: Admin
Modified Date	: 4/19/2017 11:08:57 AM

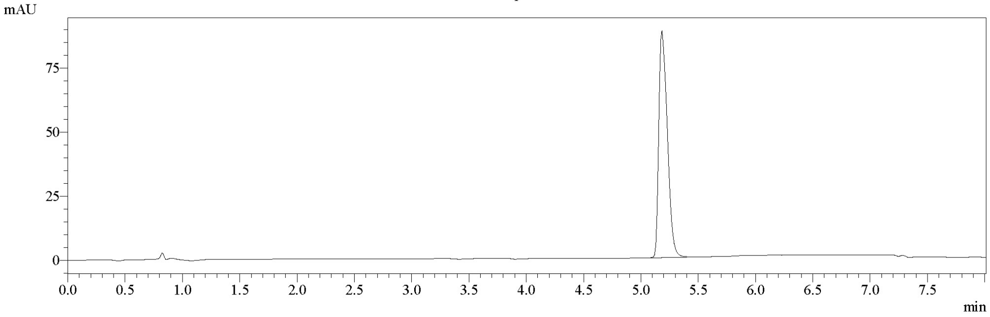

PDA Ch1 254nm 4nm

Peak#	Name	Ret. Time	Area	Area %
1		5.178	460616	100.000

MS Spectrum Graph

#:1 Ret.Time:Single 5.250(Scan#:526)
Mass Peaks:18 Base Peak:368.00(22258) Polarity:Pos Segment1 - Event1

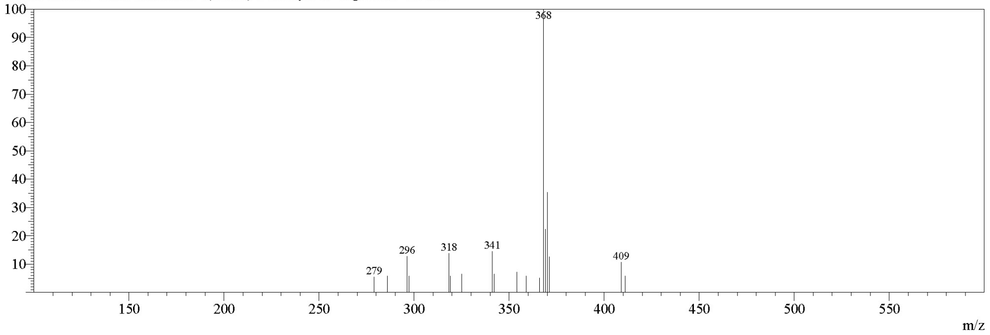

MS Spectrum Table

#:1 Ret.Time:
BG Mode:None
Mass Peaks:18 Base Peak:368.00(22258) Polarity:Pos Segment1 - Event1

#	m/z	Abs.Inten.	Rel.Inten.	Charge	Polarity	Monoisotopic
1	278.85	1220	5.48			
2	285.85	1285	5.77			
3	296.20	2831	12.72			
4	297.20	1297	5.83			
5	318.20	3083	13.85			
6	319.15	1303	5.85			
7	325.15	1431	6.43			
8	341.15	3222	14.48			
9	342.15	1463	6.57			

#	m/z	Abs.Inten.	Rel.Inten.	Charge	Polarity	Monoisotopic
10	354.10	1616	7.26			
11	359.10	1286	5.78			
12	366.10	1123	5.05			
13	368.00	22258	100.00			
14	369.15	4968	22.32			
15	370.05	7876	35.39			
16	371.00	2806	12.61			
17	408.95	2369	10.64			
18	410.95	1282	5.76			

Figure S171 LCMS spectrum of compound NPD-3075

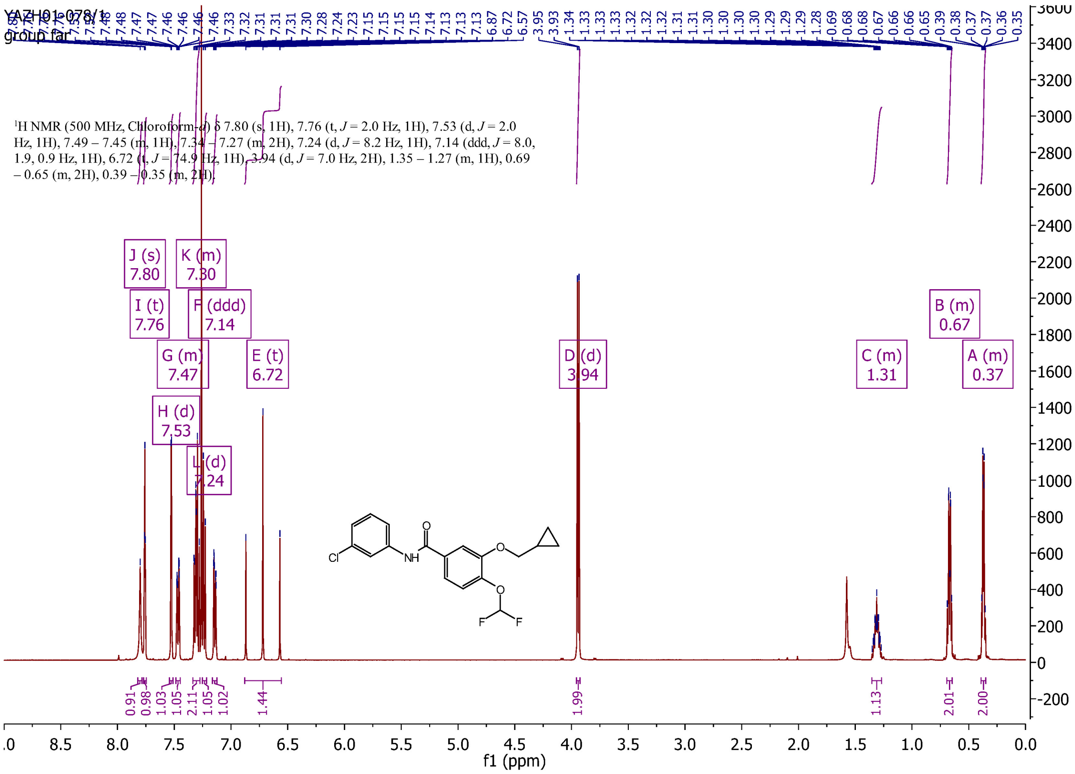

Figure S172 ^{1}H NMR spectrum of compound NPD-3075

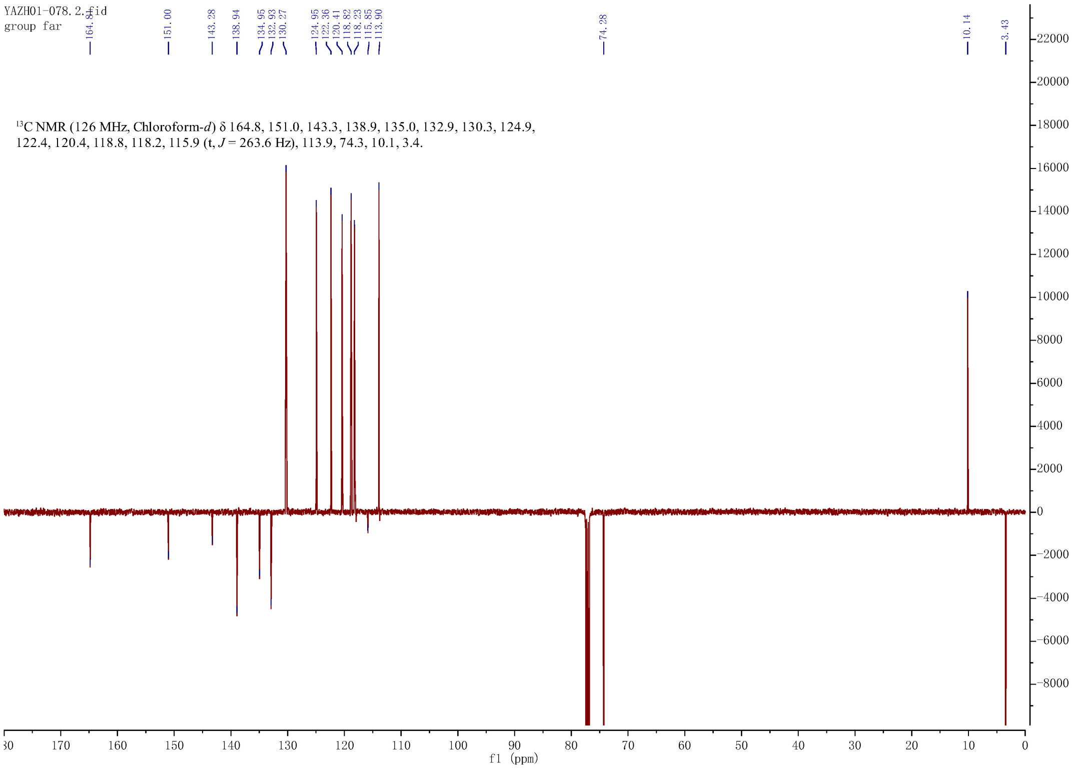

Figure S173 ^{13}C NMR spectrum of compound NPD-3075

Medicinal Chemistry
Vrije Universiteit Amsterdam

Acquired by	: Admin
Date Acquired	: 12/5/2017 11:50:26 AM
Sample Name	: YAZH01-079
Sample ID	:
Tray#	: 1
Vial#	: 22
Injection Volume	: 1
Data File	: C:\LabSolutions\Data\2017\2017-wk19\YAZH01-079.lcd
Background File	: blanco 12052017.lcd
Method File	: Method SCAN ACID standard.lcm
Report Format	: DefaultLCMS.lcr
Tuning File	: C:\LabSolutions\Tuning\Tuning-ESI-pos-neg01072015a.lct
Processed by	: Admin
Modified Date	: 14/6/2019 12:10:57 PM

PDA Graph

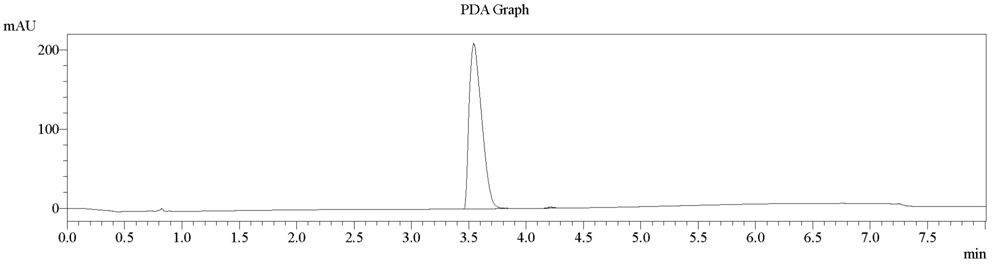

PeakTable

PDA Ch1 254nm 4nm

Peak#	Name	Ret. Time	Area	Area %
1		3.539	1584592	99.557
2		4.205	7059	0.443

MS Chromatogram
YAZH01-079 C:\LabSolutions\Data\2017\2017-wk11-20\2017-wk19\YAZH01-079.lcd

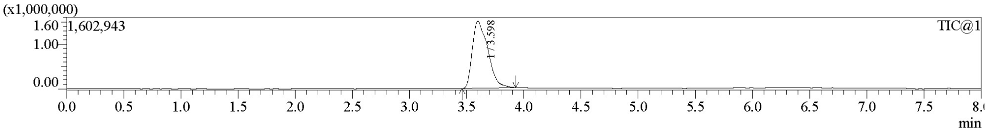

MS Spectrum Graph

#:1 Ret.Time:Averaged 3.590-3.610(Scan#:360-362)
BG Mode:Calc 3.460<->3.930(347<->394)
Mass Peaks:4 Base Peak:335.15(1242993) Polarity:Pos Segment1 - Event1

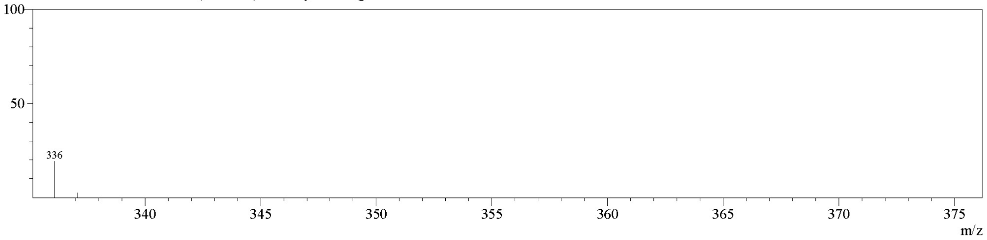

MS Spectrum Table

#:1 Ret.Time:
BG Mode:Calc 3.460<->3.930(347<->394)
Mass Peaks:4 Base Peak:335.15(1242993) Polarity:Pos Segment1 - Event1

#	m/z	Abs.Inten.	Rel.Inten.	Charge	Polarity	Monoisotopic
1	335.15	1242993	100.00			
2	336.10	238815	19.21			
3	337.10	29418	2.37			
4	376.20	30999	2.49			

Figure S174 LCMS spectrum of compound NPD-3076

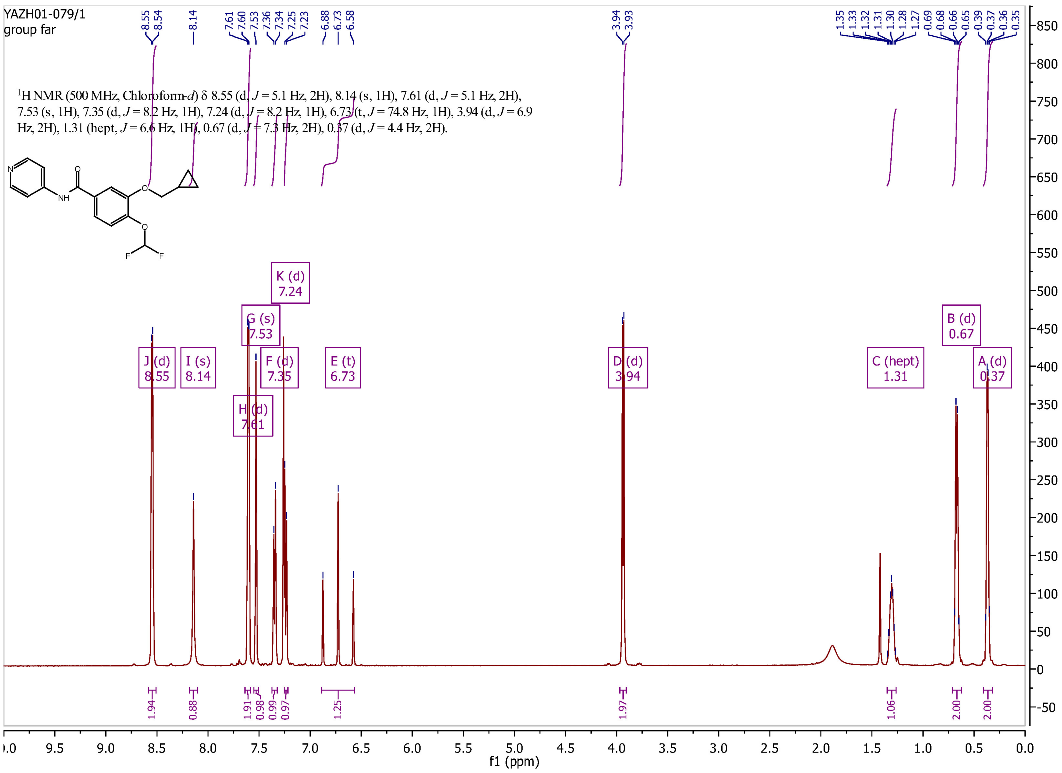

Figure S175 ^{1}H NMR spectrum of compound NPD-3076

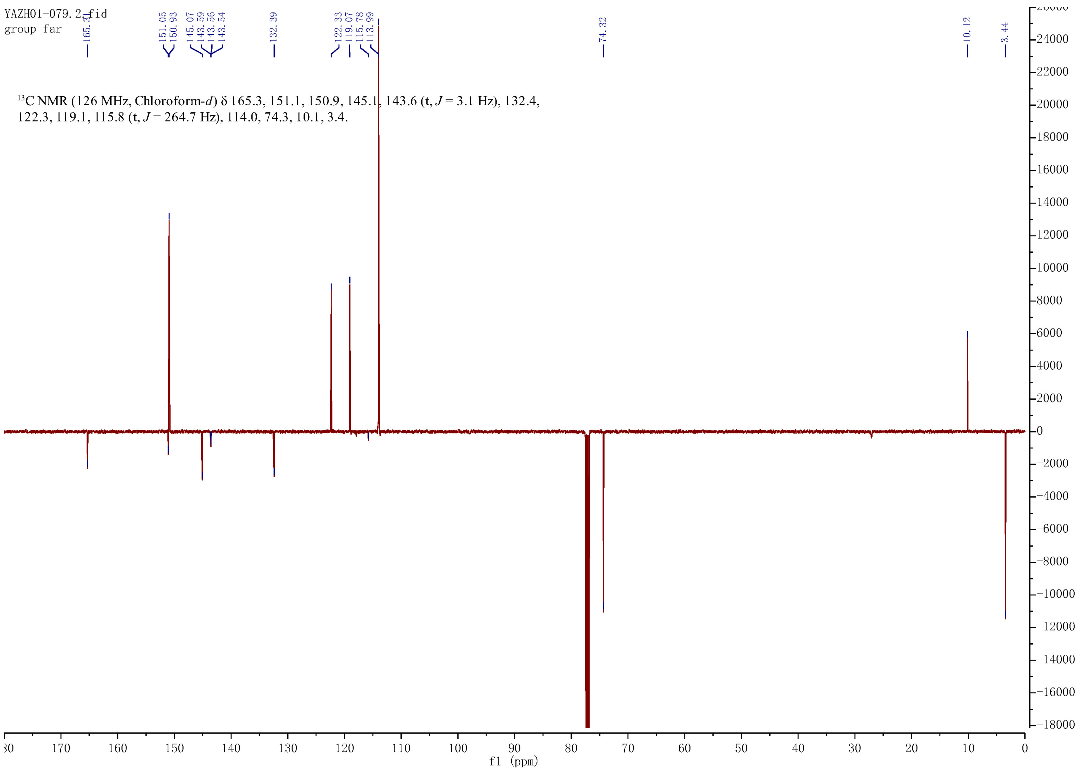

Figure S176 ^{13}C NMR spectrum of compound NPD-3076

Medicinal Chemistry
Vrije Universiteit Amsterdam

Acquired by : Admin
Date Acquired : 4/24/2017 10:17:07 AM
Sample Name : YAZH01-084
Sample ID :
Tray# : 1
Vial# : 15
Injection Volume : 1
Data File : C:\LabSolutions\Data\2017\2017-wk17\YAZH01-084.lcd
Background File : blanco 24042017.lcd
Method File : Method SCAN ACID standard.lcm
Report Format : DefaultLCMS.lcr
Tuning File : C:\LabSolutions\Tuning\Tuning-ESI-pos-neg01072015a.lct
Processed by : Admin
Modified Date : 4/24/2017 12:09:47 PM

Chromatogram
YAZH01-084 C:\LabSolutions\Data\2017\2017-wk17\YAZH01-084.lcd

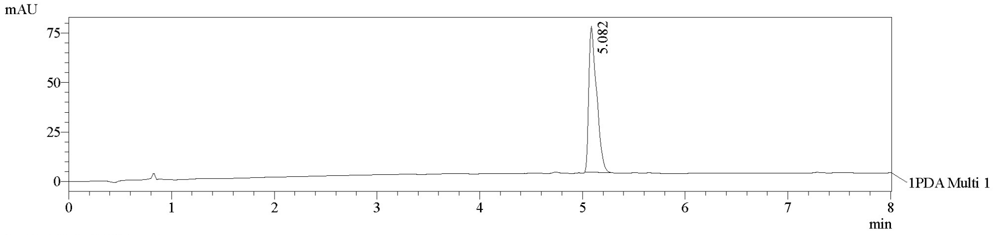

1 PDA Multi 1 / 254nm 4nm

PeakTable

PDA Ch1 254nm 4nm

Peak#	Name	Ret. Time	Area	Area %
1		5.082	386966	100.000

MS Chromatogram
YAZH01-084 C:\LabSolutions\Data\2017\2017-wk17\YAZH01-084.lcd

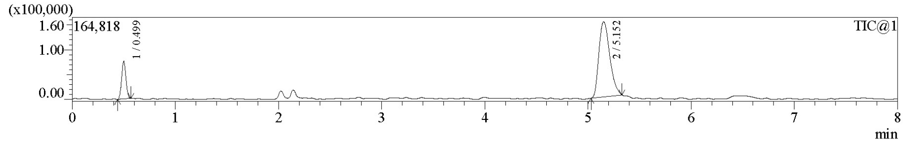

MS Spectrum Graph

#:1 Ret.Time:Averaged 5.140-5.160(Scan#:515-517)
Mass Peaks:17 Base Peak:368.05(79748) Polarity:Pos Segment1 - Event1

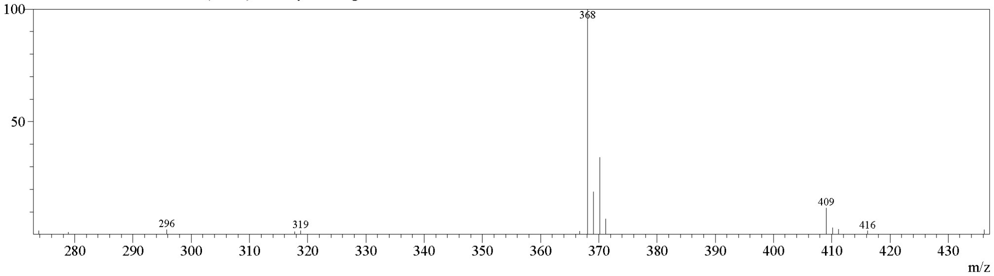

MS Spectrum Table

#:1 Ret.Time:
BG Mode:Calc 5.030<->5.330(504<->534)
Mass Peaks:17 Base Peak:368.05(79748) Polarity:Pos Segment1 - Event1

#	m/z	Abs.Inten.	Rel.Inten.	Charge	Polarity	Monoisotopic
1	272.85	1747	2.19			
2	273.85	1303	1.63			
3	278.85	828	1.04			
4	295.80	1617	2.03			
5	317.80	932	1.17			
6	318.75	1363	1.71			
7	366.70	1133	1.42			
8	368.05	79748	100.00			

#	m/z	Abs.Inten.	Rel.Inten.	Charge	Polarity	Monoisotopic
9	369.05	15089	18.92			
10	370.15	27281	34.21			
11	371.20	5504	6.90			
12	409.05	9311	11.68			
13	410.15	2387	2.99			
14	411.15	1756	2.20			
15	416.15	1230	1.54			
16	436.20	1718	2.15			

Figure S177 LCMS spectrum of compound NPD-3080

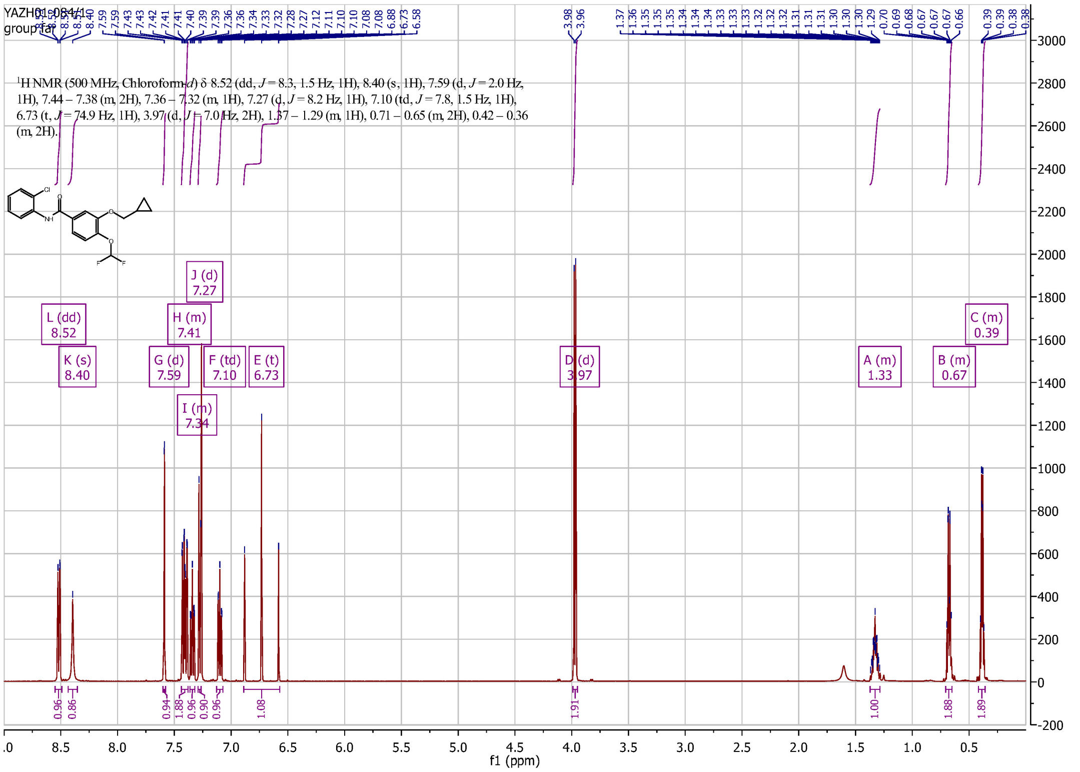

Figure S178 ^{1}H NMR spectrum of compound NPD-3080

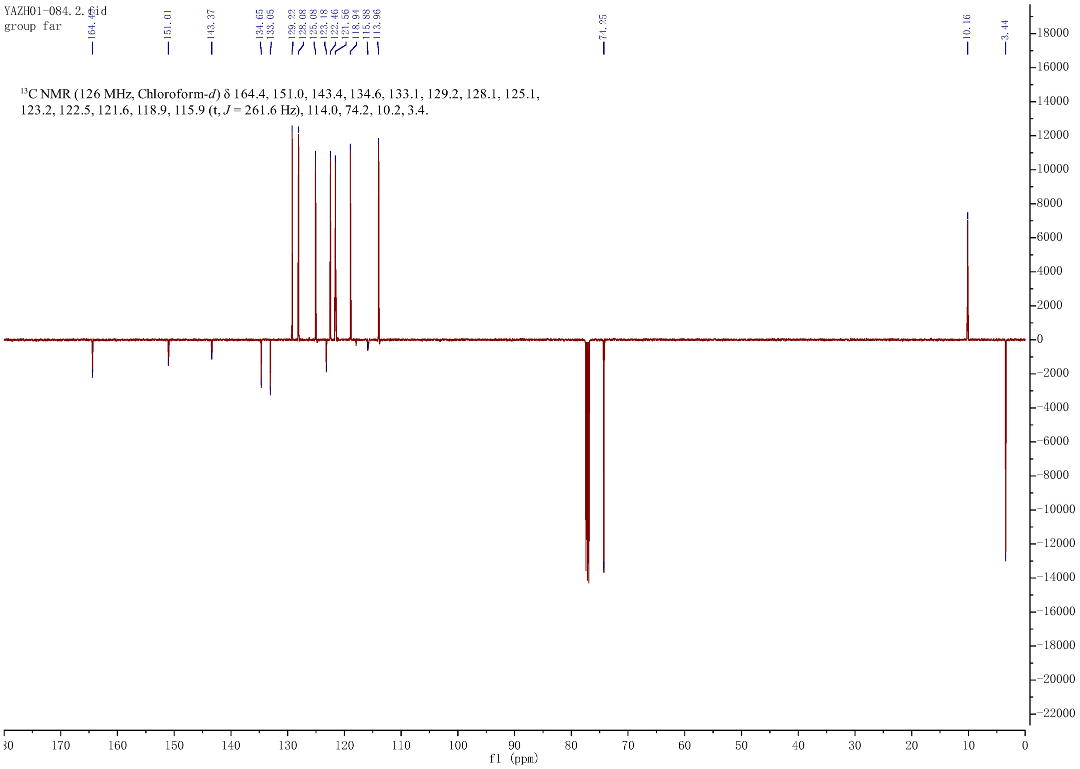

Figure S179 ^{13}C NMR spectrum of compound NPD-3080

Acquired by	: Admin
Date Acquired	: 12/5/2017 12:07:39 PM
Sample Name	: YAZH01-088
Sample ID	:
Tray#	: 1
Vial#	: 23
Injection Volume	: 1
Data File	: C:\LabSolutions\Data\2017\2017-wk19\YAZH01-088.lcd
Background File	: blanco 12052017.lcd
Method File	: Method SCAN ACID standard.lcm
Report Format	: DefaultLCMS.lcr
Tuning File	: C:\LabSolutions\Tuning\Tuning-ESI-pos-neg01072015a.lct
Processed by	: Admin
Modified Date	: 10/5/2019 1:04:48 PM

PDA Graph

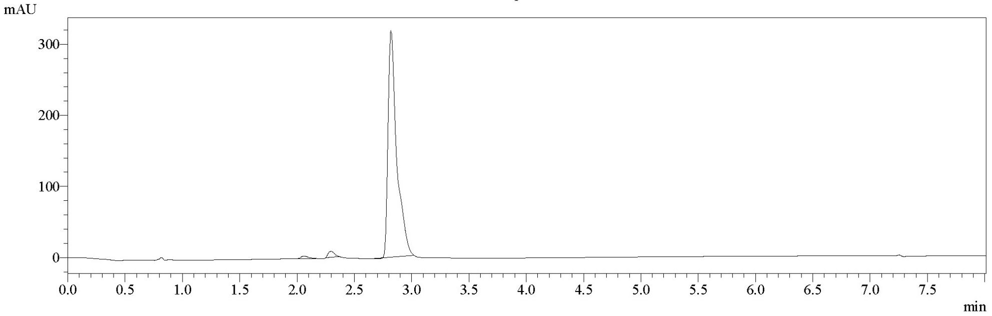

PDA Ch1 254nm 4nm

Peak#	Name	Ret. Time	Area	Area %
1		2.055	16150	0.935
2		2.290	30545	1.769
3		2.815	1680226	97.296

MS Spectrum Graph

#:1 Ret.Time:Averaged 2.870-2.890(Scan#:288-290)
Mass Peaks:6 Base Peak:260.05(166073) Polarity:Pos Segment1 - Event1

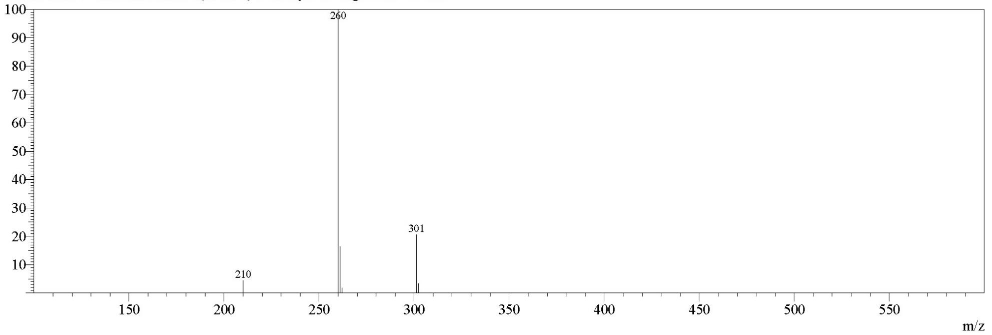

MS Spectrum Table

#:1 Ret.Time:
BG Mode:Calc 2.770<->3.100(278<->311)
Mass Peaks:6 Base Peak:260.05(166073) Polarity:Pos Segment1 - Event1

#	m/z	Abs.Inten.	Rel.Inten.	Charge	Polarity	Monoisotopic
1	209.90	7420	4.47			
2	260.05	166073	100.00			
3	261.10	27240	16.40			

#	m/z	Abs.Inten.	Rel.Inten.	Charge	Polarity	Monoisotopic
4	262.15	3063	1.84			
5	301.10	34183	20.58			
6	302.20	5541	3.34			

Figure S180 LCMS spectrum of compound NPD-3081

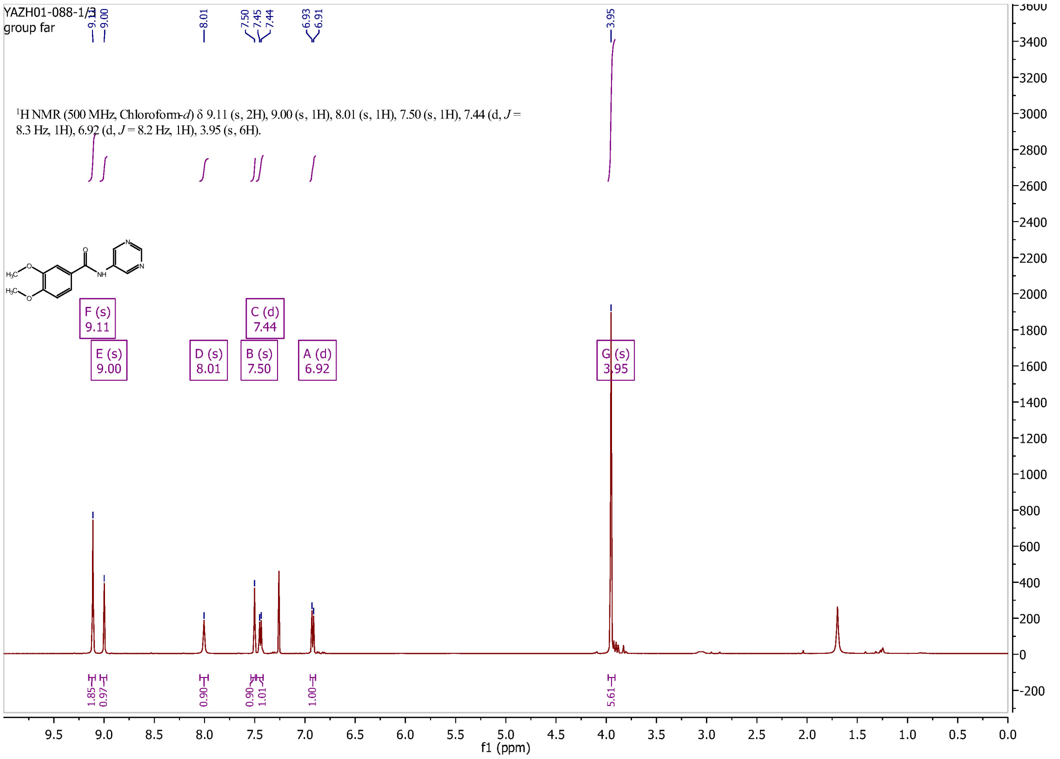

Figure S181 ^{1}H NMR spectrum of compound NPD-3081

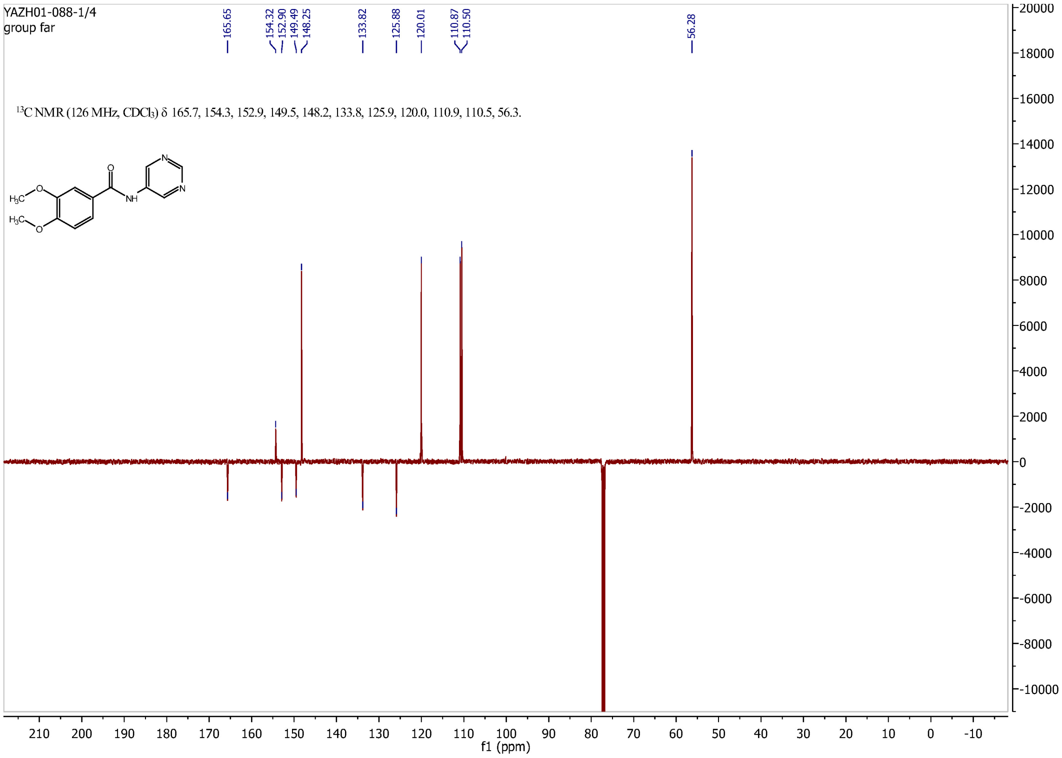

Figure S182 ^{13}C NMR spectrum of compound NPD-3081

Medicinal Chemistry
Vrije Universiteit Amsterdam

Acquired by	: Admin
Date Acquired	: 12/6/2019 11:20:23 AM
Sample Name	: YAZH01-090
Sample ID	:
Tray#	: 1
Vial#	: 8
Injection Volume	: 1
Data File	: C:\LabSolutions\Data\2019\2019-wk24\YAZH01-090.lcd
Background File	: Blanco12062019.lcd
Method File	: Method SCAN ACID standard.lcm
Report Format	: DefaultLCMS.lcr
Tuning File	: C:\LabSolutions\Tuning\Tuning-ESI-pos-neg01072015a.lct
Processed by	: Admin
Modified Date	: 12/6/2019 1:32:15 PM

PDA Graph

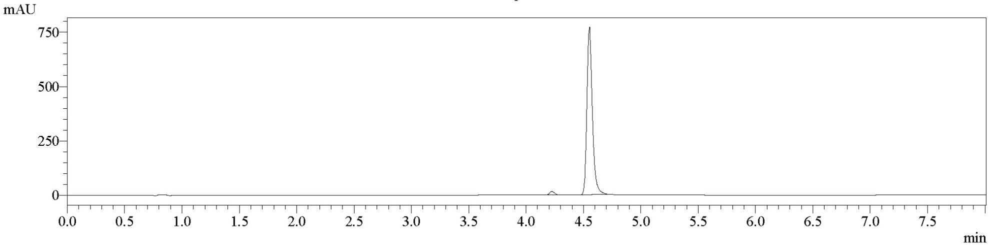

PeakTable

PDA Ch1 254nm 4nm

Peak#	Name	Ret. Time	Area	Area %
1		4.219	49829	1.939
2		4.546	2520313	98.061

MS Chromatogram
YAZH01-090 C:\LabSolutions\Data\2019\2019-wk24\YAZH01-090.lcd

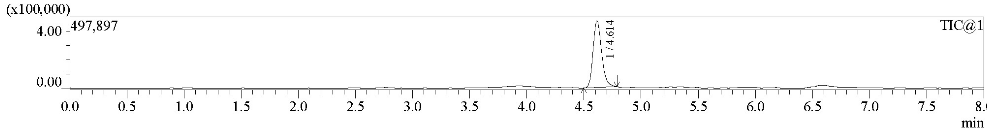

MS Spectrum Graph

#:1 Ret.Time:Averaged 4.600-4.620(Scan#:461-463)
BG Mode:Calc 4.500<->4.790(451<->480)
Mass Peaks:13 Base Peak:390.05(263512) Polarity:Pos Segment1 - Event1

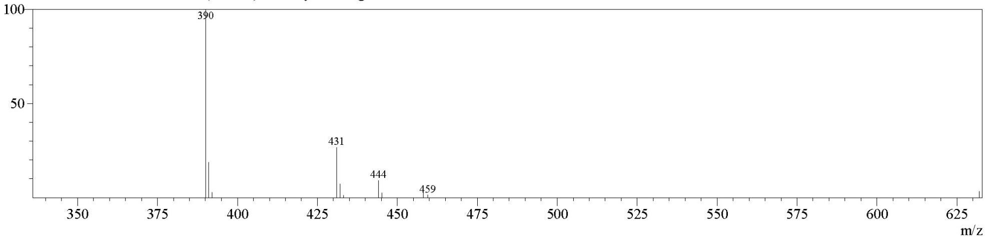

MS Spectrum Table

#:1 Ret.Time:
BG Mode:Calc 4.500<->4.790(451<->480)
Mass Peaks:13 Base Peak:390.05(263512) Polarity:Pos Segment1 - Event1

#	m/z	Abs.Inten.	Rel.Inten.	Charge	Polarity	Monoisotopic
1	336.00	4295	1.63			
2	390.05	263512	100.00			
3	391.05	49449	18.77			
4	392.15	6842	2.60			
5	431.05	70092	26.60			
6	432.10	18744	7.11			
7	433.10	3133	1.19			
8	444.15	23916	9.08			
9	445.20	6710	2.55			
10	458.15	8657	3.29			
11	459.50	3424	1.30			
12	631.95	8285	3.14			
13	632.90	2853	1.08			

Figure S183 LCMS spectrum of compound NPD-3083

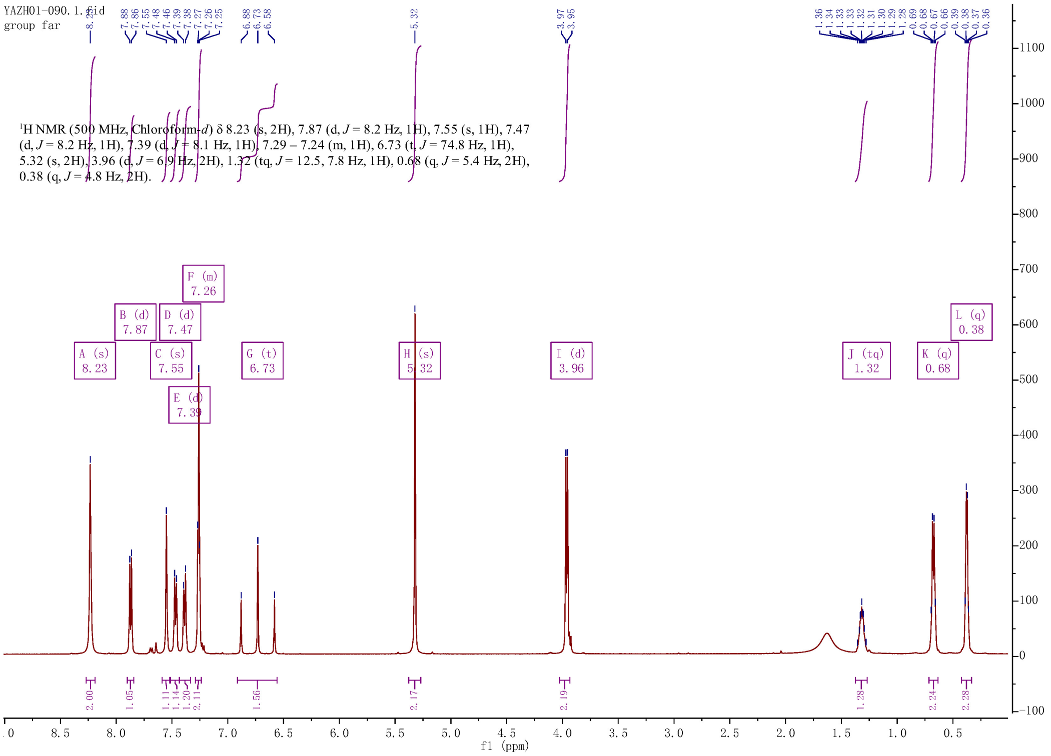

Figure S184 ^{1}H NMR spectrum of compound NPD-3083

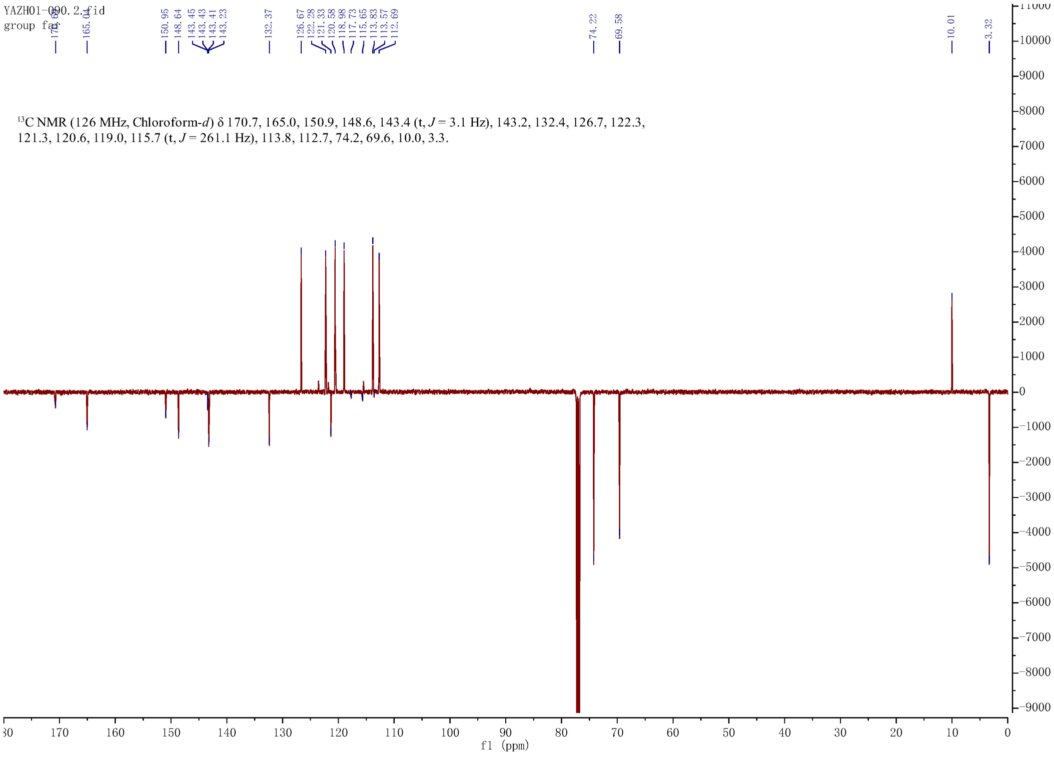

Figure S185 ^{13}C NMR spectrum of compound NPD-3083

Acquired by	: Admin
Date Acquired	: 5/16/2017 4:29:57 PM
Sample Name	: YAZH01-092
Sample ID	:
Tray#	: 1
Vial#	: 47
Injection Volume	: 1
Data File	: C:\LabSolutions\Data\2017\2017-wk20\YAZH01-092.lcd
Background File	: blanco acid 16052017.lcd
Method File	: Method SCAN ACID standard.lcm
Report Format	: DefaultLCMS.lcr
Tuning File	: C:\LabSolutions\Tuning\Tuning-ESI-pos-neg01072015a.lct
Processed by	: Admin
Modified Date	: 5/16/2017 4:59:33 PM

PDA Graph

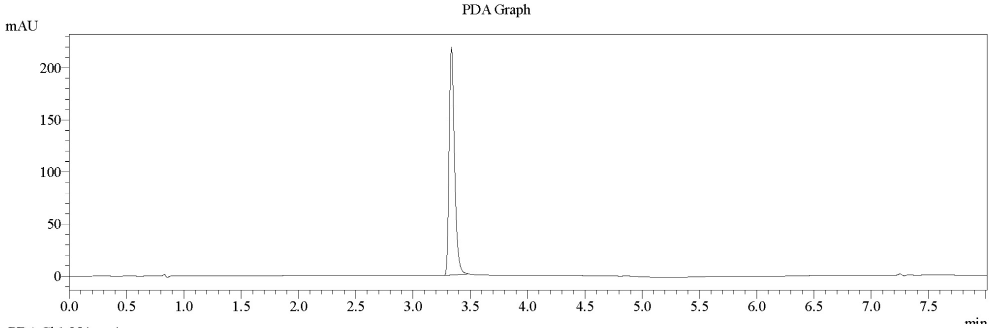

PDA Ch1 254nm 4nm

Peak#	Name	Ret. Time	Area	Area %
1		3.330	718824	100.000

MS Spectrum Graph

#:1 Ret.Time:Averaged 3.380-3.400(Scan#:339-341)
Mass Peaks:8 Base Peak:263.05(235116) Polarity:Pos Segment1 - Event1

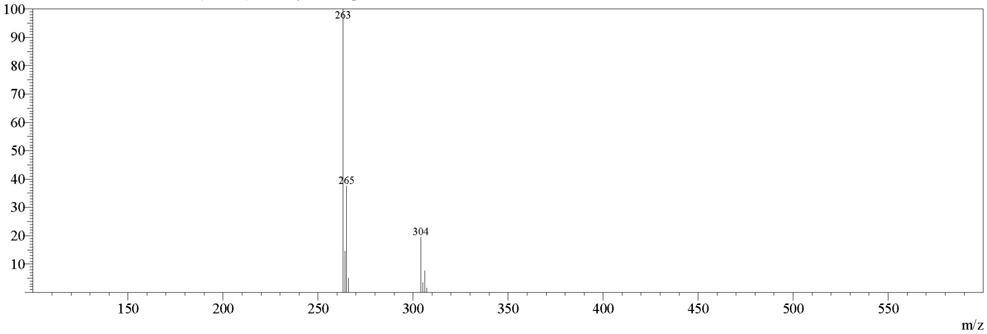

MS Spectrum Table

#:1 Ret.Time:
BG Mode:Calc 3.280<->3.540(329<->355)
Mass Peaks:8 Base Peak:263.05(235116) Polarity:Pos Segment1 - Event1

#	m/z	Abs.Inten.	Rel.Inten.	Charge	Polarity	Monoisotopic
1	263.05	235116	100.00			
2	264.05	34390	14.63			
3	265.05	88056	37.45			
4	266.00	11890	5.06			
5	304.05	45539	19.37			
6	305.05	8033	3.42			
7	306.05	17888	7.61			
8	307.10	3432	1.46			

Figure S186 LCMS spectrum of compound NPD-3085

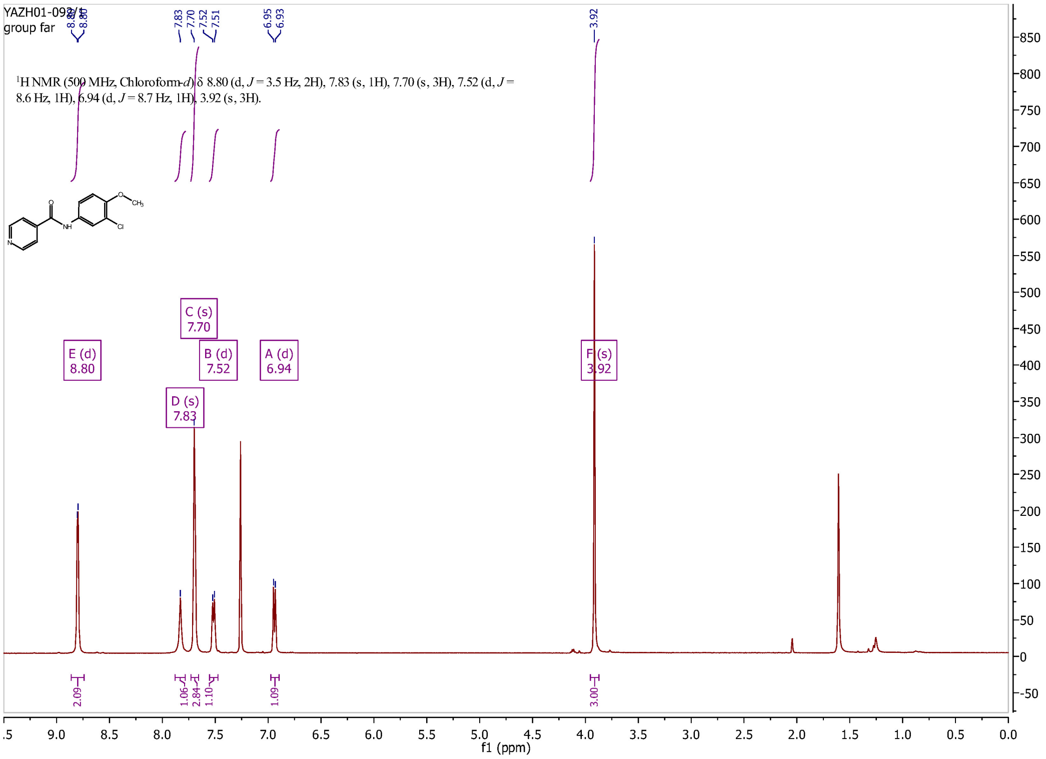

Figure S187 ^{1}H NMR spectrum of compound NPD-3085

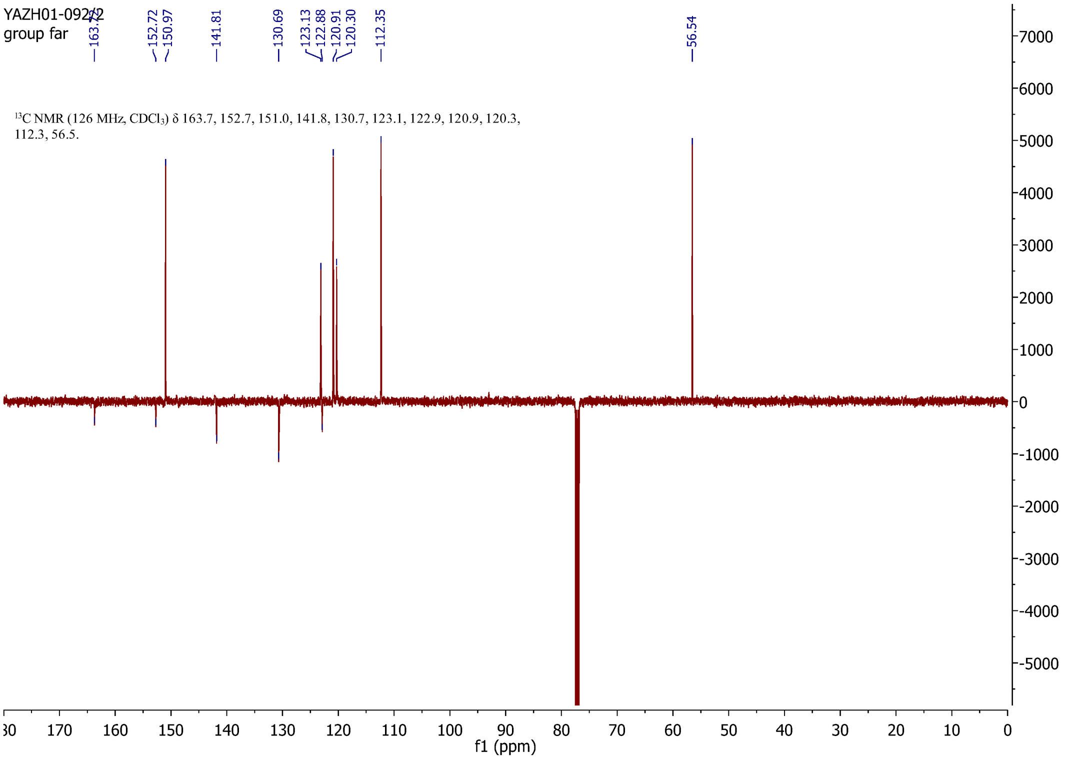

Figure S188 ^{13}C NMR spectrum of compound NPD-3085

Medicinal Chemistry
Vrije Universiteit Amsterdam

Acquired by	: Admin
Date Acquired	: 5/17/2017 9:12:22 AM
Sample Name	: YAZH01-093
Sample ID	:
Tray#	: 1
Vial#	: 11
Injection Volume	: 1
Data File	: C:\LabSolutions\Data\2017\2017-wk20\YAZH01-093.lcd
Background File	: blanco17052017.lcd
Method File	: Method SCAN ACID standard.lcm
Report Format	: DefaultLCMS.lcr
Tuning File	: C:\LabSolutions\Tuning\Tuning-ESI-pos-neg01072015a.lct
Processed by	: Admin
Modified Date	: 5/17/2017 9:42:43 AM

PDA Graph

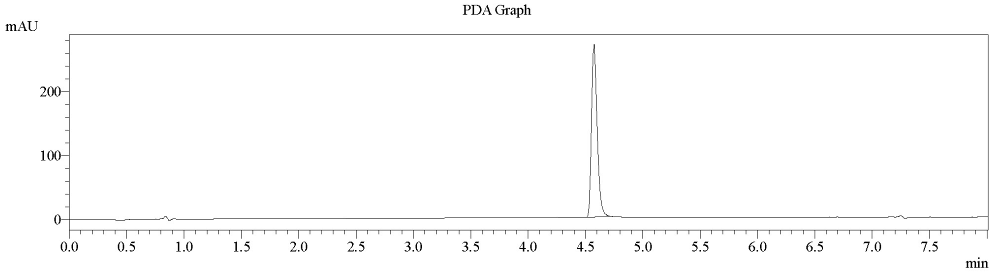

PeakTable

PDA Ch1 254nm 4nm

Peak#	Name	Ret. Time	Area	Area %
1		4.569	921148	100.000

MS Chromatogram
YAZH01-093 C:\LabSolutions\Data\2017\2017-wk20\YAZH01-093.lcd

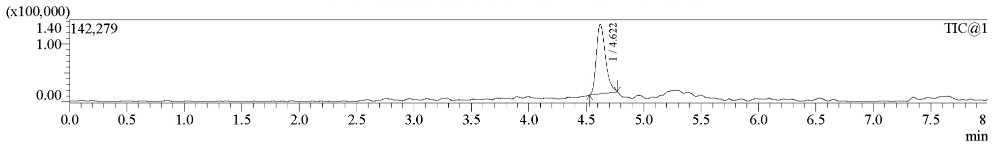

MS Spectrum Graph

#:1 Ret.Time:Averaged 4.610-4.630(Scan#:462-464)
Mass Peaks:19 Base Peak:328.10(67364) Polarity:Pos Segment1 - Event1

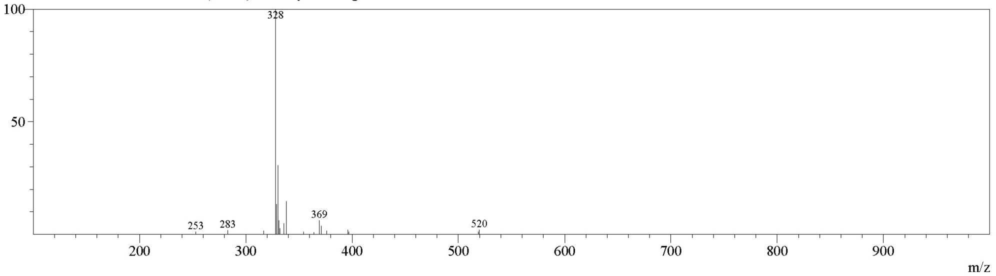

MS Spectrum Table

#:1 Ret.Time:
BG Mode:Calc 4.530<->4.770(454<->478)
Mass Peaks:19 Base Peak:328.10(67364) Polarity:Pos Segment1 - Event1

#	m/z	Abs.Inten.	Rel.Inten.	Charge	Polarity	Monoisotopic
1	252.85	787	1.17			
2	282.85	1206	1.79			
3	316.80	1036	1.54			
4	328.10	67364	100.00			
5	329.10	8988	13.34			
6	330.10	20637	30.64			
7	331.20	4201	6.24			
8	332.30	1871	2.78			
9	336.00	3207	4.76			
10	338.25	9871	14.65			
11	354.25	850	1.26			
12	364.25	689	1.02			
13	369.25	4189	6.22			
14	371.15	2563	3.80			
15	376.20	1150	1.71			
16	396.20	1321	1.96			
17	397.05	737	1.09			
18	518.95	727	1.08			

Figure S189 LCMS spectrum of compound NPD-3086

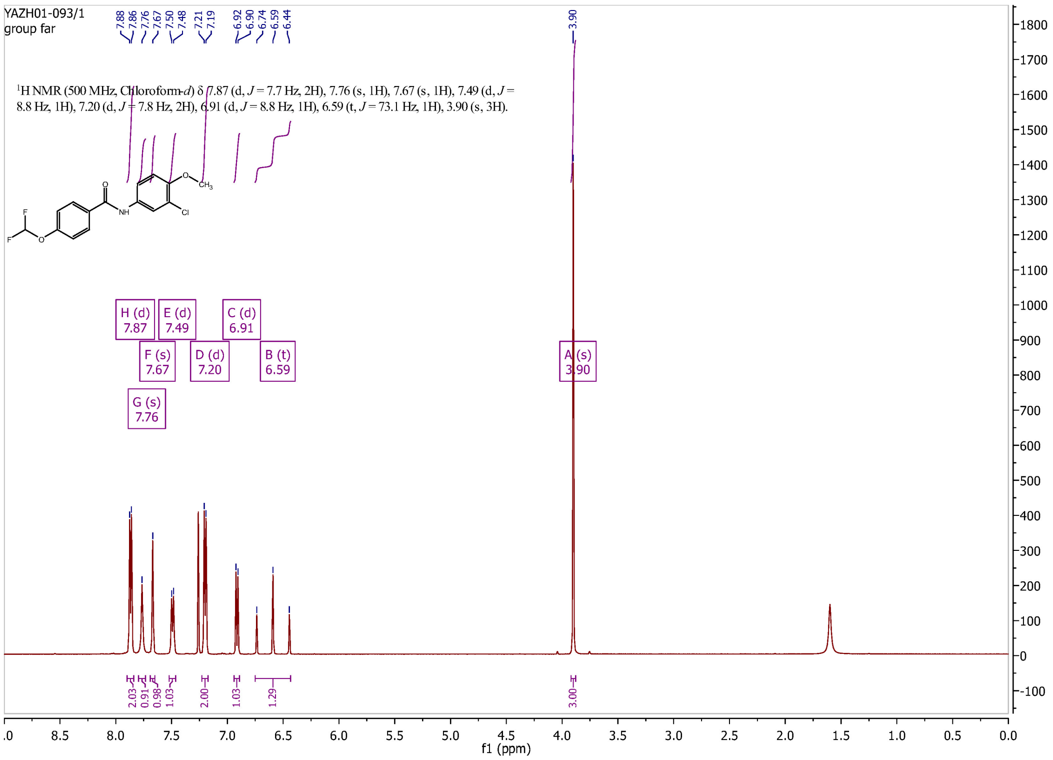

Figure S190 ^{1}H NMR spectrum of compound NPD-3086

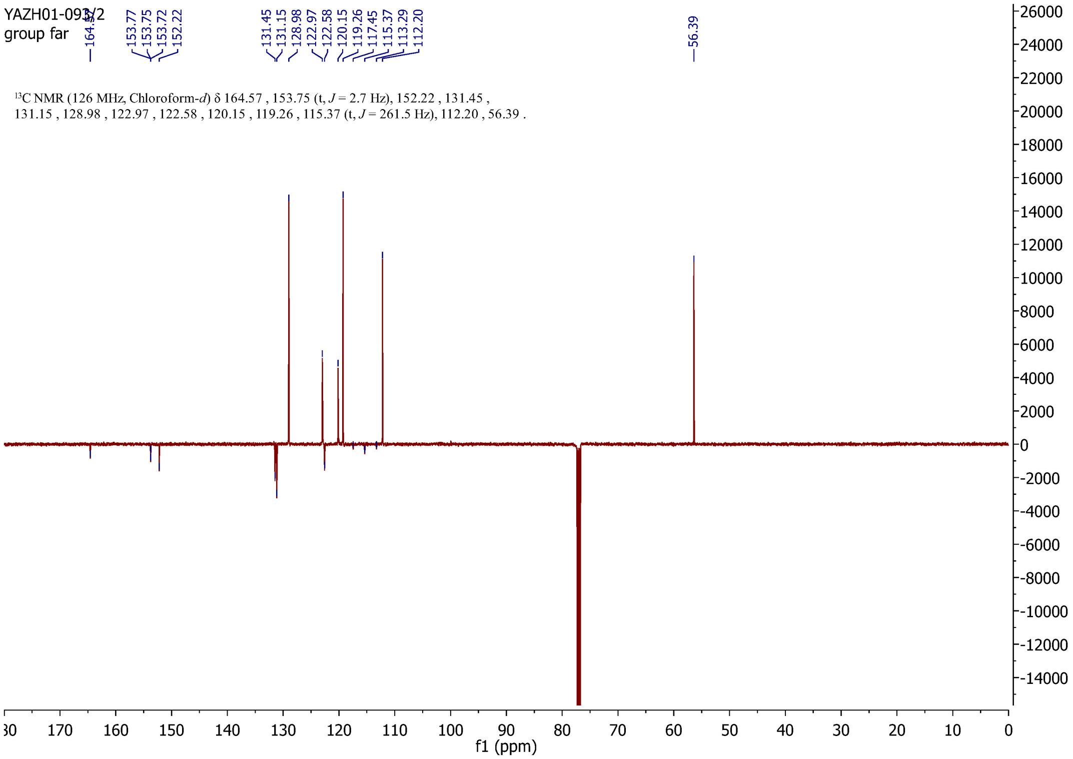

Figure S191 ^{13}C NMR spectrum of compound NPD-3086

Medicinal Chemistry
Vrije Universiteit Amsterdam

Acquired by	: Admin
Date Acquired	: 6/12/2017 11:36:26 AM
Sample Name	: YAZH01-106
Sample ID	:
Tray#	: 1
Vial#	: 23
Injection Volume	: 5
Data File	: C:\LabSolutions\Data\2017\2017-wk24\YAZH01-106.lcd
Background File	: blanco 120617.lcd
Method File	: Method SCAN ACID standard.lcm
Report Format	: DefaultLCMS.lcr
Tuning File	: C:\LabSolutions\Tuning\Tuning-ESI-pos-neg01072015a.lct
Processed by	: Admin
Modified Date	: 6/12/2017 12:11:03 PM

Chromatogram
YAZH01-106 C:\LabSolutions\Data\2017\2017-wk24\YAZH01-106.lcd

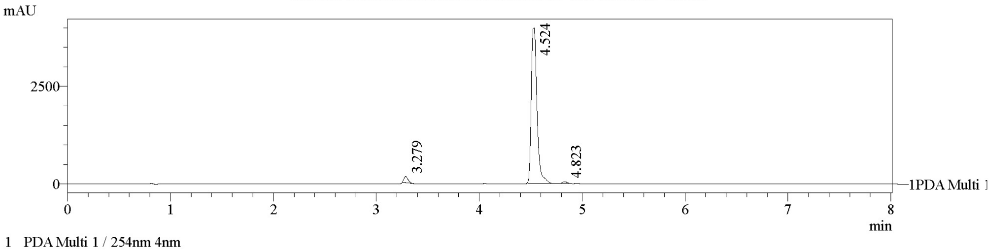

1 PDA Multi 1 / 254nm 4nm

PeakTable

PDA Ch1 254nm 4nm

Peak#	Name	Ret. Time	Area	Area %
1		3.279	415054	2.652
2		4.524	15085442	96.406
3		4.823	147330	0.942

MS Chromatogram
YAZH01-106 C:\LabSolutions\Data\2017\2017-wk24\YAZH01-106.lcd

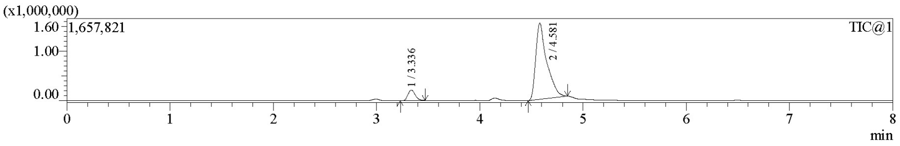

MS Spectrum Graph

#:1 Ret.Time:Averaged 4.570-4.590(Scan#:458-460)
Mass Peaks:14 Base Peak:355.05(650409) Polarity:Pos Segment1 - Event1

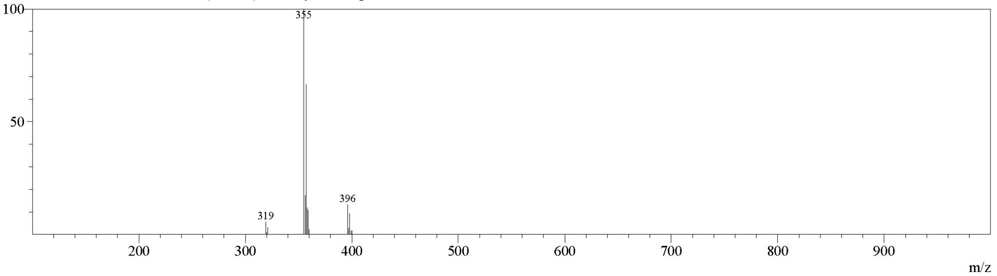

MS Spectrum Table

#:1 Ret.Time:
BG Mode:Calc 4.470<->4.850(448<->486)
Mass Peaks:14 Base Peak:355.05(650409) Polarity:Pos Segment1 - Event1

#	m/z	Abs.Inten.	Rel.Inten.	Charge	Polarity	Monoisotopic
1	319.00	36020	5.54			
2	320.10	6725	1.03			
3	321.10	19904	3.06			
4	355.05	650409	100.00			
5	356.05	112147	17.24			
6	357.05	432794	66.54			
7	358.05	77271	11.88			

#	m/z	Abs.Inten.	Rel.Inten.	Charge	Polarity	Monoisotopic
8	359.00	69909	10.75			
9	360.10	14187	2.18			
10	396.05	85324	13.12			
11	397.05	17455	2.68			
12	398.10	60249	9.26			
13	399.15	10412	1.60			
14	400.10	11893	1.83			

Figure S192 LCMS spectrum of compound NPD-3093

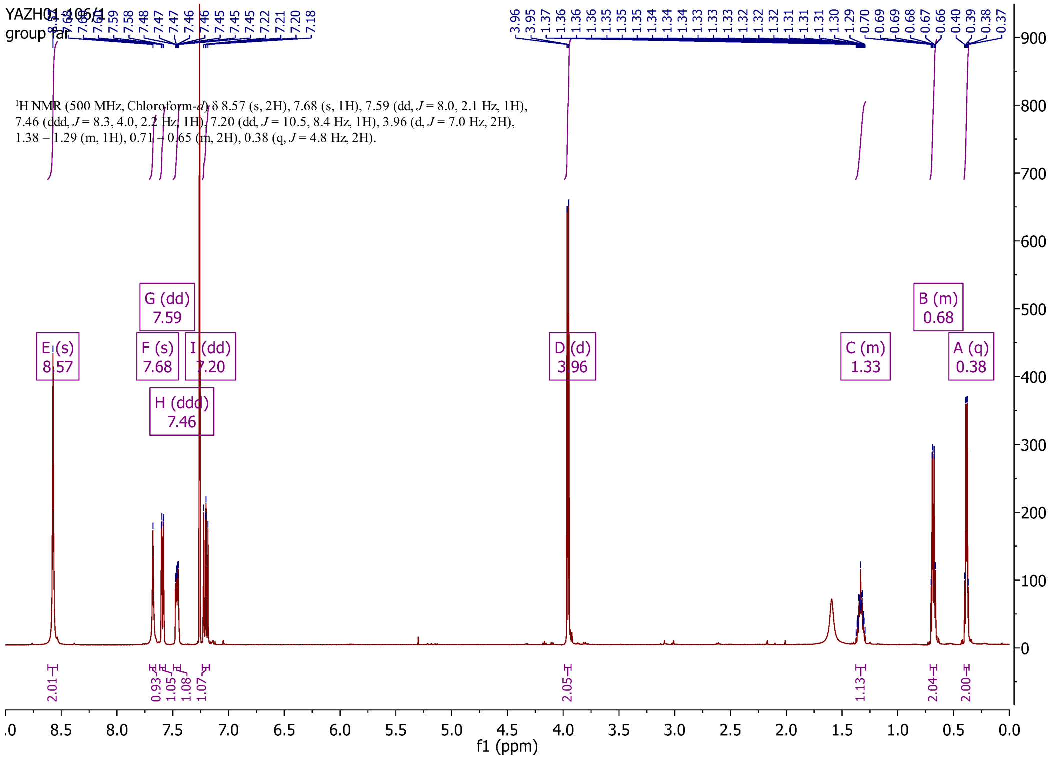

Figure S193 ^{1}H NMR spectrum of compound NPD-3093

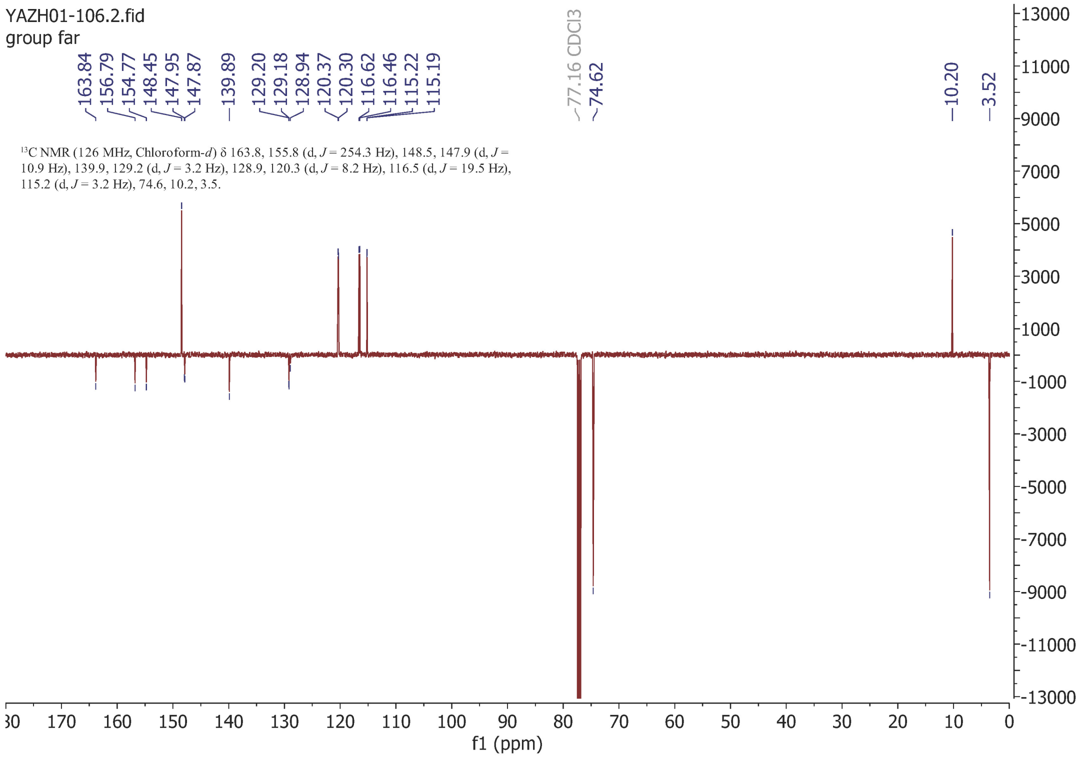

Figure S194 ^{13}C NMR spectrum of compound NPD-3093

Medicinal Chemistry
Vrije Universiteit Amsterdam

Acquired by	: Admin
Date Acquired	: 6/13/2017 11:04:21 AM
Sample Name	: YAZH01-107-17
Sample ID	:
Tray#	: 1
Vial#	: 16
Injection Volume	: 5
Data File	: C:\LabSolutions\Data\2017\2017-wk24\YAZH01-107-17.lcd
Background File	: blanco 130617.lcd
Method File	: Method SCAN ACID standard.lcm
Report Format	: DefaultLCMS.lcr
Tuning File	: C:\LabSolutions\Tuning\Tuning-ESI-pos-neg01072015a.lct
Processed by	: Admin
Modified Date	: 6/13/2017 1:43:07 PM

Chromatogram
YAZH01-107-17 C:\LabSolutions\Data\2017\2017-wk24\YAZH01-107-17.lcd

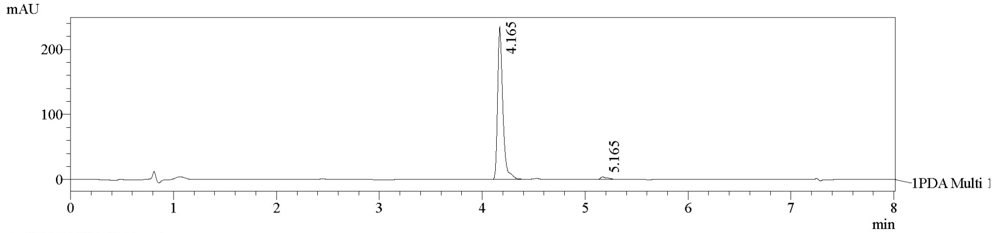

1 PDA Multi 1 / 254nm 4nm

PeakTable

PDA Ch1 254nm 4nm

Peak#	Name	Ret. Time	Area	Area %
1		4.165	824927	97.855
2		5.165	18083	2.145

MS Chromatogram
YAZH01-107-17 C:\LabSolutions\Data\2017\2017-wk24\YAZH01-107-17.lcd

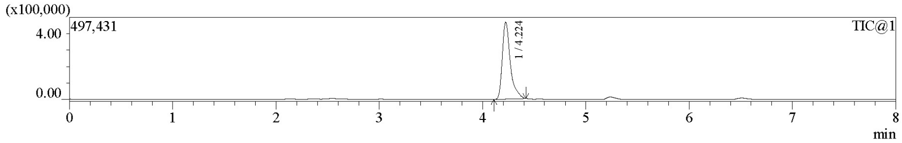

MS Spectrum Graph

#:1 Ret.Time:Averaged 4.210-4.230(Scan#:422-424)
Mass Peaks:17 Base Peak:367.05(201830) Polarity:Pos Segment1 - Event1

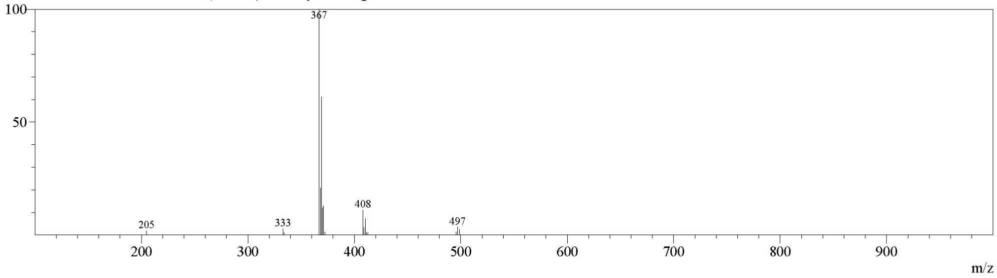

MS Spectrum Table

#:1 Ret.Time:
BG Mode:Calc 4.110<->4.420(412<->443)
Mass Peaks:17 Base Peak:367.05(201830) Polarity:Pos Segment1 - Event1

#	m/z	Abs.Inten.	Rel.Inten.	Charge	Polarity	Monoisotopic
1	204.95	3679	1.82			
2	333.10	5364	2.66			
3	334.10	2122	1.05			
4	367.05	201830	100.00			
5	368.10	42127	20.87			
6	369.05	123618	61.25			
7	370.05	24134	11.96			
8	371.00	25963	12.86			
9	372.20	2827	1.40			
10	408.15	22053	10.93			
11	409.15	6829	3.38			
12	410.25	14735	7.30			
13	411.10	2372	1.18			
14	412.55	2504	1.24			
15	495.45	2906	1.44			
16	496.95	7050	3.49			

Figure S195 LCMS spectrum of compound NPD-3094

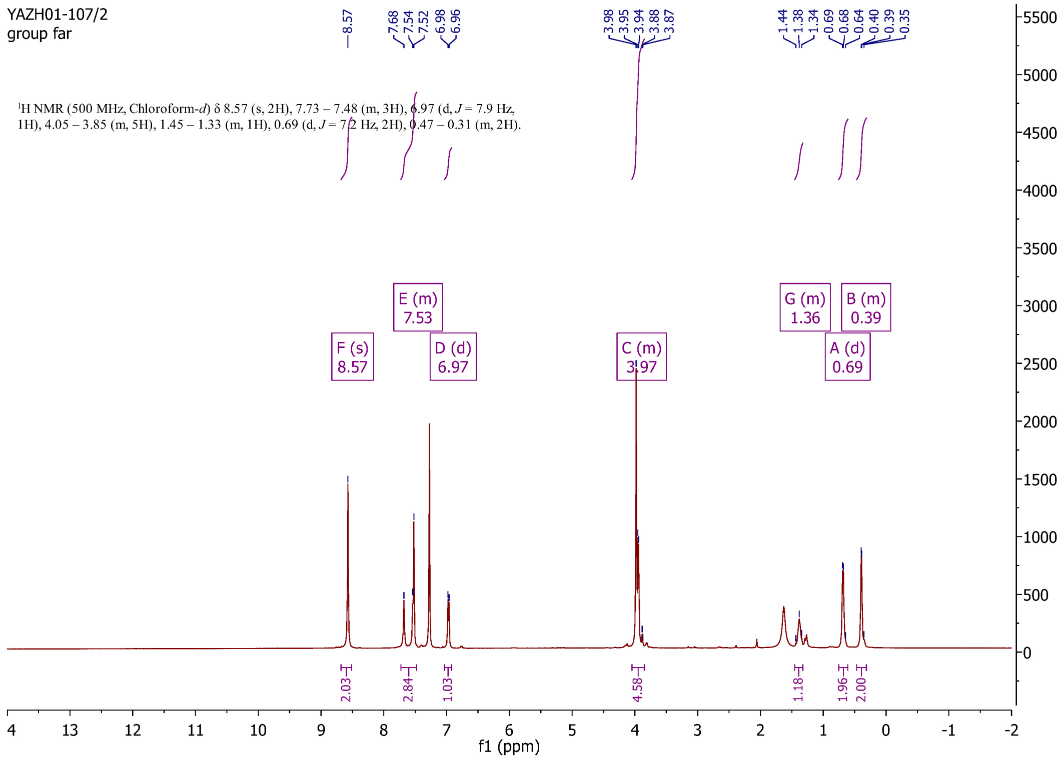

Figure S196 ^{1}H NMR spectrum of compound NPD-3094

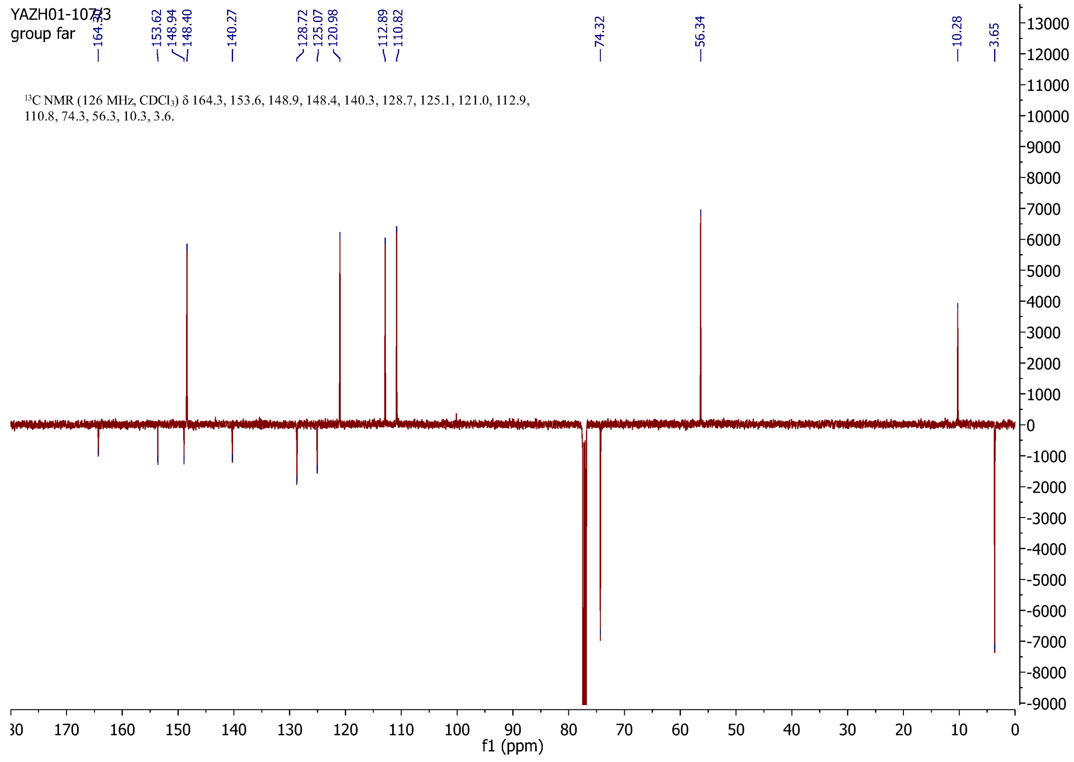

Figure S197 ^{13}C NMR spectrum of compound NPD-3094

Acquired by	: Admin
Date Acquired	: 6/13/2017 4:46:24 PM
Sample Name	: YAZH01-110
Sample ID	:
Tray#	: 1
Vial#	: 37
Injection Volume	: 1
Data File	: C:\LabSolutions\Data\2017\2017-wk24\YAZH01-110.lcd
Background File	: blanco 130617.lcd
Method File	: Method SCAN ACID standard.lcm
Report Format	: DefaultLCMS.lcr
Tuning File	: C:\LabSolutions\Tuning\Tuning-ESI-pos-neg01072015a.lct
Processed by	: Admin
Modified Date	: 6/14/2017 1:19:38 PM

PDA Graph

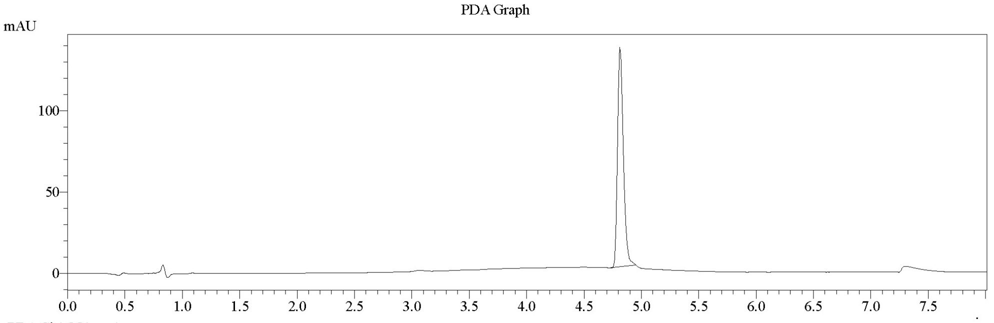

PDA Ch1 254nm 4nm

Peak#	Name	Ret. Time	Area	Area %
1		4.809	471773	100.000

MS Spectrum Graph

#:1 Ret.Time:Averaged 4.860-4.880(Scan#:487-489)
Mass Peaks:23 Base Peak:371.00(61613) Polarity:Pos Segment1 - Event1

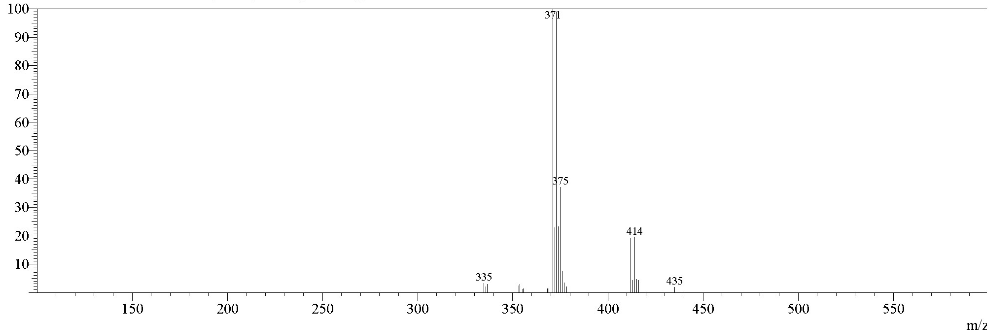

MS Spectrum Table

#:1 Ret.Time:
BG Mode:Calc 4.770<->5.050(478<->506)
Mass Peaks:23 Base Peak:371.00(61613) Polarity:Pos Segment1 - Event1

#	m/z	Abs.Inten.	Rel.Inten.	Charge	Polarity	Monoisotopic
1	334.75	2002	3.25			
2	335.75	1222	1.98			
3	336.75	1765	2.86			
4	353.15	1447	2.35			
5	353.70	1783	2.89			
6	355.20	672	1.09			
7	355.70	832	1.35			
8	368.15	815	1.32			
9	369.10	796	1.29			
10	371.00	61613	100.00			
11	372.05	14081	22.85			
12	373.00	61021	99.04			
13	374.00	14328	23.25			
14	375.05	22855	37.09			
15	376.05	4665	7.57			
16	376.95	2130	3.46			
17	378.20	1272	2.06			
18	411.95	11738	19.05			
19	413.15	2627	4.26			
20	413.95	12012	19.50			
21	415.10	2901	4.71			
22	416.05	2677	4.34			
23	435.00	1176	1.91			

Figure S198 LCMS spectrum of compound NPD-3096

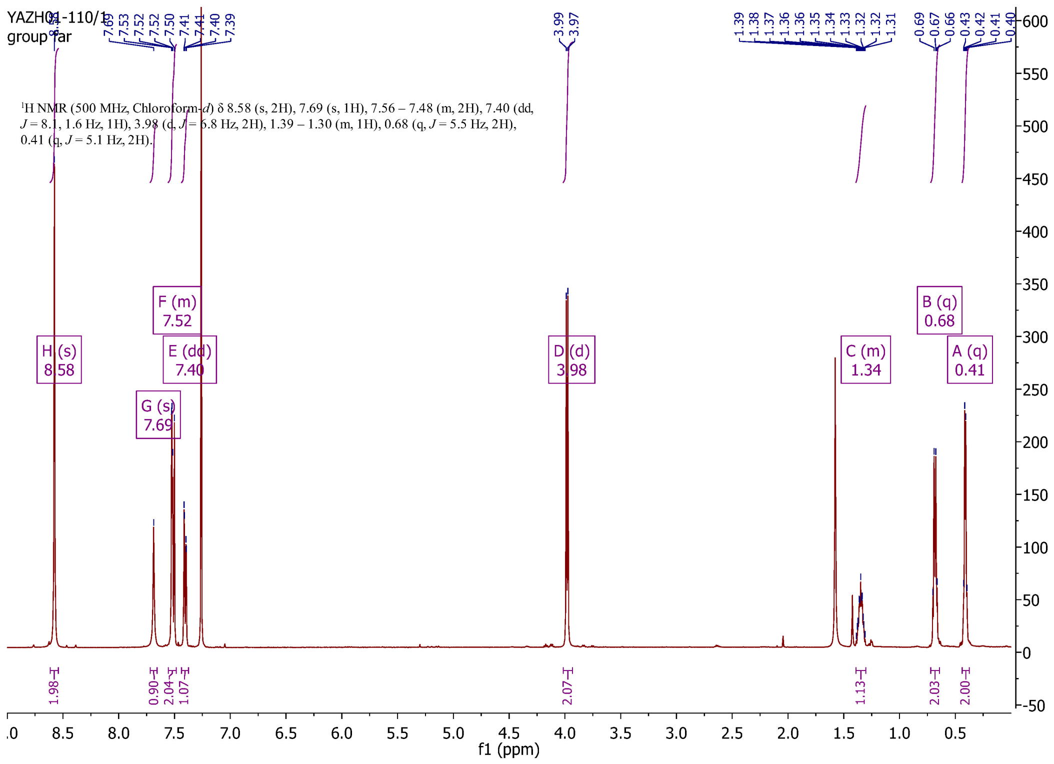

Figure S199 ^{1}H NMR spectrum of compound NPD-3096

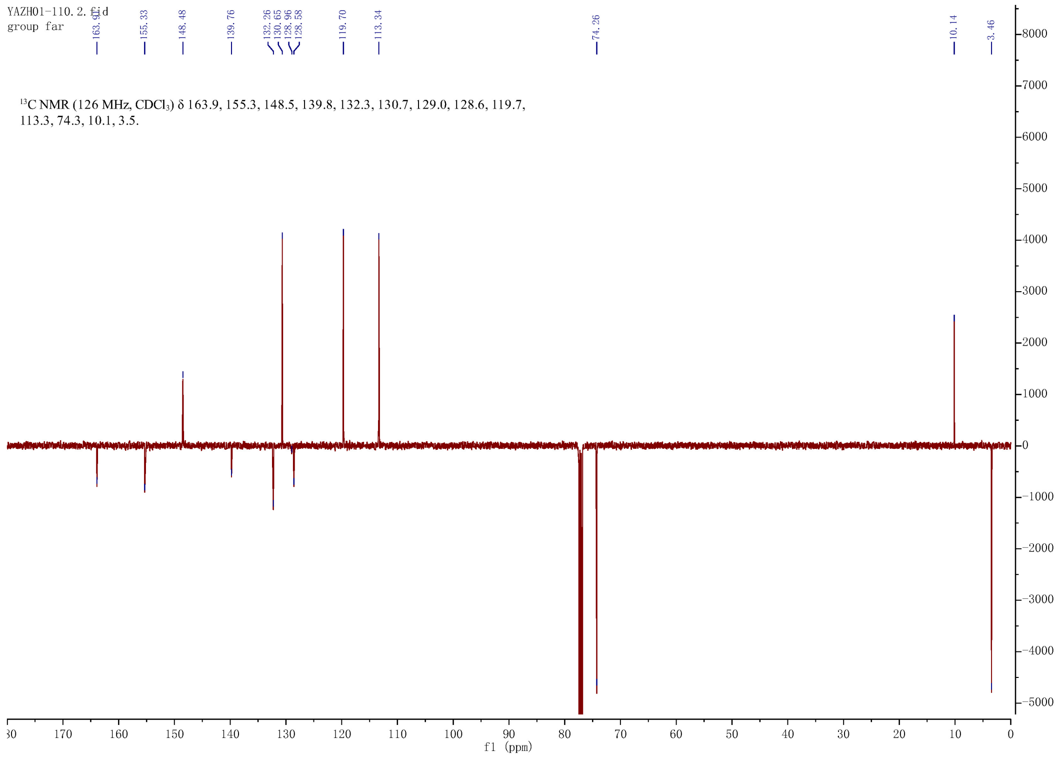

Figure S200 ^{13}C NMR spectrum of compound NPD-3096

Acquired by	: Admin
Date Acquired	: 19/2/2018 2:18:26 PM
Sample Name	: PC-149
Sample ID	:
Tray#	: 1
Vial#	: 35
Injection Volume	: 1
Data File	: C:\LabSolutions\Data\2018\2018-wk08\PC-149.lcd
Background File	: blanco 19022018.lcd
Method File	: Method SCAN ACID standard.lcm
Report Format	: DefaultLCMS.lcr
Tuning File	: C:\LabSolutions\Tuning\Tuning-ESI-pos-neg01072015a.lct
Processed by	: Admin
Modified Date	: 19/2/2018 2:44:57 PM

Medicinal Chemistry
Vrije Universiteit Amsterdam

Chromatogram
PC-149 C:\LabSolutions\Data\2018\2018-wk08\PC-149.lcd

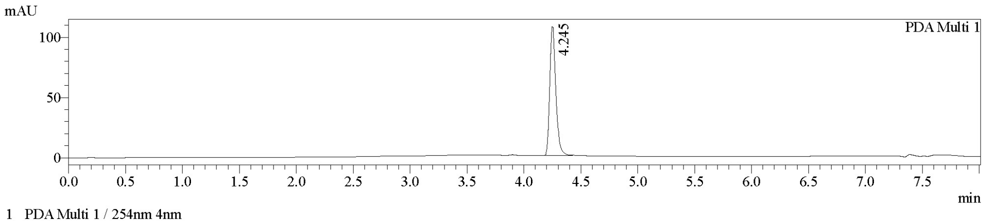

1 PDA Multi 1 / 254nm 4nm

PeakTable

PDA Ch1 254nm 4nm

Peak#	Name	Ret. Time	Area	Area %
1		4.245	396497	100.000

MS Chromatogram
PC-149 C:\LabSolutions\Data\2018\2018-wk08\PC-149.lcd

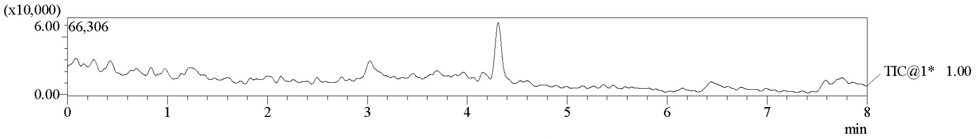

MS Spectrum Graph

#:1 Ret.Time:Single 4.310(Scan#:432)
Mass Peaks:10 Base Peak:291.95(35707) Polarity:Pos Segment1 - Event1

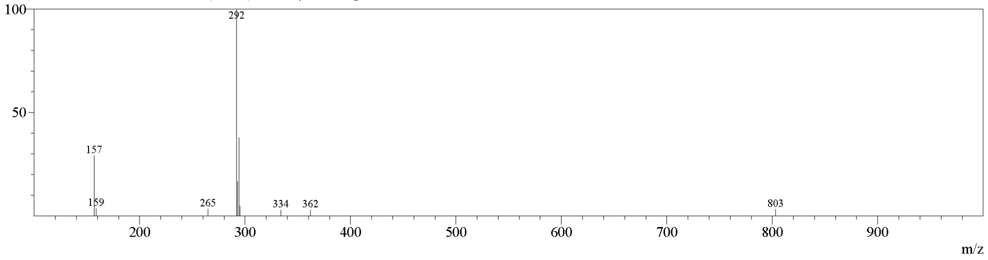

MS Spectrum Table

#:1 Ret.Time:
BG Mode:None
Mass Peaks:10 Base Peak:291.95(35707) Polarity:Pos Segment1 - Event1

#	m/z	Abs.Inten.	Rel.Inten.	Charge	Polarity	Monoisotopic
1	157.05	10374	29.05			
2	158.95	1299	3.64			
3	264.80	1209	3.39			
4	291.95	35707	100.00			
5	293.00	5925	16.59			
6	294.00	13482	37.76			
7	295.15	1720	4.82			
8	334.10	1065	2.98			
9	362.05	1037	2.90			
10	803.25	1159	3.25			

Figure S201 LCMS spectrum of compound NPD-3286

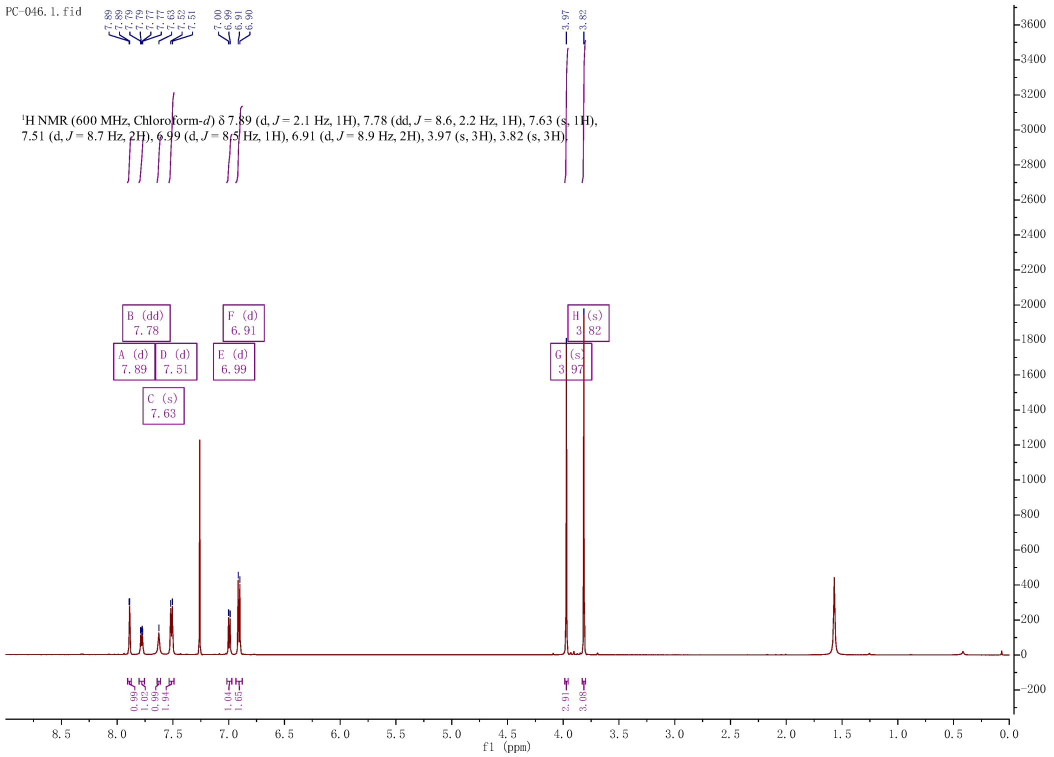

Figure S202 ^{1}H NMR spectrum of compound NPD-3286

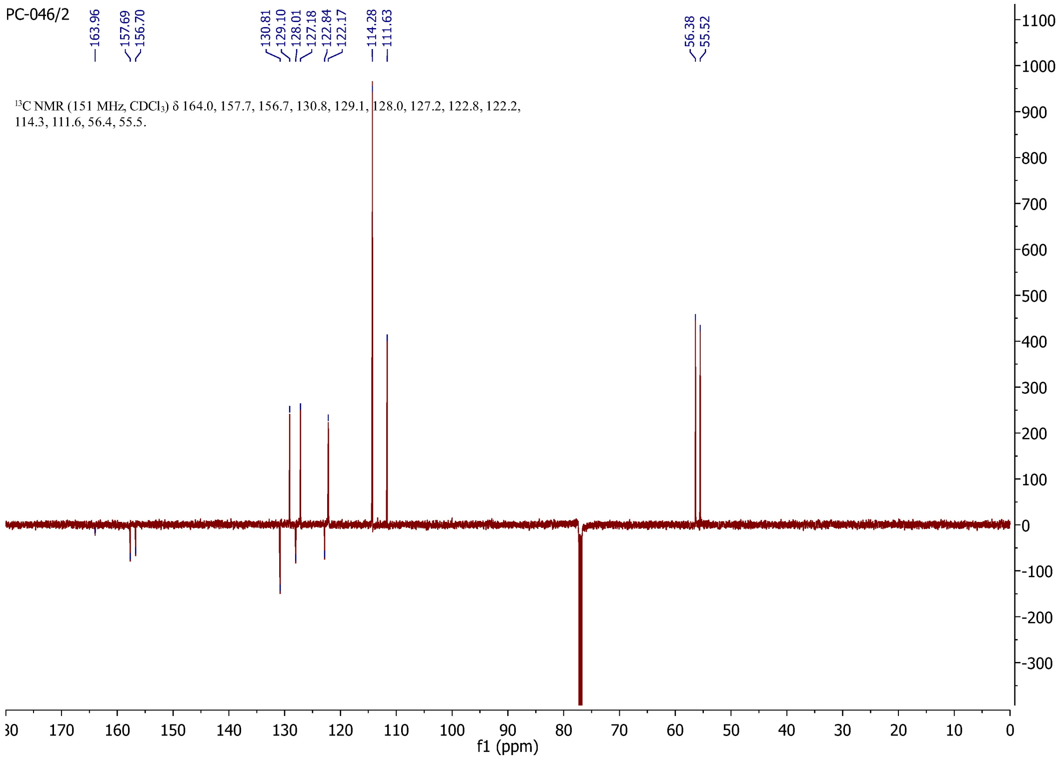

Figure S203 ^{13}C NMR spectrum of compound NPD-3286

Acquired by : Admin
Date Acquired : 19/2/2018 2:37:55 PM
Sample Name : PC-150
Sample ID :
Tray# : 1
Vial# : 36
Injection Volume : 1
Data File : C:\LabSolutions\Data\2018\2018-wk08\PC-150.lcd
Background File : blanco 19022018.lcd
Method File : Method SCAN ACID standard.lcm
Report Format : DefaultLCMS.lcr
Tuning File : C:\LabSolutions\Tuning\Tuning-ESI-pos-neg01072015a.lct
Processed by : Admin
Modified Date : 1/3/2018 12:46:12 PM

Medicinal Chemistry
Vrije Universiteit Amsterdam

Chromatogram
PC-150 C:\LabSolutions\Data\2018\2018-wk08\PC-150.lcd

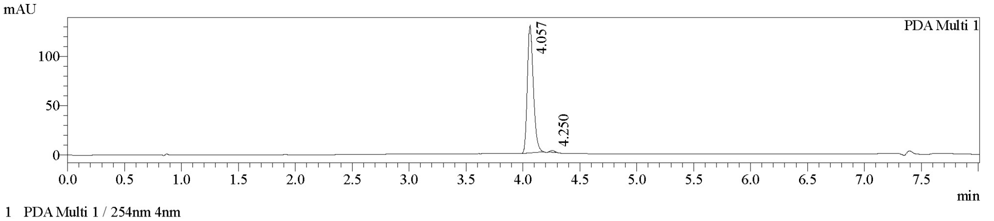

1 PDA Multi 1 / 254nm 4nm

PeakTable

PDA Ch1 254nm 4nm

Peak#	Name	Ret. Time	Area	Area %
1		4.057	468487	99.018
2		4.250	4647	0.982

MS Chromatogram
PC-150 C:\LabSolutions\Data\2018\2018-wk08\PC-150.lcd

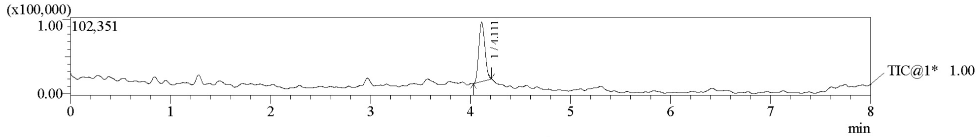

MS Spectrum Graph
#:1 Ret.Time:Averaged 4.100-4.120(Scan#:411-413)
Mass Peaks:7 Base Peak:293.00(53322) Polarity:Pos Segment1 - Event1

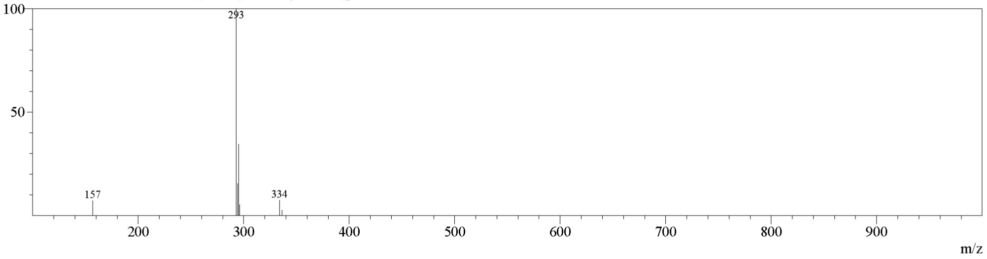

MS Spectrum Table
#:1 Ret.Time:
BG Mode:Calc 4.030<->4.210(404<->422)
Mass Peaks:7 Base Peak:293.00(53322) Polarity:Pos Segment1 - Event1

#	m/z	Abs.Inten.	Rel.Inten.	Charge	Polarity	Monoisotopic
1	157.00	3767	7.06			
2	293.00	53322	100.00			
3	294.05	8219	15.41			
4	295.00	18471	34.64			
5	296.10	2842	5.33			
6	333.90	3900	7.31			
7	336.10	1452	2.72			

Figure S204 LCMS spectrum of compound NPD-3287

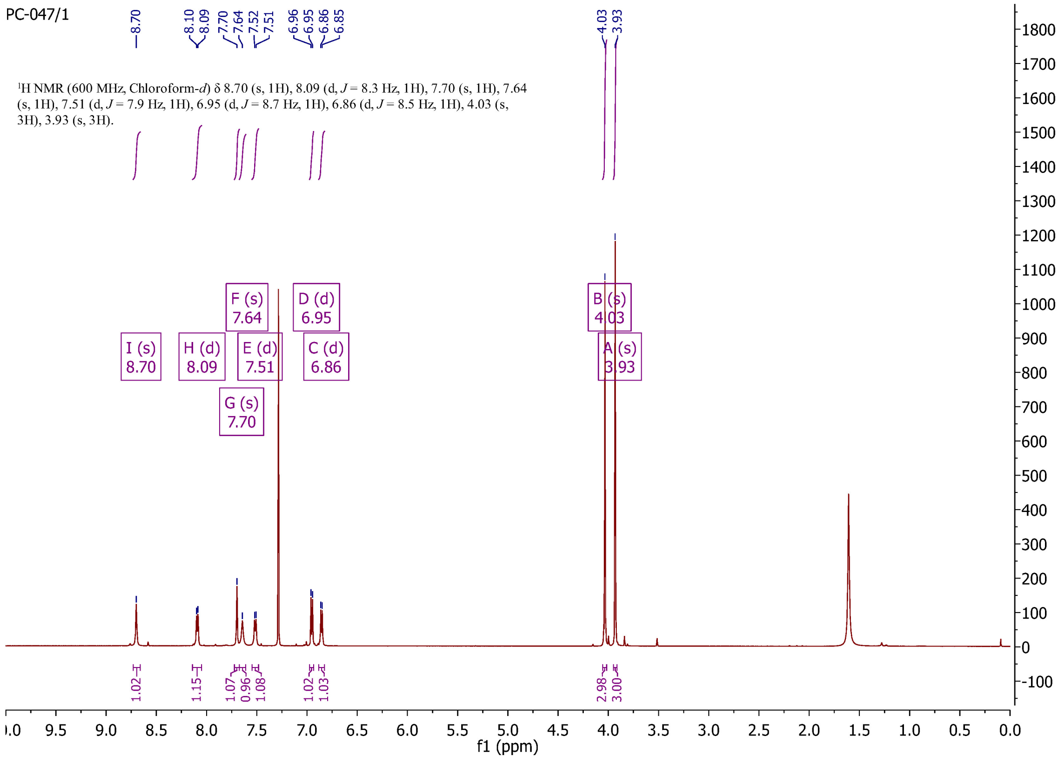

Figure S205 ^{1}H NMR spectrum of compound NPD-3287

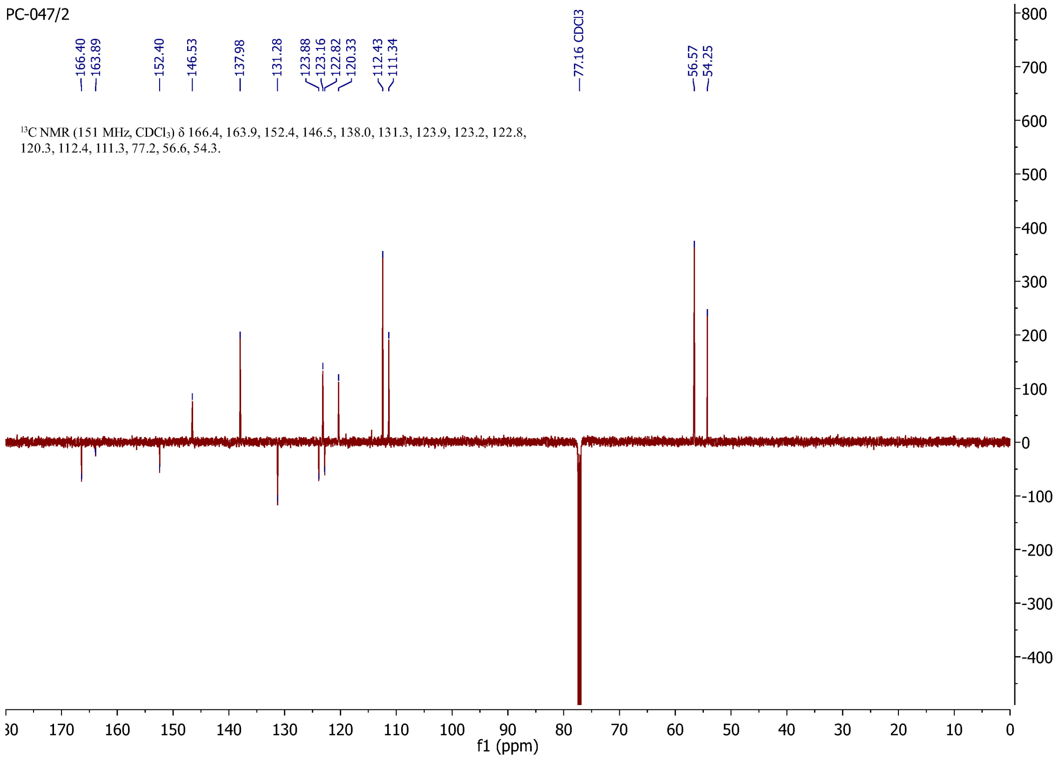

Figure S206 ^{13}C NMR spectrum of compound NPD-3287

Medicinal Chemistry
Vrije Universiteit Amsterdam

Acquired by	: Admin
Date Acquired	: 19/2/2018 2:46:34 PM
Sample Name	: PC-151
Sample ID	:
Tray#	: 1
Vial#	: 37
Injection Volume	: 1
Data File	: C:\LabSolutions\Data\2018\2018-wk08\PC-151.lcd
Background File	: blanco 19022018.lcd
Method File	: Method SCAN ACID standard.lcm
Report Format	: DefaultLCMS.lcr
Tuning File	: C:\LabSolutions\Tuning\Tuning-ESI-pos-neg01072015a.lct
Processed by	: Admin
Modified Date	: 19/2/2018 5:31:36 PM

Chromatogram
PC-151 C:\LabSolutions\Data\2018\2018-wk08\PC-151.lcd

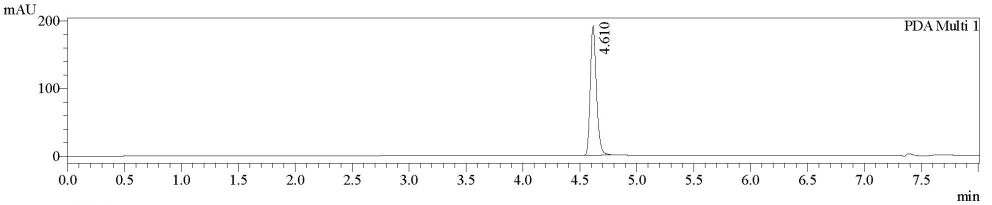

1 PDA Multi 1 / 254nm 4nm

PeakTable

PDA Ch1 254nm 4nm

Peak#	Name	Ret. Time	Area	Area %
1		4.610	714742	100.000

MS Chromatogram
PC-151 C:\LabSolutions\Data\2018\2018-wk08\PC-151.lcd

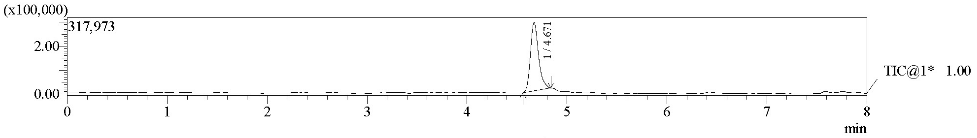

MS Spectrum Graph

#:1 Ret.Time:Averaged 4.660-4.680(Scan#:467-469)
Mass Peaks:10 Base Peak:352.05(177912) Polarity:Pos Segment1 - Event1

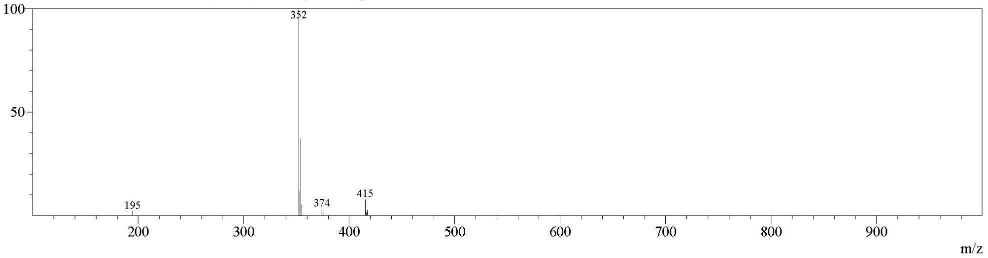

MS Spectrum Table

#:1 Ret.Time:
BG Mode:Calc 4.560<->4.840(457<->485)
Mass Peaks:10 Base Peak:352.05(177912) Polarity:Pos Segment1 - Event1

#	m/z	Abs.Inten.	Rel.Inten.	Charge	Polarity	Monoisotopic
1	194.95	3762	2.11			
2	352.05	177912	100.00			
3	353.10	20562	11.56			
4	354.05	65945	37.07			
5	355.00	9550	5.37			
6	374.00	5700	3.20			
7	376.00	2427	1.36			
8	415.15	13568	7.63			
9	416.15	2698	1.52			
10	417.05	4652	2.61			

Figure S207 LCMS spectrum of compound NPD-3288

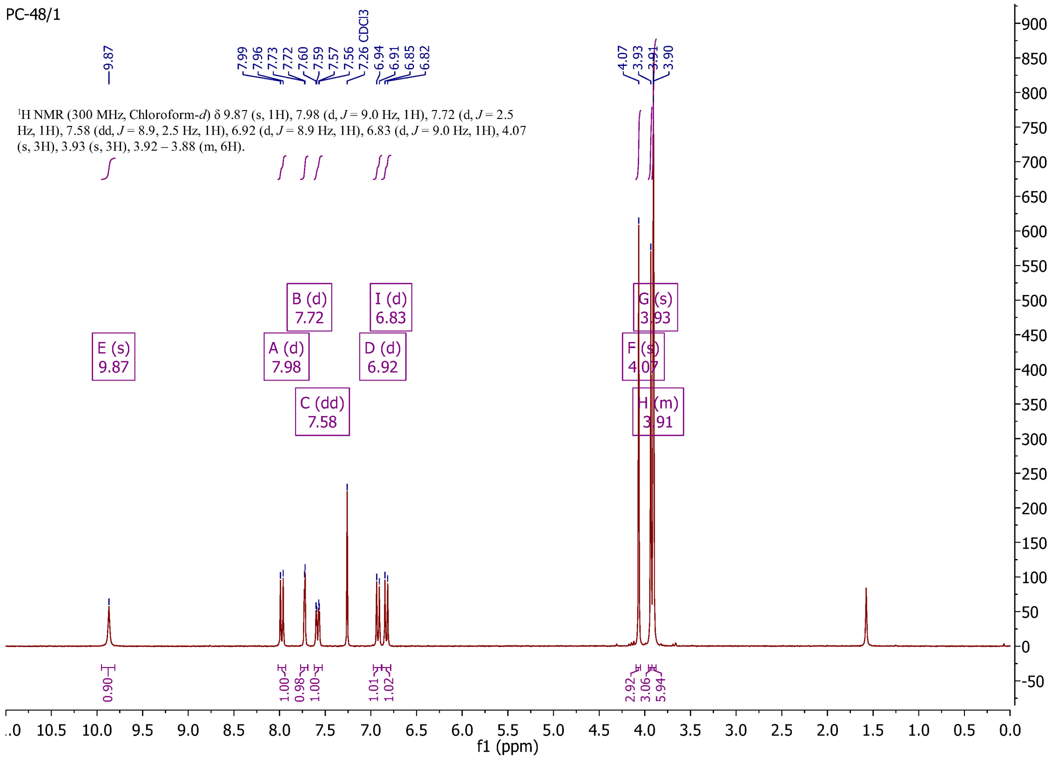

Figure S208 ^{1}H NMR spectrum of compound NPD-3288

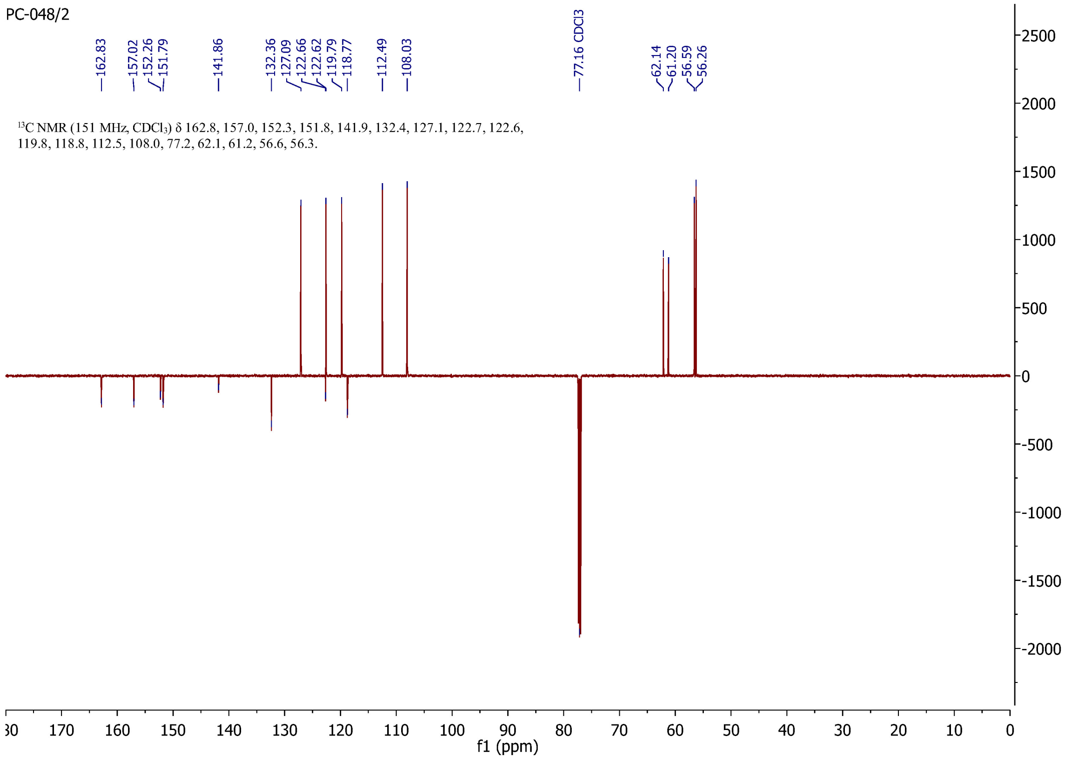

Figure S209 ^{13}C NMR spectrum of compound NPD-3288

Medicinal Chemistry
Vrije Universiteit Amsterdam

Acquired by	: Admin
Date Acquired	: 26/2/2018 10:02:07 AM
Sample Name	: PC-151-Thijs
Sample ID	:
Tray#	: 1
Vial#	: 1
Injection Volume	: 1
Data File	: C:\LabSolutions\Data\2018\2018-wk09\PC-151-Thijs.lcd
Background File	: Blanco_26022018.lcd
Method File	: Method SCAN ACID standard.lcm
Report Format	: DefaultLCMS.lcr
Tuning File	: C:\LabSolutions\Tuning\Tuning-ESI-pos-neg01072015a.lct
Processed by	: Admin
Modified Date	: 26/2/2018 10:30:02 AM

Chromatogram
PC-151-Thijs C:\LabSolutions\Data\2018\2018-wk09\PC-151-Thijs.lcd

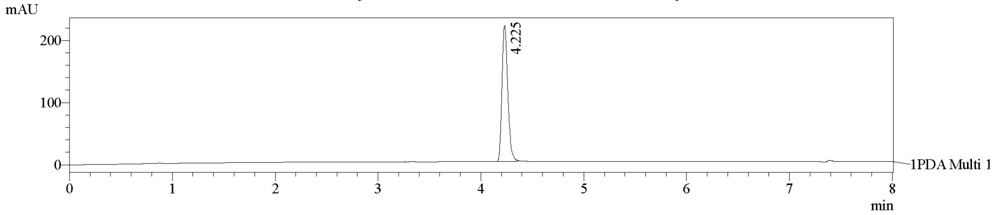

1 PDA Multi 1 / 254nm 4nm
PDA Ch1 254nm 4nm

Peak#	Name	Ret. Time	Area	Area %
1		4.225	851213	100.000

MS Chromatogram
PC-151-Thijs C:\LabSolutions\Data\2018\2018-wk09\PC-151-Thijs.lcd

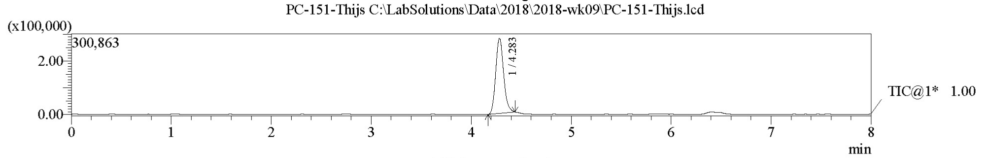

MS Spectrum Graph
#:1 Ret.Time:Averaged 4.270-4.290(Scan#:428-430)
Mass Peaks:6 Base Peak:352.10(192449) Polarity:Pos Segment1 - Event1

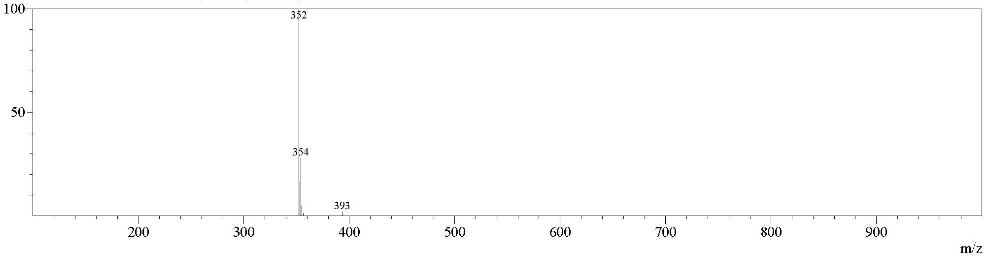

MS Spectrum Table
#:1 Ret.Time:
BG Mode:Calc 4.170<->4.440(418<->445)
Mass Peaks:6 Base Peak:352.10(192449) Polarity:Pos Segment1 - Event1

#	m/z	Abs.Inten.	Rel.Inten.	Charge	Polarity	Monoisotopic
1	352.10	192449	100.00			
2	353.05	31737	16.49			
3	354.05	53672	27.89			
4	355.10	9257	4.81			
5	356.20	2157	1.12			
6	393.15	3681	1.91			

Figure S210 LCMS spectrum of compound NPD-3289

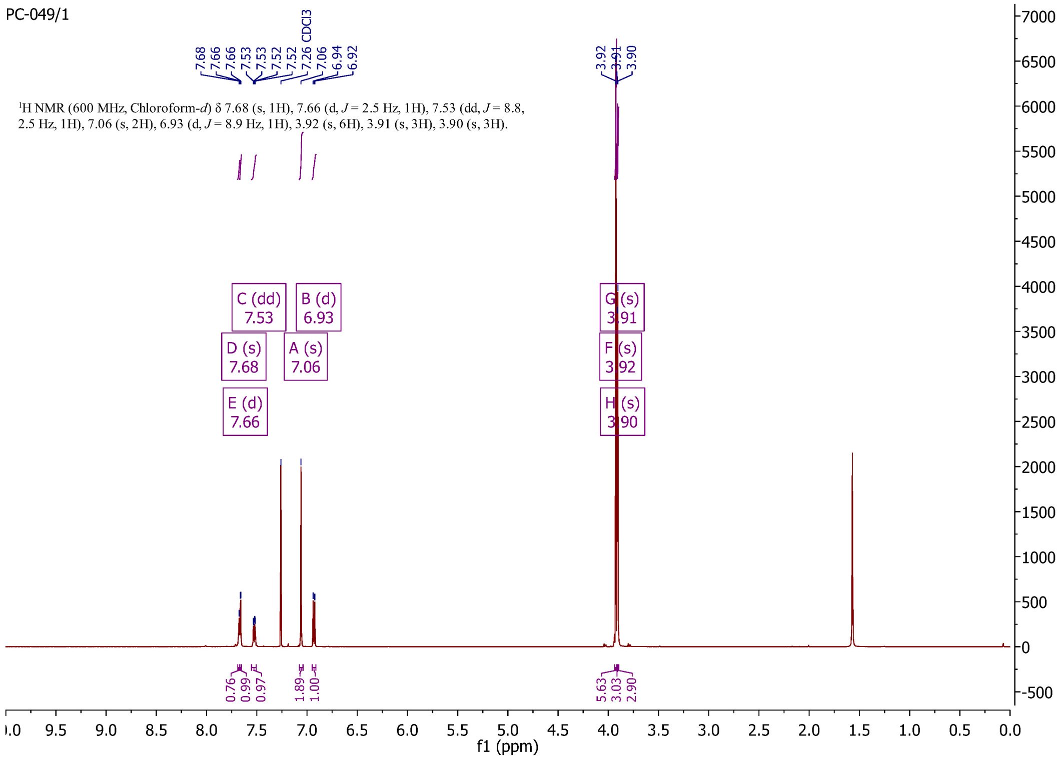

Figure S211 ^{1}H NMR spectrum of compound NPD-3289

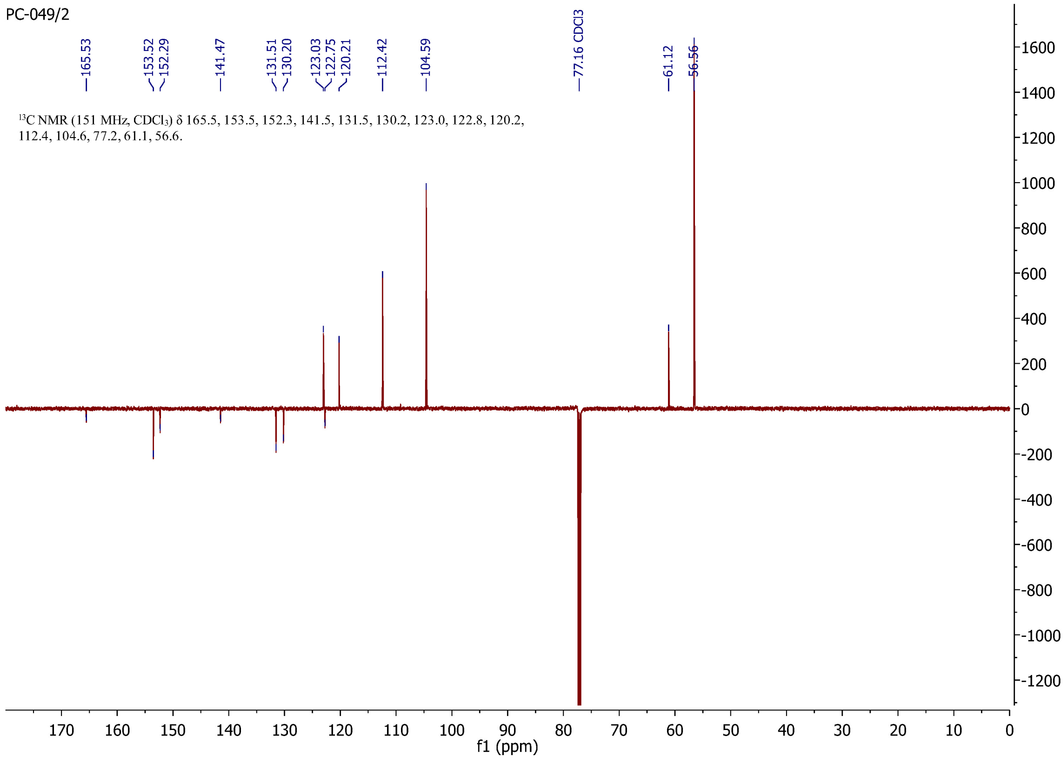

Figure S212 ^{13}C NMR spectrum of compound NPD-3289

Acquired by	: Admin
Date Acquired	: 19/2/2018 3:03:46 PM
Sample Name	: PC-152
Sample ID	:
Tray#	: 1
Vial#	: 38
Injection Volume	: 1
Data File	: C:\LabSolutions\Data\2018\2018-wk08\PC-152.lcd
Background File	: blanco 19022018.lcd
Method File	: Method SCAN ACID standard.lcm
Report Format	: DefaultLCMS.lcr
Tuning File	: C:\LabSolutions\Tuning\Tuning-ESI-pos-neg01072015a.lct
Processed by	: Admin
Modified Date	: 19/2/2018 5:33:09 PM

Medicinal Chemistry
Vrije Universiteit Amsterdam

Chromatogram
PC-152 C:\LabSolutions\Data\2018\2018-wk08\PC-152.lcd

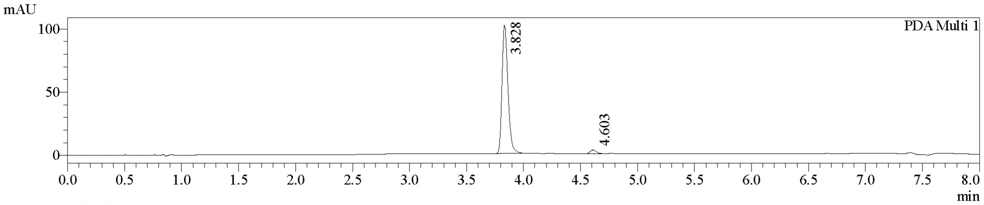

1 PDA Multi 1 / 254nm 4nm

PeakTable

PDA Ch1 254nm 4nm

Peak#	Name	Ret. Time	Area	Area %
1		3.828	365000	97.265
2		4.603	10263	2.735

MS Chromatogram
PC-152 C:\LabSolutions\Data\2018\2018-wk08\PC-152.lcd

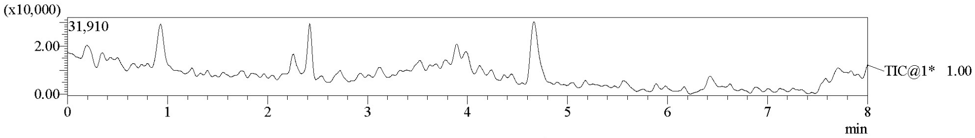

MS Spectrum Graph

#:1 Ret.Time:Single 3.900(Scan#:391)
Mass Peaks:5 Base Peak:157.05(7910) Polarity:Pos Segment1 - Event1

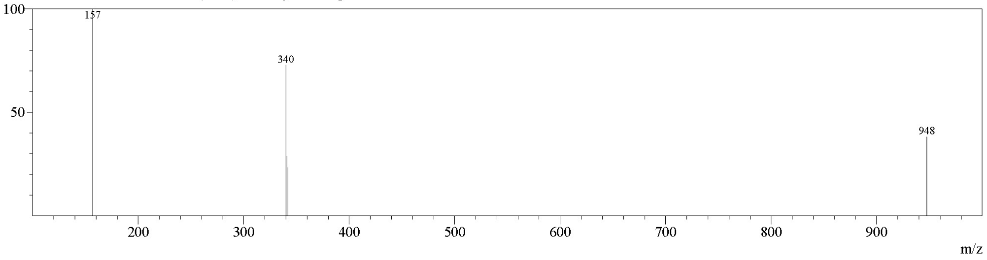

MS Spectrum Table

#:1 Ret.Time:
BG Mode:None
Mass Peaks:5 Base Peak:157.05(7910) Polarity:Pos Segment1 - Event1

#	m/z	Abs.Inten.	Rel.Inten.	Charge	Polarity	Monoisotopic
1	157.05	7910	100.00			
2	339.90	5759	72.81			
3	340.80	2284	28.87			
4	341.80	1854	23.44			
5	947.70	3011	38.07			

Figure S213 LCMS spectrum of compound NPD-3290

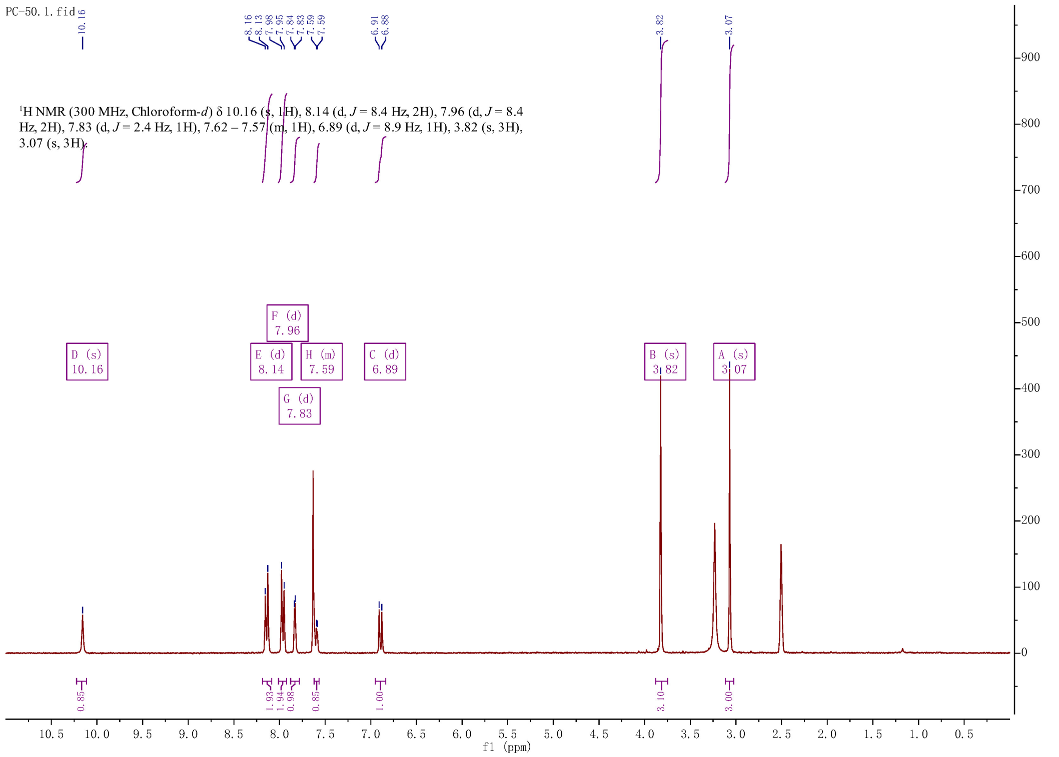

Figure S214 ^{1}H NMR spectrum of compound NPD-3290

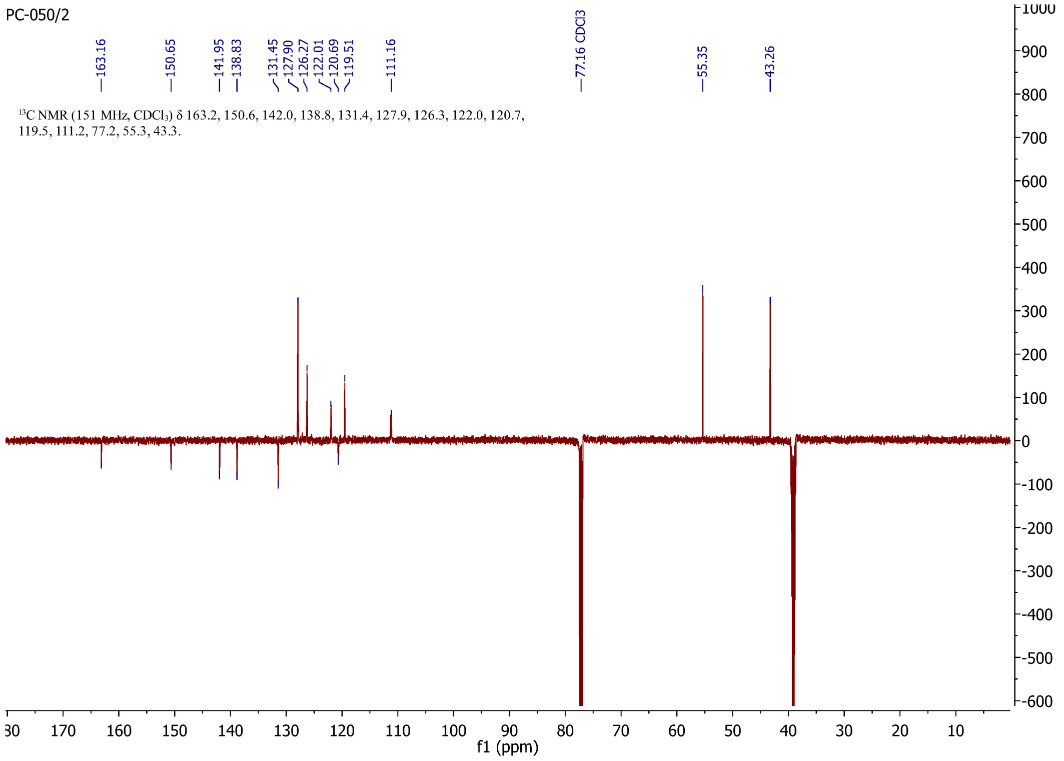

Figure S215 ^{13}C NMR spectrum of compound NPD-3290

Acquired by	: Admin
Date Acquired	: 23/2/2018 10:51:09 AM
Sample Name	: PC-155-1
Sample ID	:
Tray#	: 1
Vial#	: 6
Injection Volume	: 3
Data File	: C:\LabSolutions\Data\2018\2018-wk08\PC-155-1.lcd
Background File	: Blanco_23022018.lcd
Method File	: Method SCAN ACID standard.lcm
Report Format	: DefaultLCMS.lcr
Tuning File	: C:\LabSolutions\Tuning\Tuning-ESI-pos-neg01072015a.lct
Processed by	: Admin
Modified Date	: 23/2/2018 12:26:57 PM

Medicinal Chemistry
Vrije Universiteit Amsterdam

Chromatogram
PC-155-1 C:\LabSolutions\Data\2018\2018-wk08\PC-155-1.lcd

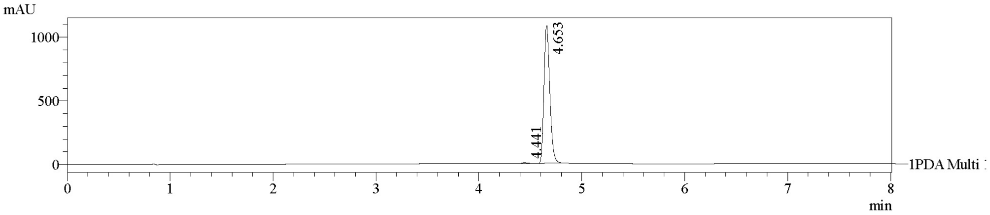

1 PDA Multi 1 / 254nm 4nm
PDA Ch1 254nm 4nm

Peak#	Name	Ret. Time	Area	Area %
1		4.441	26653	0.633
2		4.653	4183901	99.367

MS Chromatogram
PC-155-1 C:\LabSolutions\Data\2018\2018-wk08\PC-155-1.lcd

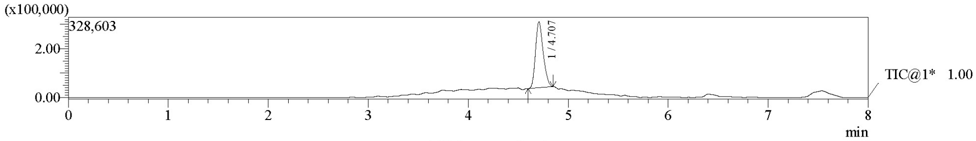

MS Spectrum Graph
#:1 Ret.Time:Averaged 4.700-4.720(Scan#:471-473)
Mass Peaks:5 Base Peak:292.00(187969) Polarity:Pos Segment1 - Event1

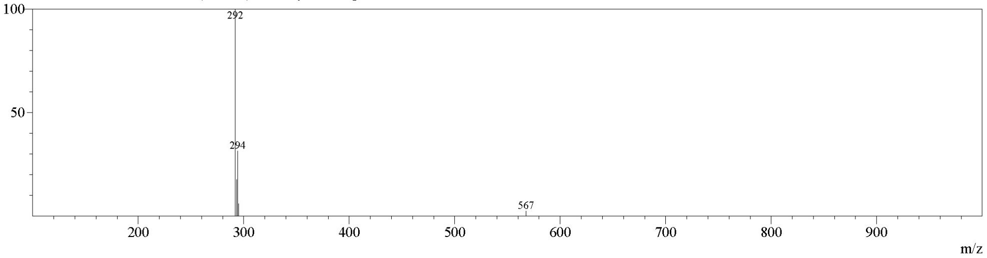

MS Spectrum Table
#:1 Ret.Time:
BG Mode:Calc 4.600<->4.850(461<->486)
Mass Peaks:5 Base Peak:292.00(187969) Polarity:Pos Segment1 - Event1

#	m/z	Abs.Inten.	Rel.Inten.	Charge	Polarity	Monoisotopic
1	292.00	187969	100.00			
2	293.05	32956	17.53			
3	294.05	58856	31.31			
4	294.95	10936	5.82			
5	567.45	4178	2.22			

Figure S216 LCMS spectrum of compound NPD-3291

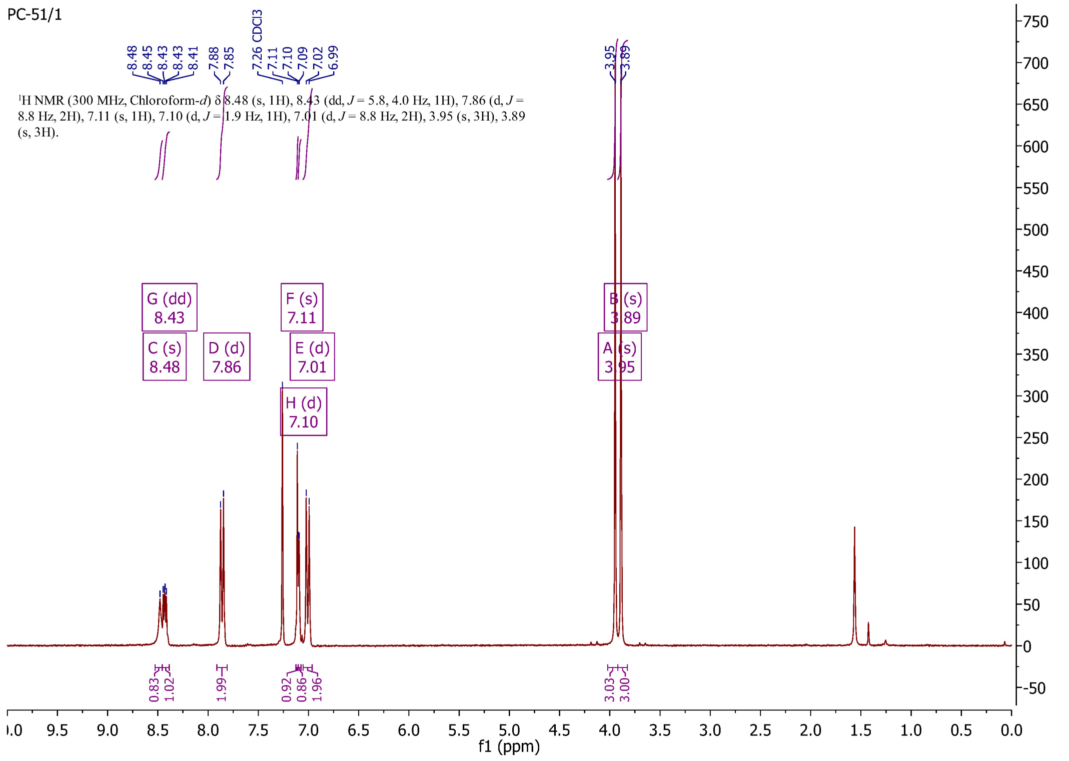

Figure S217 ^{1}H NMR spectrum of compound NPD-3291

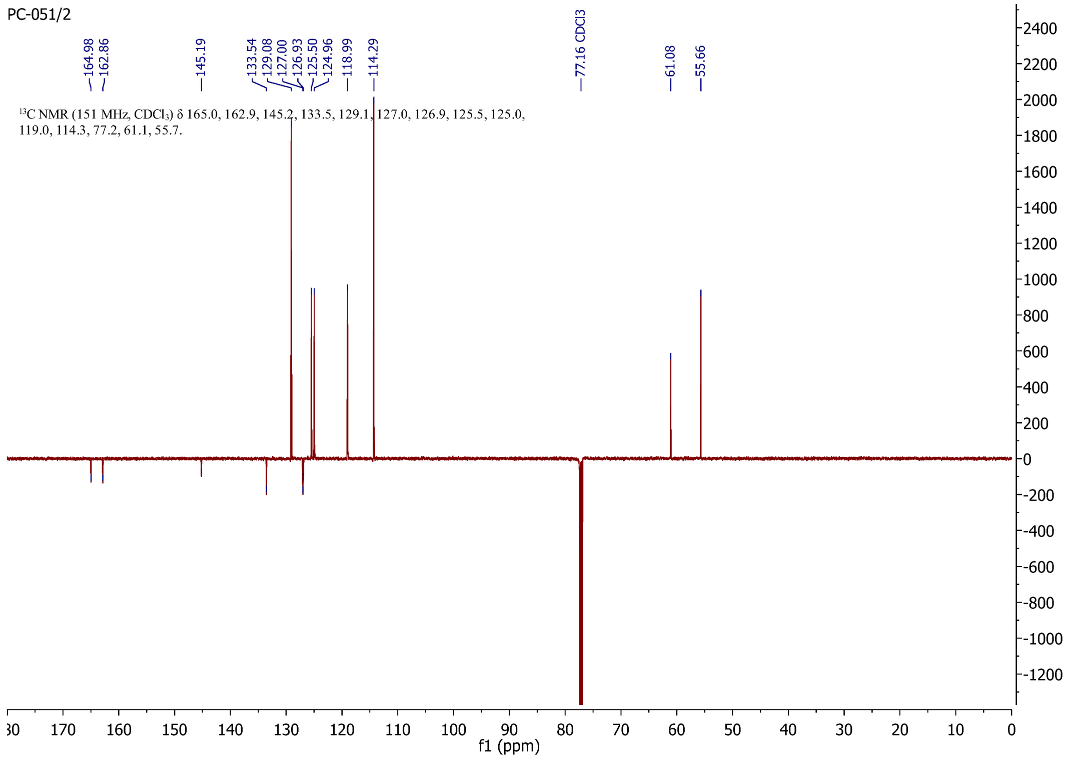

Figure S218 ^{13}C NMR spectrum of compound NPD-3291

Acquired by	: Admin
Date Acquired	: 19/2/2018 3:12:52 PM
Sample Name	: PC-156
Sample ID	:
Tray#	: 1
Vial#	: 39
Injection Volume	: 1
Data File	: C:\LabSolutions\Data\2018\2018-wk08\PC-156.lcd
Background File	: blanco 19022018.lcd
Method File	: Method SCAN ACID standard.lcm
Report Format	: DefaultLCMS.lcr
Tuning File	: C:\LabSolutions\Tuning\Tuning-ESI-pos-neg01072015a.lct
Processed by	: Admin
Modified Date	: 19/2/2018 5:34:20 PM

Medicinal Chemistry
Vrije Universiteit Amsterdam

Chromatogram
PC-156 C:\LabSolutions\Data\2018\2018-wk08\PC-156.lcd

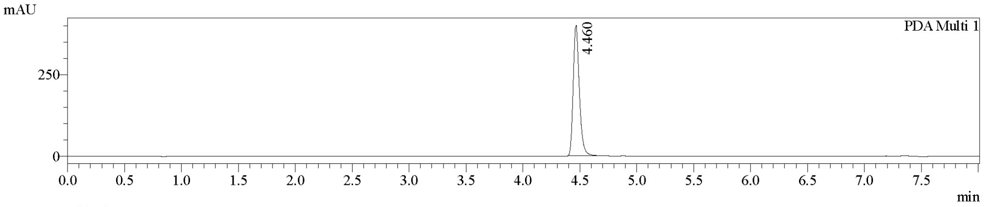

1 PDA Multi 1 / 254nm 4nm

PeakTable

PDA Ch1 254nm 4nm

Peak#	Name	Ret. Time	Area	Area %
1		4.460	1487845	100.000

MS Chromatogram
PC-156 C:\LabSolutions\Data\2018\2018-wk08\PC-156.lcd

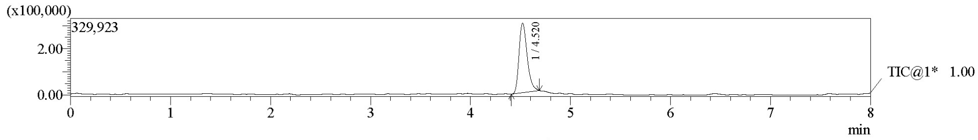

MS Spectrum Graph

#:1 Ret.Time:Averaged 4.510-4.530(Scan#:452-454)
Mass Peaks:7 Base Peak:322.05(186972) Polarity:Pos Segment1 - Event1

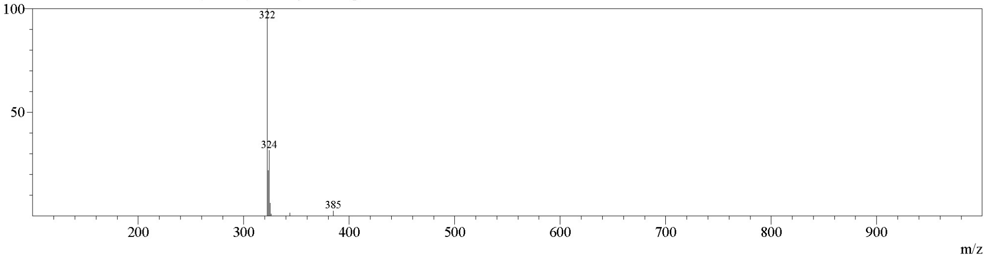

MS Spectrum Table

#:1 Ret.Time:
BG Mode:Calc 4.410<->4.690(442<->470)
Mass Peaks:7 Base Peak:322.05(186972) Polarity:Pos Segment1 - Event1

#	m/z	Abs.Inten.	Rel.Inten.	Charge	Polarity	Monoisotopic
1	322.05	186972	100.00			
2	323.10	40864	21.86			
3	324.05	59111	31.61			
4	325.05	11728	6.27			
5	326.10	1909	1.02			
6	343.95	2512	1.34			
7	385.05	4520	2.42			

Figure S219 LCMS spectrum of compound NPD-3292

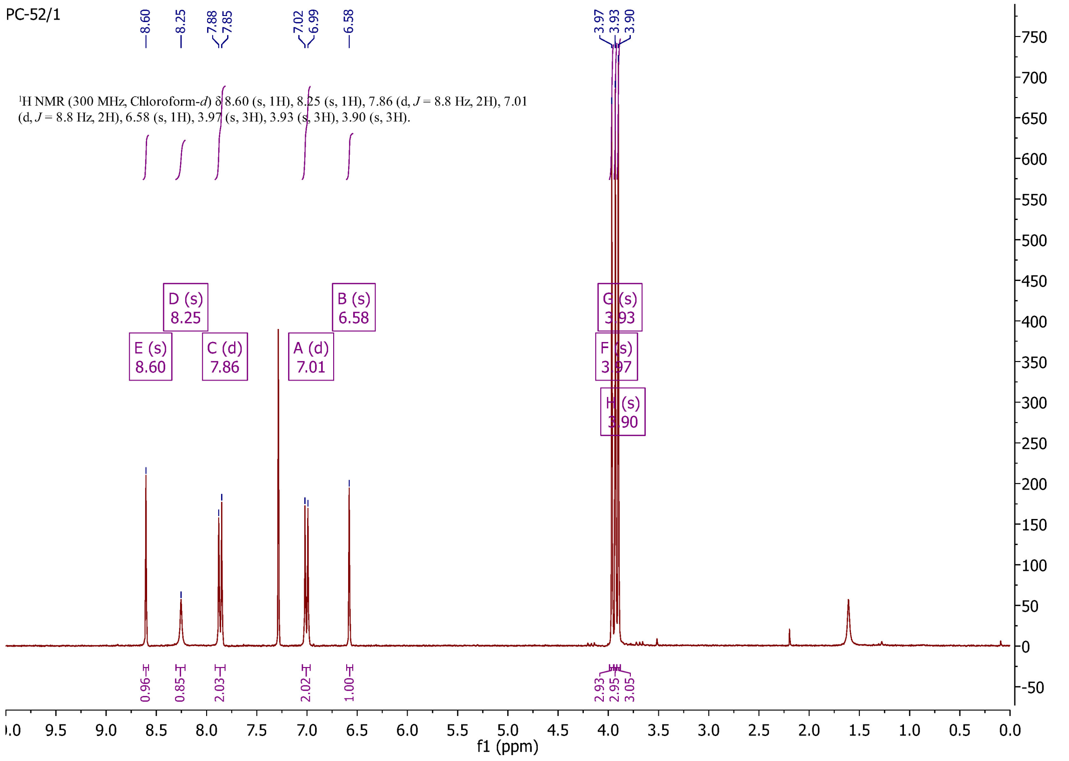

Figure S220 ^{1}H NMR spectrum of compound NPD-3292

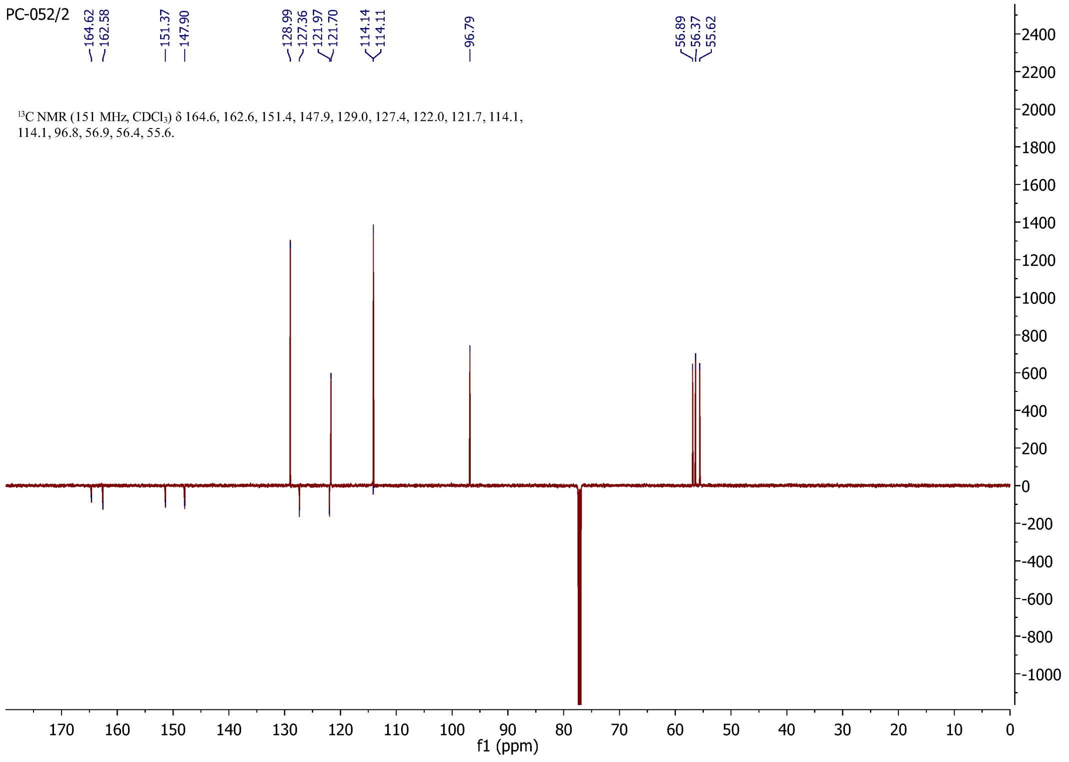

Figure S221 ^{13}C NMR spectrum of compound NPD-3292

Acquired by	: Admin
Date Acquired	: 26/2/2018 10:10:45 AM
Sample Name	: PC-157
Sample ID	:
Tray#	: 1
Vial#	: 2
Injection Volume	: 1
Data File	: C:\LabSolutions\Data\2018\2018-wk09\PC-157.lcd
Background File	: Blanco_26022018.lcd
Method File	: Method SCAN ACID standard.lcm
Report Format	: DefaultLCMS.lcr
Tuning File	: C:\LabSolutions\Tuning\Tuning-ESI-pos-neg01072015a.lct
Processed by	: Admin
Modified Date	: 26/2/2018 10:30:55 AM

Medicinal Chemistry
Vrije Universiteit Amsterdam

Chromatogram
PC-157 C:\LabSolutions\Data\2018\2018-wk09\PC-157.lcd

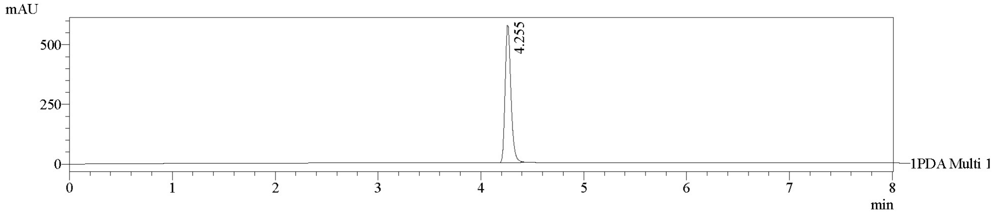

1 PDA Multi 1 / 254nm 4nm
PDA Ch1 254nm 4nm

Peak#	Name	Ret. Time	Area	Area %
1		4.255	2260471	100.000

MS Chromatogram
PC-157 C:\LabSolutions\Data\2018\2018-wk09\PC-157.lcd

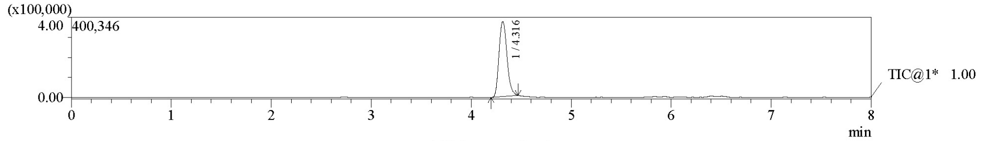

MS Spectrum Graph
#:1 Ret.Time:Averaged 4.310-4.330(Scan#:432-434)
Mass Peaks:5 Base Peak:292.05(246719) Polarity:Pos Segment1 - Event1

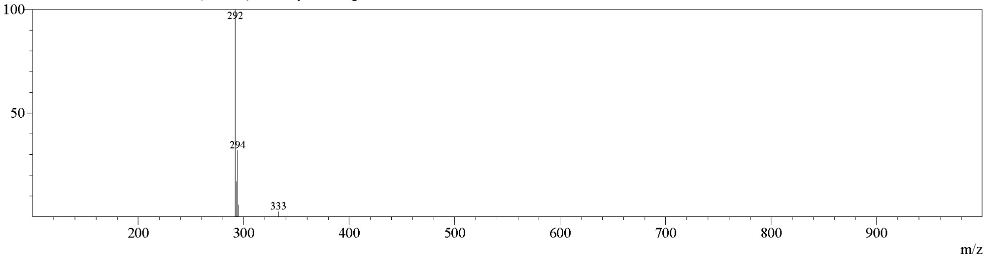

MS Spectrum Table
#:1 Ret.Time:
BG Mode:Calc 4.200<->4.470(421<->448)
Mass Peaks:5 Base Peak:292.05(246719) Polarity:Pos Segment1 - Event1

#	m/z	Abs.Inten.	Rel.Inten.	Charge	Polarity	Monoisotopic
1	292.05	246719	100.00			
2	293.05	41250	16.72			
3	294.05	78405	31.78			
4	295.00	13930	5.65			
5	333.05	5381	2.18			

Figure S222 LCMS spectrum of compound NPD-3293

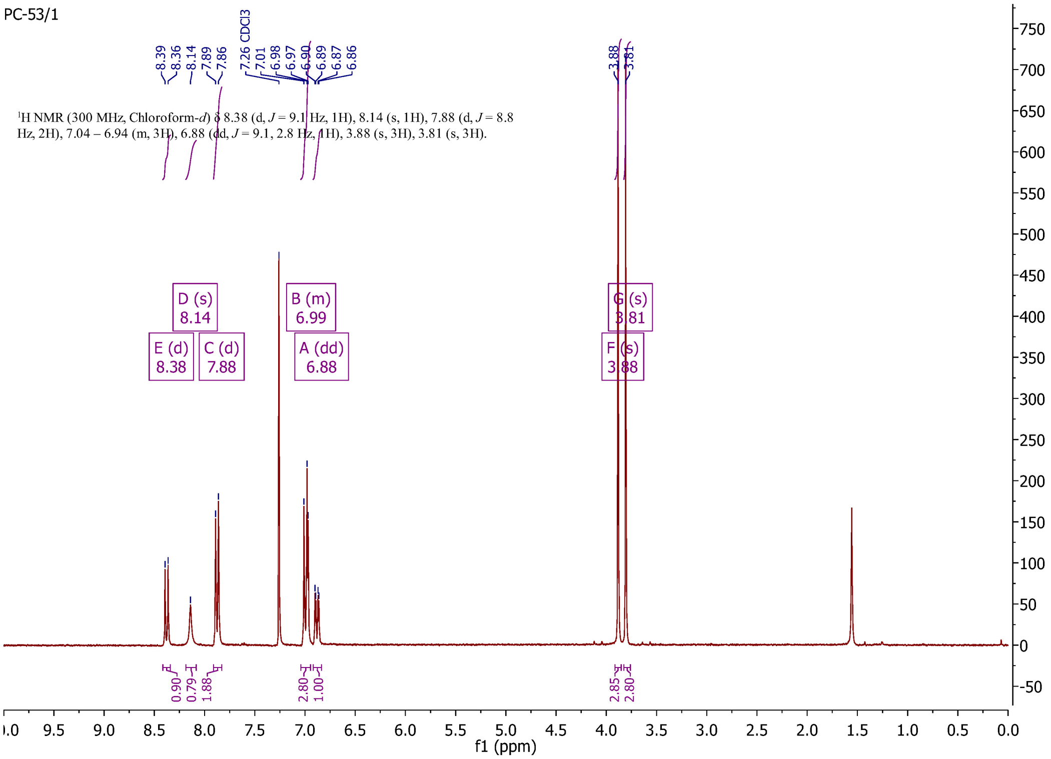

Figure S223 ^{1}H NMR spectrum of compound NPD-3293

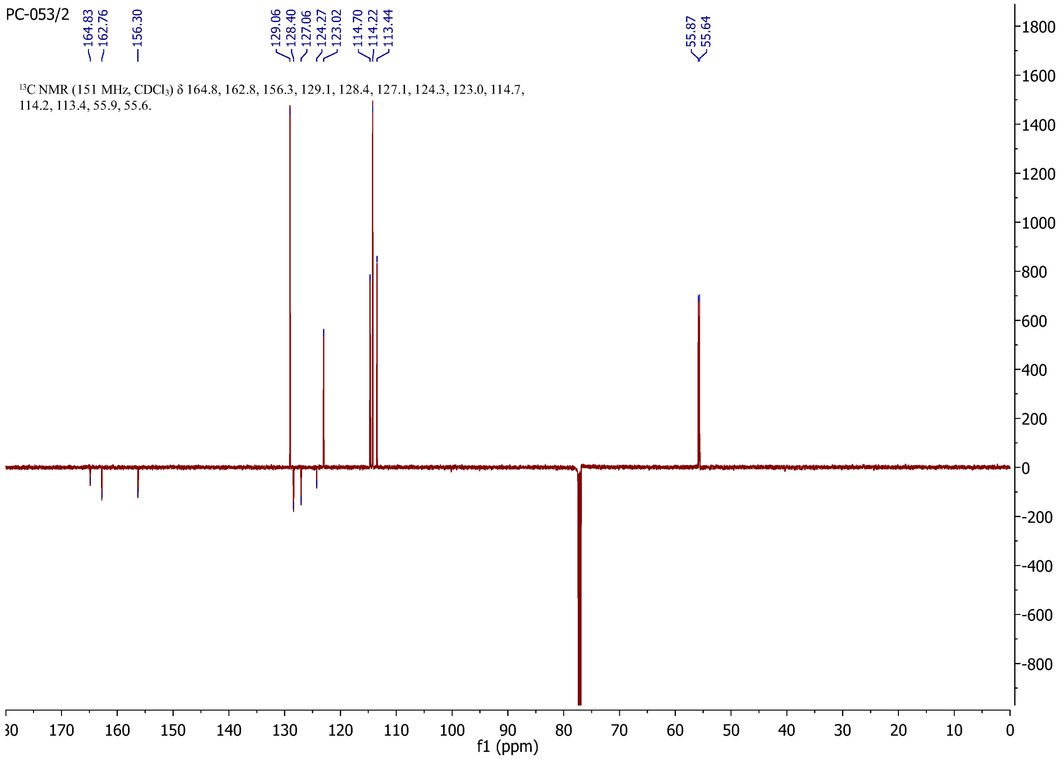

Figure S224 ^{13}C NMR spectrum of compound NPD-3293

Acquired by	: Admin
Date Acquired	: 21/2/2018 3:44:46 PM
Sample Name	: PC-159
Sample ID	:
Tray#	: 1
Vial#	: 32
Injection Volume	: 1
Data File	: C:\LabSolutions\Data\2018\2018-wk08\PC-159.lcd
Background File	: blanco 21022018.lcd
Method File	: Method SCAN ACID standard.lcm
Report Format	: DefaultLCMS.lcr
Tuning File	: C:\LabSolutions\Tuning\Tuning-ESI-pos-neg01072015a.lct
Processed by	: Admin
Modified Date	: 21/2/2018 3:58:32 PM

PDA Graph

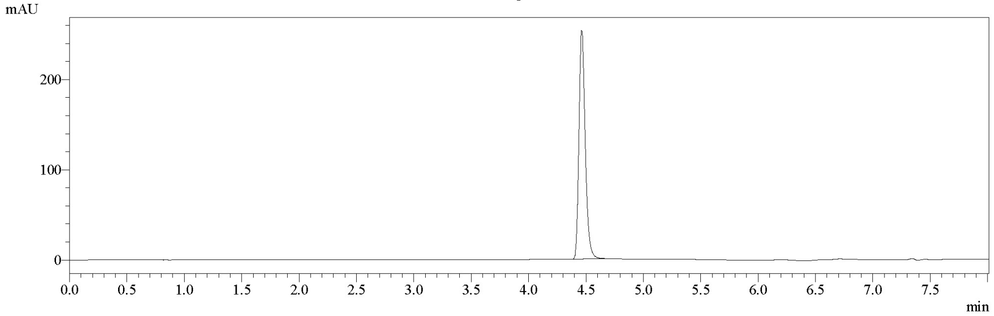

PDA Ch1 254nm 4nm

Peak#	Name	Ret. Time	Area	Area %
1		4.458	971595	100.000

MS Spectrum Graph

#:1 Ret.Time:Averaged 4.510-4.530(Scan#:452-454)
Mass Peaks:11 Base Peak:292.00(80593) Polarity:Pos Segment1 - Event1

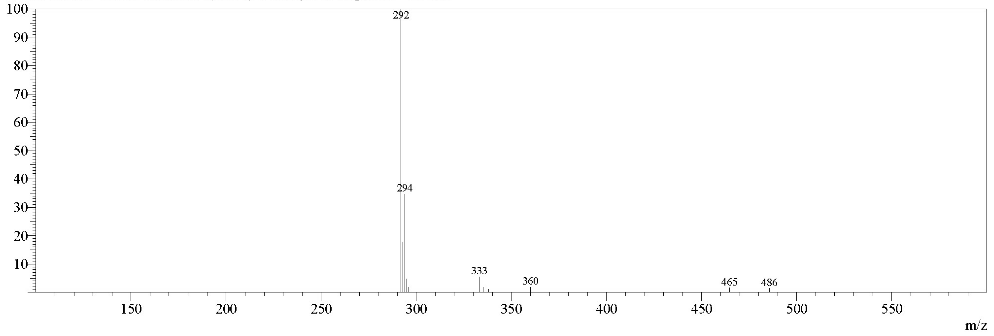

MS Spectrum Table

#:1 Ret.Time:
BG Mode:Calc 4.420<->4.650(443<->466)
Mass Peaks:11 Base Peak:292.00(80593) Polarity:Pos Segment1 - Event1

#	m/z	Abs.Inten.	Rel.Inten.	Charge	Polarity	Monoisotopic
1	292.00	80593	100.00			
2	293.00	14279	17.72			
3	294.00	27976	34.71			
4	295.10	3835	4.76			
5	296.10	1420	1.76			
6	333.00	4410	5.47			

#	m/z	Abs.Inten.	Rel.Inten.	Charge	Polarity	Monoisotopic
7	335.10	1402	1.74			
8	338.00	903	1.12			
9	359.95	1511	1.87			
10	464.80	1265	1.57			
11	485.80	1108	1.37			

Figure S225 LCMS spectrum of compound NPD-3294

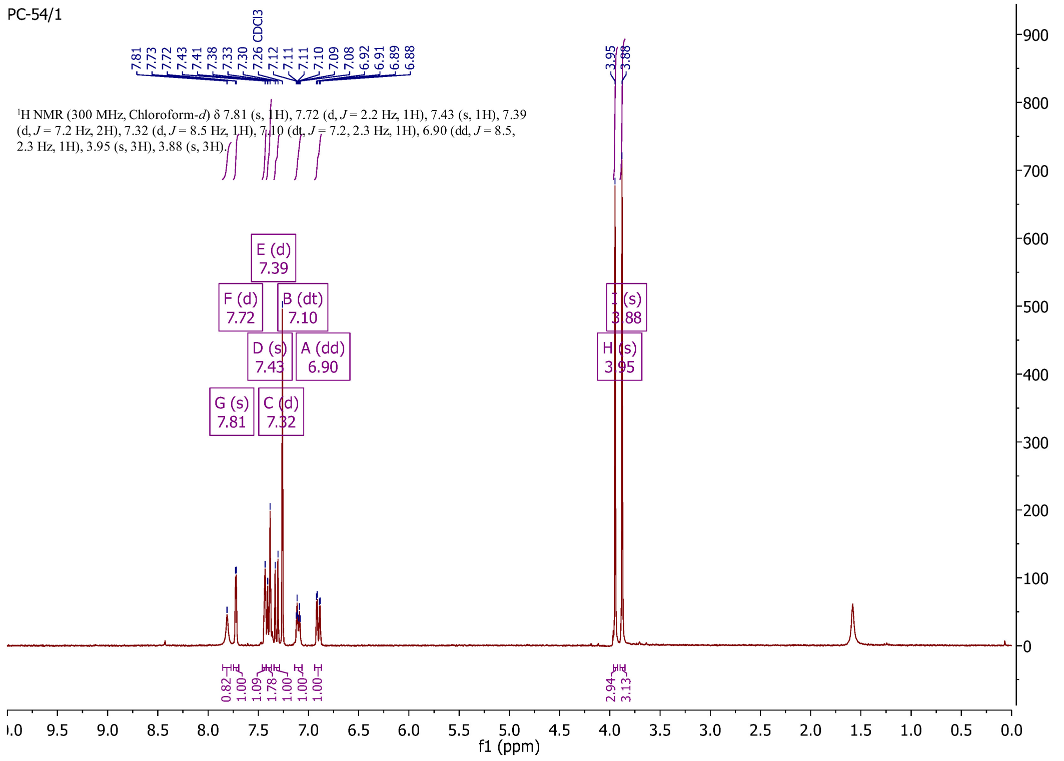

Figure S226 ^{1}H NMR spectrum of compound NPD-3294

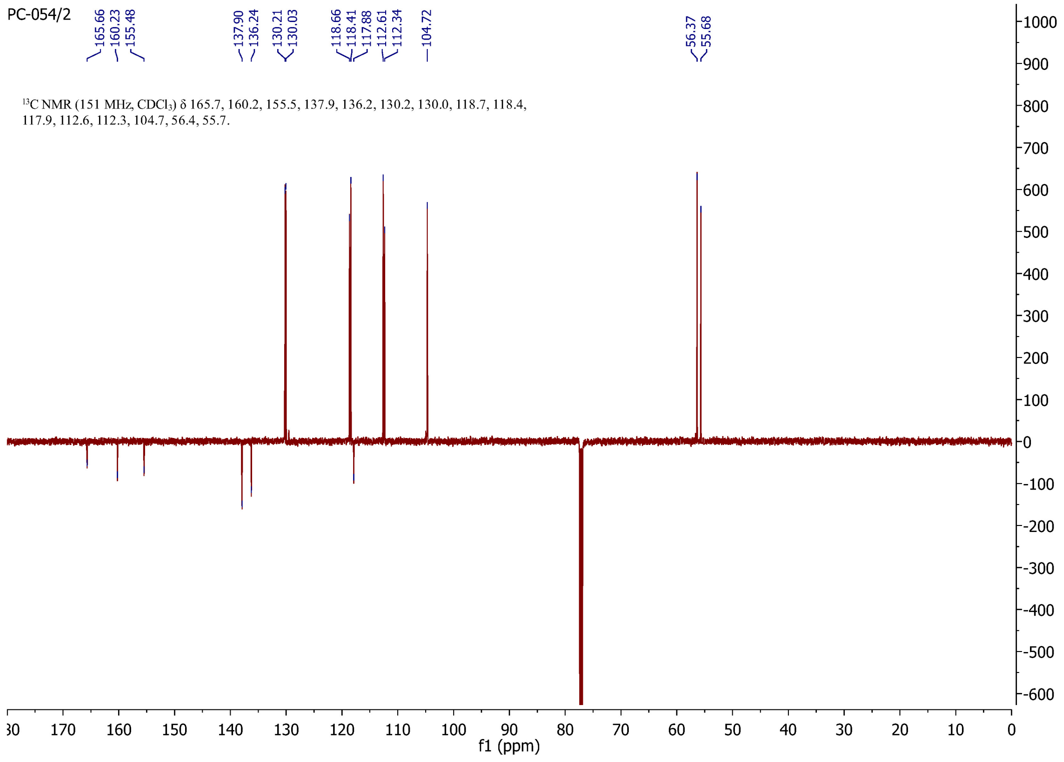

Figure S227 ^{13}C NMR spectrum of compound NPD-3294

Acquired by	: Admin
Date Acquired	: 26/2/2018 10:19:23 AM
Sample Name	: PC-161
Sample ID	:
Tray#	: 1
Vial#	: 3
Injection Volume	: 1
Data File	: C:\LabSolutions\Data\2018\2018-wk09\PC-161.lcd
Background File	: Blanco_26022018.lcd
Method File	: Method SCAN ACID standard.lcm
Report Format	: DefaultLCMS.lcr
Tuning File	: C:\LabSolutions\Tuning\Tuning-ESI-pos-neg01072015a.lct
Processed by	: Admin
Modified Date	: 26/2/2018 10:31:42 AM

Medicinal Chemistry
Vrije Universiteit Amsterdam

Chromatogram
PC-161 C:\LabSolutions\Data\2018\2018-wk09\PC-161.lcd

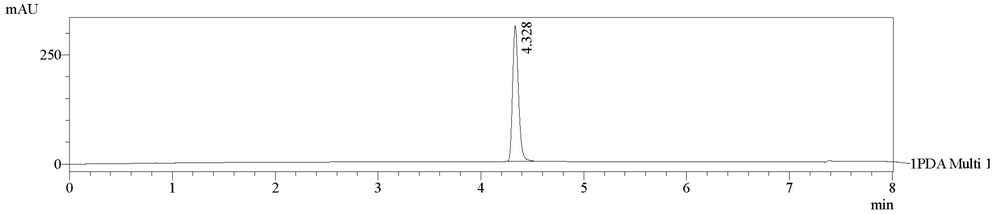

1 PDA Multi 1 / 254nm 4nm
PDA Ch1 254nm 4nm

Peak#	Name	Ret. Time	Area	Area %
1		4.328	1216400	100.000

MS Chromatogram
PC-161 C:\LabSolutions\Data\2018\2018-wk09\PC-161.lcd

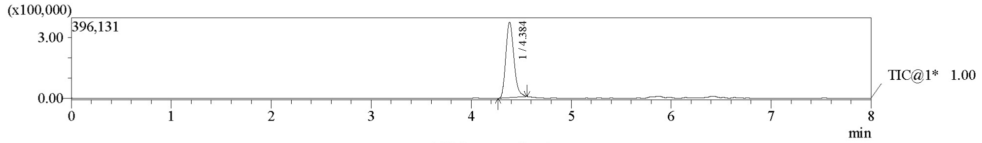

MS Spectrum Graph
#:1 Ret.Time:Averaged 4.370-4.390(Scan#:438-440)
Mass Peaks:7 Base Peak:292.00(245124) Polarity:Pos Segment1 - Event1

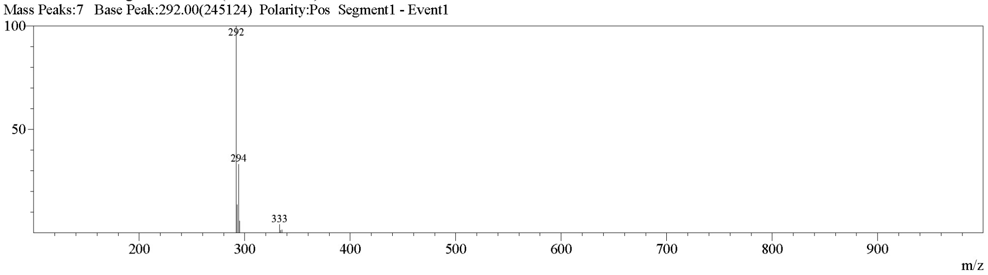

MS Spectrum Table
#:1 Ret.Time:
BG Mode:Calc 4.270<->4.560(428<->457)
Mass Peaks:7 Base Peak:292.00(245124) Polarity:Pos Segment1 - Event1

#	m/z	Abs.Inten.	Rel.Inten.	Charge	Polarity	Monoisotopic
1	292.00	245124	100.00			
2	293.00	33269	13.57			
3	294.00	80877	32.99			
4	295.00	14030	5.72			
5	333.10	9442	3.85			
6	334.00	2712	1.11			
7	335.10	3083	1.26			

Figure S228 LCMS spectrum of compound NPD-3295

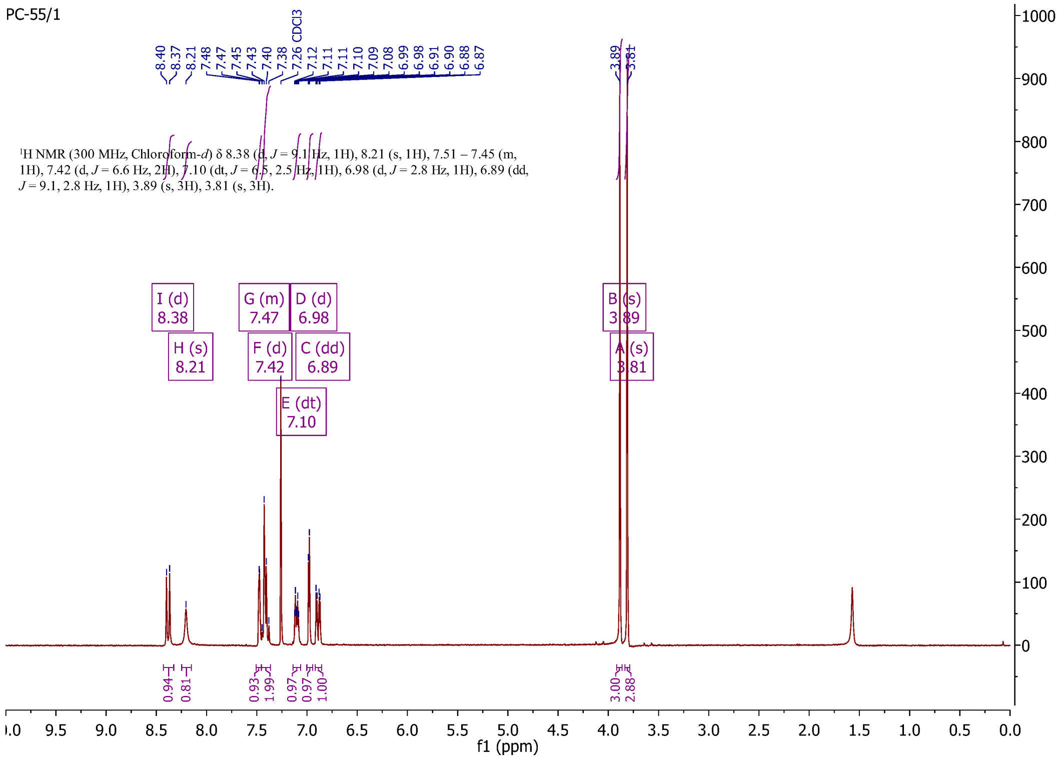

Figure S229 ^{1}H NMR spectrum of compound NPD-3295

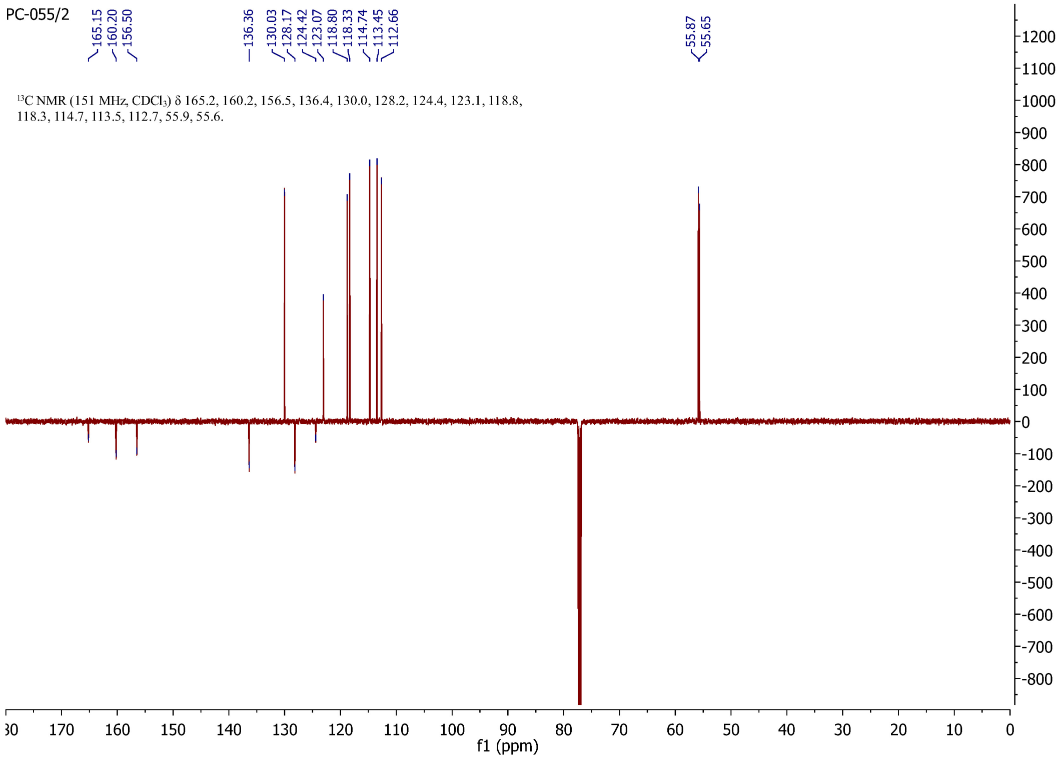

Figure S230 ^{13}C NMR spectrum of compound NPD-3295

Acquired by	: Admin
Date Acquired	: 26/2/2018 10:28:00 AM
Sample Name	: PC-162
Sample ID	:
Tray#	: 1
Vial#	: 4
Injection Volume	: 1
Data File	: C:\LabSolutions\Data\2018\2018-wk09\PC-162.lcd
Background File	: Blanco_26022018.lcd
Method File	: Method SCAN ACID standard.lcm
Report Format	: DefaultLCMS.lcr
Tuning File	: C:\LabSolutions\Tuning\Tuning-ESI-pos-neg01072015a.lct
Processed by	: Admin
Modified Date	: 26/2/2018 10:43:14 AM

Medicinal Chemistry
Vrije Universiteit Amsterdam

Chromatogram
PC-162 C:\LabSolutions\Data\2018\2018-wk09\PC-162.lcd

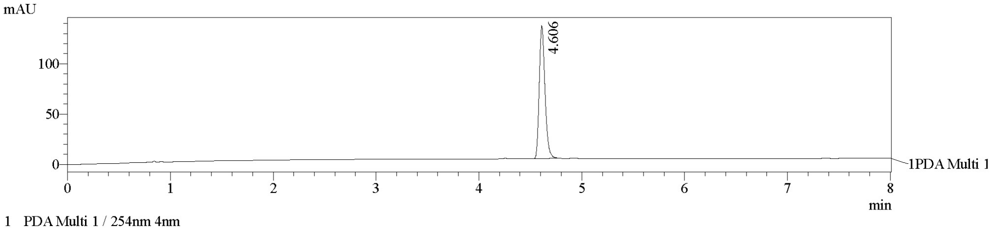

1 PDA Multi 1 / 254nm 4nm
PDA Ch1 254nm 4nm

Peak#	Name	Ret. Time	Area	Area %
1		4.606	514067	100.000

MS Chromatogram
PC-162 C:\LabSolutions\Data\2018\2018-wk09\PC-162.lcd

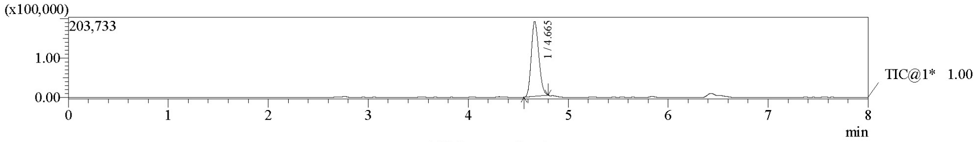

MS Spectrum Graph
#:1 Ret.Time:Averaged 4.660-4.680(Scan#:467-469)
Mass Peaks:8 Base Peak:306.05(112146) Polarity:Pos Segment1 - Event1

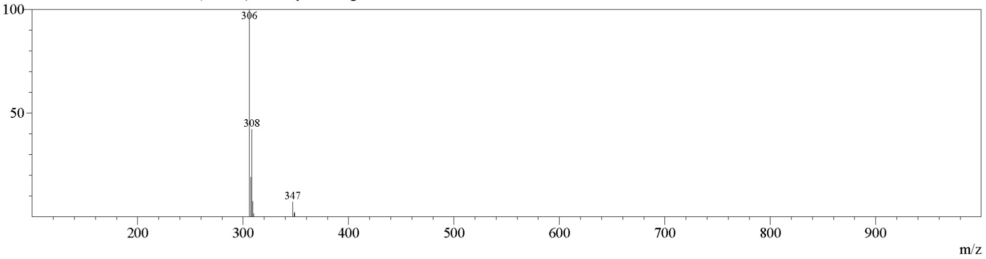

MS Spectrum Table
#:1 Ret.Time:
BG Mode:Calc 4.560<->4.800(457<->481)
Mass Peaks:8 Base Peak:306.05(112146) Polarity:Pos Segment1 - Event1

#	m/z	Abs.Inten.	Rel.Inten.	Charge	Polarity	Monoisotopic
1	306.05	112146	100.00			
2	307.05	21393	19.08			
3	308.05	47234	42.12			
4	309.05	8193	7.31			
5	310.05	1551	1.38			
6	347.15	8050	7.18			
7	348.20	1764	1.57			
8	349.00	2376	2.12			

Figure S231 LCMS spectrum of compound NPD-3296

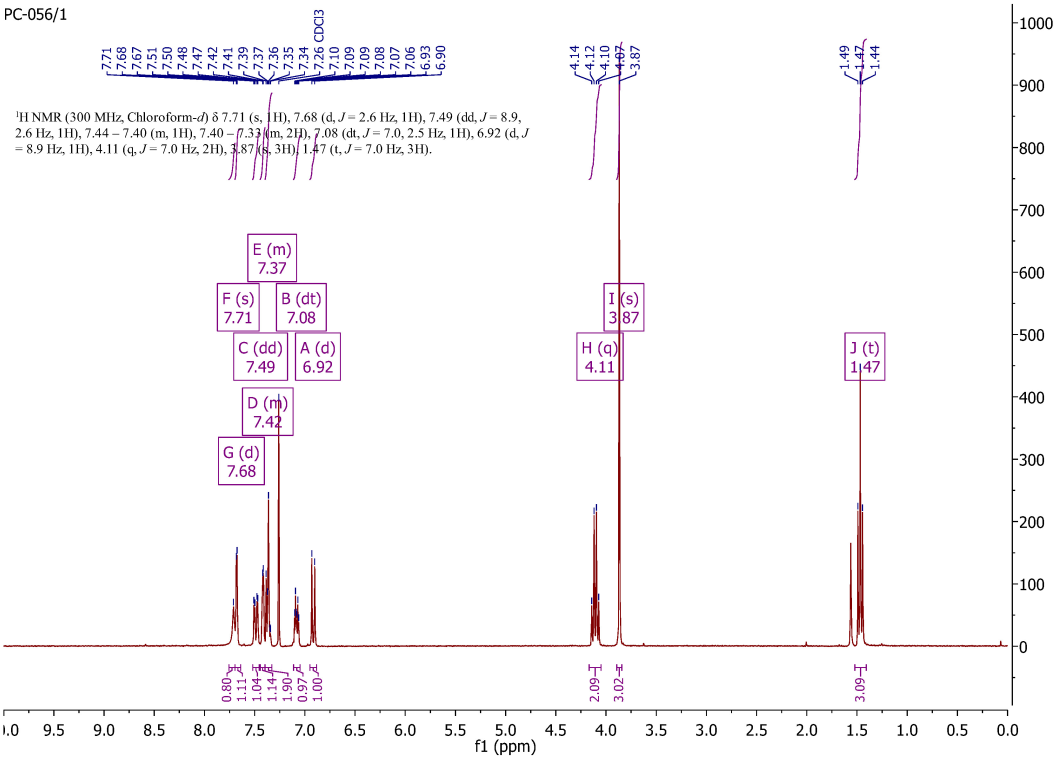

Figure S232 ^{1}H NMR spectrum of compound NPD-3296

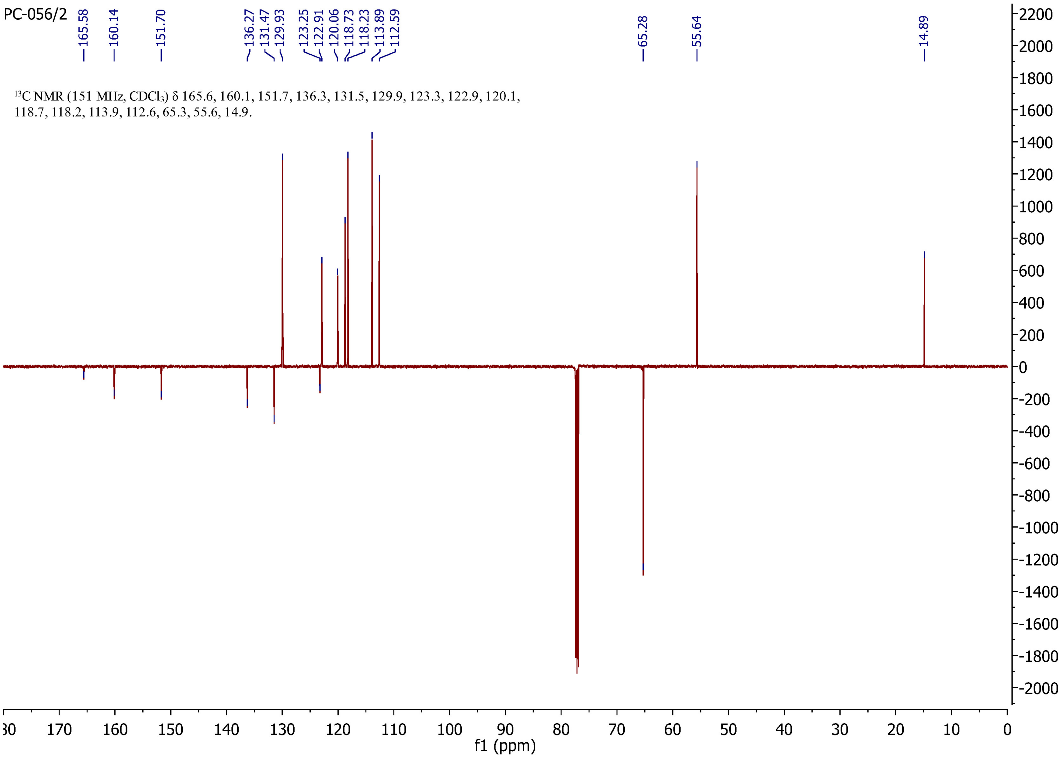

Figure S233 ^{13}C NMR spectrum of compound NPD-3296

Acquired by	: Admin
Date Acquired	: 26/2/2018 11:45:35 AM
Sample Name	: PC-163
Sample ID	:
Tray#	: 1
Vial#	: 8
Injection Volume	: 3
Data File	: C:\LabSolutions\Data\2018\2018-wk09\PC-163-1.lcd
Background File	: Blanco_26022018.lcd
Method File	: Method SCAN ACID standard.lcm
Report Format	: DefaultLCMS.lcr
Tuning File	: C:\LabSolutions\Tuning\Tuning-ESI-pos-neg01072015a.lct
Processed by	: Admin
Modified Date	: 26/2/2018 12:23:22 PM

PDA Graph

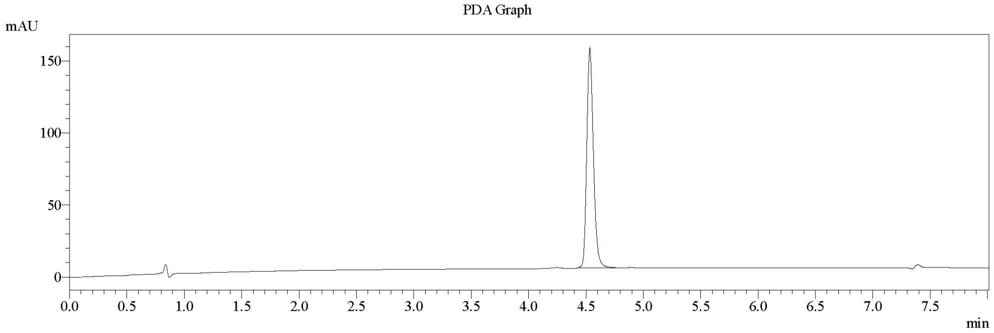

PDA Ch1 254nm 4nm

Peak#	Name	Ret. Time	Area	Area %
1		4.528	608948	100.000

MS Spectrum Graph

#:1 Ret.Time:Averaged 4.570-4.590(Scan#:458-460)
Mass Peaks:5 Base Peak:322.00(238263) Polarity:Pos Segment1 - Event1

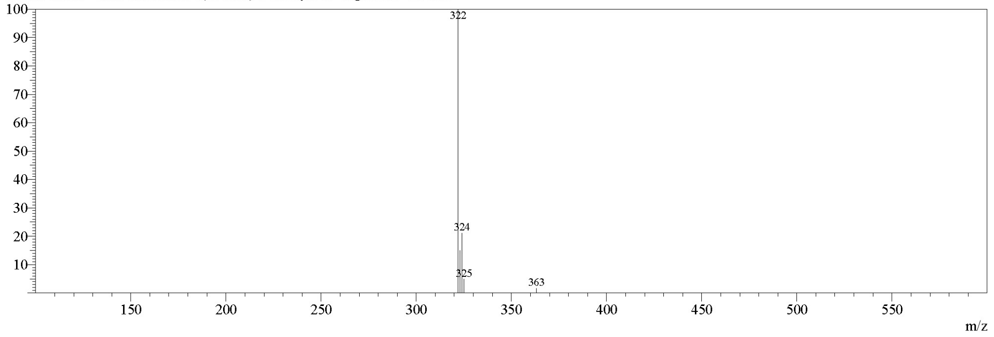

MS Spectrum Table

#:1 Ret.Time:
BG Mode:Calc 4.470<->4.740(448<->475)
Mass Peaks:5 Base Peak:322.00(238263) Polarity:Pos Segment1 - Event1

#	m/z	Abs.Inten.	Rel.Inten.	Charge	Polarity	Monoisotopic
1	322.00	238263	100.00			
2	323.10	35687	14.98			
3	324.05	50159	21.05			
4	325.05	11620	4.88			
5	363.05	4028	1.69			

Figure S234 LCMS spectrum of compound NPD-3297

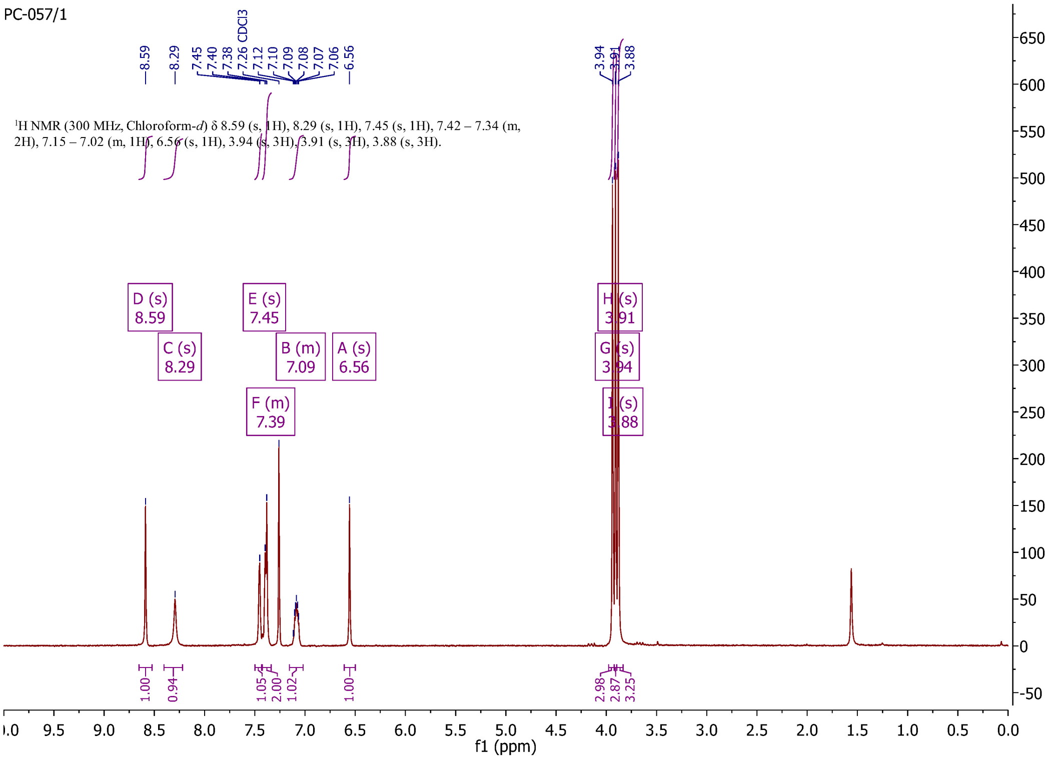

Figure S235 ^{1}H NMR spectrum of compound NPD-3297

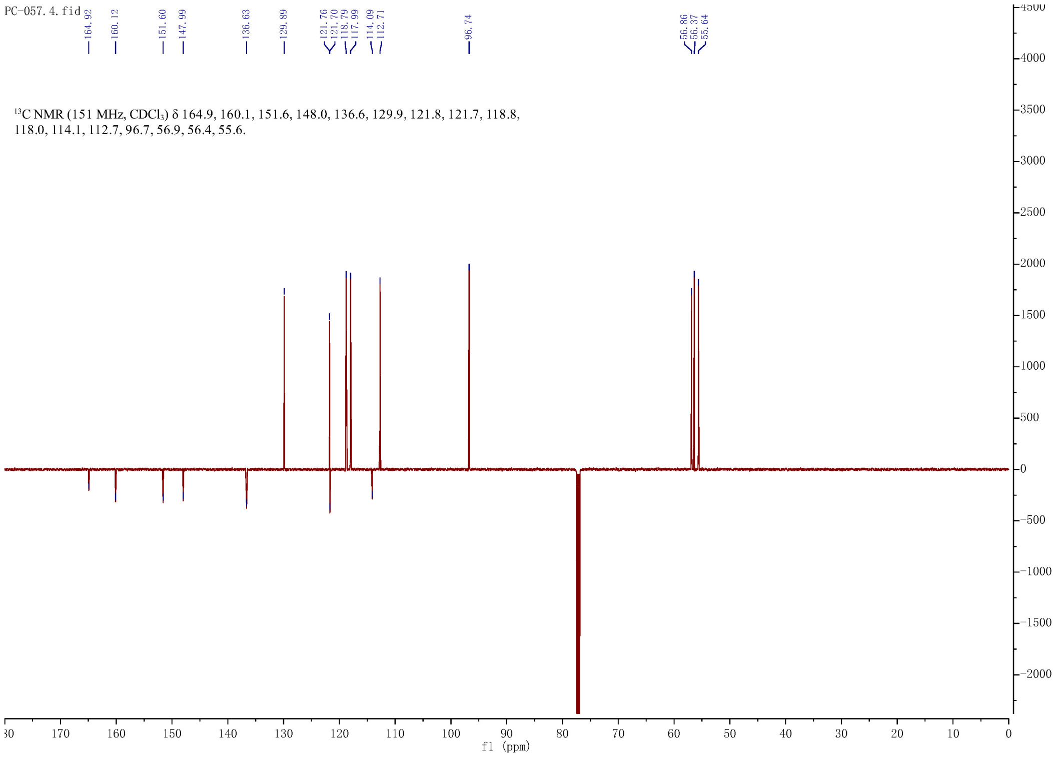

Figure S236 ^{13}C NMR spectrum of compound NPD-3297

Acquired by : Admin
Date Acquired : 26/2/2018 11:11:08 AM
Sample Name : PC-165
Sample ID :
Tray# : 1
Vial# : 9
Injection Volume : 1
Data File : C:\LabSolutions\Data\2018\2018-wk09\PC-165.lcd
Background File : Blanco_26022018.lcd
Method File : Method SCAN ACID standard.lcm
Report Format : DefaultLCMS.lcr
Tuning File : C:\LabSolutions\Tuning\Tuning-ESI-pos-neg01072015a.lct
Processed by : Admin
Modified Date : 26/2/2018 11:23:02 AM

PDA Graph

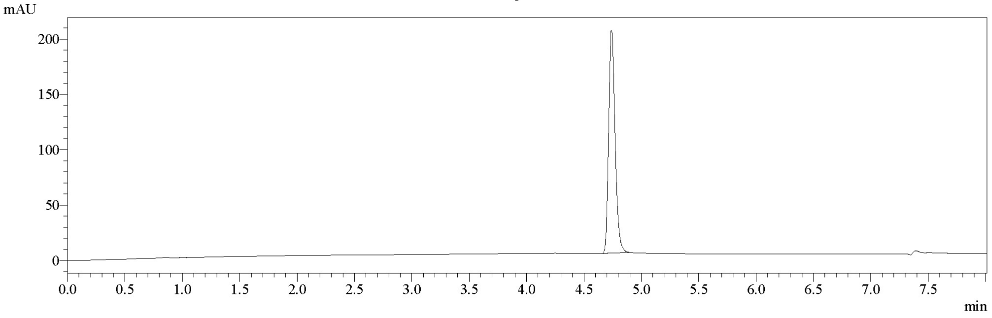

PDA Ch1 254nm 4nm

Peak#	Name	Ret. Time	Area	Area %
1		4.735	787463	100.000

MS Spectrum Graph

#:1 Ret.Time:Averaged 4.790-4.810(Scan#:480-482)
Mass Peaks:12 Base Peak:290.00(138269) Polarity:Pos Segment1 - Event1

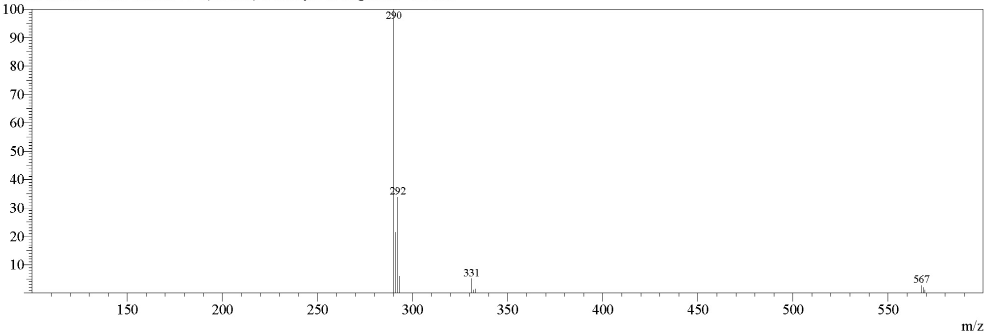

MS Spectrum Table

#:1 Ret.Time:
BG Mode:Calc 4.690<->4.930(470<->494)
Mass Peaks:12 Base Peak:290.00(138269) Polarity:Pos Segment1 - Event1

#	m/z	Abs.Inten.	Rel.Inten.	Charge	Polarity	Monoisotopic
1	290.00	138269	100.00			
2	291.05	29704	21.48			
3	292.10	46657	33.74			
4	293.10	8236	5.96			
5	331.00	6970	5.04			
6	332.00	1636	1.18			

#	m/z	Abs.Inten.	Rel.Inten.	Charge	Polarity	Monoisotopic
7	333.15	2105	1.52			
8	567.40	3766	2.72			
9	568.50	2826	2.04			
10	569.45	1539	1.11			
11	688.20	1420	1.03			
12	690.25	2169	1.57			

Figure S237 LCMS spectrum of compound NPD-3298

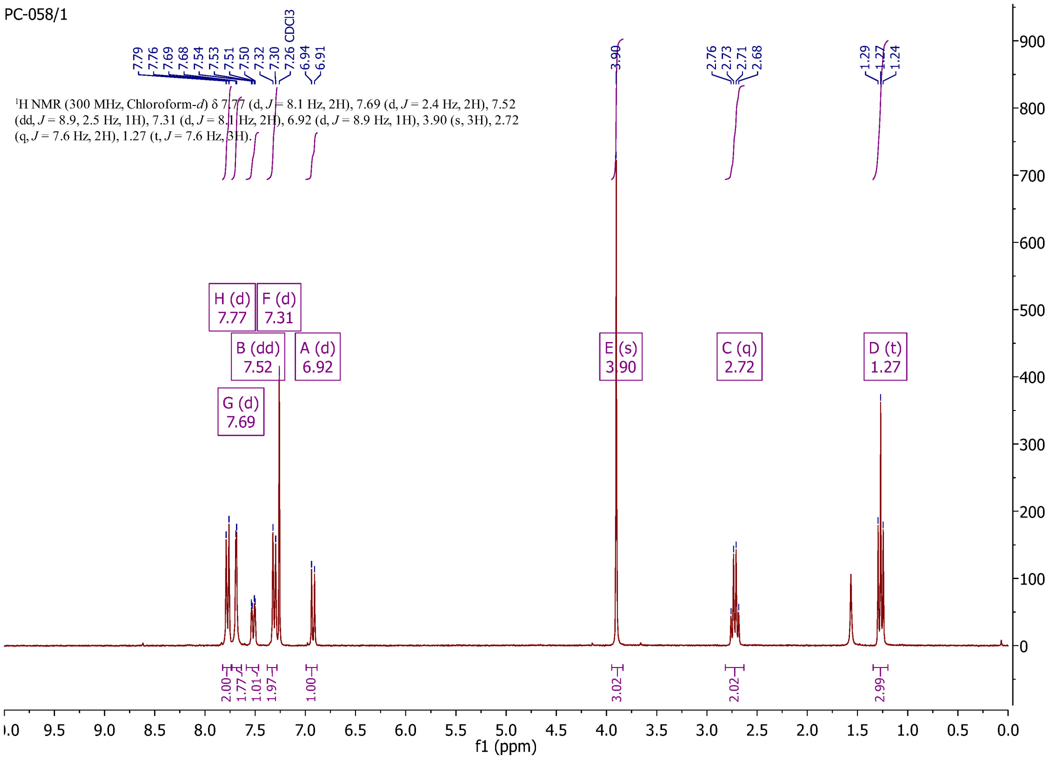

Figure S238 ^{1}H NMR spectrum of compound NPD-3298

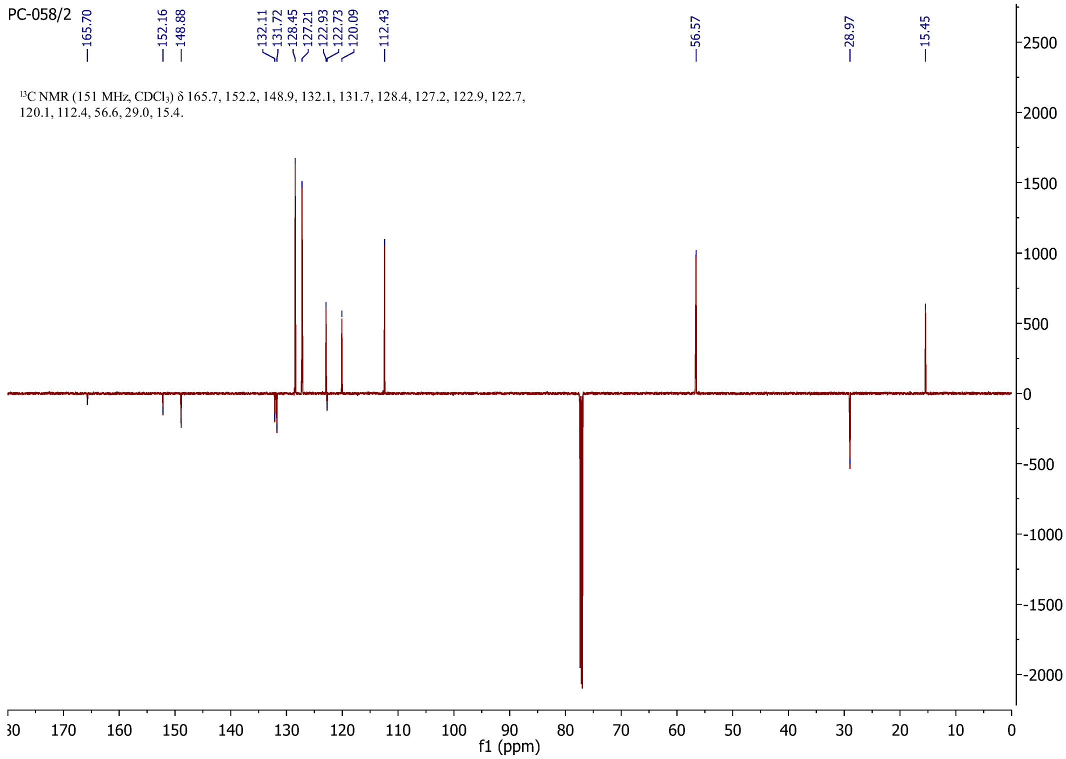

Figure S239 ^{13}C NMR spectrum of compound NPD-3298

Acquired by : Admin
Date Acquired : 26/2/2018 11:19:45 AM
Sample Name : PC-166
Sample ID :
Tray# : 1
Vial# : 10
Injection Volume : 1
Data File : C:\LabSolutions\Data\2018\2018-wk09\PC-166.lcd
Background File : Blanco_26022018.lcd
Method File : Method SCAN ACID standard.lcm
Report Format : DefaultLCMS.lcr
Tuning File : C:\LabSolutions\Tuning\Tuning-ESI-pos-neg01072015a.lct
Processed by : Admin
Modified Date : 26/2/2018 12:24:34 PM

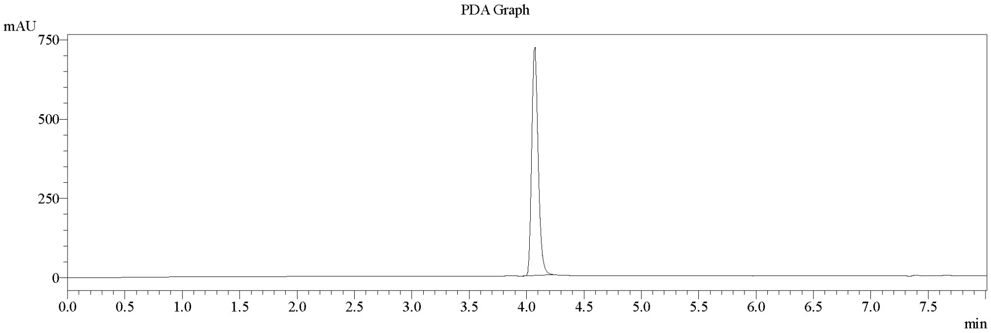

PDA Ch1 254nm 4nm

Peak#	Name	Ret. Time	Area	Area %
1		4.065	2796959	100.000

MS Spectrum Graph
#:1 Ret.Time:Averaged 4.120-4.140(Scan#:413-415)
Mass Peaks:6 Base Peak:322.05(298138) Polarity:Pos Segment1 - Event1

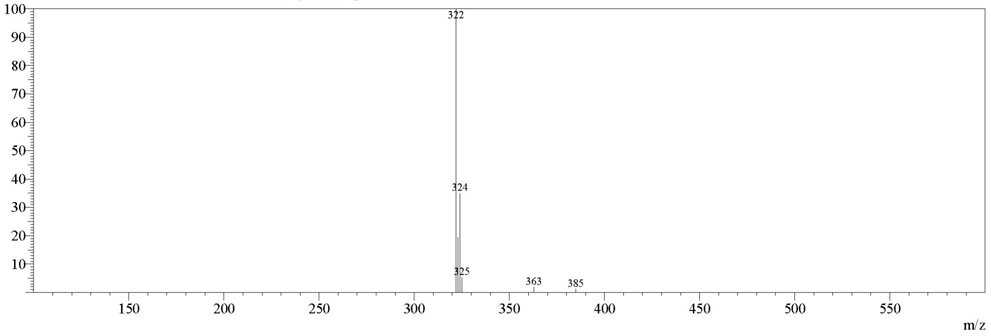

MS Spectrum Table
#:1 Ret.Time:
BG Mode:Calc 4.020<->4.310(403<->432)
Mass Peaks:6 Base Peak:322.05(298138) Polarity:Pos Segment1 - Event1

#	m/z	Abs.Inten.	Rel.Inten.	Charge	Polarity	Monoisotopic
1	322.05	298138	100.00			
2	323.05	57859	19.41			
3	324.05	104210	34.95			
4	325.05	15475	5.19			
5	363.05	5473	1.84			
6	384.95	3319	1.11			

Figure S240 LCMS spectrum of compound NPD-3299

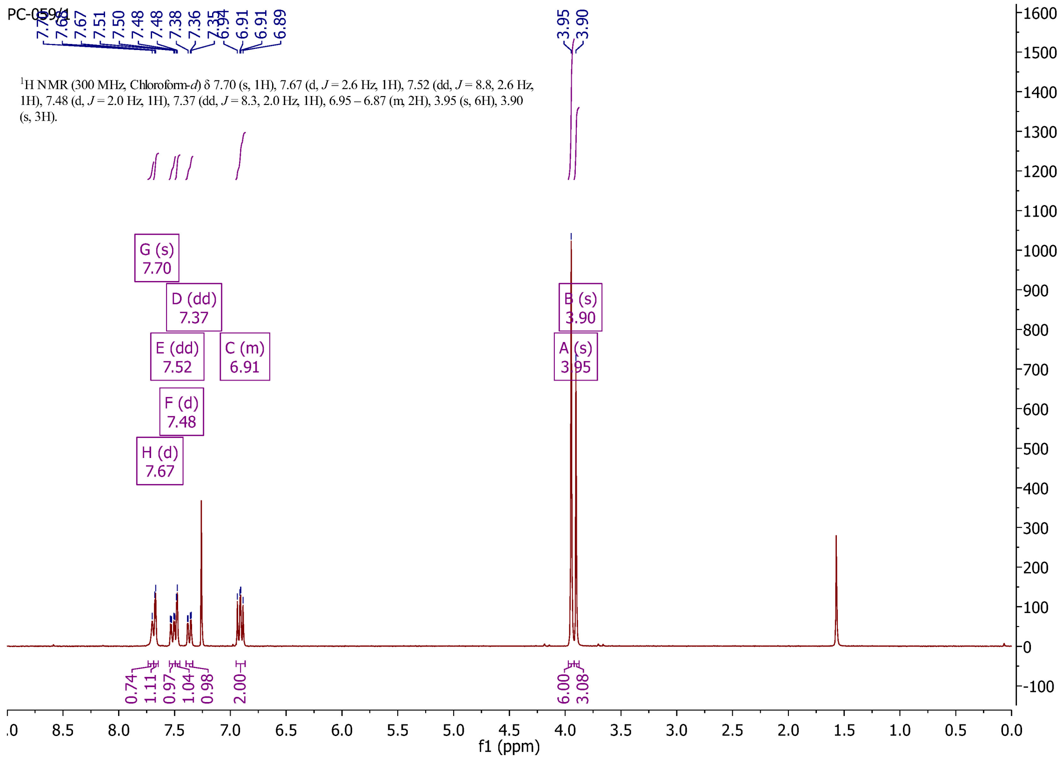

Figure S241 ^{1}H NMR spectrum of compound NPD-3299

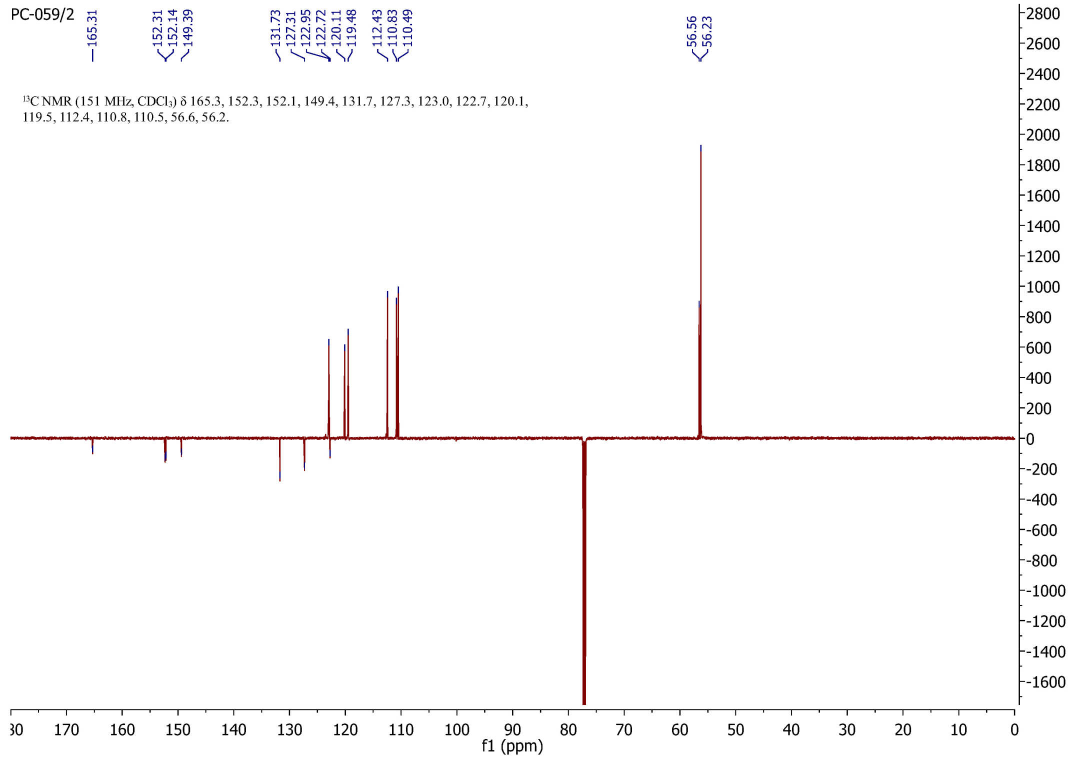

Figure S242 ^{13}C NMR spectrum of compound NPD-3299

Acquired by	: Admin
Date Acquired	: 26/2/2018 11:36:57 AM
Sample Name	: PC-167
Sample ID	:
Tray#	: 1
Vial#	: 11
Injection Volume	: 1
Data File	: C:\LabSolutions\Data\2018\2018-wk09\PC-167.lcd
Background File	: Blanco_26022018.lcd
Method File	: Method SCAN ACID standard.lcm
Report Format	: DefaultLCMS.lcr
Tuning File	: C:\LabSolutions\Tuning\Tuning-ESI-pos-neg01072015a.lct
Processed by	: Admin
Modified Date	: 26/2/2018 12:25:13 PM

PDA Graph

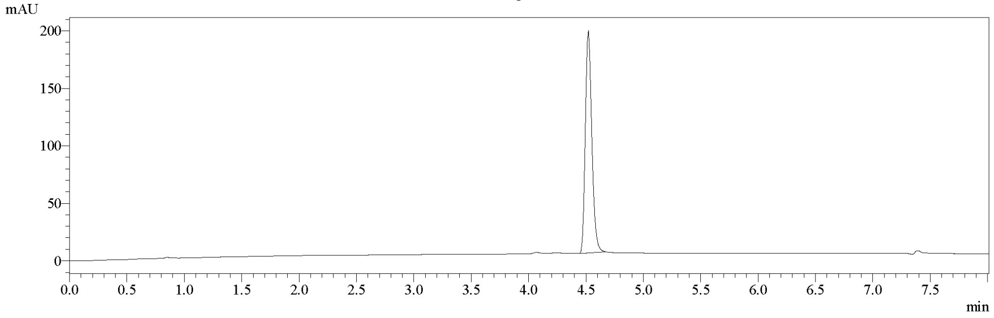

PDA Ch1 254nm 4nm

Peak#	Name	Ret. Time	Area	Area %
1		4.515	752916	100.000

MS Spectrum Graph

#:1 Ret.Time:Averaged 4.570-4.590(Scan#:458-460)
Mass Peaks:5 Base Peak:306.00(133343) Polarity:Pos Segment1 - Event1

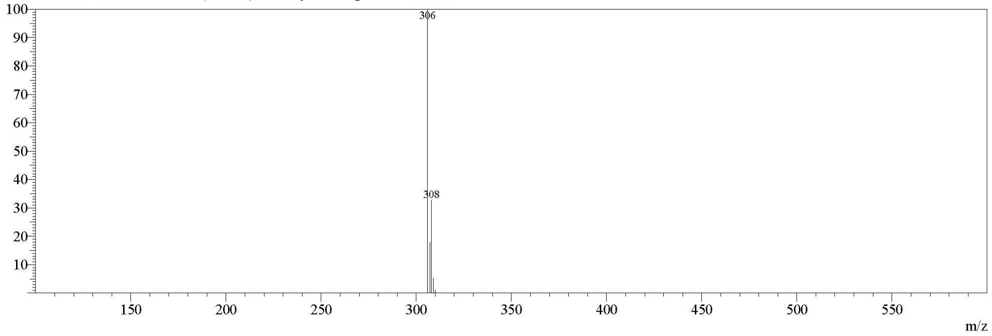

MS Spectrum Table

#:1 Ret.Time:
BG Mode:Calc 4.470<->4.710(448<->472)
Mass Peaks:5 Base Peak:306.00(133343) Polarity:Pos Segment1 - Event1

#	m/z	Abs.Inten.	Rel.Inten.	Charge	Polarity	Monoisotopic
1	306.00	133343	100.00			
2	307.05	23782	17.84			
3	308.05	43538	32.65			

#	m/z	Abs.Inten.	Rel.Inten.	Charge	Polarity	Monoisotopic
4	309.05	7035	5.28			
5	310.05	1413	1.06			

Figure S243 LCMS spectrum of compound NPD-3300

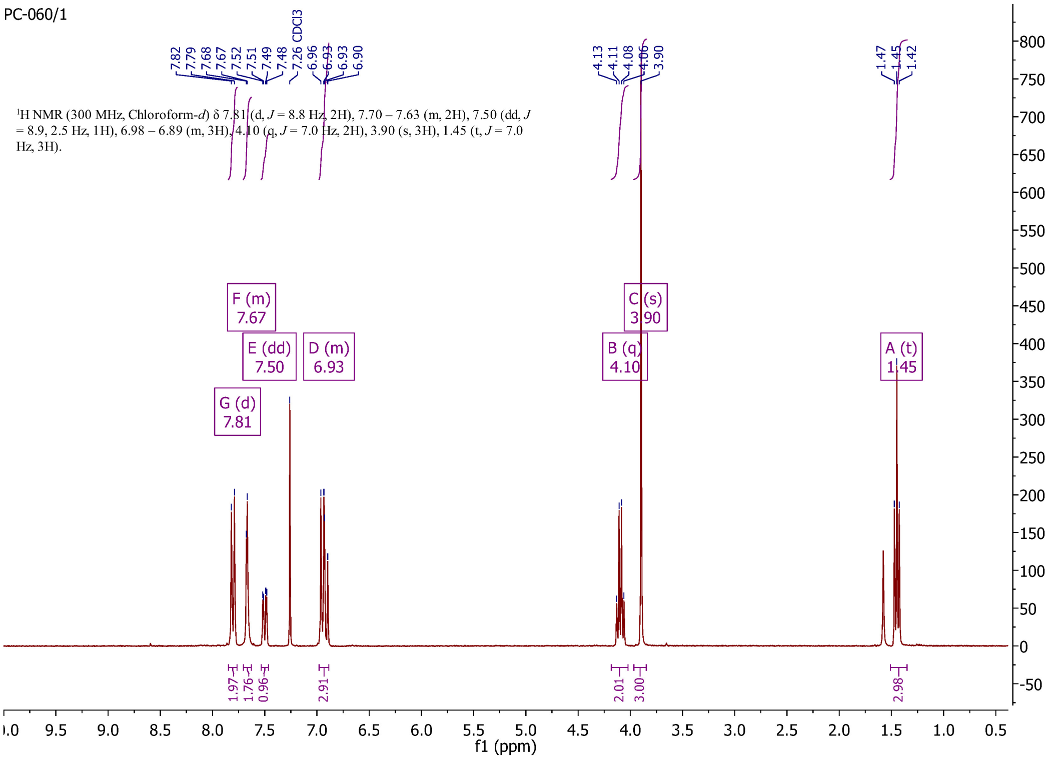

Figure S244 ^{1}H NMR spectrum of compound NPD-3300

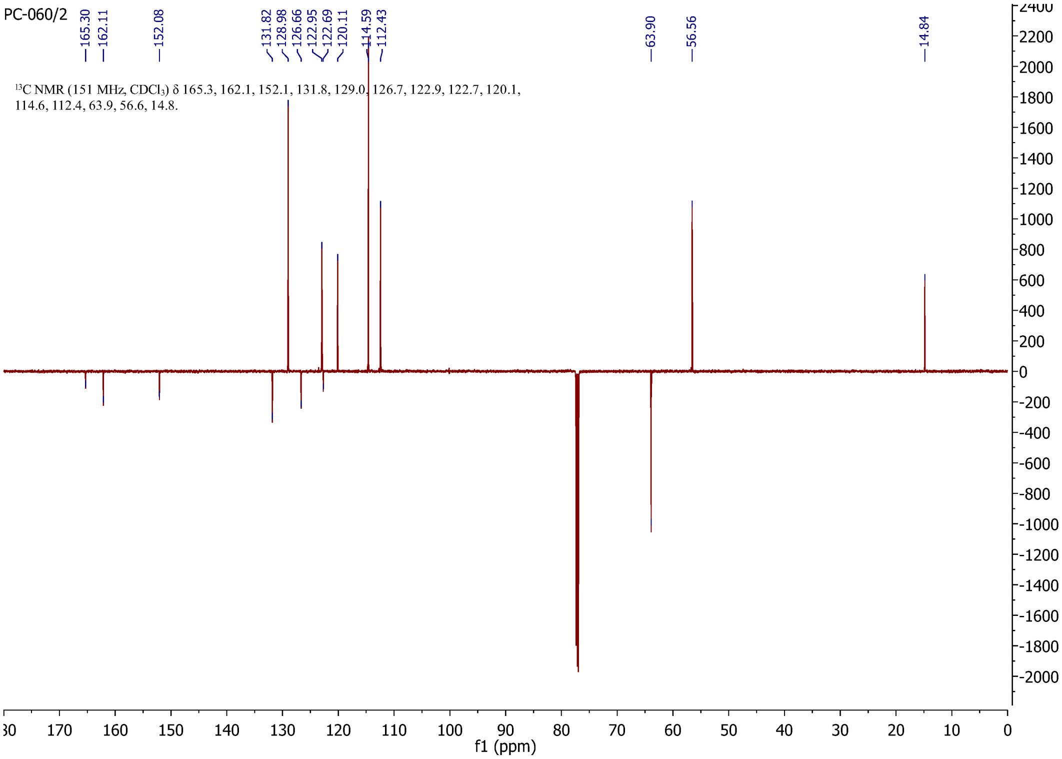

Figure S245 ^{13}C NMR spectrum of compound NPD-3300

Acquired by	: Admin
Date Acquired	: 26/2/2018 12:45:53 PM
Sample Name	: PC-168
Sample ID	:
Tray#	: 1
Vial#	: 17
Injection Volume	: 3
Data File	: C:\LabSolutions\Data\2018\2018-wk09\PC-168.lcd
Background File	: Blanco_26022018.lcd
Method File	: Method SCAN ACID standard.lcm
Report Format	: DefaultLCMS.lcr
Tuning File	: C:\LabSolutions\Tuning\Tuning-ESI-pos-neg01072015a.lct
Processed by	: Admin
Modified Date	: 26/2/2018 1:36:46 PM

PDA Graph

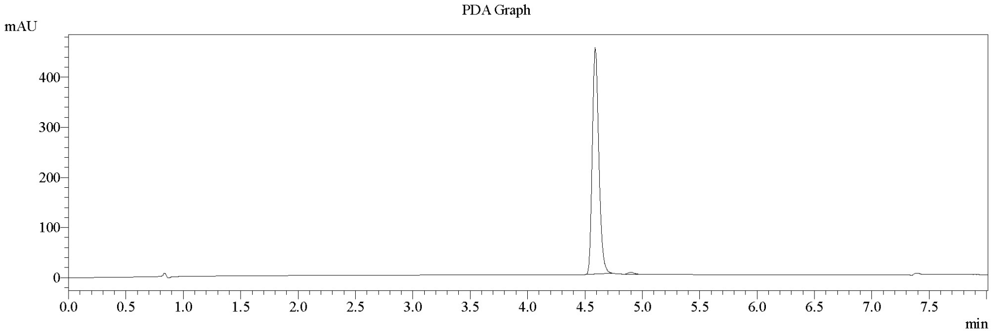

PDA Ch1 254nm 4nm

Peak#	Name	Ret. Time	Area	Area %
1		4.585	1795958	99.263
2		4.895	13330	0.737

MS Spectrum Graph

#:1 Ret.Time:Averaged 4.630-4.650(Scan#:464-466)
Mass Peaks:9 Base Peak:322.05(295796) Polarity:Pos Segment1 - Event1

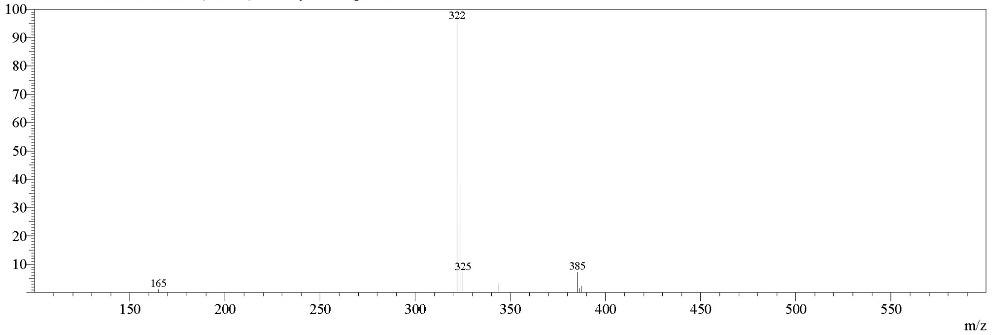

MS Spectrum Table

#:1 Ret.Time:
BG Mode:Calc 4.530<->4.810(454<->482)
Mass Peaks:9 Base Peak:322.05(295796) Polarity:Pos Segment1 - Event1

#	m/z	Abs.Inten.	Rel.Inten.	Charge	Polarity	Monoisotopic	#	m/z	Abs.Inten.	Rel.Inten.	Charge	Polarity	Monoisotopic
1	164.95	3424	1.16				6	344.05	9240	3.12			
2	322.05	295796	100.00				7	385.05	21226	7.18			
3	323.00	68808	23.26				8	386.15	4237	1.43			
4	323.95	112837	38.15				9	387.15	6883	2.33			
5	325.05	20903	7.07										

Figure S246 LCMS spectrum of compound NPD-3301

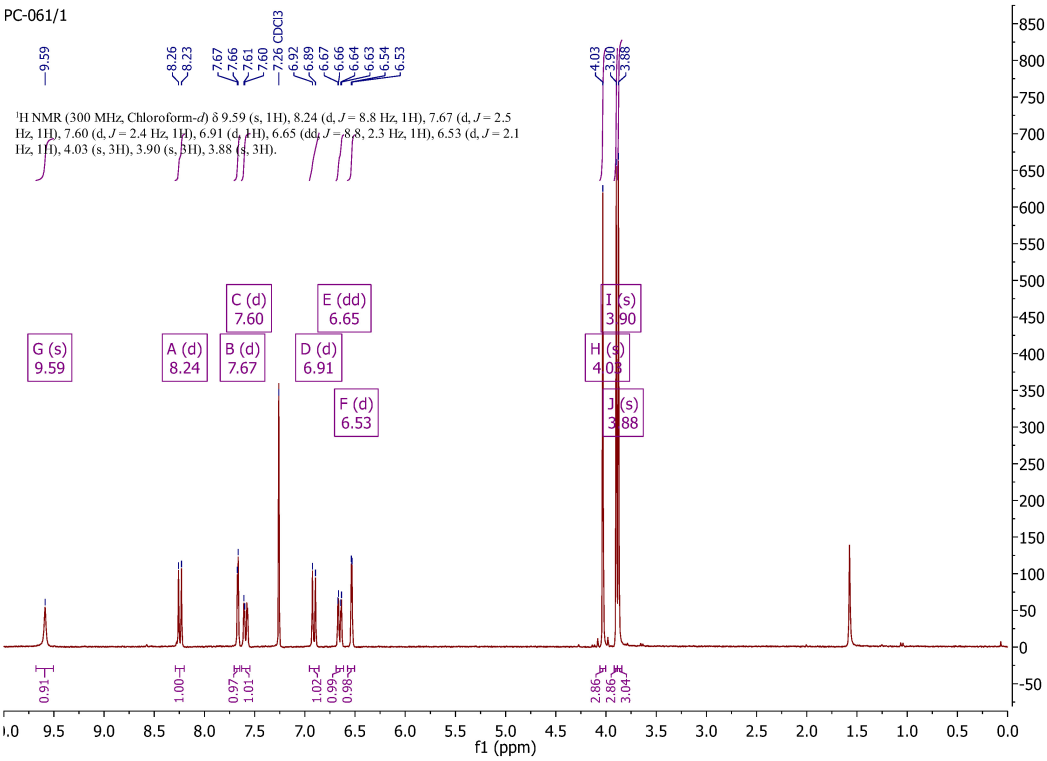

Figure S247 ^{1}H NMR spectrum of compound NPD-3301

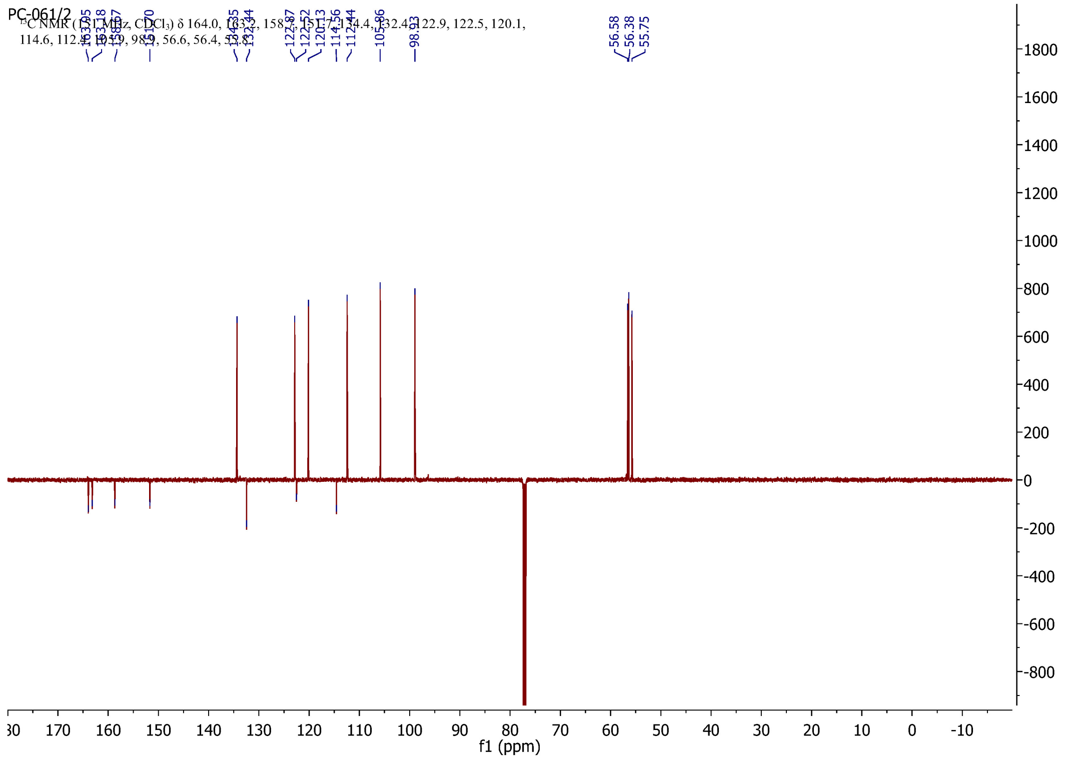

Figure S248 ^{13}C NMR spectrum of compound NPD-3301

Acquired by : Admin
Date Acquired : 26/2/2018 12:54:30 PM
Sample Name : PC-169
Sample ID :
Tray# : 1
Vial# : 18
Injection Volume : 3
Data File : C:\LabSolutions\Data\2018\2018-wk09\PC-169.lcd
Background File : Blanco_26022018.lcd
Method File : Method SCAN ACID standard.lcm
Report Format : DefaultLCMS.lcr
Tuning File : C:\LabSolutions\Tuning\Tuning-ESI-pos-neg01072015a.lct
Processed by : Admin
Modified Date : 26/2/2018 1:37:38 PM

PDA Graph

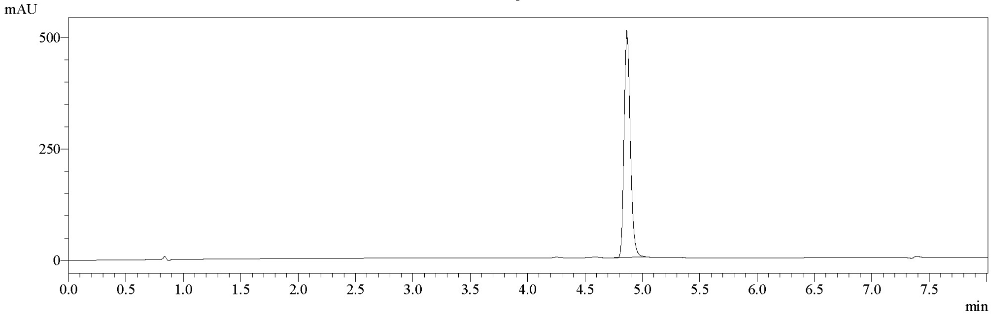

PDA Ch1 254nm 4nm

Peak#	Name	Ret. Time	Area	Area %
1		4.860	1917737	100.000

MS Spectrum Graph

#:1 Ret.Time:Averaged 4.910-4.930(Scan#:492-494)
Mass Peaks:8 Base Peak:346.00(57689) Polarity:Pos Segment1 - Event1

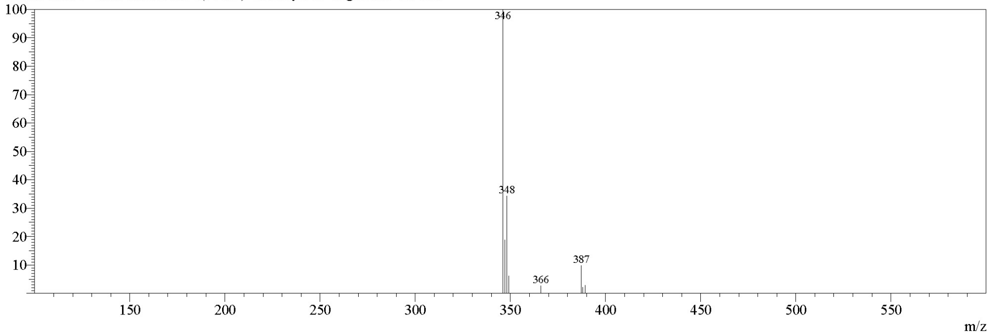

MS Spectrum Table

#:1 Ret.Time:
BG Mode:Calc 4.810<->5.090(482<->510)
Mass Peaks:8 Base Peak:346.00(57689) Polarity:Pos Segment1 - Event1

#	m/z	Abs.Inten.	Rel.Inten.	Charge	Polarity	Monoisotopic
1	346.00	57689	100.00			
2	347.05	10904	18.90			
3	348.00	19782	34.29			
4	349.05	3498	6.06			
5	366.05	1538	2.67			
6	387.10	5665	9.82			
7	388.00	1231	2.13			
8	389.15	1638	2.84			

Figure S249 LCMS spectrum of compound NPD-3302

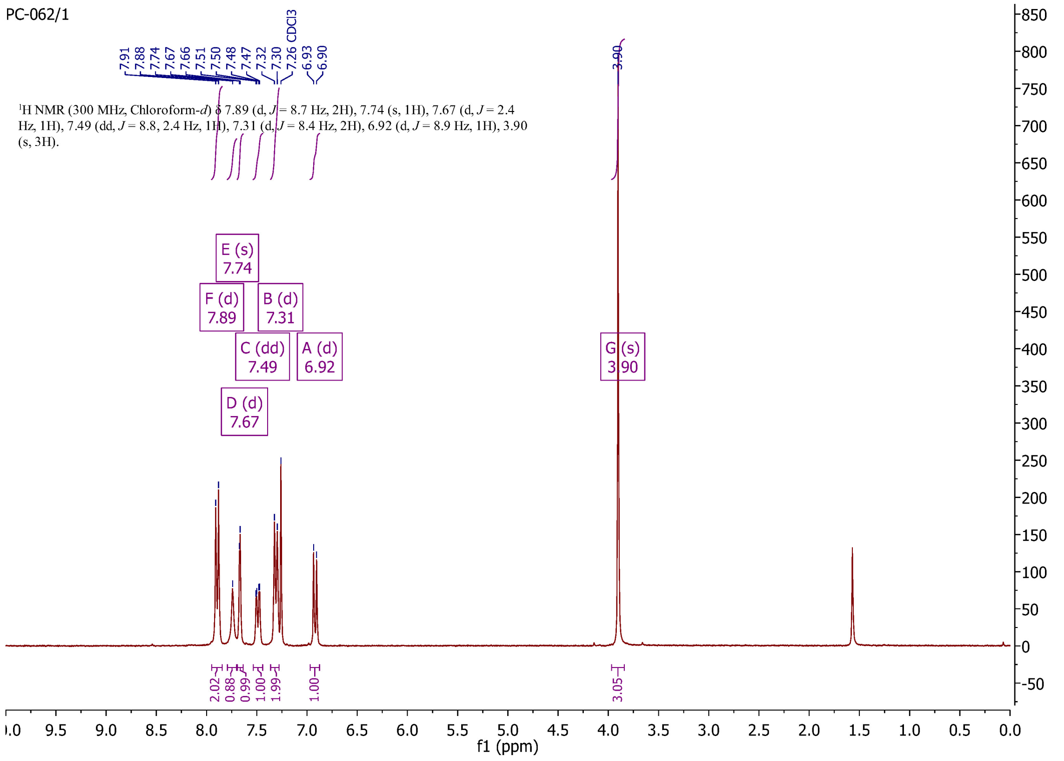

Figure S250 ^{1}H NMR spectrum of compound NPD-3302

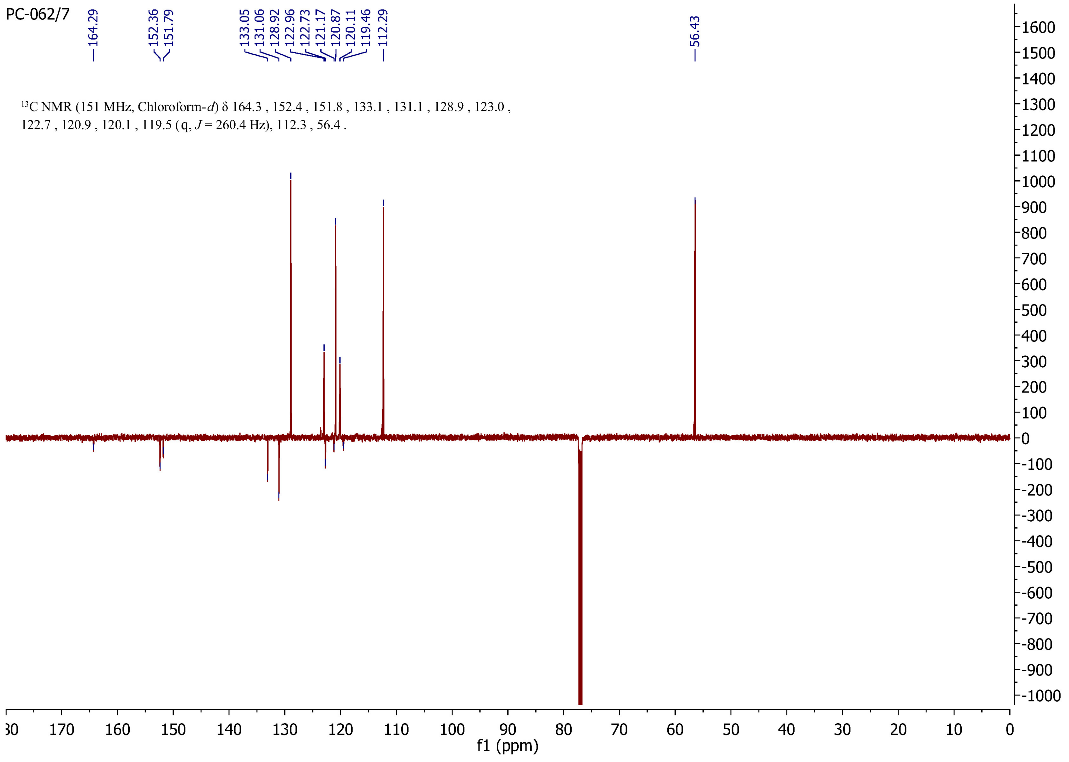

Figure S251 ^{13}C NMR spectrum of compound NPD-3302

Acquired by	: Admin
Date Acquired	: 28/2/2018 10:45:33 AM
Sample Name	: PC-170-3
Sample ID	:
Tray#	: 1
Vial#	: 6
Injection Volume	: 3
Data File	: C:\LabSolutions\Data\2018\2018-wk09\PC-170-3.lcd
Background File	: blanco 28022018.lcd
Method File	: Method SCAN ACID standard.lcm
Report Format	: DefaultLCMS.lcr
Tuning File	: C:\LabSolutions\Tuning\Tuning-ESI-pos-neg01072015a.lct
Processed by	: Admin
Modified Date	: 28/2/2018 11:27:33 AM

Medicinal Chemistry
Vrije Universiteit Amsterdam

Chromatogram
PC-170-3 C:\LabSolutions\Data\2018\2018-wk09\PC-170-3.lcd

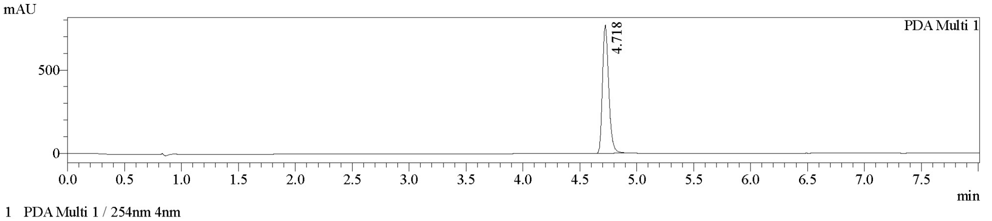

1 PDA Multi 1 / 254nm 4nm

PeakTable

PDA Ch1 254nm 4nm

Peak#	Name	Ret. Time	Area	Area %
1		4.718	2912045	100.000

MS Chromatogram
PC-170-3 C:\LabSolutions\Data\2018\2018-wk09\PC-170-3.lcd

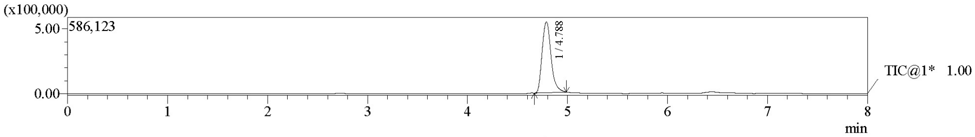

MS Spectrum Graph

#:1 Ret.Time:Averaged 4.780-4.800(Scan#:479-481)
Mass Peaks:6 Base Peak:320.05(355884) Polarity:Pos Segment1 - Event1

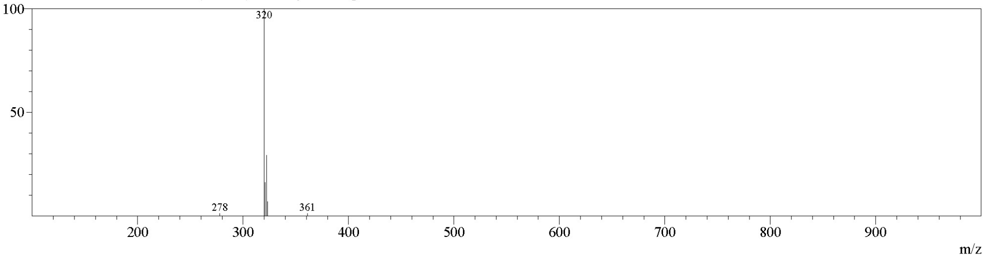

MS Spectrum Table

#:1 Ret.Time:
BG Mode:Calc 4.670<->4.990(468<->500)
Mass Peaks:6 Base Peak:320.05(355884) Polarity:Pos Segment1 - Event1

#	m/z	Abs.Inten.	Rel.Inten.	Charge	Polarity	Monoisotopic
1	278.00	4713	1.32			
2	320.05	355884	100.00			
3	321.05	57697	16.21			
4	322.05	104423	29.34			
5	323.05	24296	6.83			
6	361.15	4157	1.17			

Figure S252 LCMS spectrum of compound NPD-3303

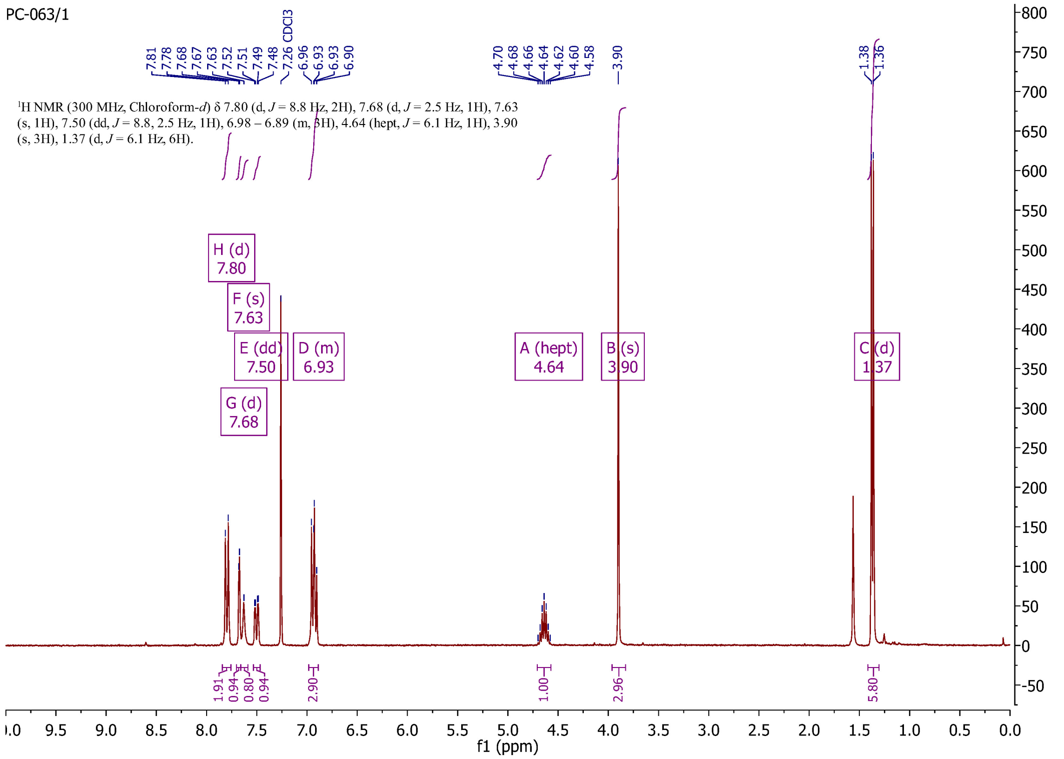

Figure S253 ^{1}H NMR spectrum of compound NPD-3303

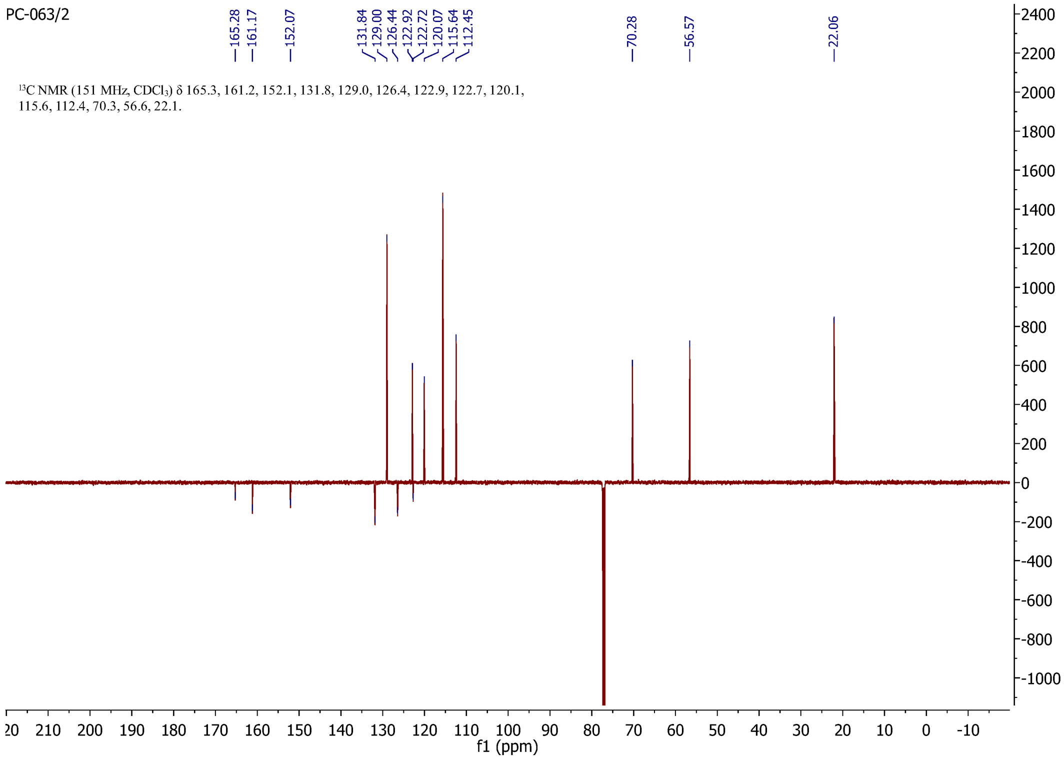

Figure S254 ^{13}C NMR spectrum of compound NPD-3303

Acquired by	: Admin
Date Acquired	: 26/2/2018 1:11:45 PM
Sample Name	: PC-172
Sample ID	:
Tray#	: 1
Vial#	: 20
Injection Volume	: 3
Data File	: C:\LabSolutions\Data\2018\2018-wk09\PC-172.lcd
Background File	: Blanco_26022018.lcd
Method File	: Method SCAN ACID standard.lcm
Report Format	: DefaultLCMS.lcr
Tuning File	: C:\LabSolutions\Tuning\Tuning-ESI-pos-neg01072015a.lct
Processed by	: Admin
Modified Date	: 26/2/2018 1:39:01 PM

PDA Graph

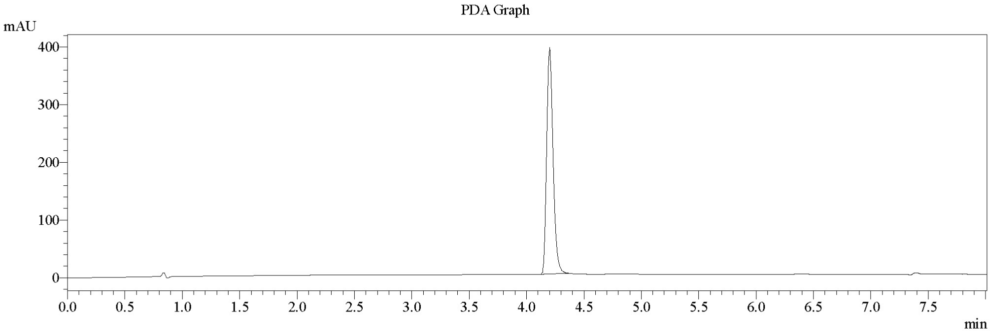

PDA Ch1 254nm 4nm

Peak#	Name	Ret. Time	Area	Area %
1		4.195	1506868	100.000

MS Spectrum Graph

#:1 Ret.Time:Single 4.270(Scan#:428)
Mass Peaks:7 Base Peak:287.05(16488) Polarity:Pos Segment1 - Event1

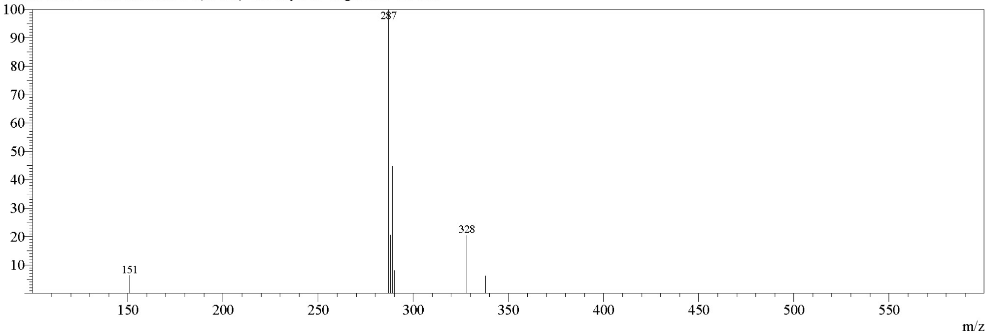

MS Spectrum Table

#:1 Ret.Time:
BG Mode:None
Mass Peaks:7 Base Peak:287.05(16488) Polarity:Pos Segment1 - Event1

#	m/z	Abs.Inten.	Rel.Inten.	Charge	Polarity	Monoisotopic
1	151.00	1034	6.27			
2	287.05	16488	100.00			
3	288.00	3385	20.53			
4	289.15	7381	44.77			
5	290.00	1334	8.09			
6	328.15	3365	20.41			
7	338.00	1021	6.19			

Figure S255 LCMS spectrum of compound NPD-3304

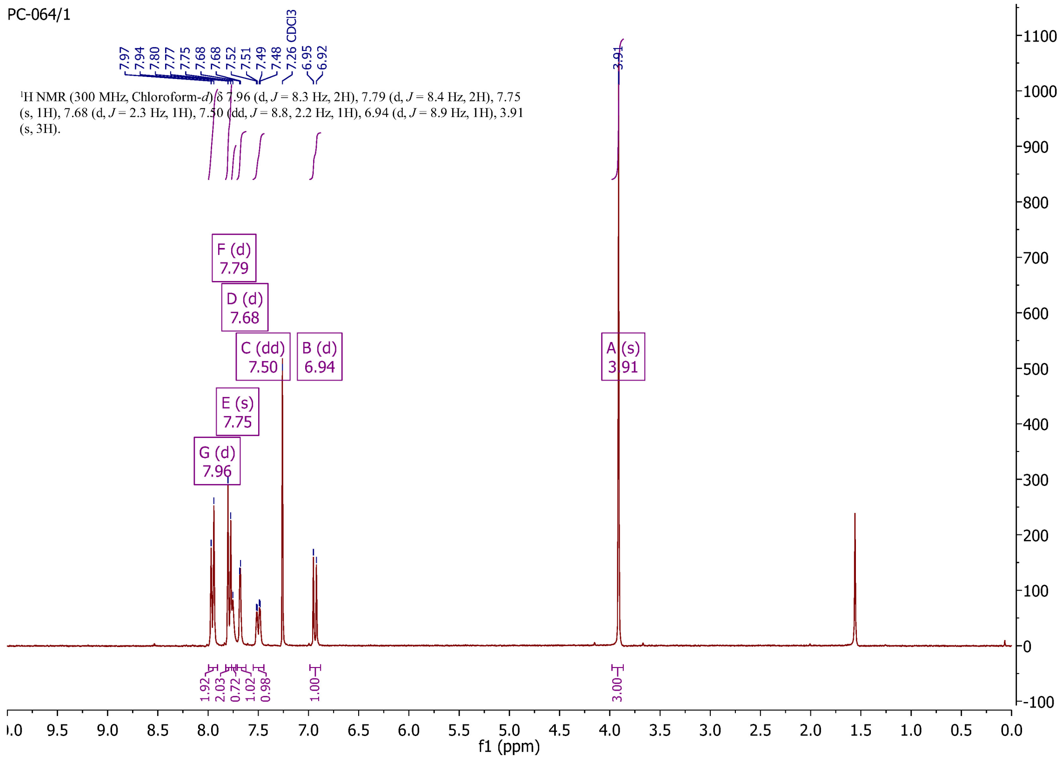

Figure S256 ^{1}H NMR spectrum of compound NPD-3304

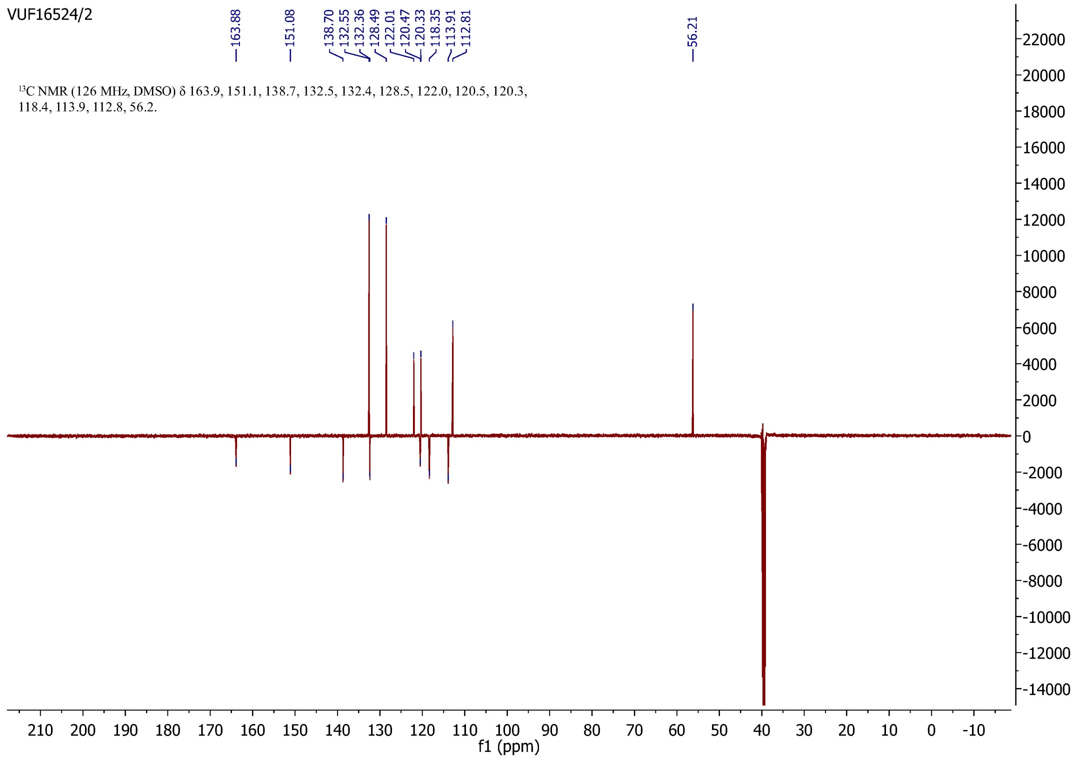

Figure S257 ^{13}C NMR spectrum of compound NPD-3304

Reference:

1. Ashton, M.J.; Cook, D.C.; Fenton, G.; Karlsson, J.A.; Palfreyman, M.N.; Raeburn, D.; Ratcliffe, A.J.; Souness, J.E.; Thurairatnam, S.; Vicker, N. Selective Type IV Phosphodiesterase Inhibitors as Antiasthmatic Agents. The Syntheses and Biological Activities of 3-(Cyclopentyloxy)-4-Methoxybenzamides and Analogues. *J Med Chem* **1994**, *37*, 1696–1703, doi:10.1021/jm00037a021.

2. Lin, Y.; Huang, P.; Liu, S.; Sima, L.; Chen, L.; Wang, D. A Convenient Method for the Synthesis of Roflumilast. *Research on Chemical Intermediates* **2013**, *39*, 2107–2113, doi:10.1007/s11164-012-0742-3.

3. Felding, J.; Sørensen, M.D.; Poulsen, T.D.; Larsen, J.; Andersson, C.; Refer, P.; Engell, K.; Ladefoged, L.G.; Thormann, T.; Vinggaard, A.M.; et al. Discovery and Early Clinical Development of 2-{6-[2-(3,5-Dichloro-4-Pyridyl)Acetyl]-2,3-Dimethoxyphenoxy}- N -Propylacetamide (LEO 29102), a Soft-Drug Inhibitor of Phosphodiesterase 4 for Topical Treatment of Atopic Dermatitis. *J Med Chem* **2014**, *57*, 5893–5903, doi:10.1021/jm500378a.

4. Bland, N.D.; Wang, C.; Tallman, C.; Gustafson, A.E.; Wang, Z.; Ashton, T.D.; Ochiana, S.O.; McAllister, G.; Cotter, K.; Fang, A.P.; et al. Pharmacological Validation of Trypanosoma Brucei Phosphodiesterases B1 and B2 as Druggable Targets for African Sleeping Sickness. *J Med Chem* **2011**, *54*, 8188–8194, doi:10.1021/jm201148s.

5. Yao, L.; Xiao, Z. The Preparation Method of Roflumilast N Oxide 2016.

6. Zhou, Z.Z.; Ge, B.C.; Zhong, Q.P.; Huang, C.; Cheng, Y.F.; Yang, X.M.; Wang, H.T.; Xu, J.P. Development of Highly Potent Phosphodiesterase 4 Inhibitors with Anti-Neuroinflammation Potential: Design, Synthesis, and Structure-Activity Relationship Study of Catecholamides Bearing Aromatic Rings. *Eur J Med Chem* **2016**, *124*, 372–379, doi:10.1016/j.ejmech.2016.08.052.

7. Freitag, A.; Wessier, I.; Racké, K. Phosphodiesterase Inhibitors Suppress A2-Adrenoceptor-Mediated 5-Hydroxytryptamine Release from Tracheae of Newborn Rabbits. *Eur J Pharmacol* **1998**, *354*, 67–71, doi:10.1016/S0014-2999(98)00439-7.

8. Francis, S.H.; Sekhar, K.R.; Ke, H.; Corbin, J.D. Inhibition of Cyclic Nucleotide Phosphodiesterases by Methylxanthines and Related Compounds BT - Methylxanthines. In; Fredholm, B.B., Ed.; Springer Berlin Heidelberg: Berlin, Heidelberg, 2011; pp. 93–133 ISBN 978-3-642-13443-2.

9. Thompson, W.J. Cyclic Nucleotide Phosphodiesterases: Pharmacology, Biochemistry and Function. *Pharmacol Ther* **1991**, *51*, 13–33, doi:10.1016/0163-7258(91)90039-O.

10. Menniti, F.S.; Faraci, W.S.; Schmidt, C.J. Phosphodiesterases in the CNS: Targets for Drug Development. *Nat Rev Drug Discov* **2006**, *5*, 660–670, doi:10.1038/nrd2058.

11. Bell, A.S.; Terrett, N.K. Pyrazolo[4,3-d]Pyrimidine Derivatives and Pharmaceutical Compositions Containing Them 1999.

12. Maurice, D.H.; Ke, H.; Ahmad, F.; Wang, Y.; Chung, J.; Manganiello, V.C. Advances in Targeting Cyclic Nucleotide Phosphodiesterases. *Nat Rev Drug Discov* **2014**, *13*, 290–314, doi:10.1038/nrd4228.

13. Ruppert, D.; Weithmann, K.U. HL 725, an Extremely Potent Inhibitor of Platelet Phosphodiesterase and Induced Platelet Aggregation in Vitro. *Life Sci* **1982**, *31*, 2037–2043, doi:10.1016/0024-3205(82)90095-9.

14. Sircar, I.; Steffen, R.P.; Bobowski, G.; Burke, S.E.; Newton, R.S.; Weishaar, R.E.; Bristol, J.A.; Evans, D.B. Cardiotonic Agents. 9. Synthesis and Biological Evaluation of a Series of (E)-4,5-Dihydro-6-[2-[4-(1H-Imidazol-1-Yl)Phenyl]Ethenyl]-3(2H)-Pyridazinones: A Novel Class of Compounds with Positive Inotropic,

Antithrombotic, and Vasodilatory Activities for the Treatment of Congestive Heart Failure. *J Med Chem* **1989**, *32*, 342–350, doi:10.1021/jm00122a011.

15. Sudo, T.; Tachibana, K.; Toga, K.; Tochizawa, S.; Inoue, Y.; Kimura, Y.; Hidaka, H. Potent Effects of Novel Anti-Platelet Aggregatory Cilostamide Analogues on Recombinant Cyclic Nucleotide Phosphodiesterase Isozyme Activity. *Biochem Pharmacol* **2000**, *59*, 347–356, doi:10.1016/S0006-2952(99)00346-9.

16. Mata, M.; Pallardo, F.; Morcillo, E.J.; Cortijo, J. Piclamilast Inhibits the Pro-Apoptotic and Anti-Proliferative Responses of A549 Cells Exposed to H2O2 via Mechanisms Involving AP-1 Activation. *Free Radic Res* **2012**, *46*, 690–699, doi:10.3109/10715762.2012.669040.

17. Sterk, G.J.; Hatzelmann, A.; Barsig, J.; Marx, D.; Kley, H.-P.; Christiaans, J.A.M.; Menge, W.M.P.B. Pyrrolidinedione Substituted Piperidine-Phthalazones as Pde4 Inhibitors 2004.

18. Vellas, B.; Sol, O.; J. Snyder, P.; Ousset, P.-J.; Haddad, R.; Maurin, M.; Lemarie, J.-C.; Desire, L.; P. Pando, M. EHT0202 in Alzheimers Disease: A 3-Month, Randomized, Placebo- Controlled, Double-Blind Study. *Curr Alzheimer Res* 2011, *8*, 203–212.

19. Sterk, G.J.; Hatzelmann, A.; Marx, D.; Steinhilber, W. Phthalazinones Derivatives Useful as Pde4/7 Inhibitors 2002.

20. de Koning, H.P.; Gould, M.K.; Sterk, G.J.; Tenor, H.; Kunz, S.; Luginbuehl, E.; Seebeck, T. Pharmacological Validation of Trypanosoma Brucei Phosphodiesterases as Novel Drug Targets. *J Infect Dis* **2012**, *206*, 229–237, doi:10.1093/infdis/jir857.

21. Orrling, K.M.; Jansen, C.; Vu, X.L.; Balmer, V.; Bregy, P.; Shanmugham, A.; England, P.; Bailey, D.; Cos, P.; Maes, L.; et al. Catechol Pyrazolinones as Trypanocidals: Fragment-Based Design, Synthesis, and Pharmacological Evaluation of Nanomolar Inhibitors of Trypanosomal Phosphodiesterase B1. *J Med Chem* **2012**, *55*, 8745–8756, doi:10.1021/jm301059b.

22. Weinbrenner, S.; Dunkern, T.; Marx, D.; Schmidt, B.; Stenget, T.; Flockerzi, D.; Kautz, U.; Hauser, D.; Diefenbach, J.; Christiaans, J.A.M.; et al. 6-Benzyl-2,3,4,7-Tetrahydro-Indolo[2,3-c]Quinoline Compounds Useful as Pde5 Inhibitors 2008.

23. Haning, H.; Niewöhner, U.; Schenke, T.; Lampe, T.; Hillisch, A.; Bischoff, E. Comparison of Different Heterocyclic Scaffolds as Substrate Analog PDE5 Inhibitors. *Bioorg Med Chem Lett* **2005**, *15*, 3900–3907, doi:10.1016/j.bmcl.2005.05.090.

24. Choi, S.-H.; Choi, D.-H.; Song, K.-S.; Shin, K.-H.; Chun, B.-G. Zaprinast, an Inhibitor of CGMP-Selective Phosphodiesterases, Enhances the Secretion of TNF-α and IL-1β and the Expression of INOS and MHC Class II Molecules in Rat Microglial Cells. *J Neurosci Res* **2002**, *67*, 411–421, doi:https://doi.org/10.1002/jnr.10102.

25. Smith, S.J.; Cieslinski, L.B.; Newton, R.; Donnelly, L.E.; Fenwick, P.S.; Nicholson, A.G.; Barnes, P.J.; Barnette, M.S.; Giembycz, M.A. Discovery of BRL 50481 [3-(<Em>N,N</Em>-Dimethylsulfonamido)-4-Methyl-Nitrobenzene], a Selective Inhibitor of Phosphodiesterase 7: In Vitro Studies in Human Monocytes, Lung Macrophages, and CD8⁺ T-Lymphocytes. *Mol Pharmacol* **2004**, *66*, 1679 LP – 1689, doi:10.1124/mol.104.002246.

26. Redondo, M.; Brea, J.; Perez, D.I.; Soteras, I.; Val, C.; Perez, C.; Morales-García, J.A.; Alonso-Gil, S.; Paul-Fernandez, N.; Martin-Alvarez, R.; et al. Effect of Phosphodiesterase 7 (PDE7) Inhibitors in Experimental Autoimmune Encephalomyelitis Mice. Discovery of a New Chemically Diverse Family of Compounds. *J Med Chem* **2012**, *55*, 3274–3284, doi:10.1021/jm201720d.

27. García, A.M.; Brea, J.; Morales-García, J.A.; Perez, D.I.; González, A.; Alonso-Gil, S.; Gracia-Rubio, I.; Ros-Simó, C.; Conde, S.; Cadavid, M.I.; et al. Modulation of CAMP-Specific PDE without Emetogenic Activity: New Sulfide-Like PDE7 Inhibitors. *J Med Chem* **2014**, *57*, 8590–8607, doi:10.1021/jm501090m.

28. DeNinno, M.P.; Wright, S.W.; Visser, M.S.; Etienne, J.B.; Moore, D.E.; Olson, T. v.; Rocke, B.N.; Andrews, M.P.; Zarbo, C.; Millham, M.L.; et al. 1,5-Substituted Nipecotic Amides: Selective PDE8 Inhibitors Displaying Diastereomer-Dependent Microsomal Stability. *Bioorg Med Chem Lett* **2011**, *21*, 3095–3098, doi:10.1016/J.BMCL.2011.03.022.

29. Howard, B.L.; Harvey, K.L.; Stewart, R.J.; Azevedo, M.F.; Crabb, B.S.; Jennings, I.G.; Sanders, P.R.; Manallack, D.T.; Thompson, P.E.; Tonkin, C.J.; et al. Identification of Potent Phosphodiesterase Inhibitors That Demonstrate Cyclic Nucleotide-Dependent Functions in Apicomplexan Parasites. *ACS Chem Biol* **2015**, *10*, 1145–1154, doi:10.1021/cb501004q.

30. Blaazer, A.R.; Singh, A.K.; De Heuvel, E.; Edink, E.; Orrling, K.M.; Veerman, J.J.N.; Van Den Bergh, T.; Jansen, C.; Balasubramaniam, E.; Mooij, W.J.; et al. Targeting a Subpocket in Trypanosoma Brucei Phosphodiesterase B1 (TbrPDEB1) Enables the Structure-Based Discovery of Selective Inhibitors with Trypanocidal Activity. *J Med Chem* **2018**, *61*, 3870–3888, doi:10.1021/acs.jmedchem.7b01670.

31. Malamas, M.S.; Ni, Y.; Erdei, J.; Stange, H.; Schindler, R.; Lankau, H.-J.; Grunwald, C.; Fan, K.Y.; Parris, K.; Langen, B.; et al. Highly Potent, Selective, and Orally Active Phosphodiesterase 10A Inhibitors. *J Med Chem* **2011**, *54*, 7621–7638, doi:10.1021/jm2009138.

32. Ceyhan, O.; Birsoy, K.; Hoffman, C.S. Identification of Biologically Active PDE11-Selective Inhibitors Using a Yeast-Based High-Throughput Screen. *Chem Biol* **2012**, *19*, 155–163, doi:10.1016/J.CHEMBIOL.2011.12.010.

33. Rose, A.S.; Bradley, A.R.; Valasatava, Y.; Duarte, J.M.; Prlić, A.; Rose, P.W. NGL Viewer: Web-Based Molecular Graphics for Large Complexes. *Bioinformatics* **2018**, *34*, 3755–3758, doi:10.1093/bioinformatics/bty419.
